# Supplementary figures and images for: Intraobserver and interobserver agreement among anterior chamber angle evaluations using automated 360-degree gonio-photos
Source: PLoS One. 2021 May 6;16(5):e0251249. doi: 10.1371/journal.pone.0251249 (PMC8101769; doi:10.1371/journal.pone.0251249)

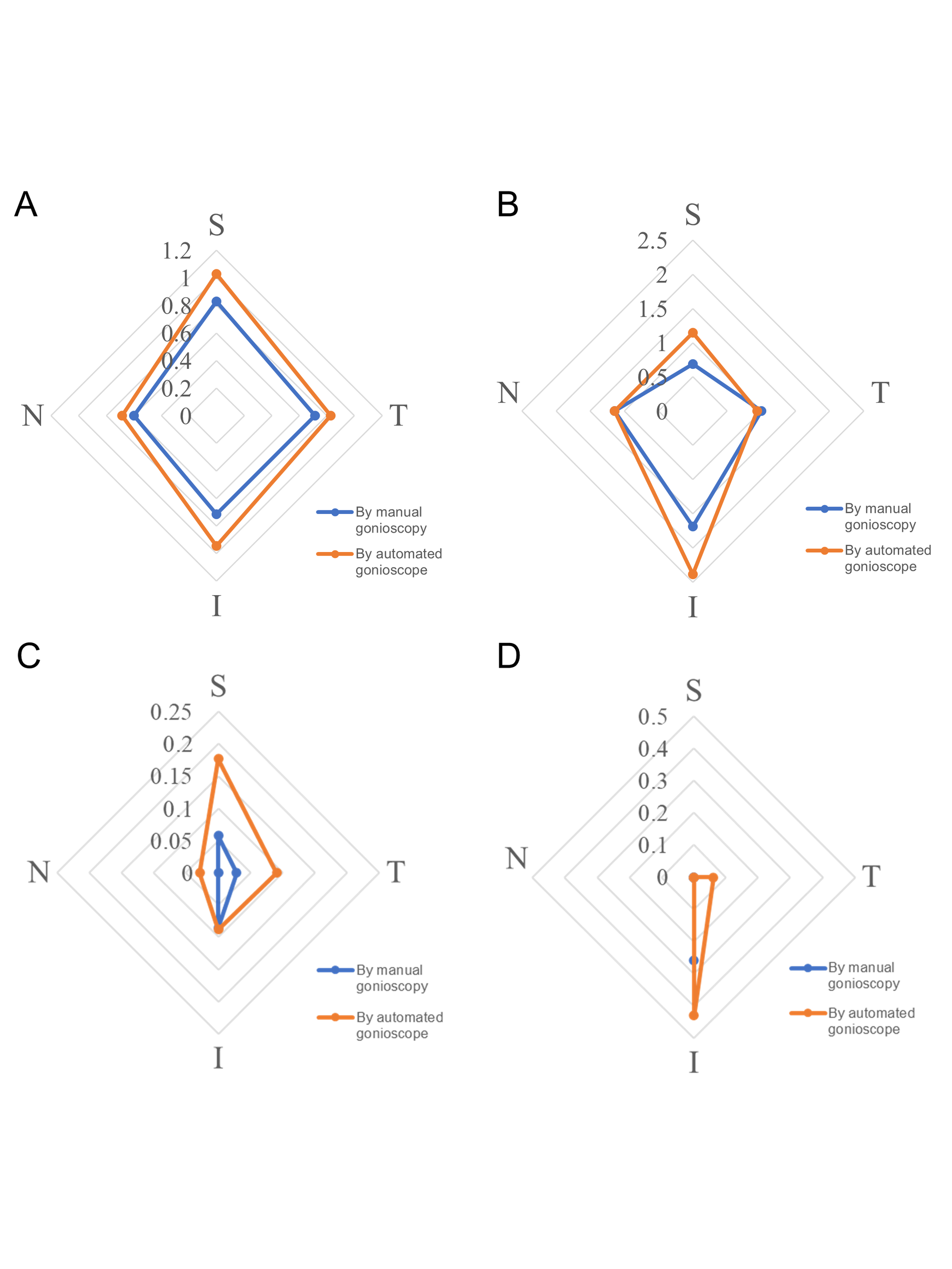

Supplement: S1 Fig — (TIF) [file pone.0251249.s001.tif]

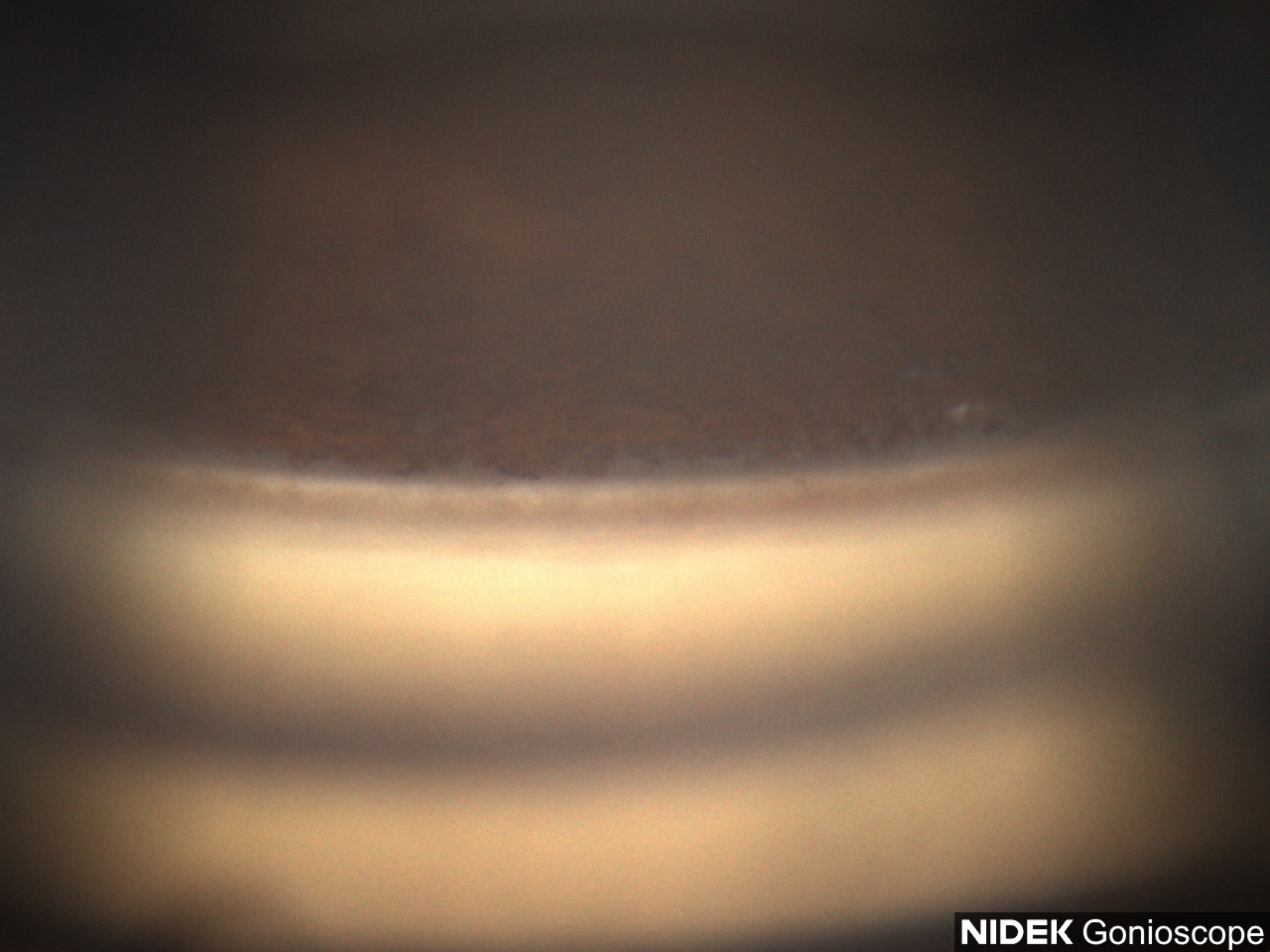

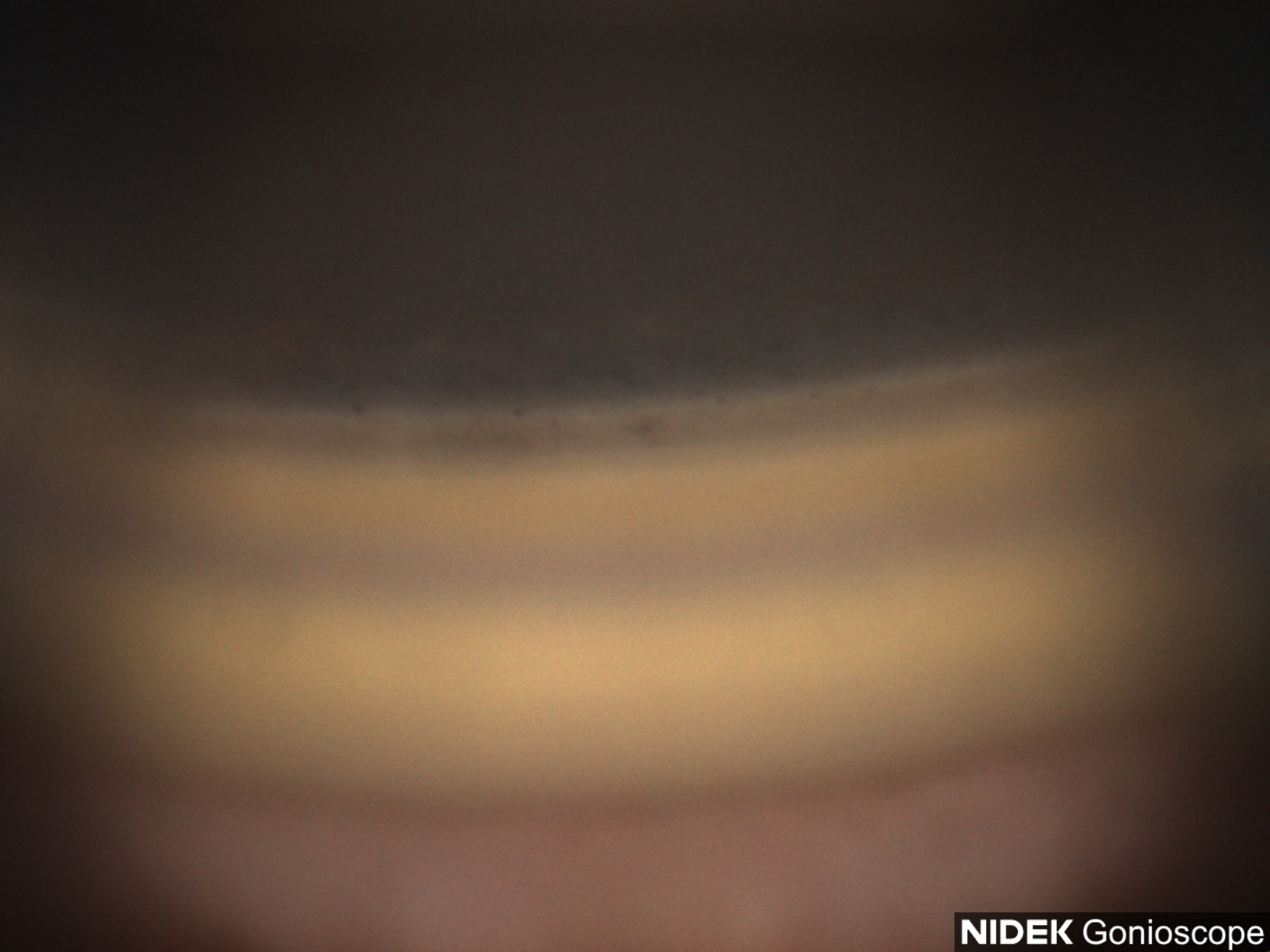

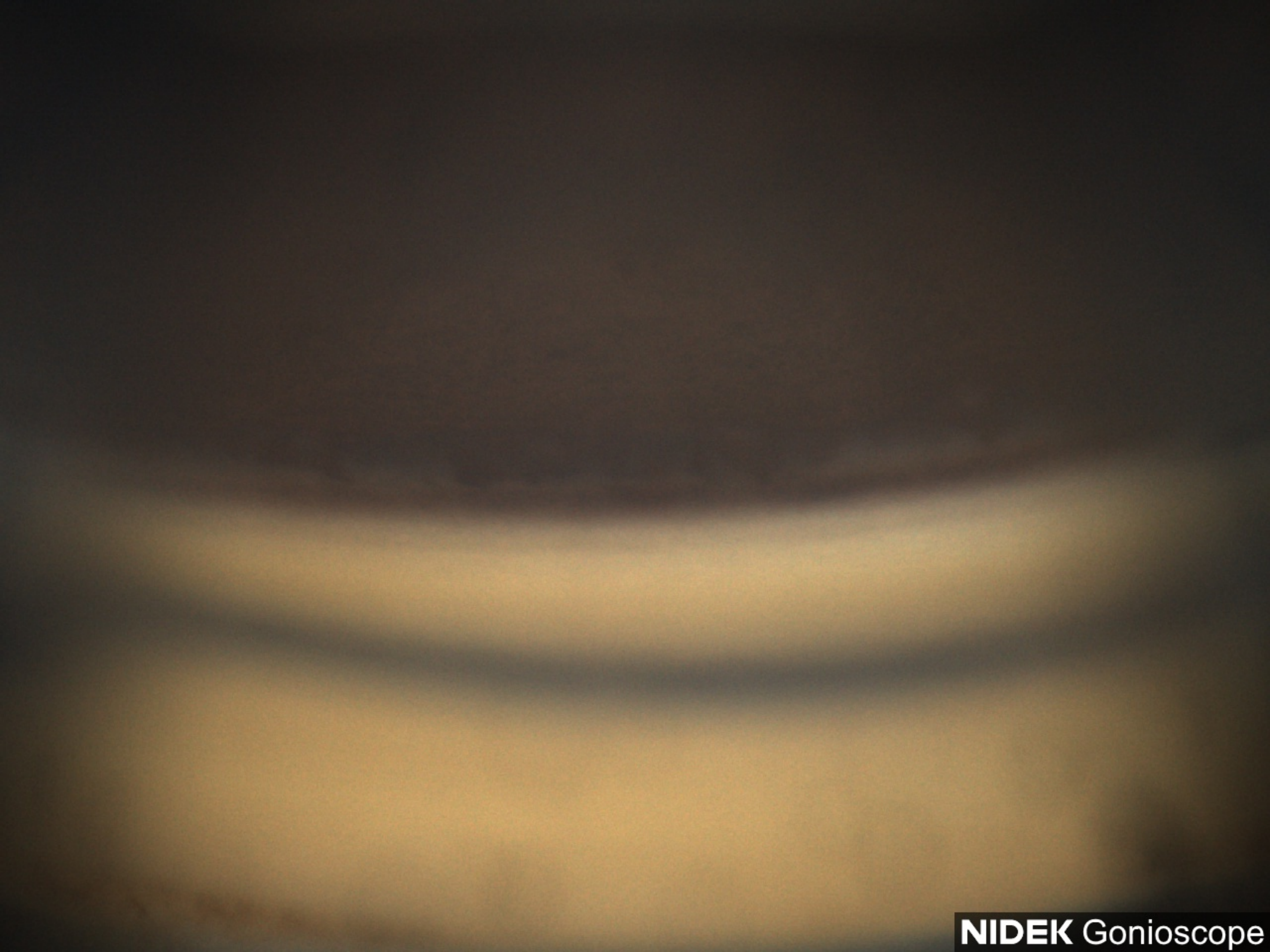

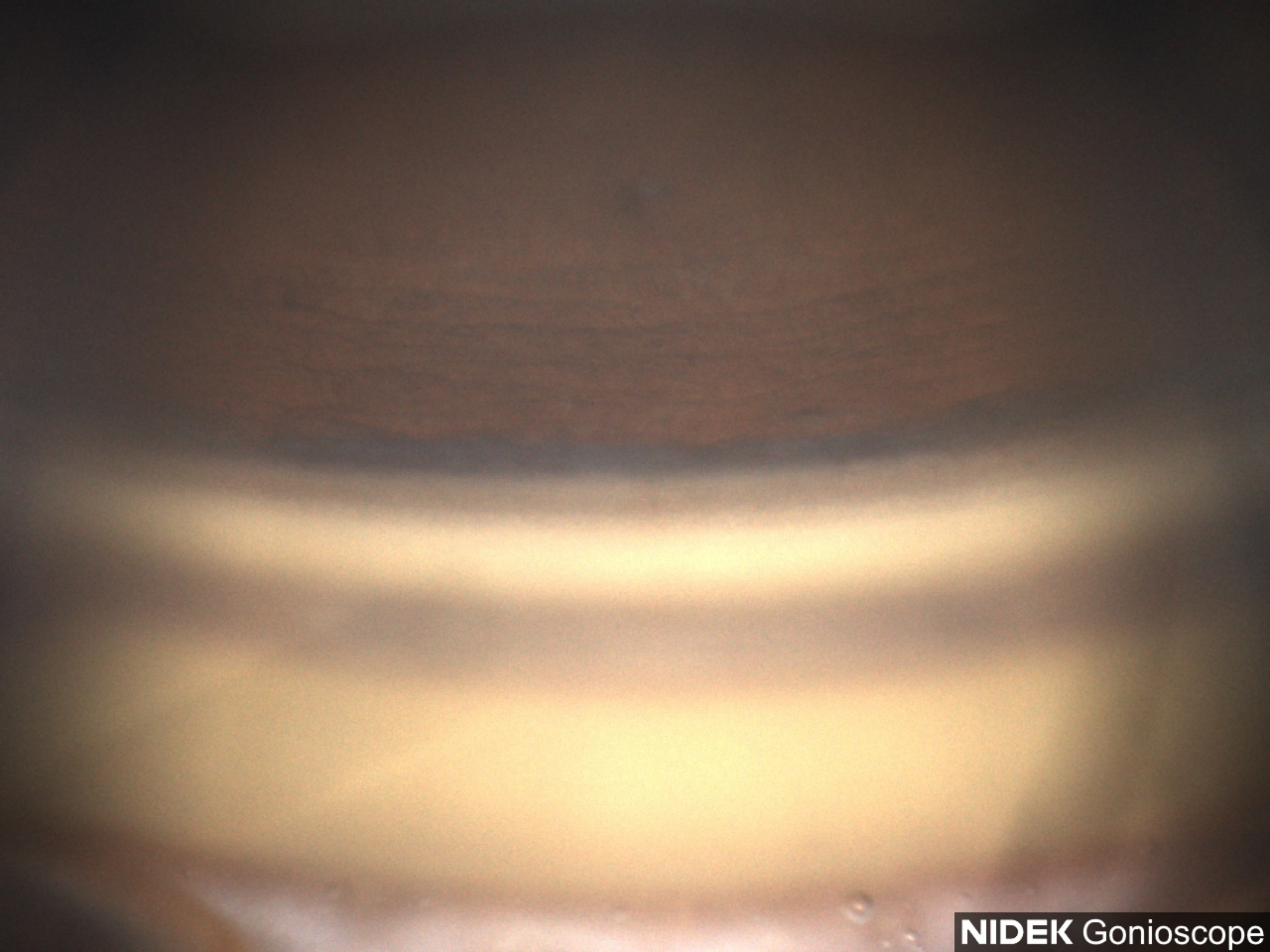

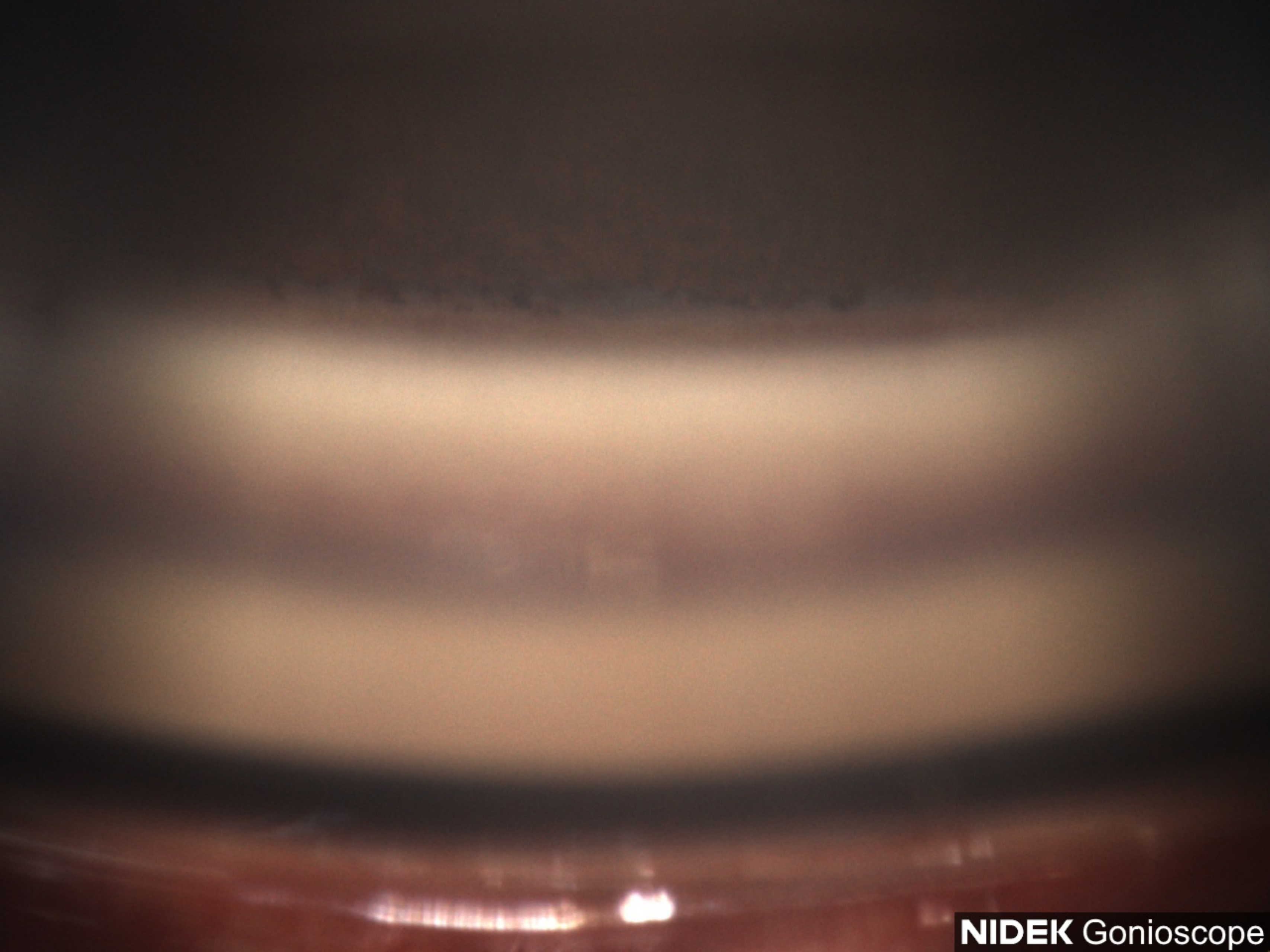

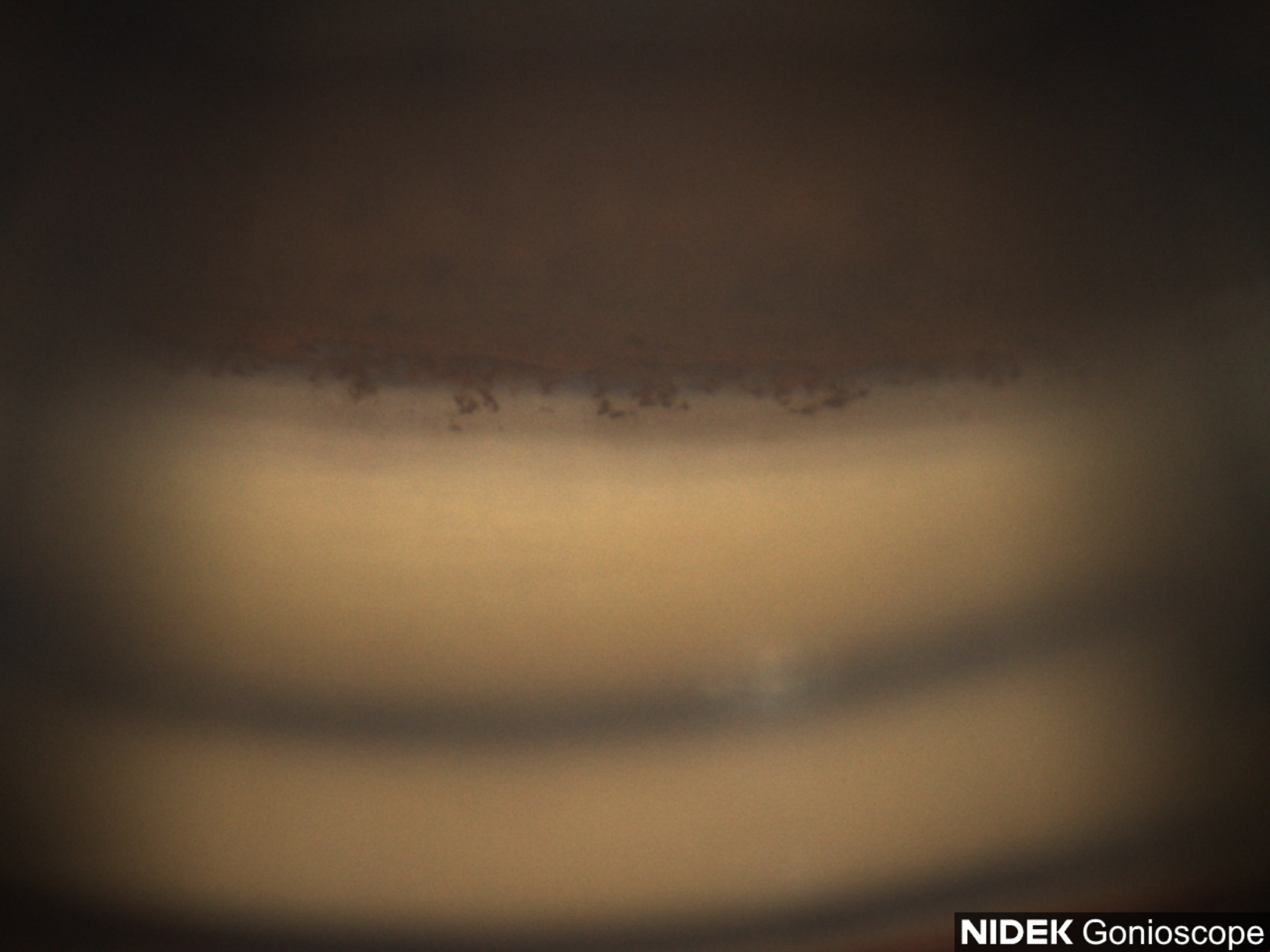

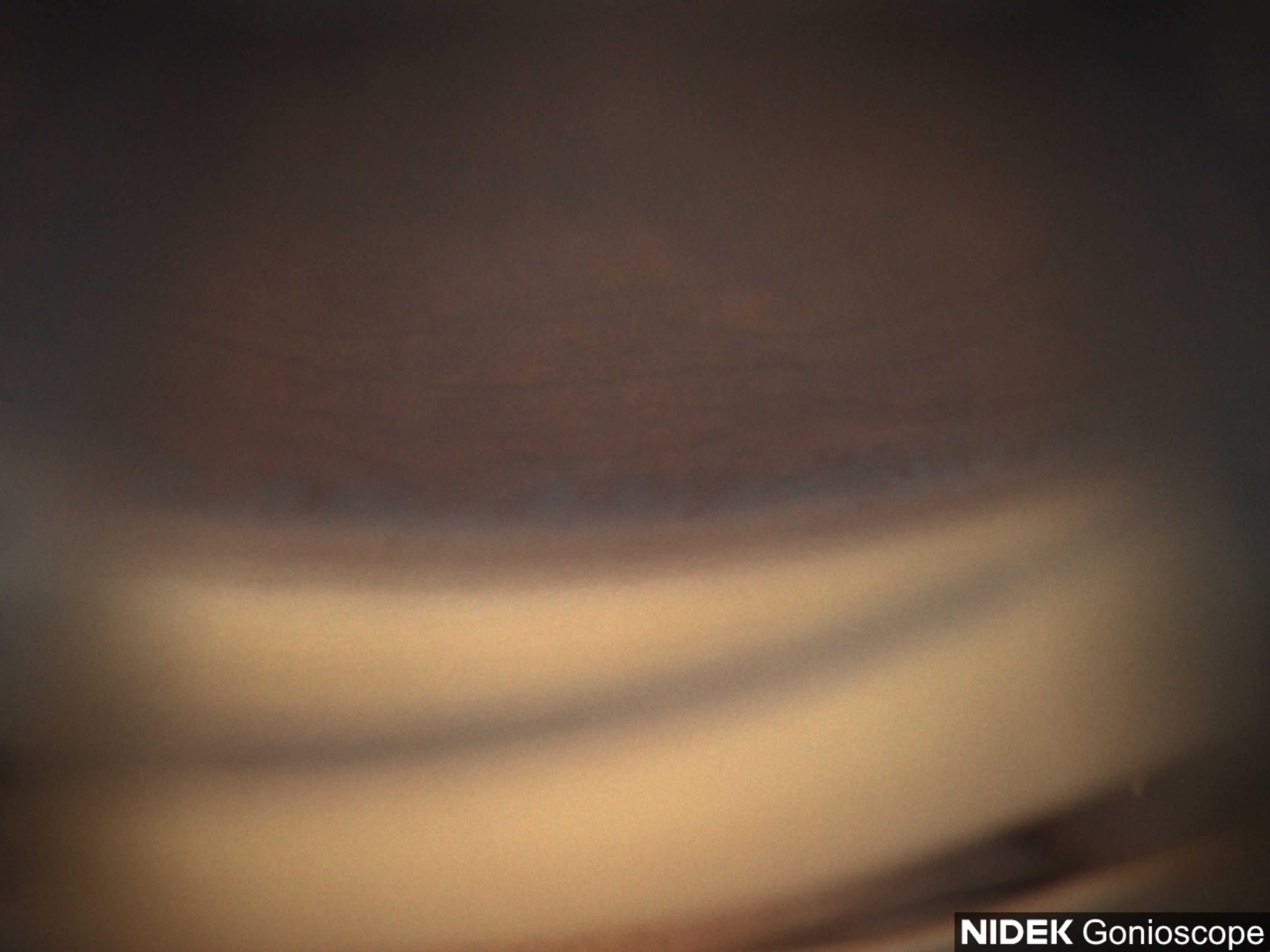

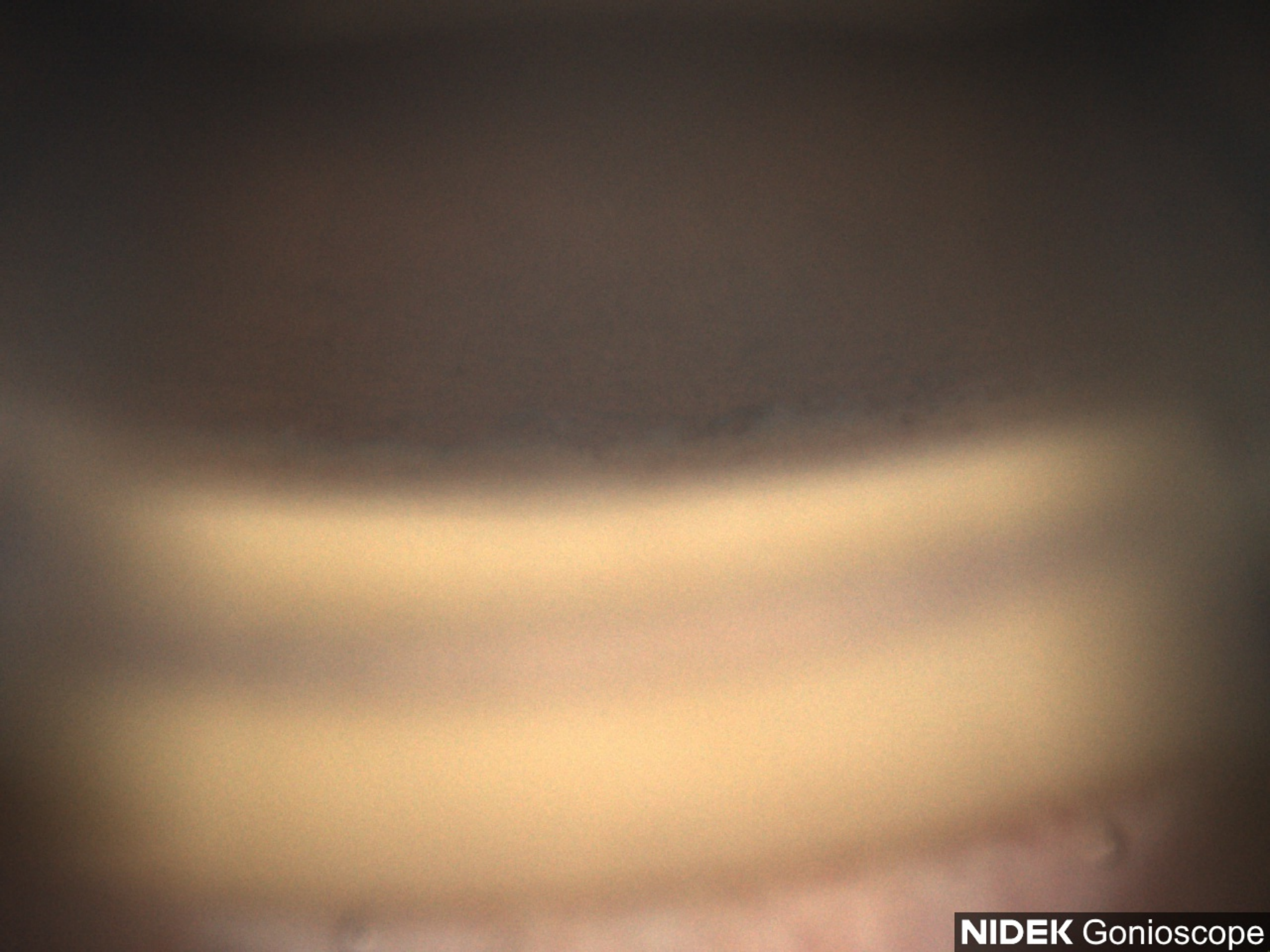

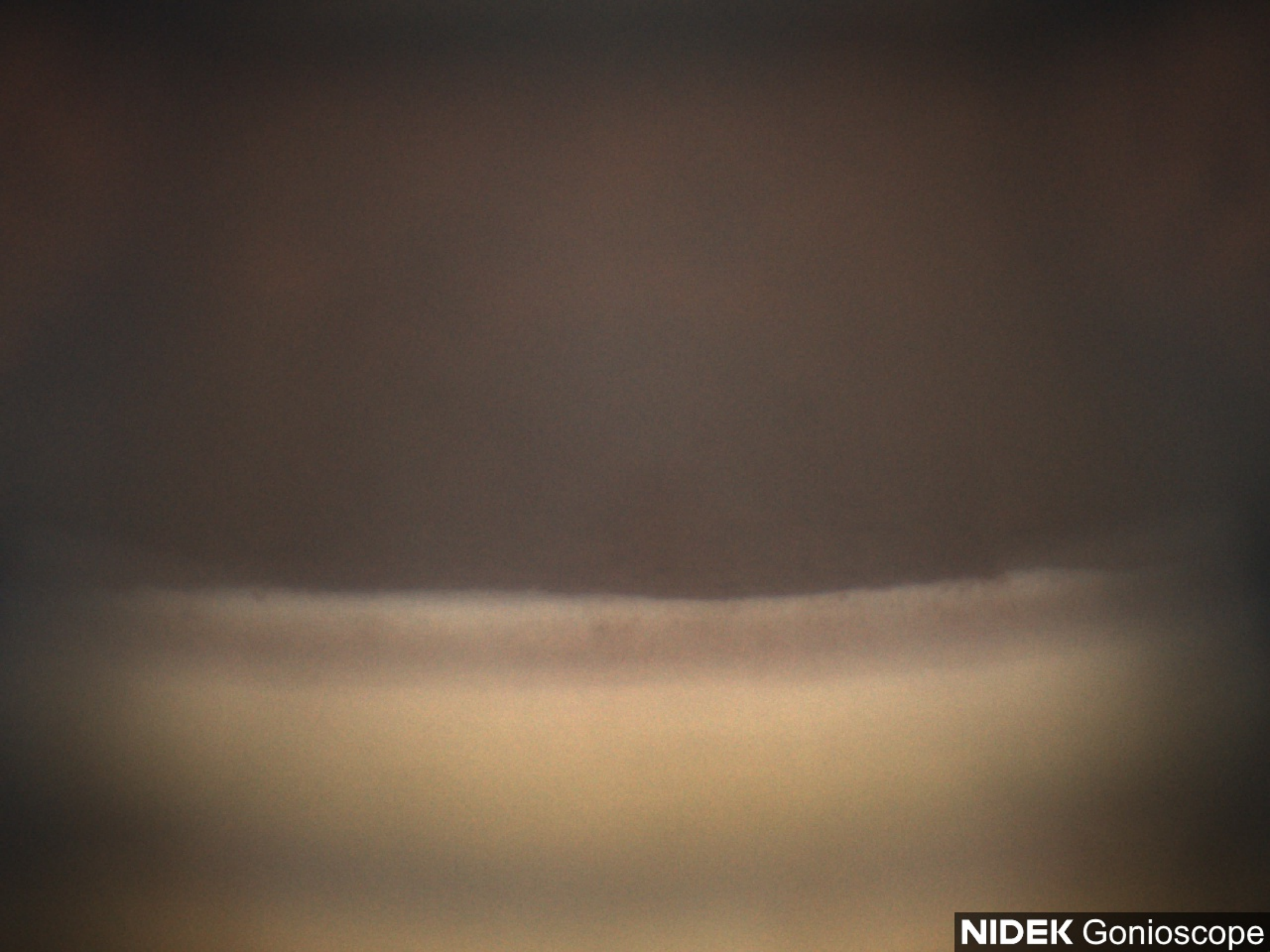

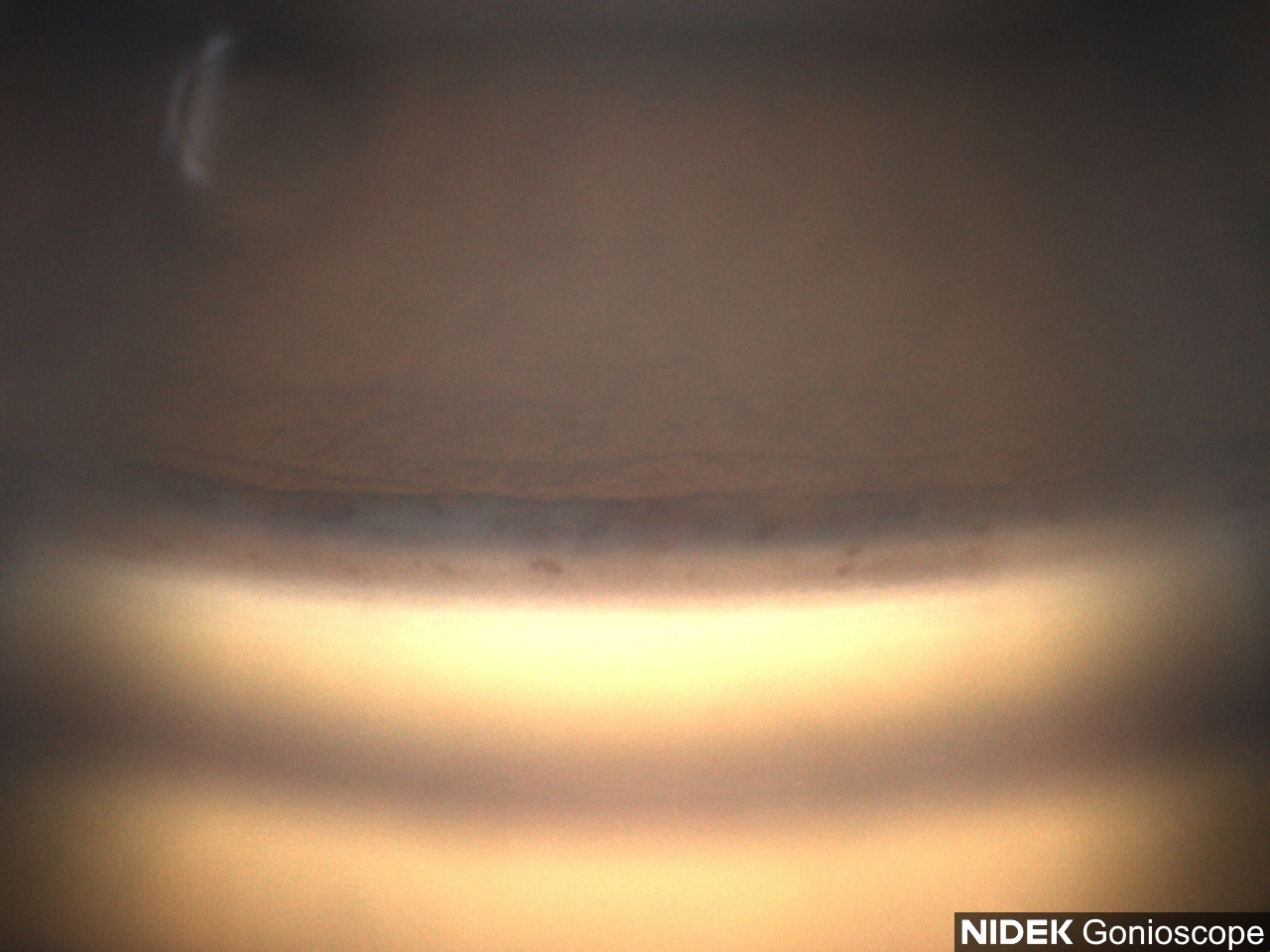

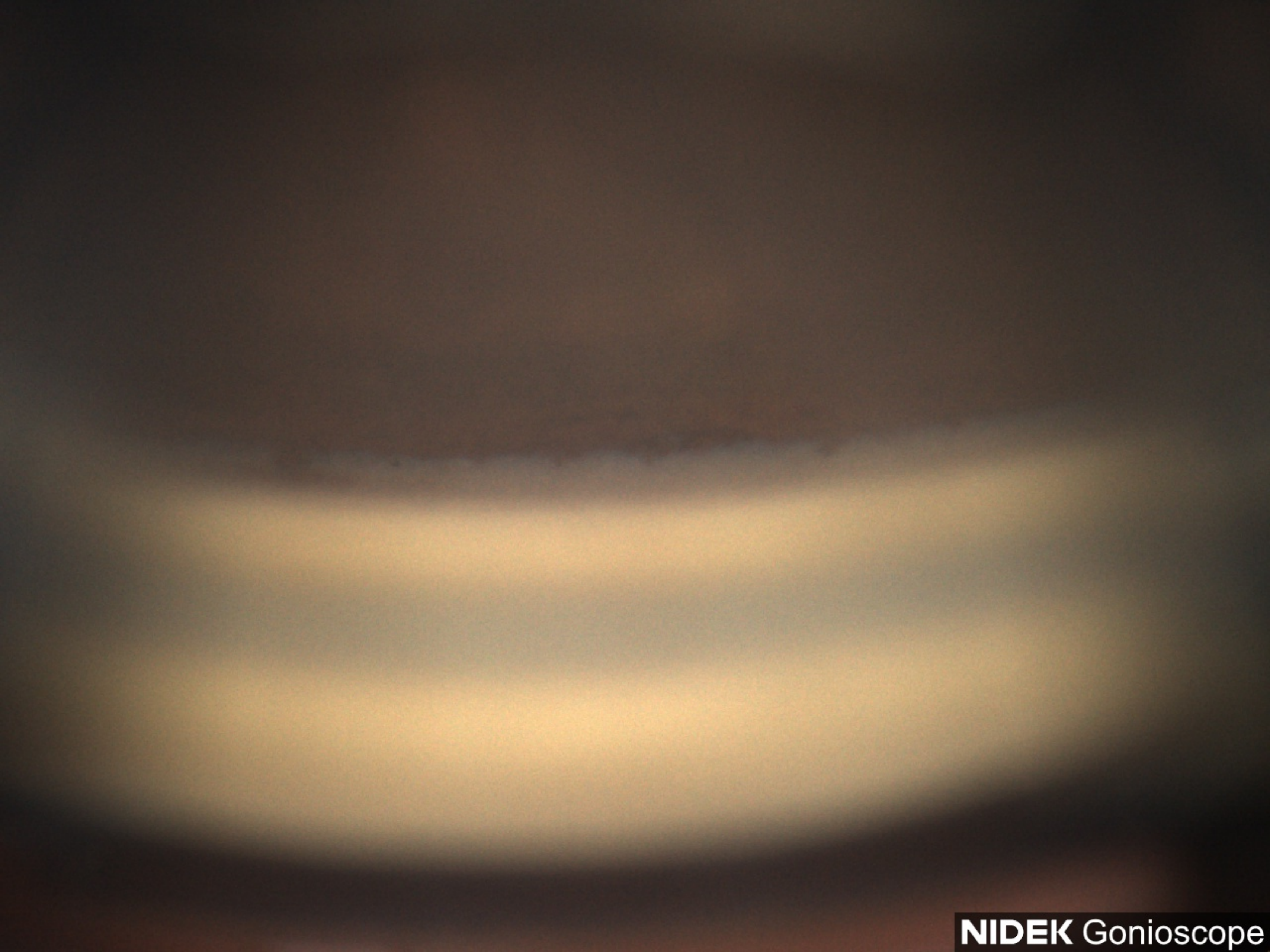

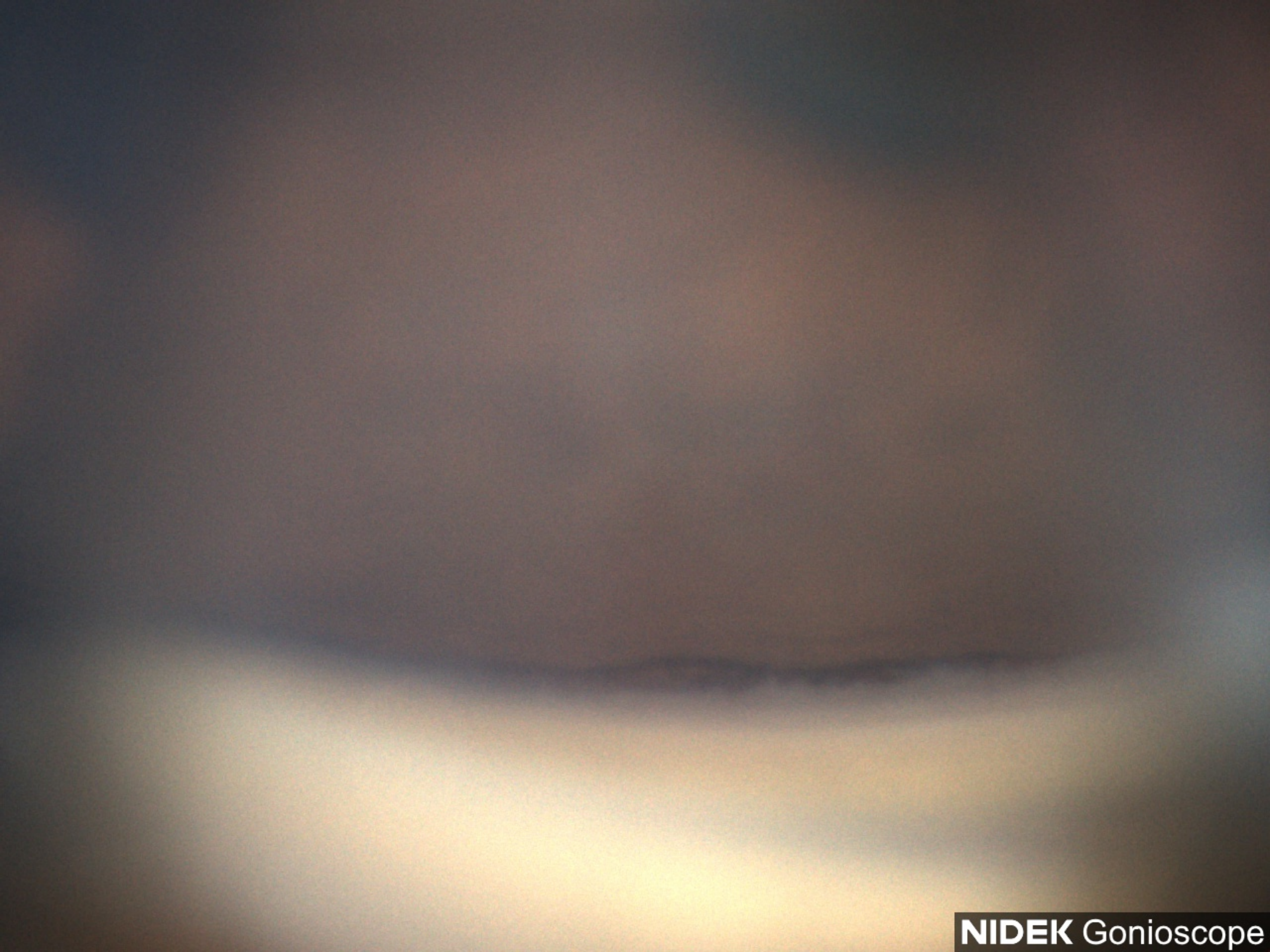

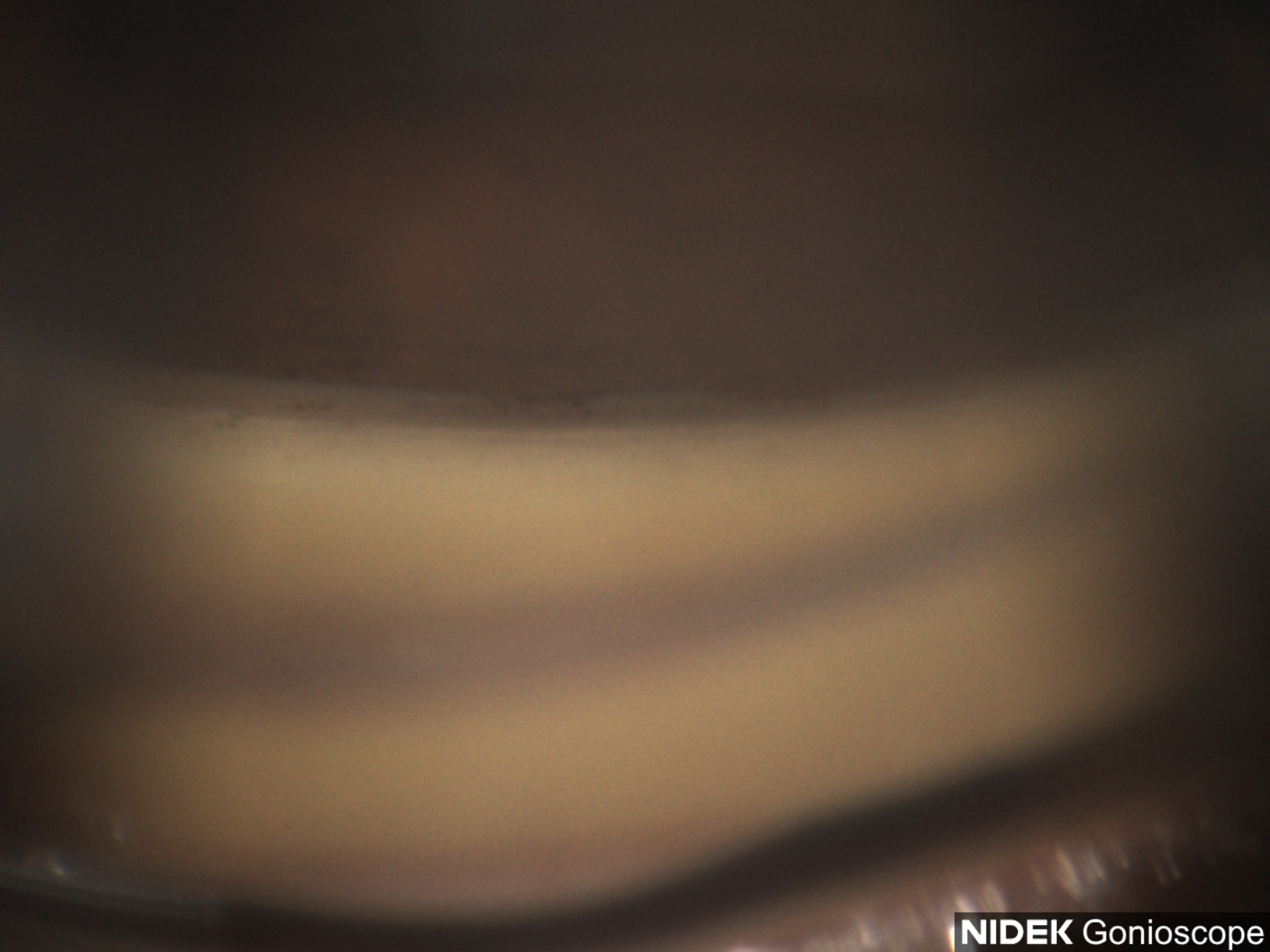

**NIDEK** Gonioscope

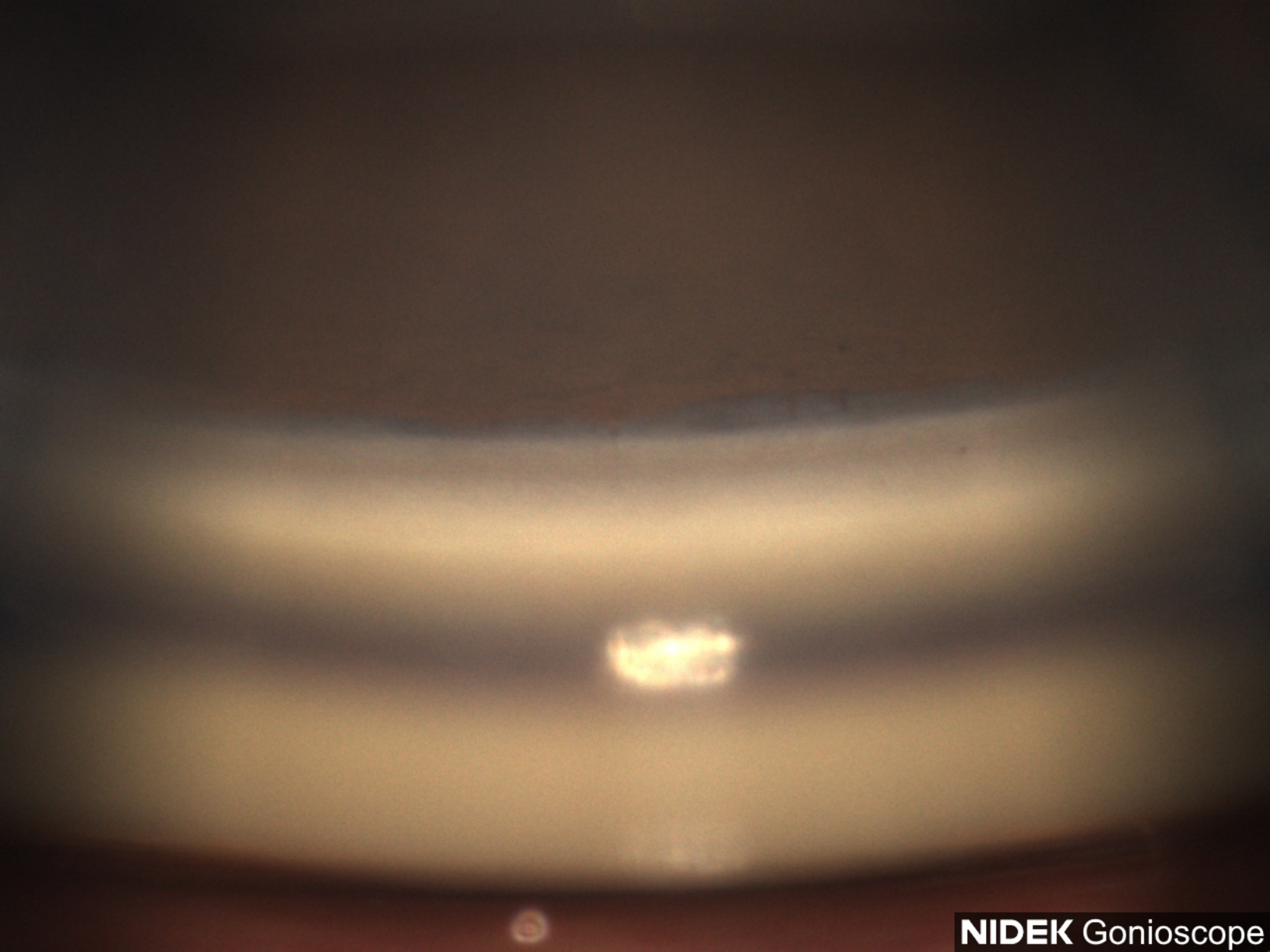

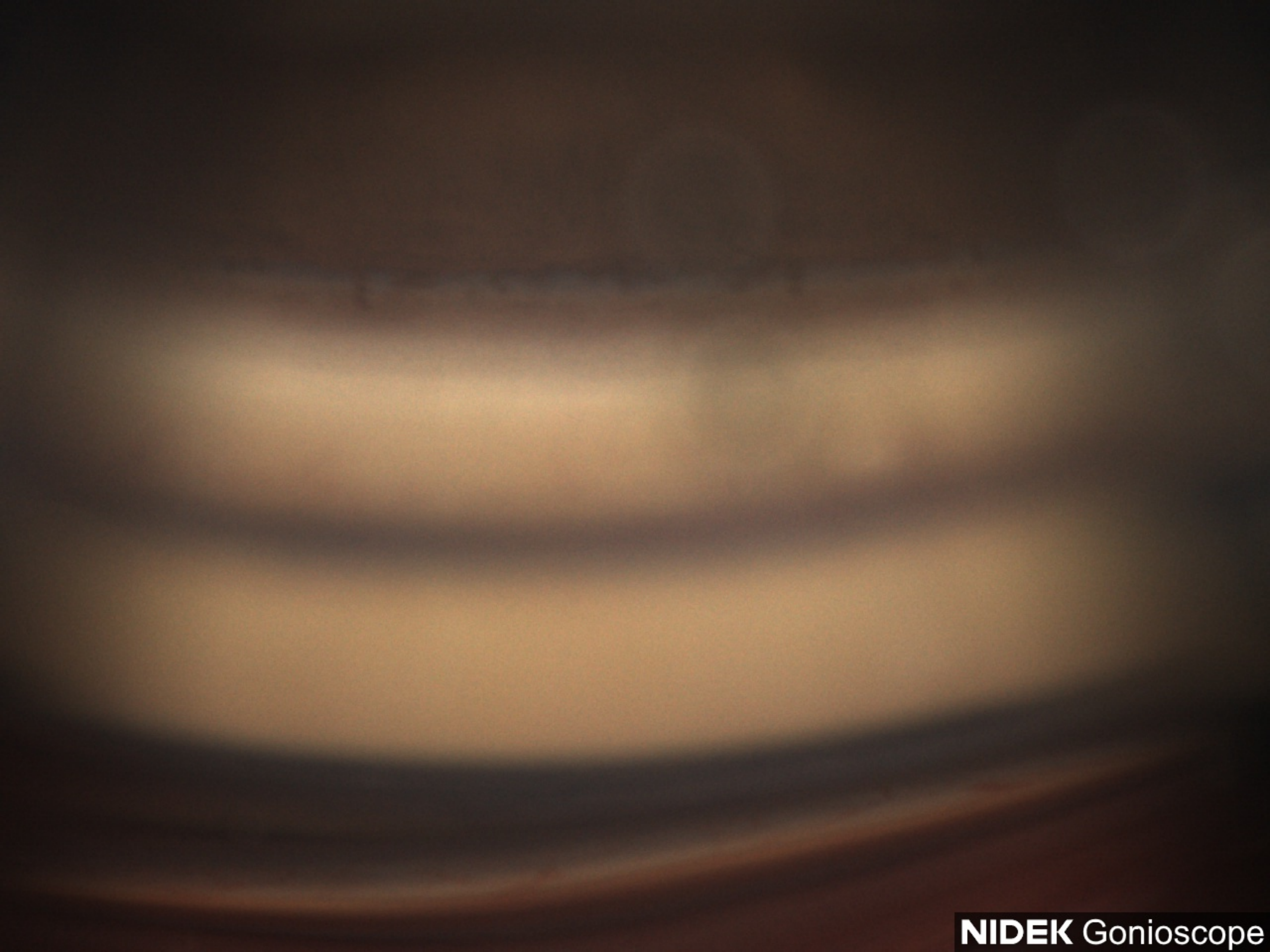

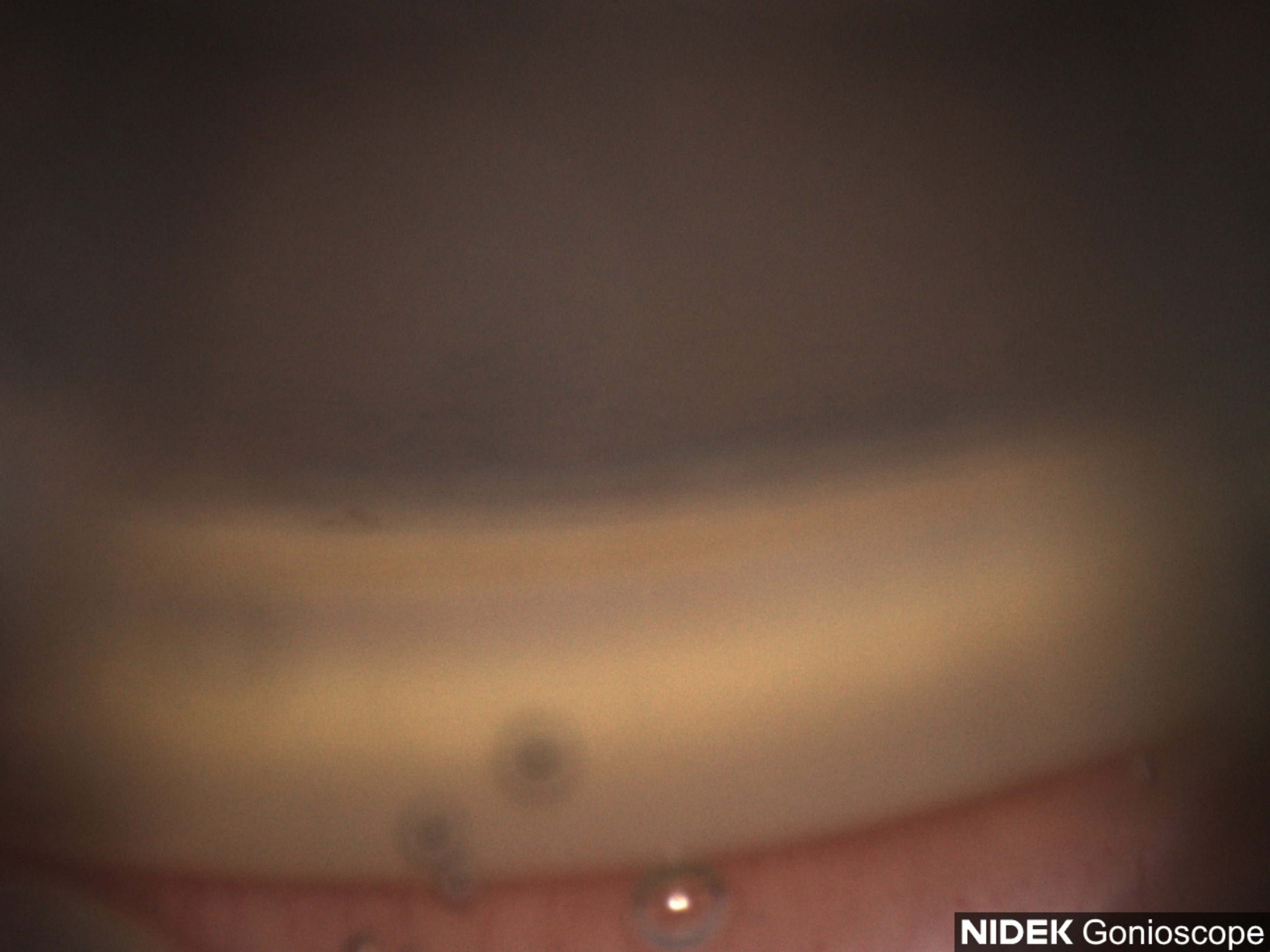

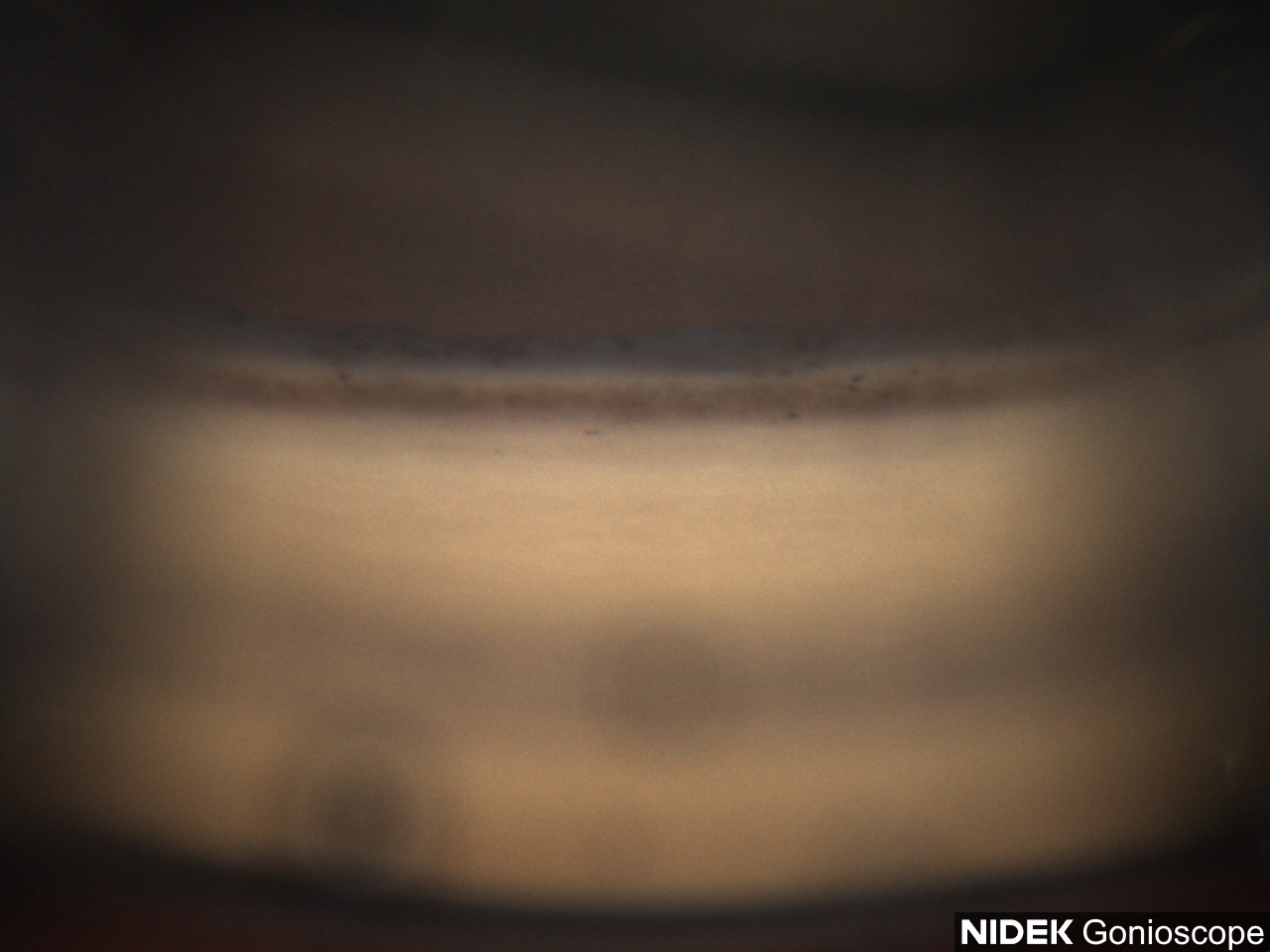

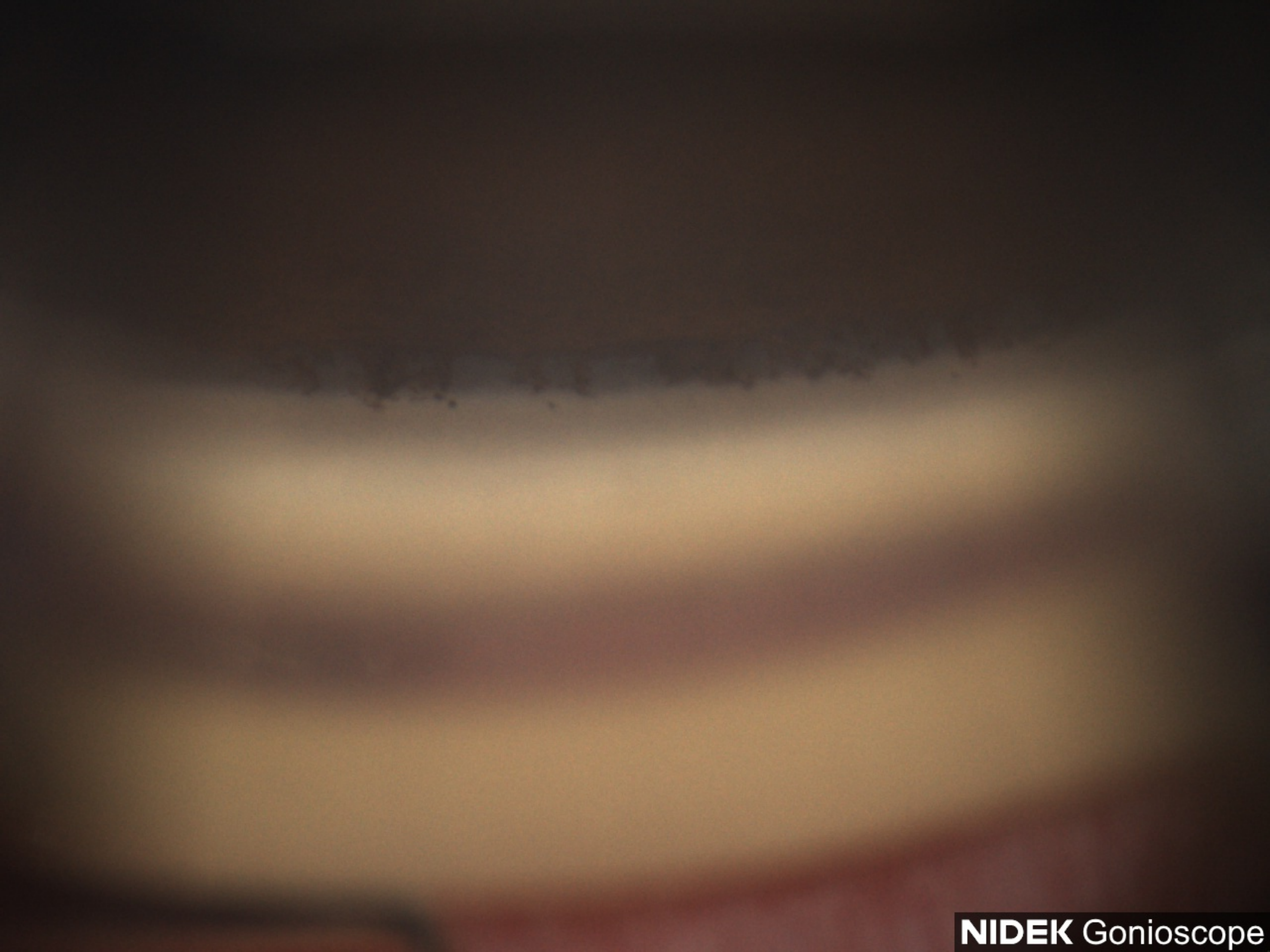

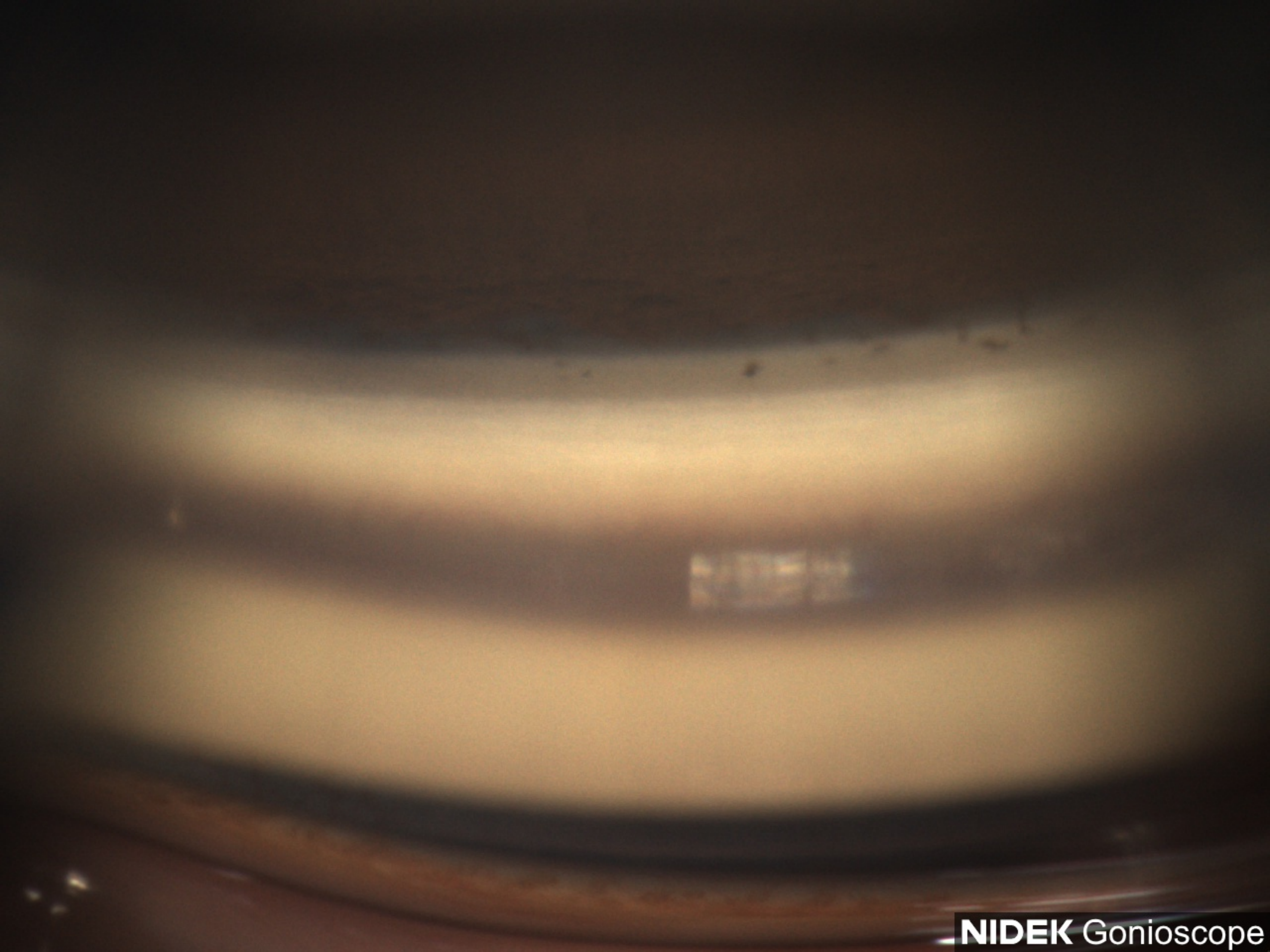

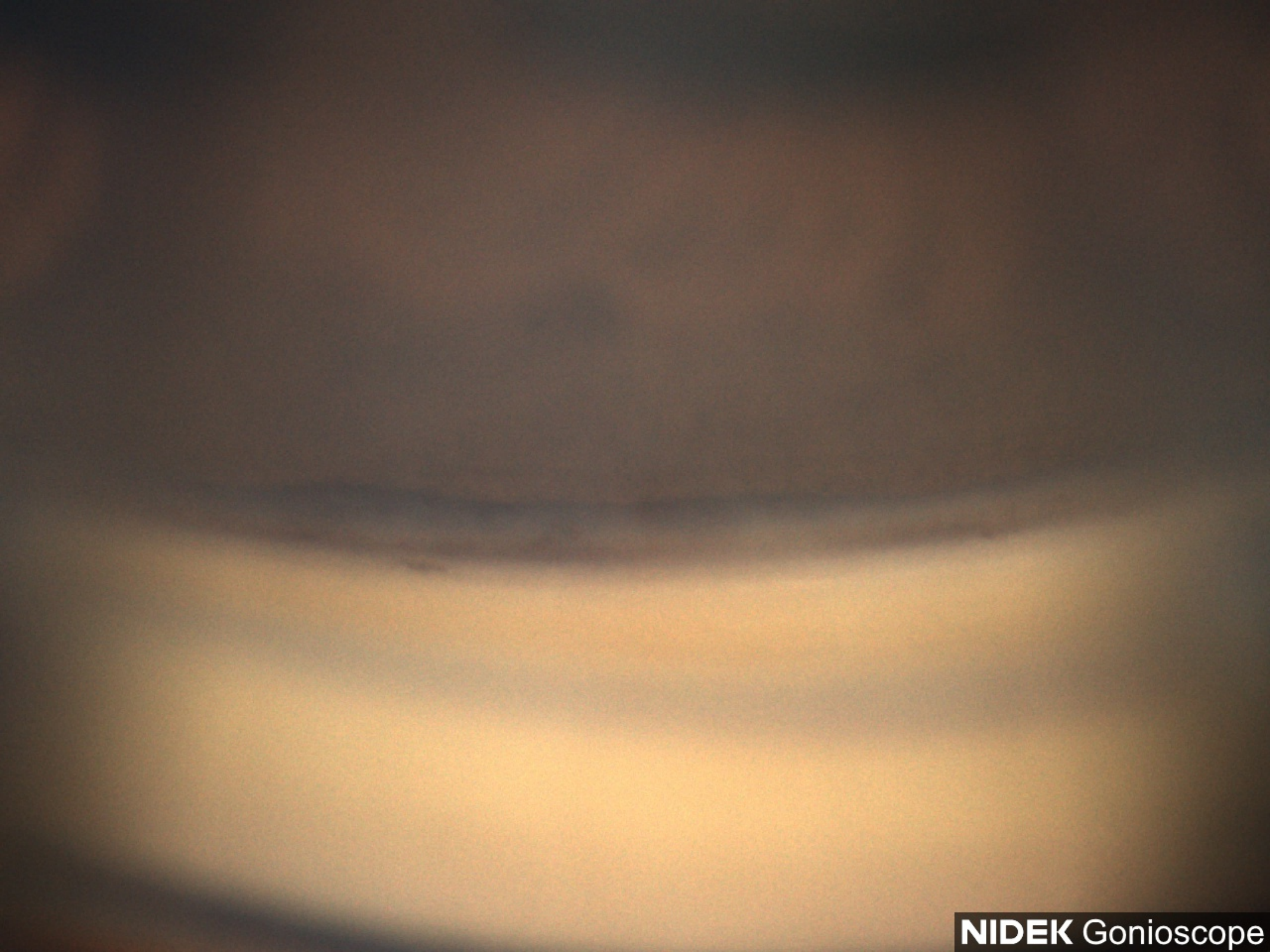

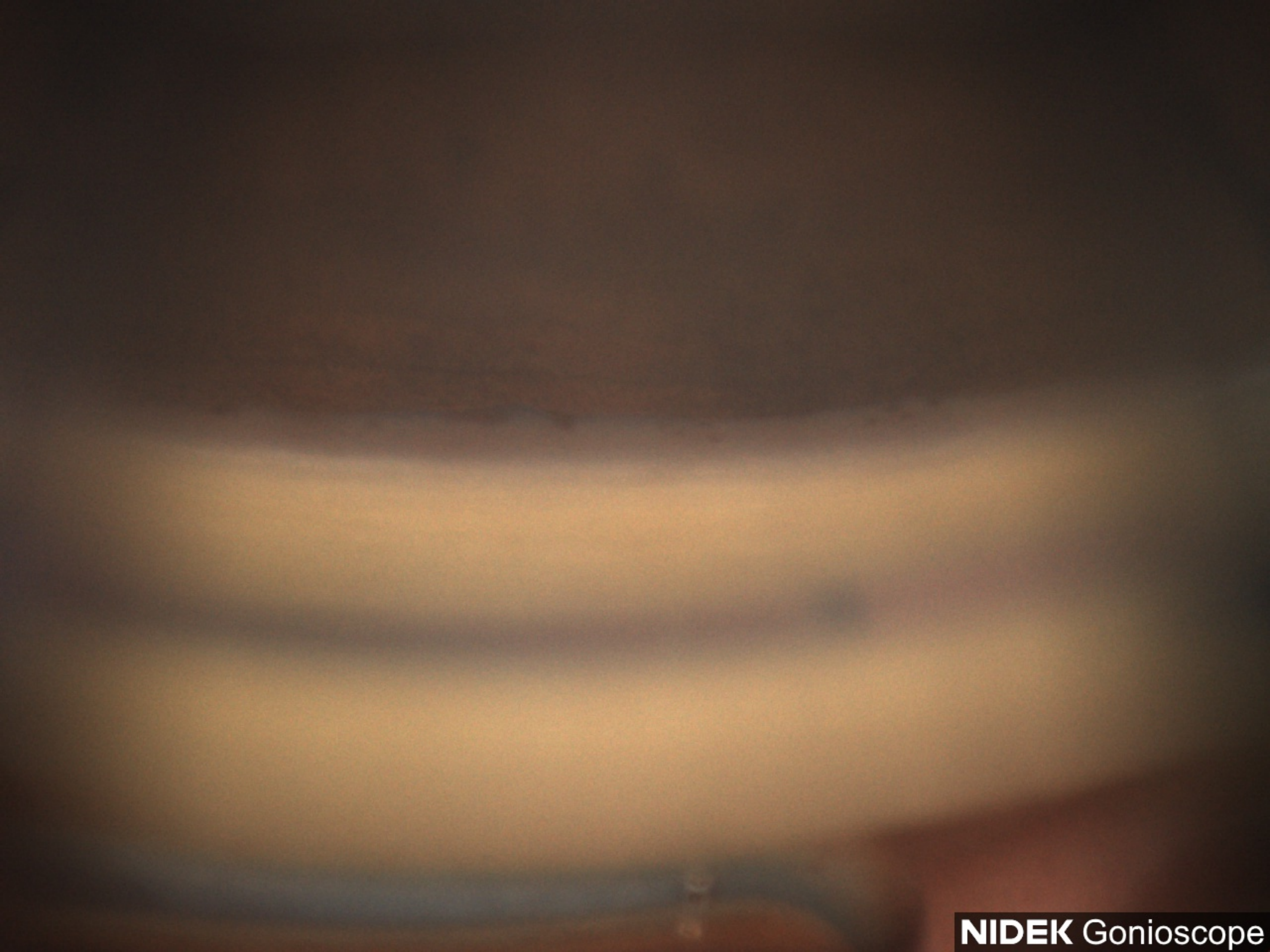

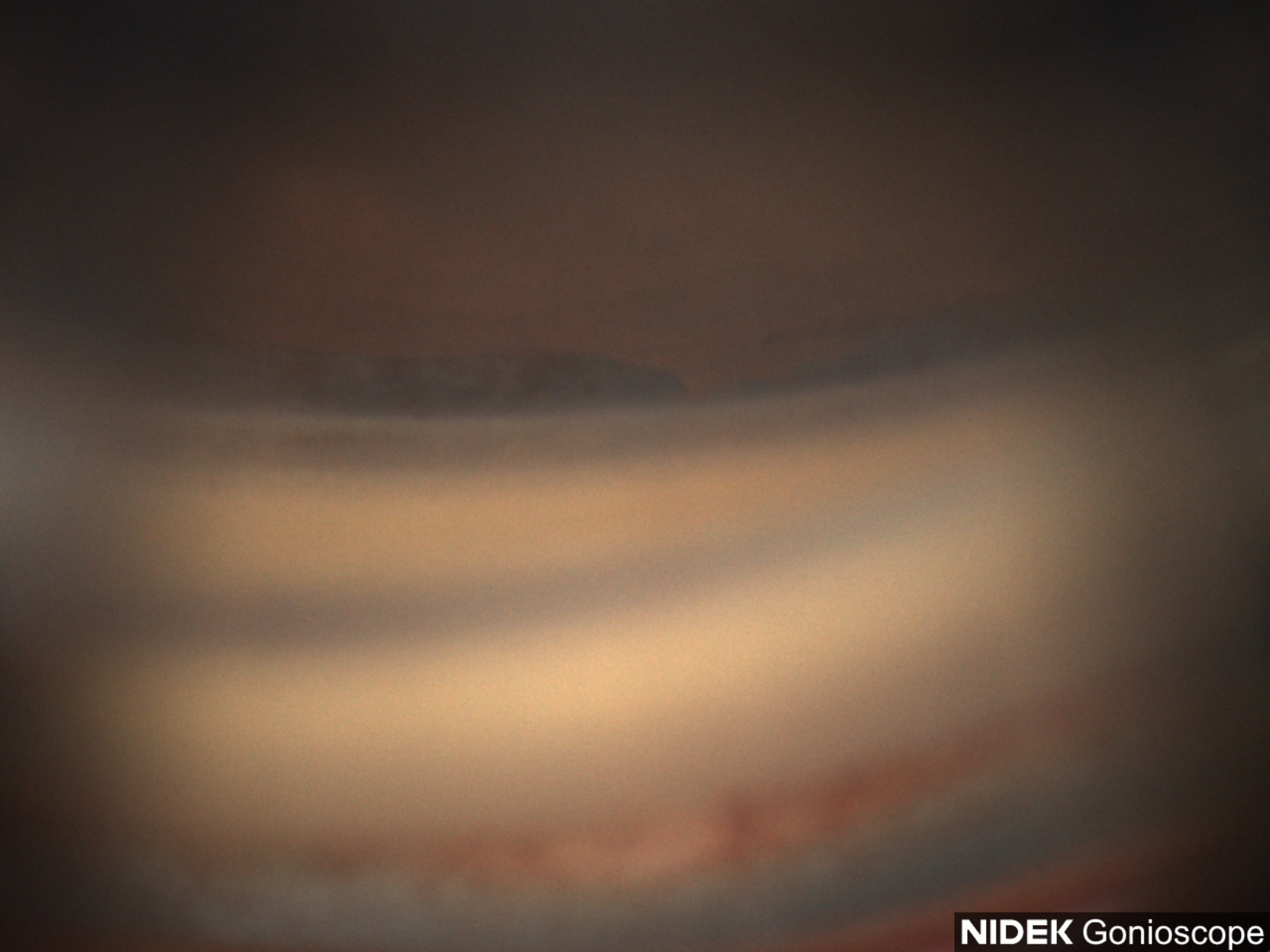

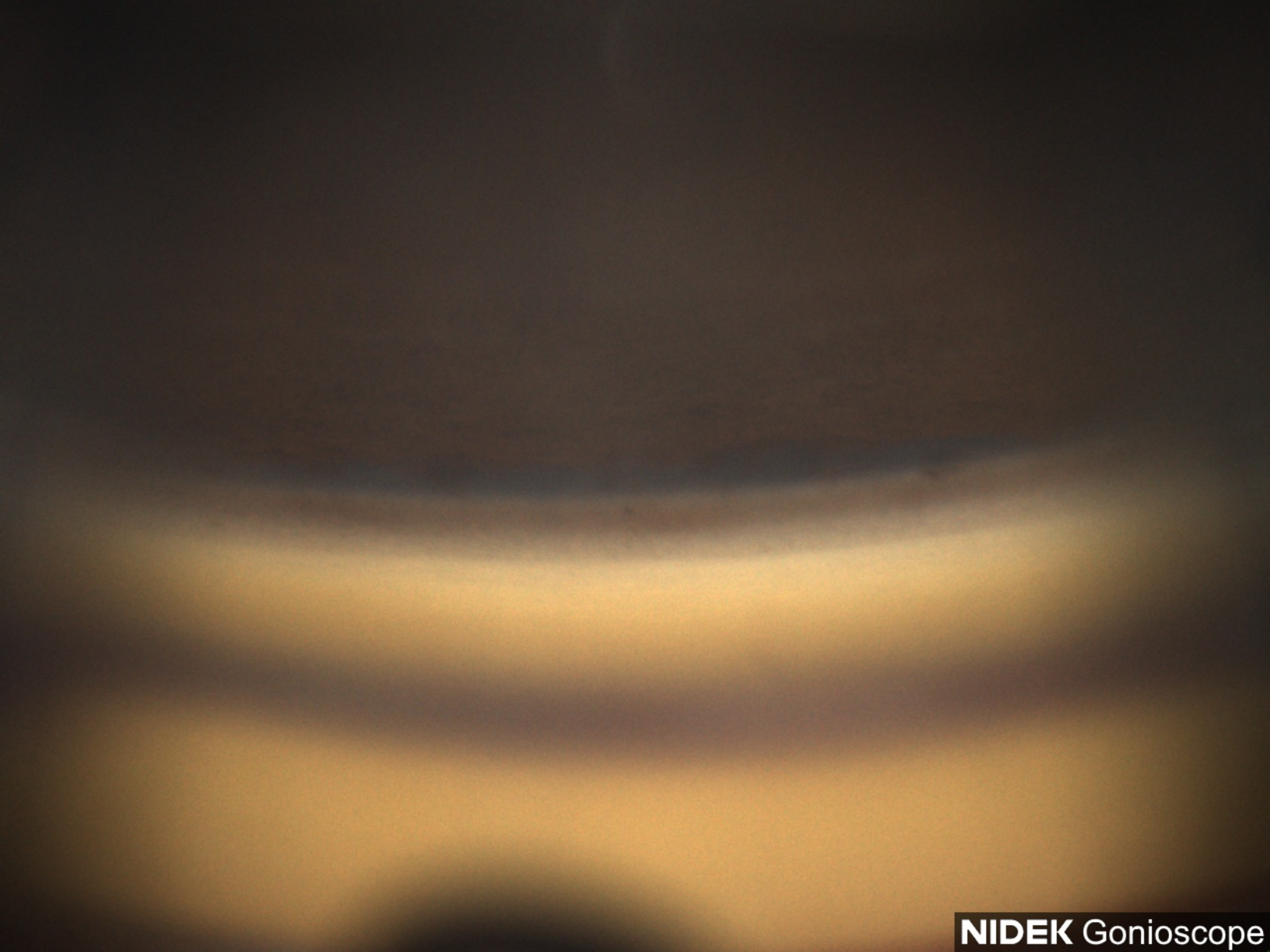

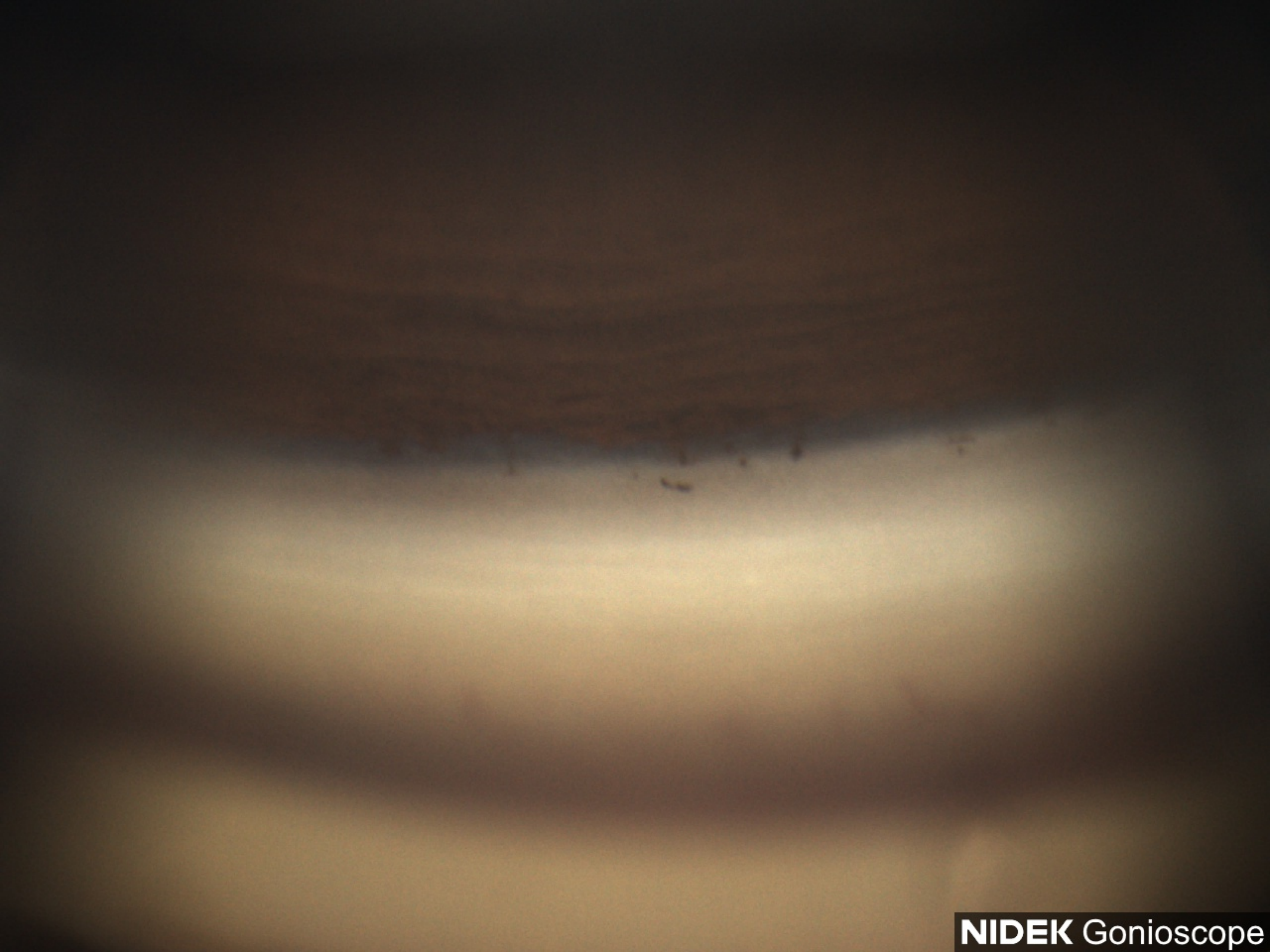

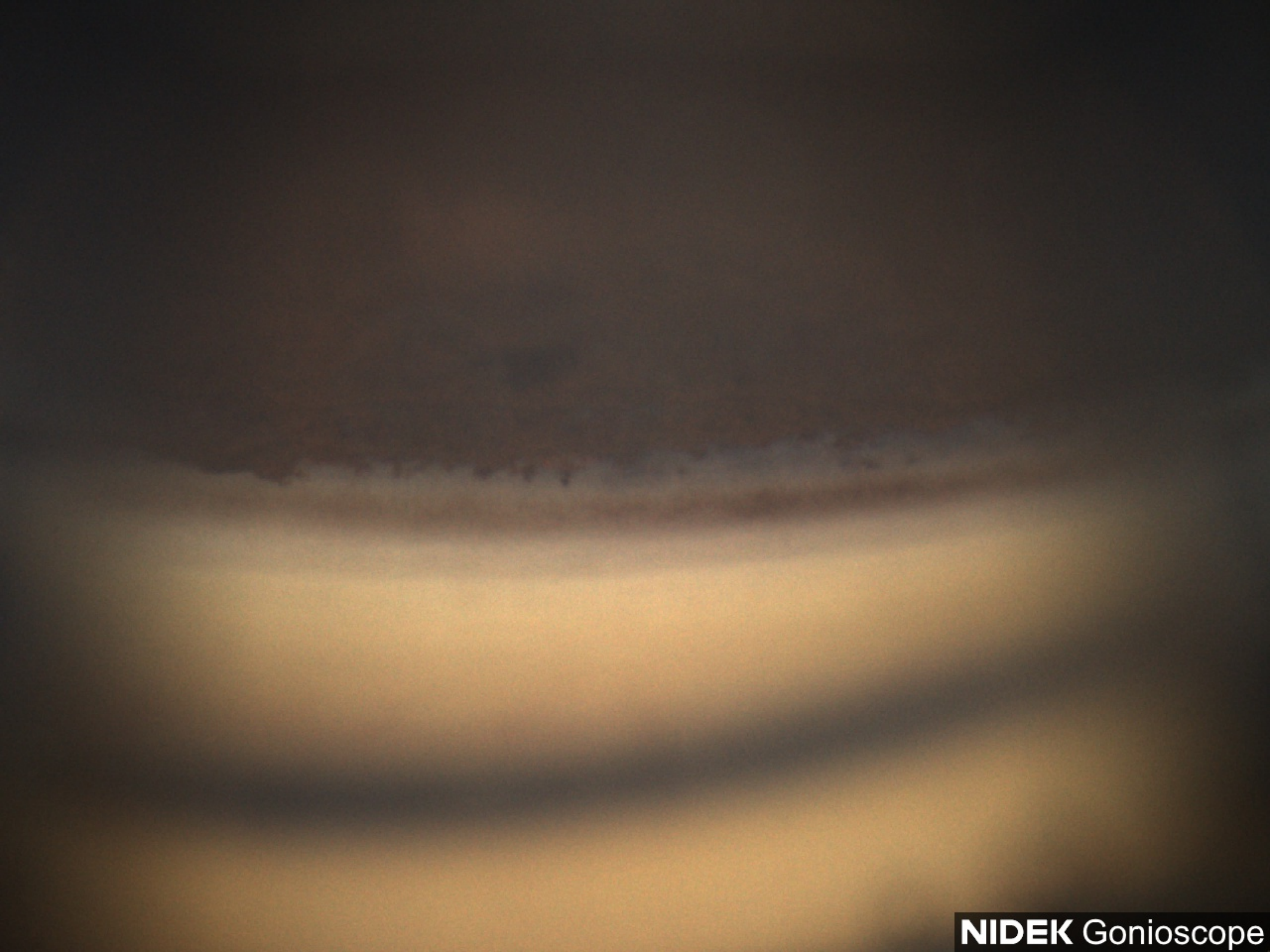

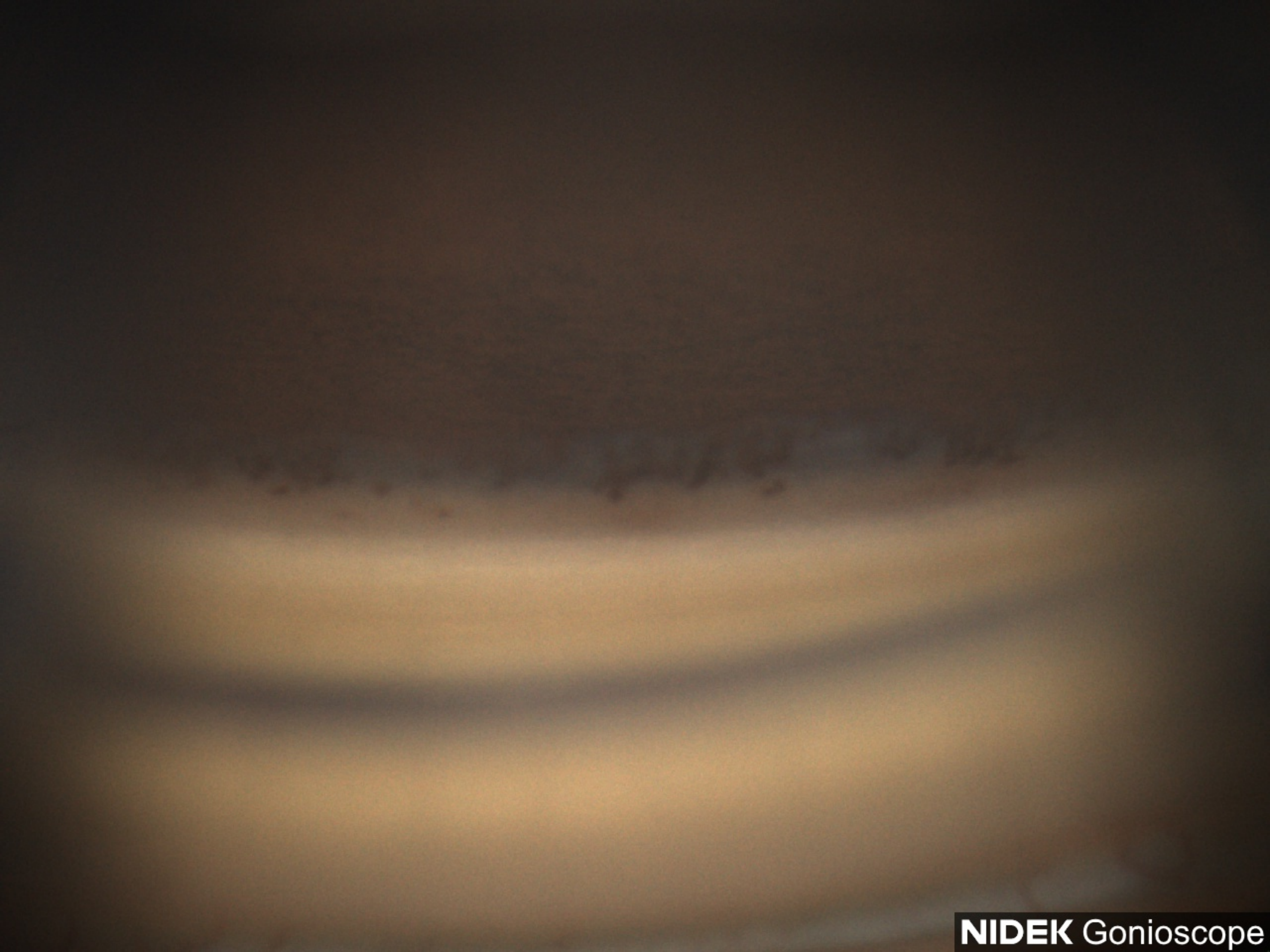

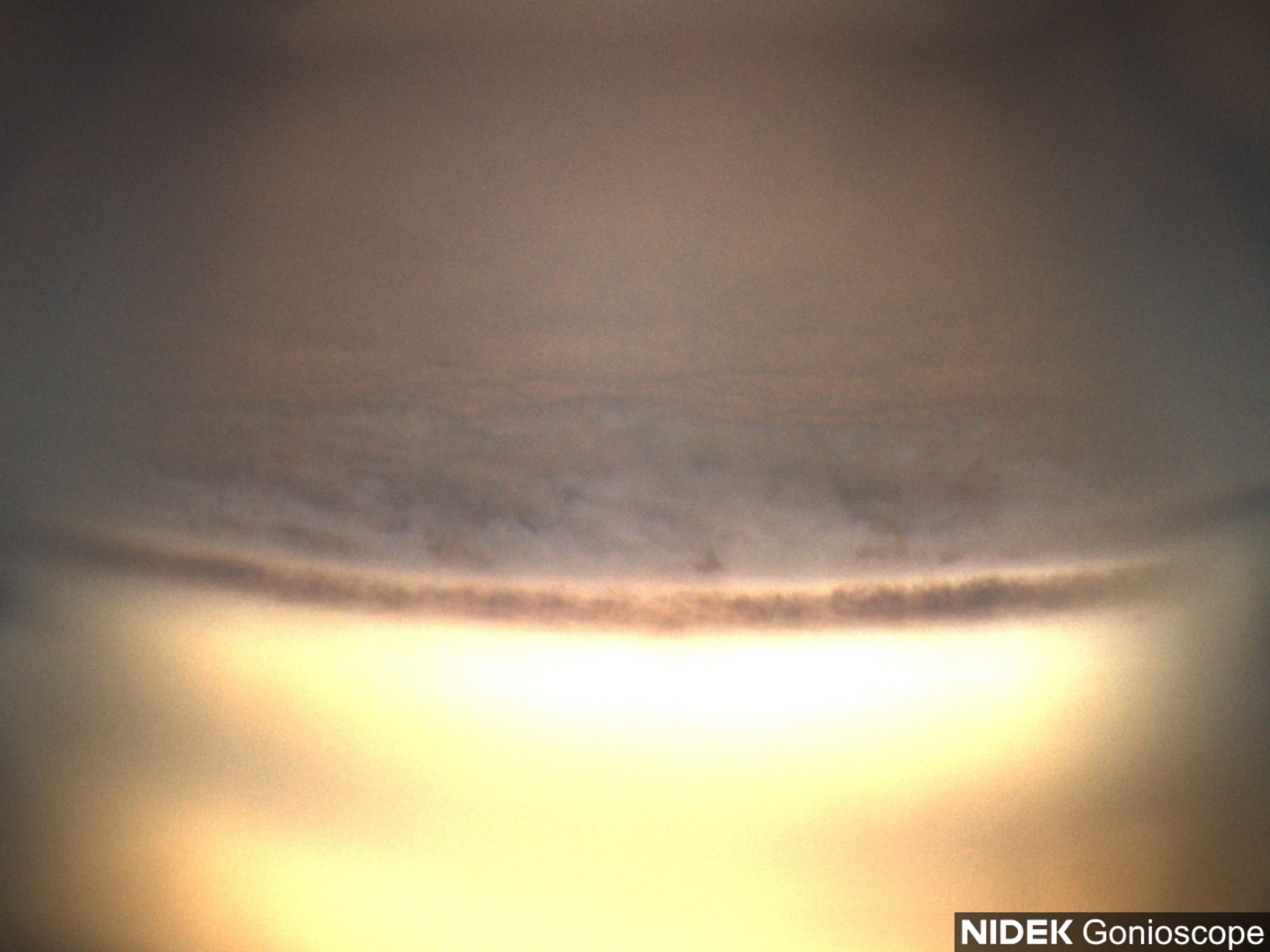

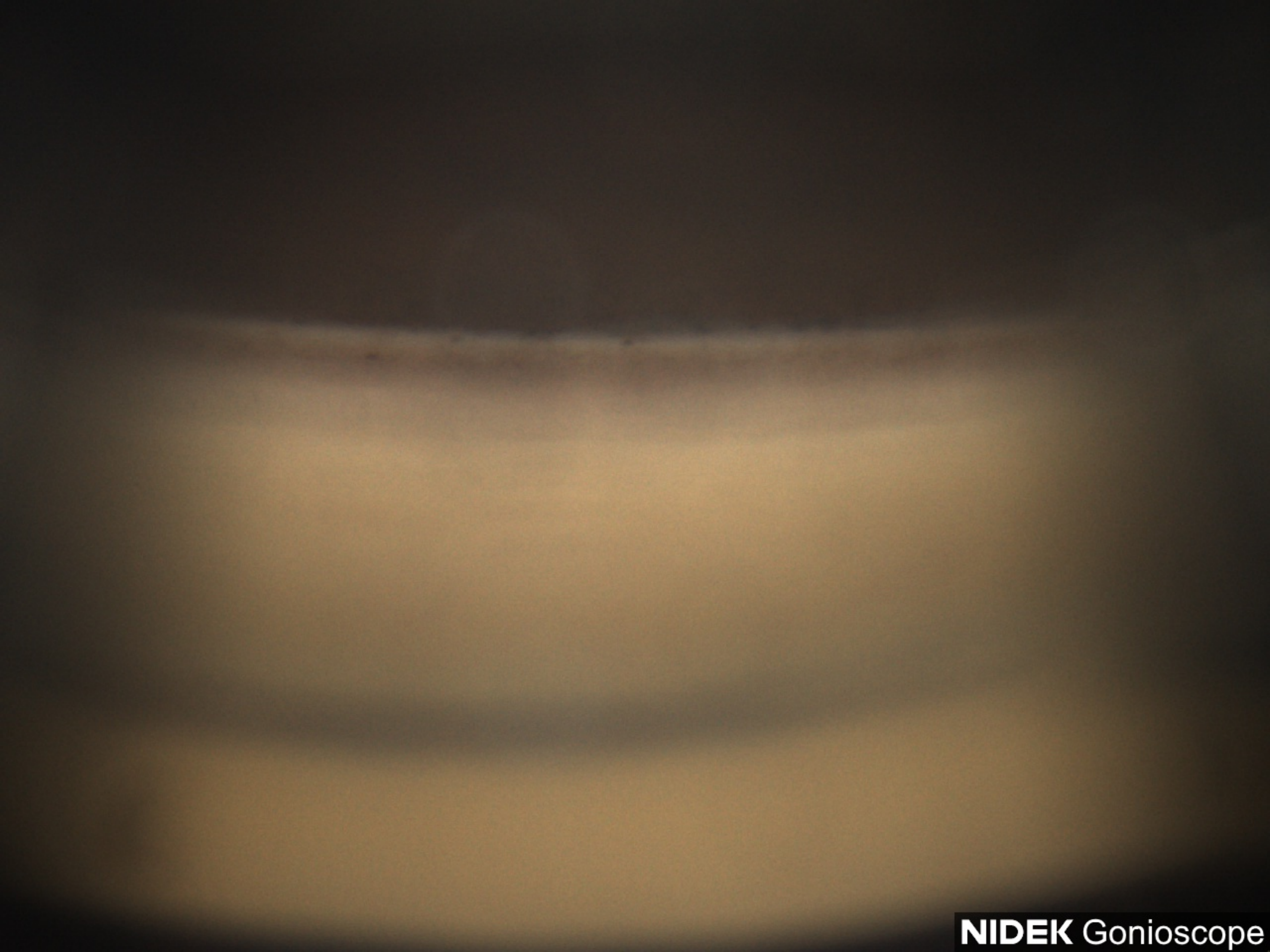

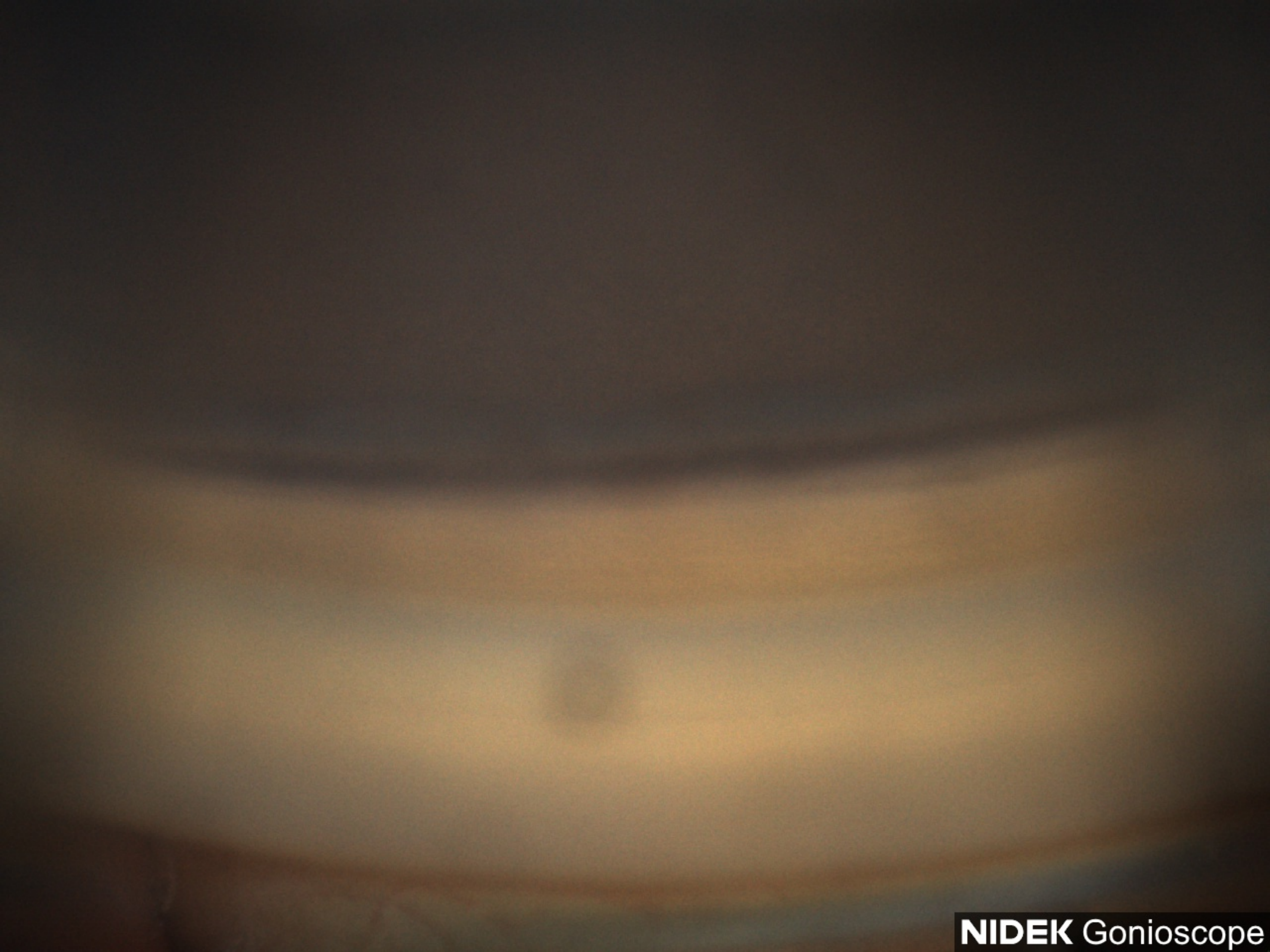

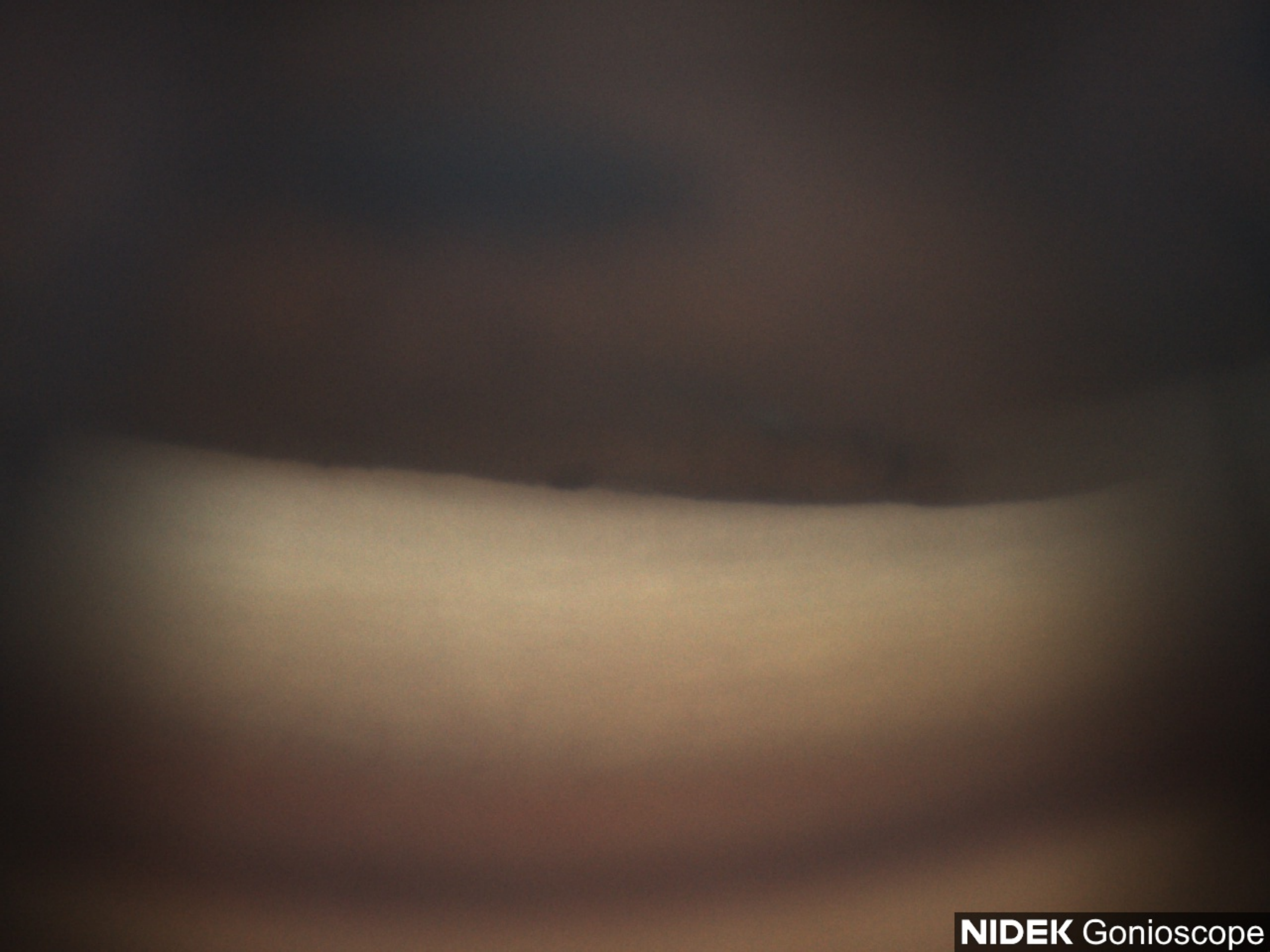

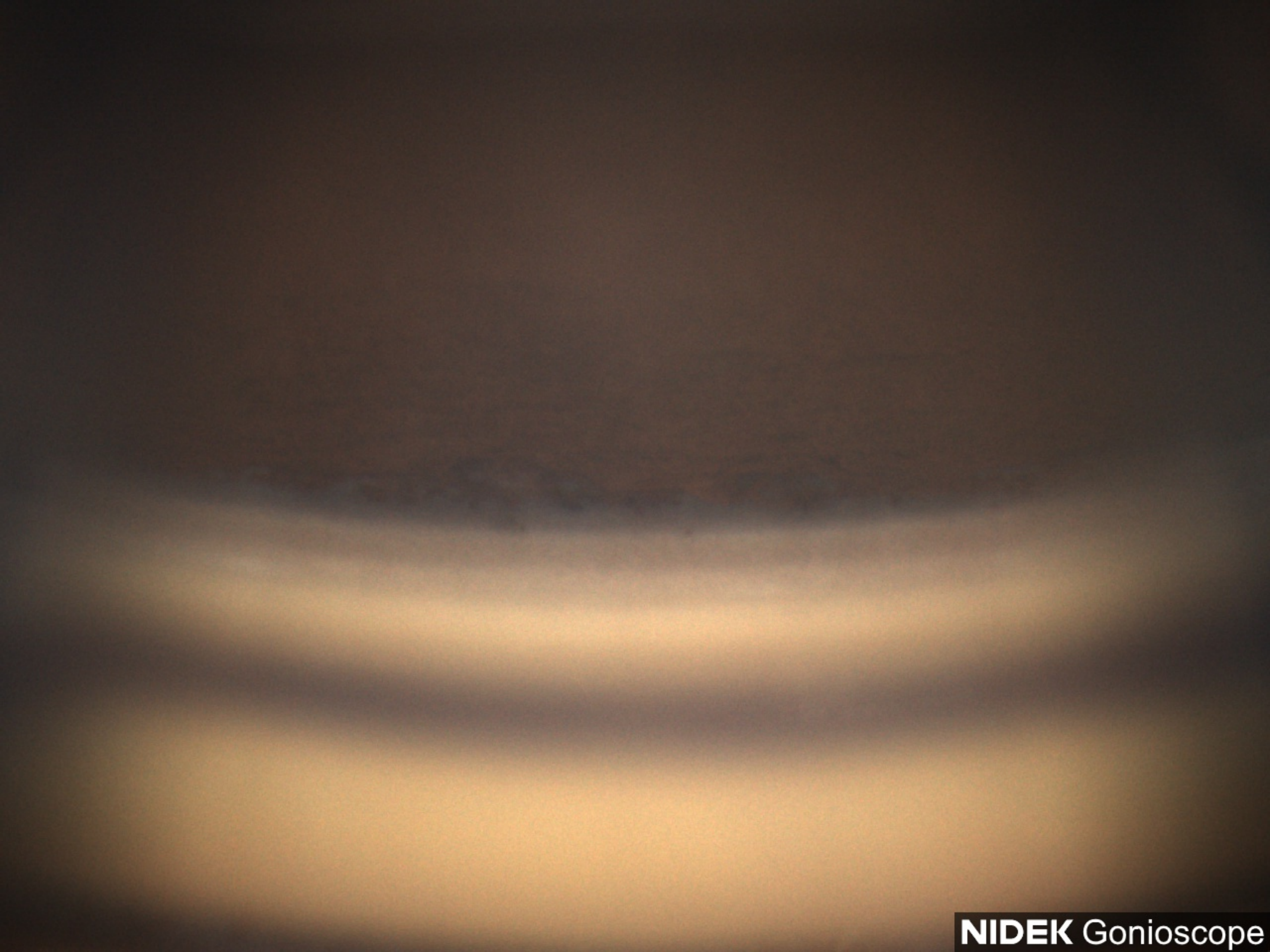

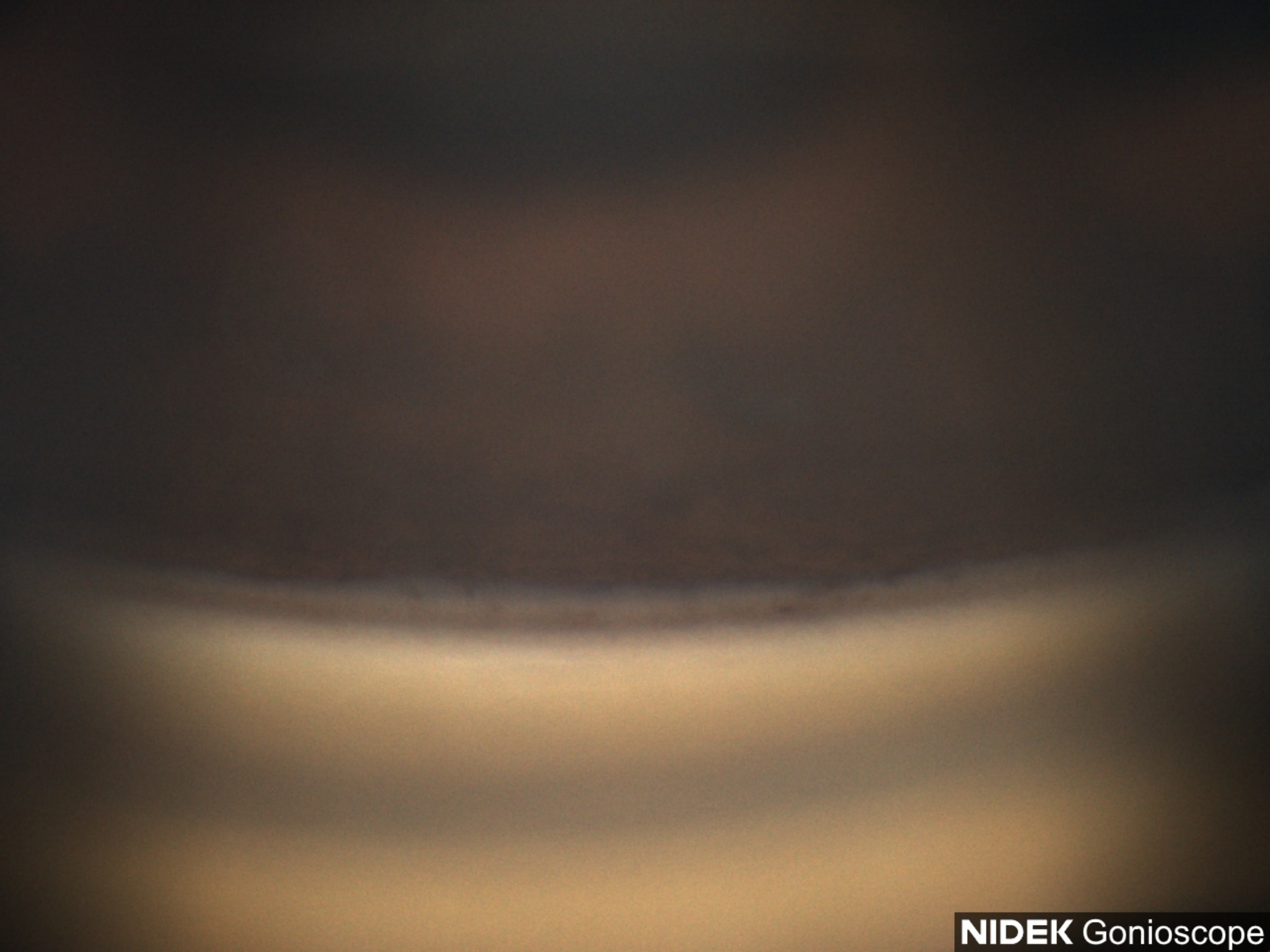

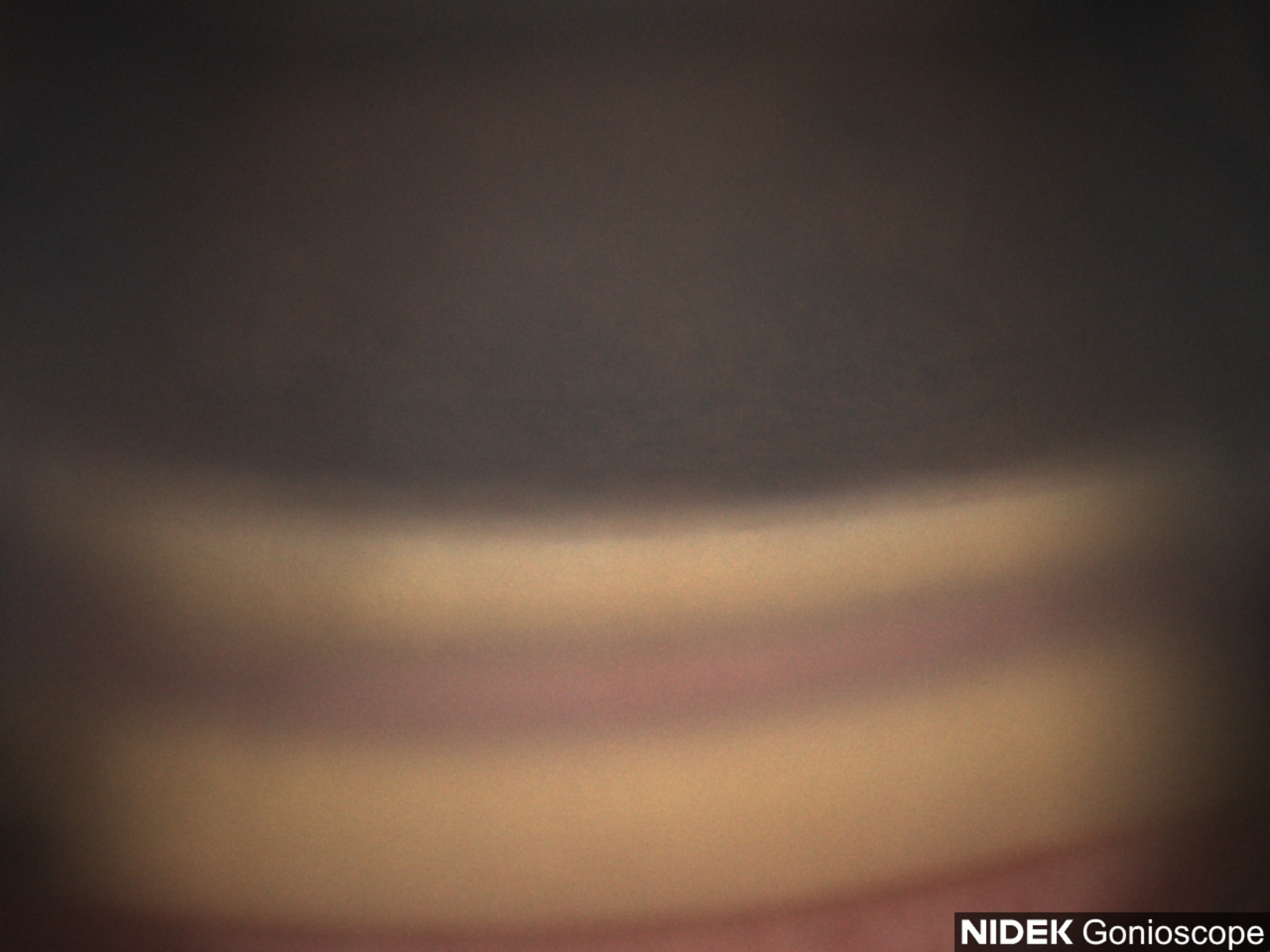

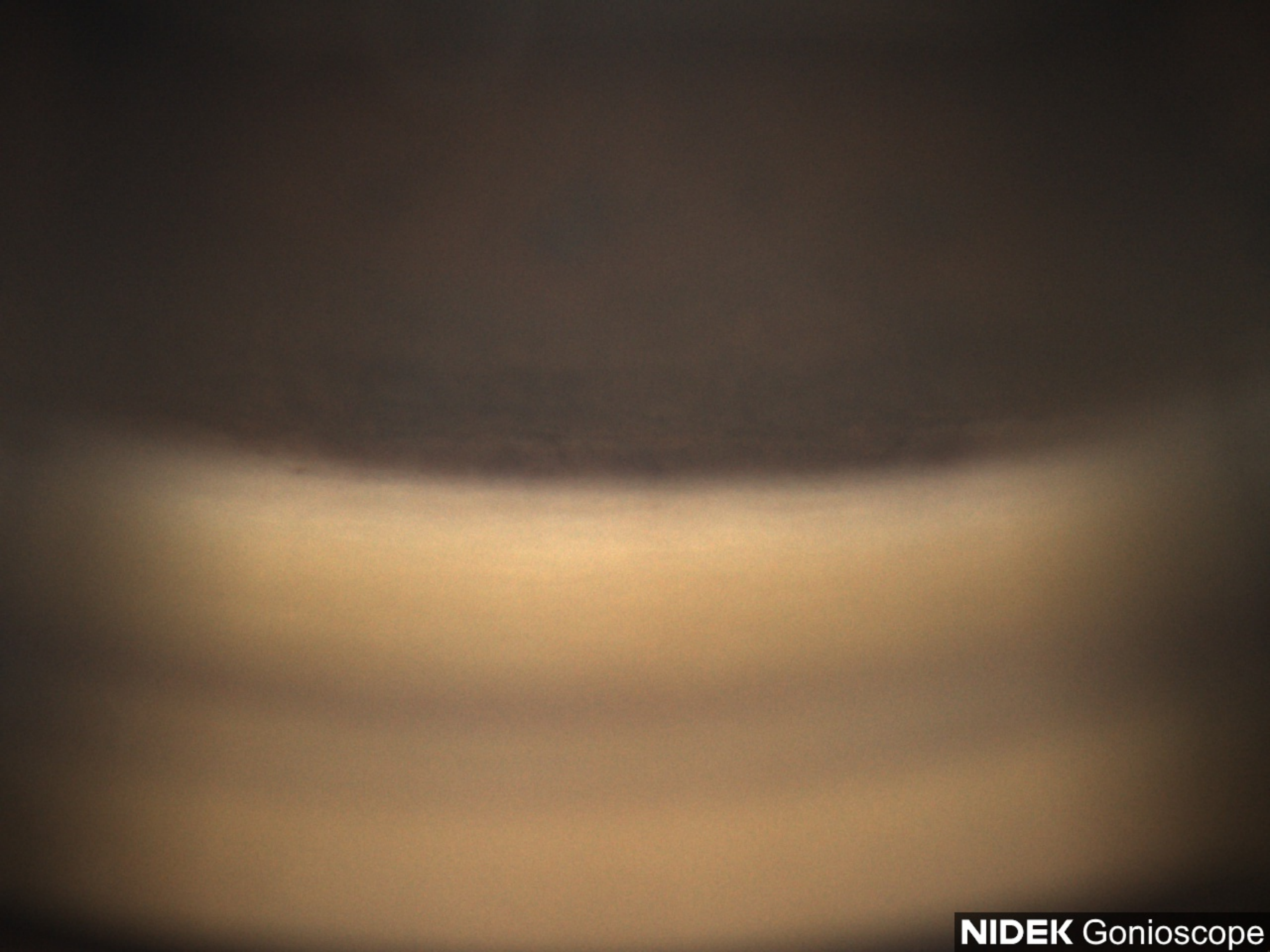

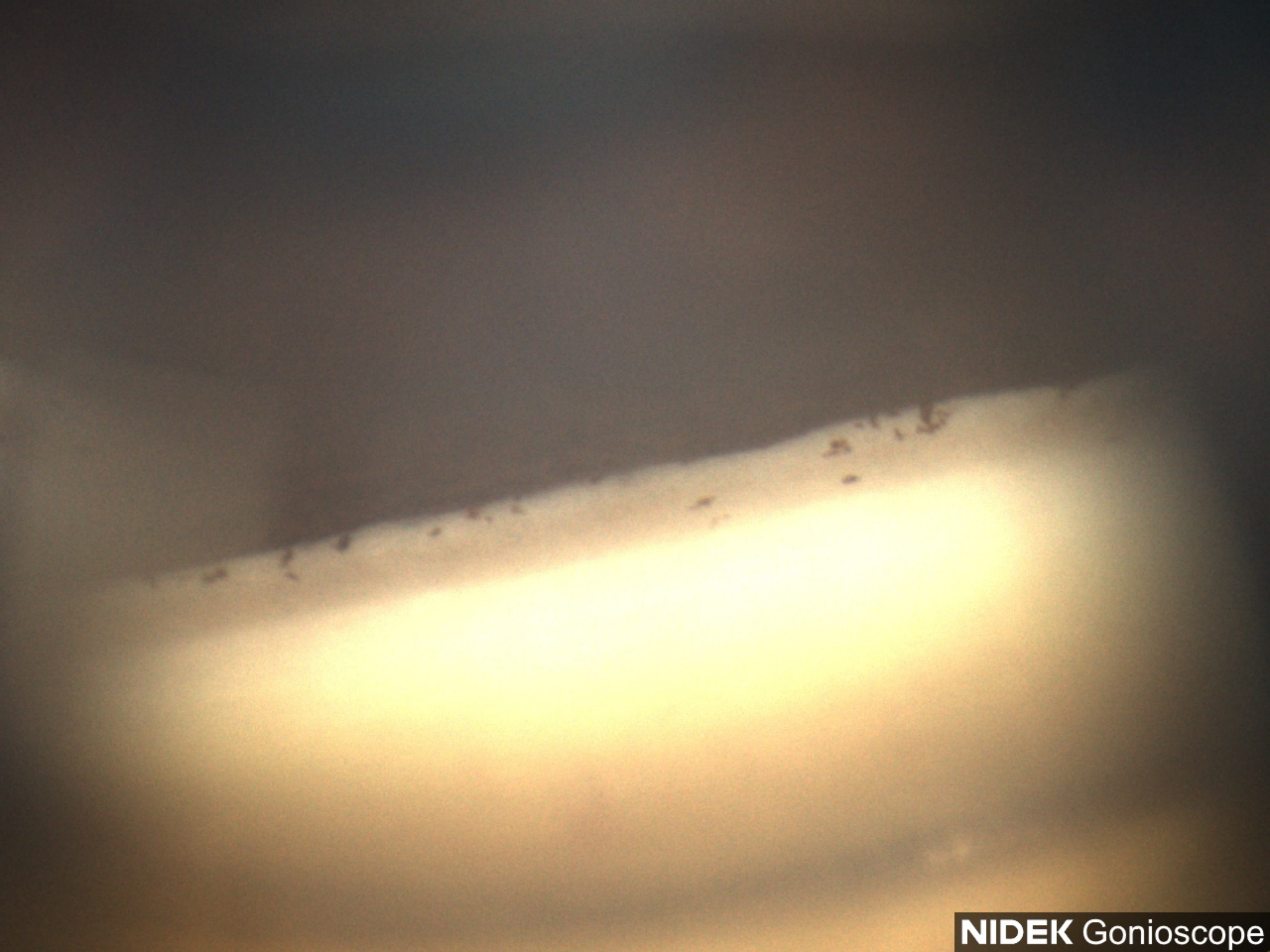

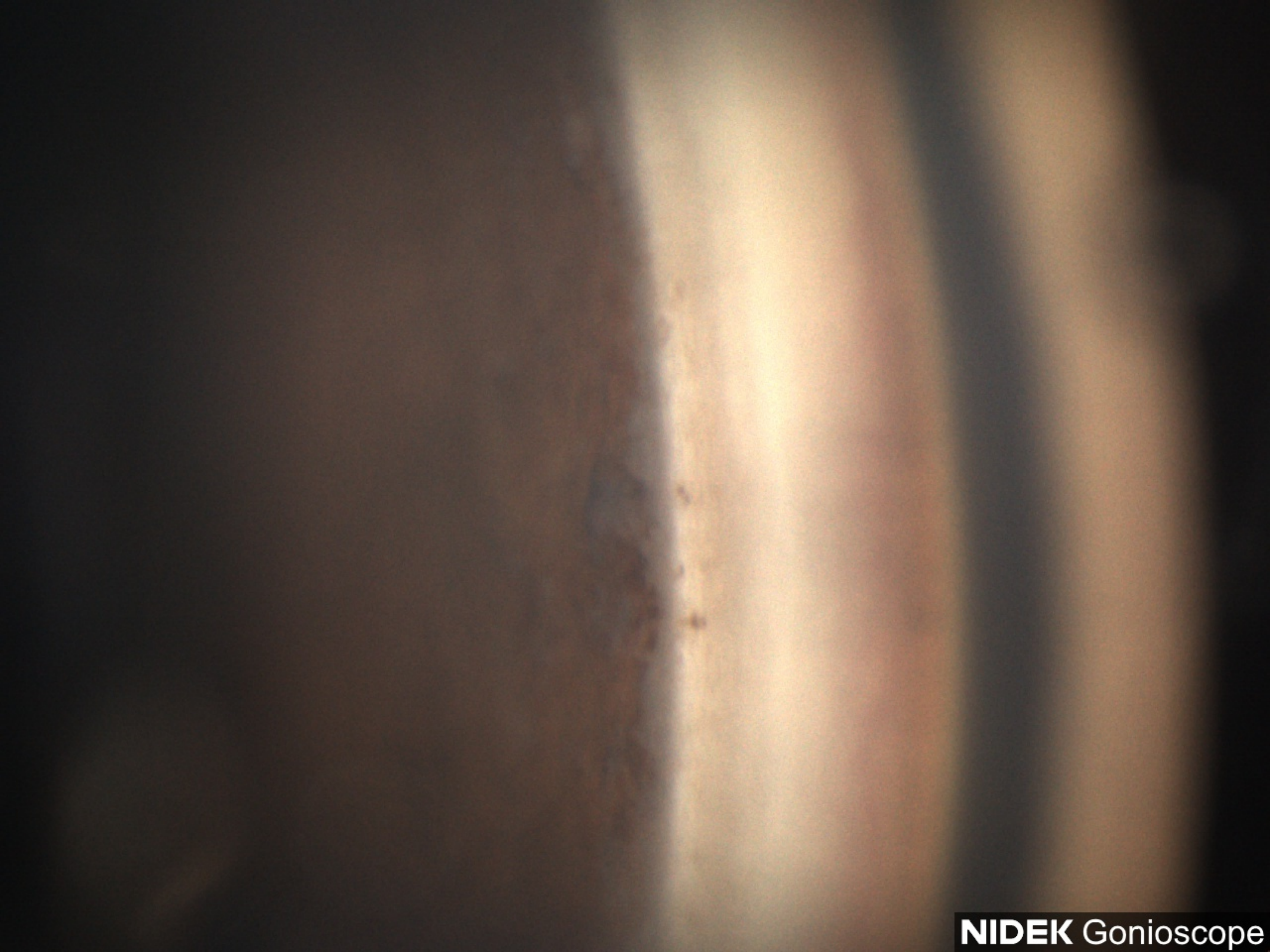

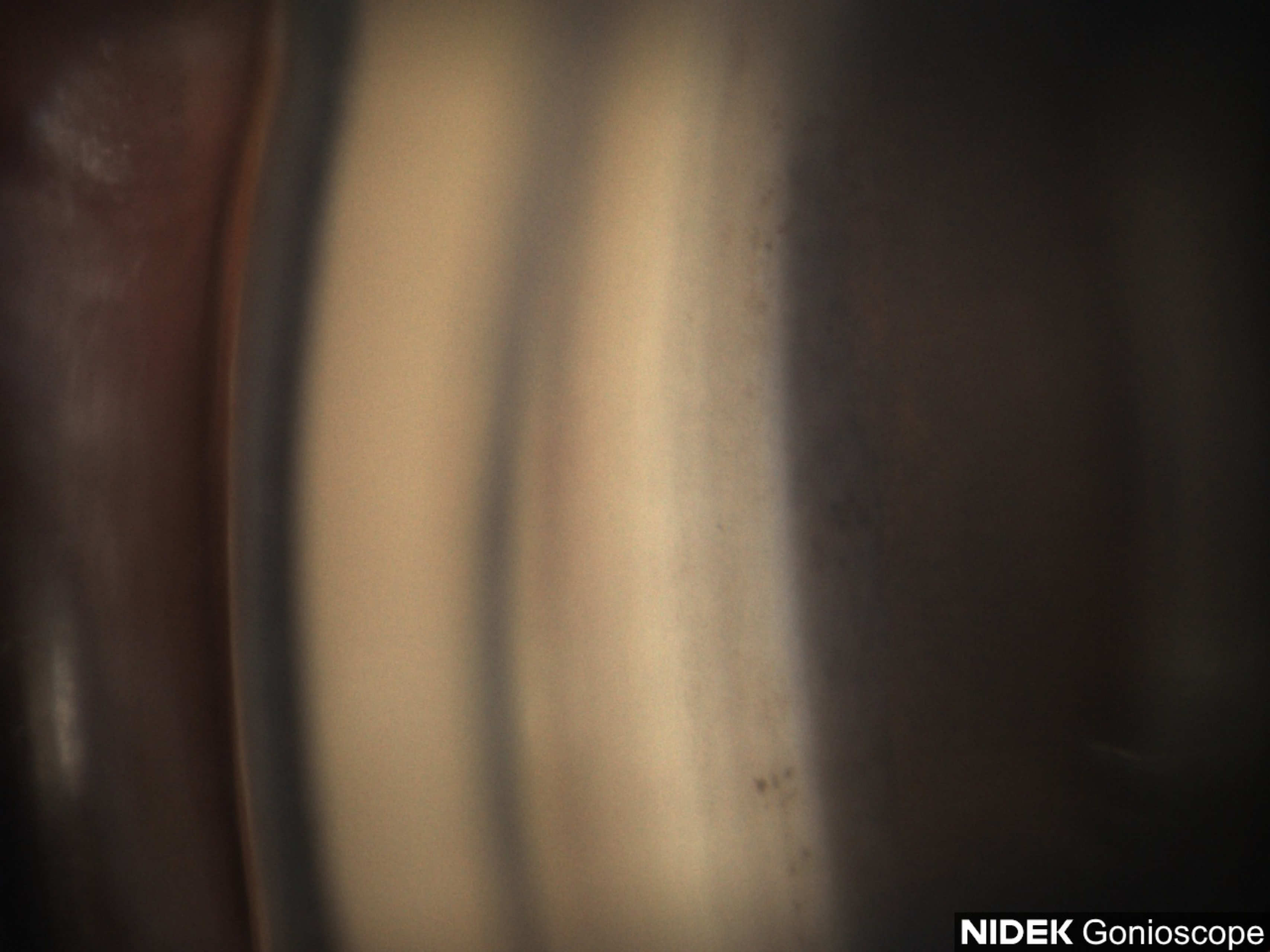

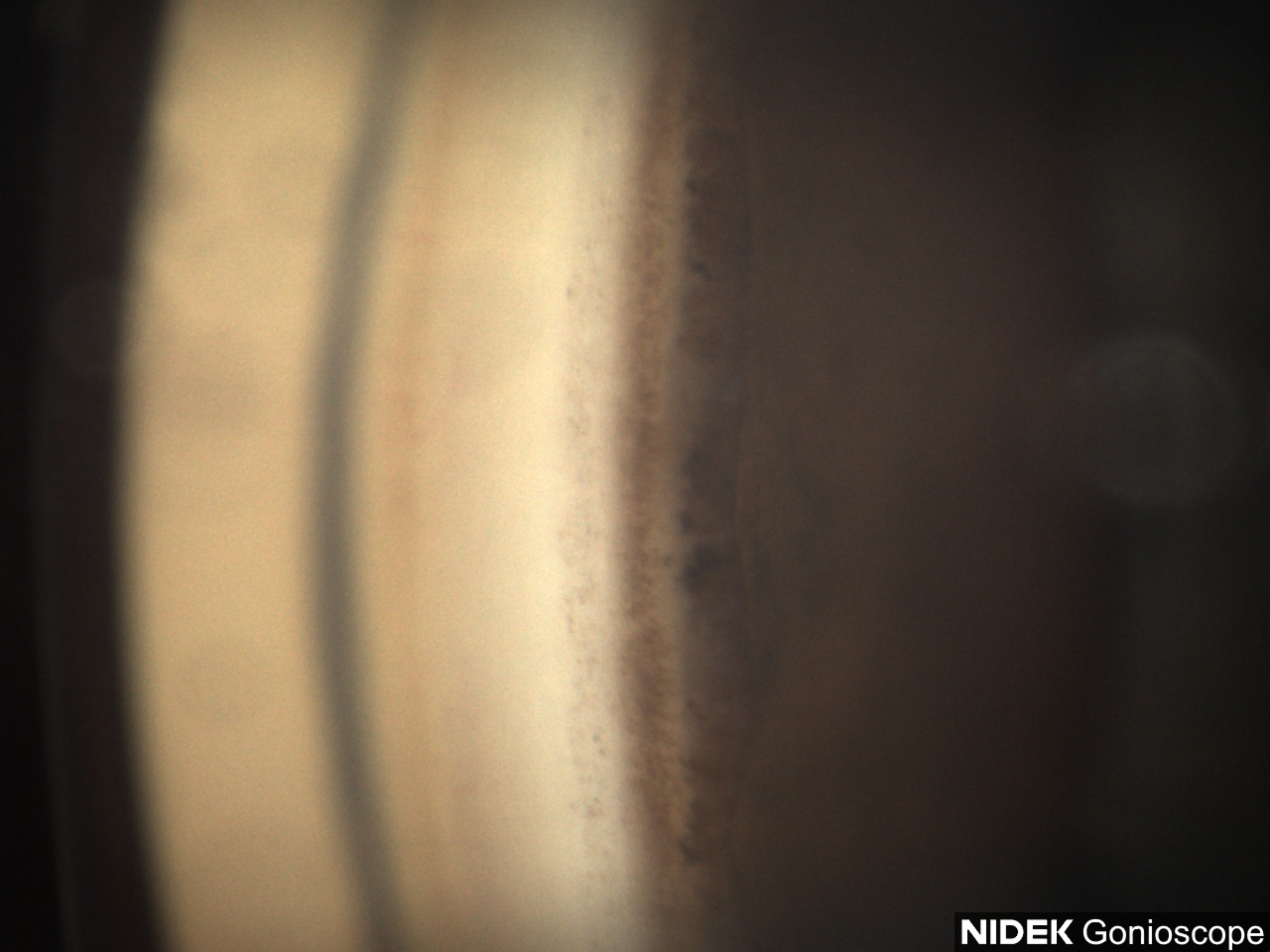

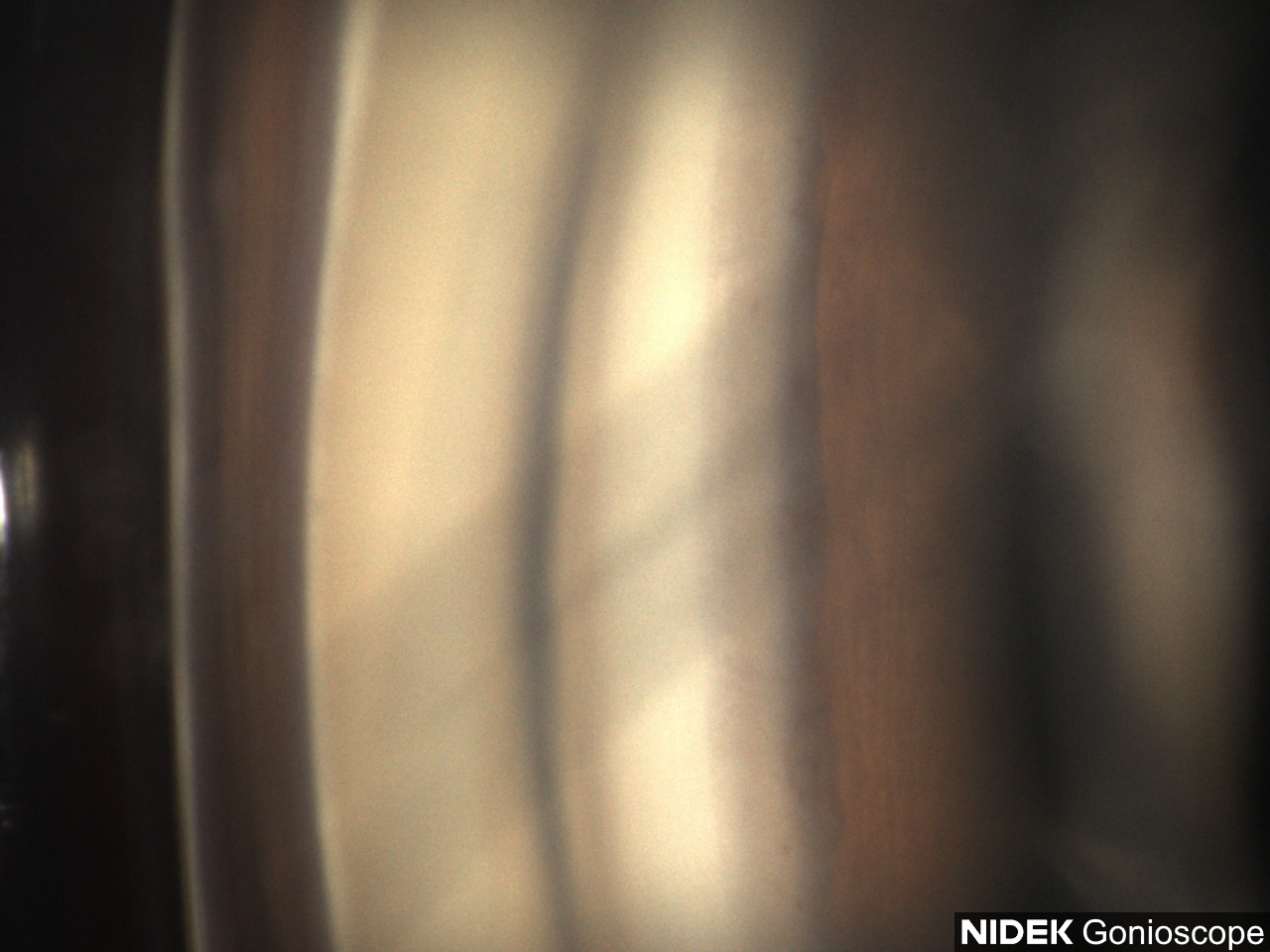

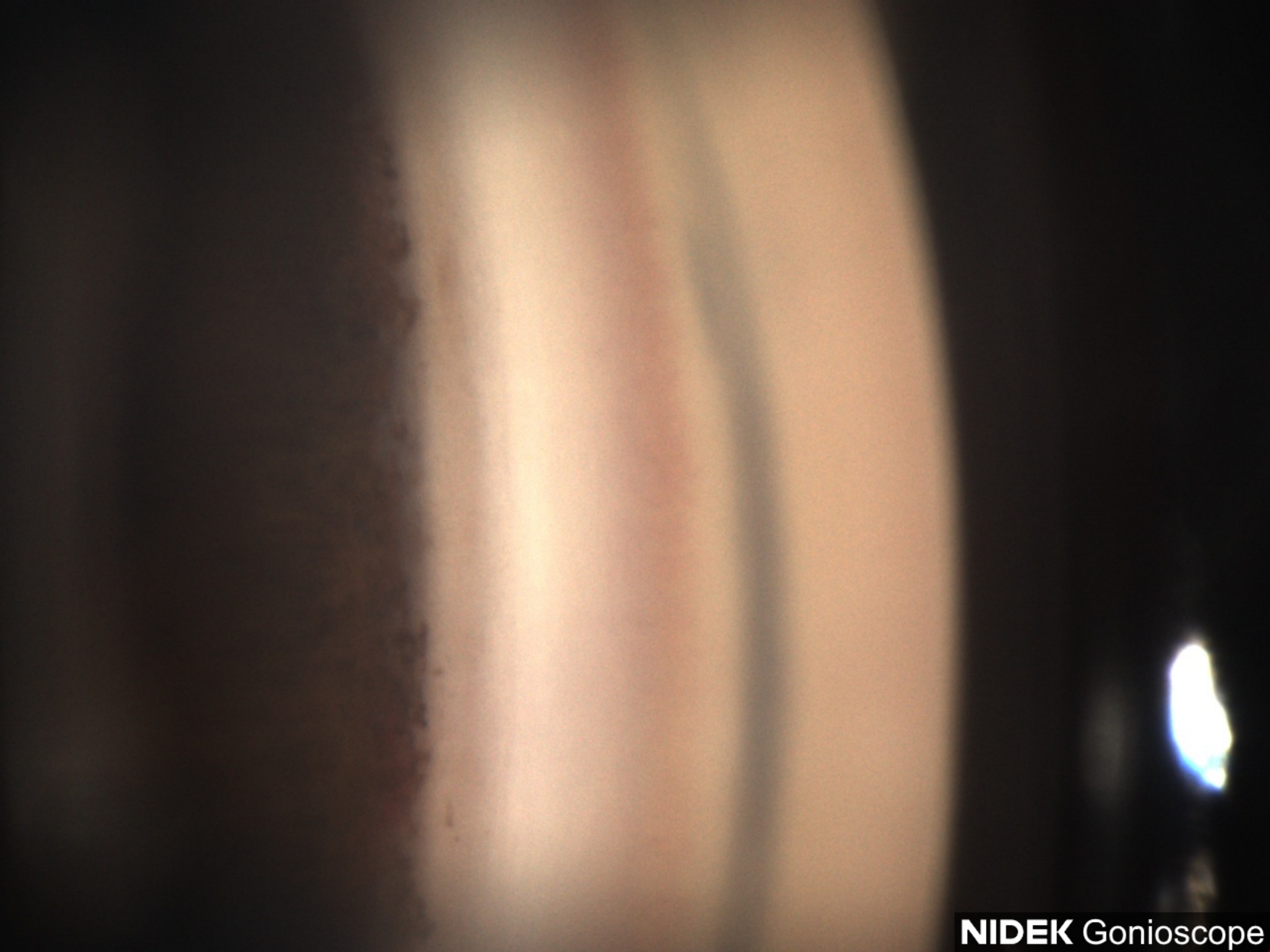

**NIDEK** Gonioscope

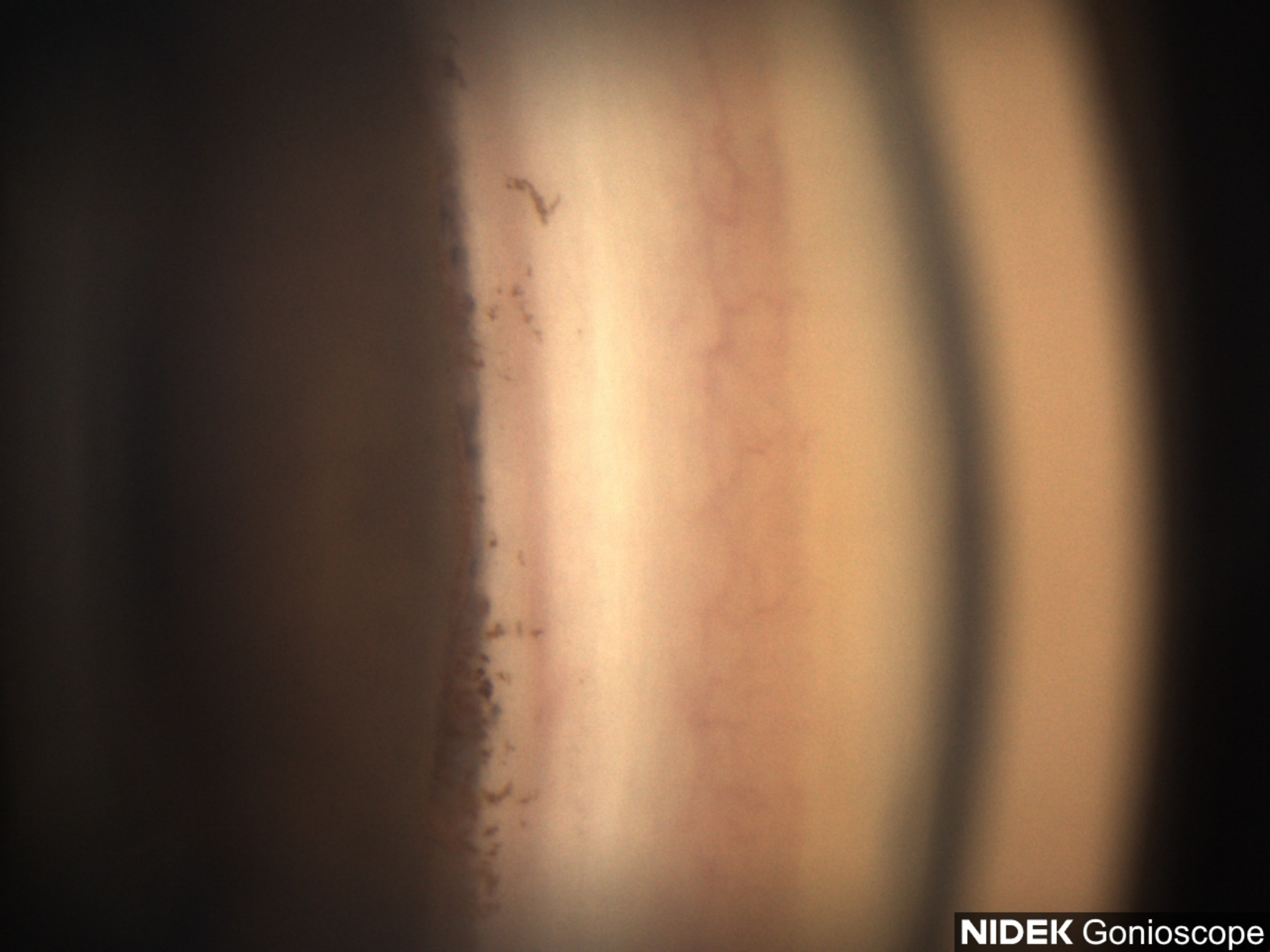

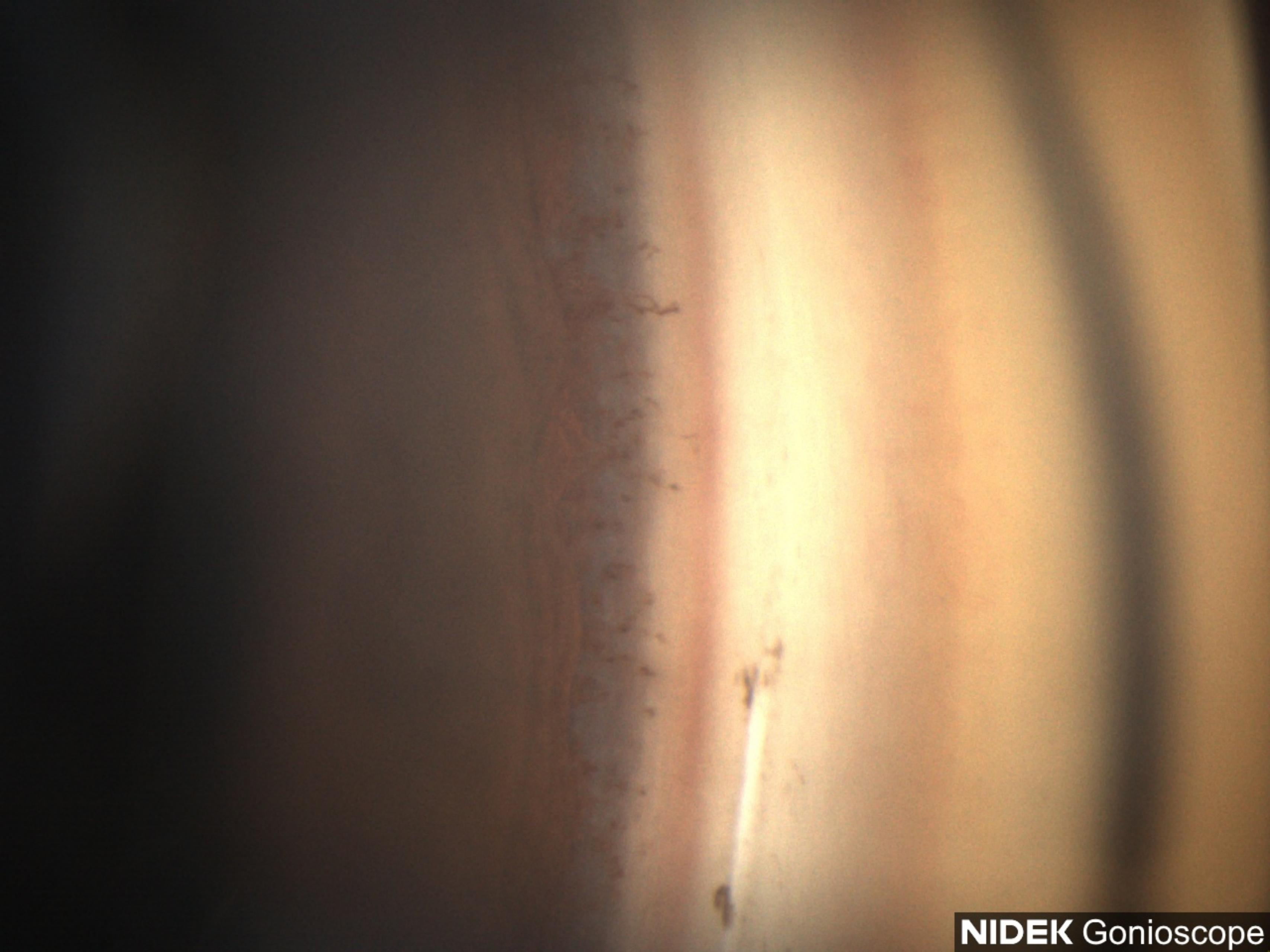

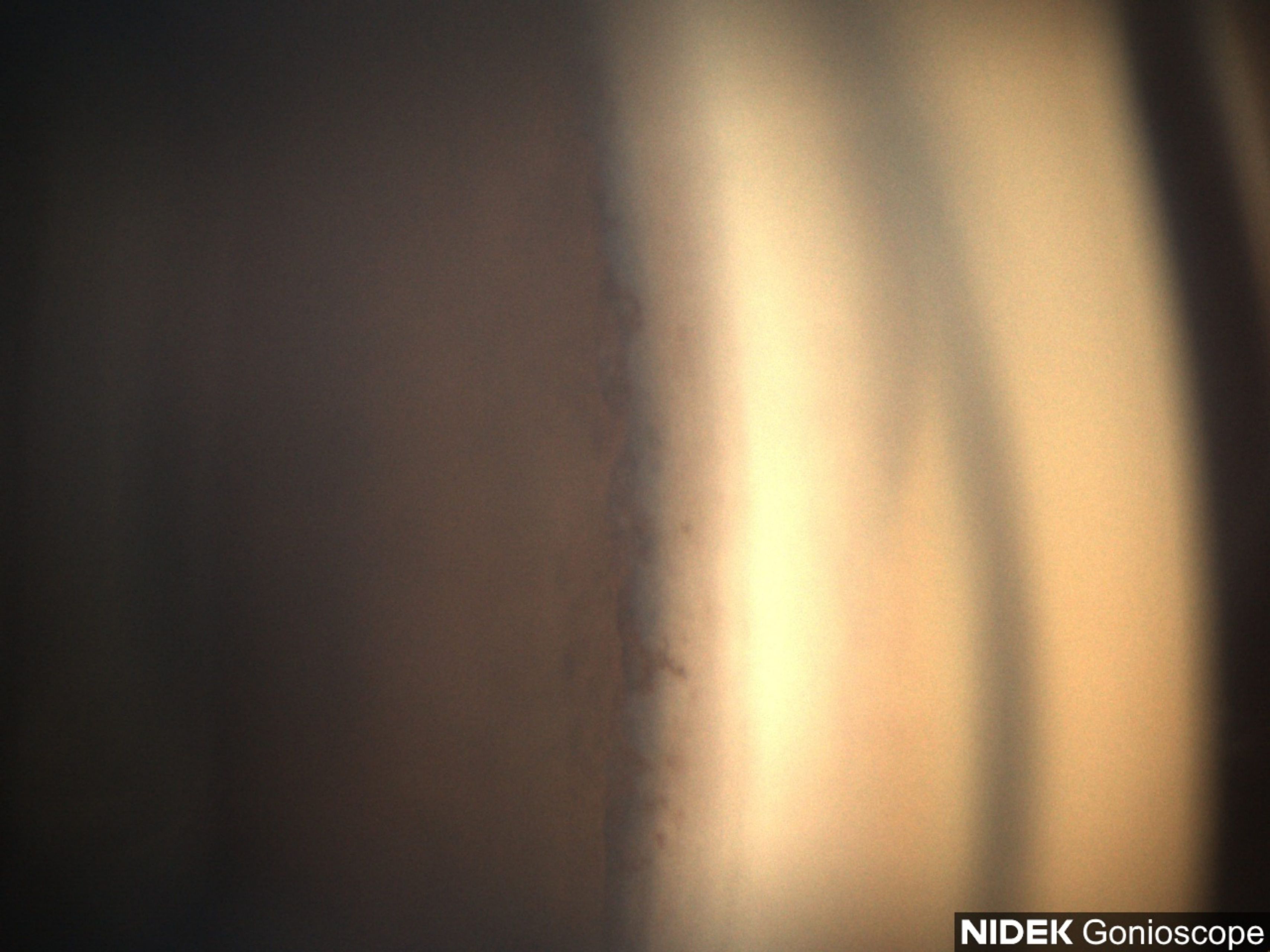



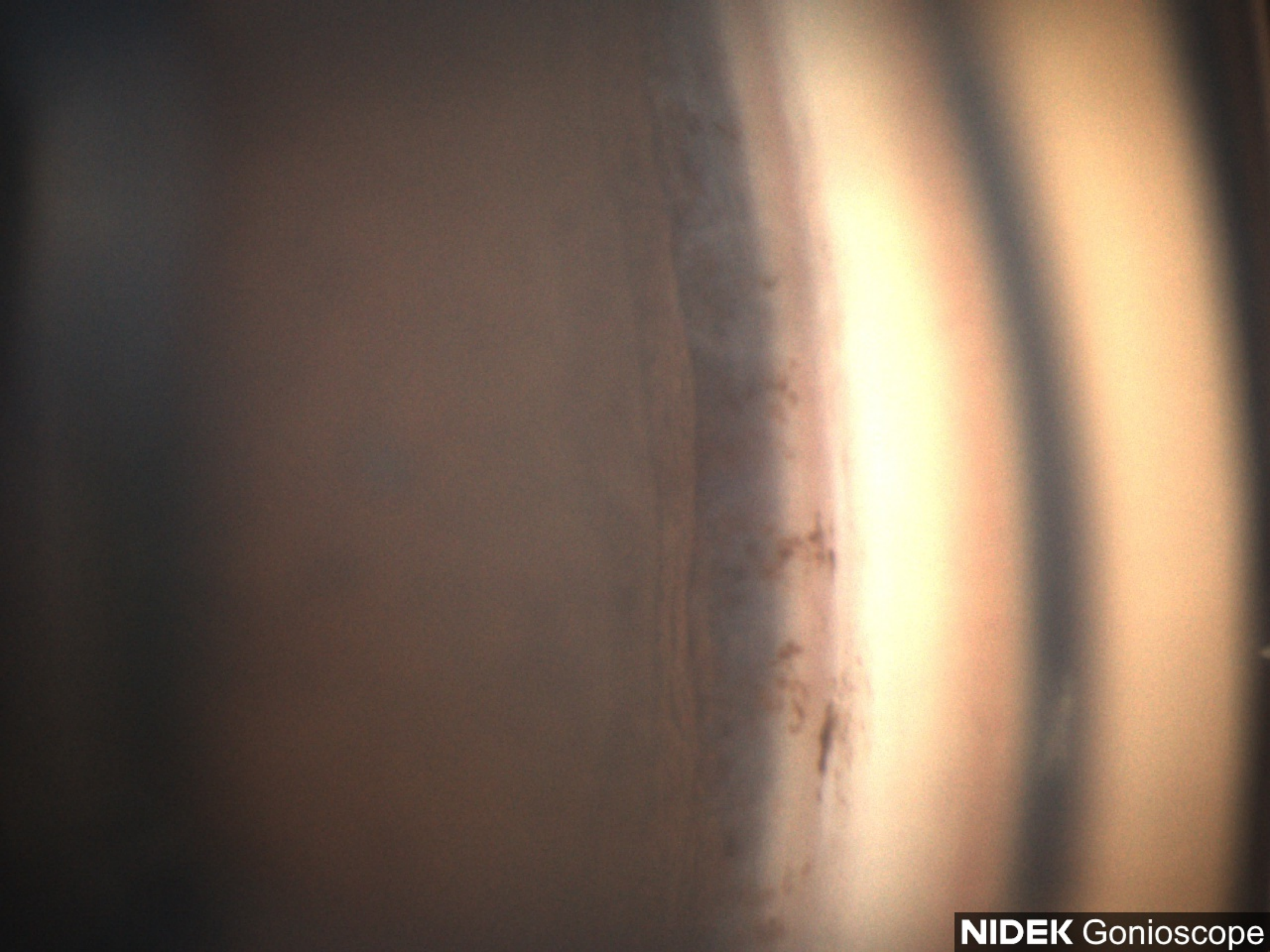

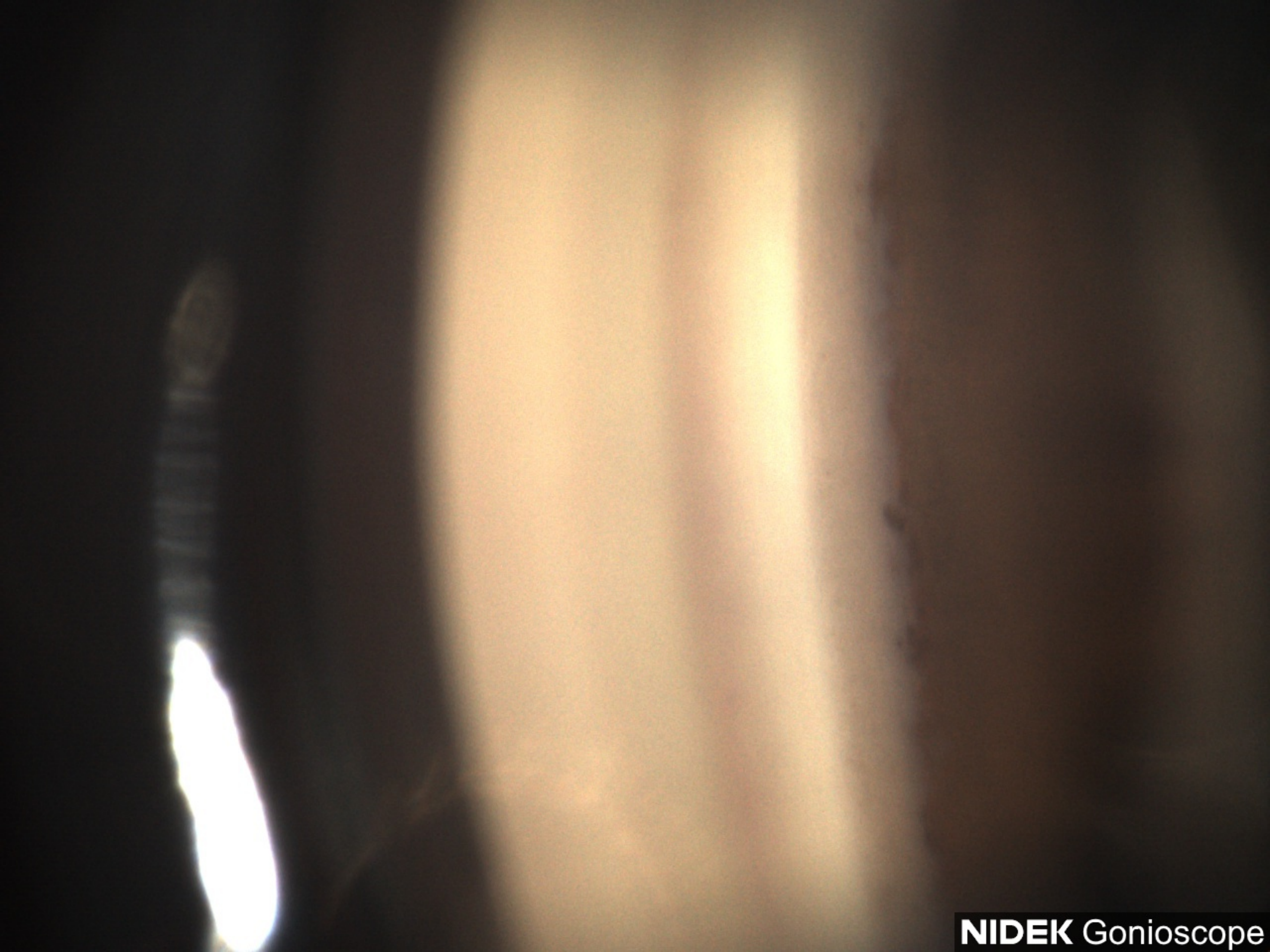

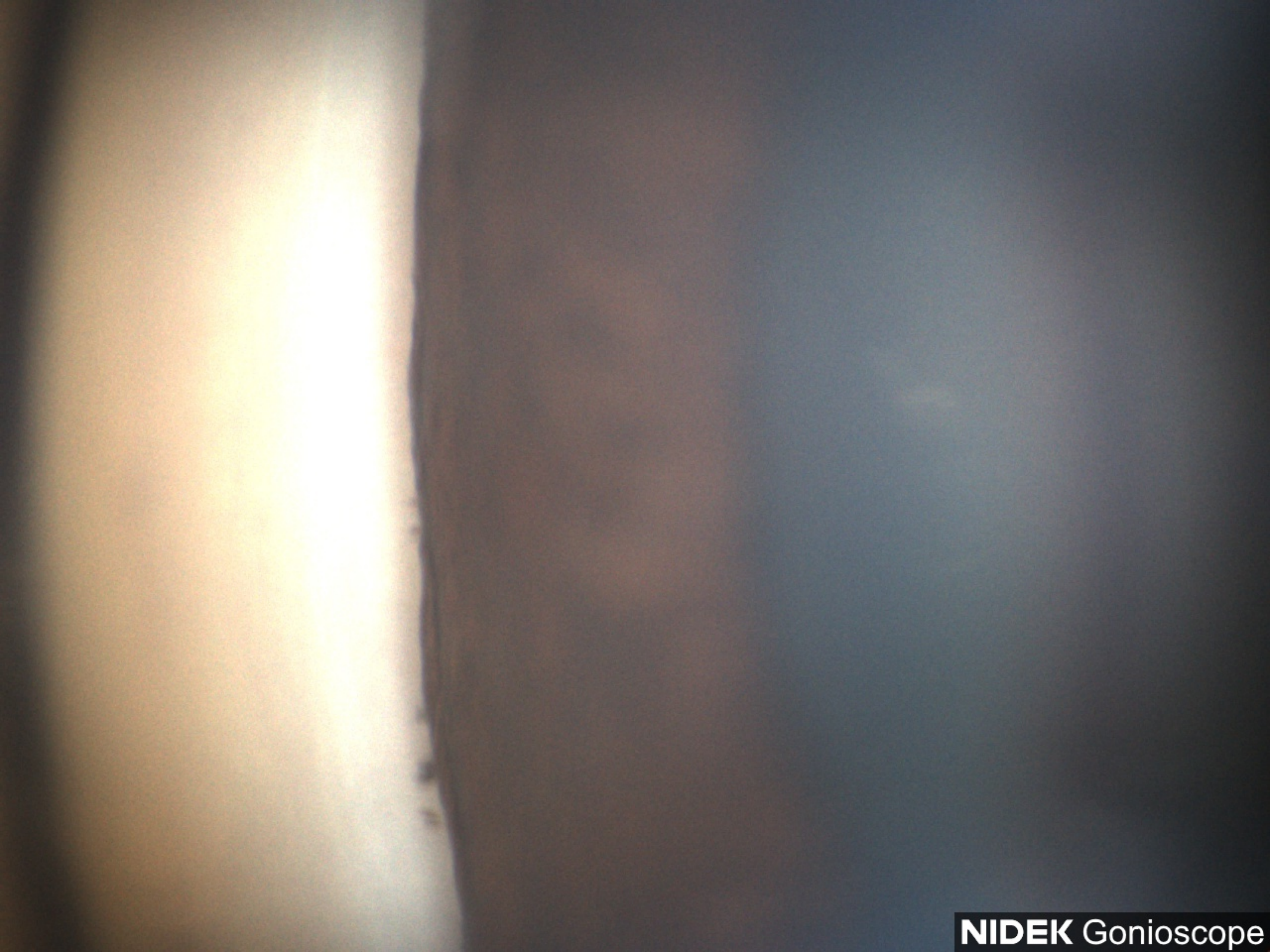

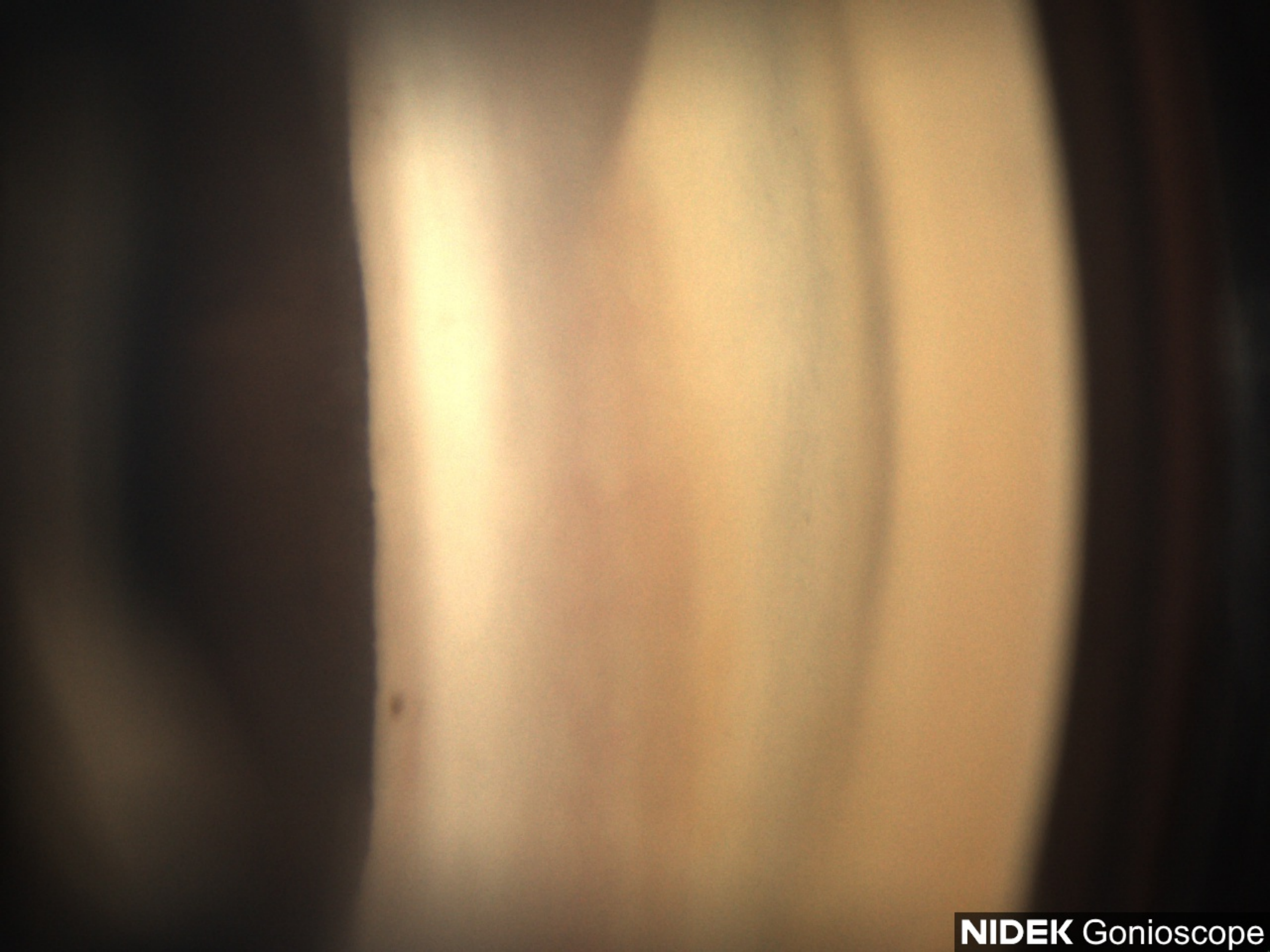



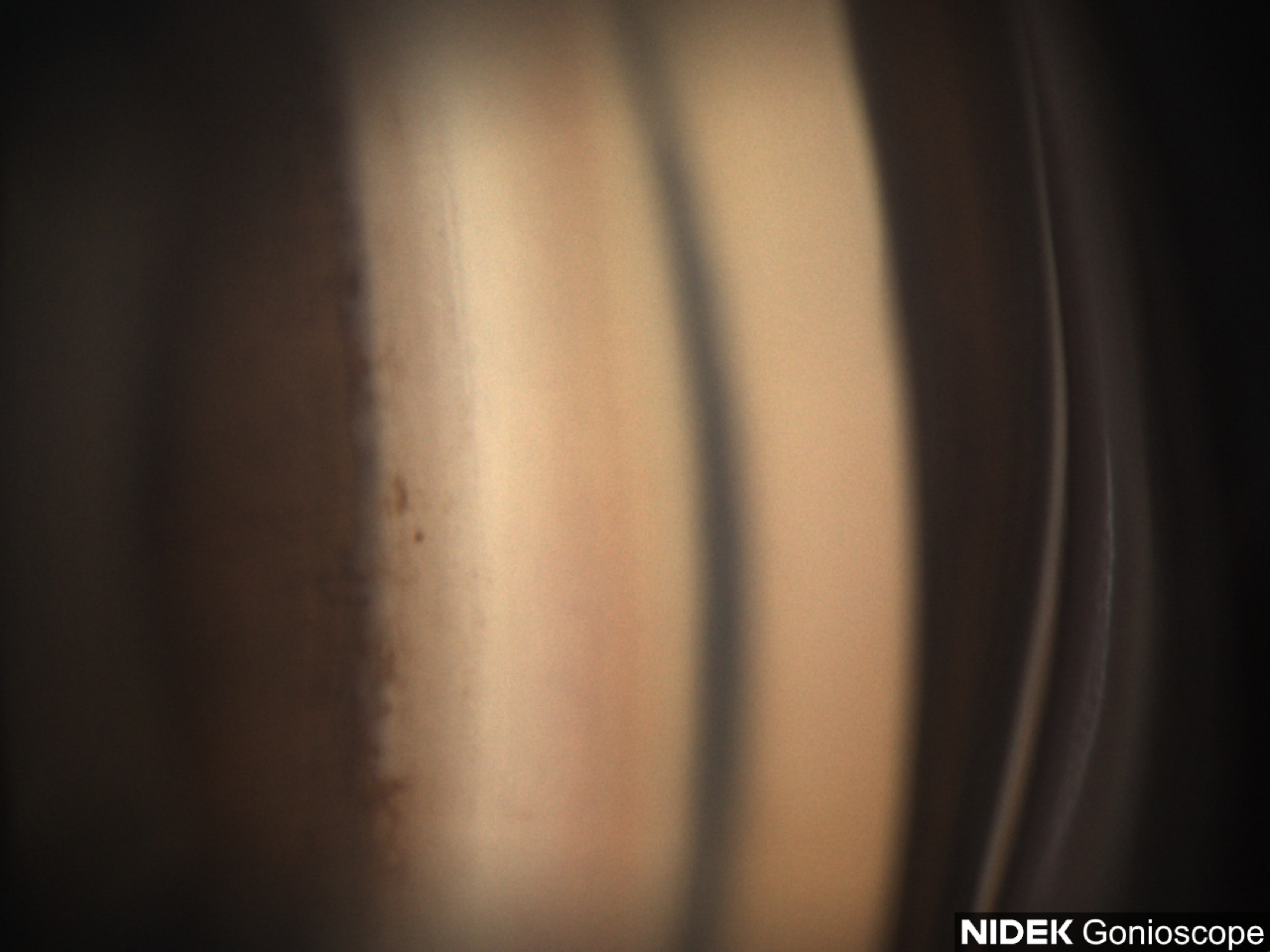

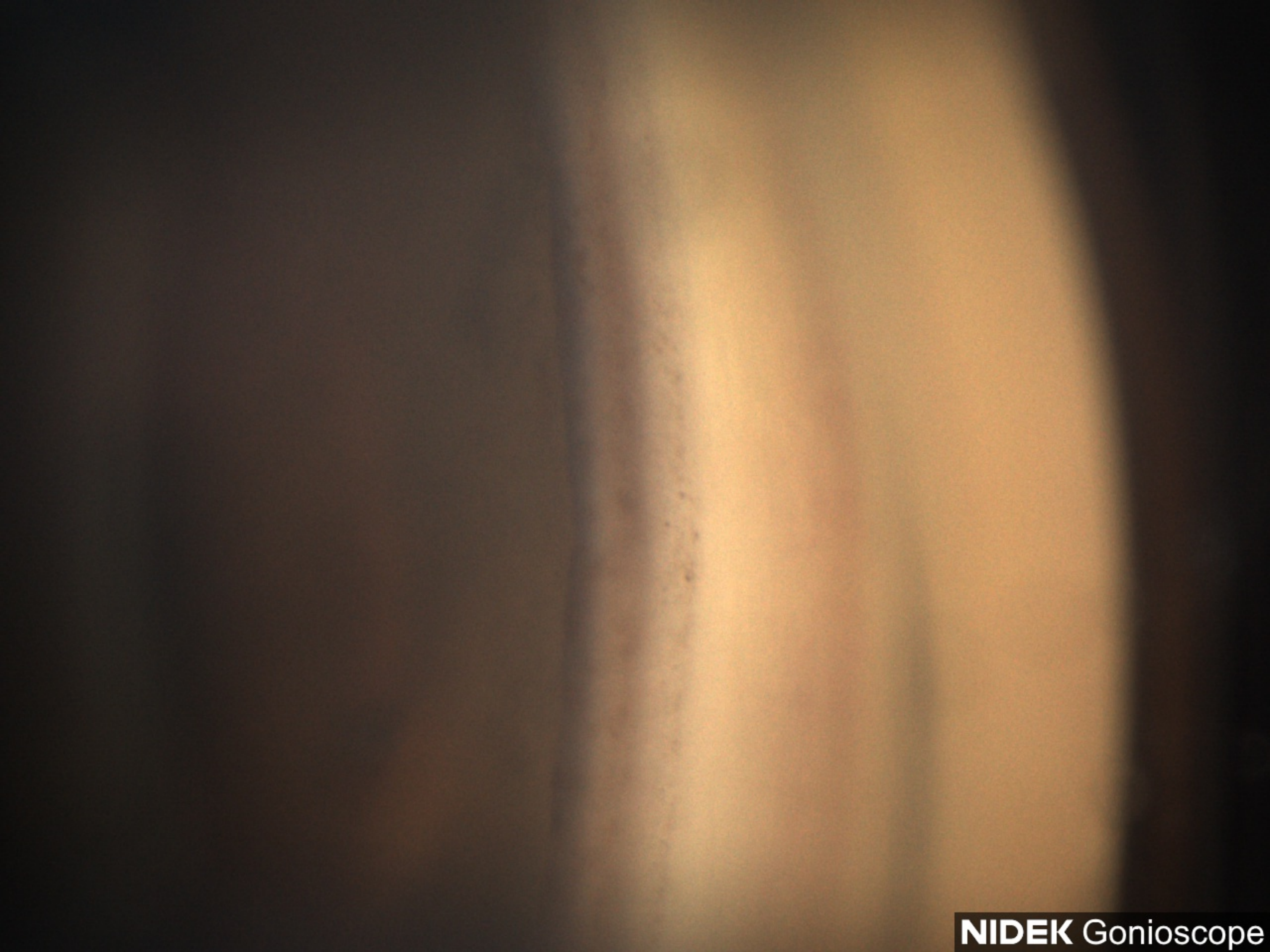

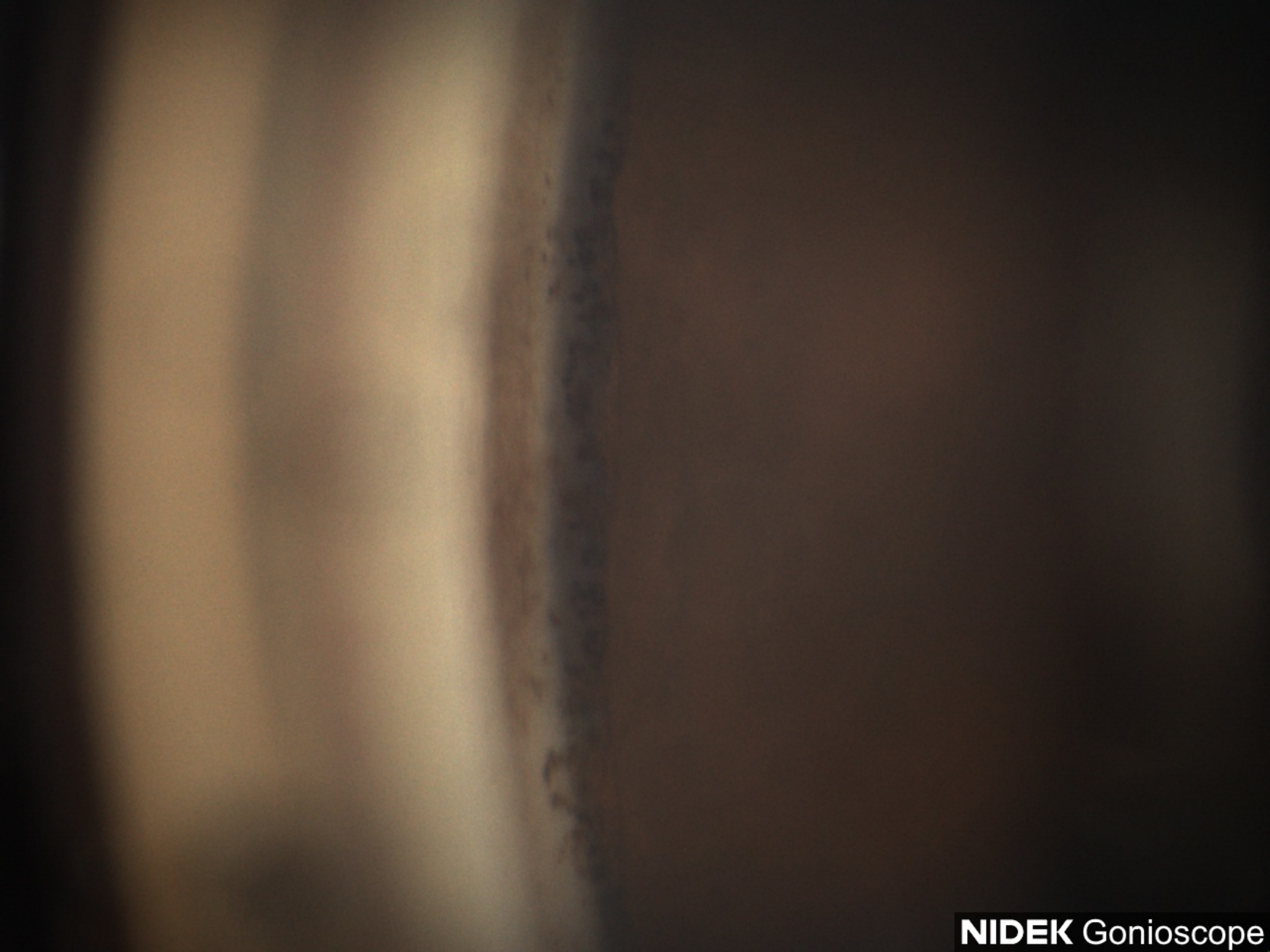

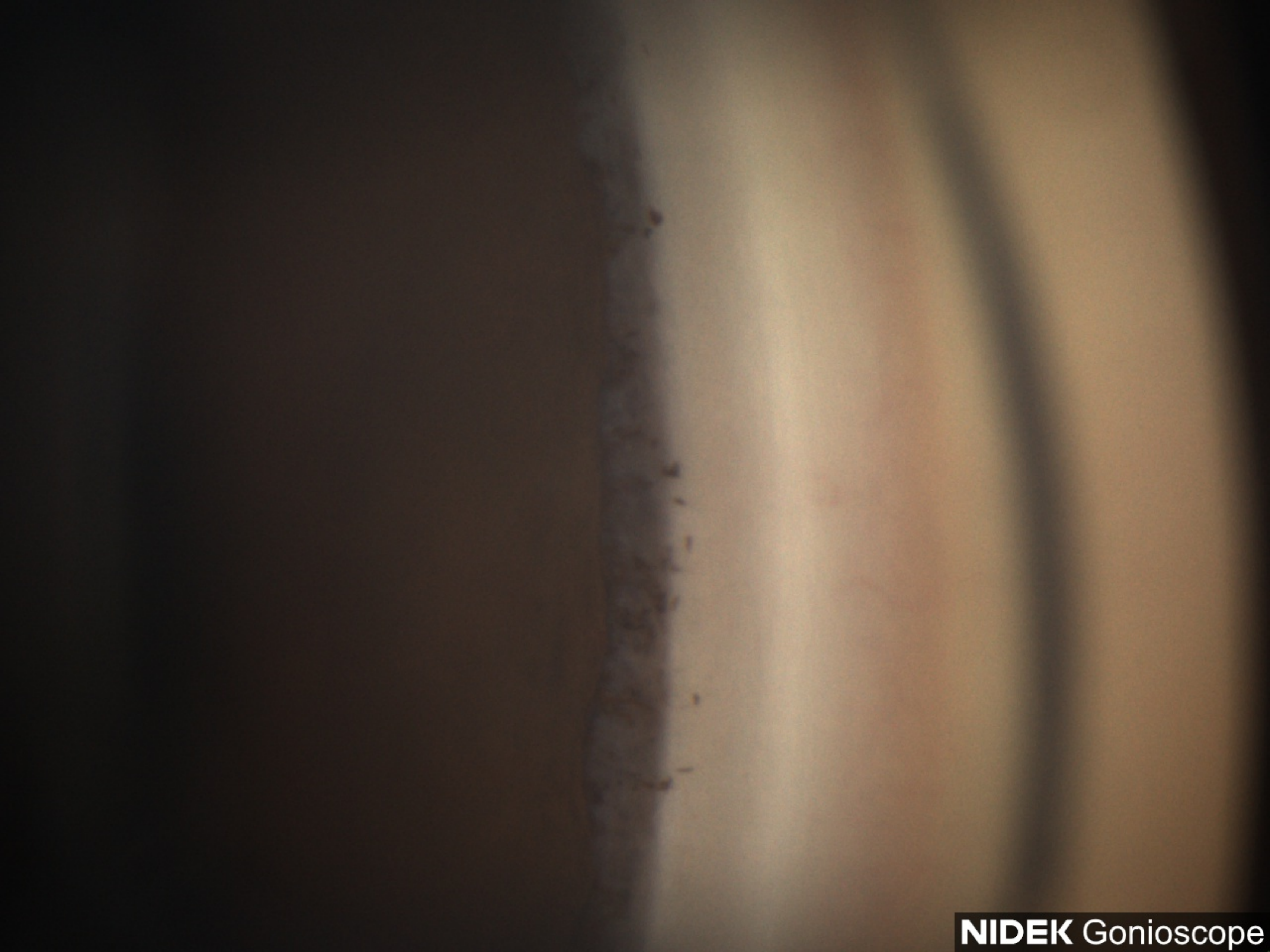



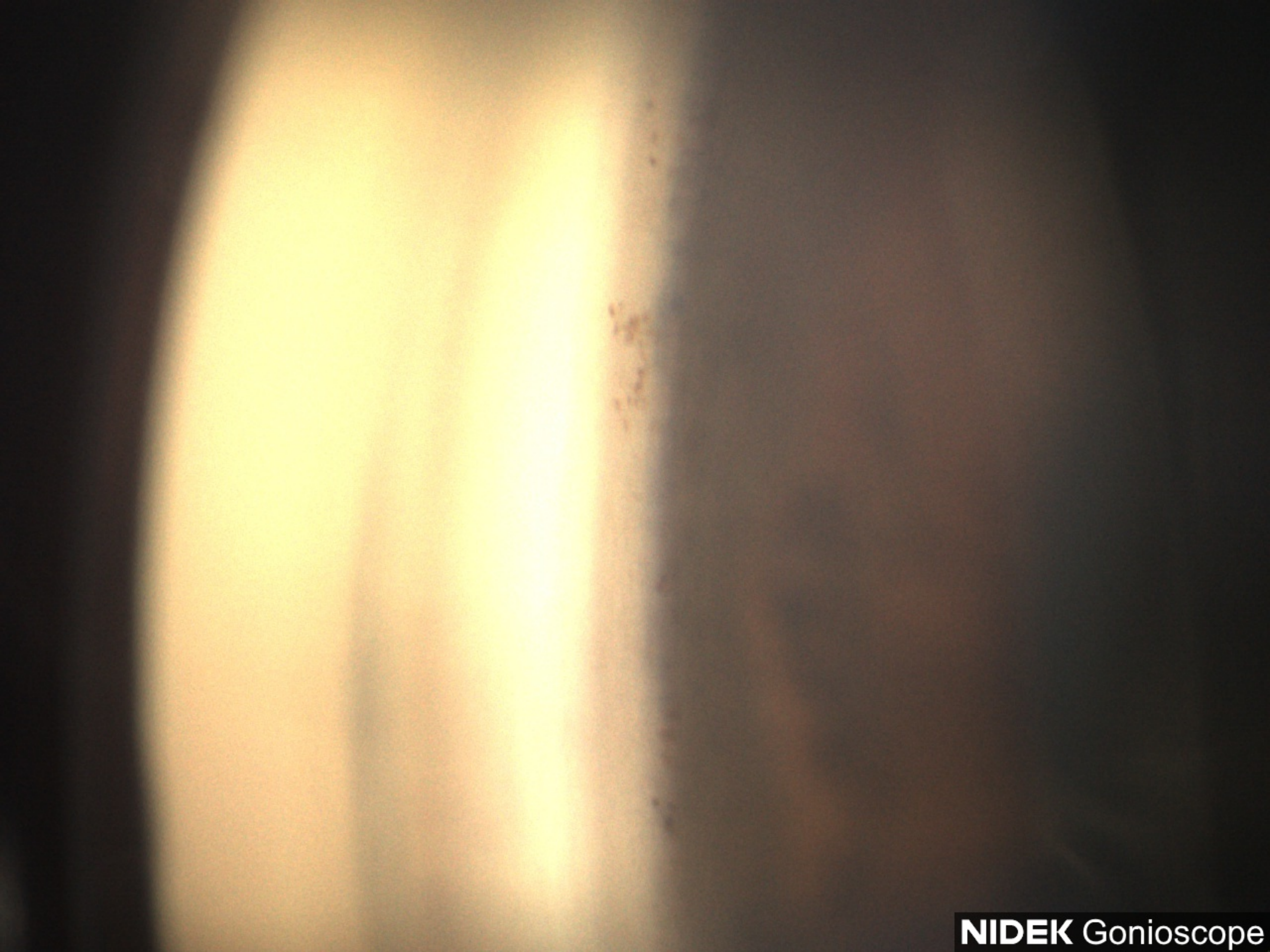

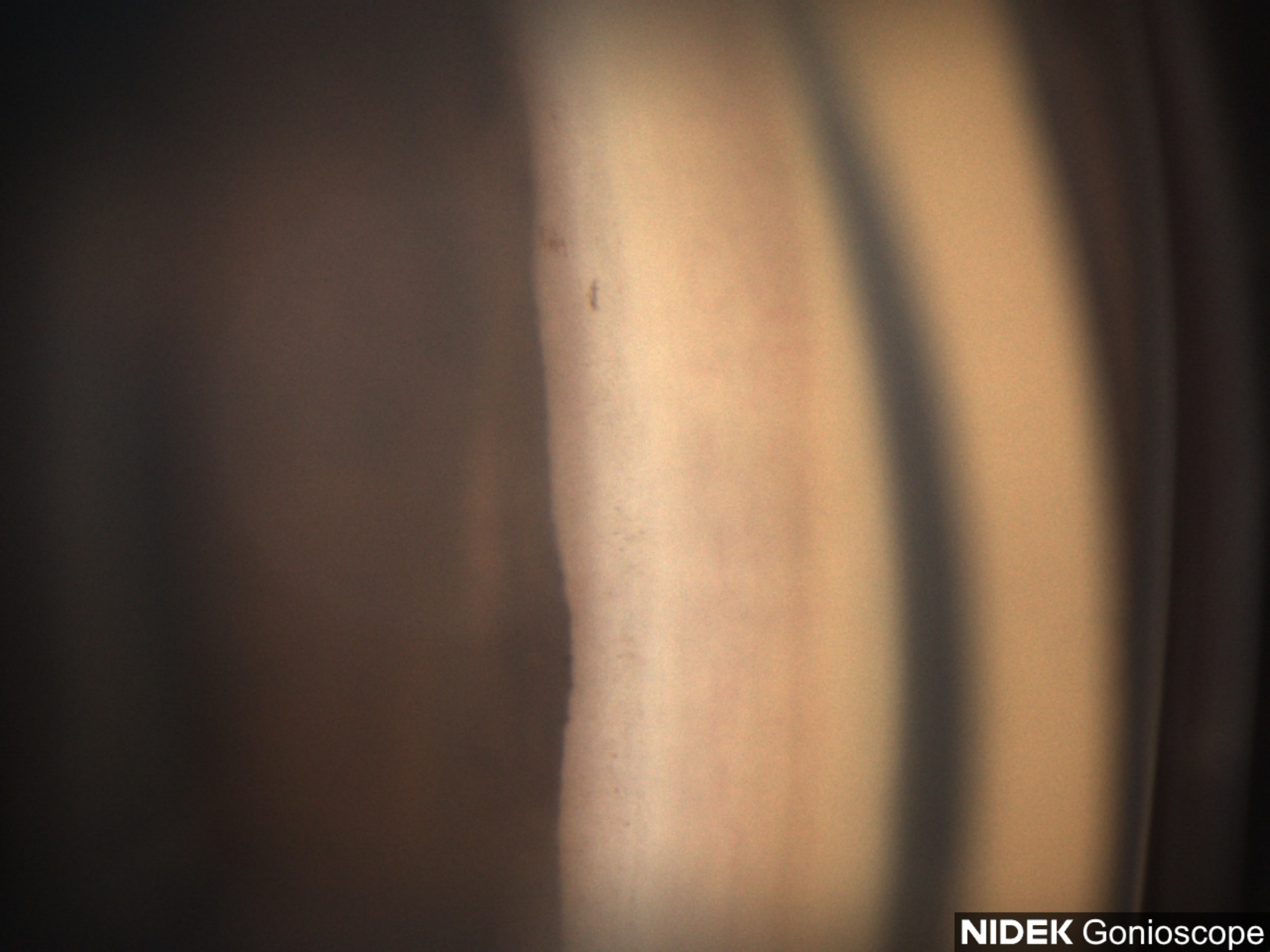

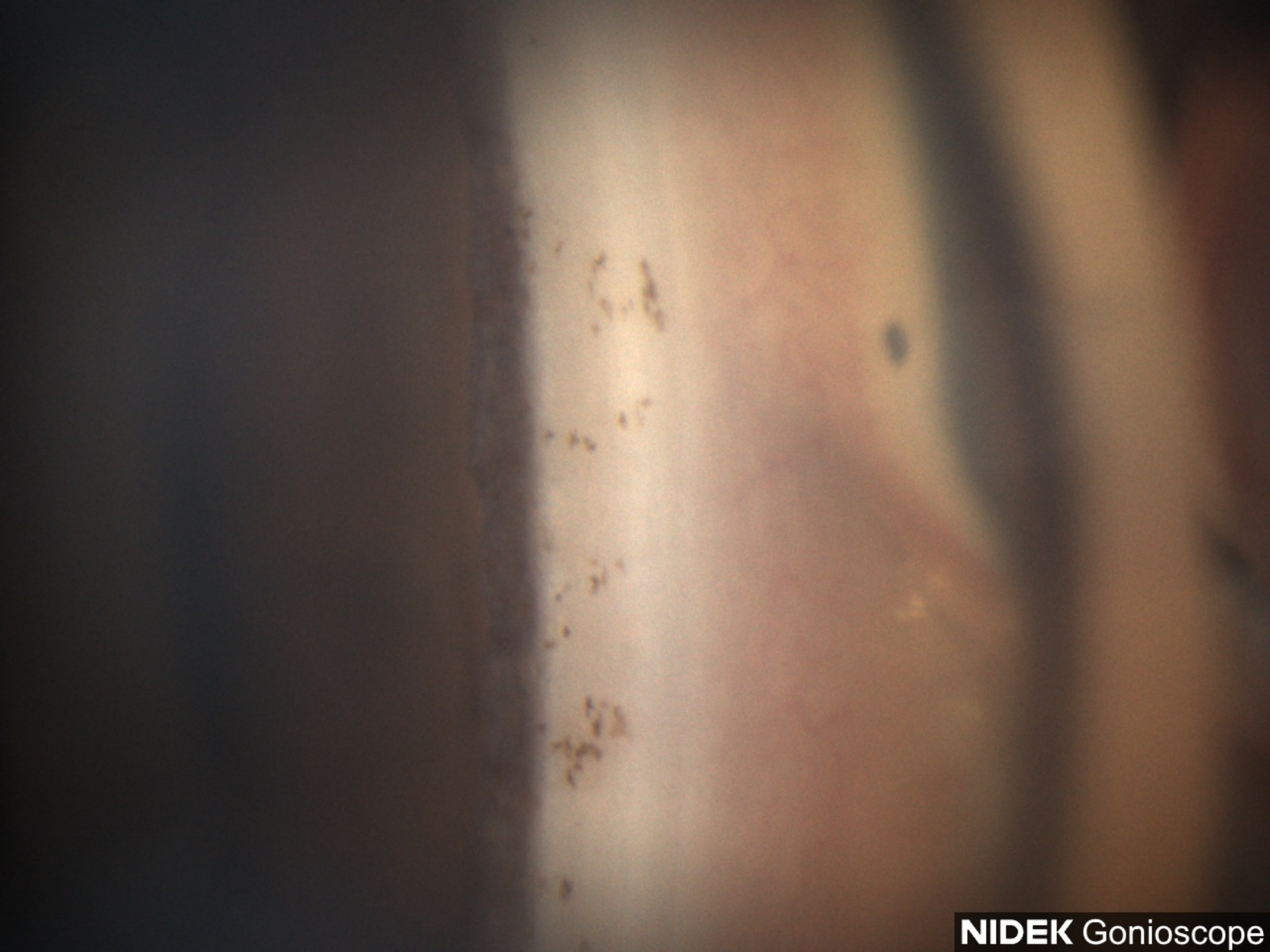

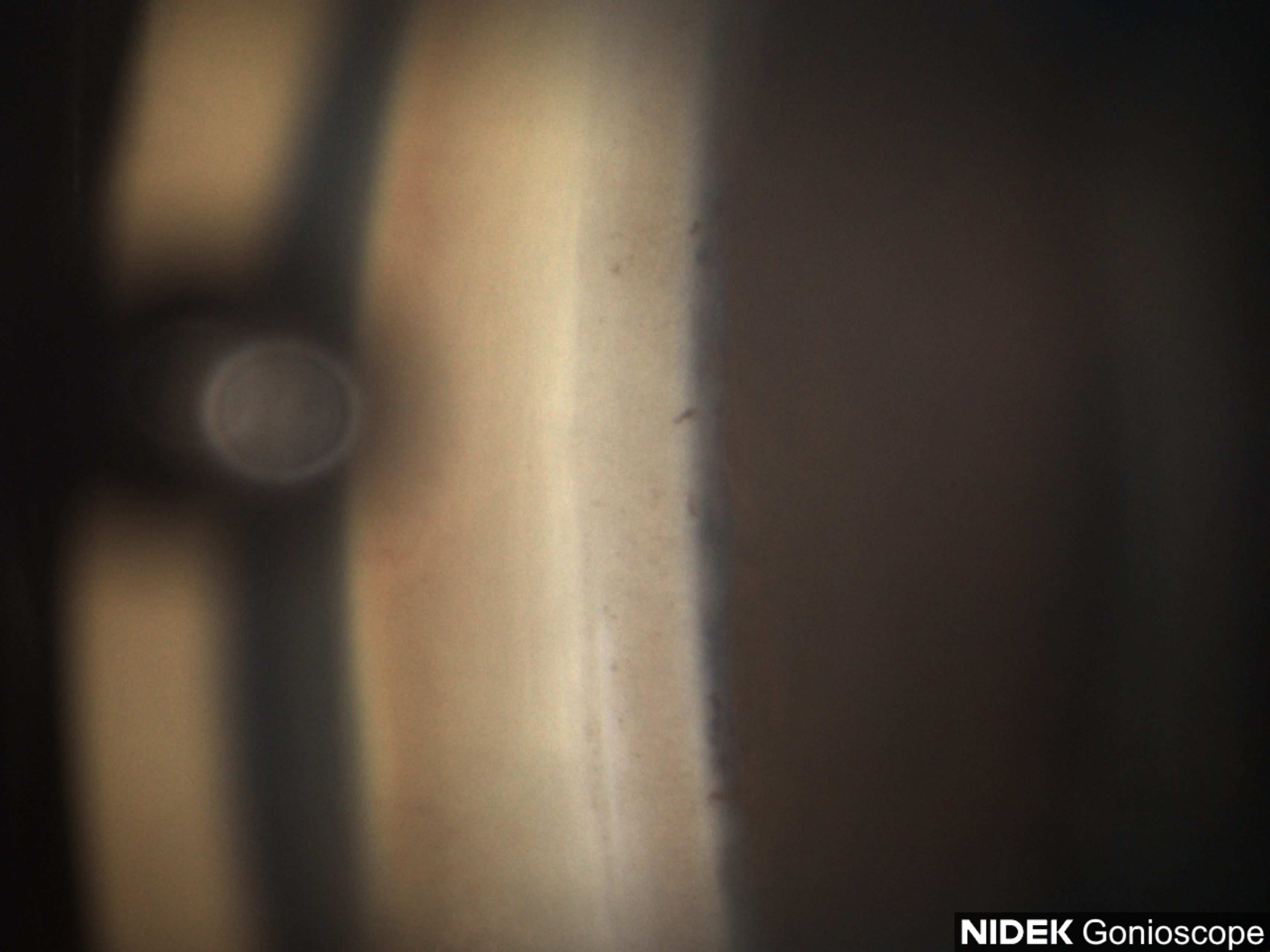

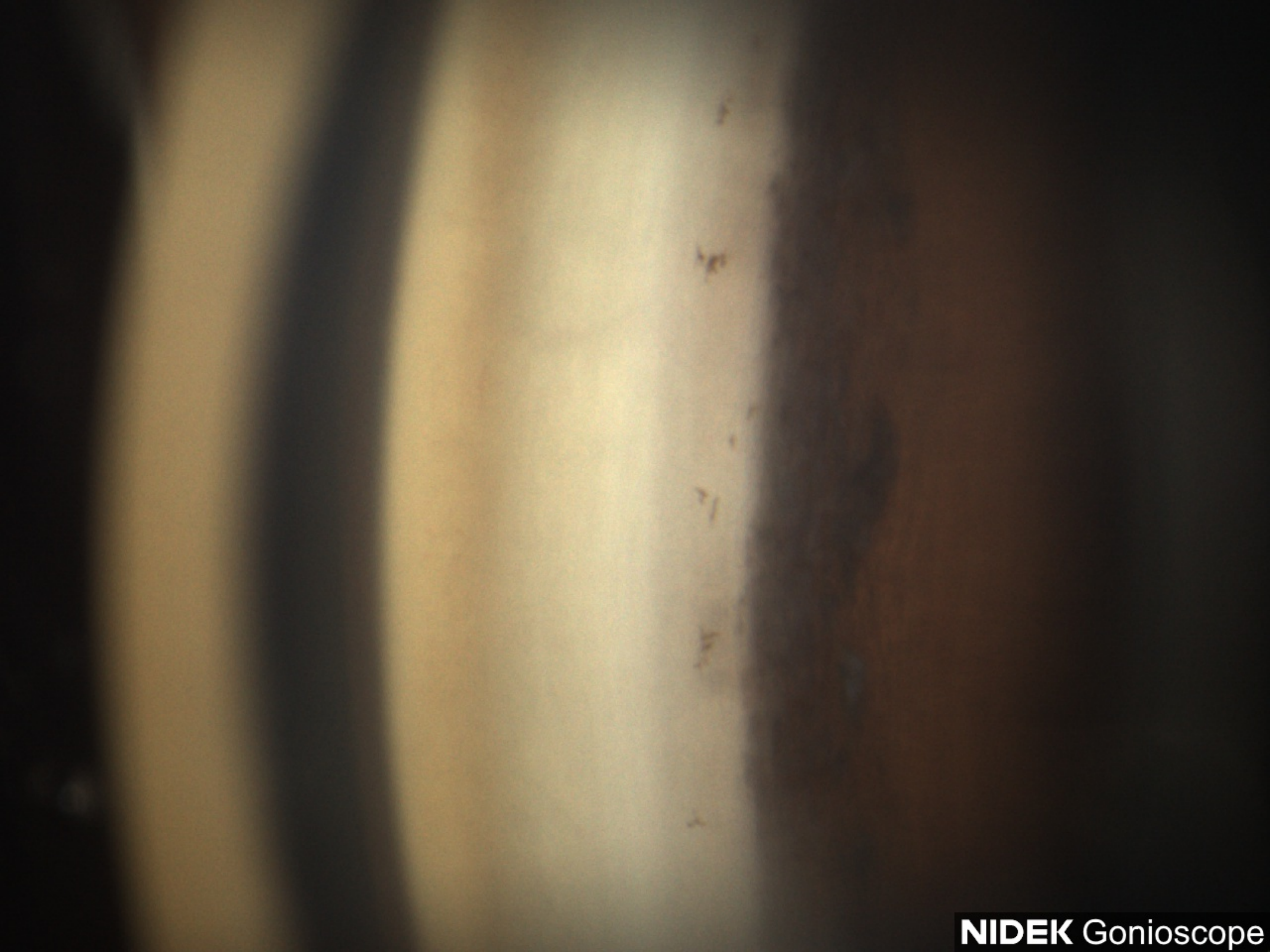

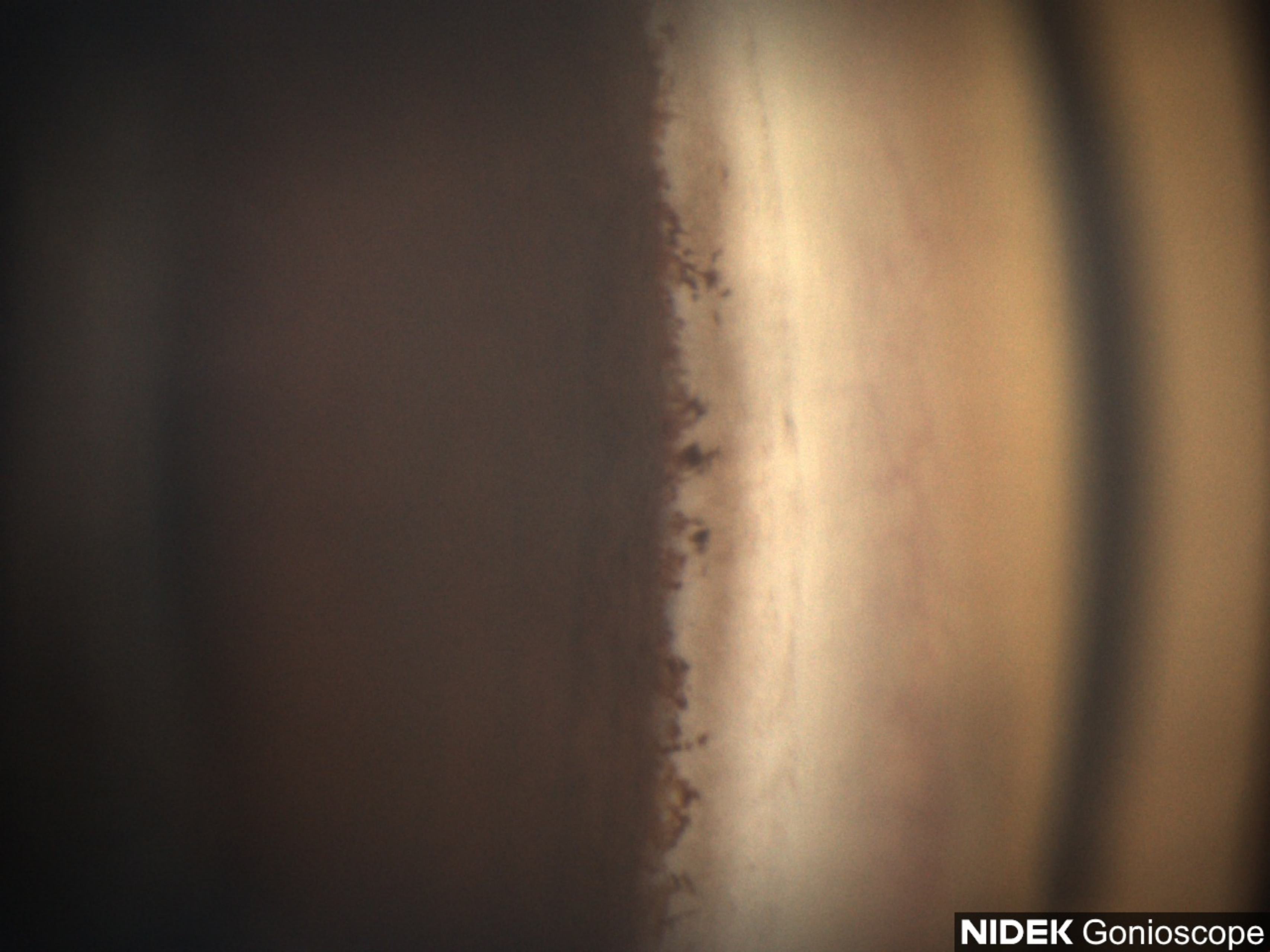

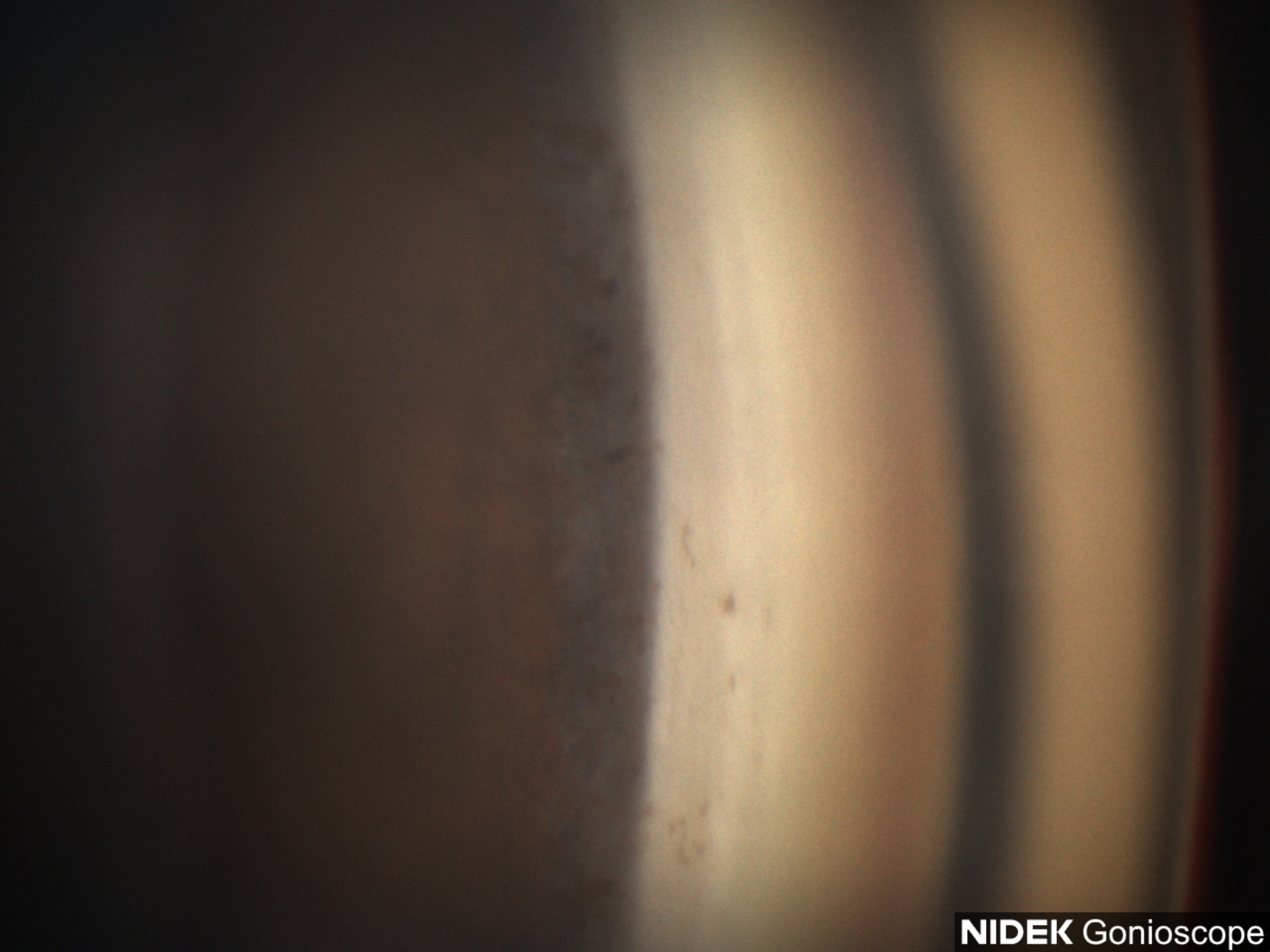

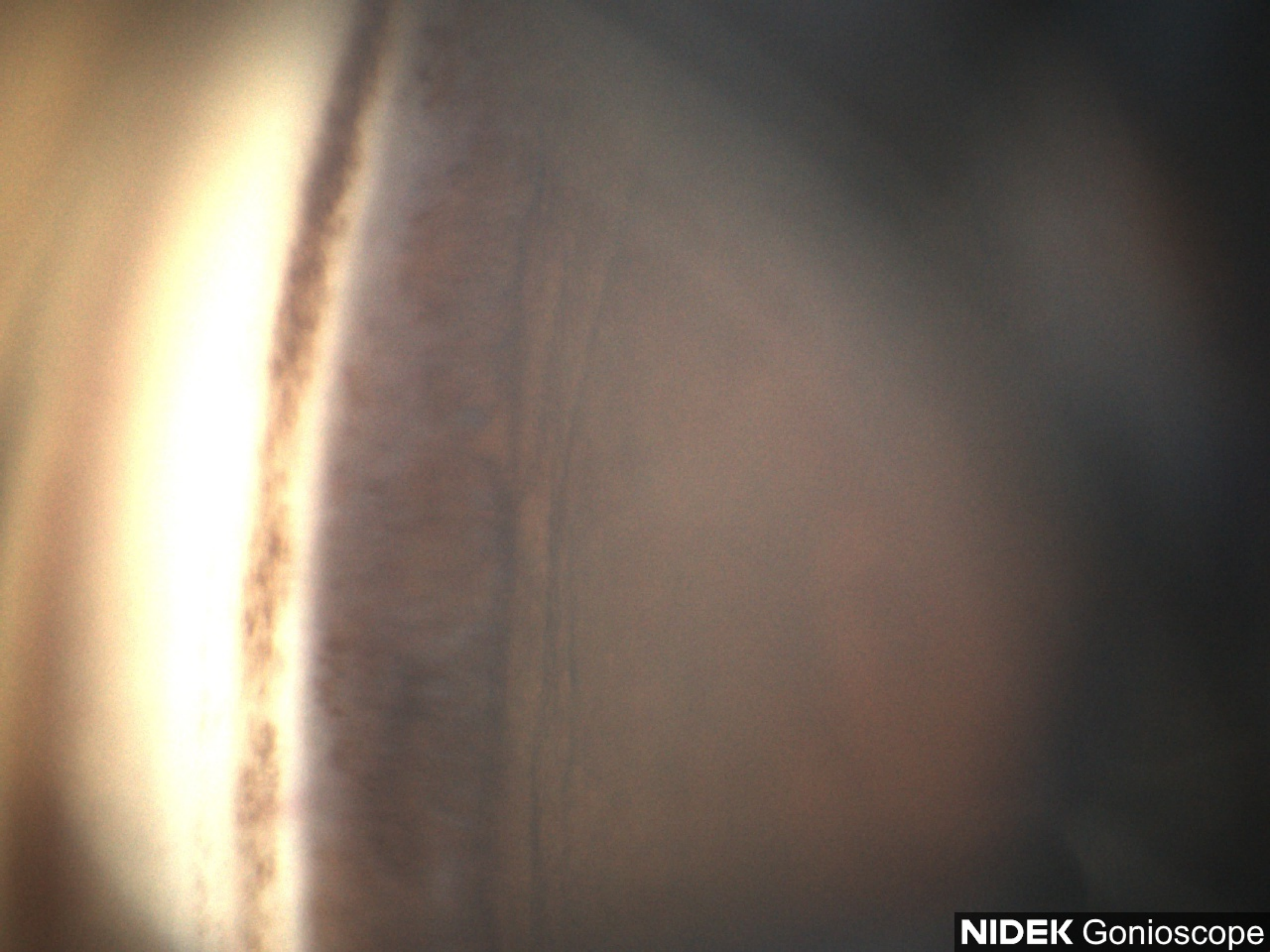



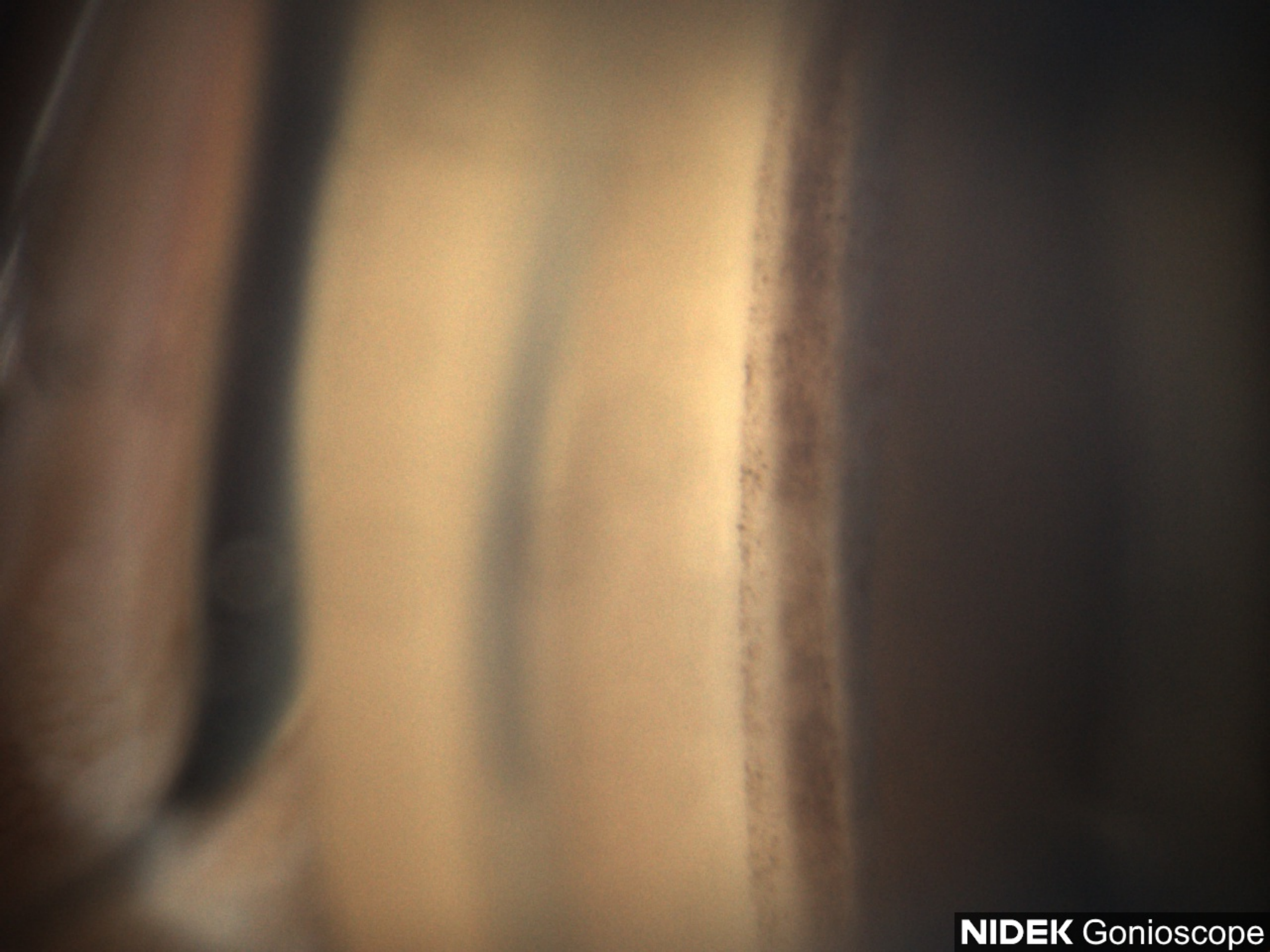

**NIDEK** Gonioscope

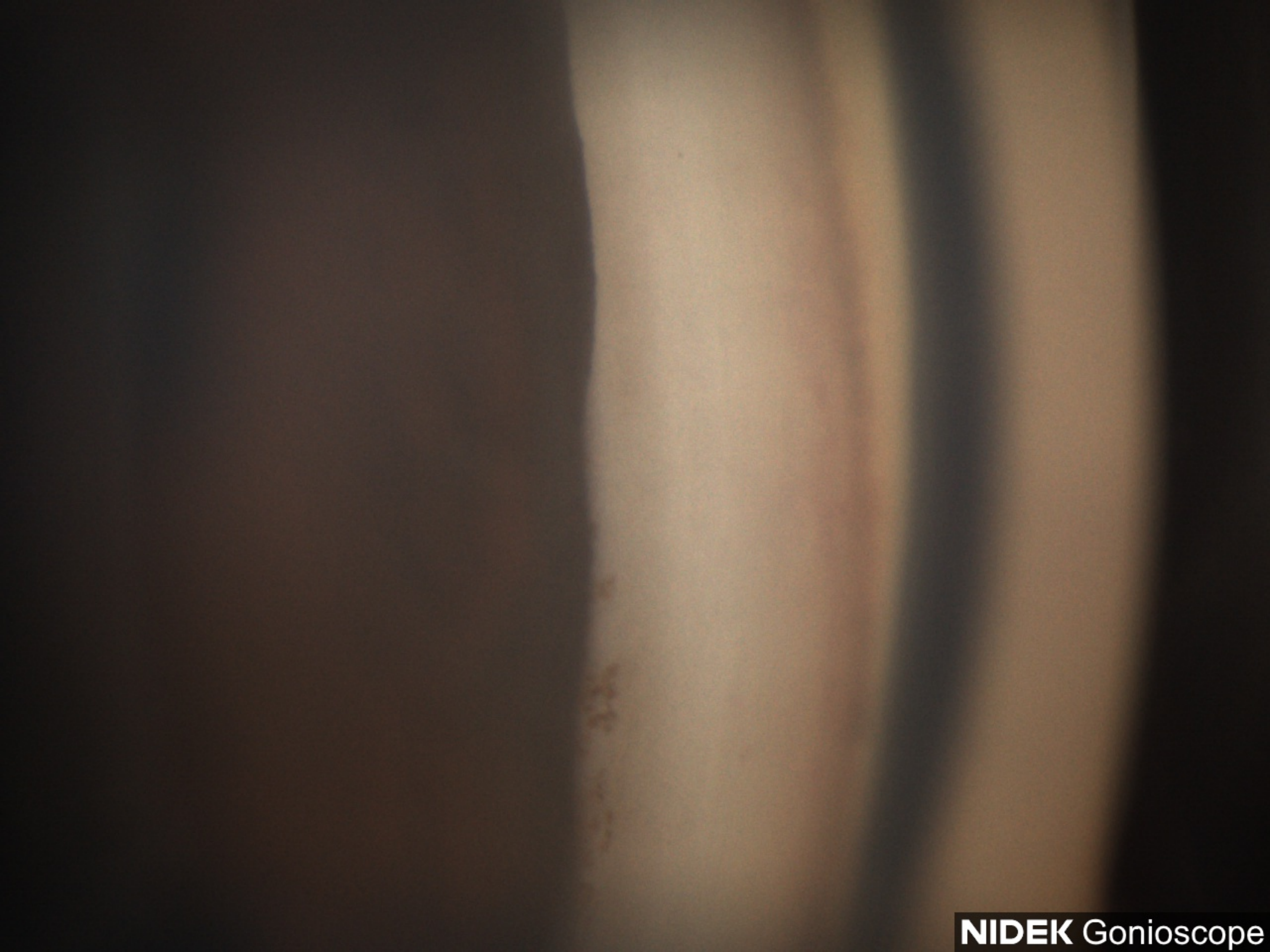

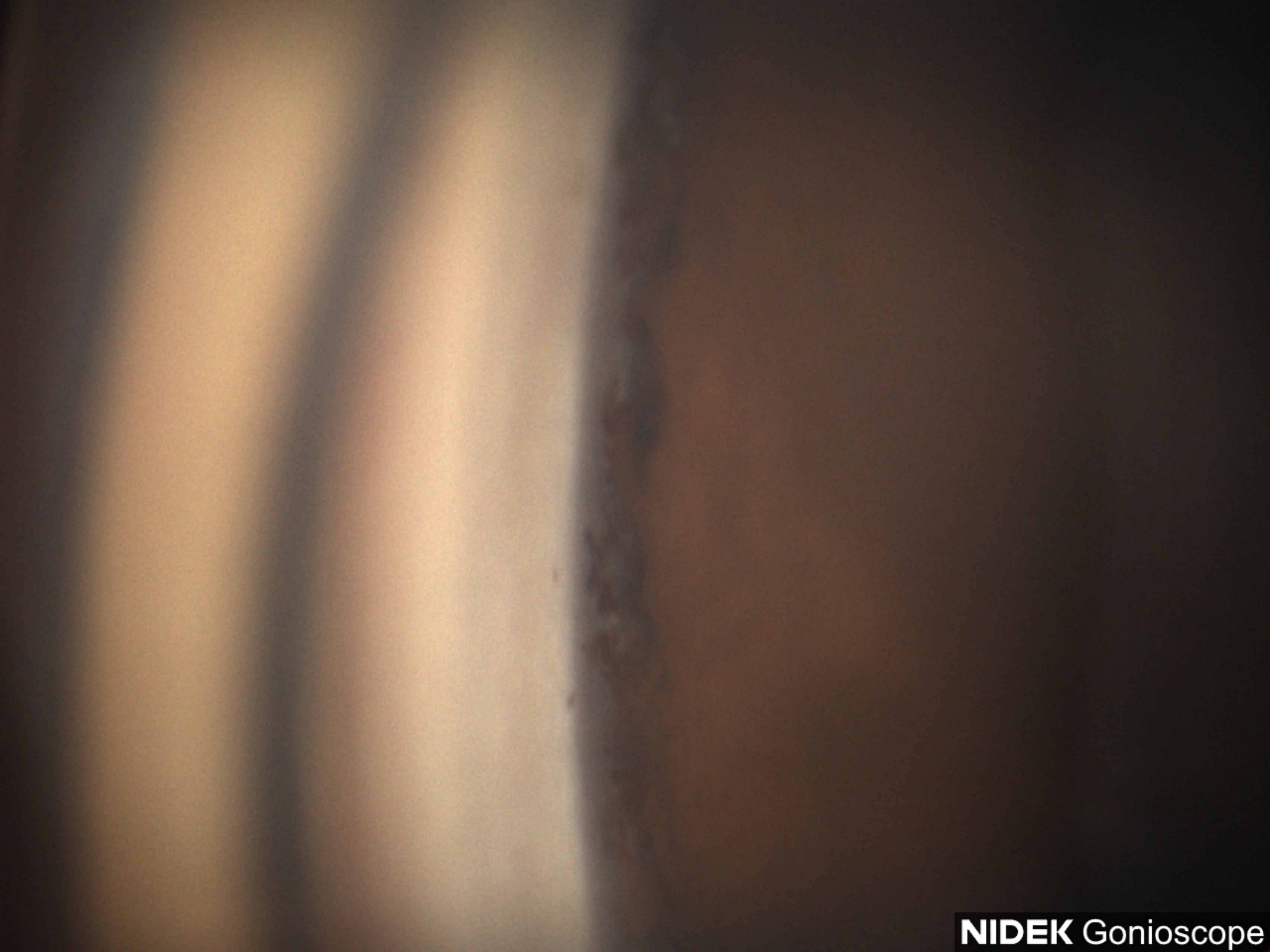

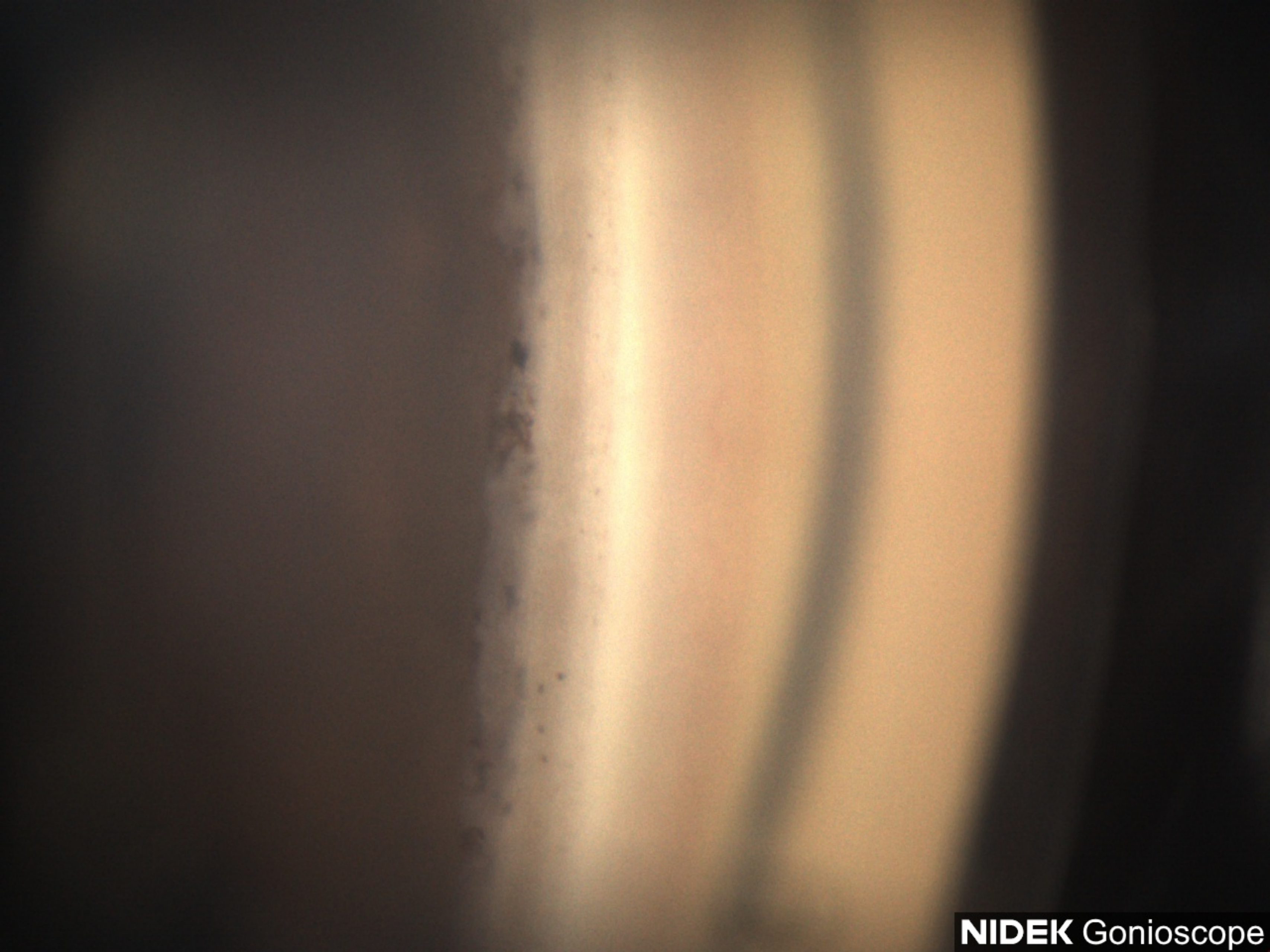

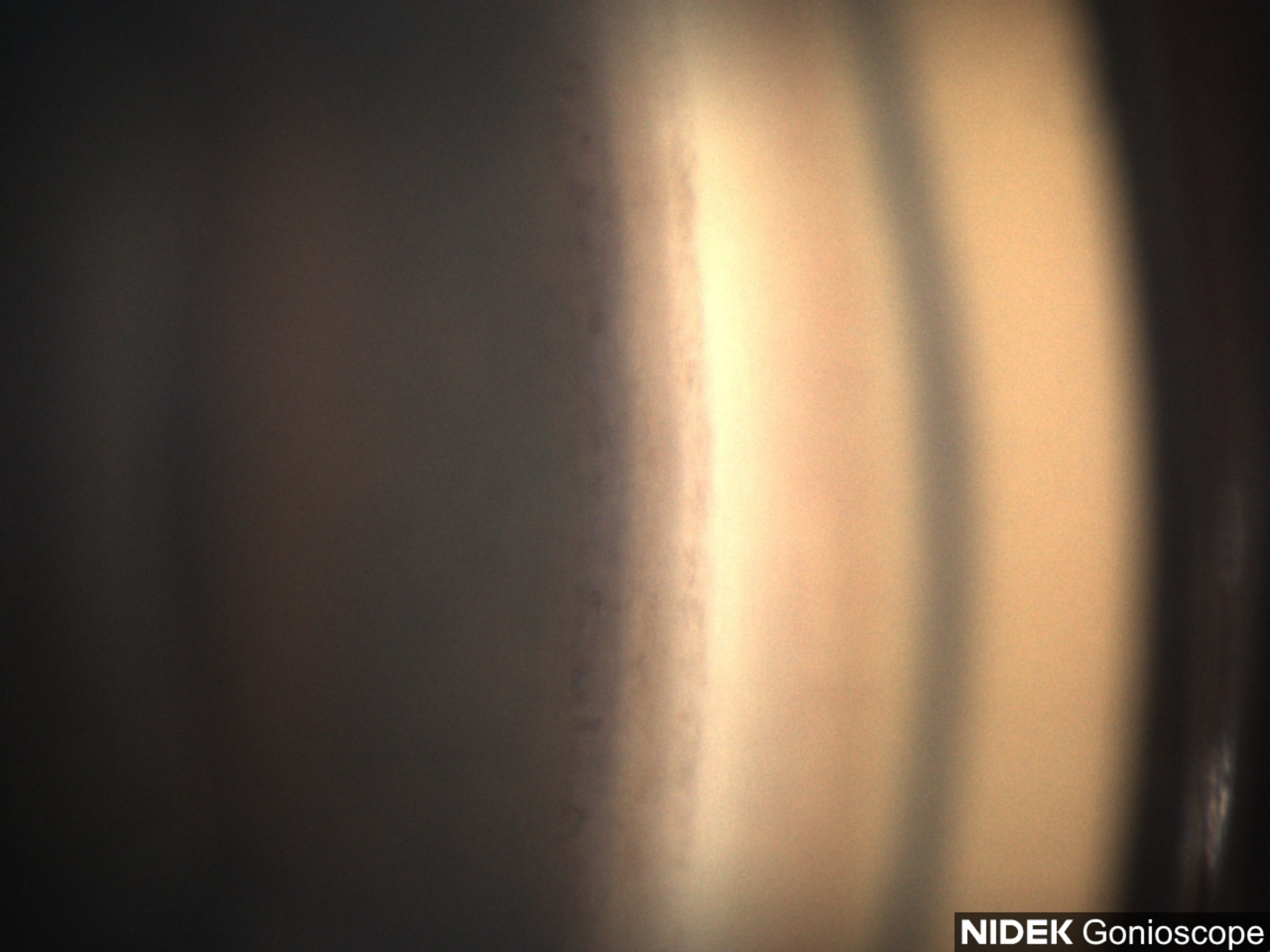

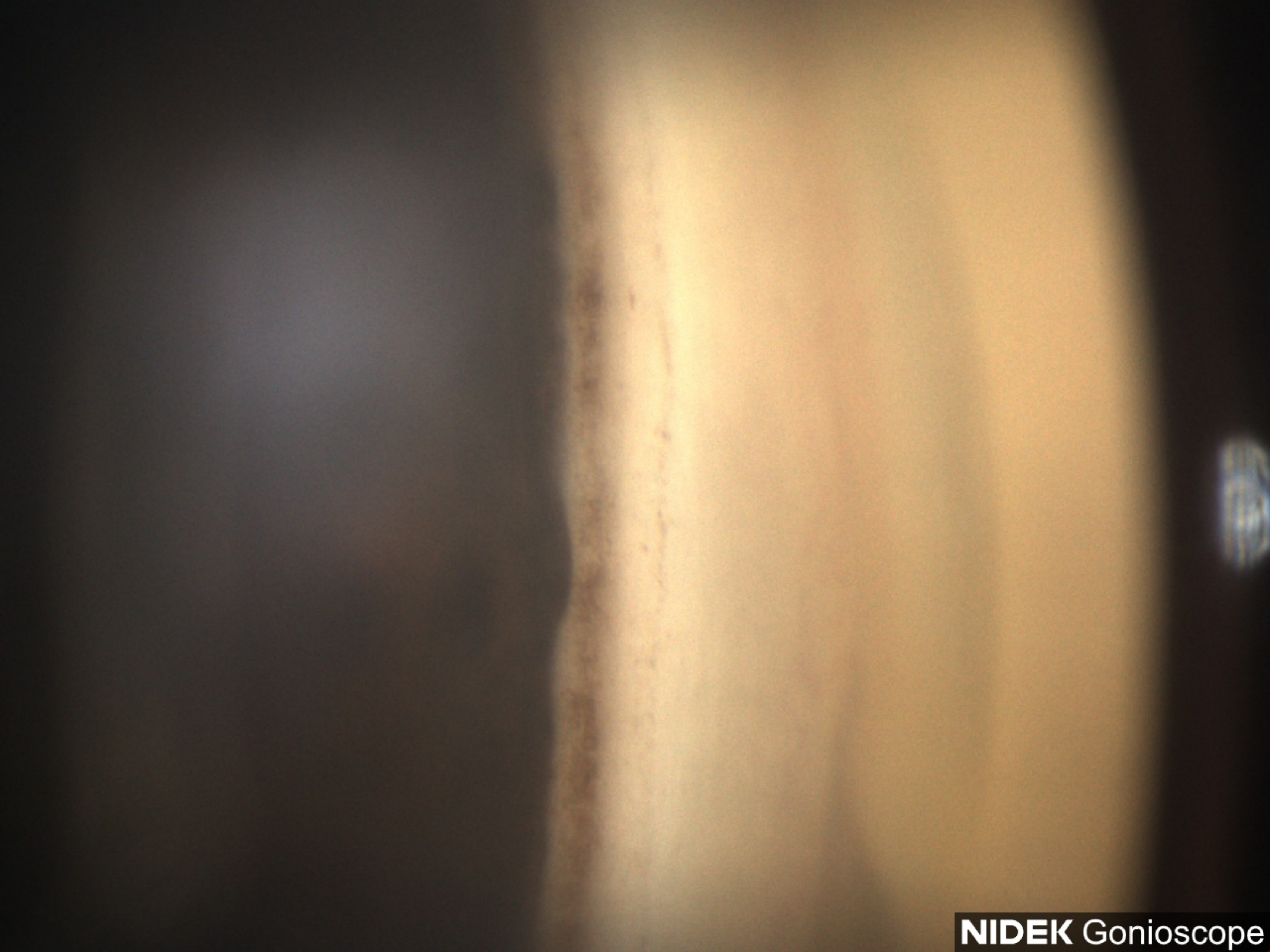

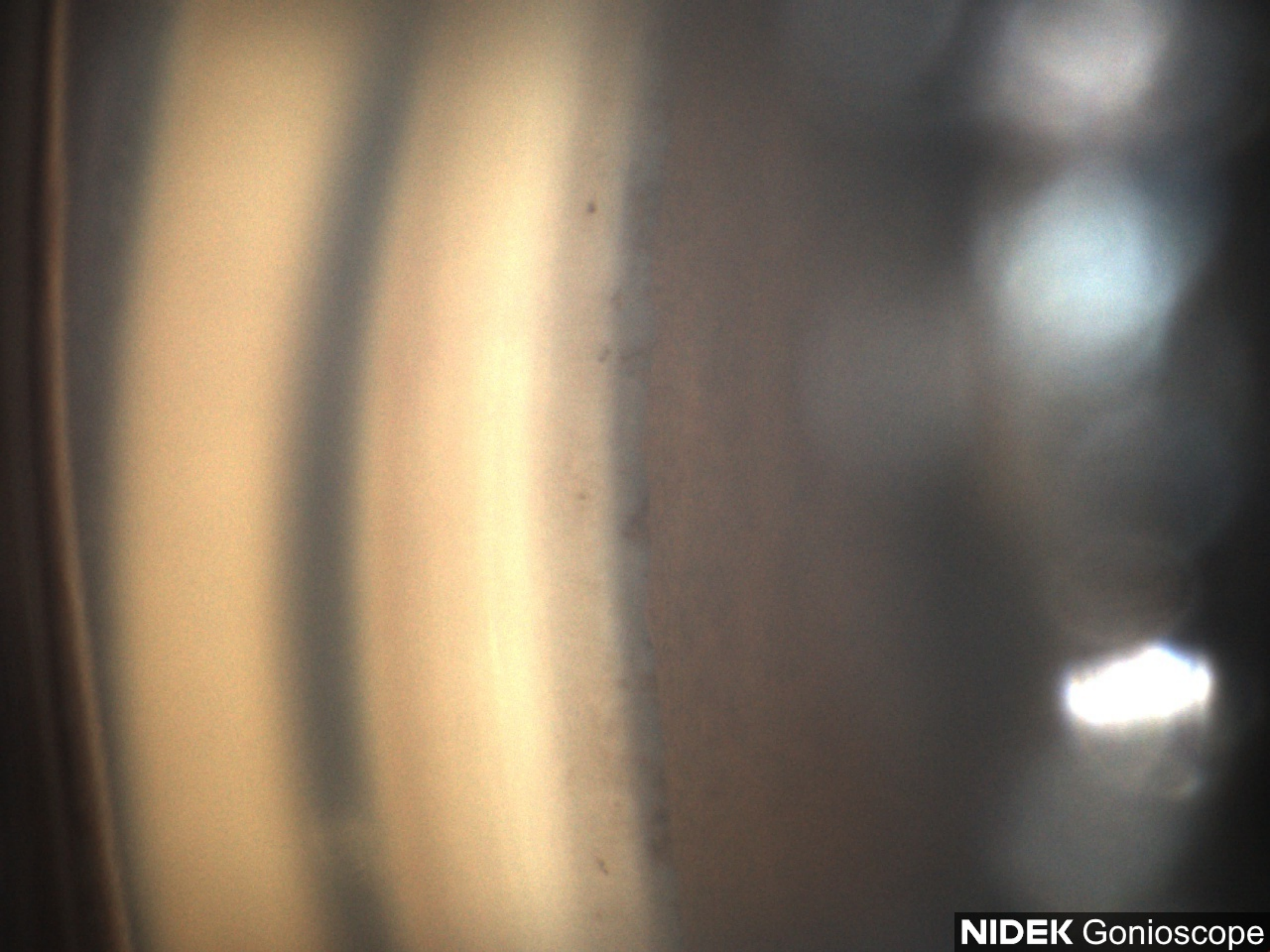

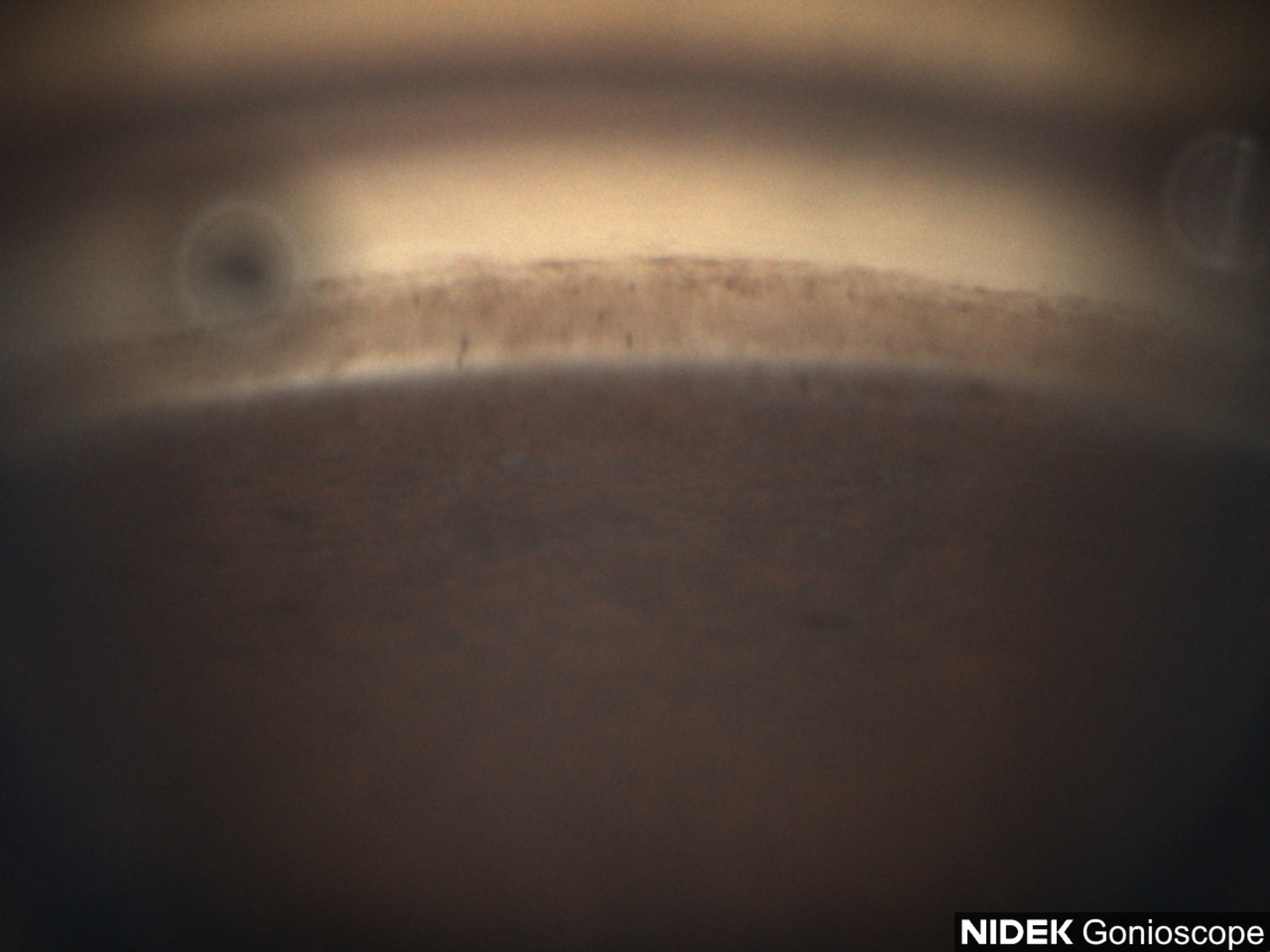

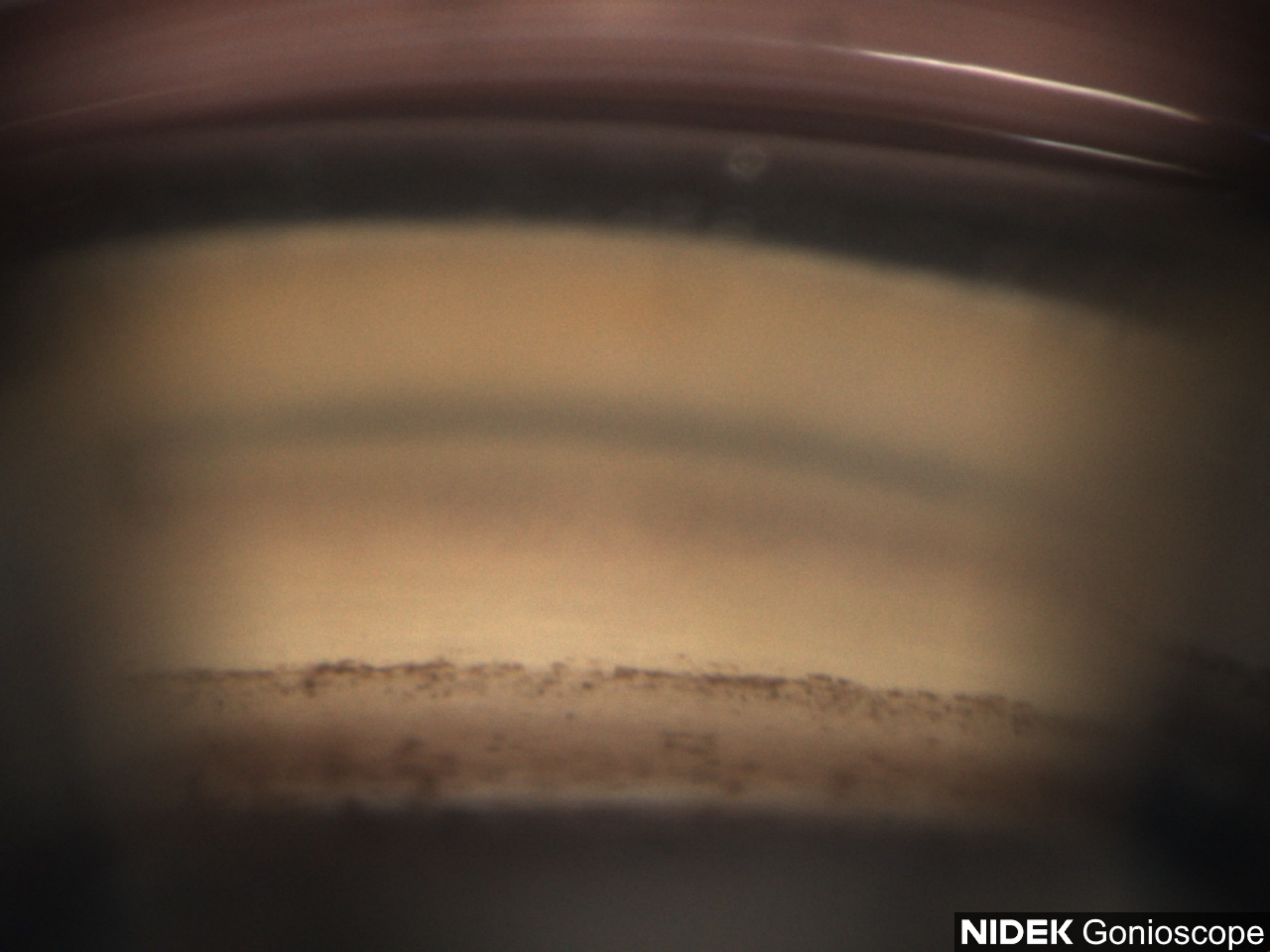

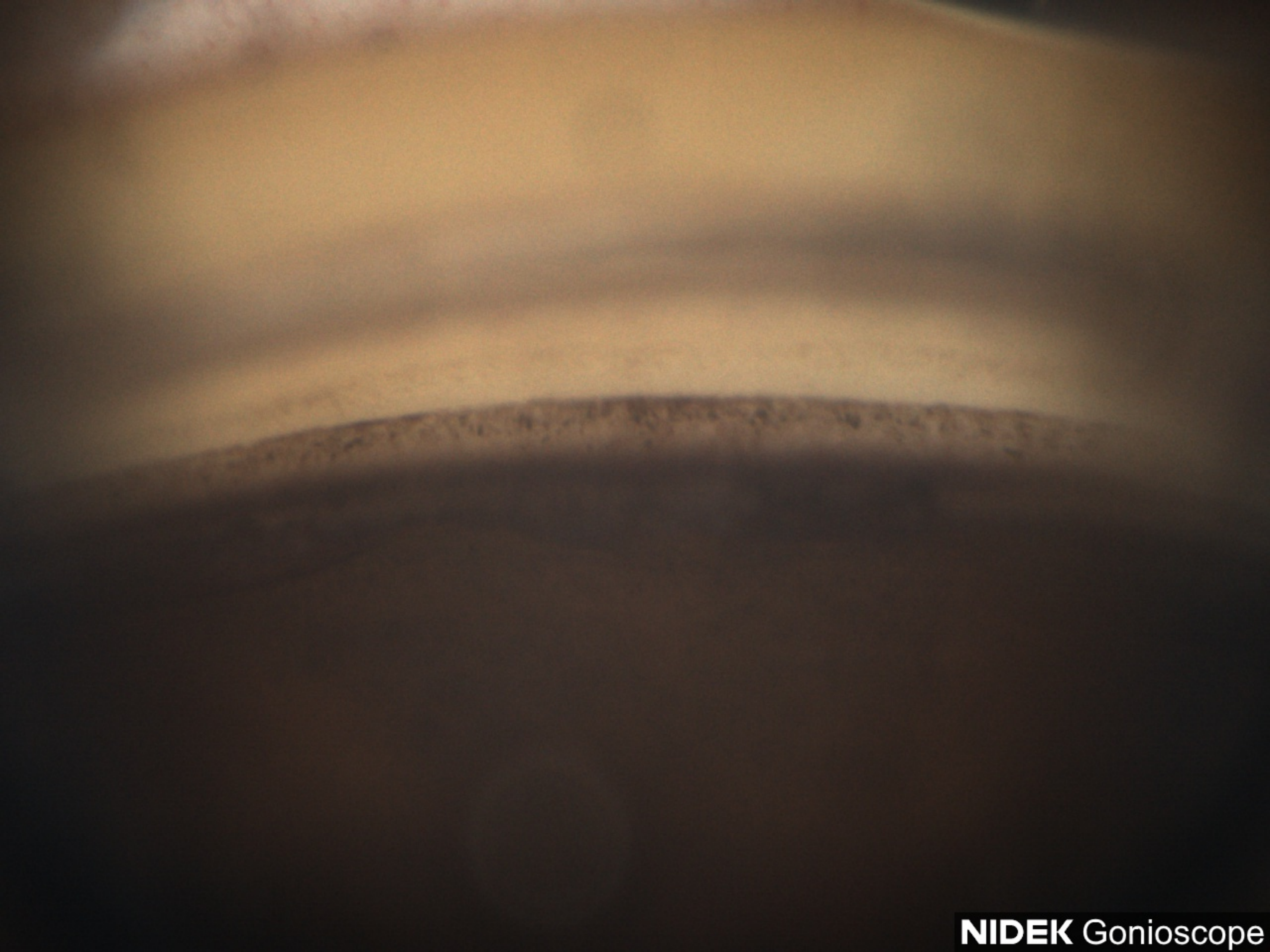

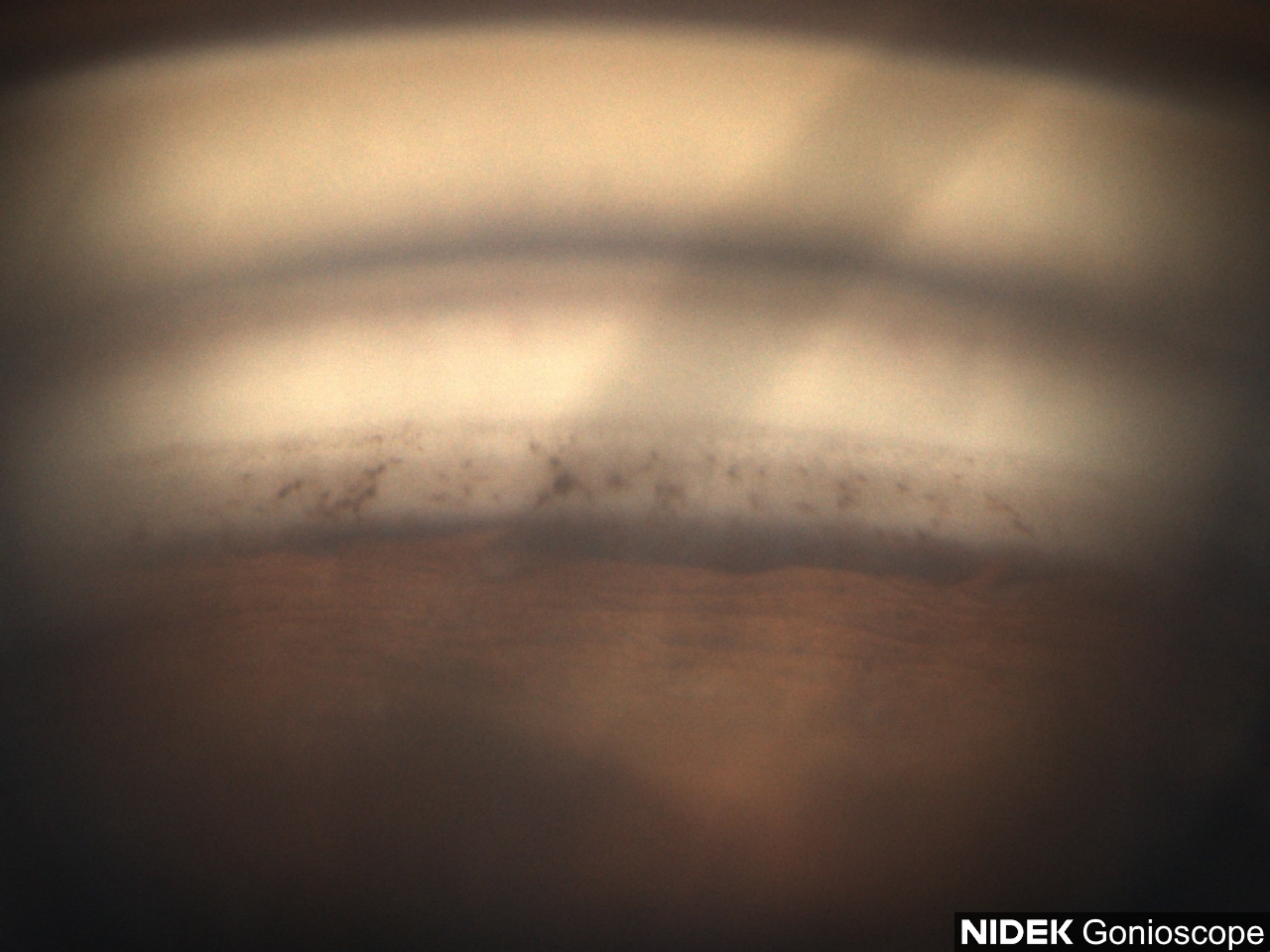

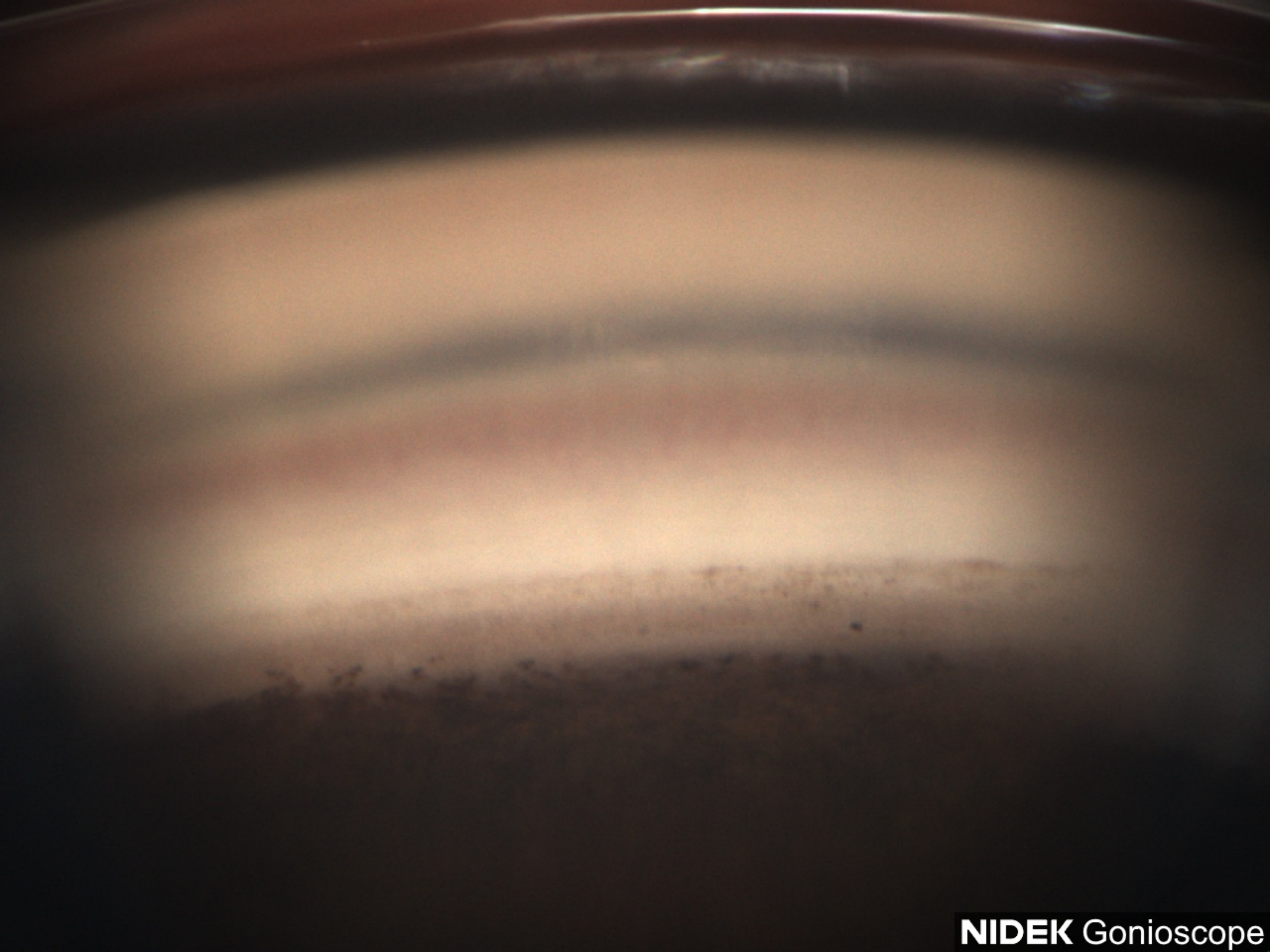

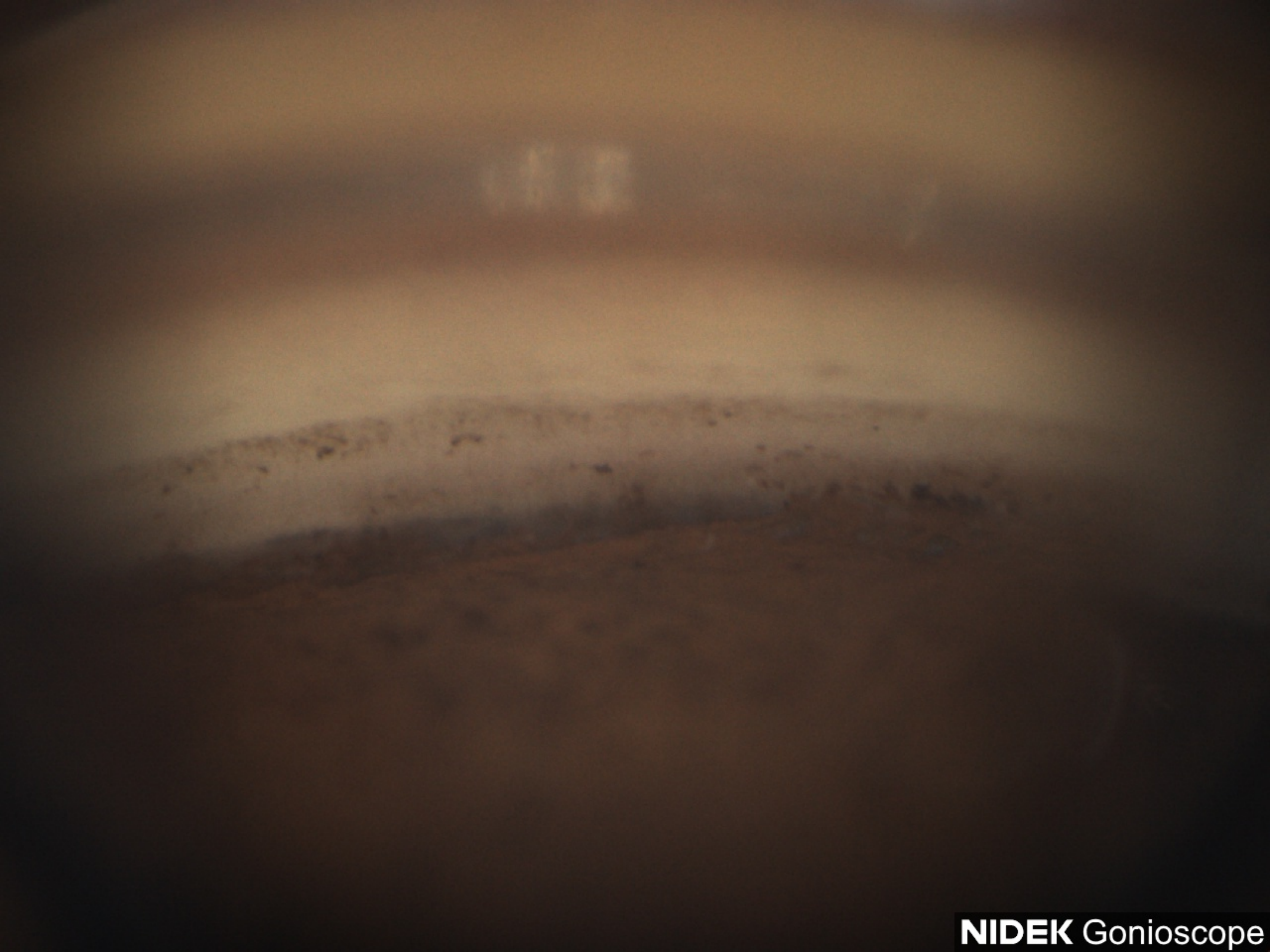

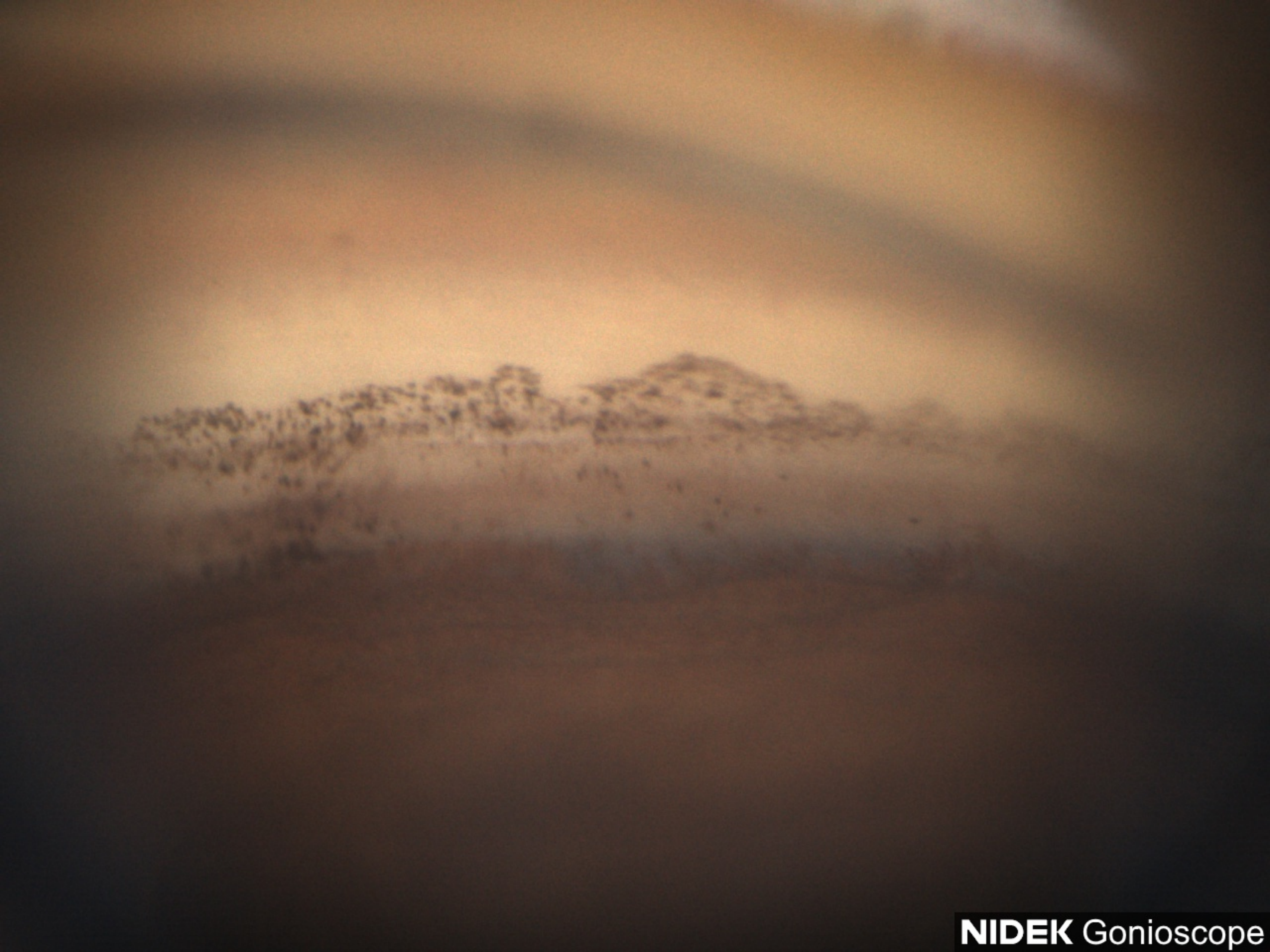

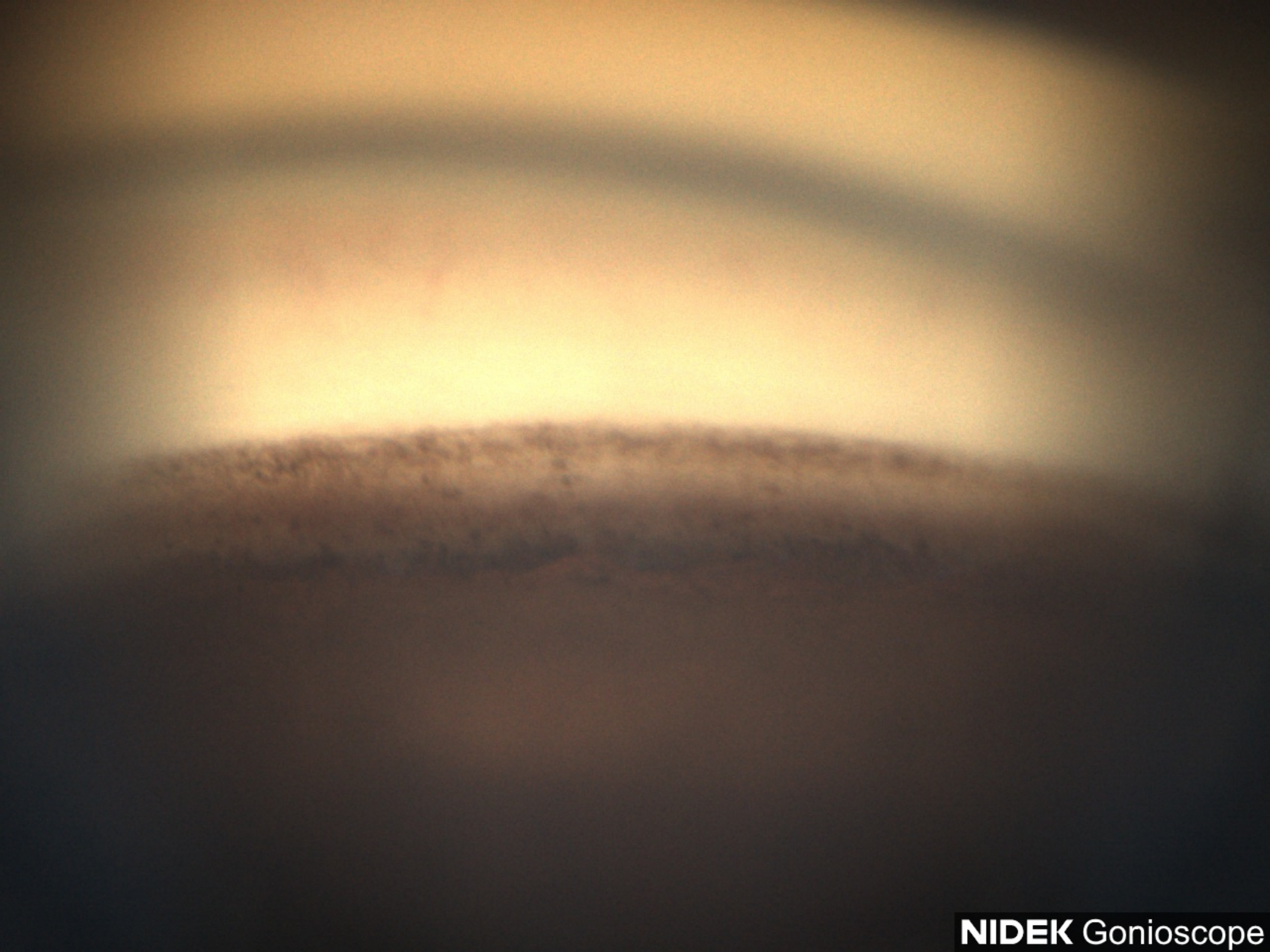

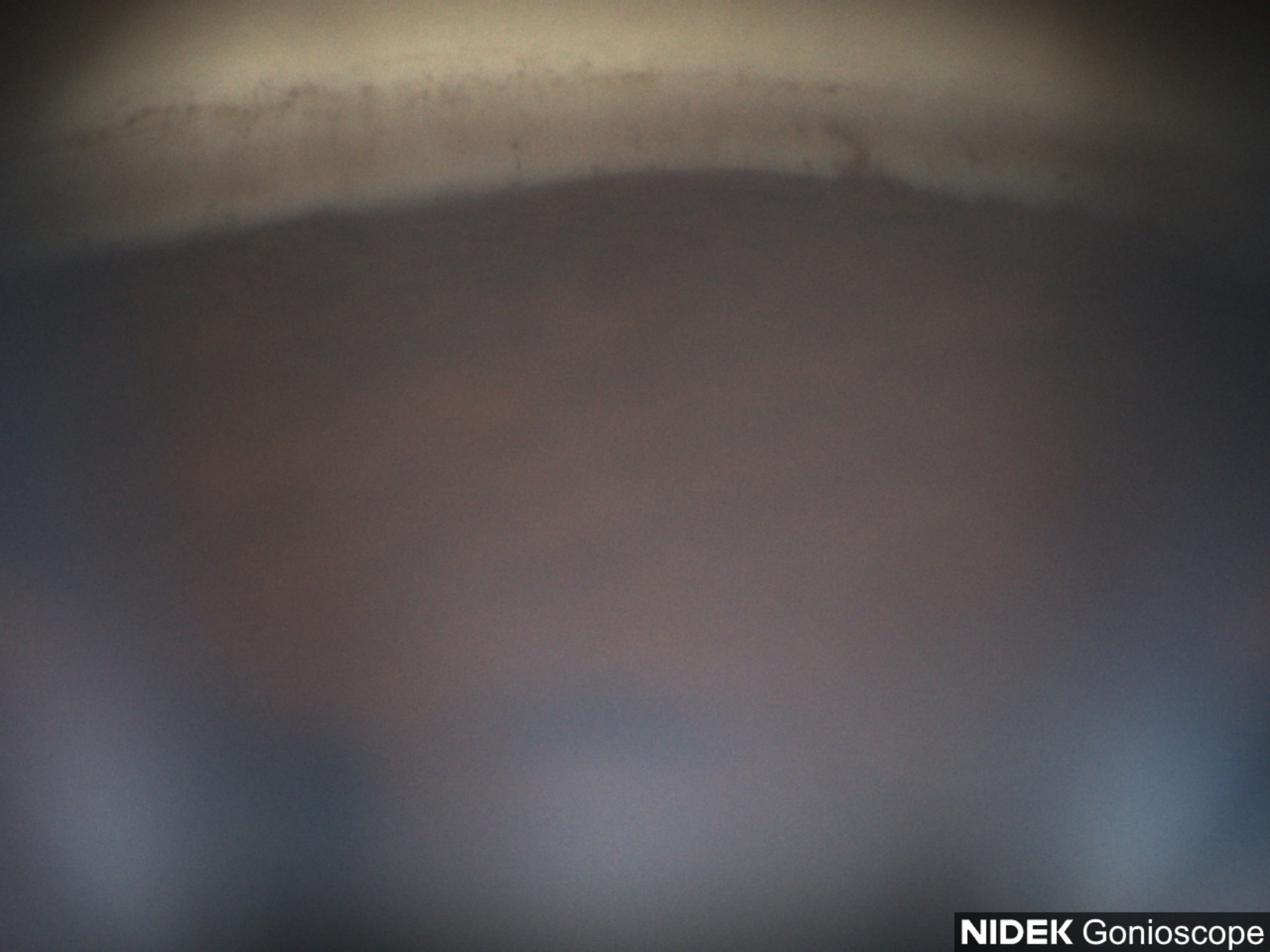

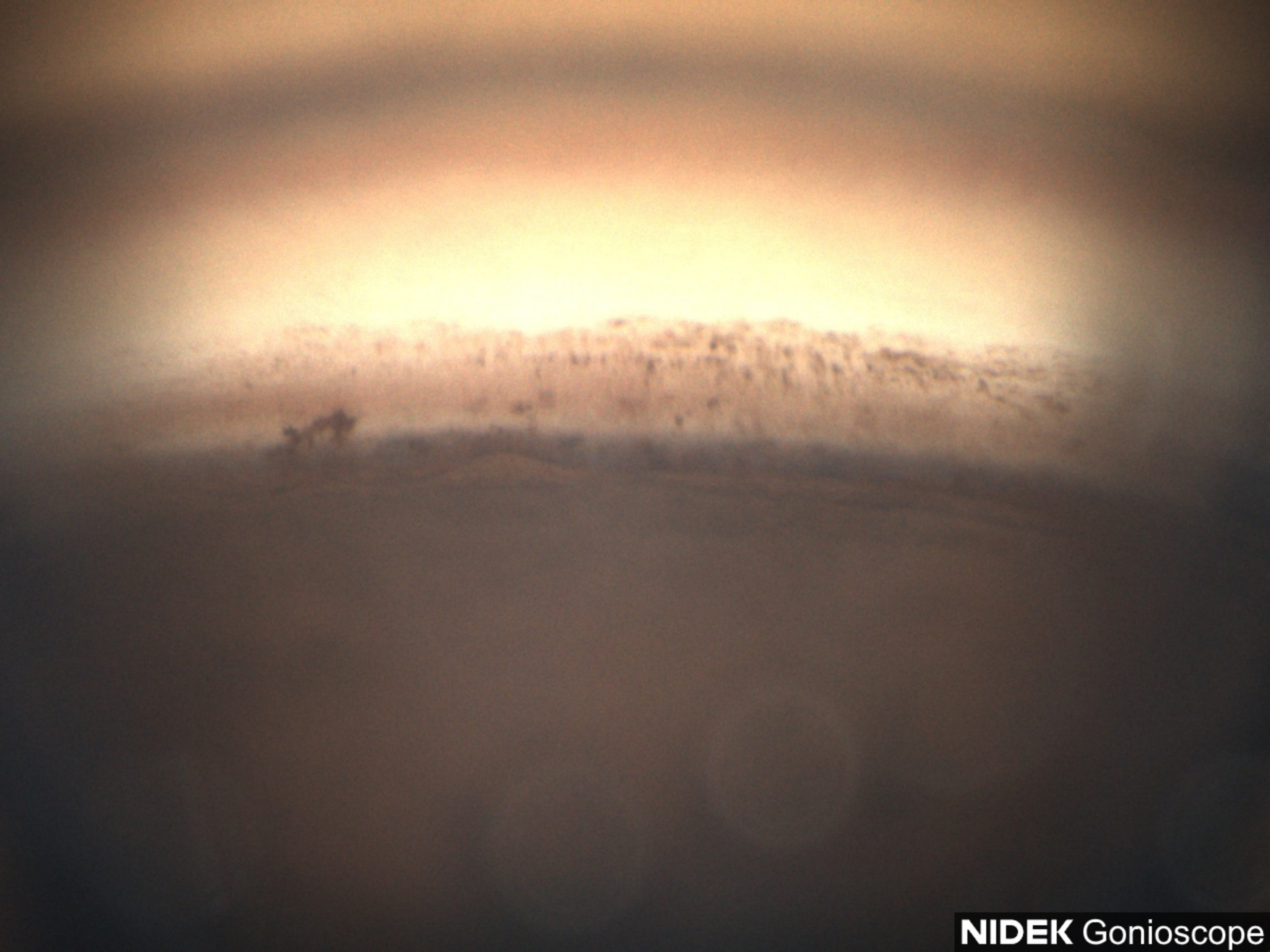

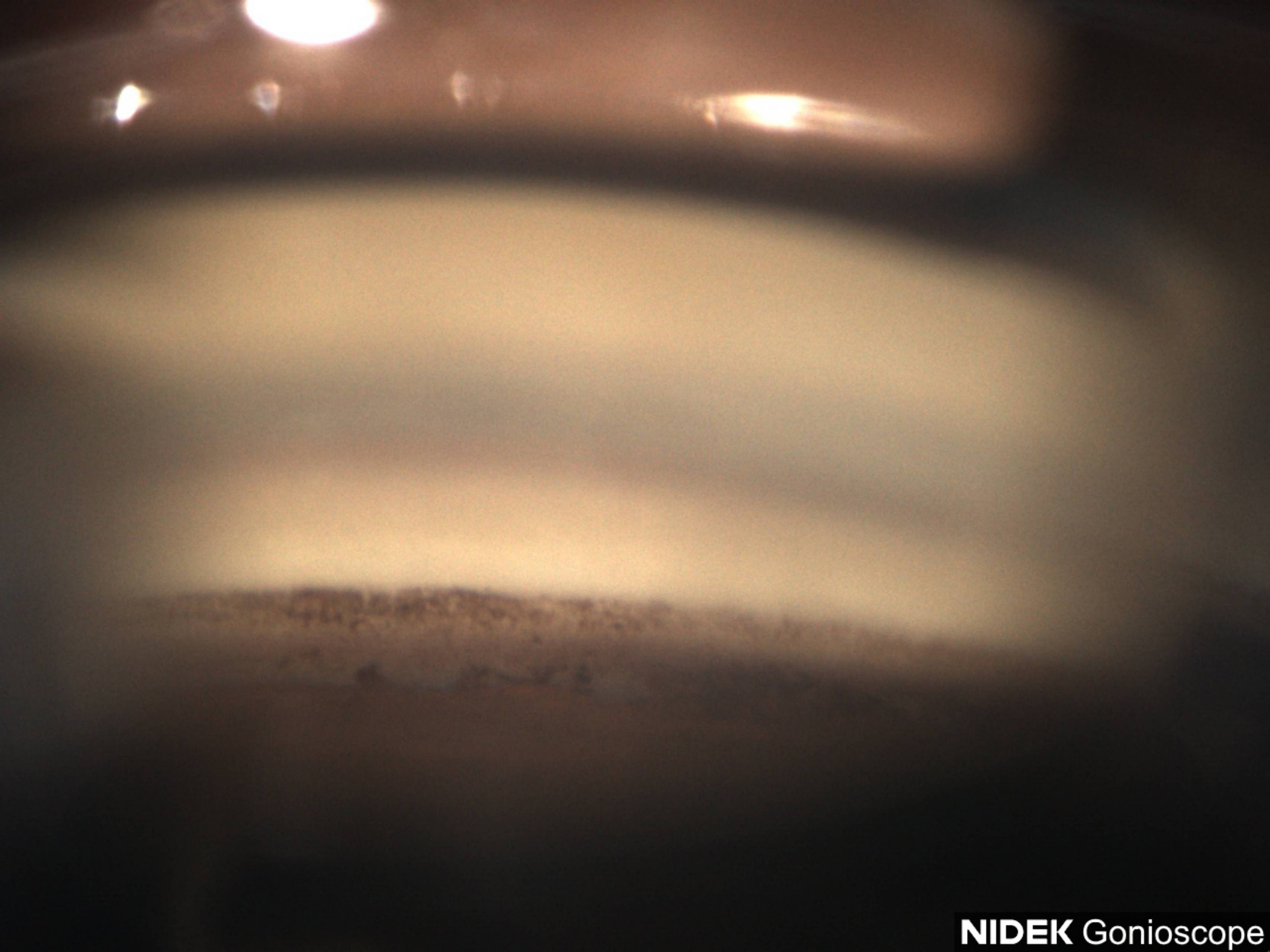

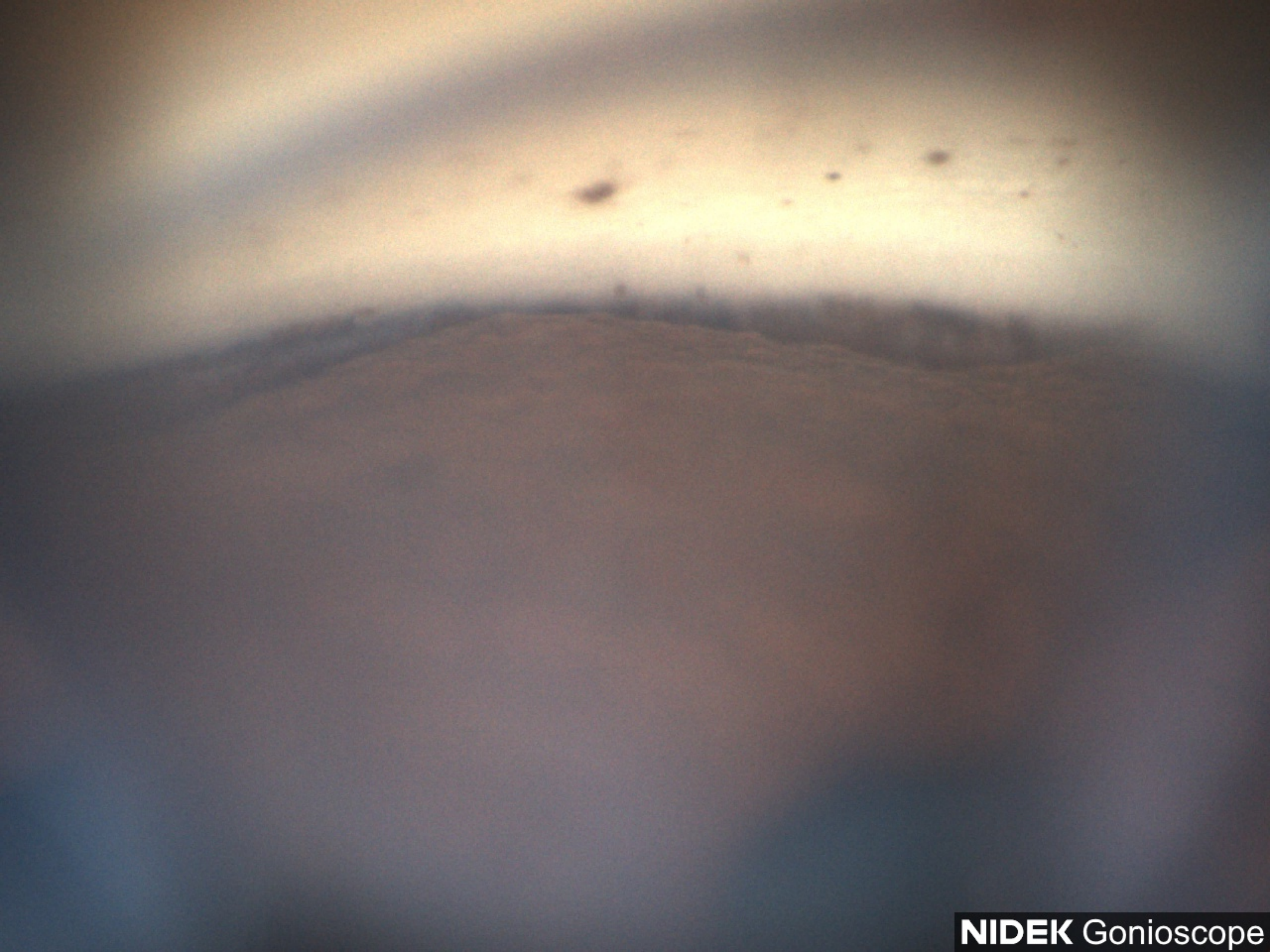

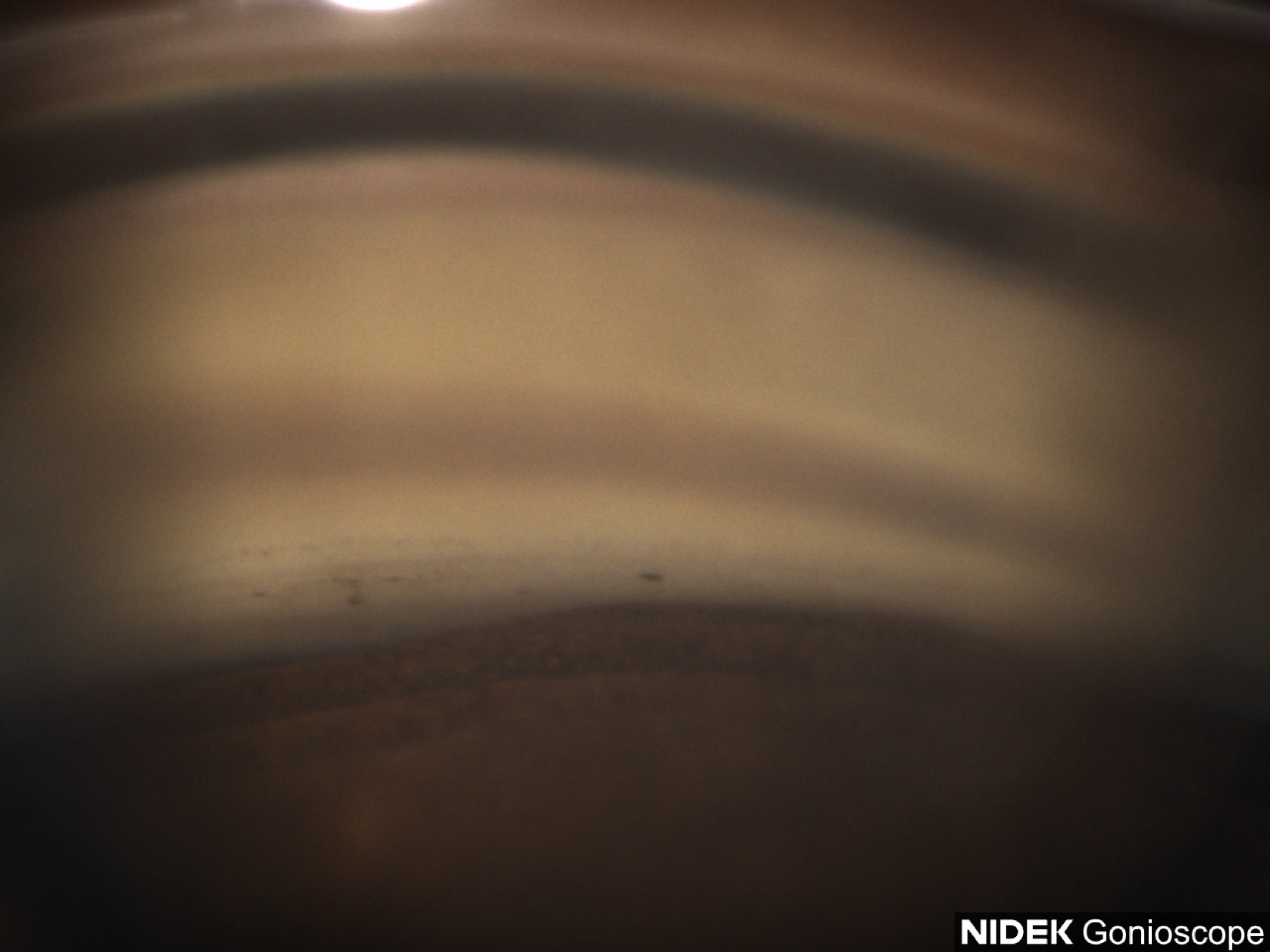

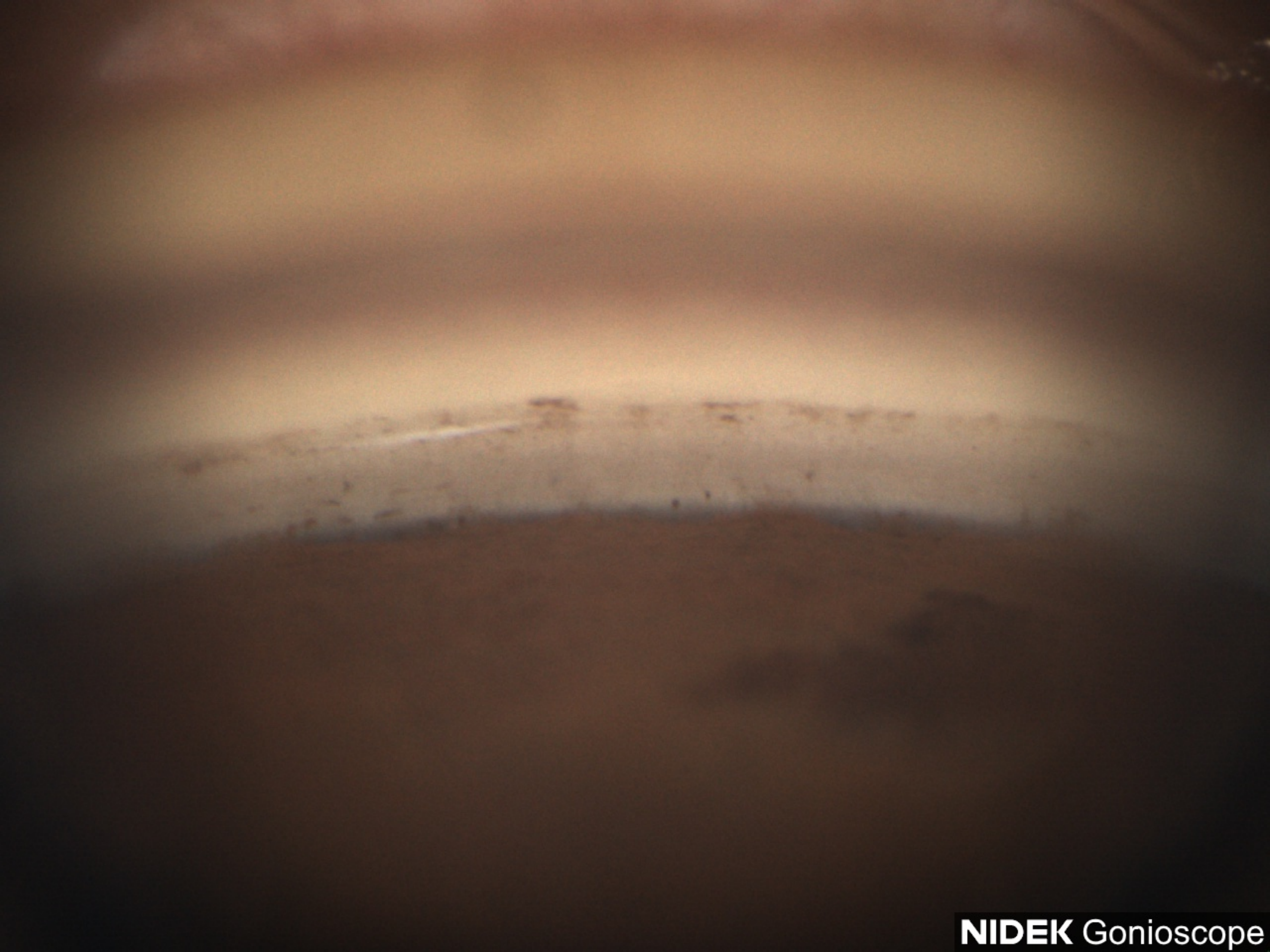

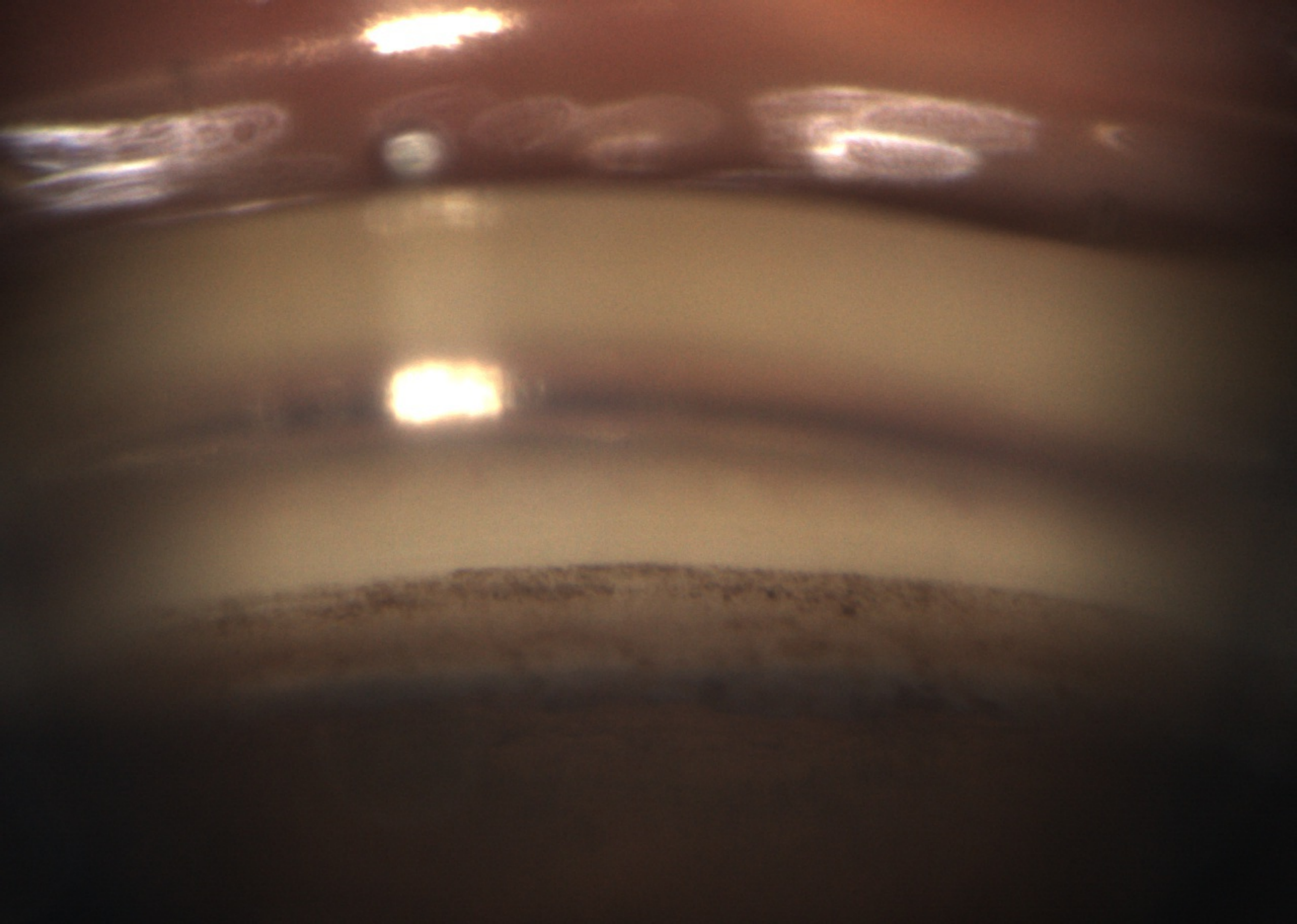

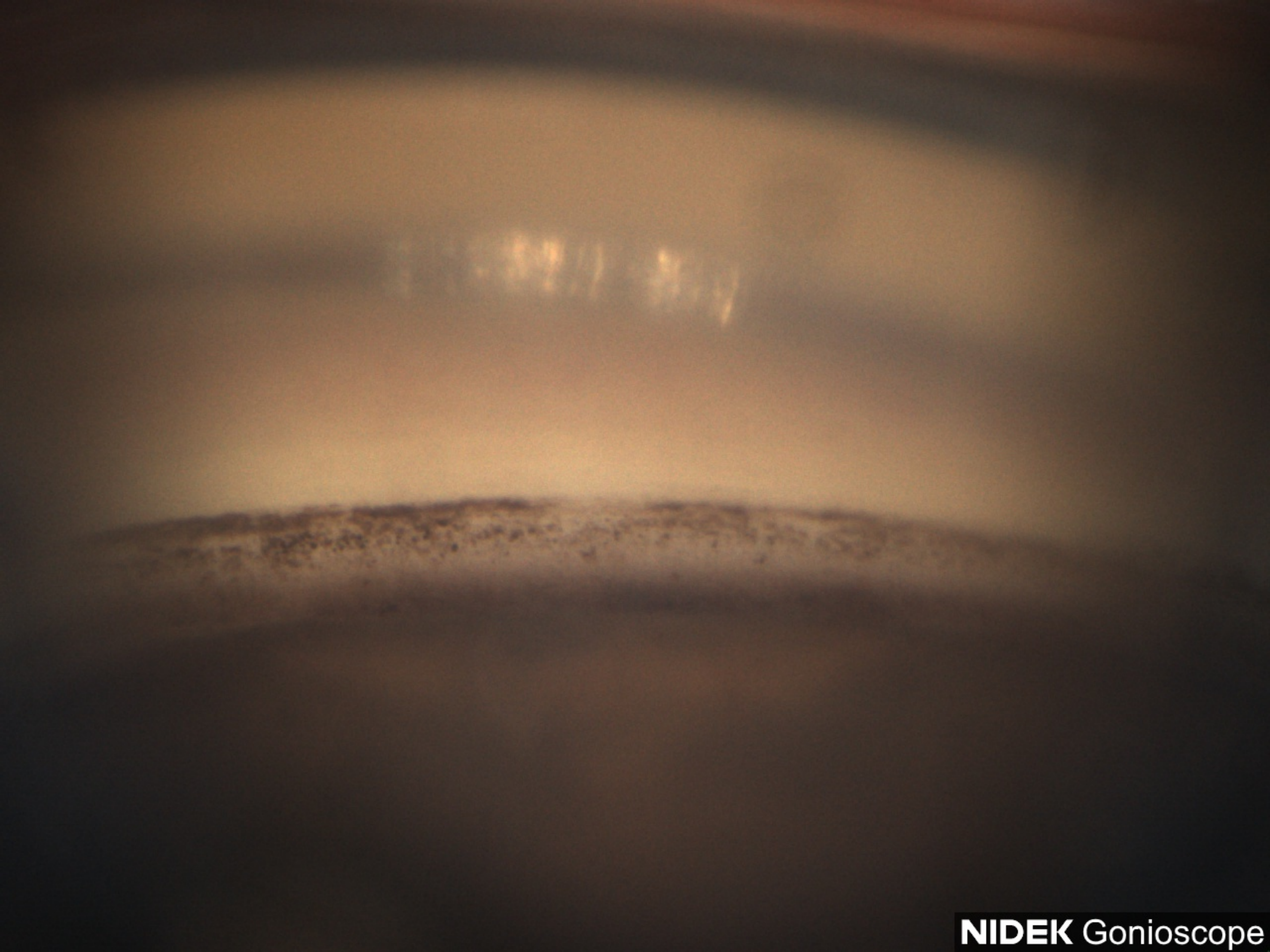

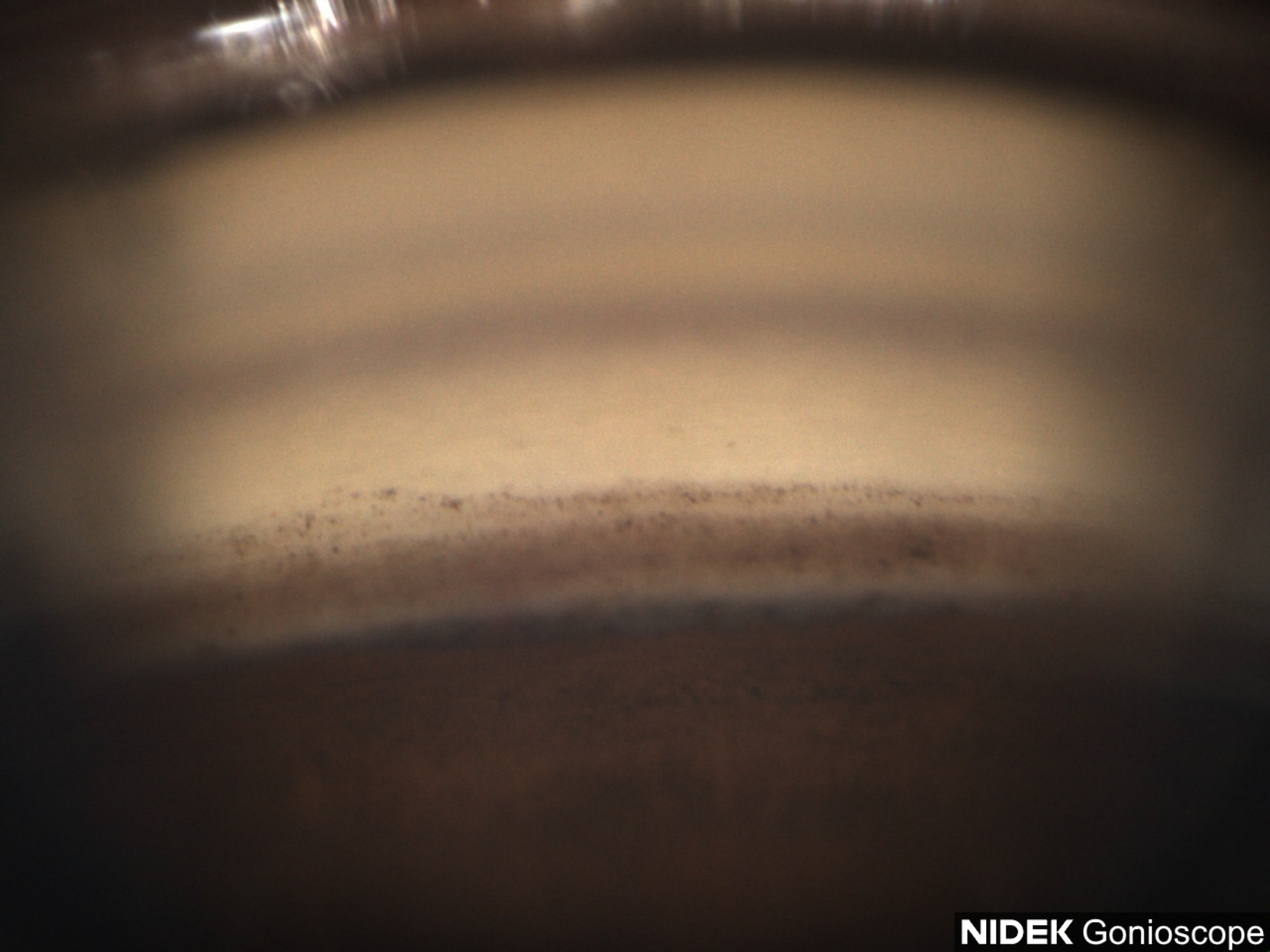

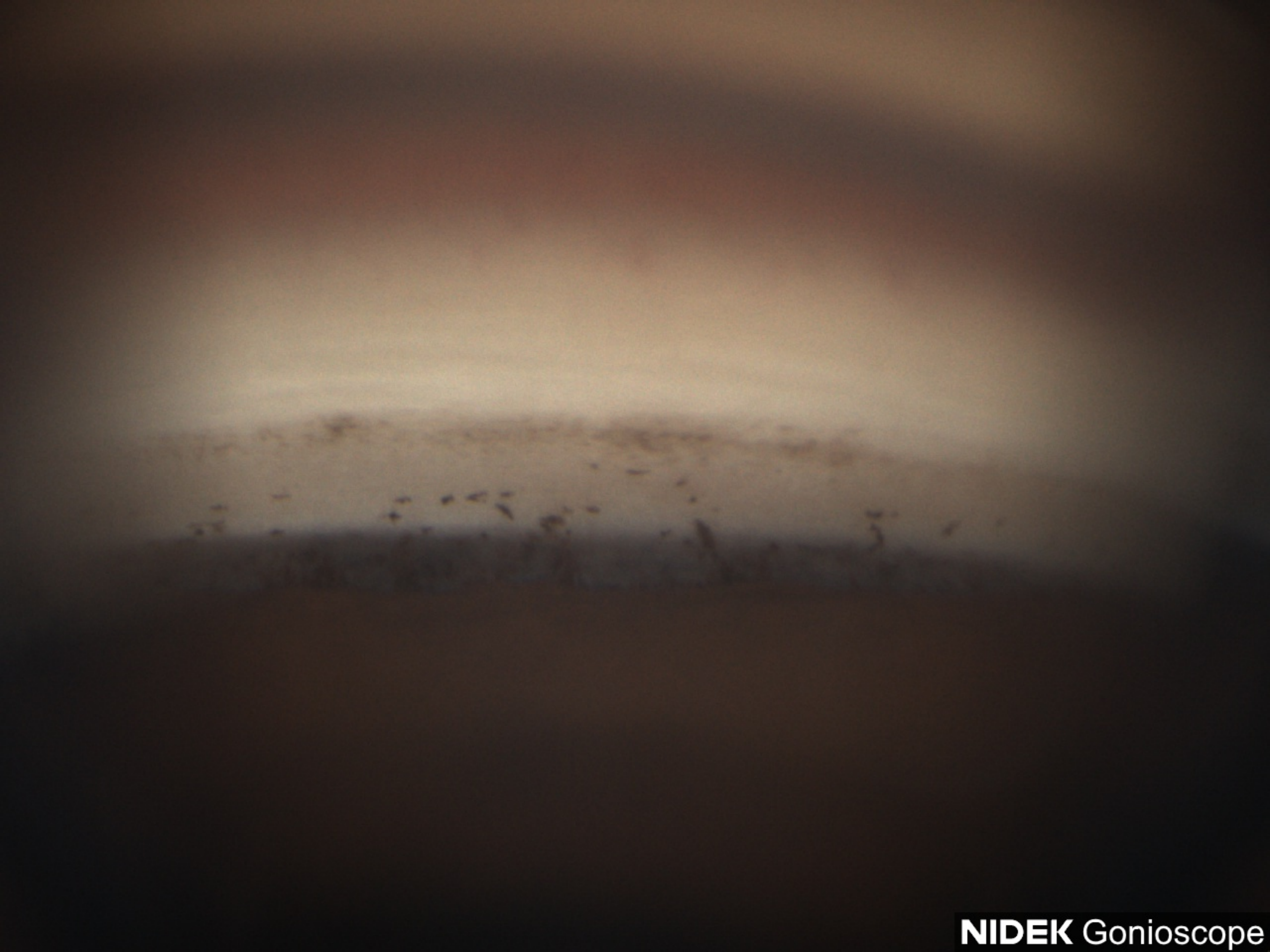

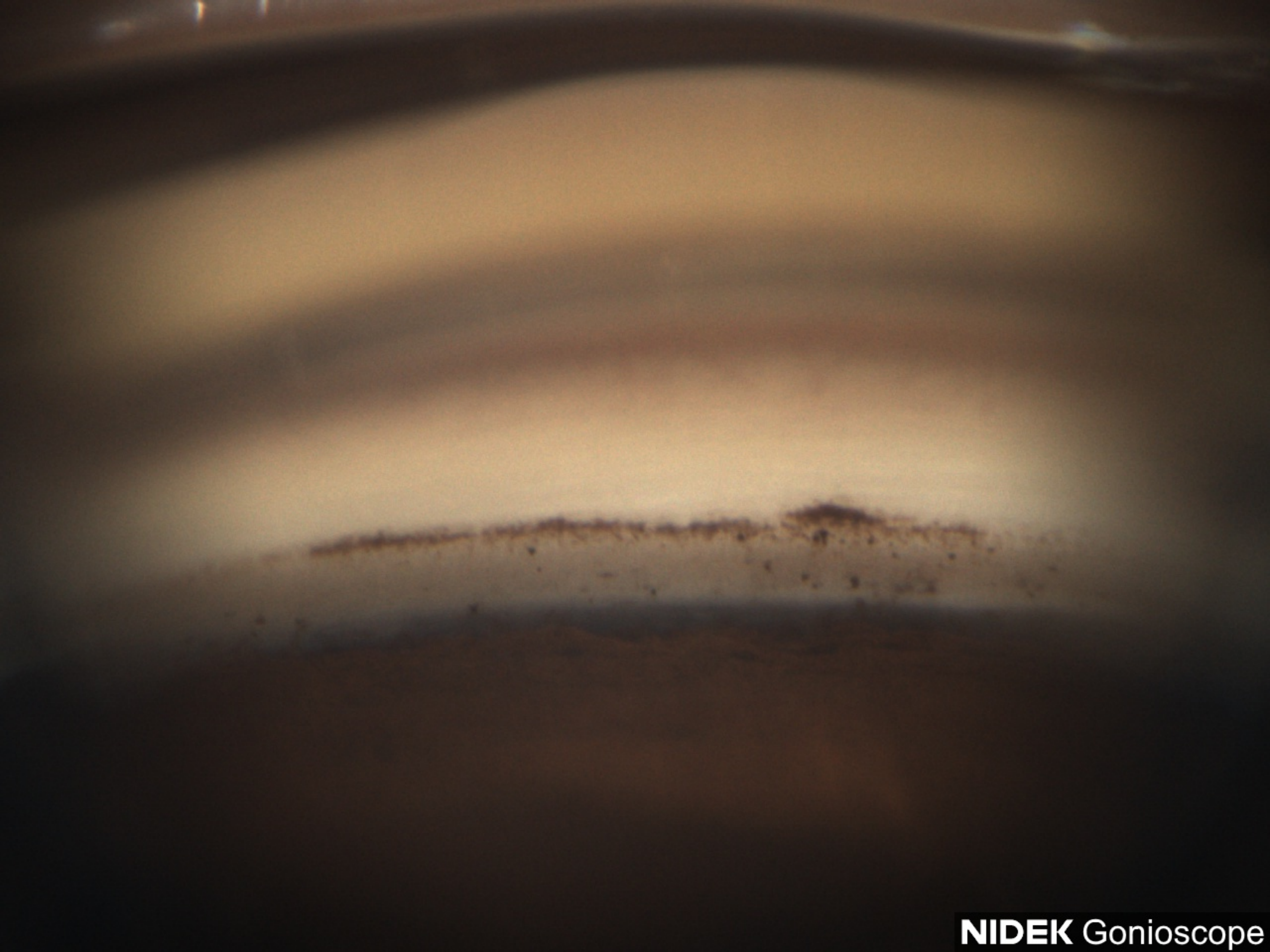

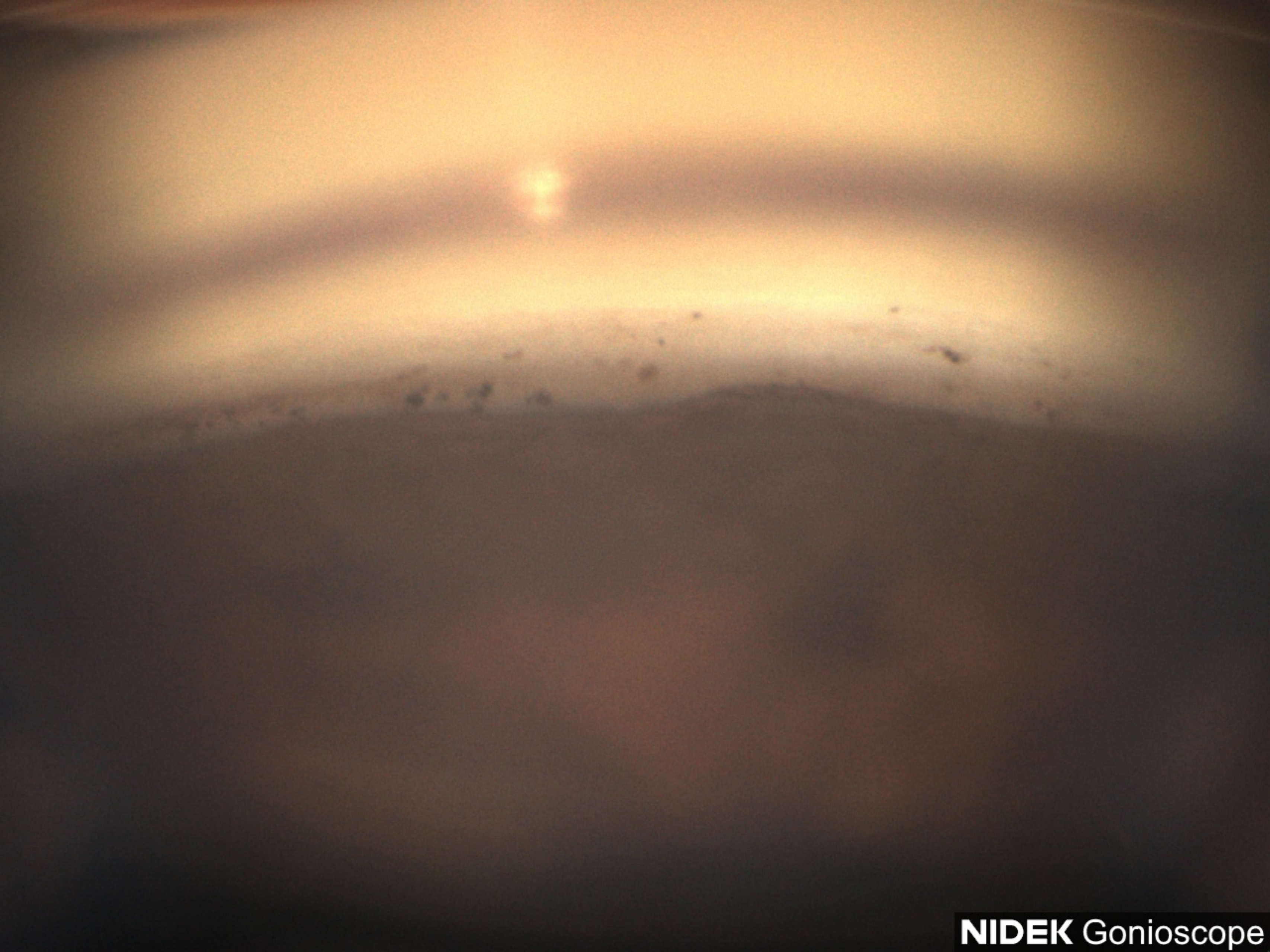

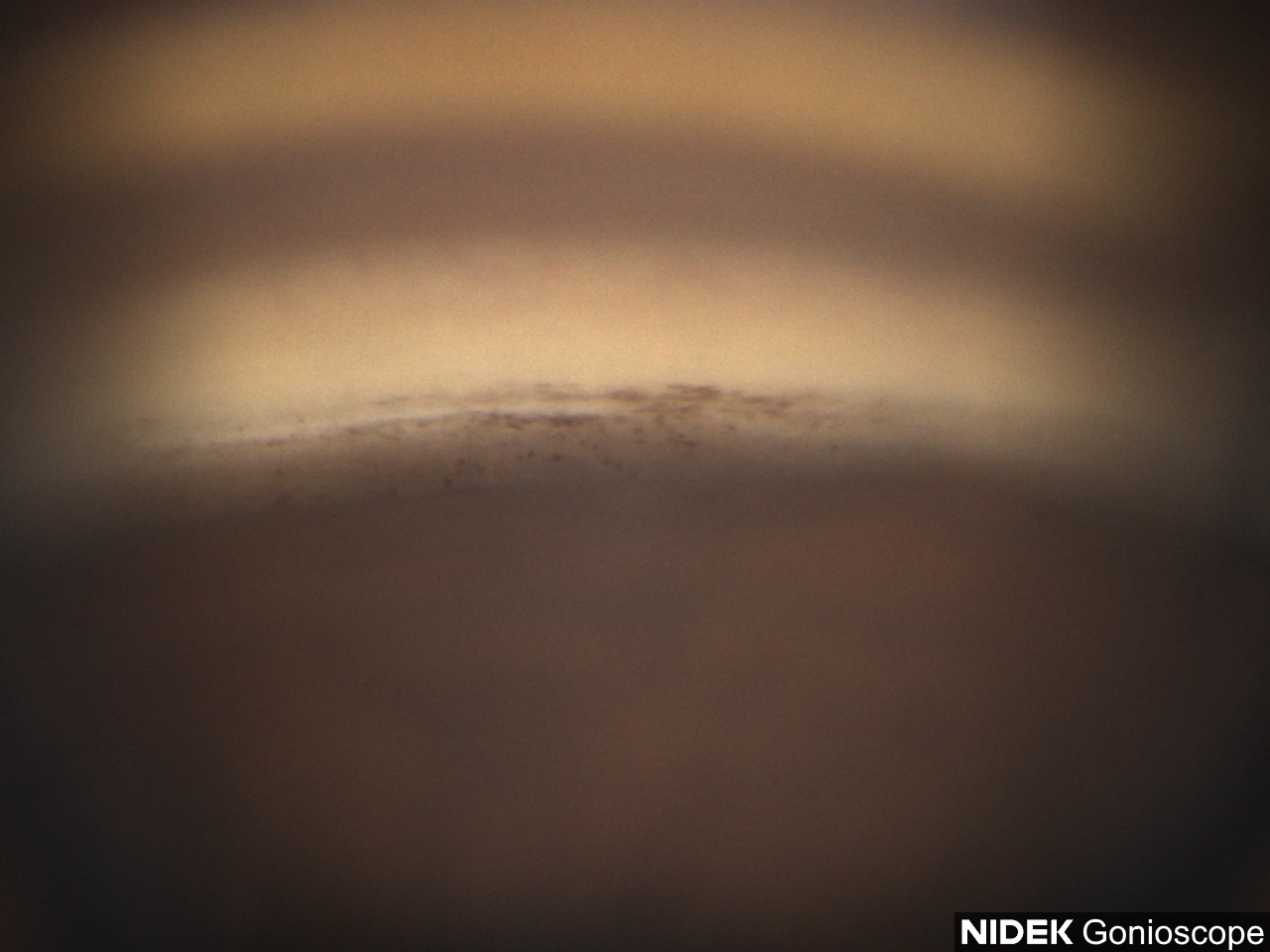

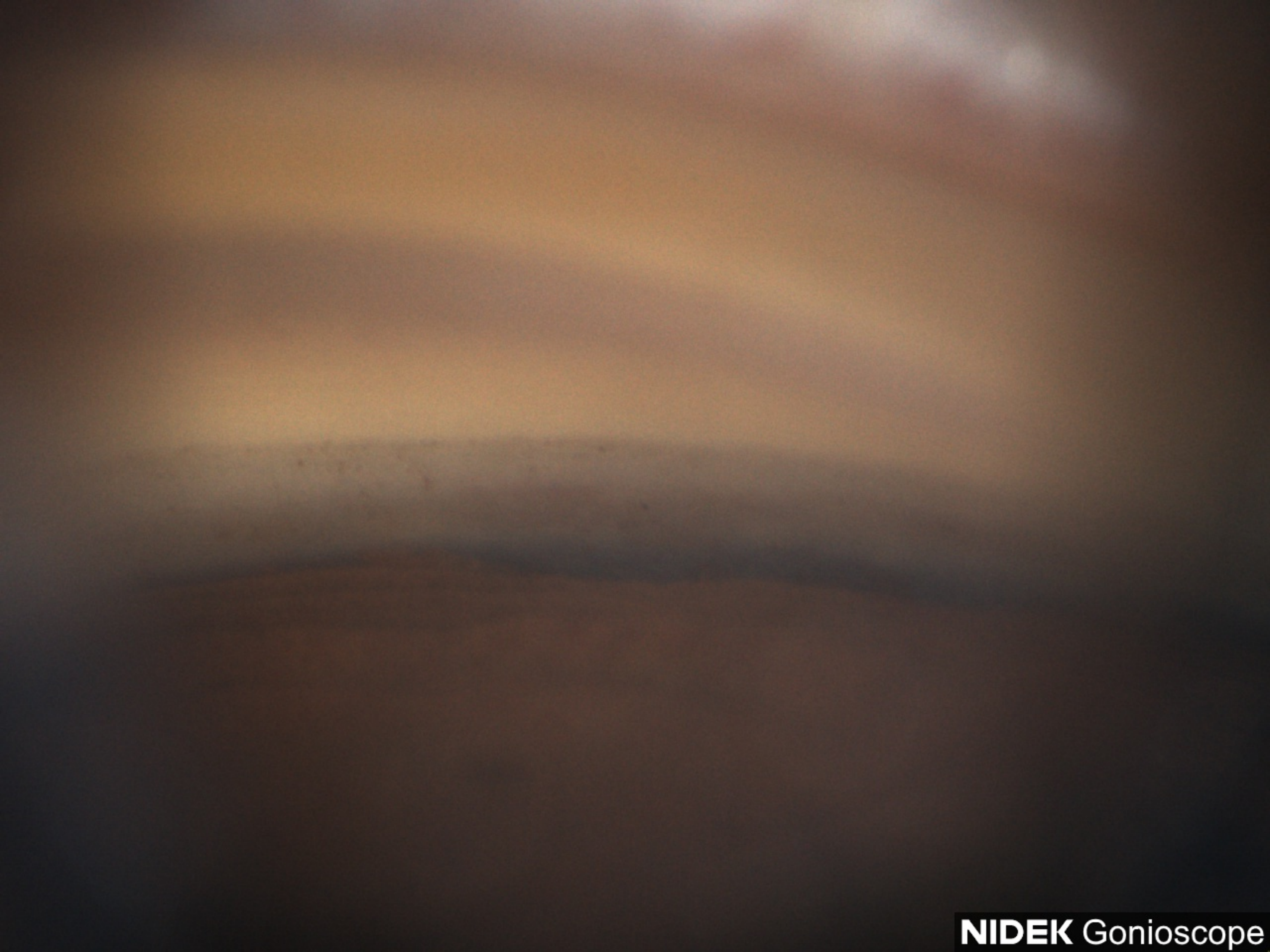

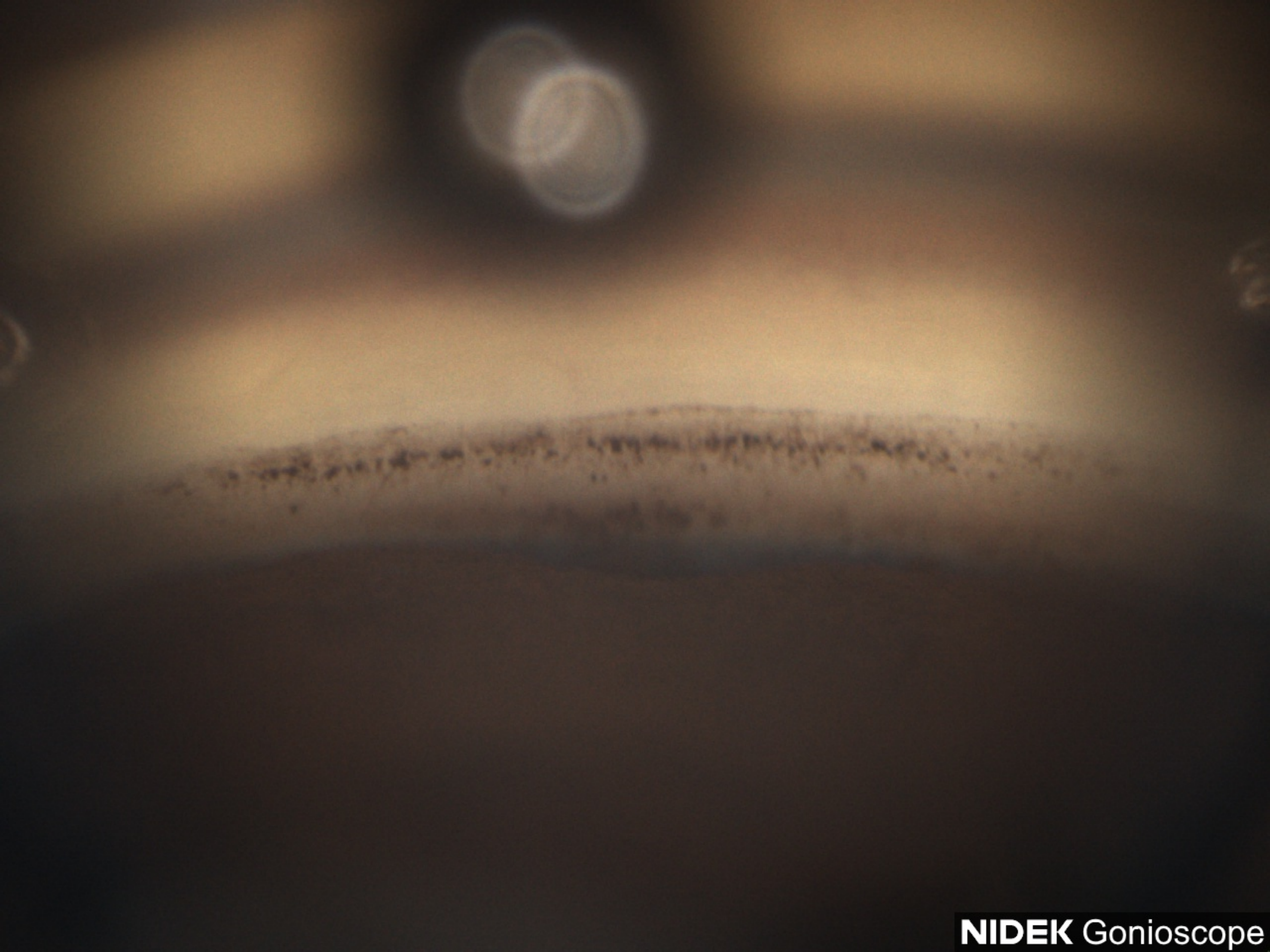

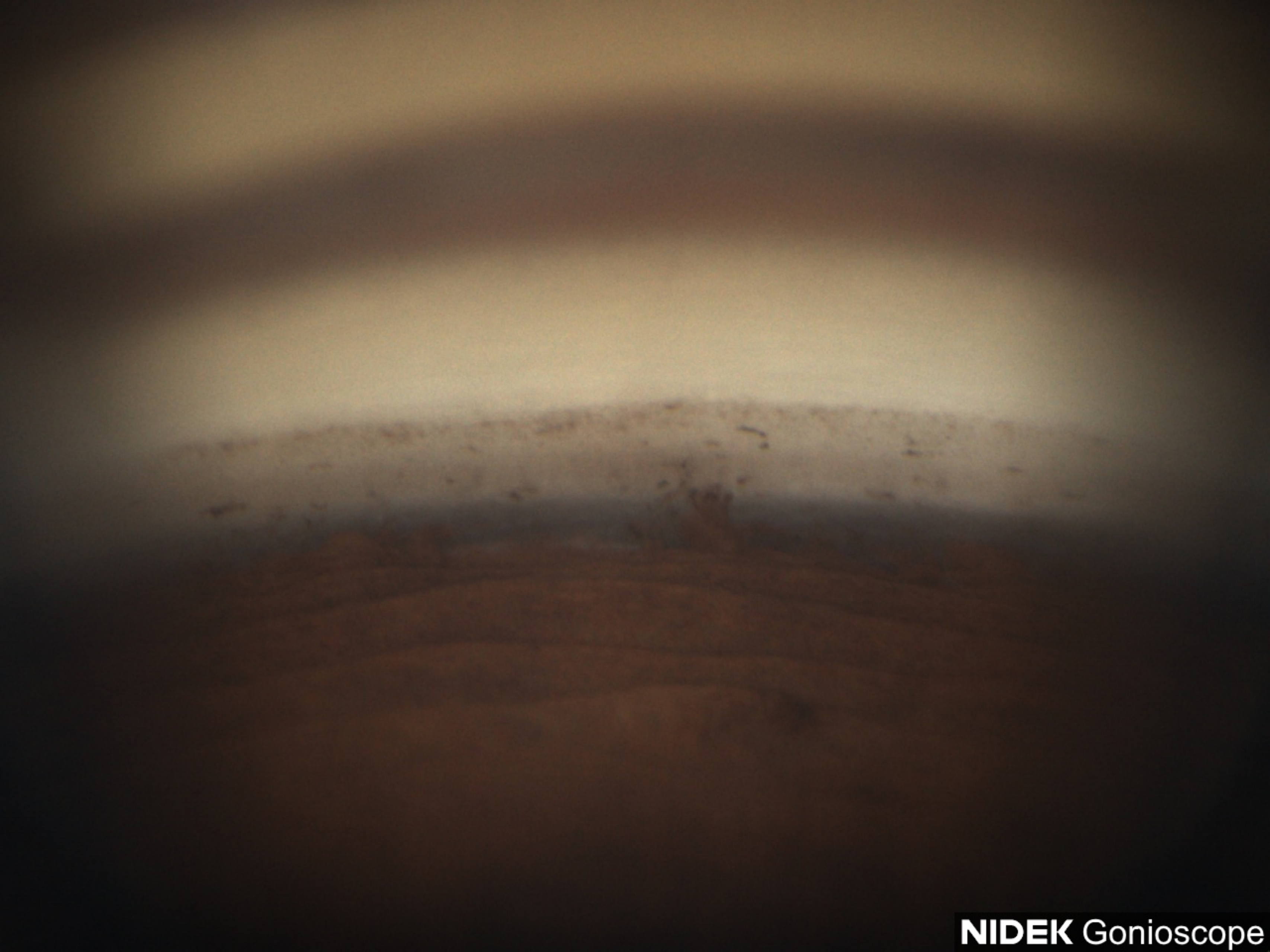

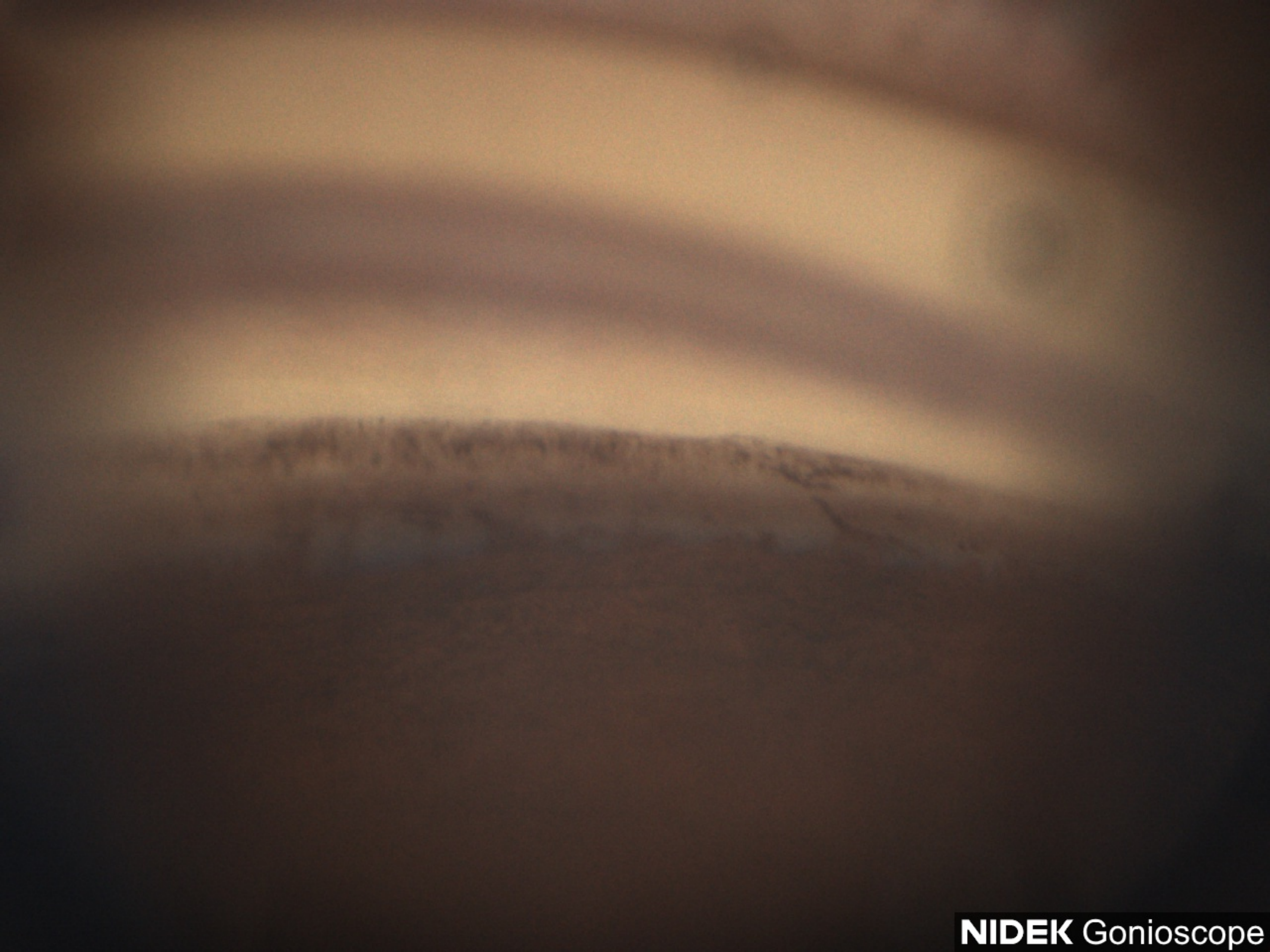

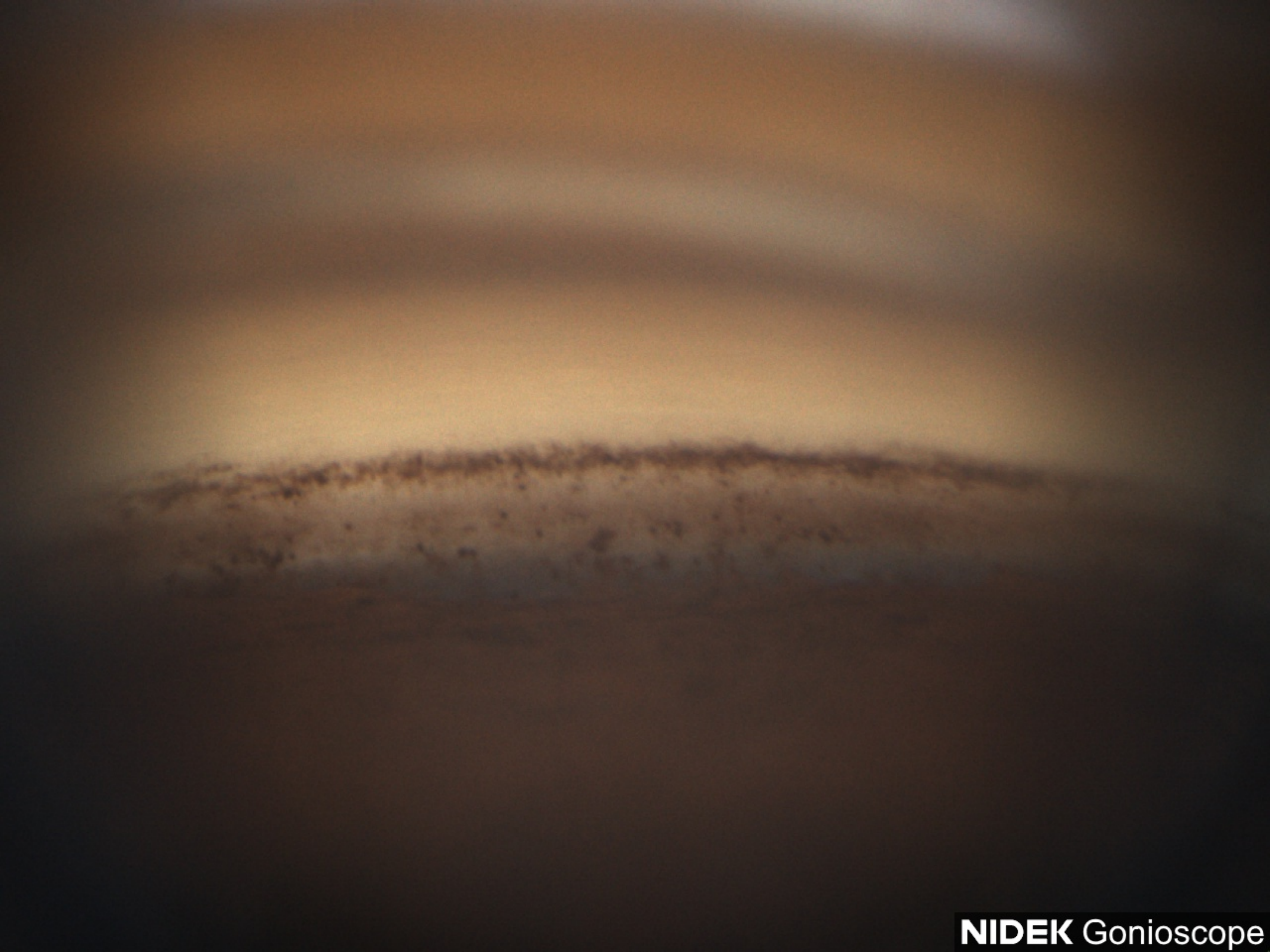

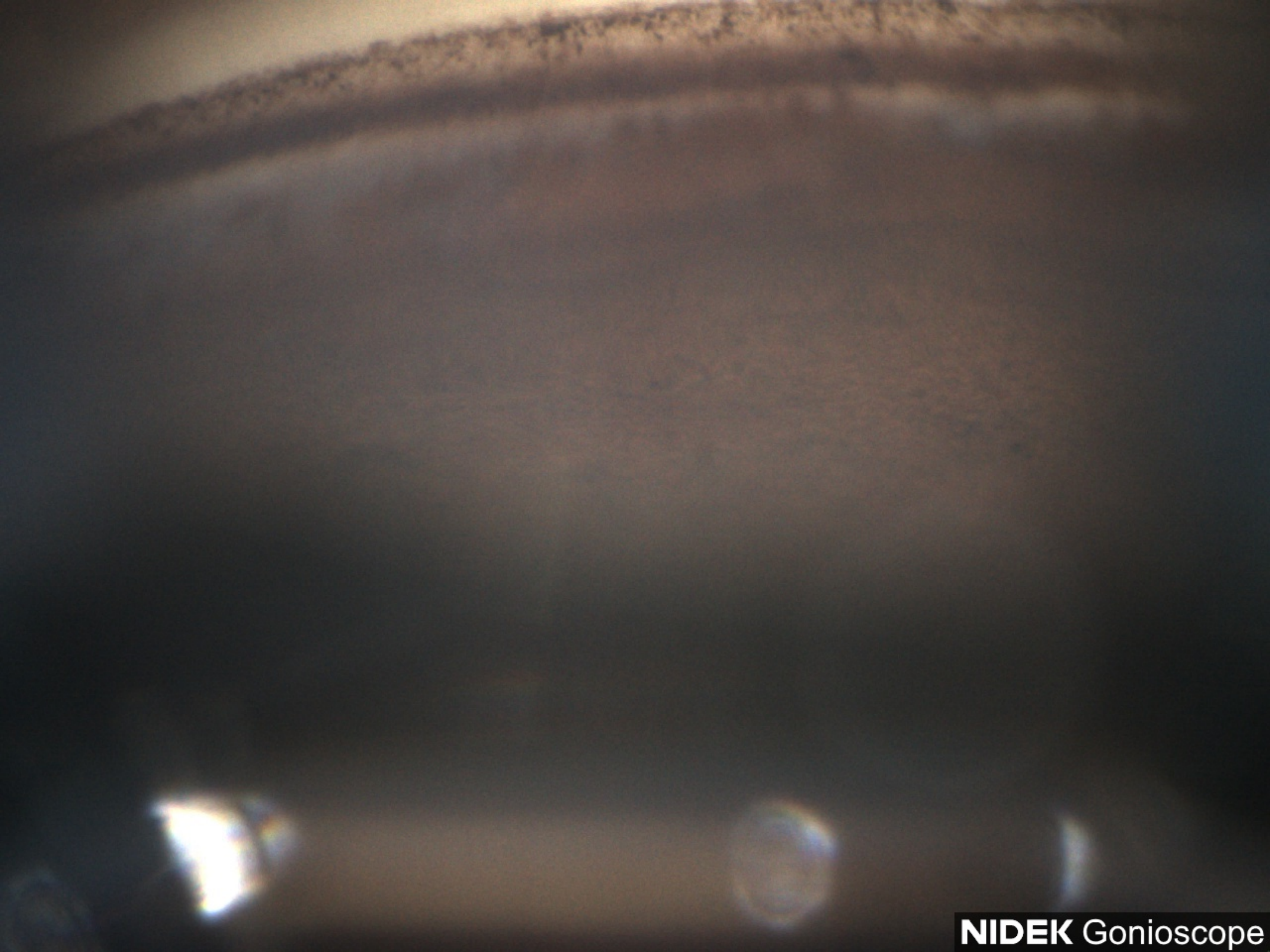

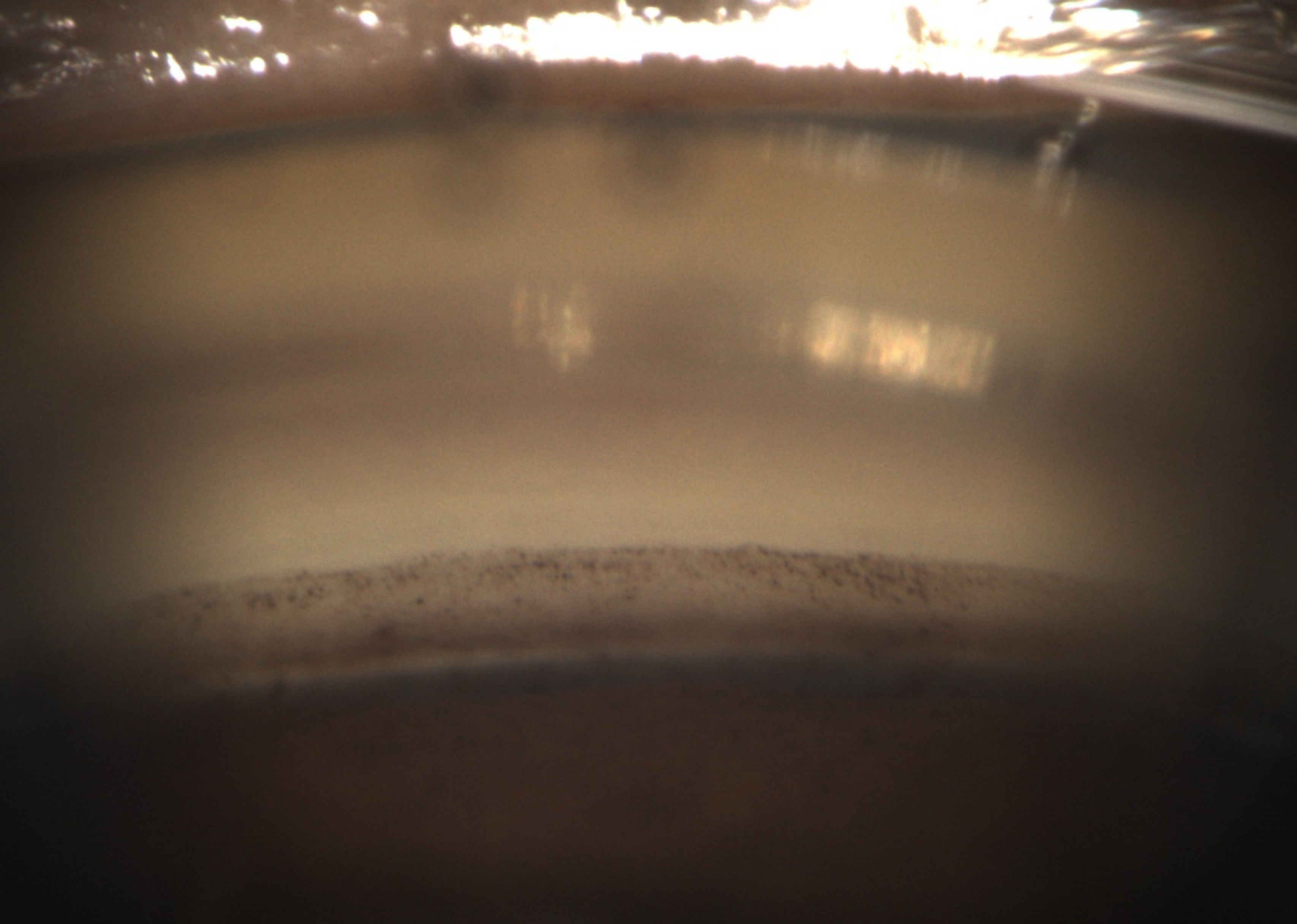

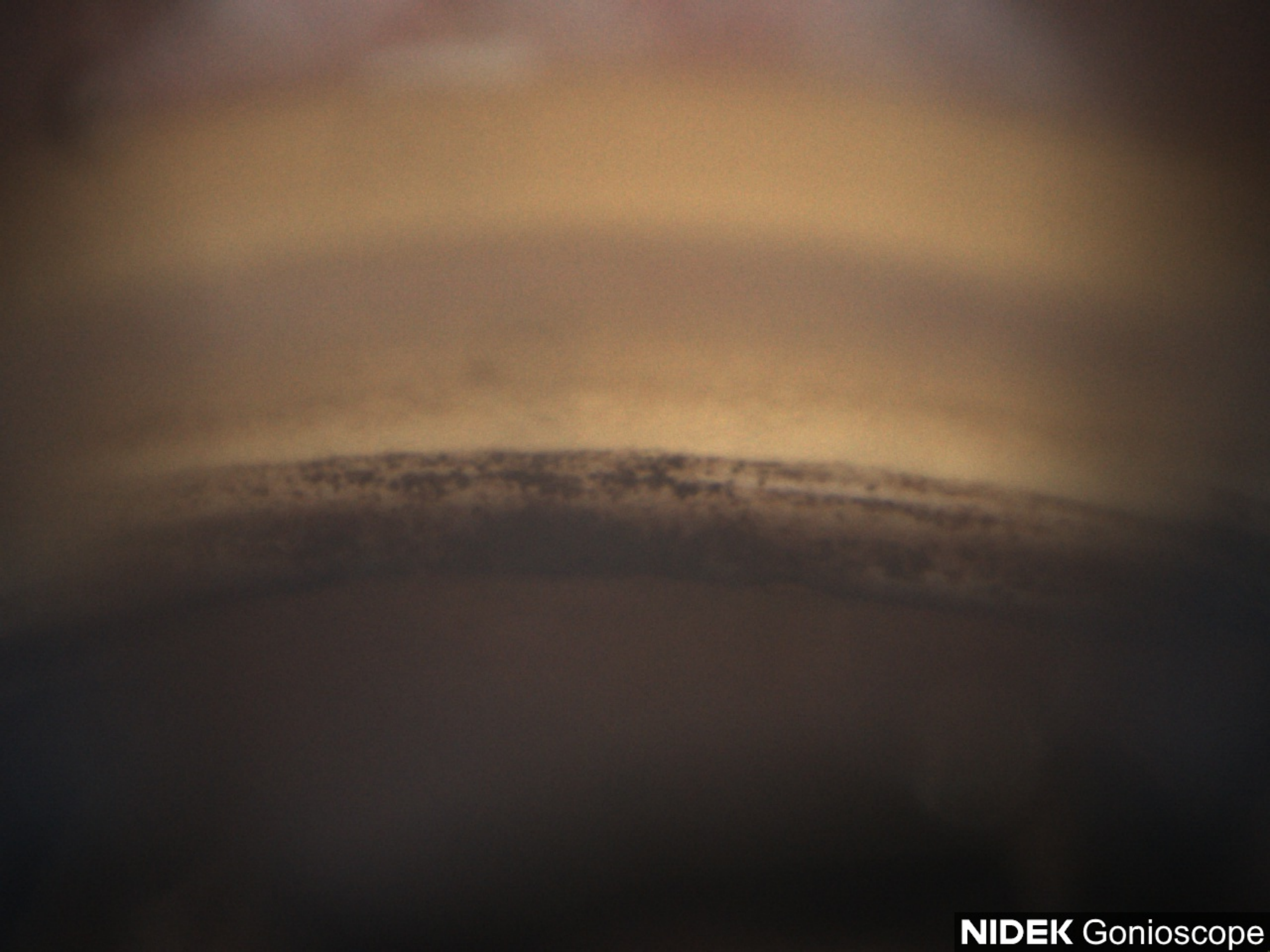

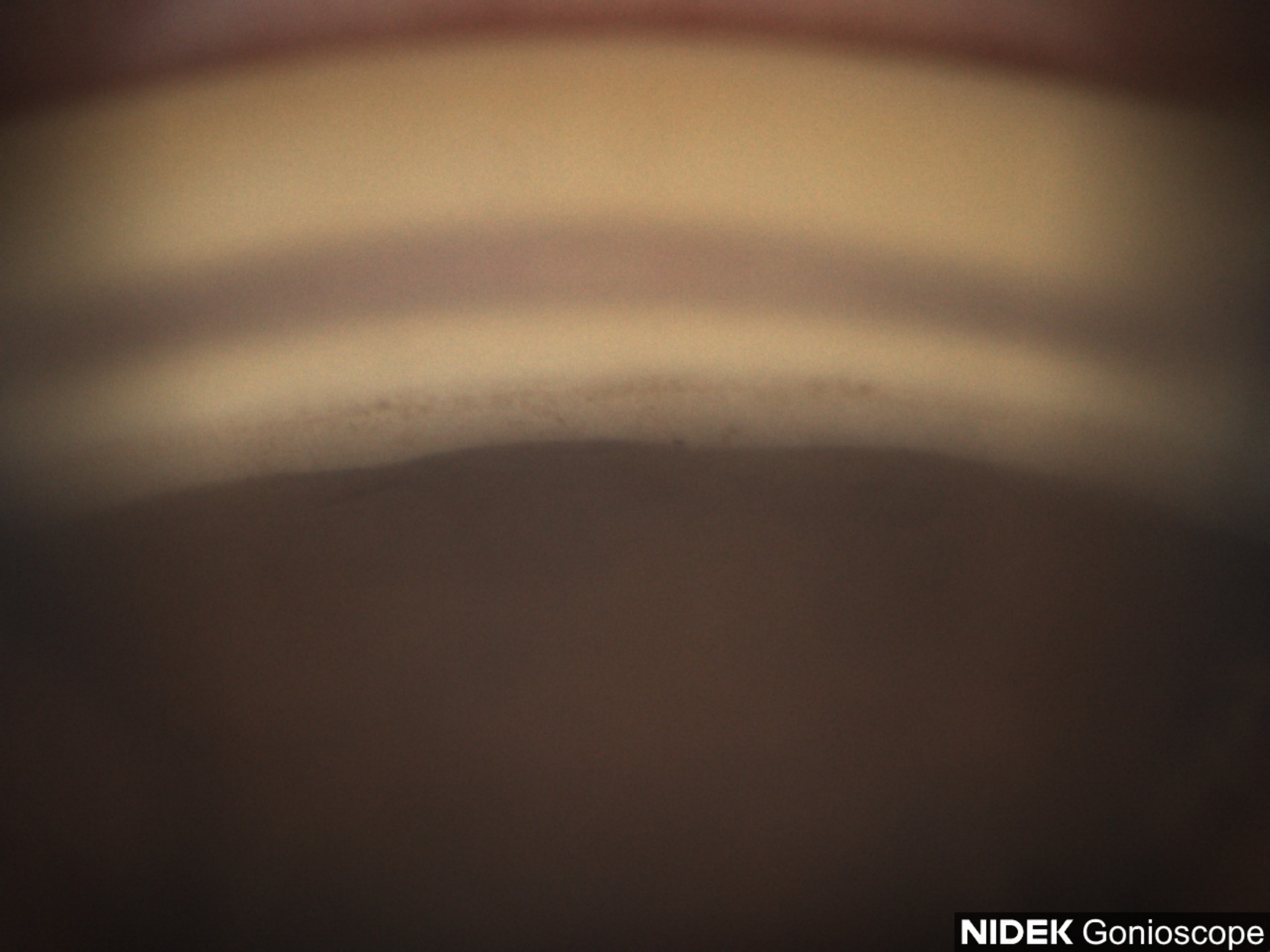

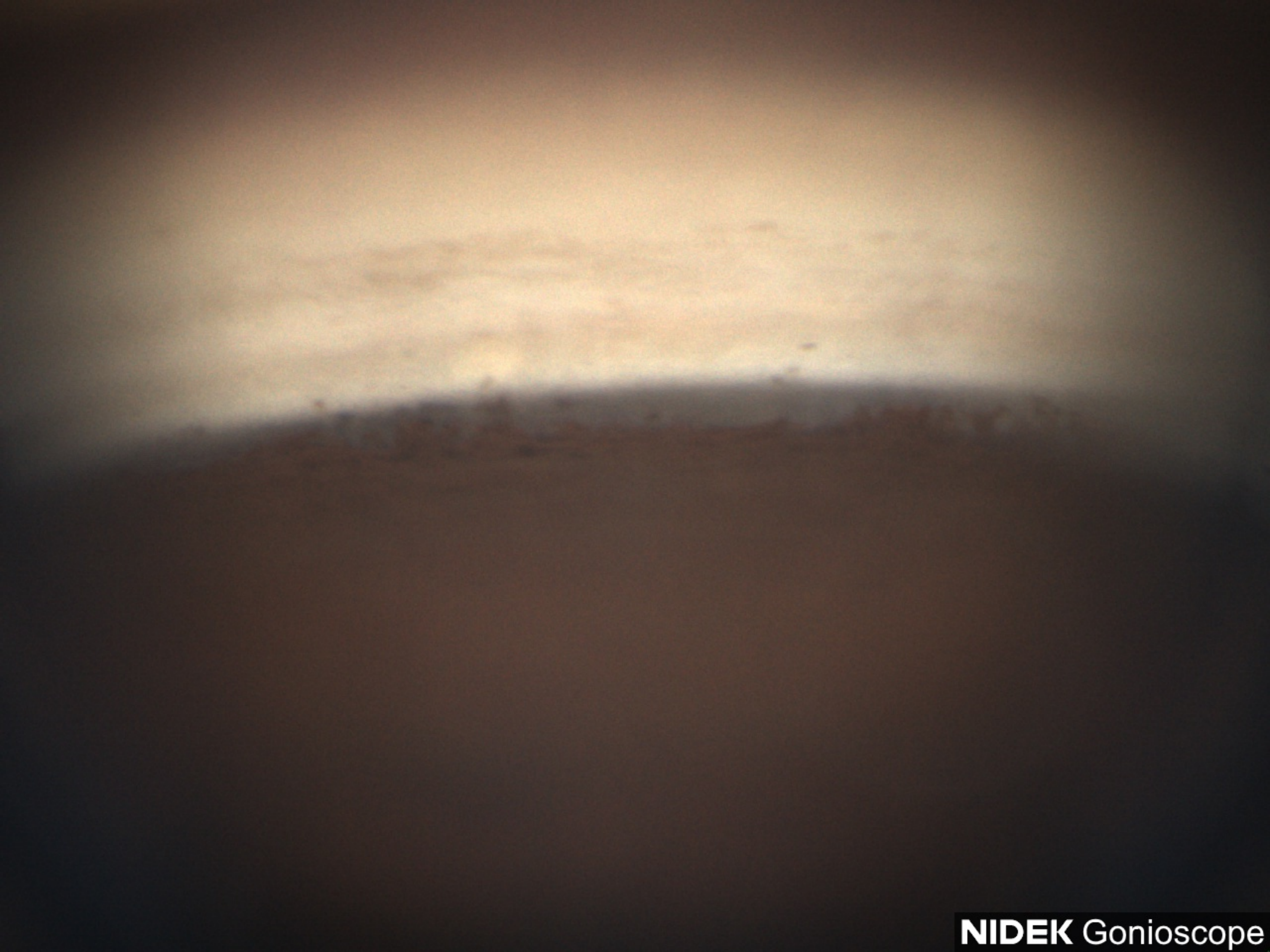

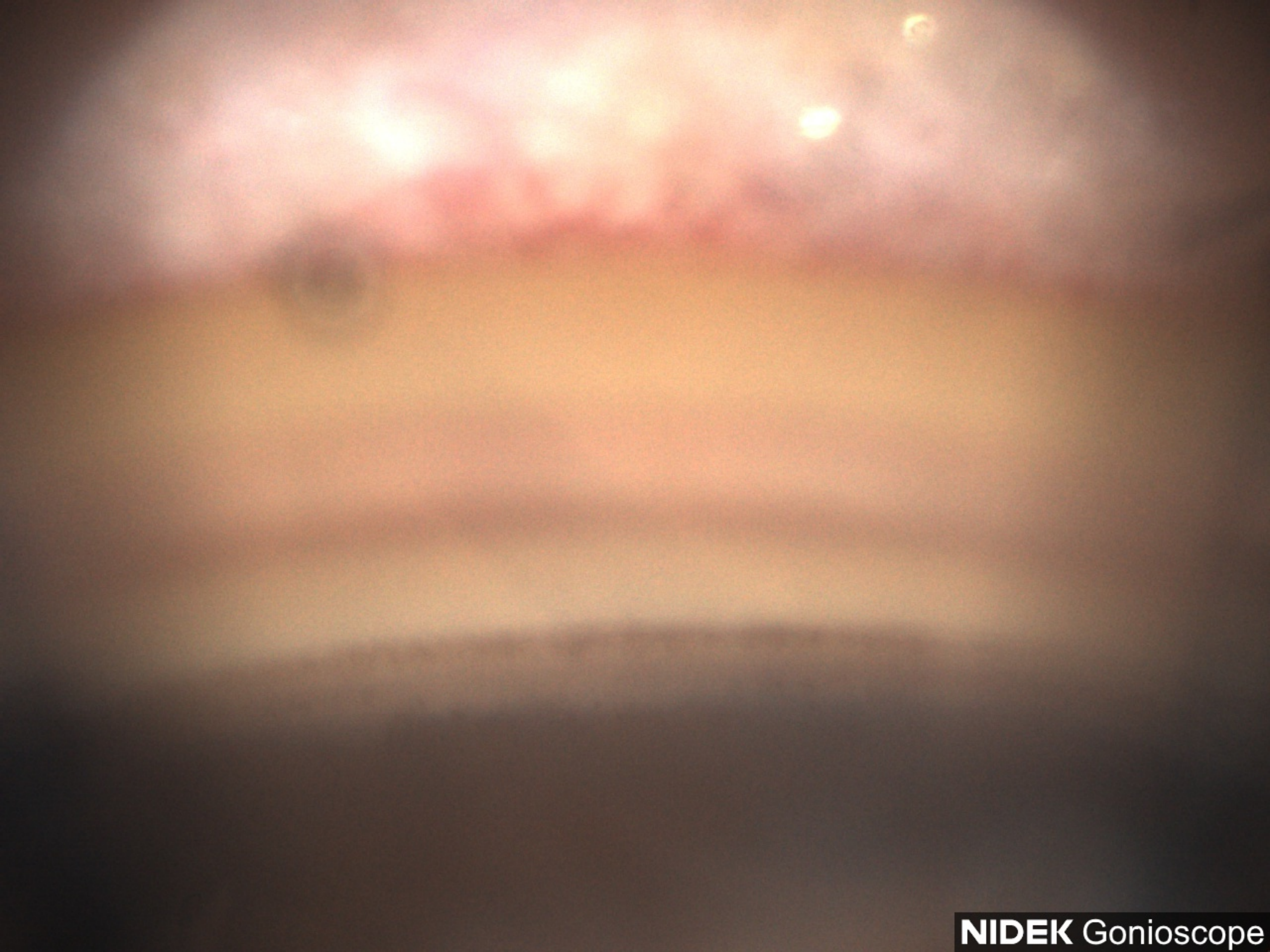

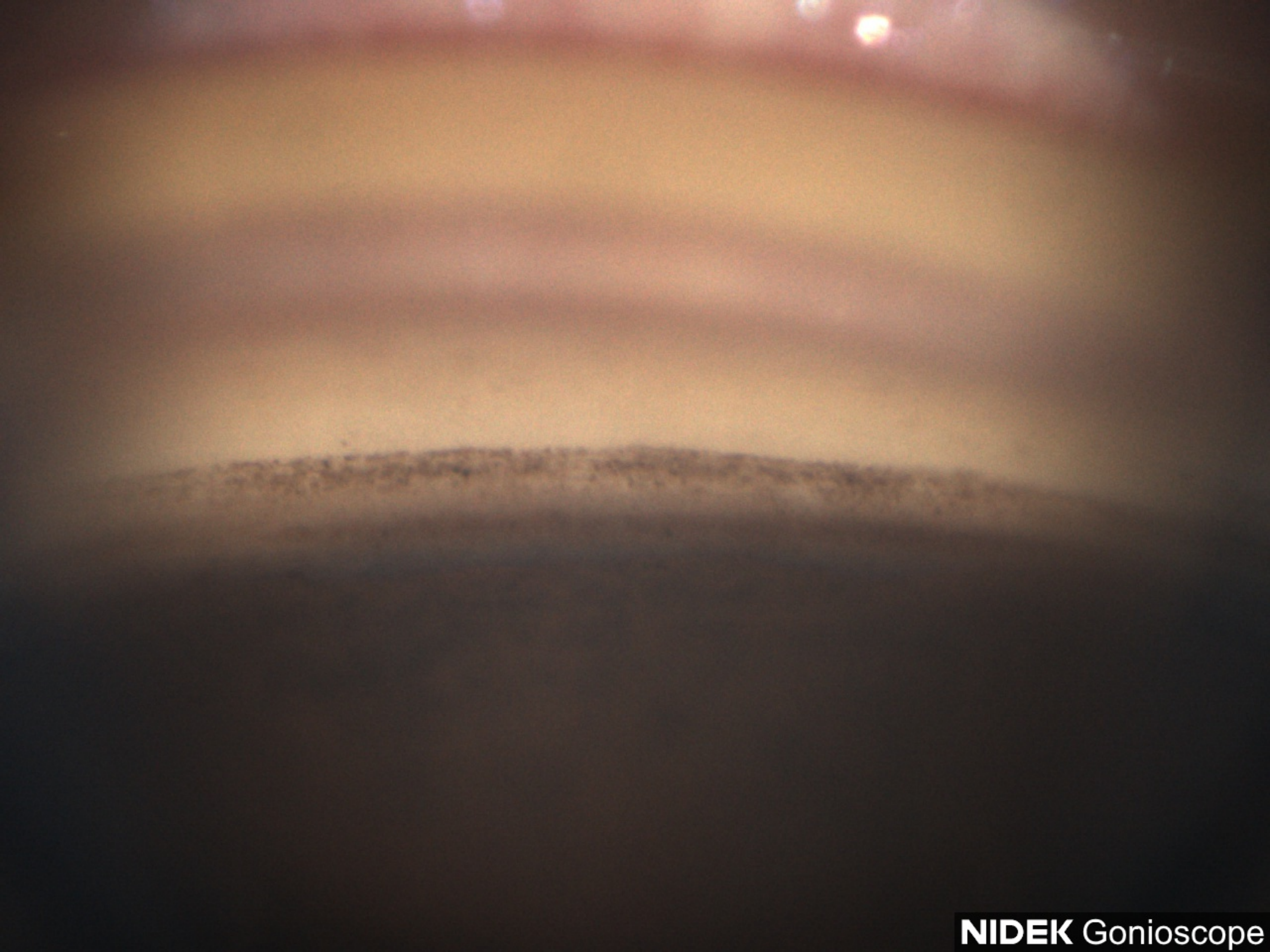

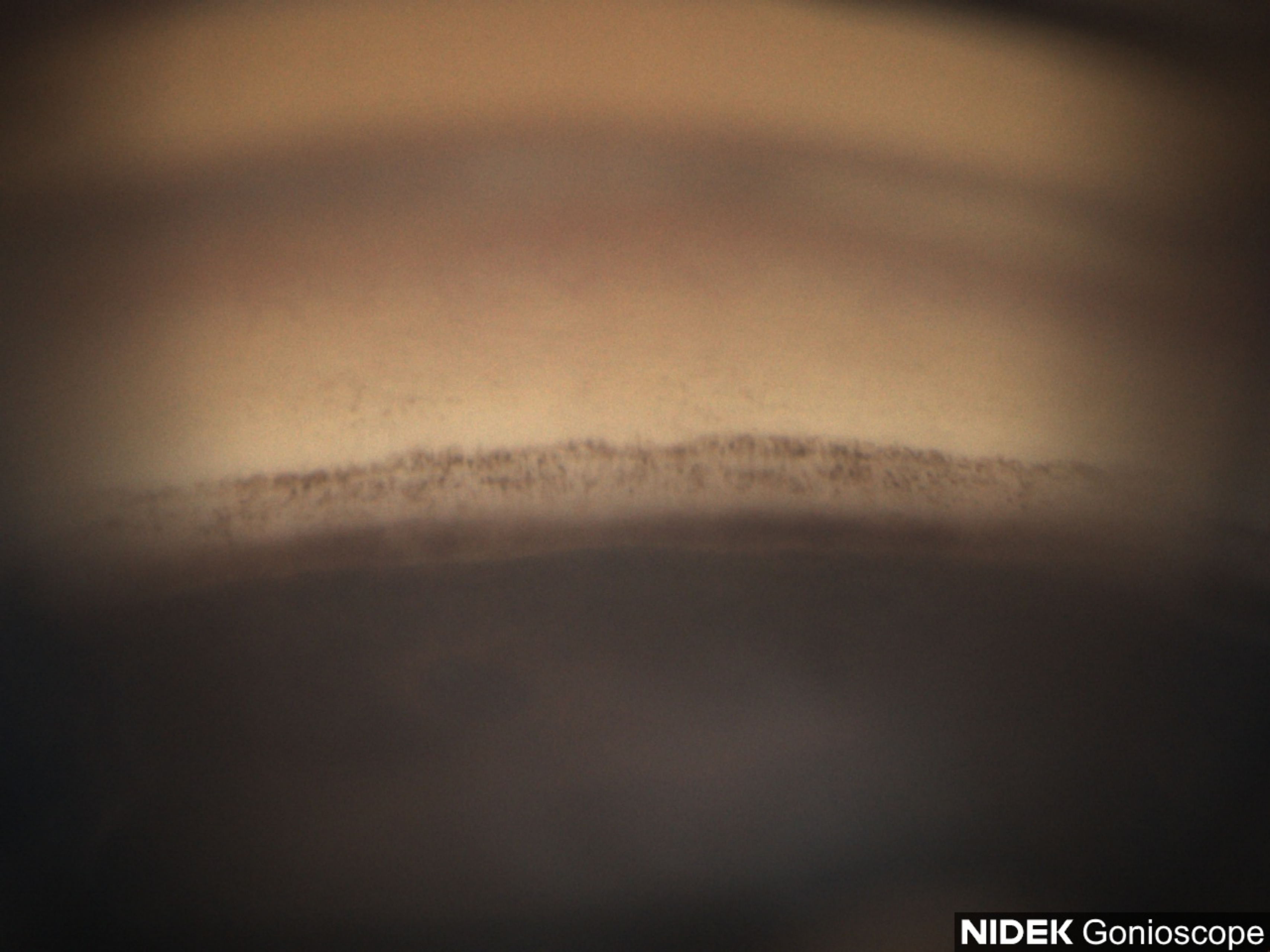

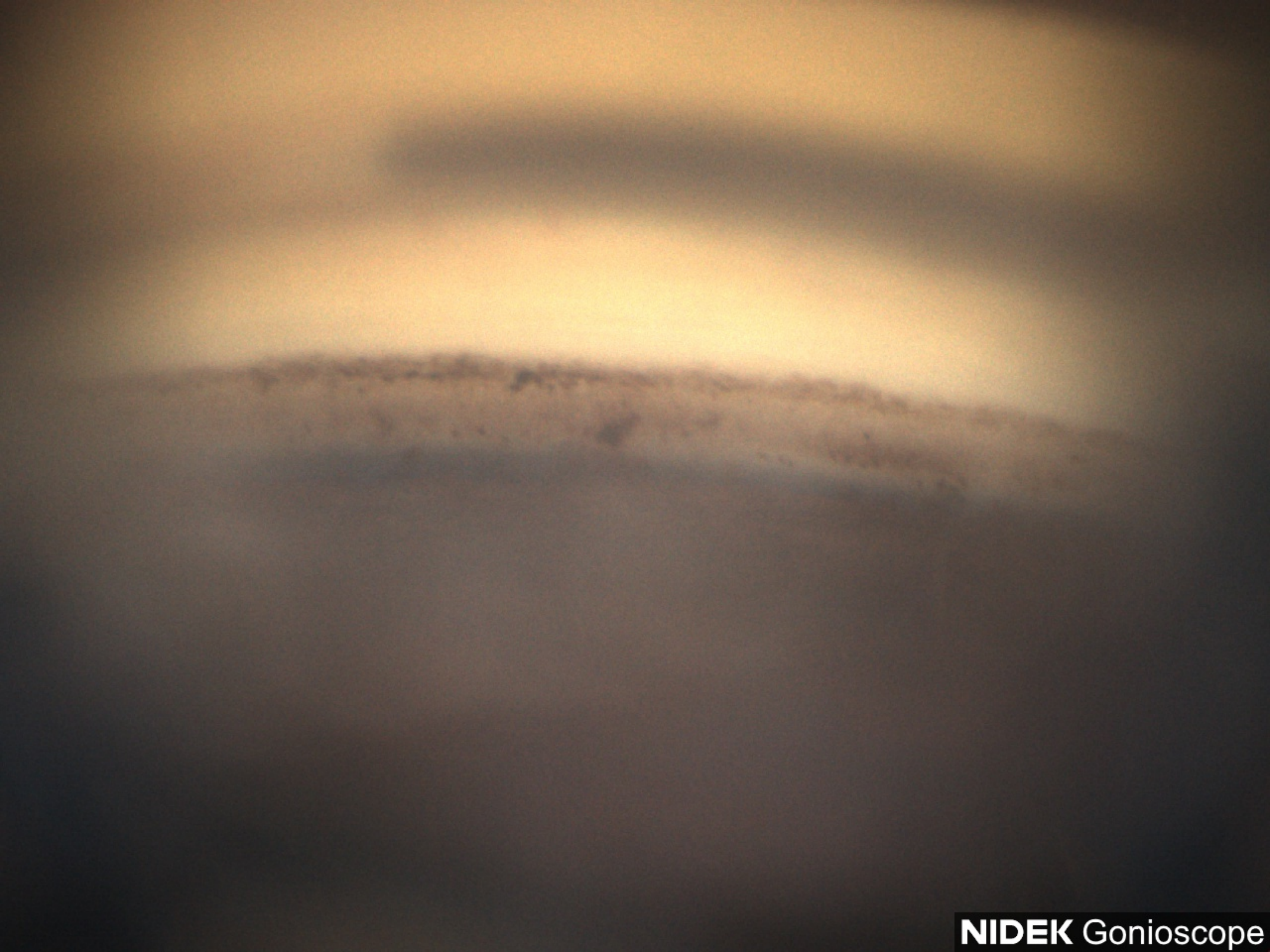

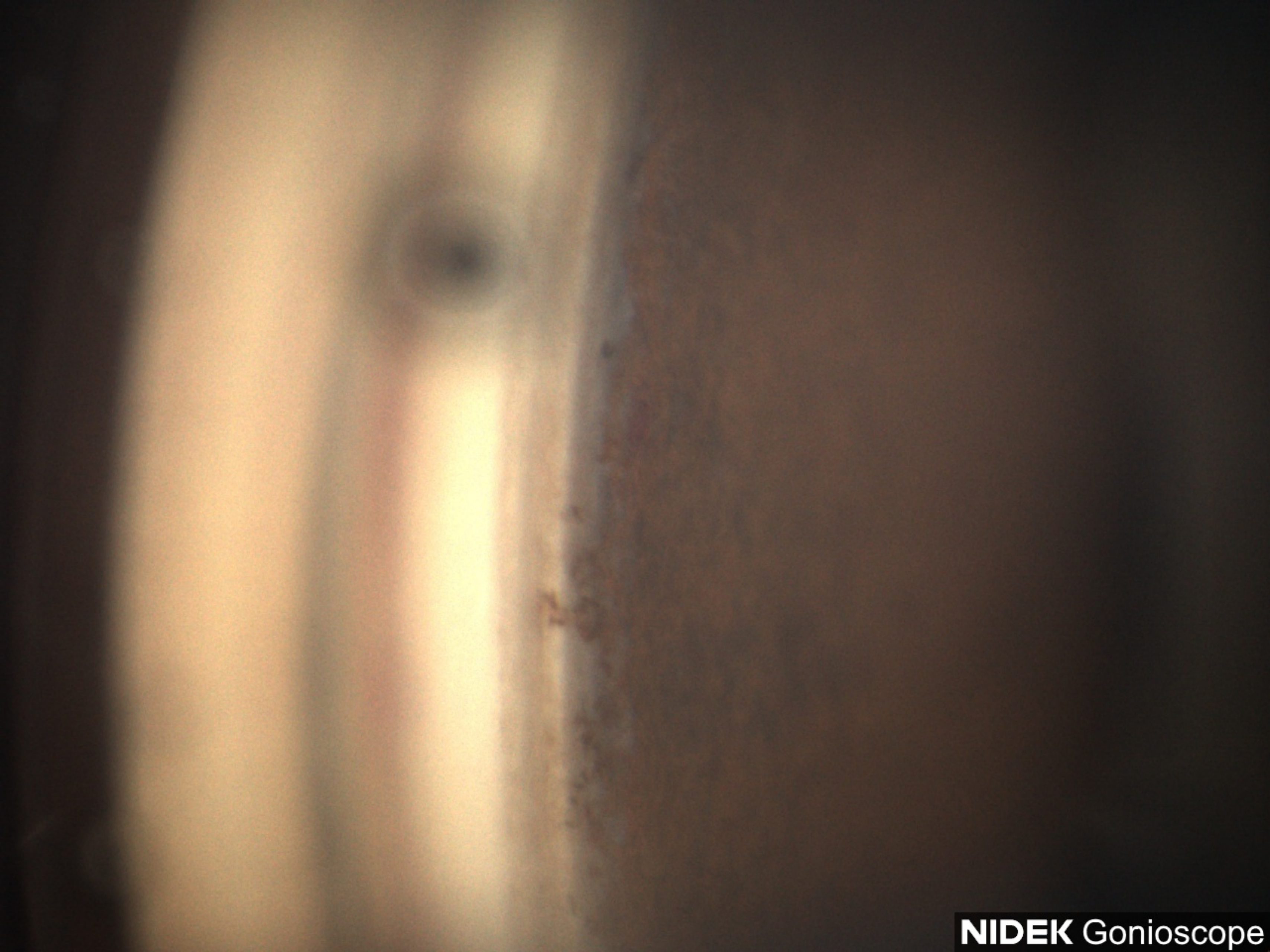

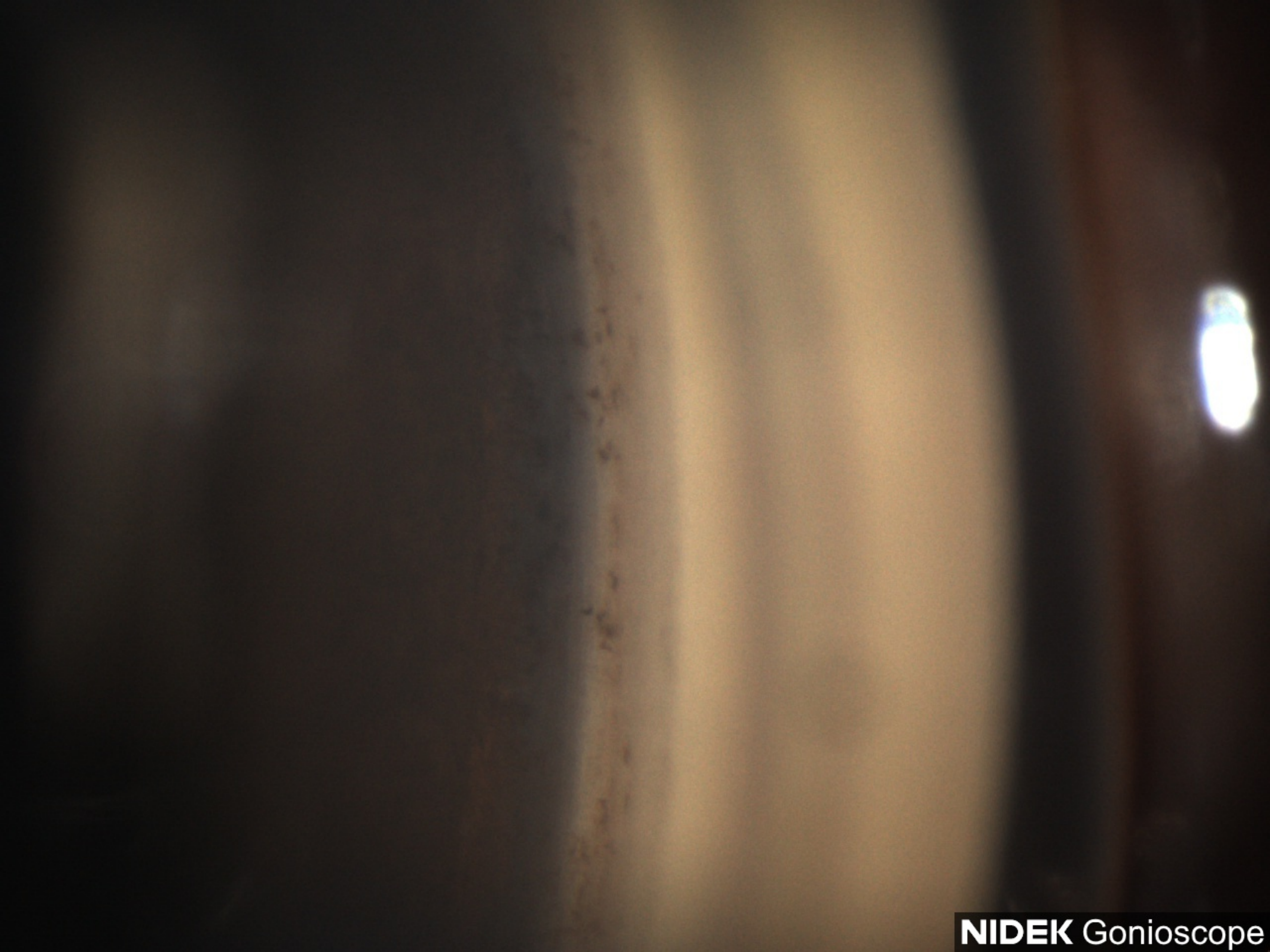

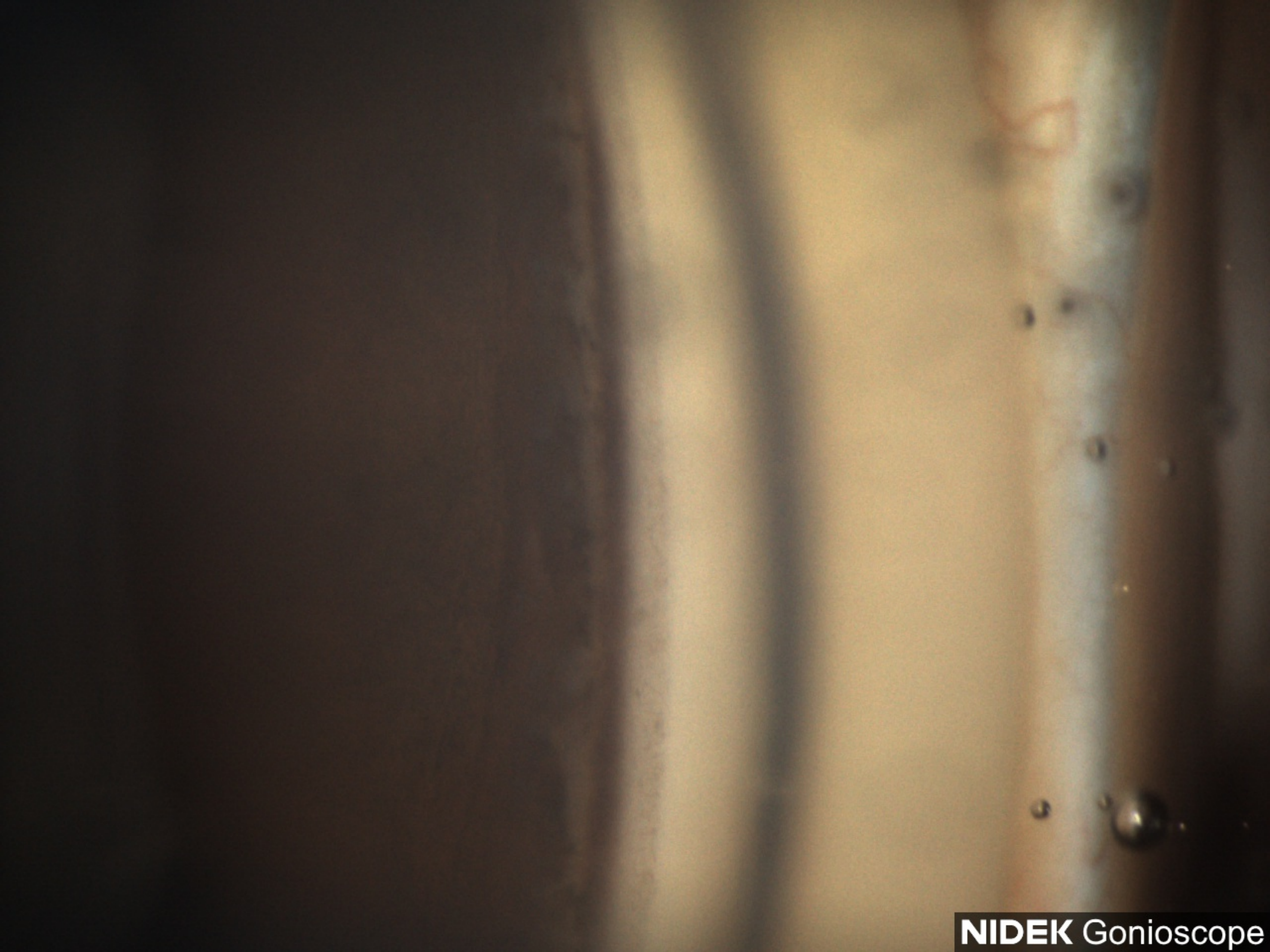



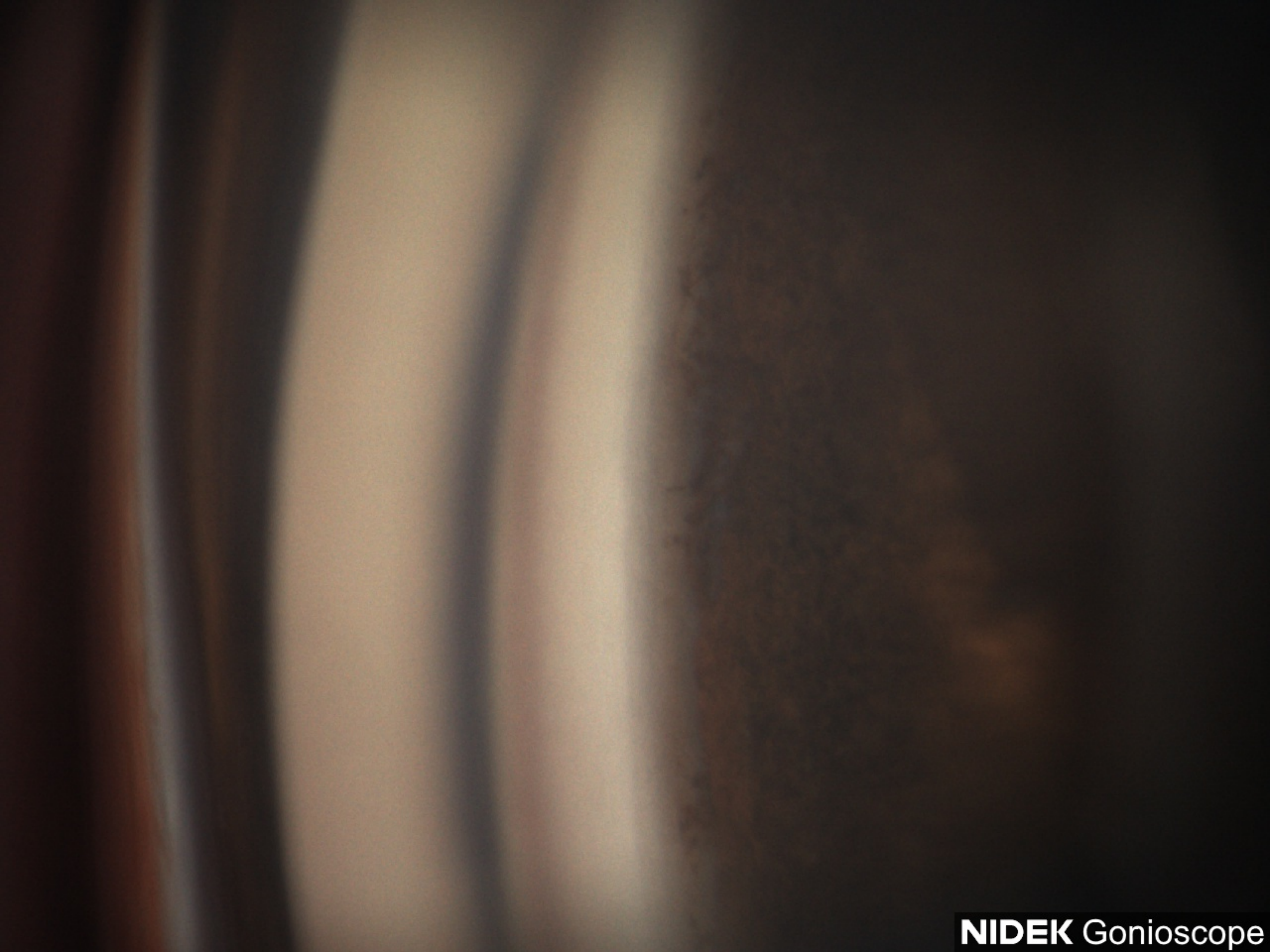

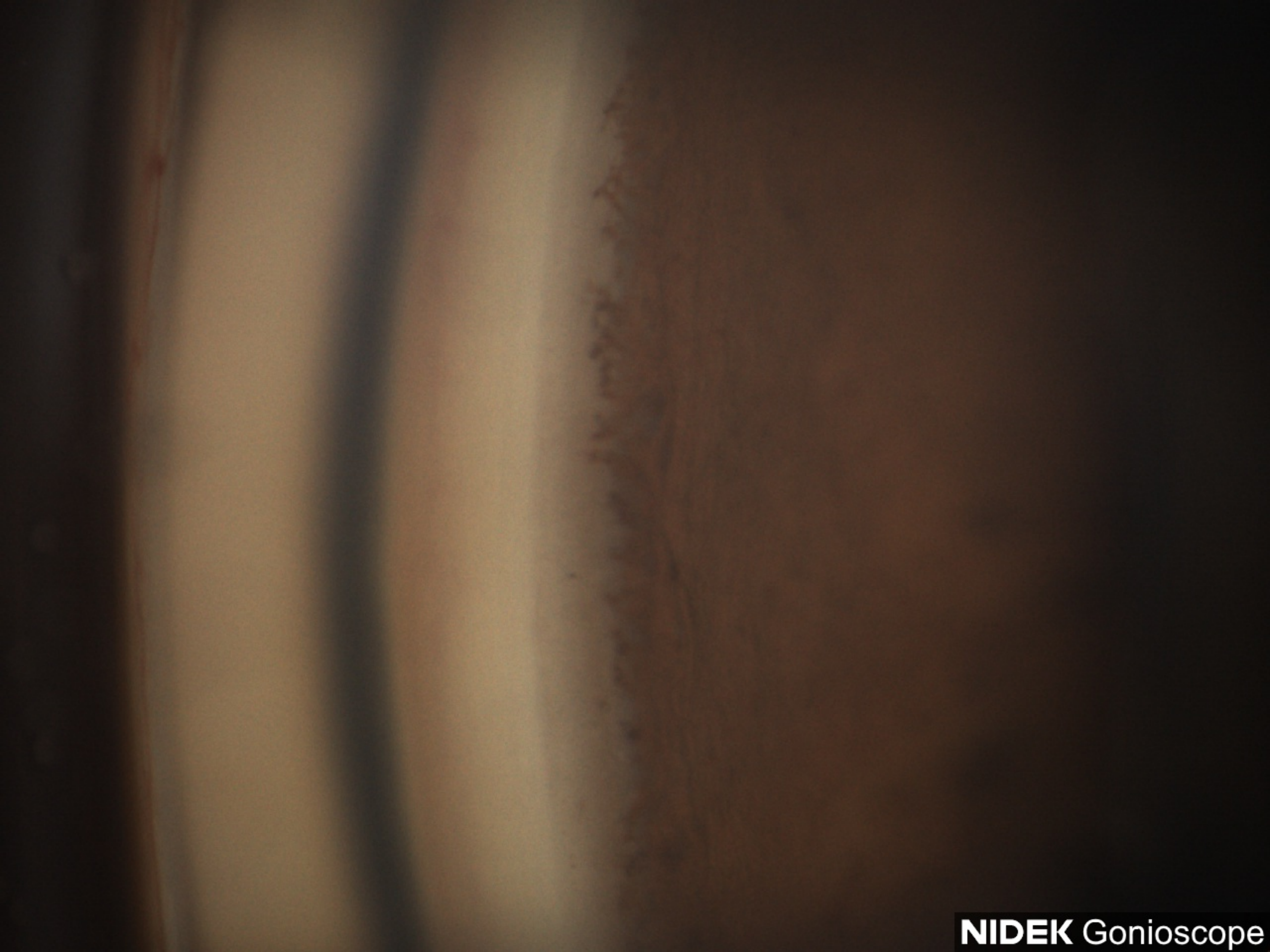



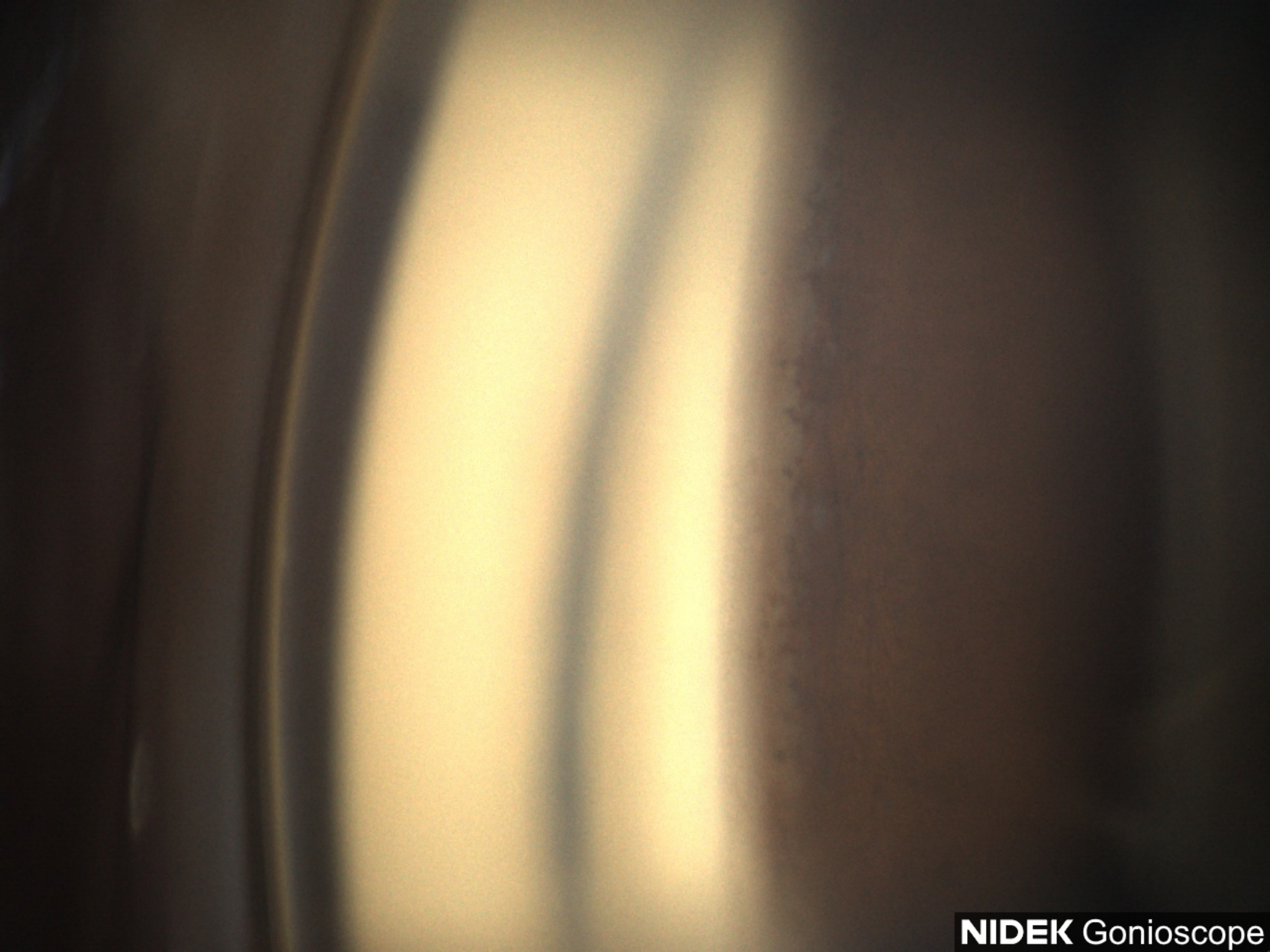



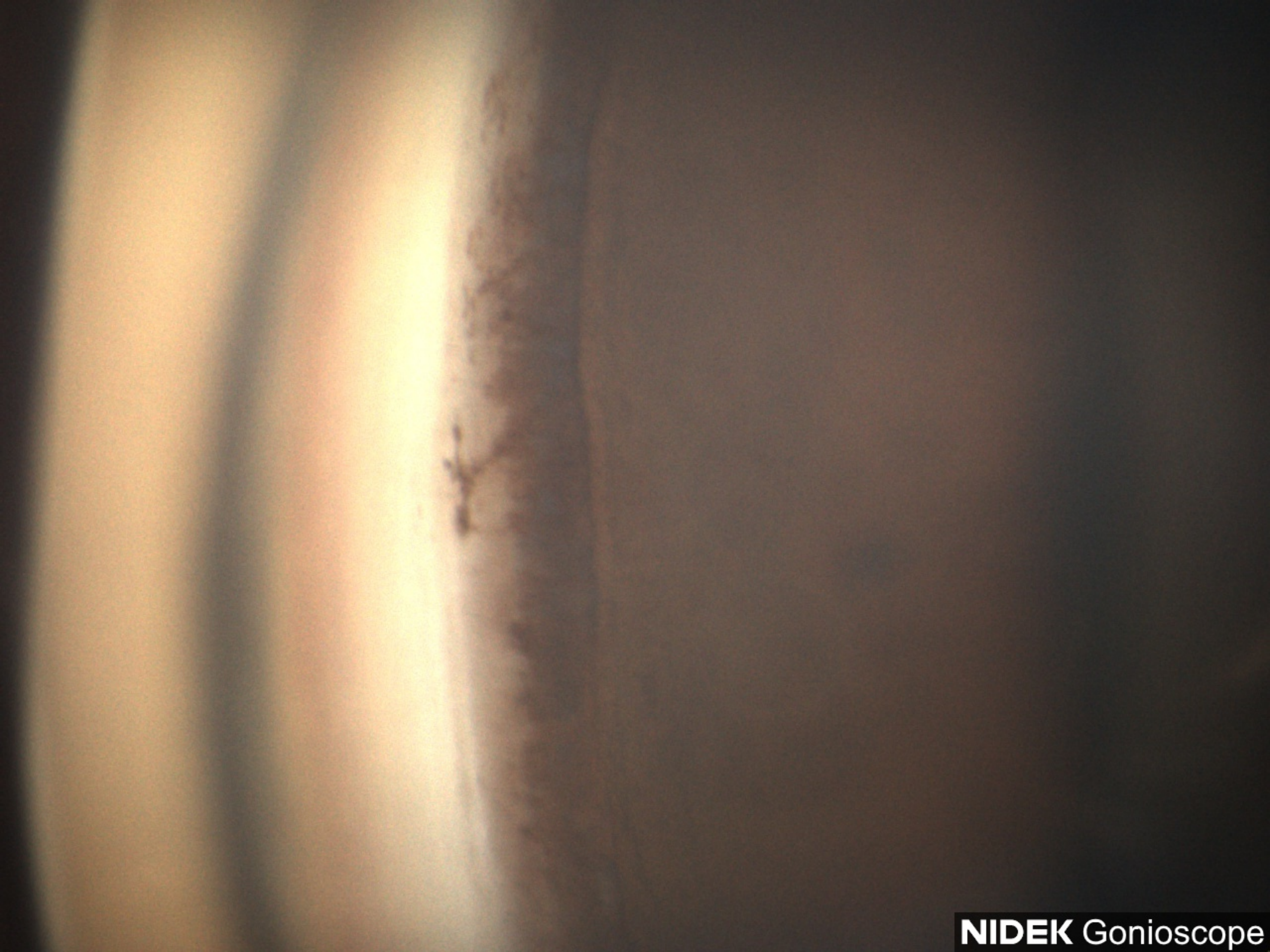

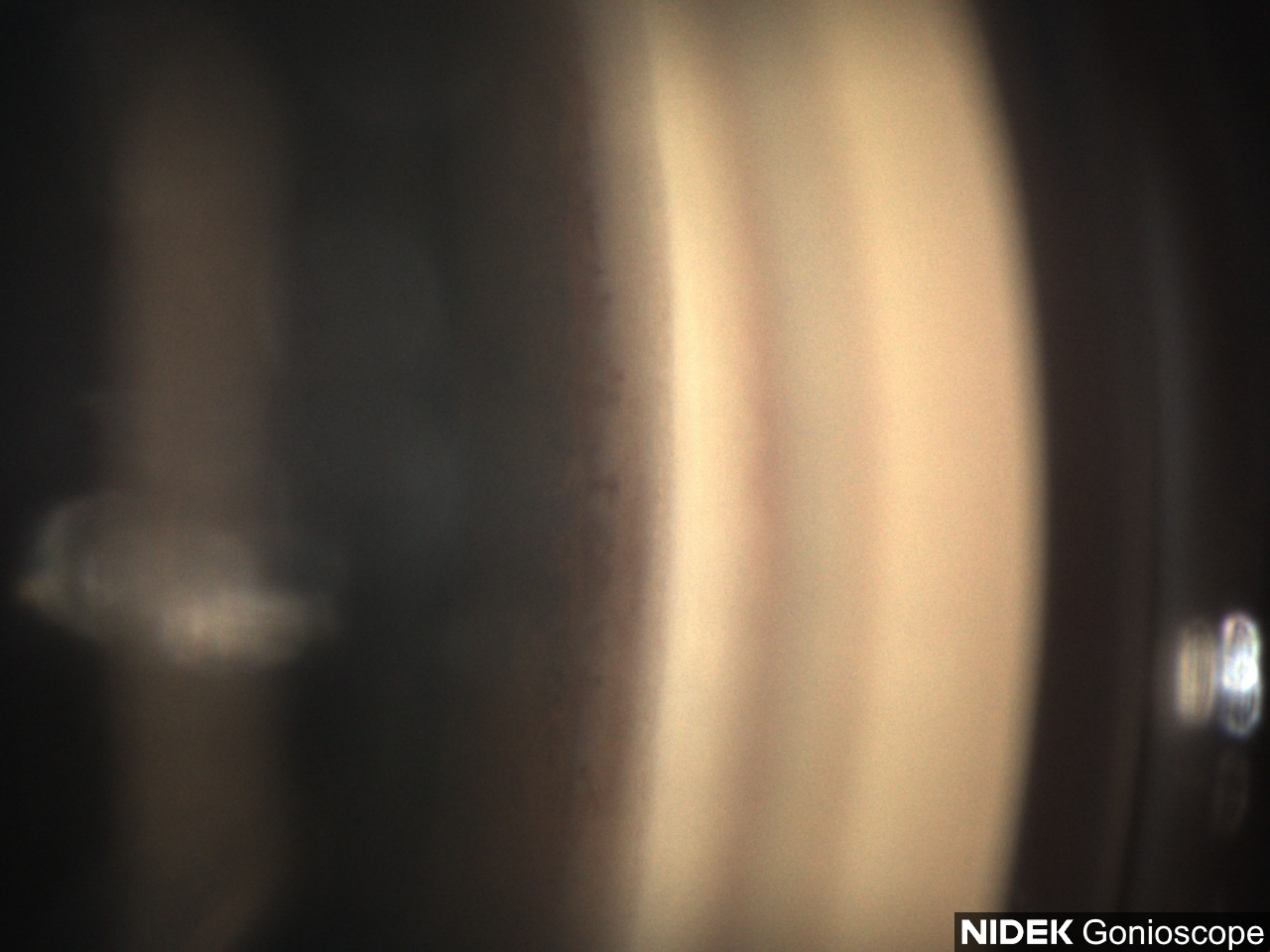

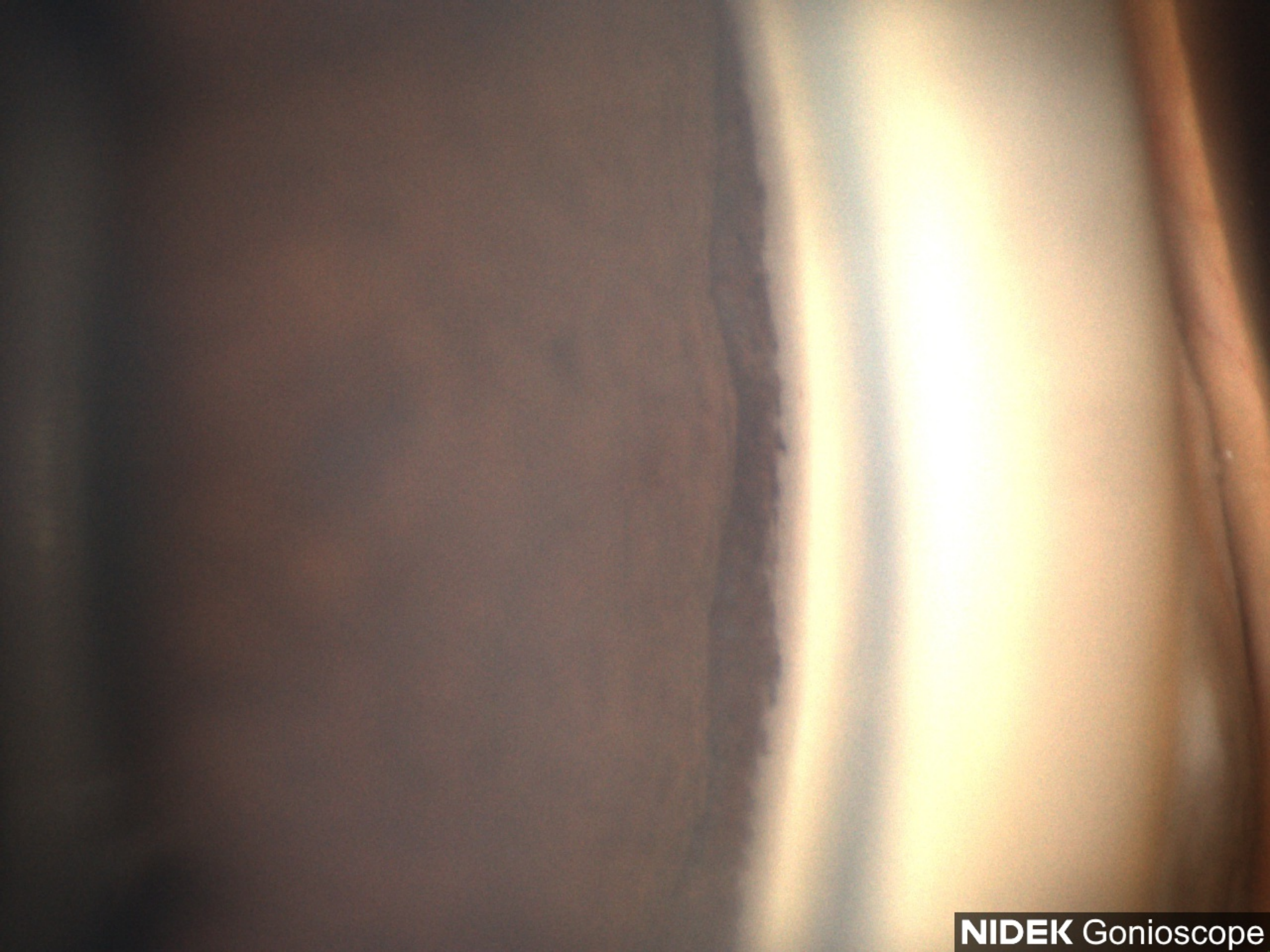

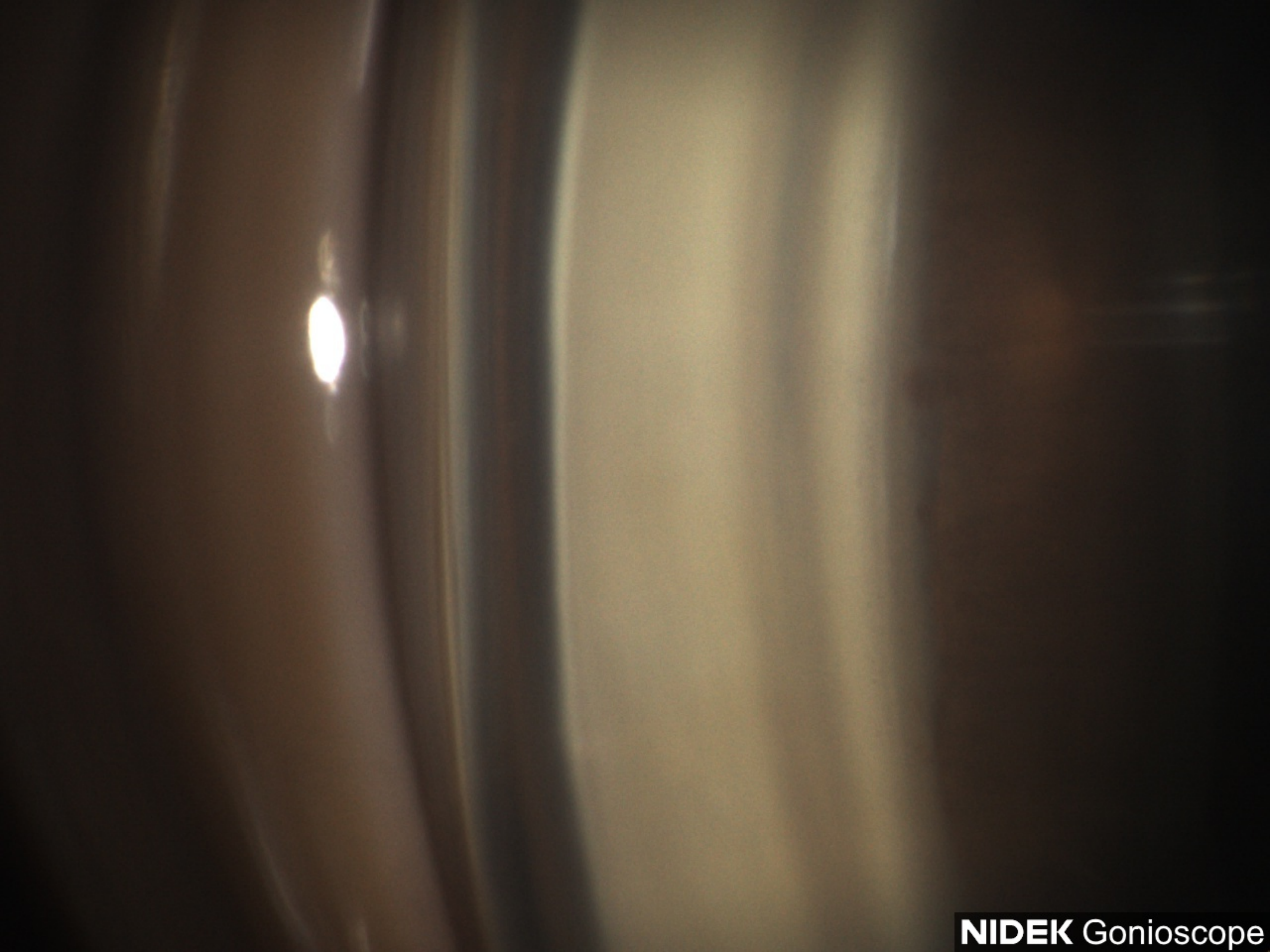





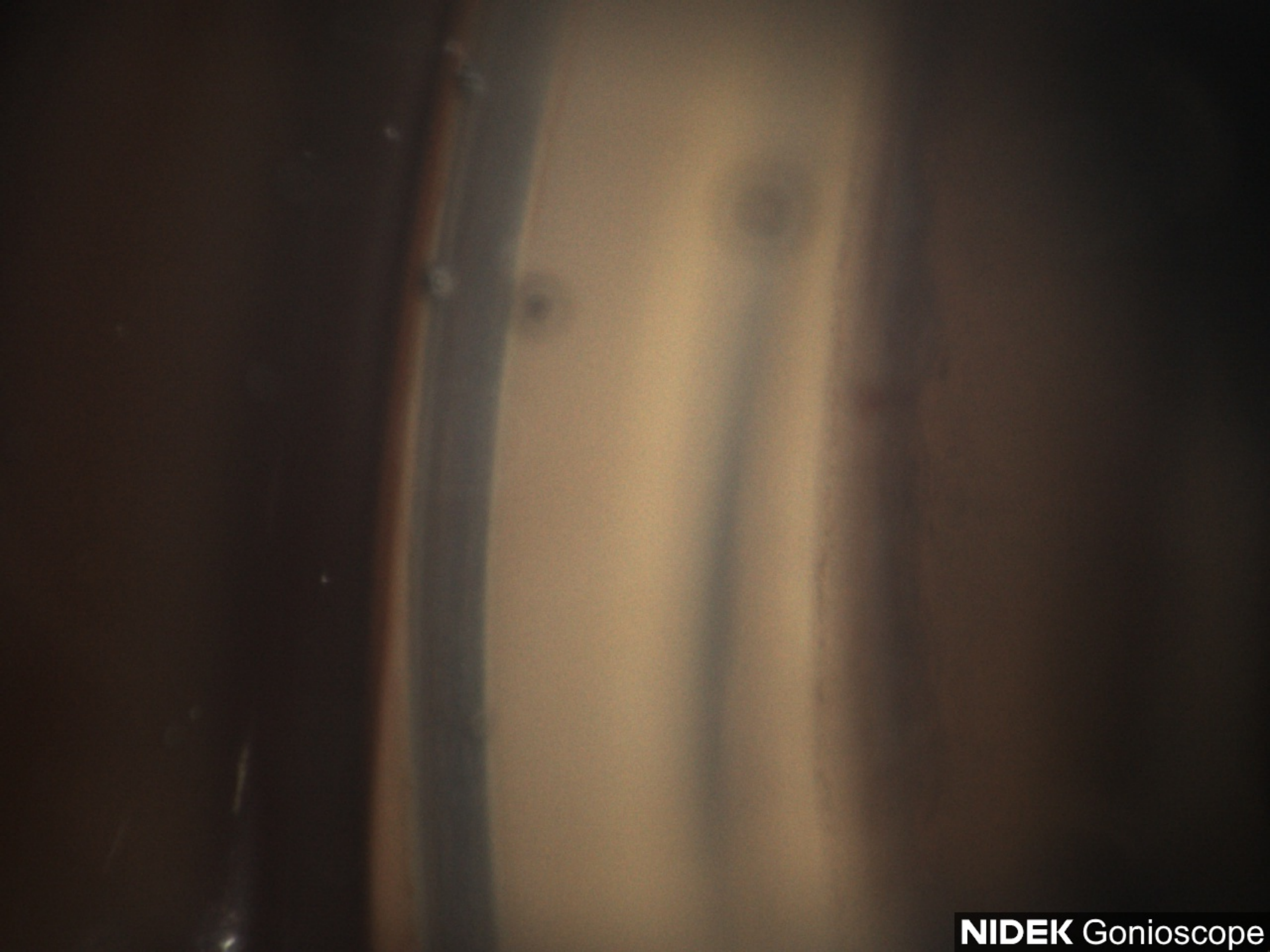

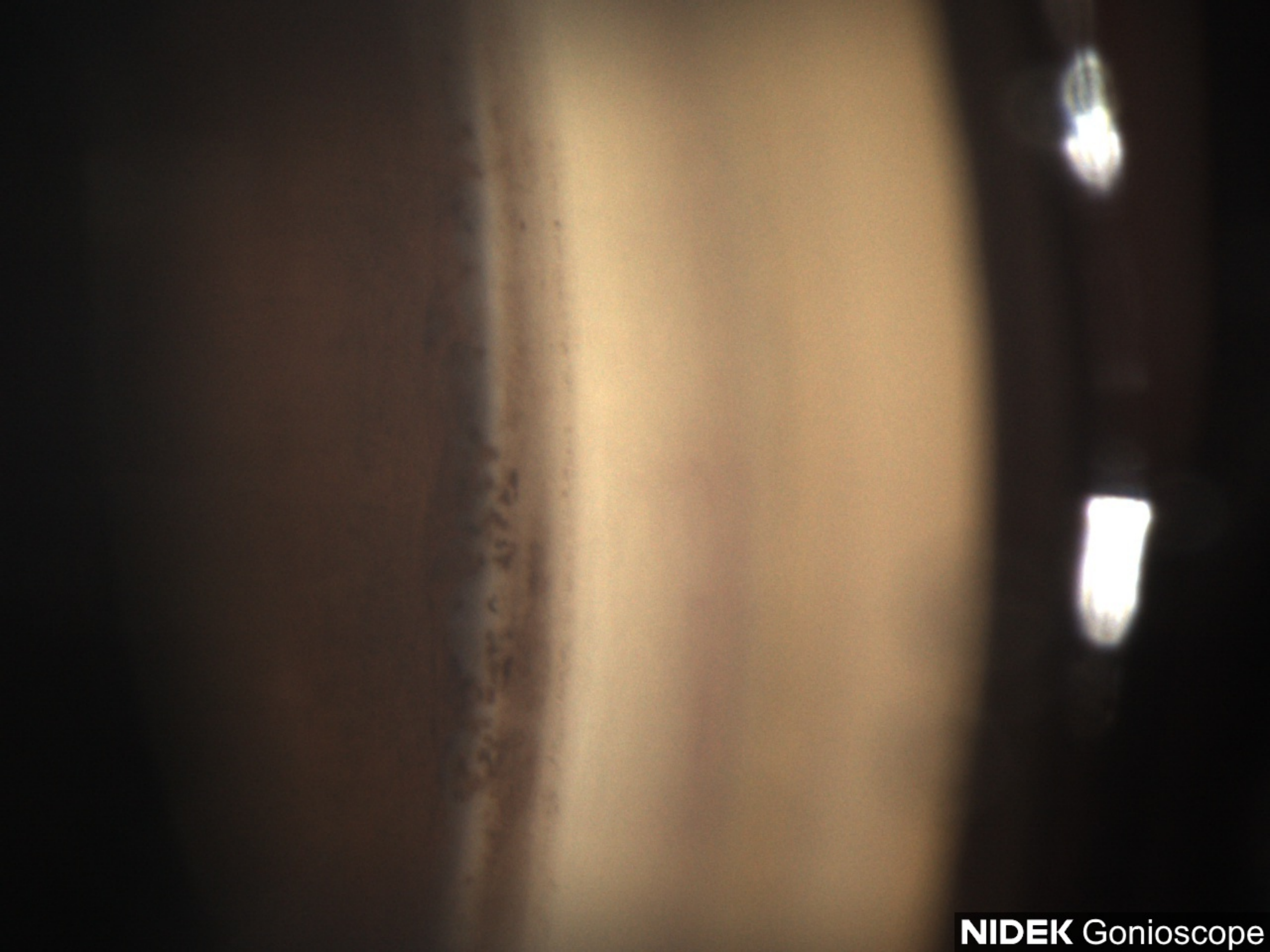

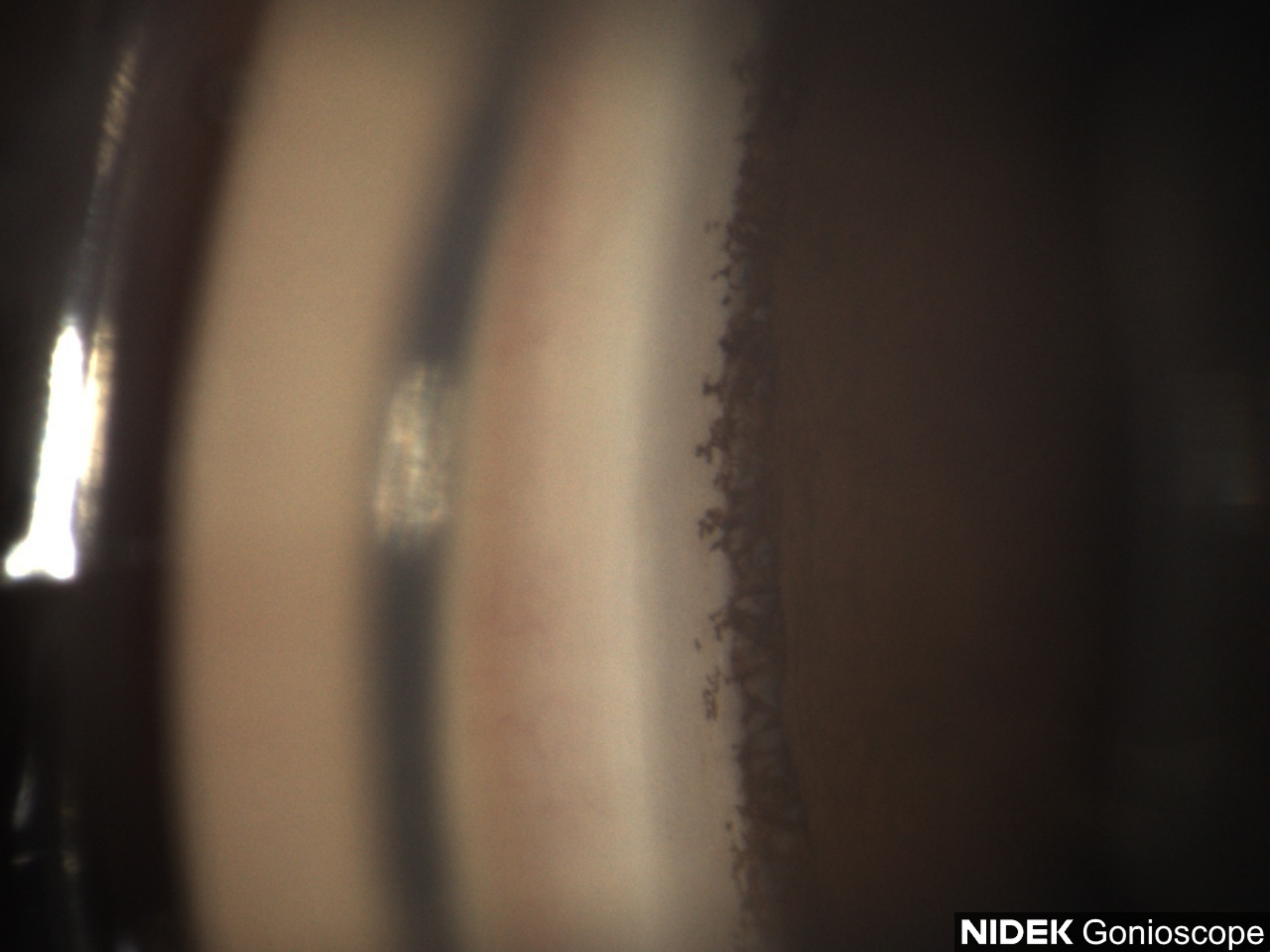

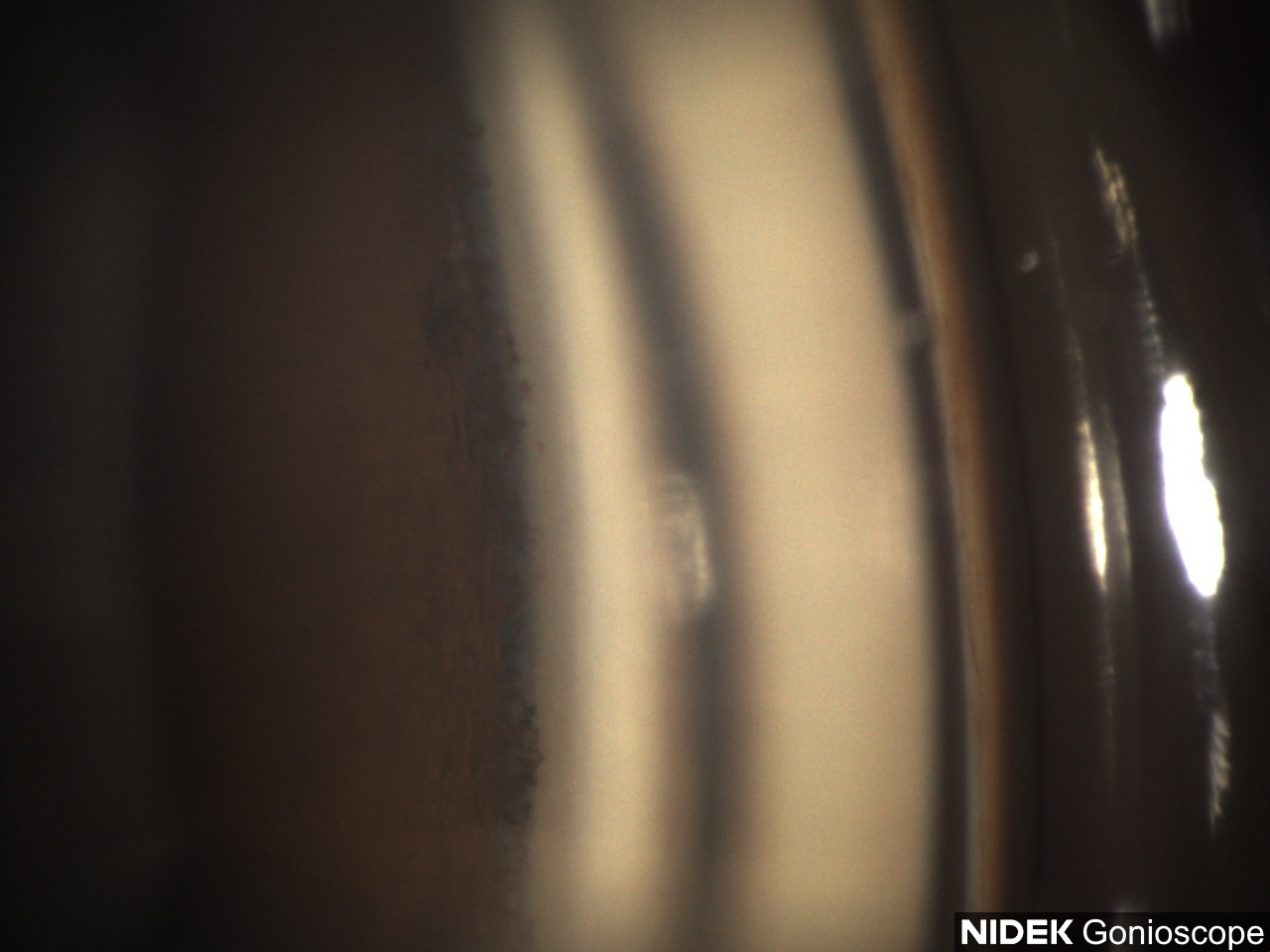

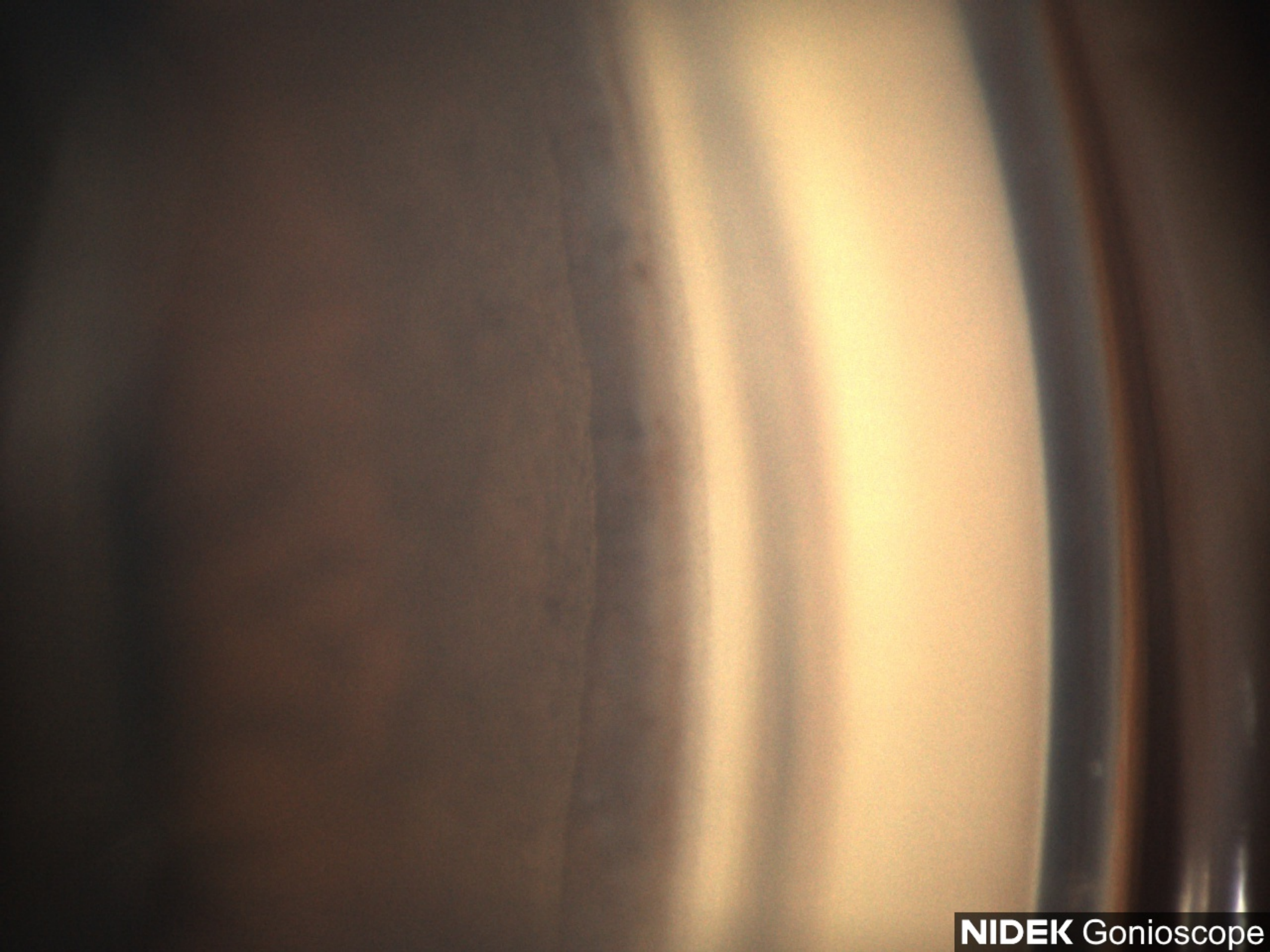

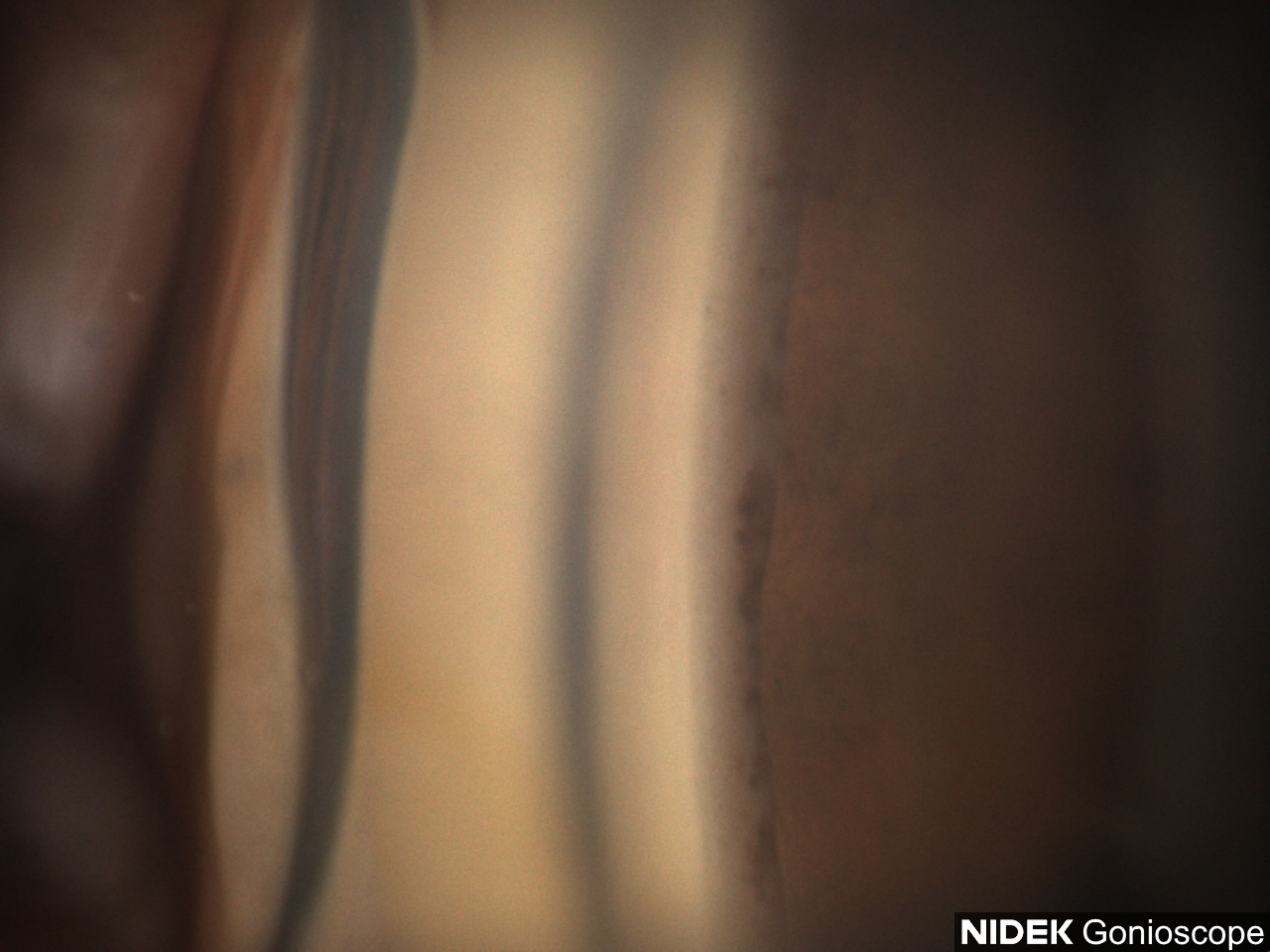

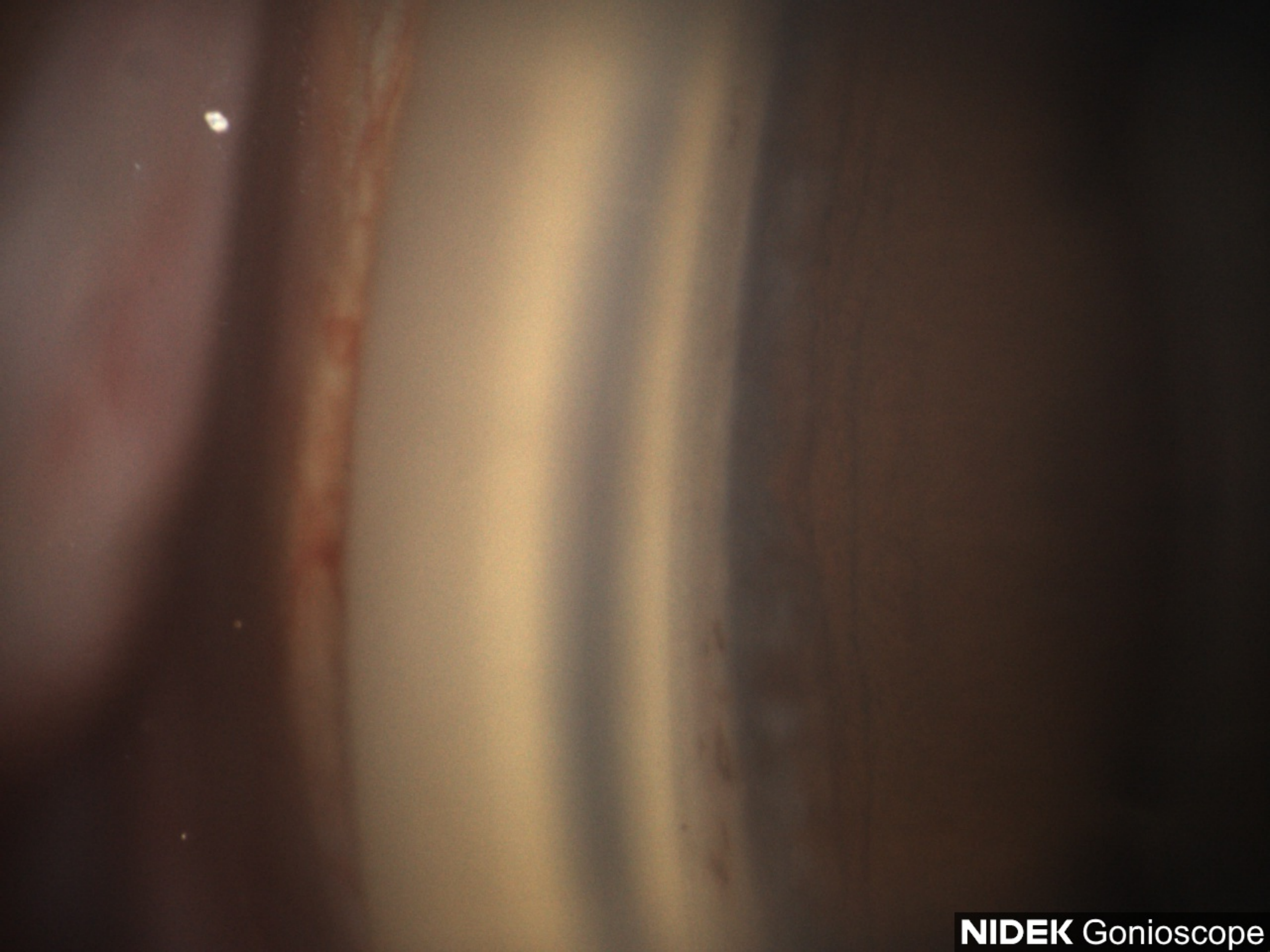

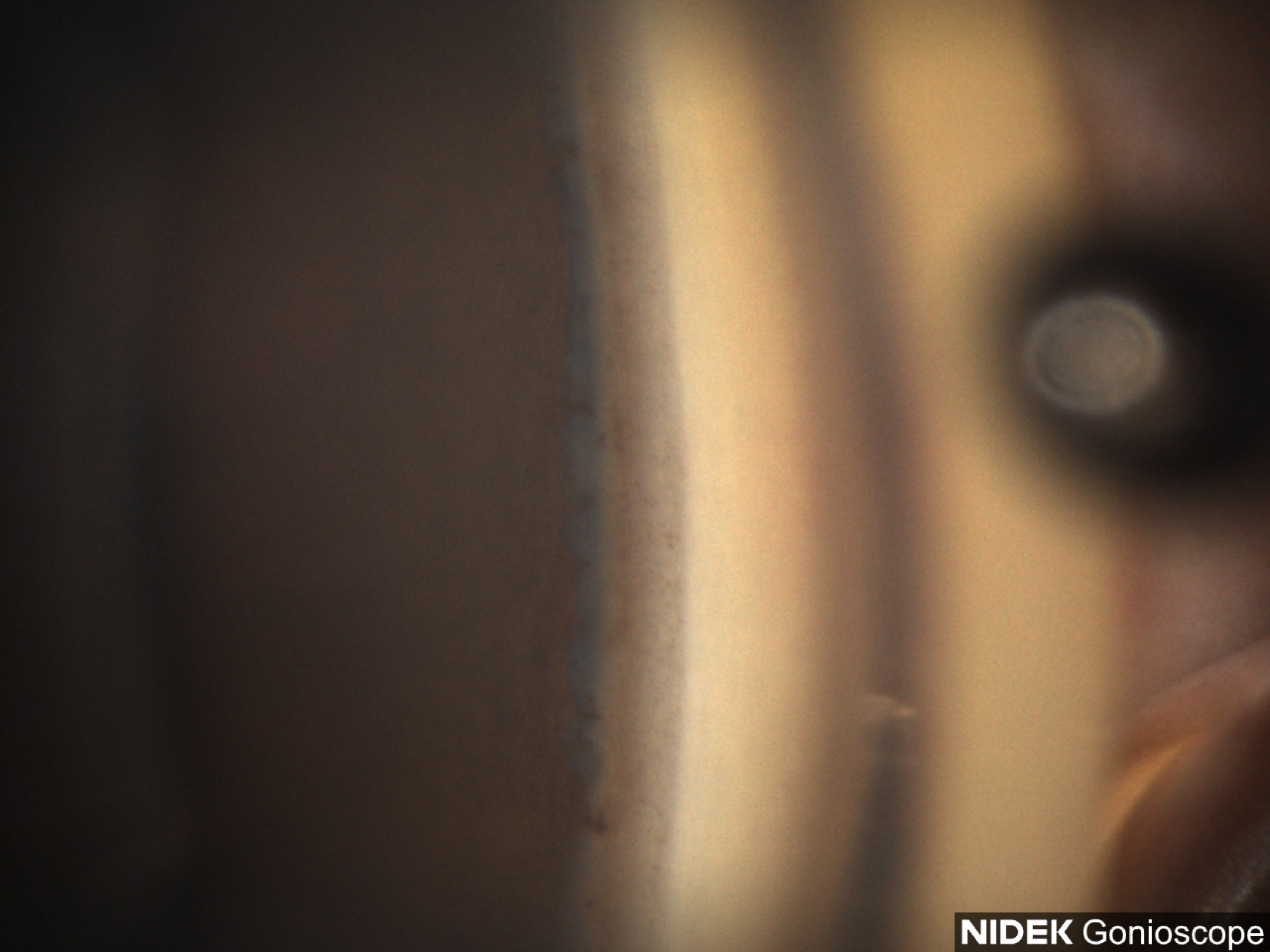

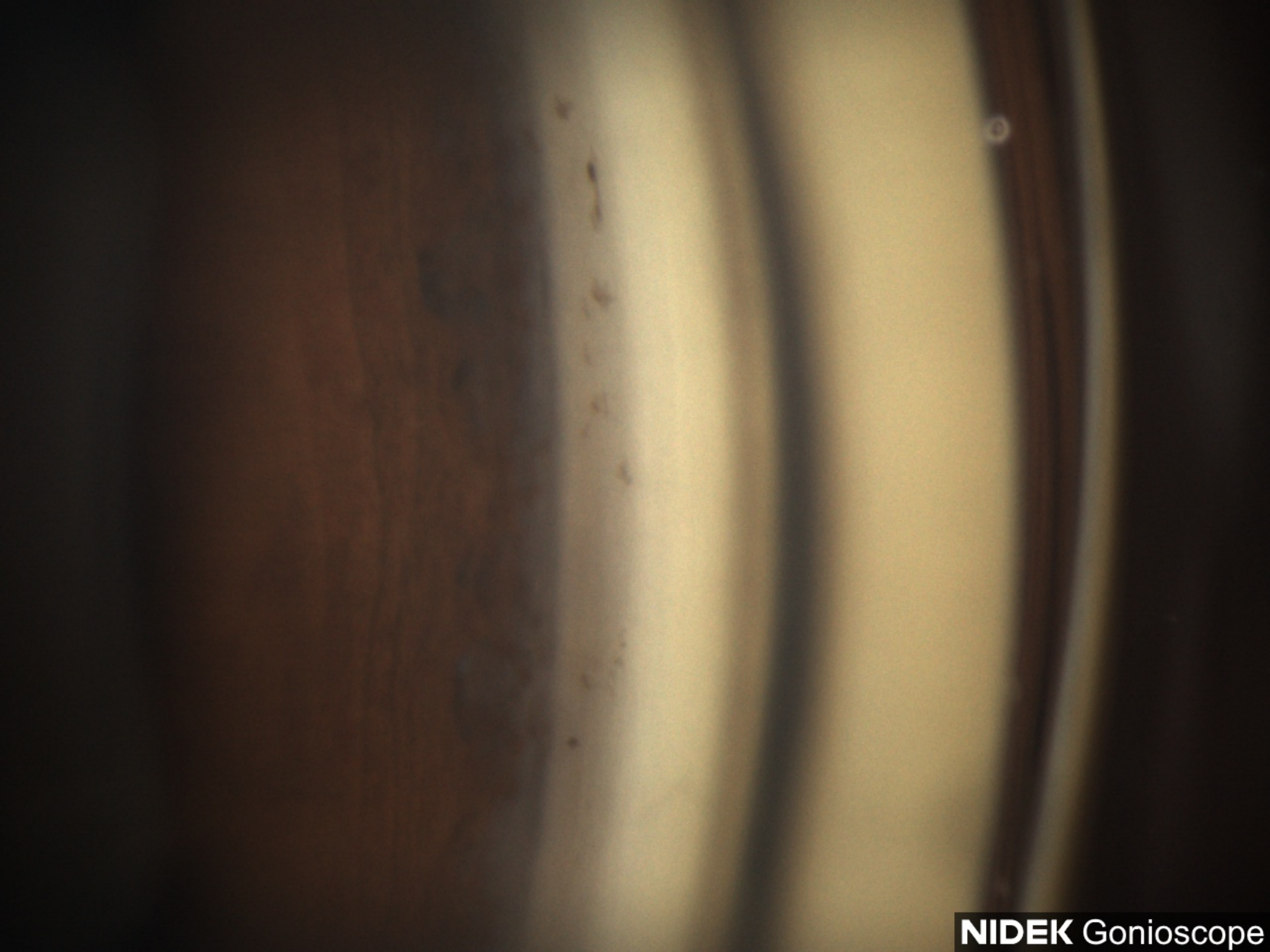

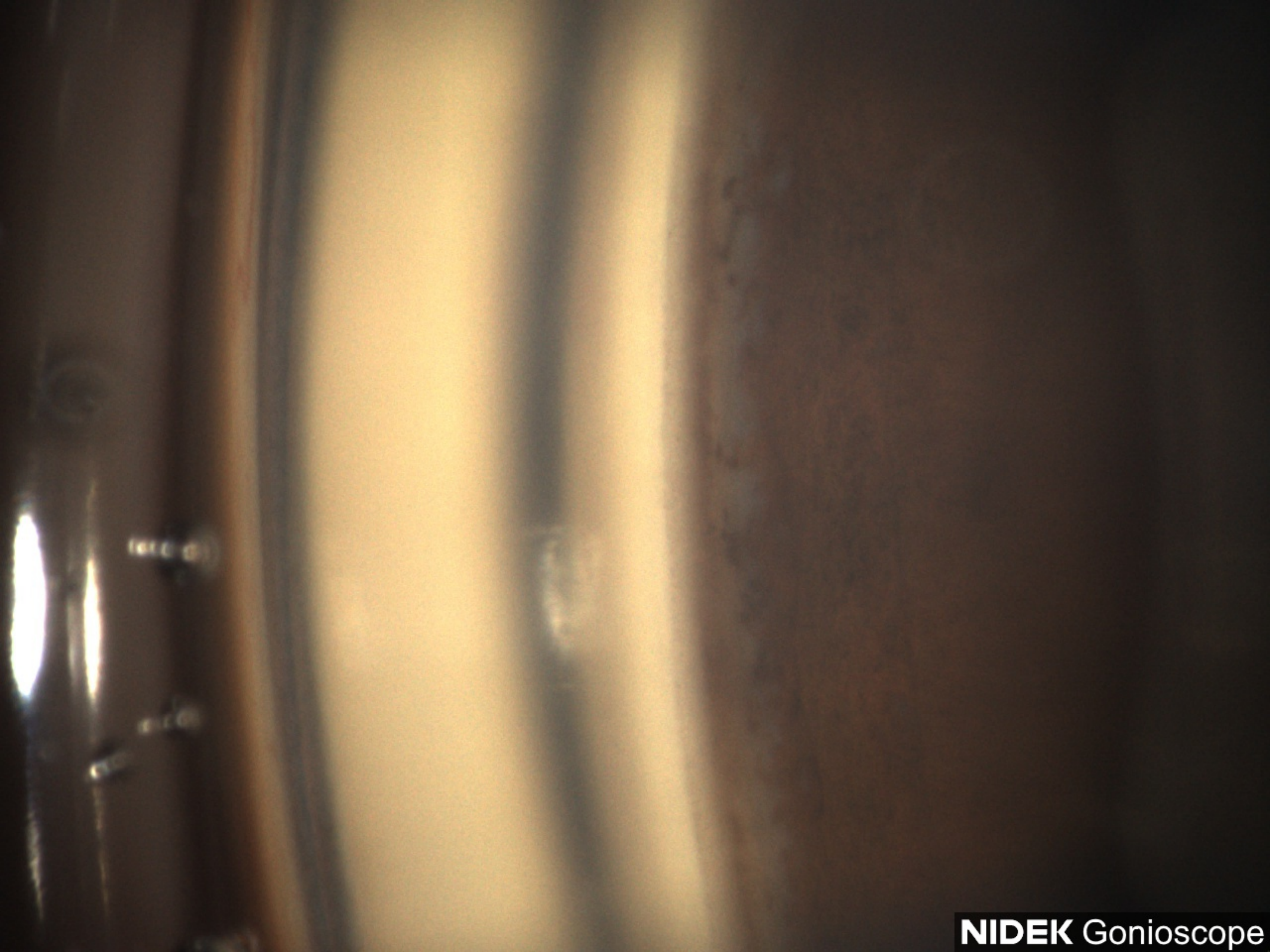

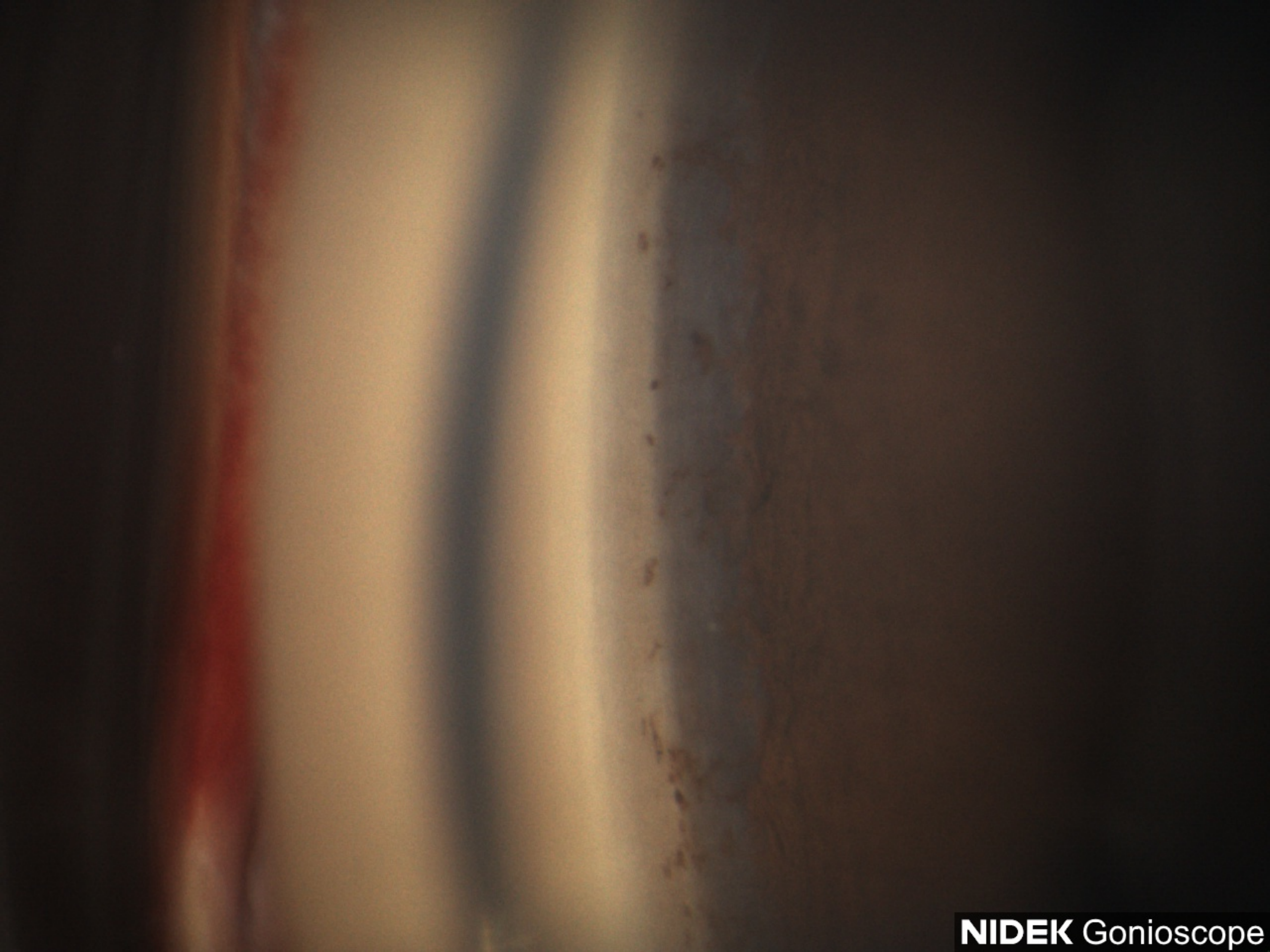

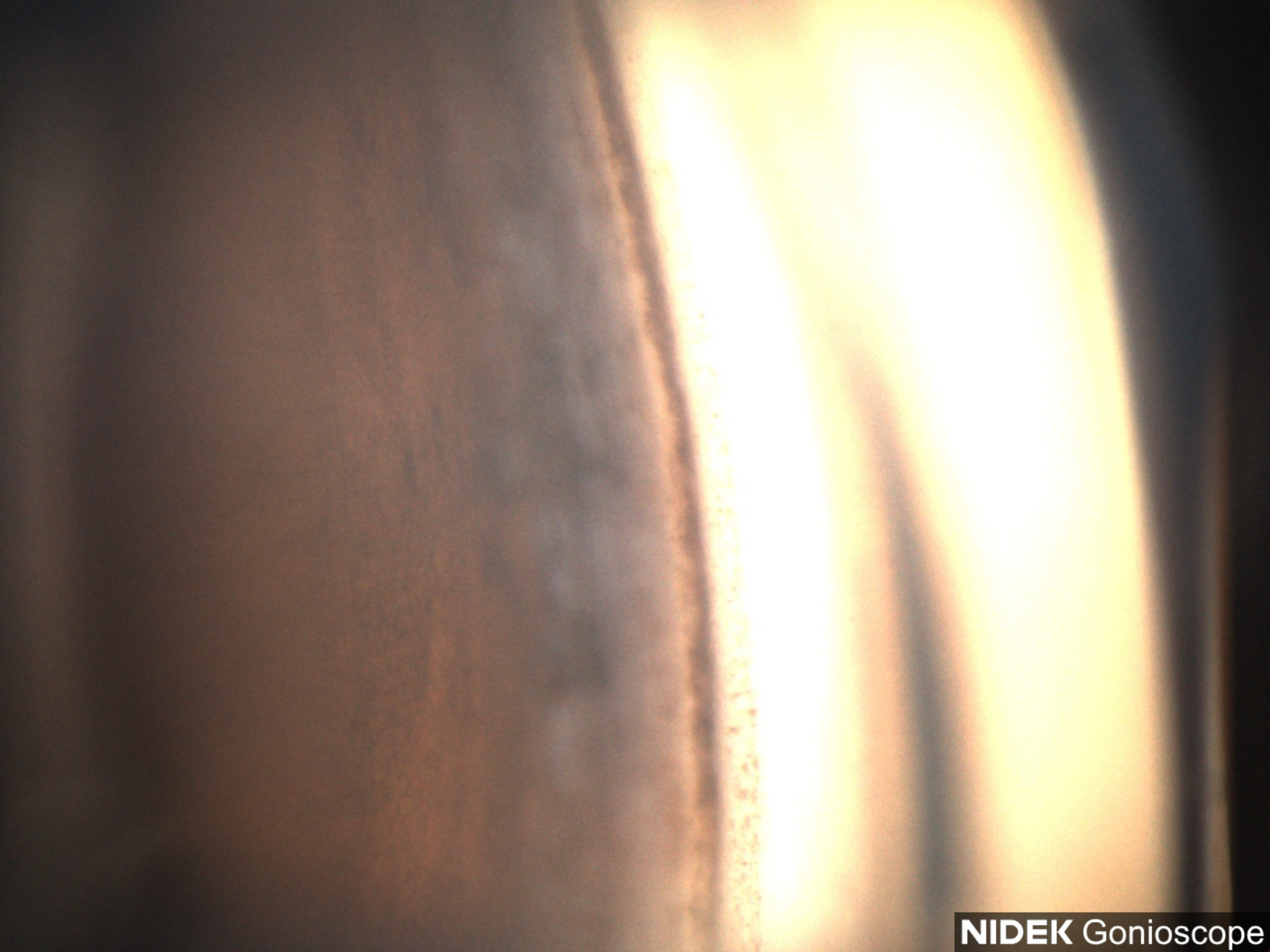

**NIDEK** Gonioscope

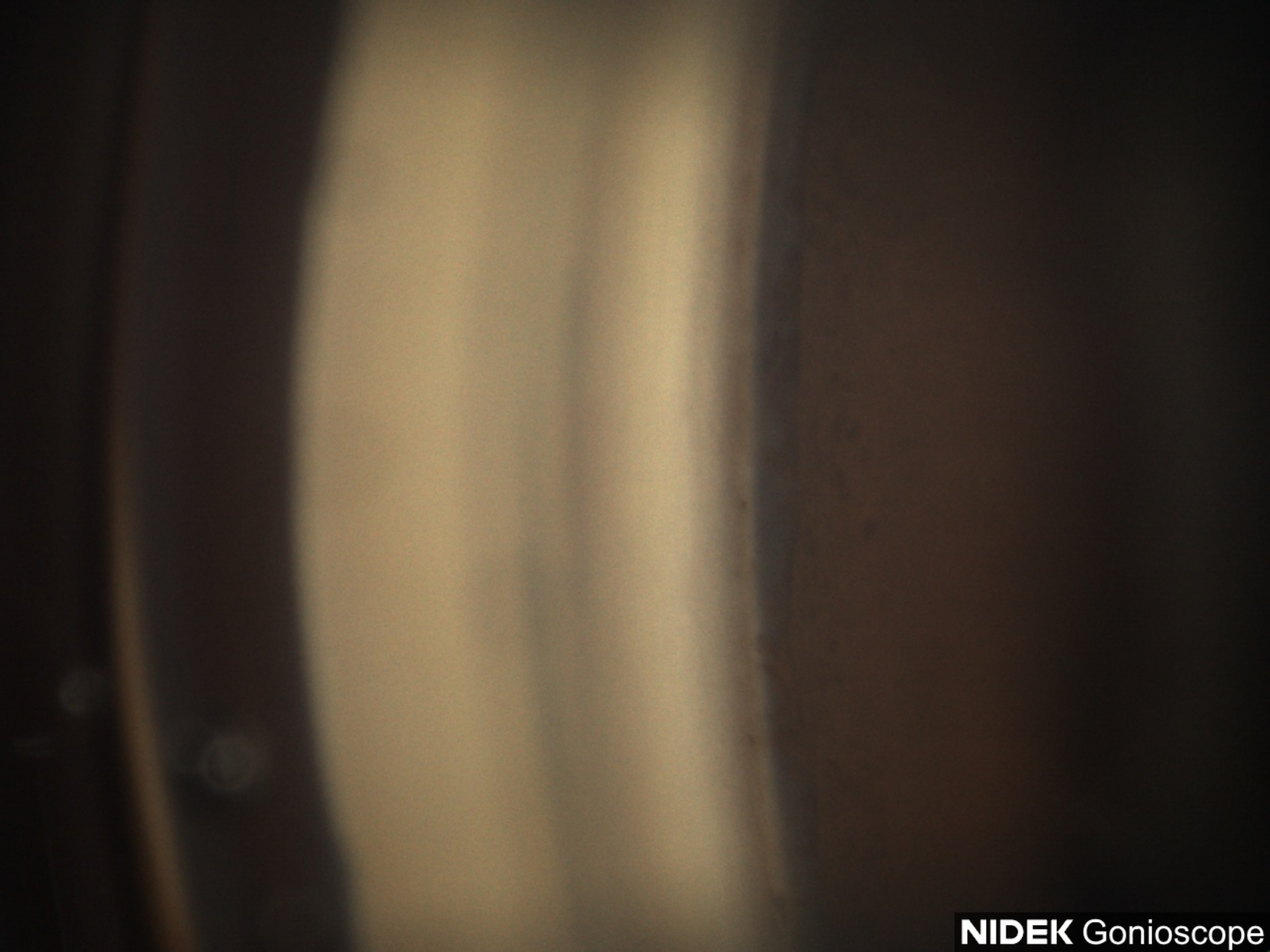

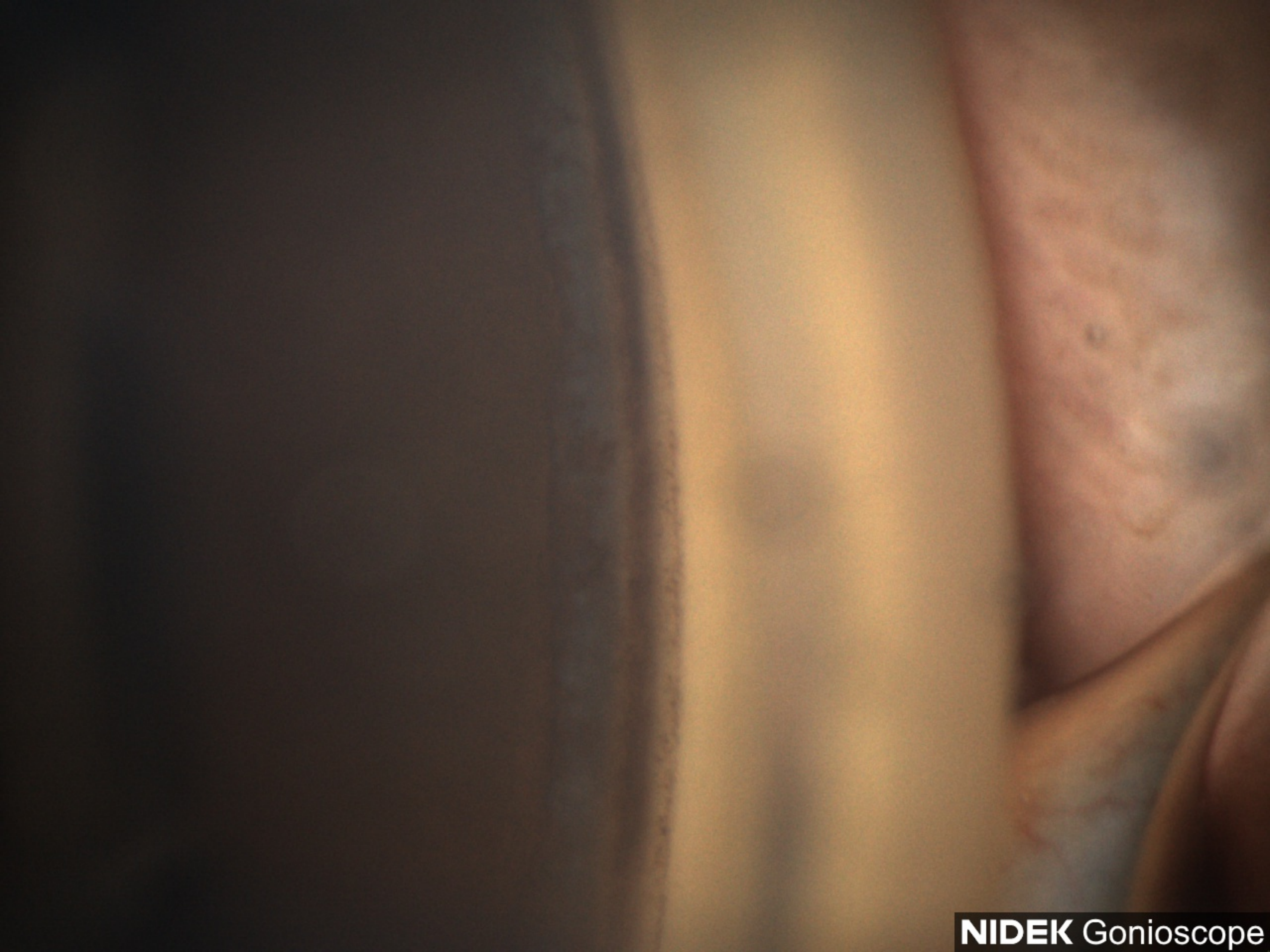

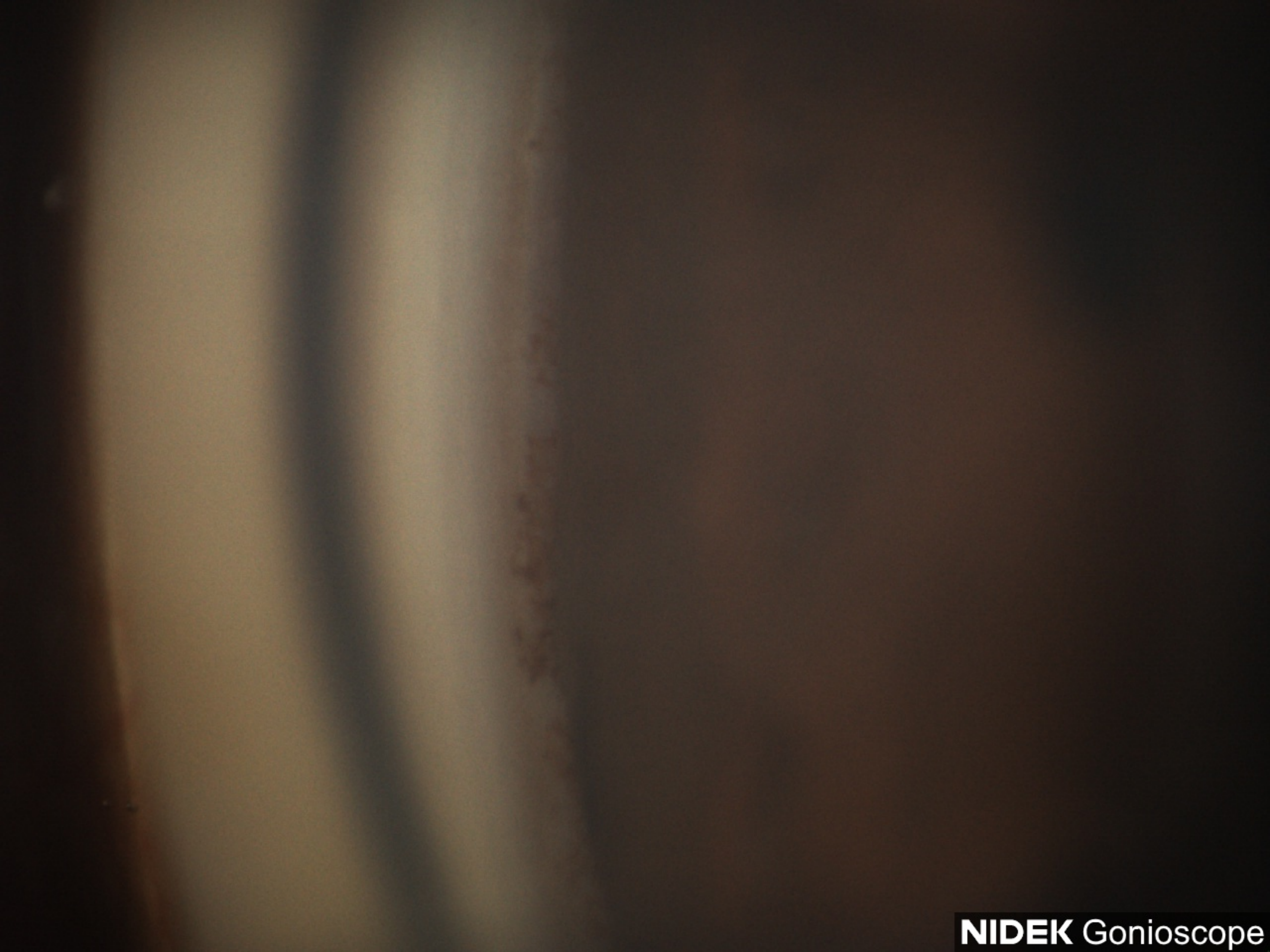

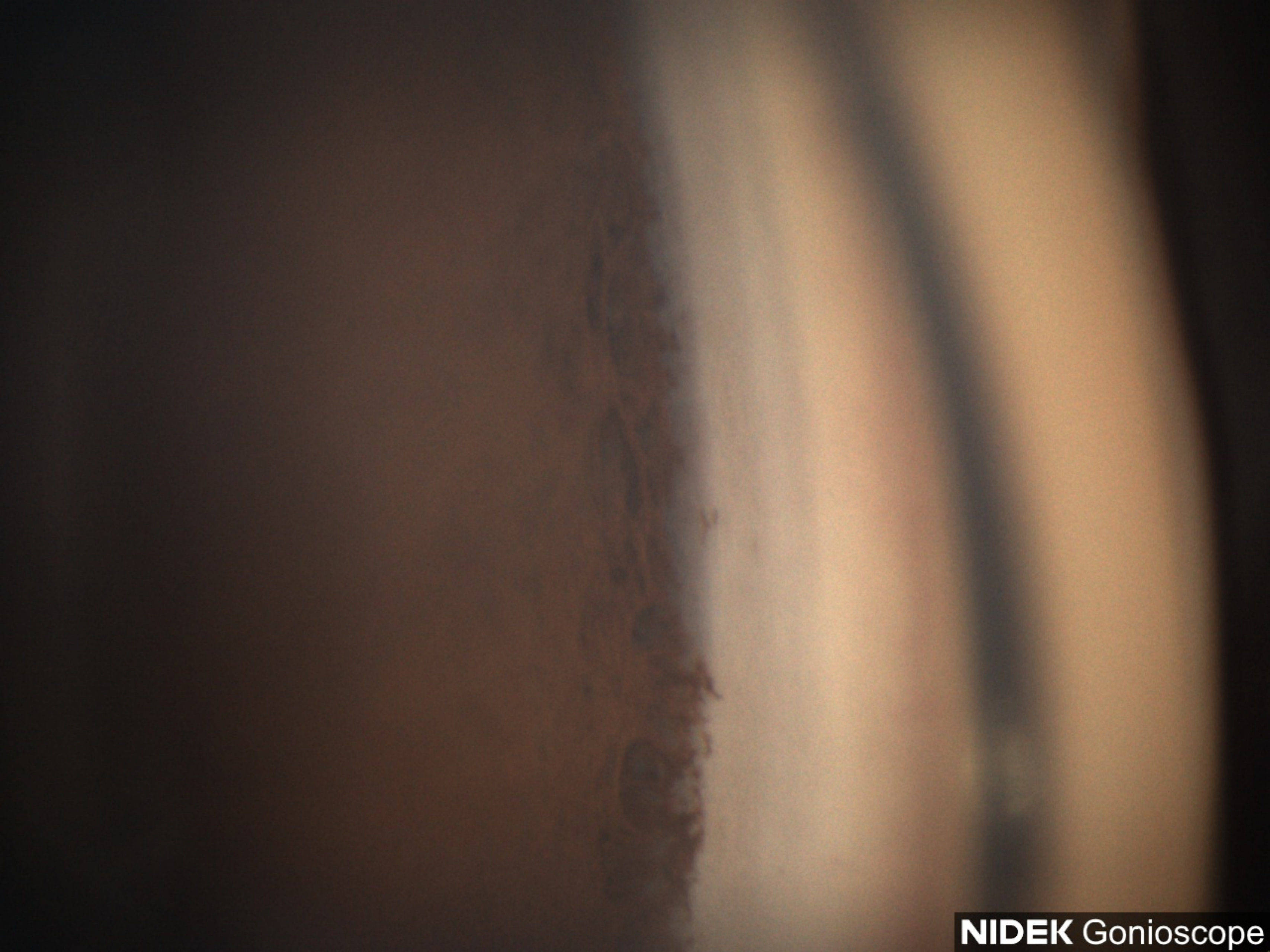

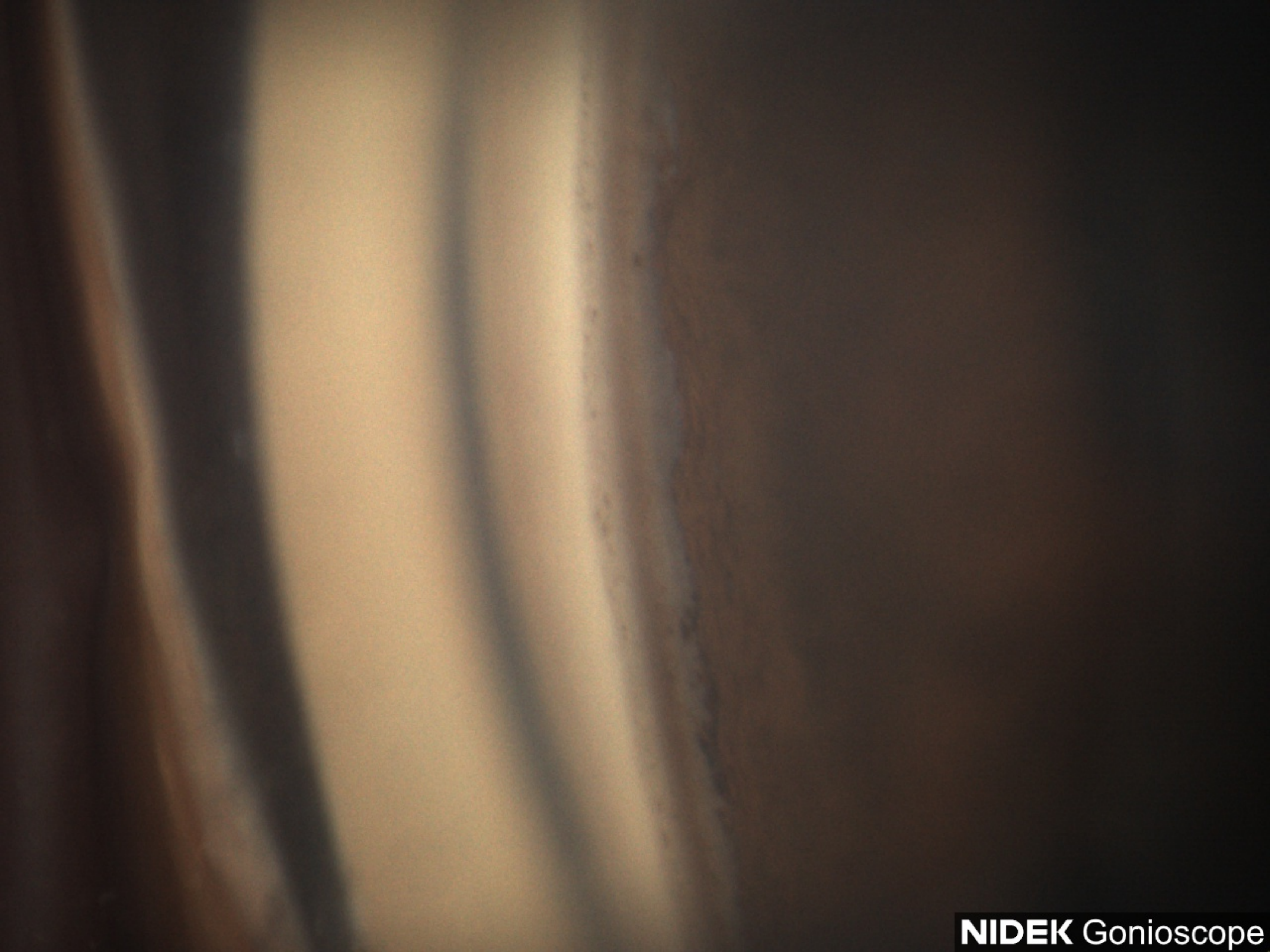

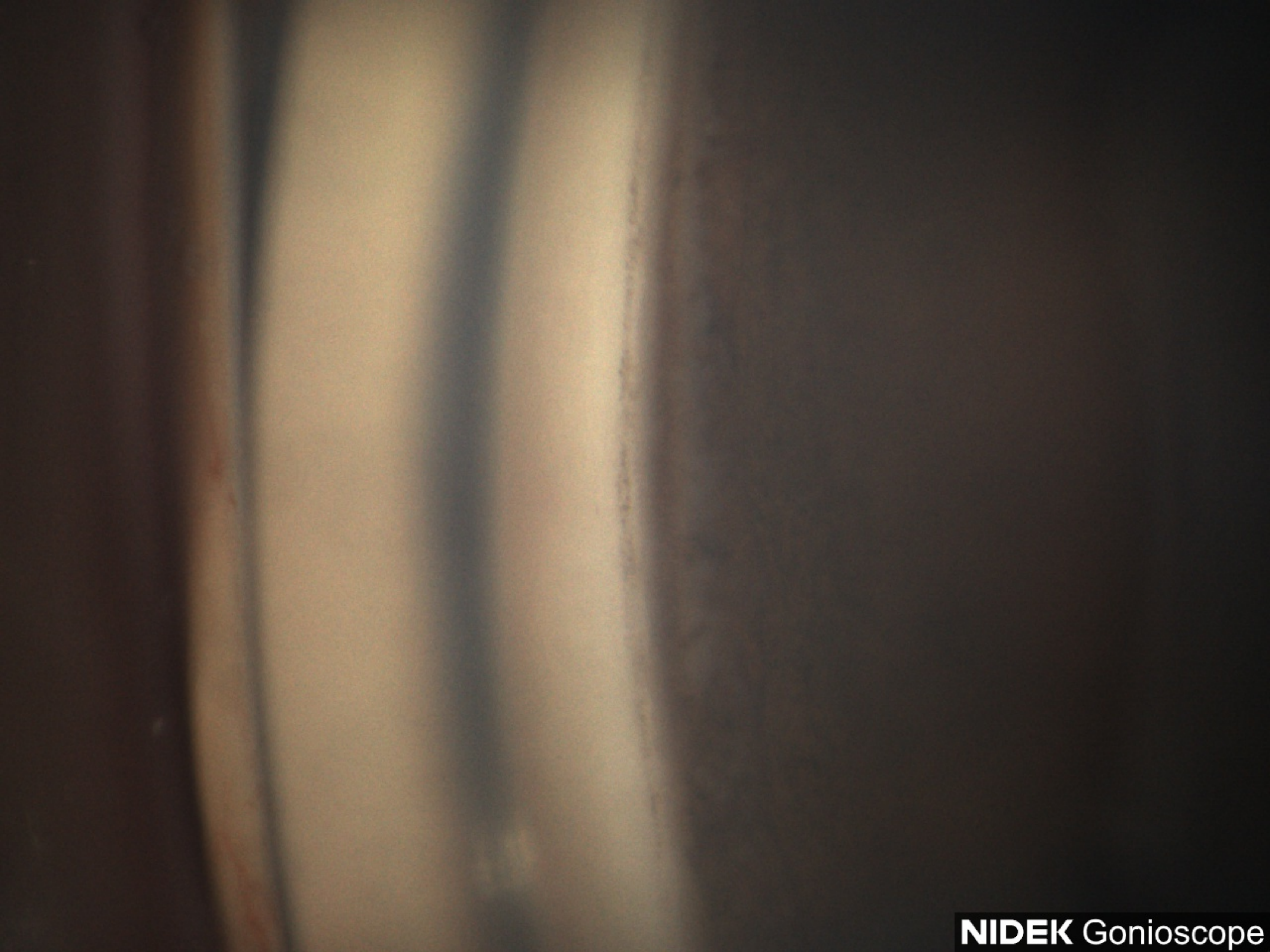

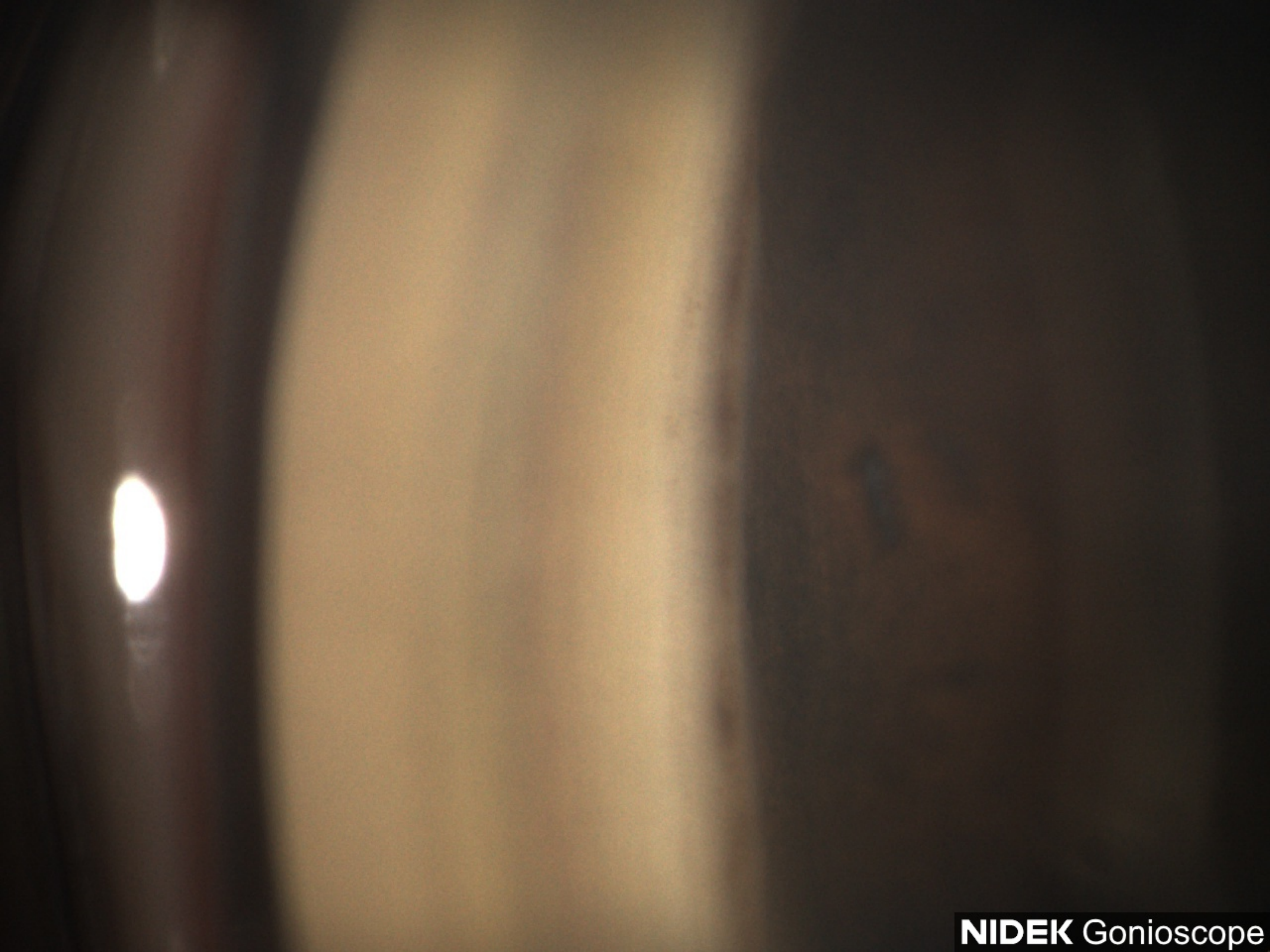

Supplement: S1 Data — (PDF) [file pone.0251249.s006.pdf]

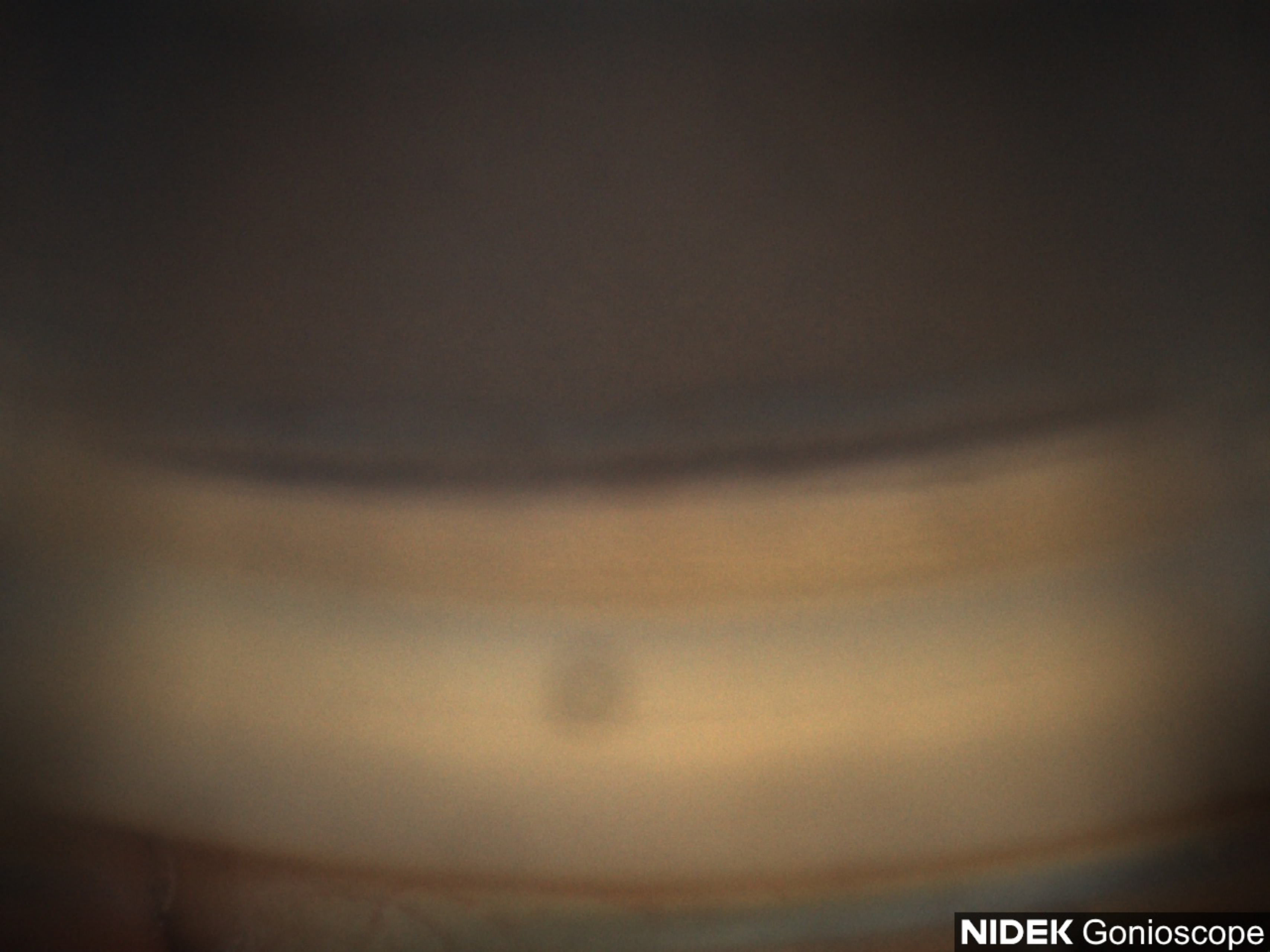

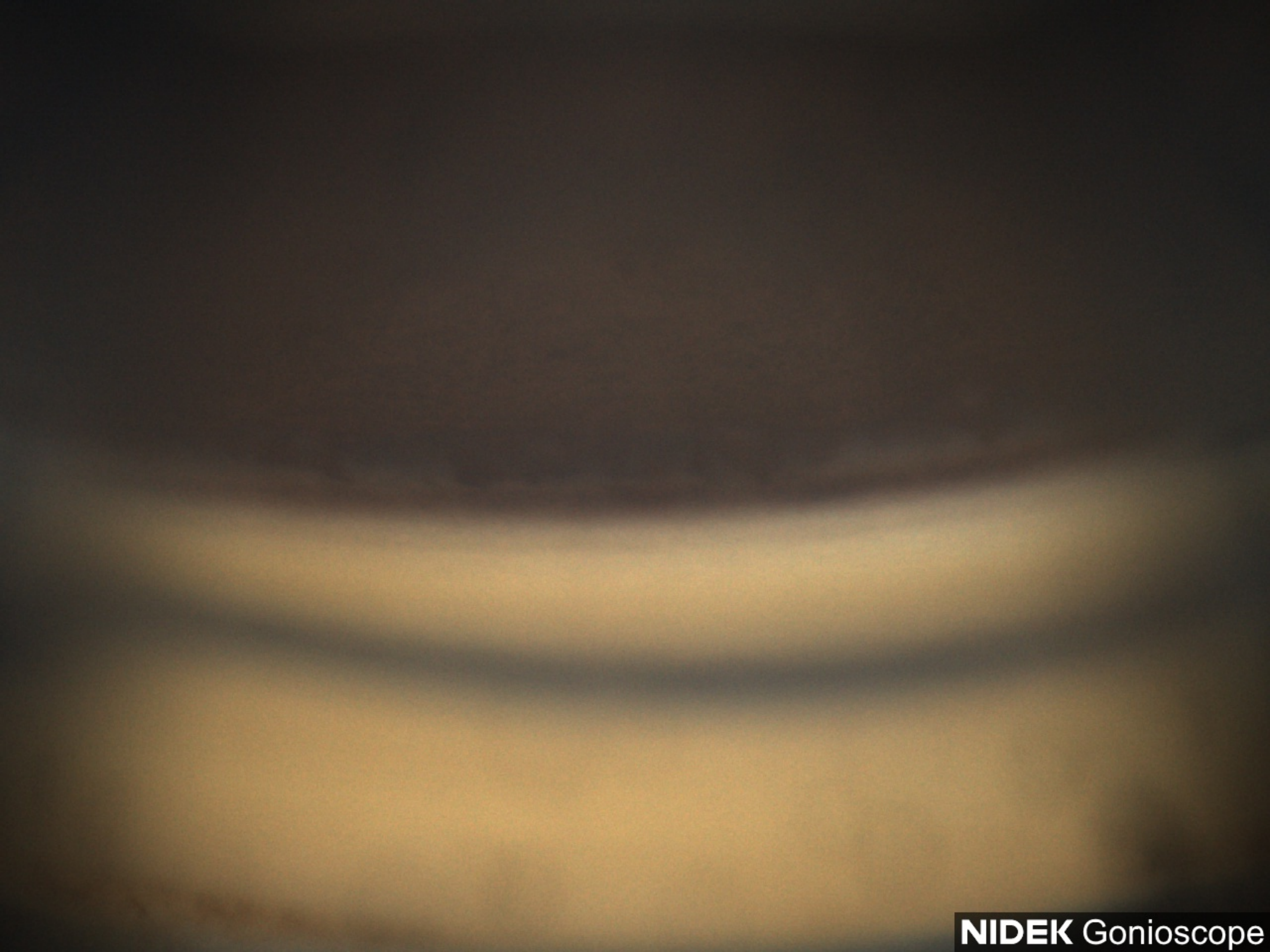

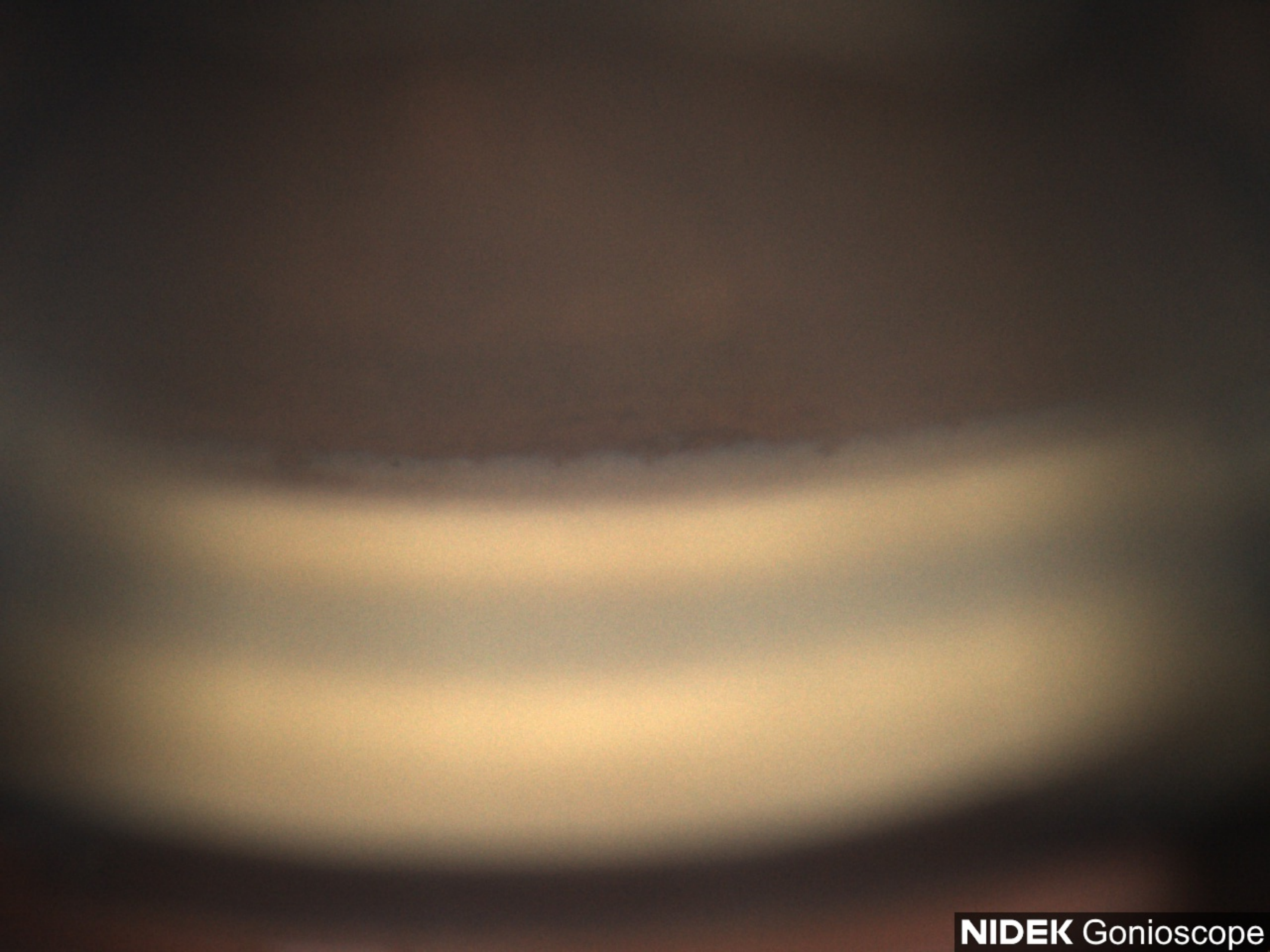

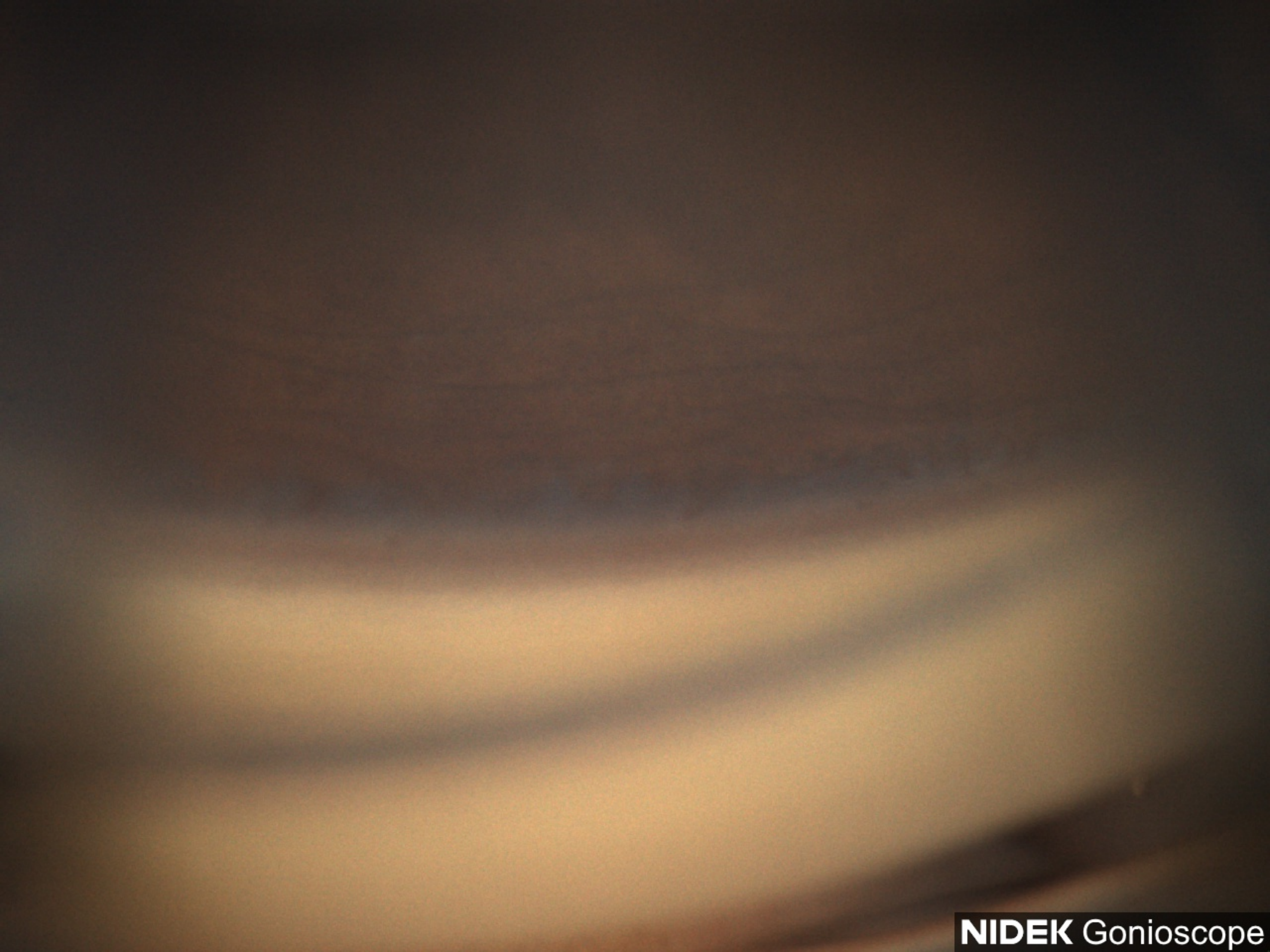

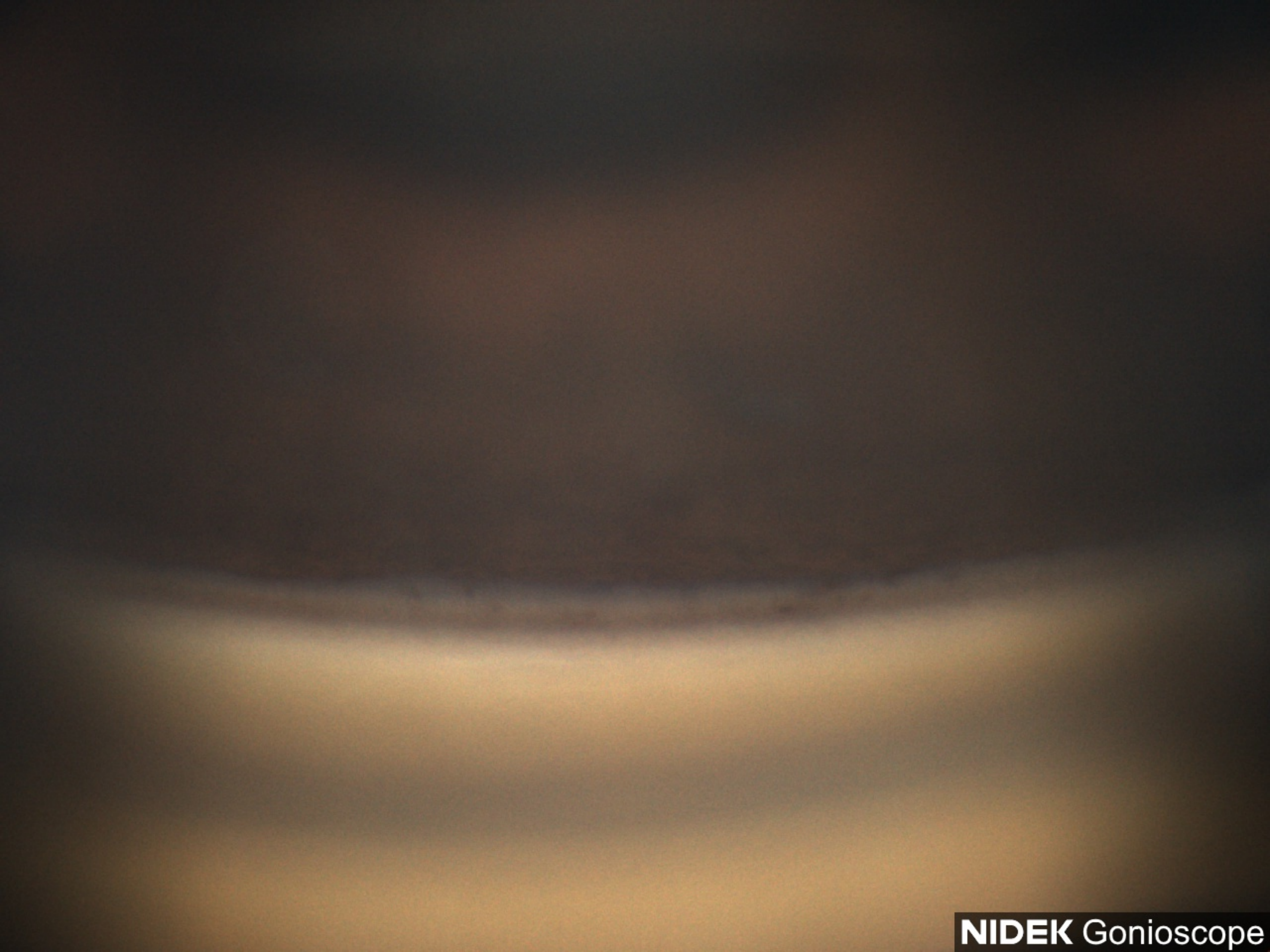

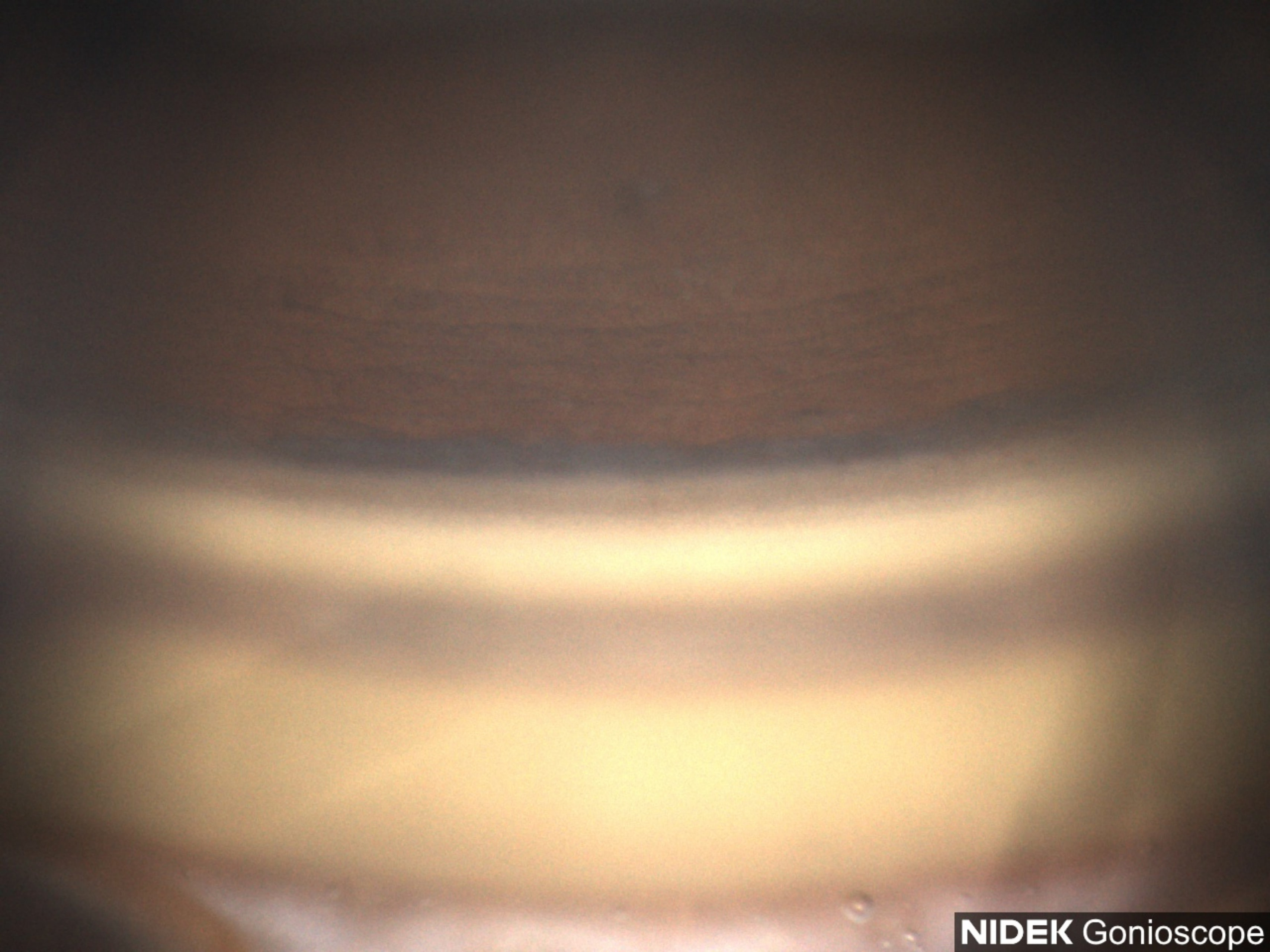

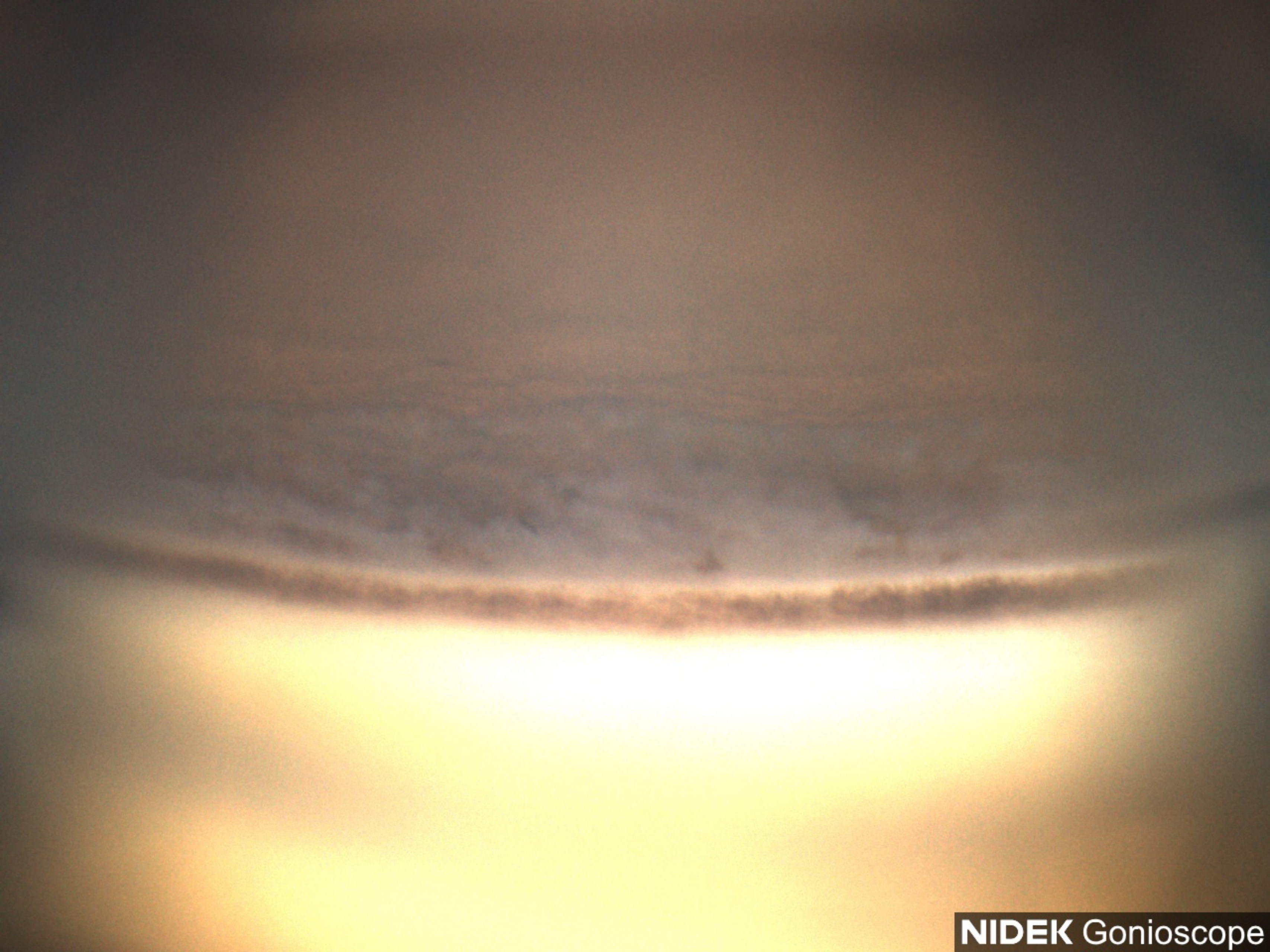

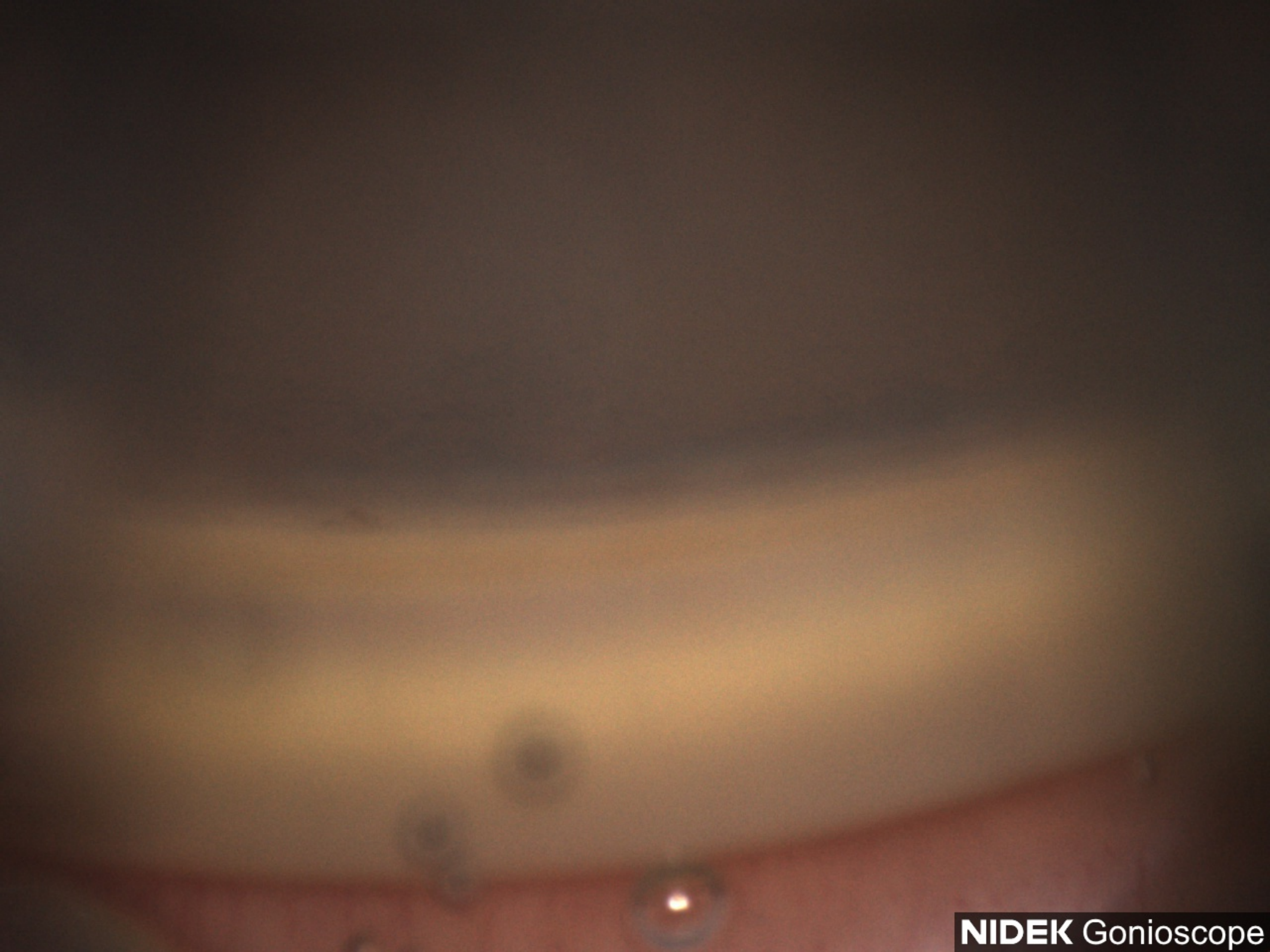

**NIDEK** Gonioscope

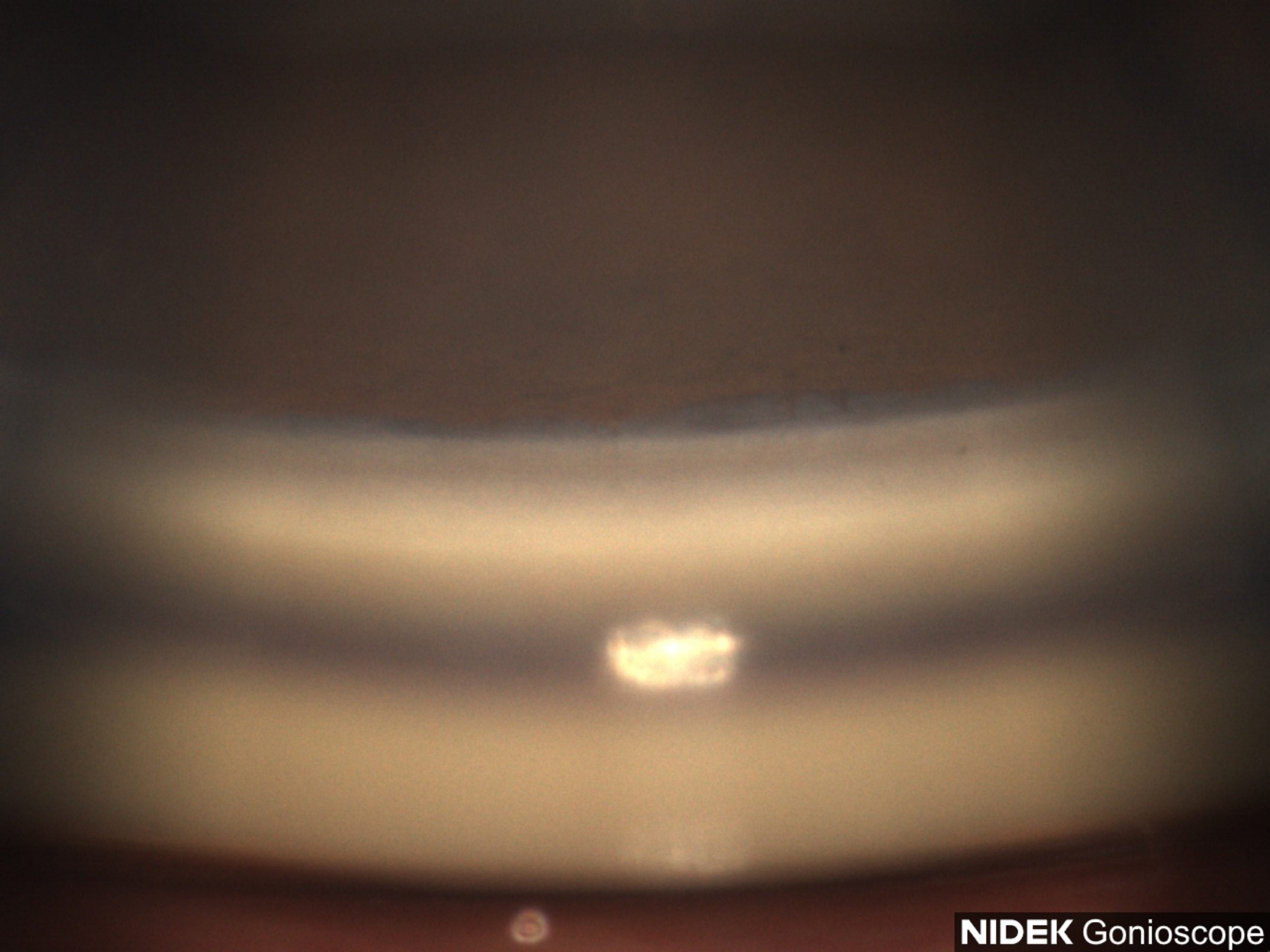

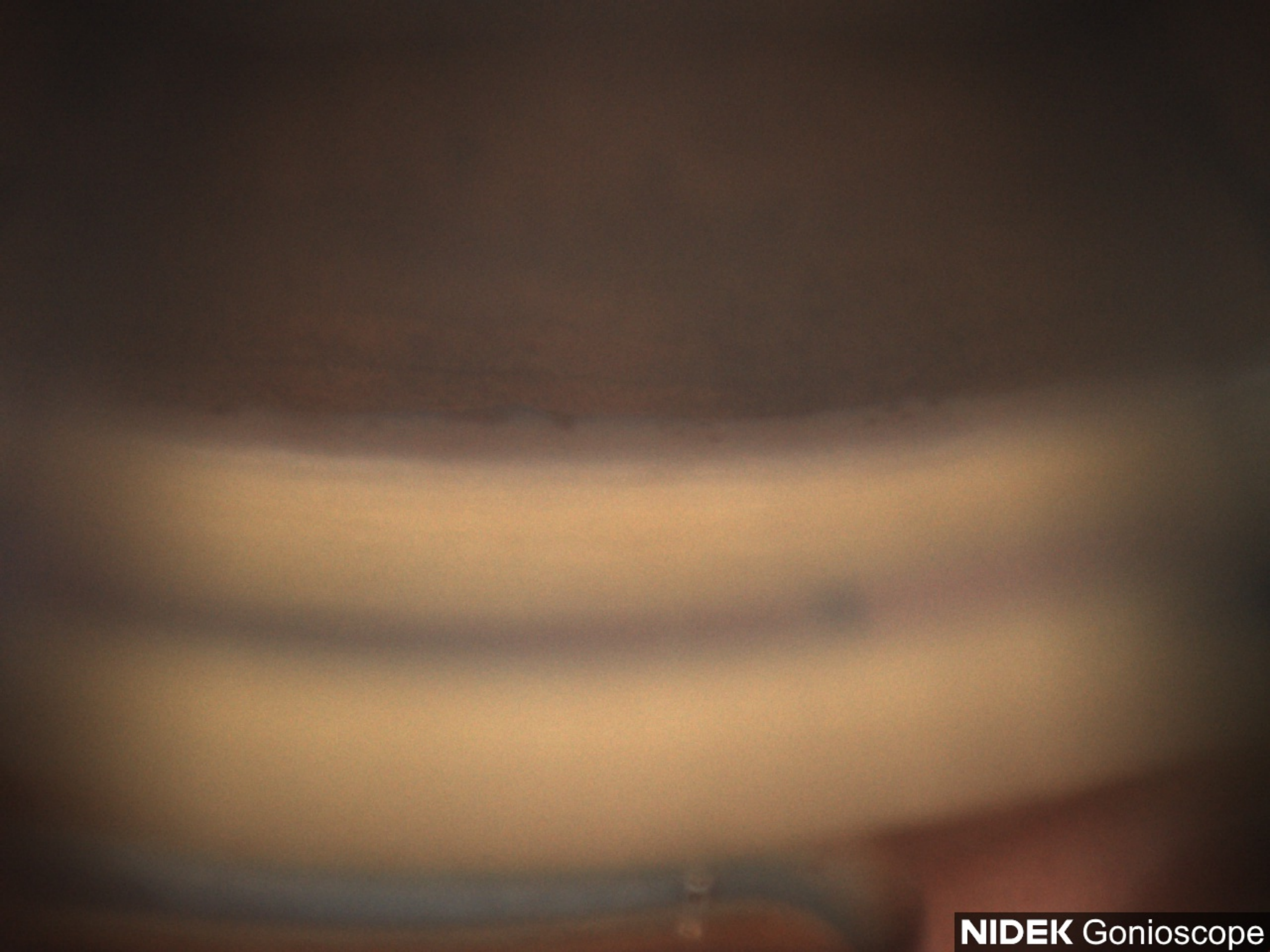

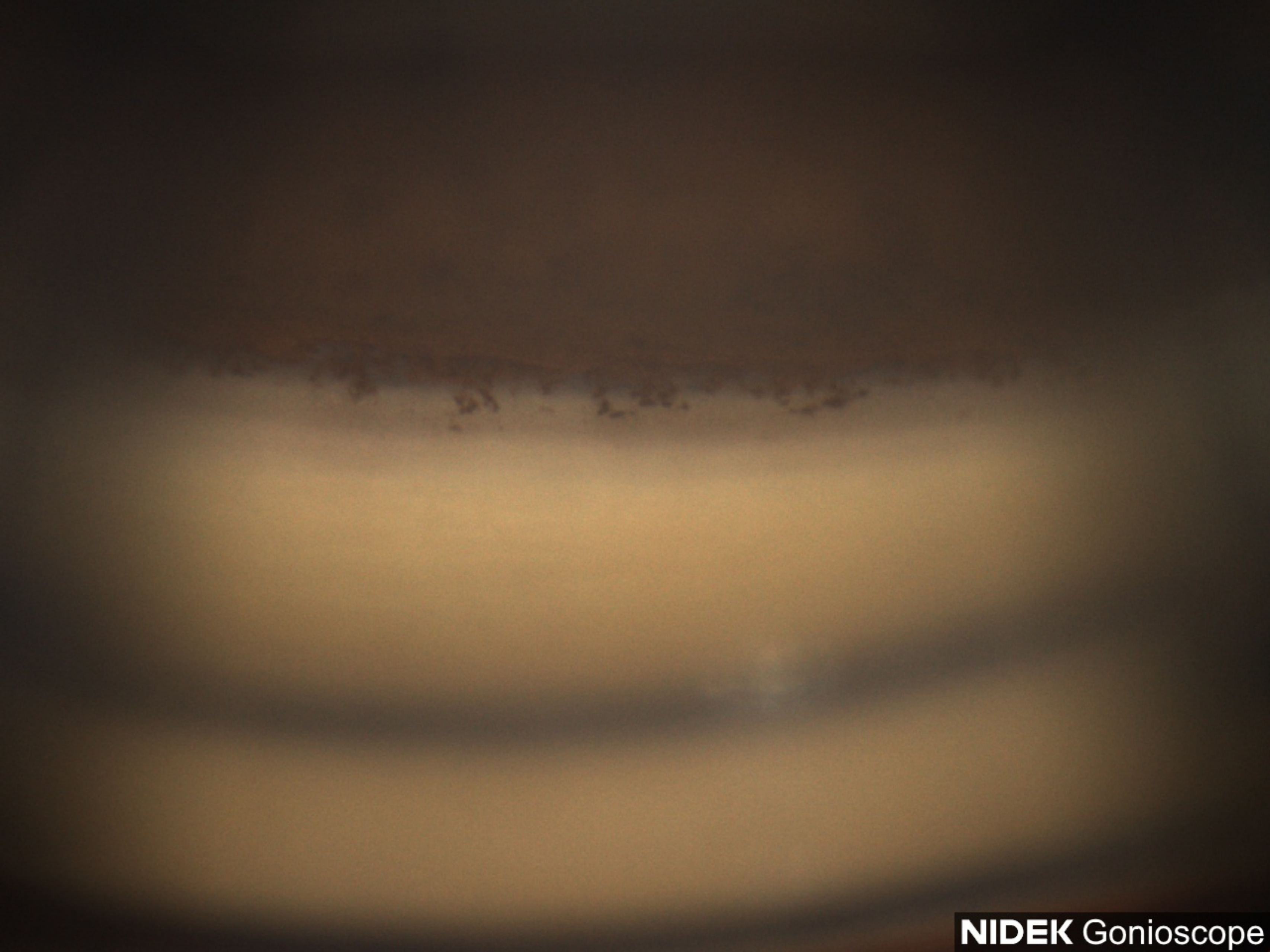

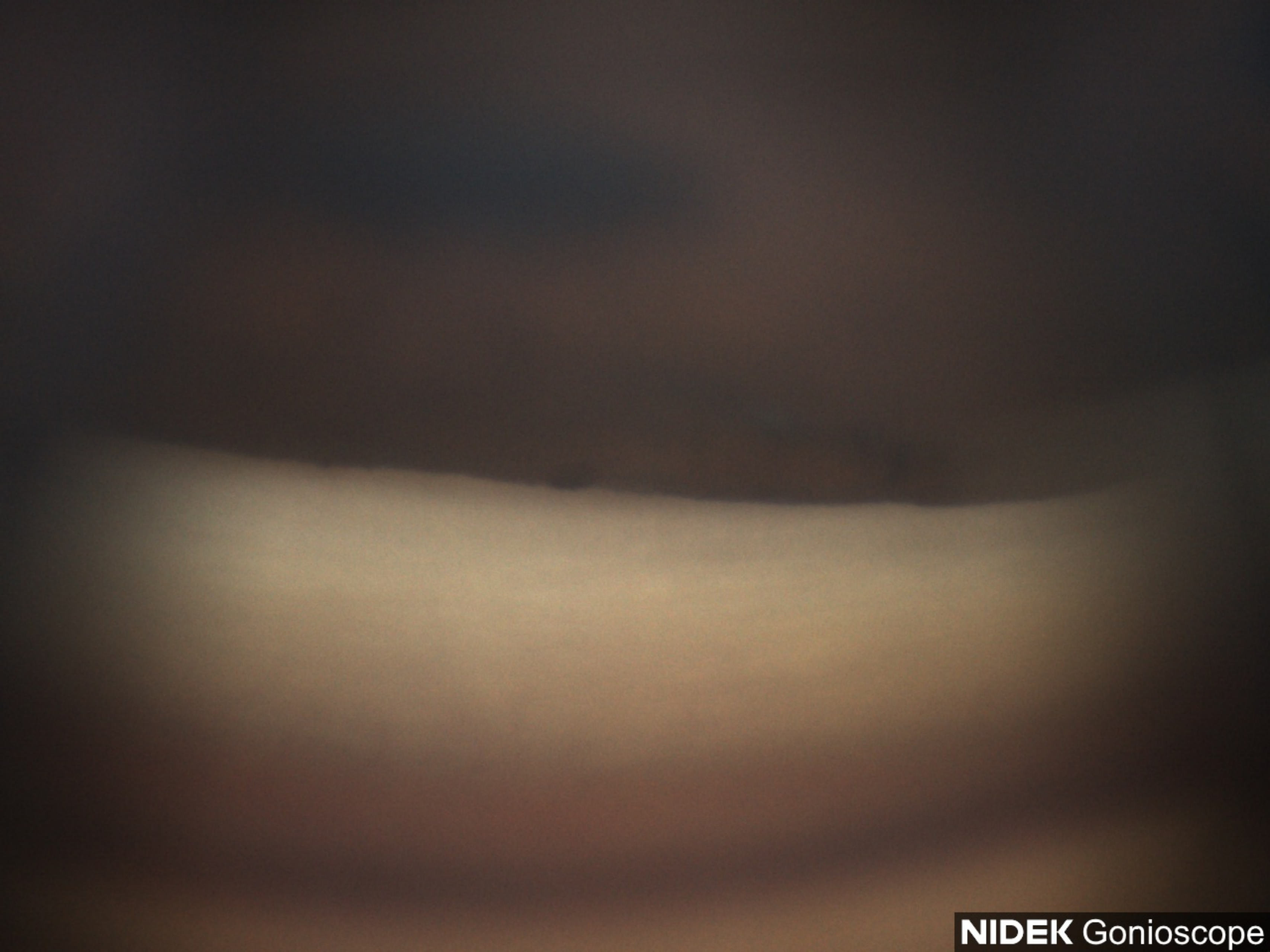

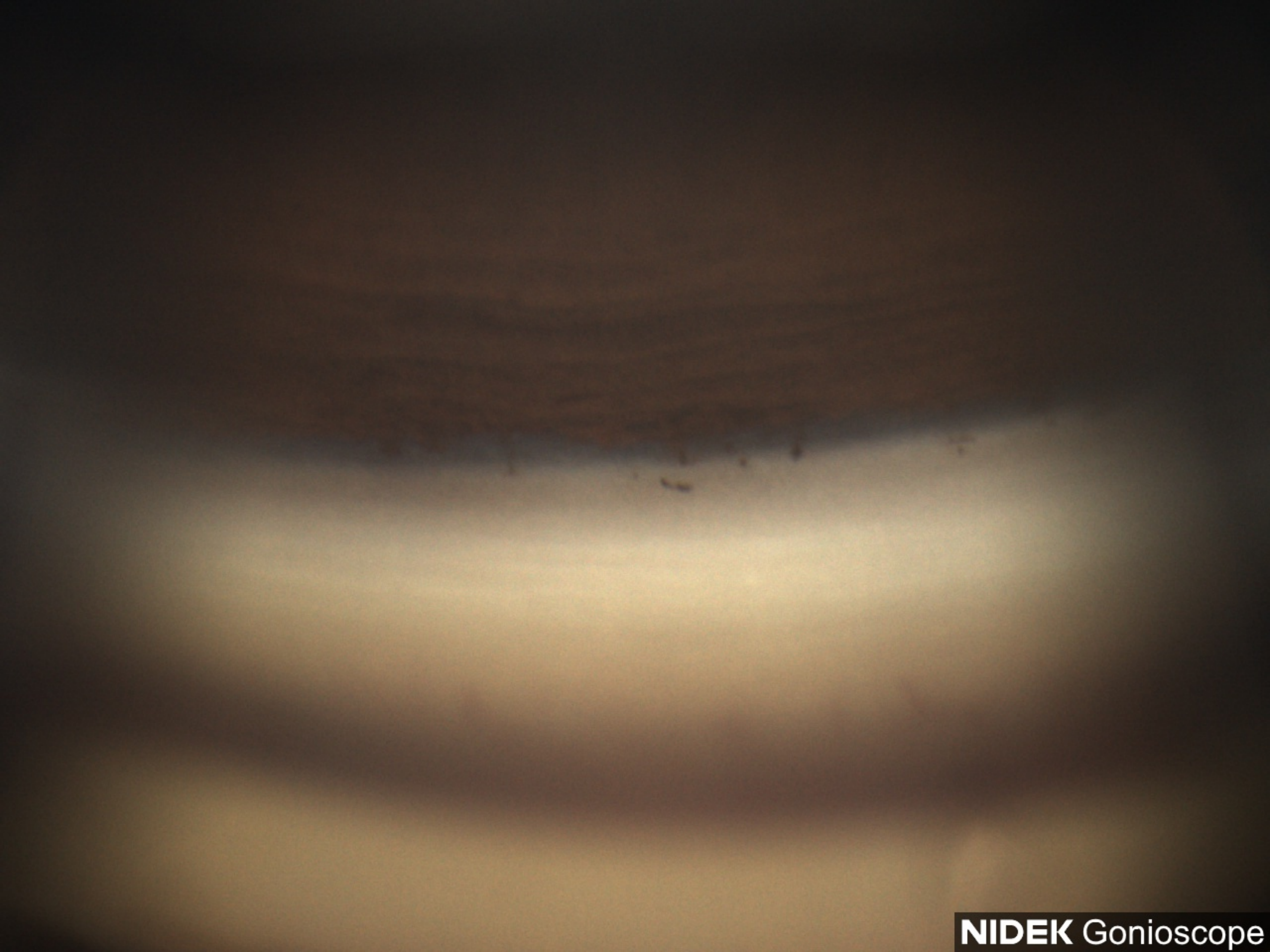

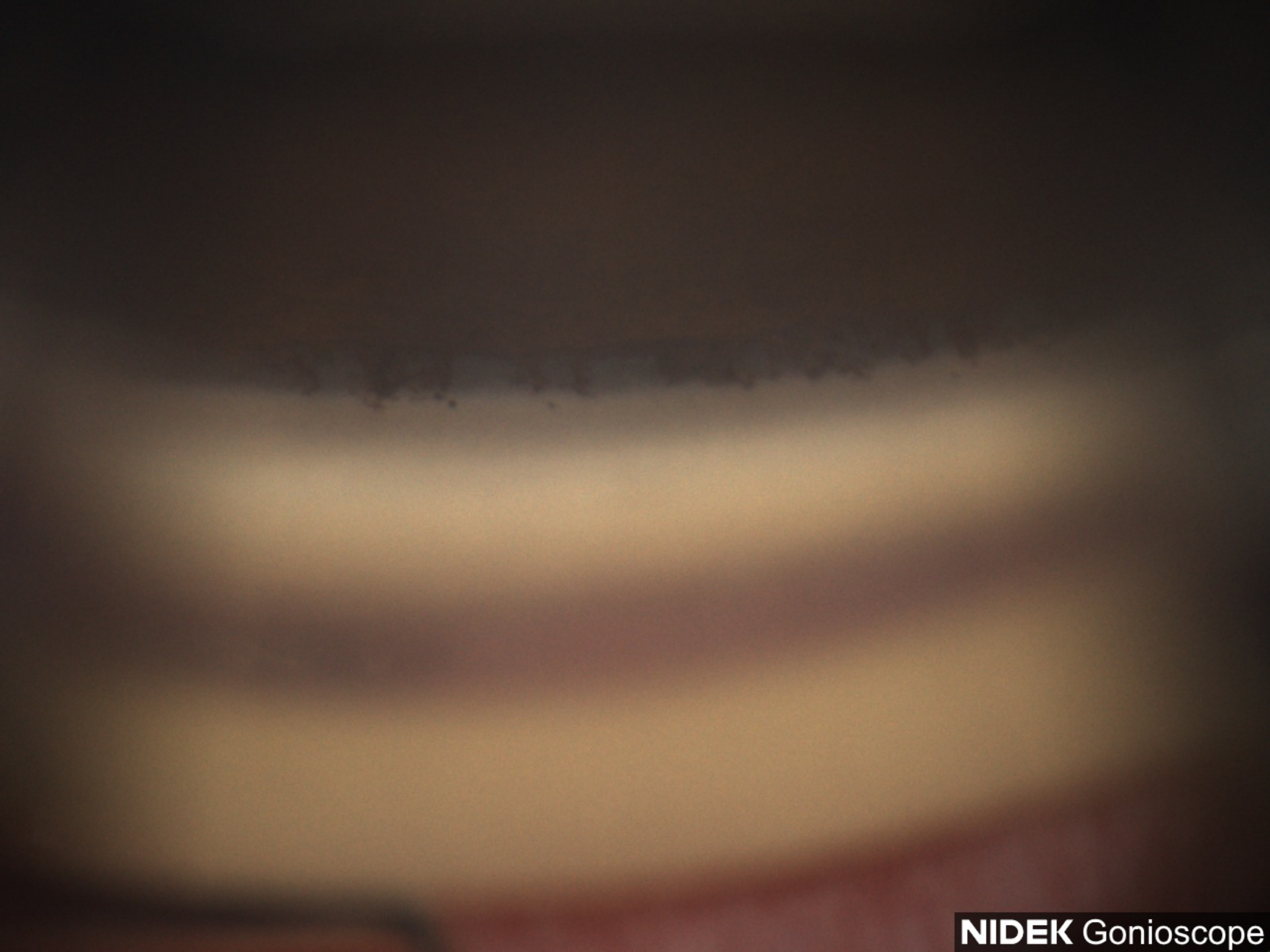

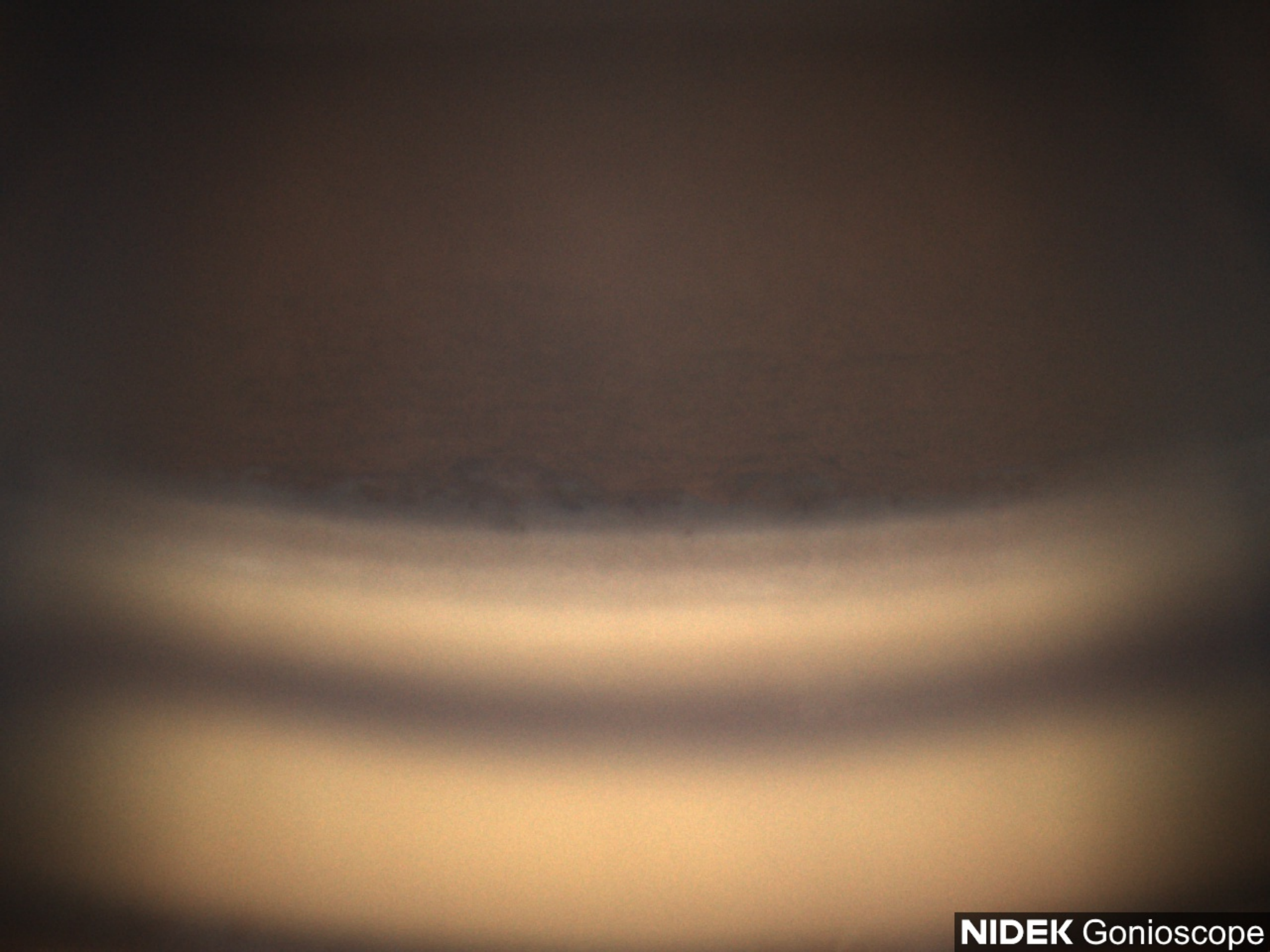

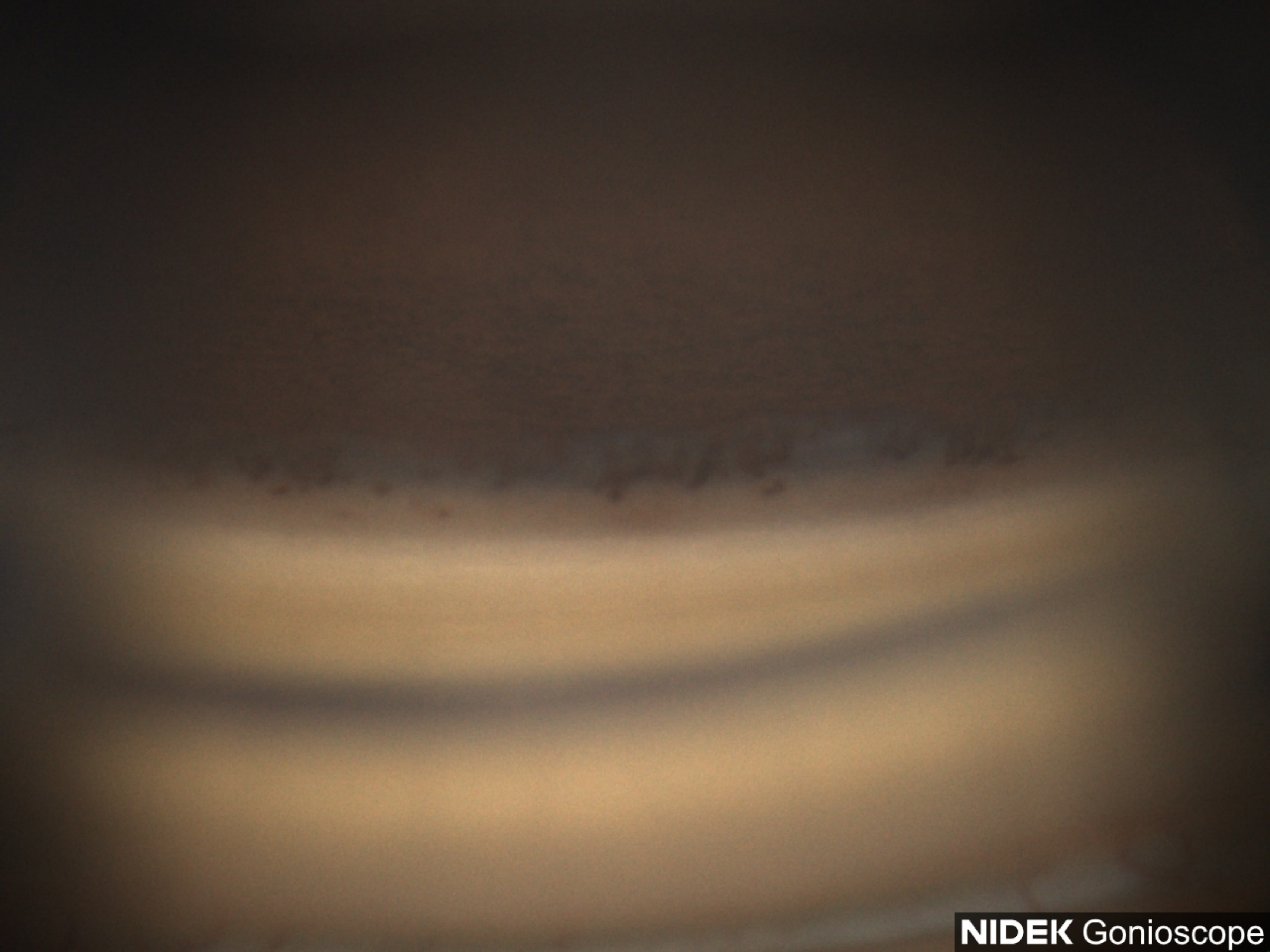

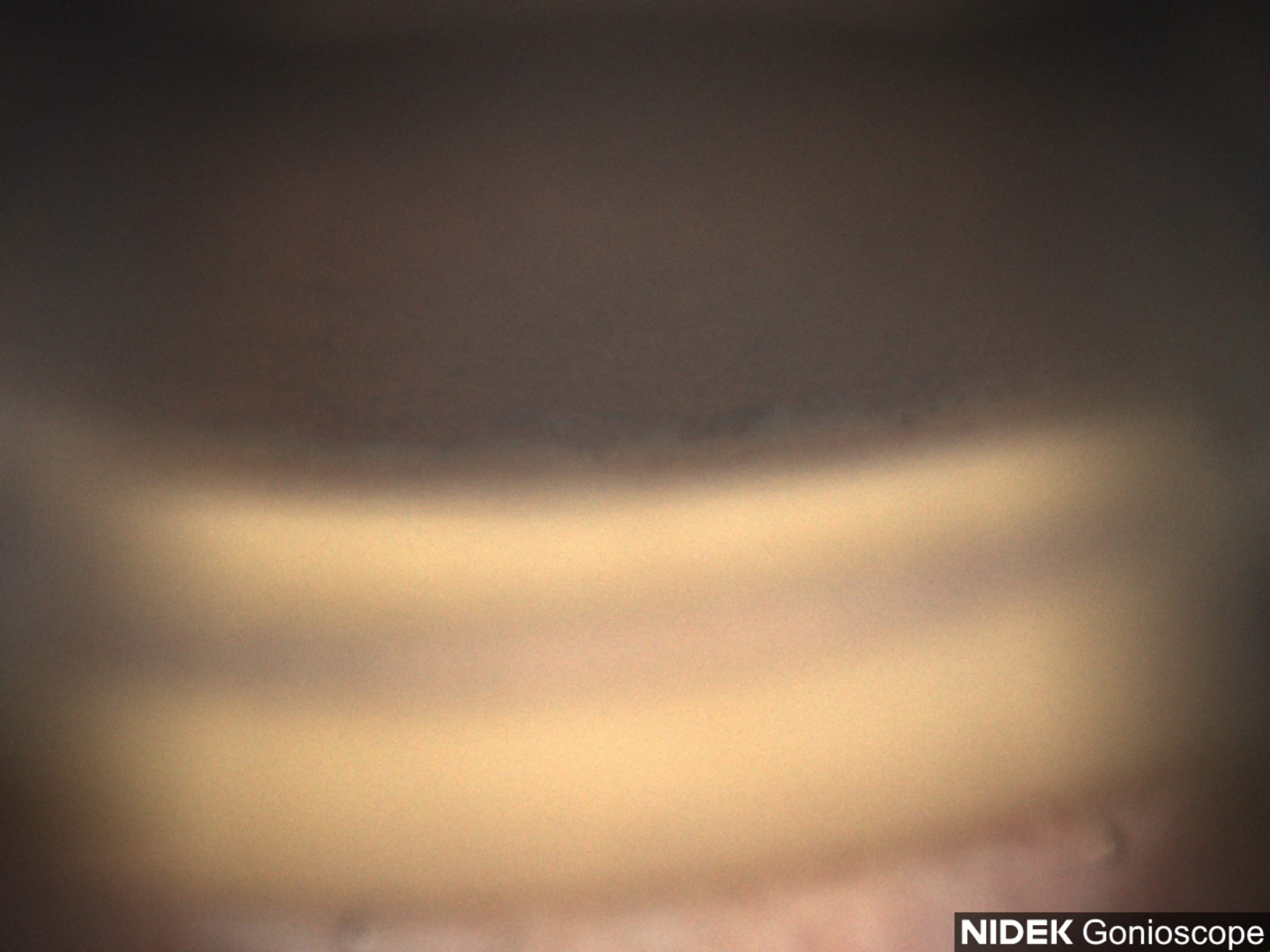

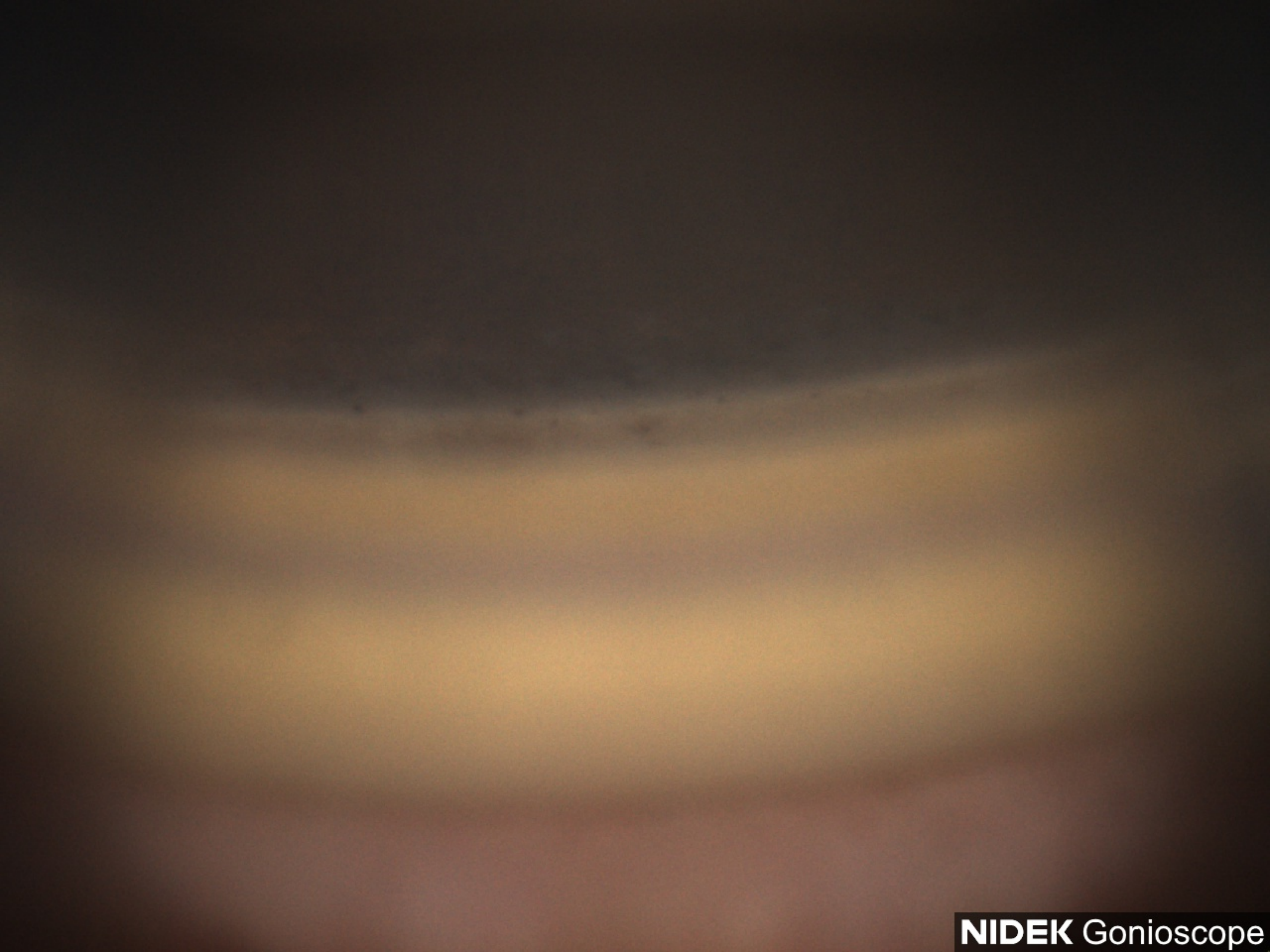

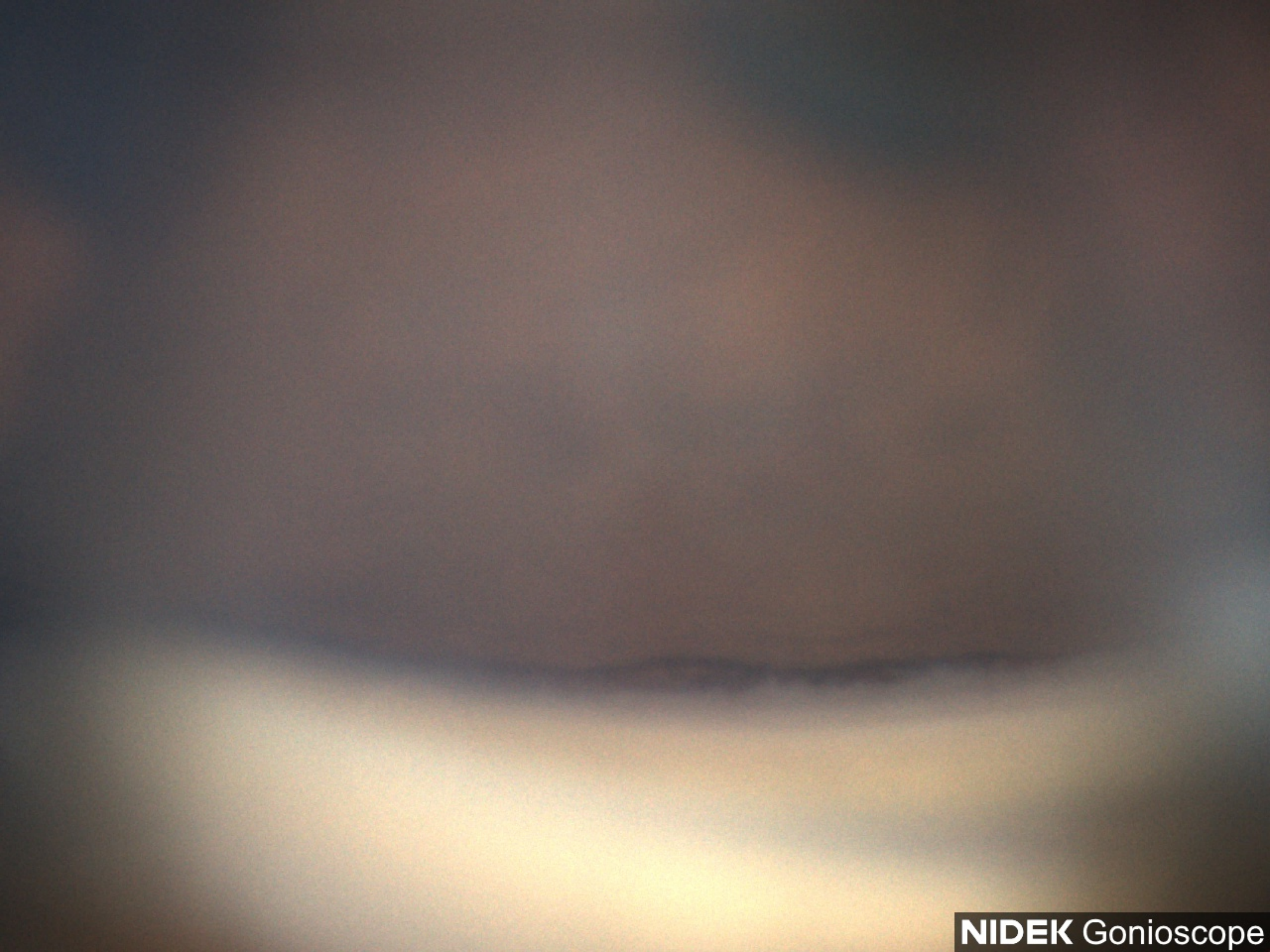

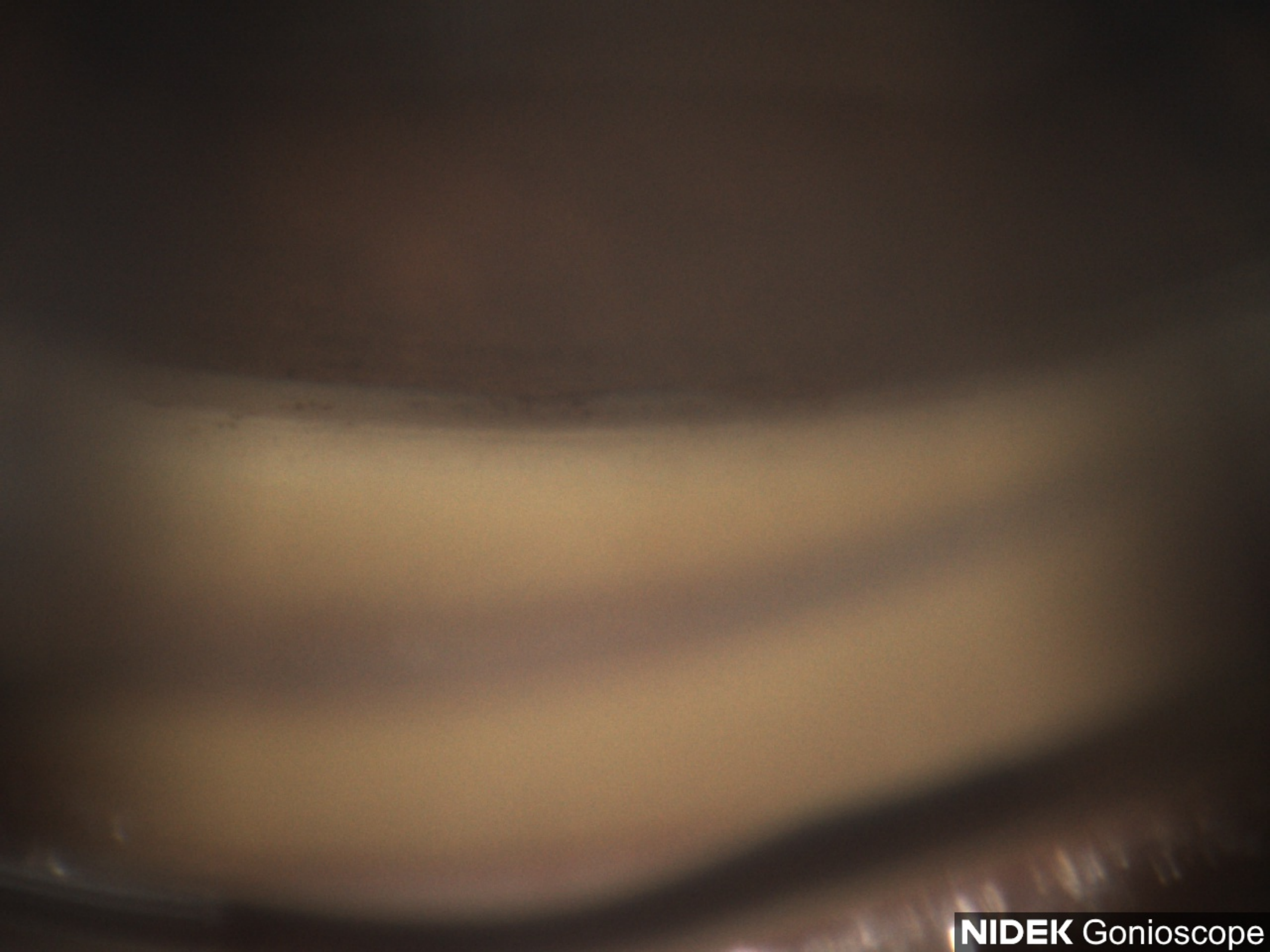

**NIDEK** Gonioscope

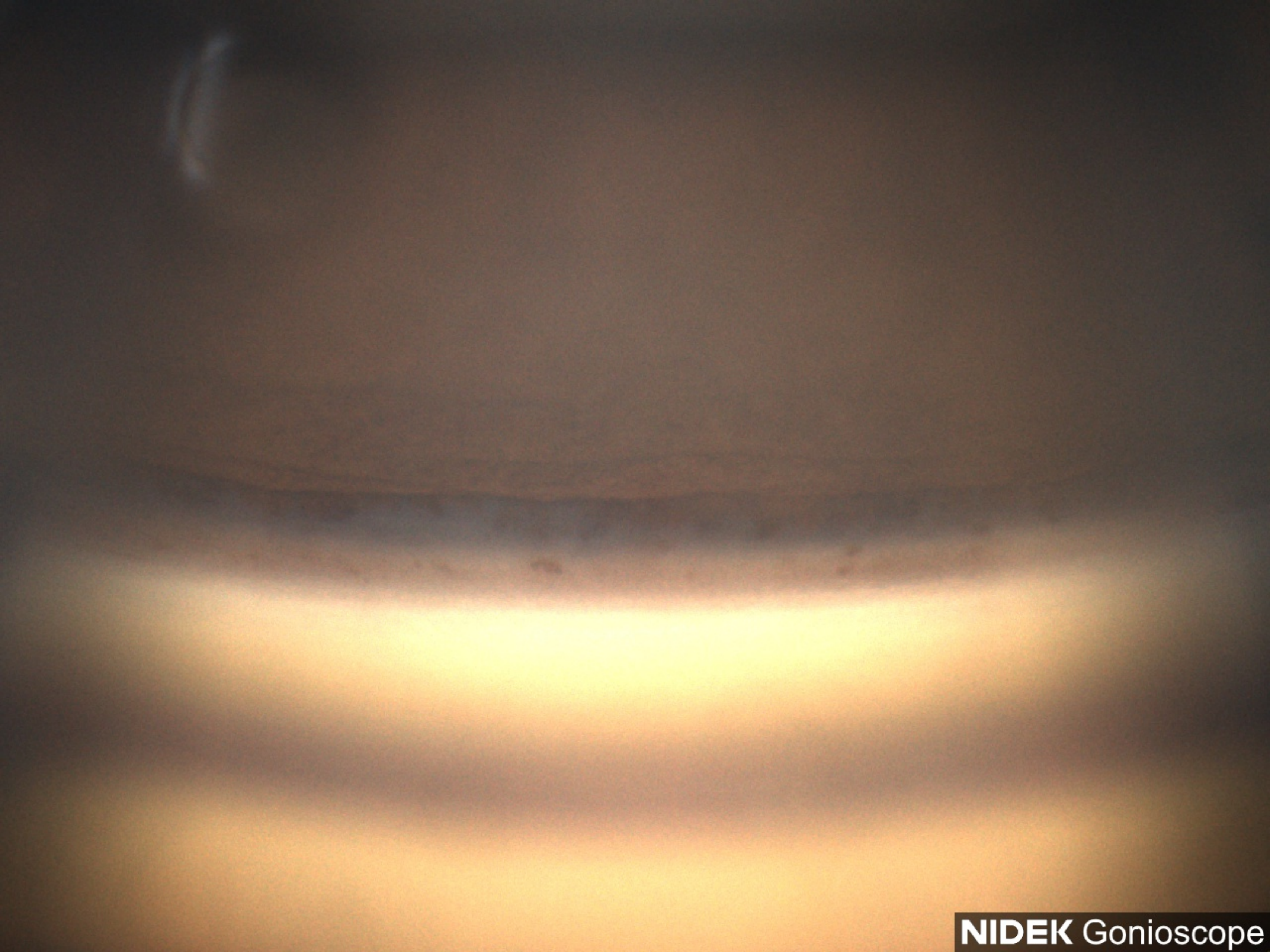

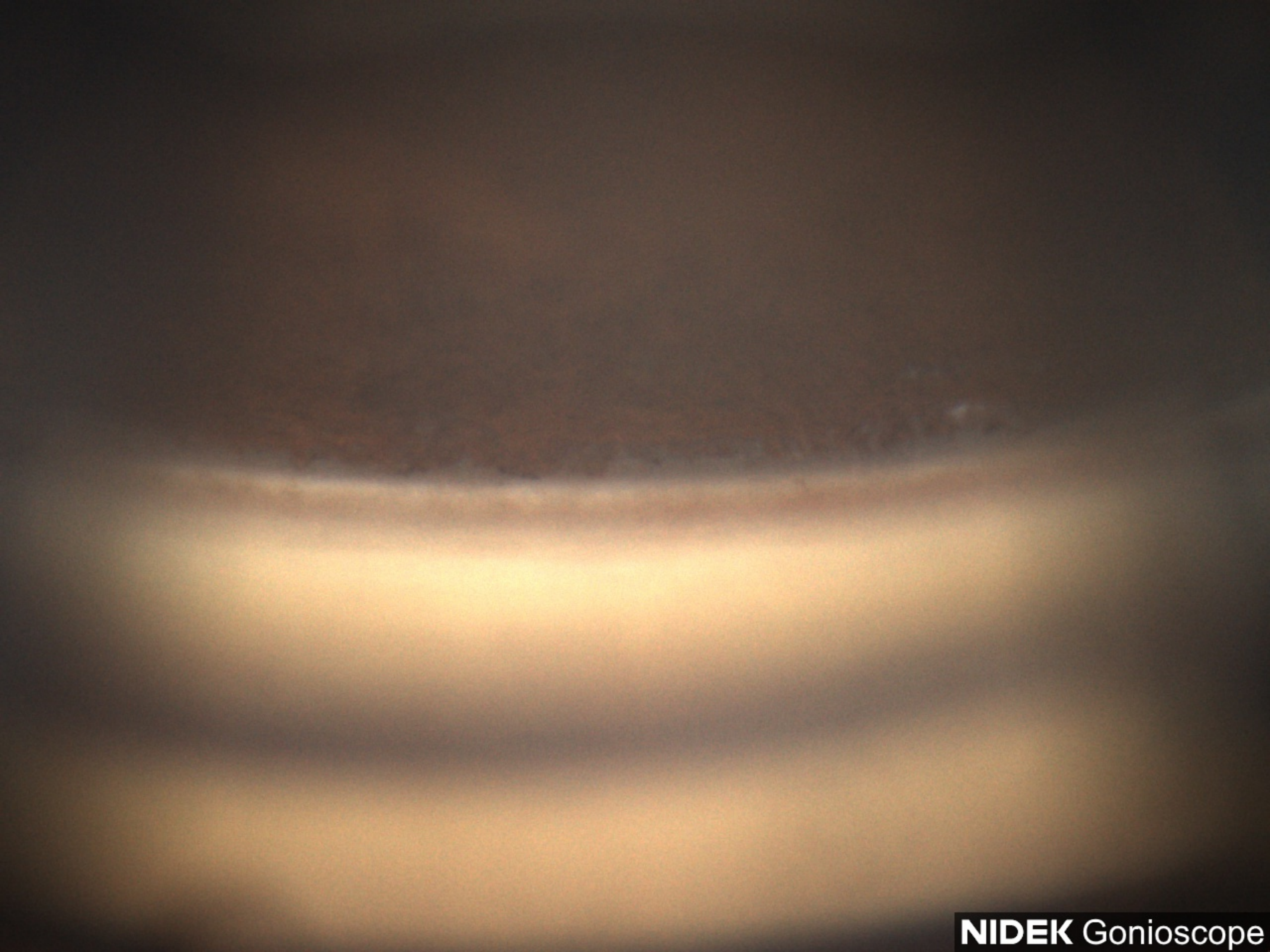

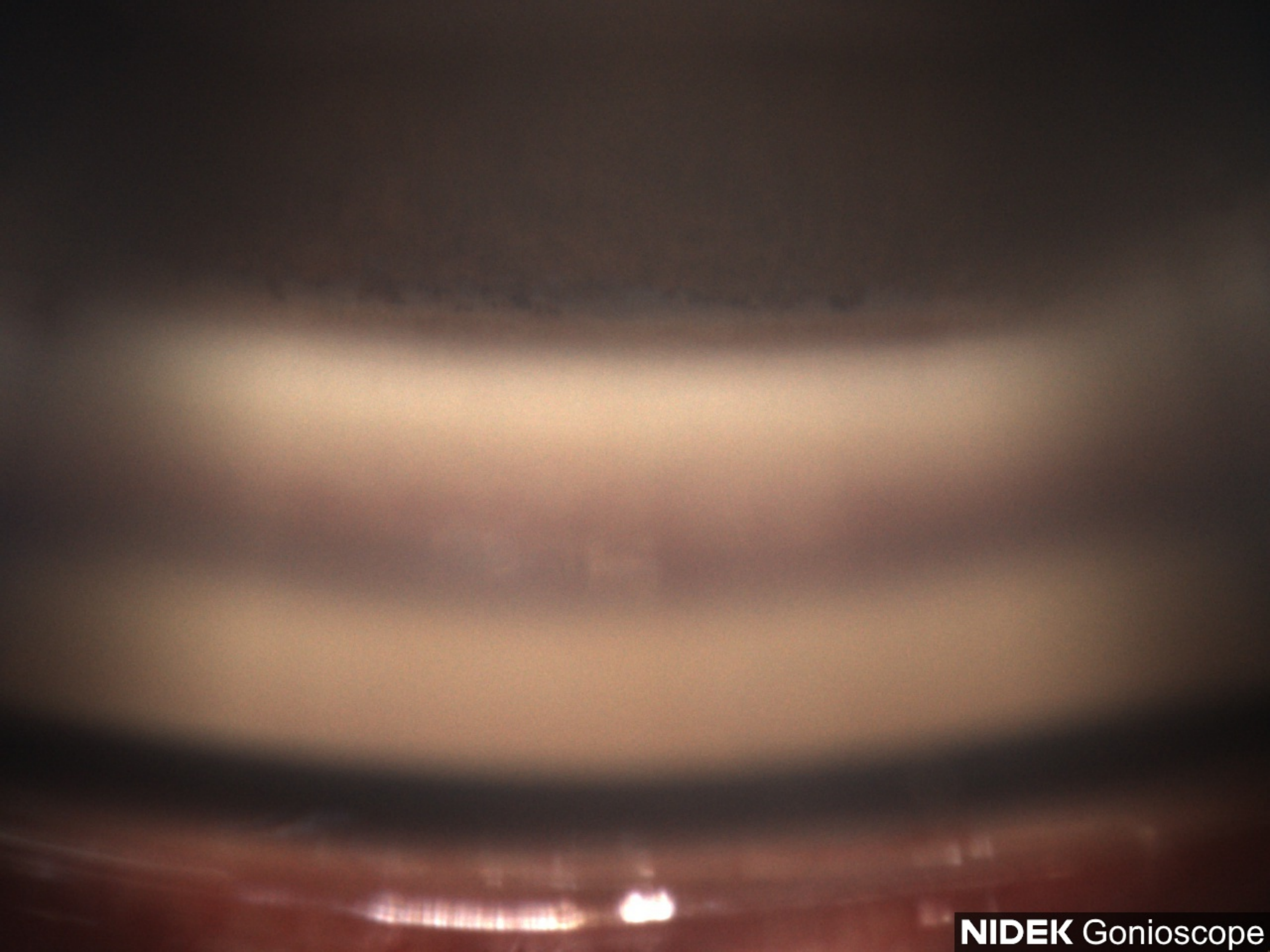

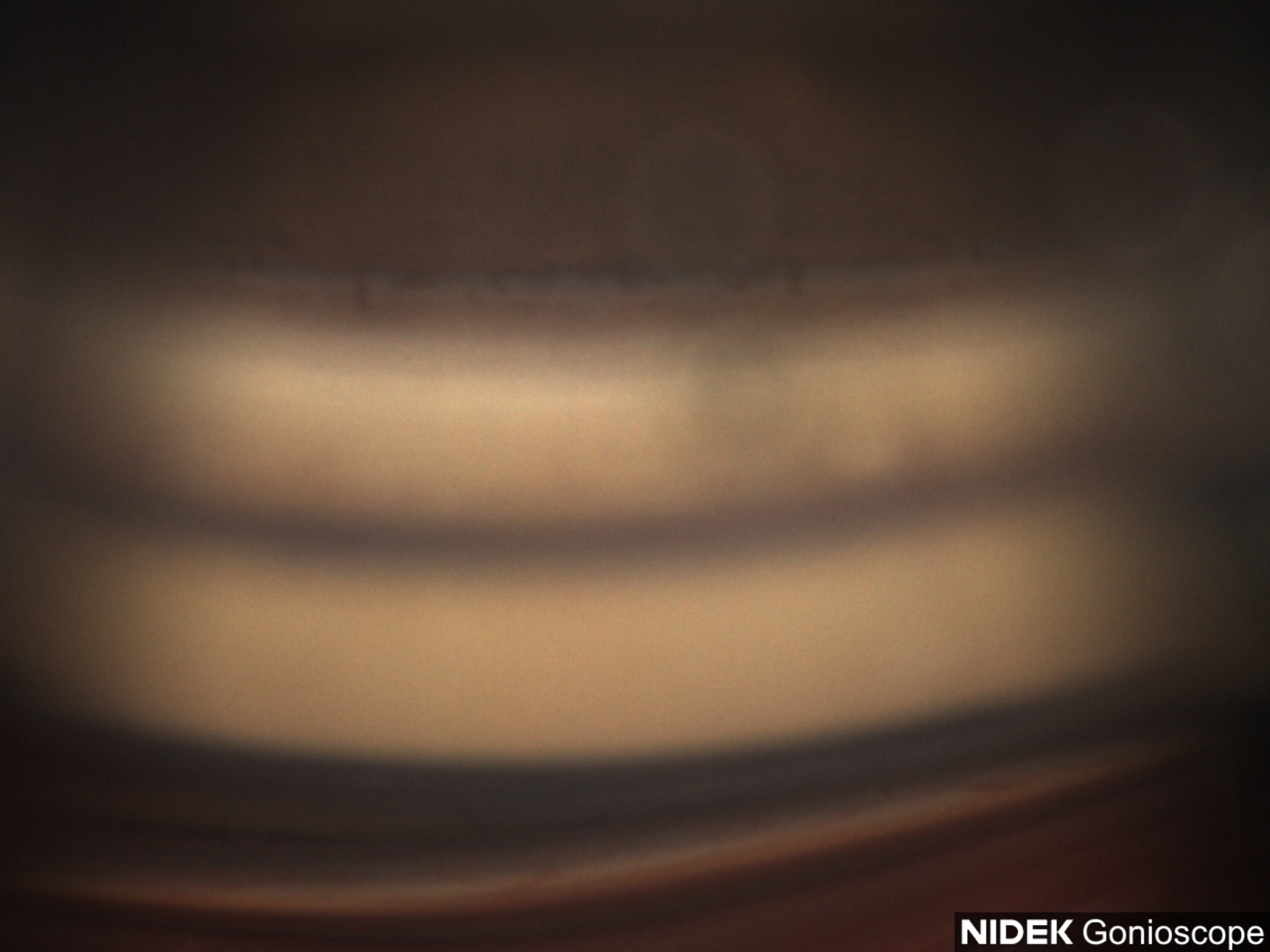

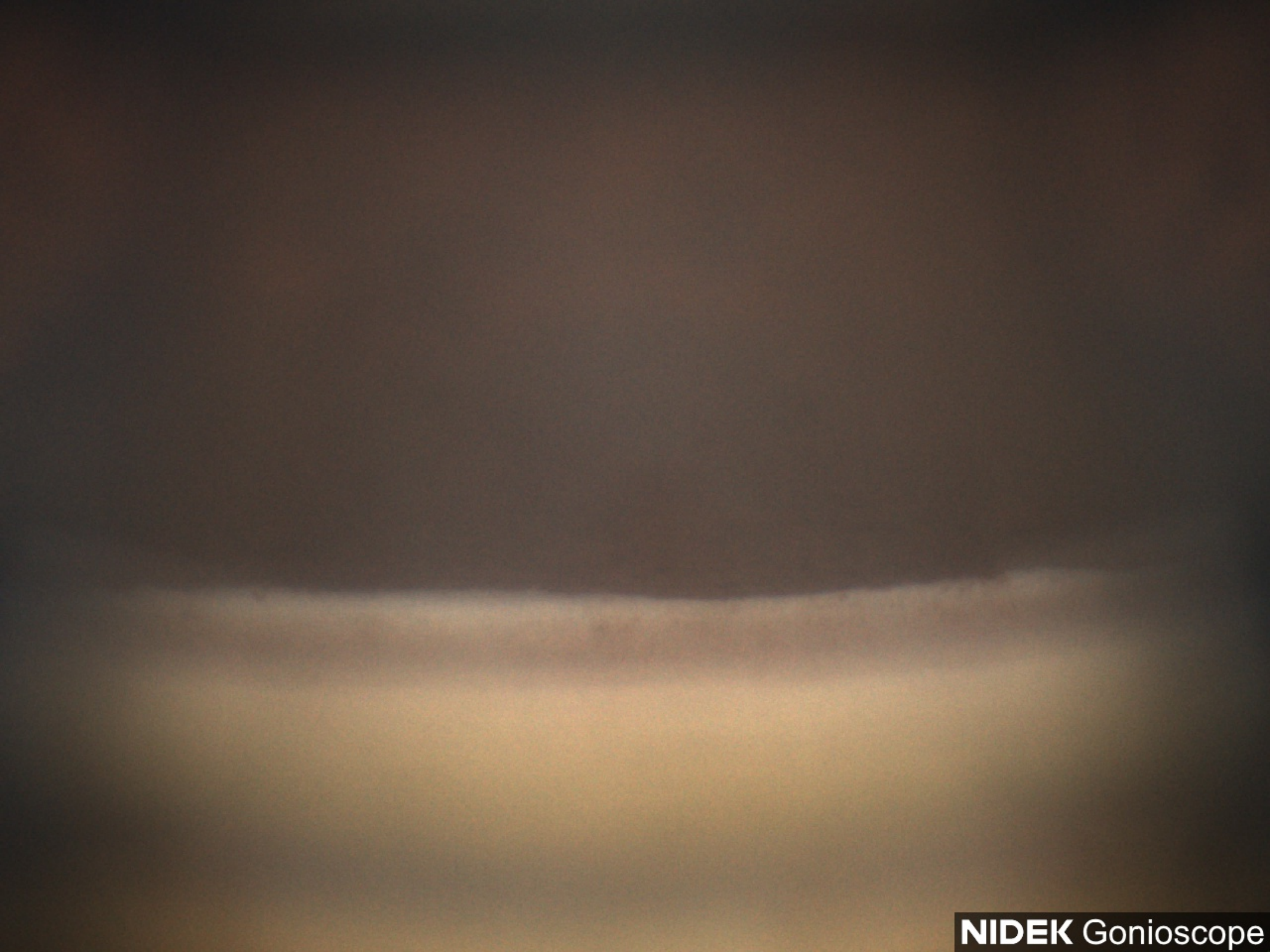

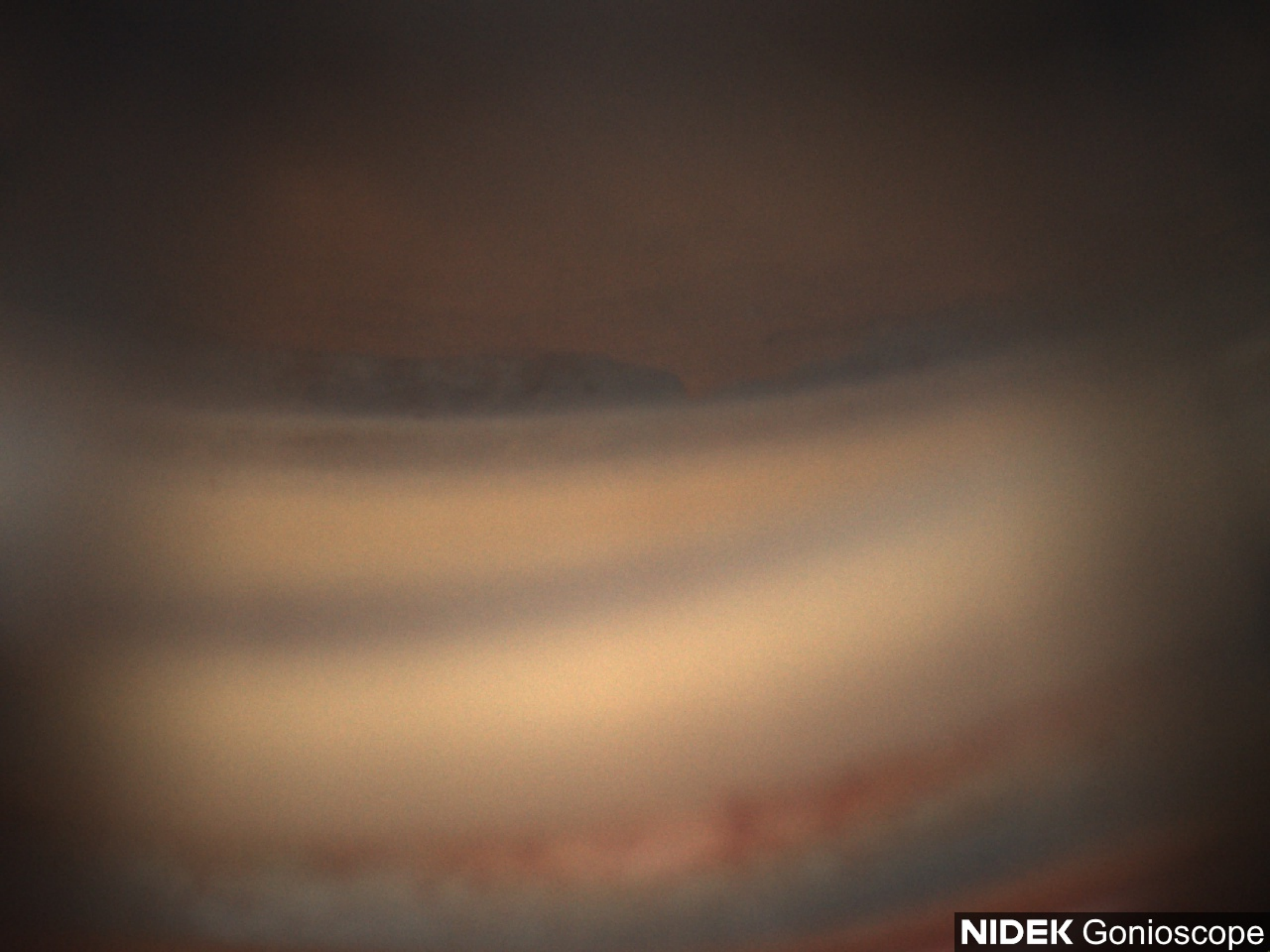

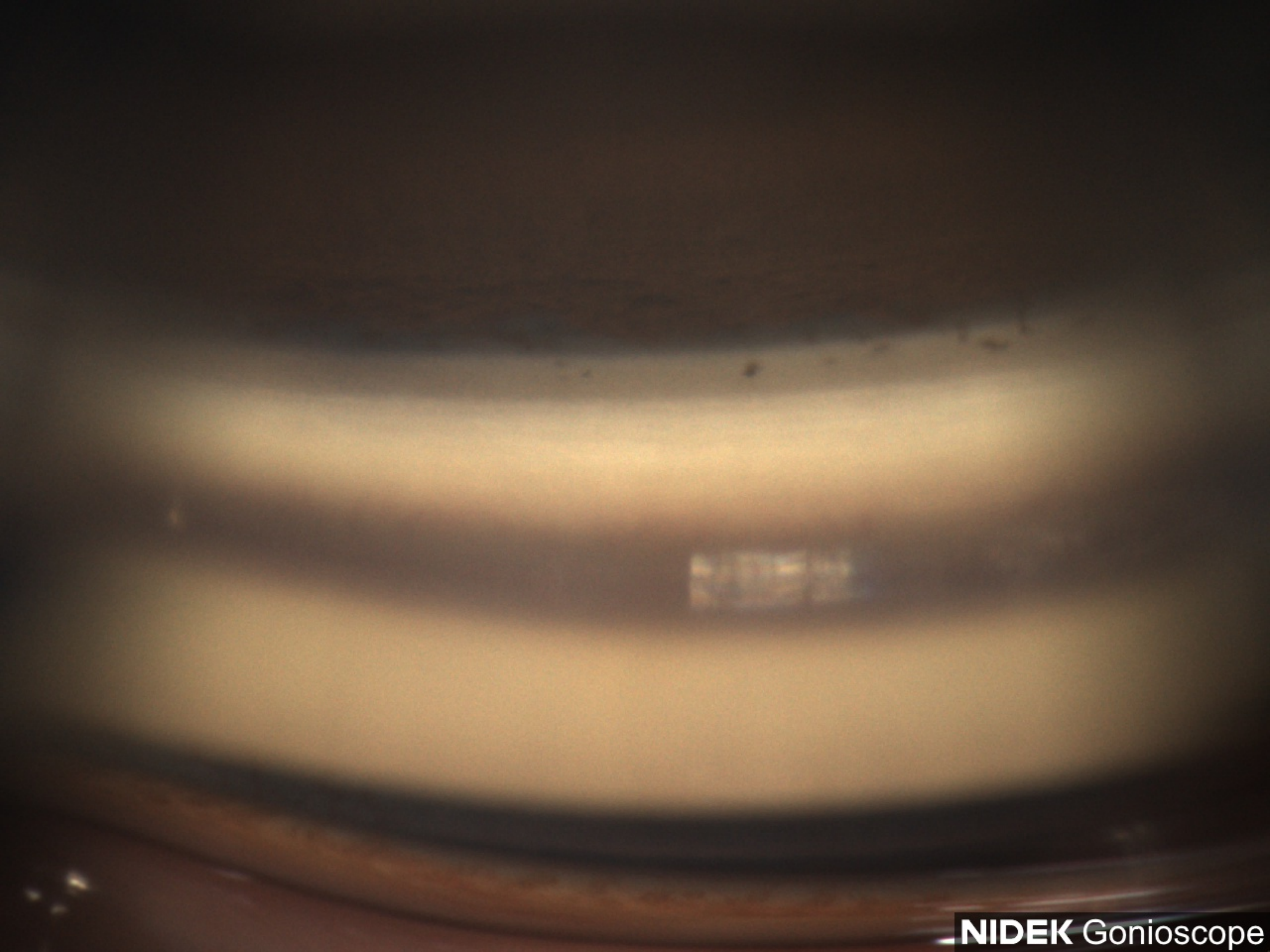

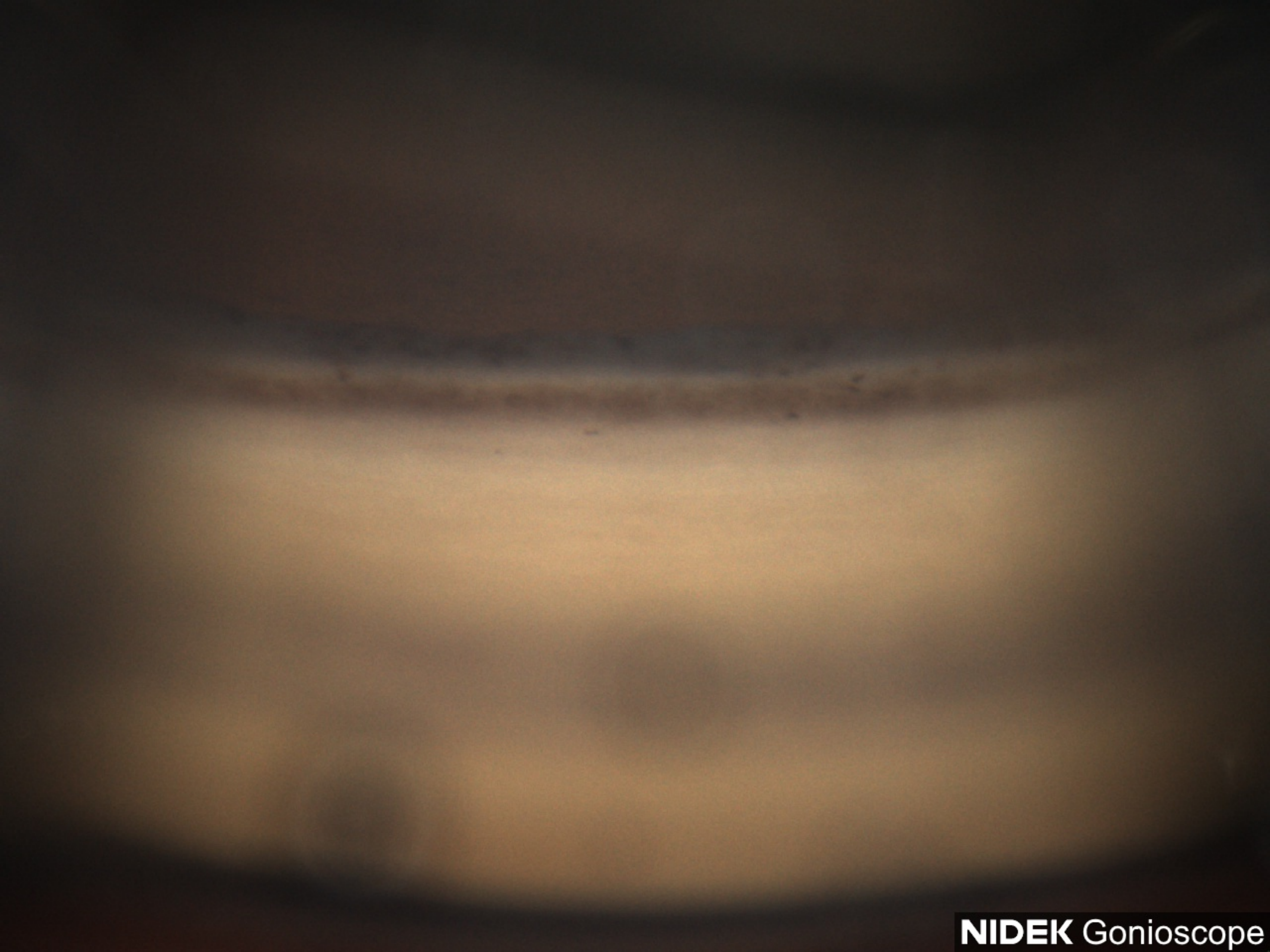

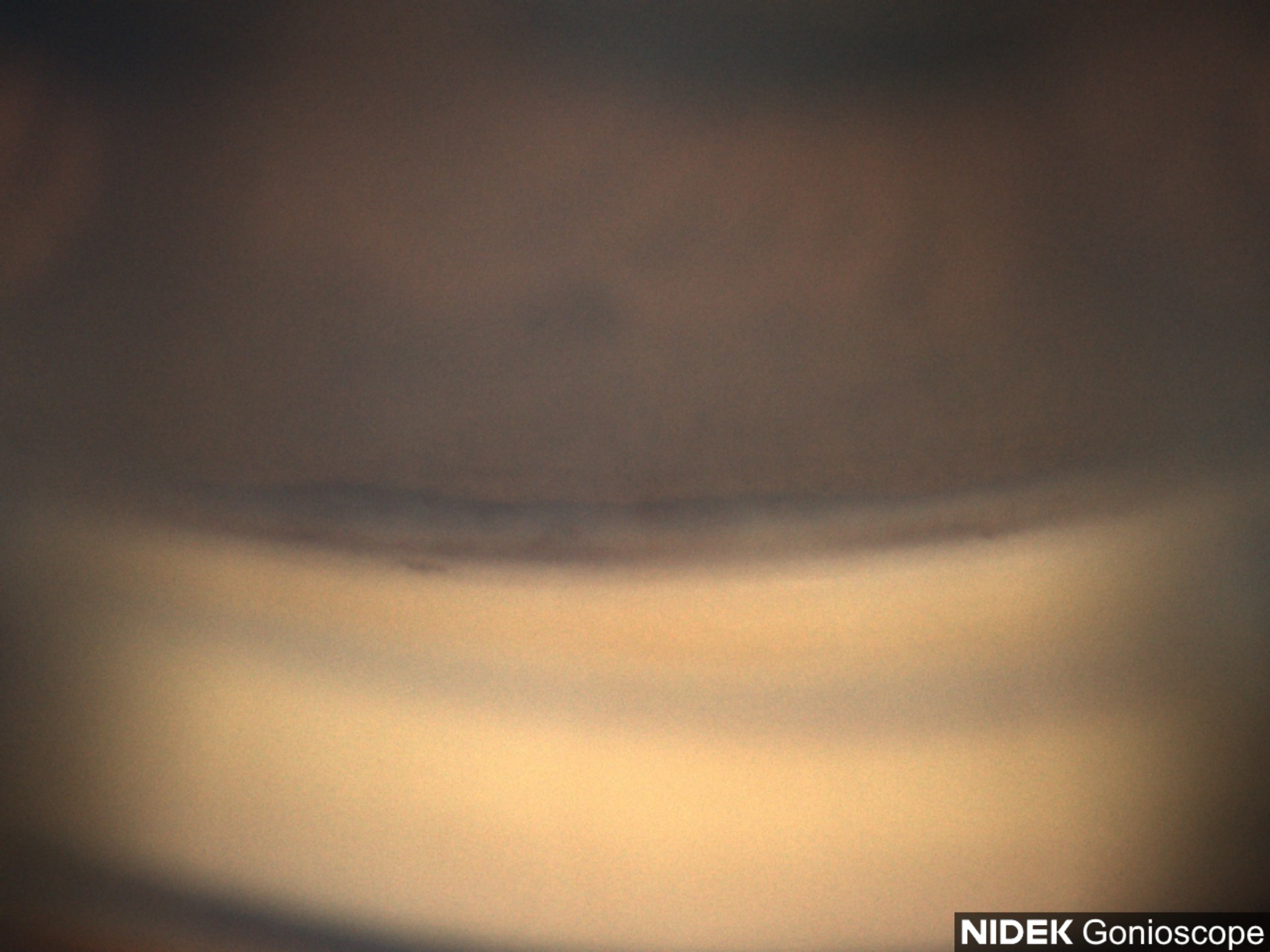

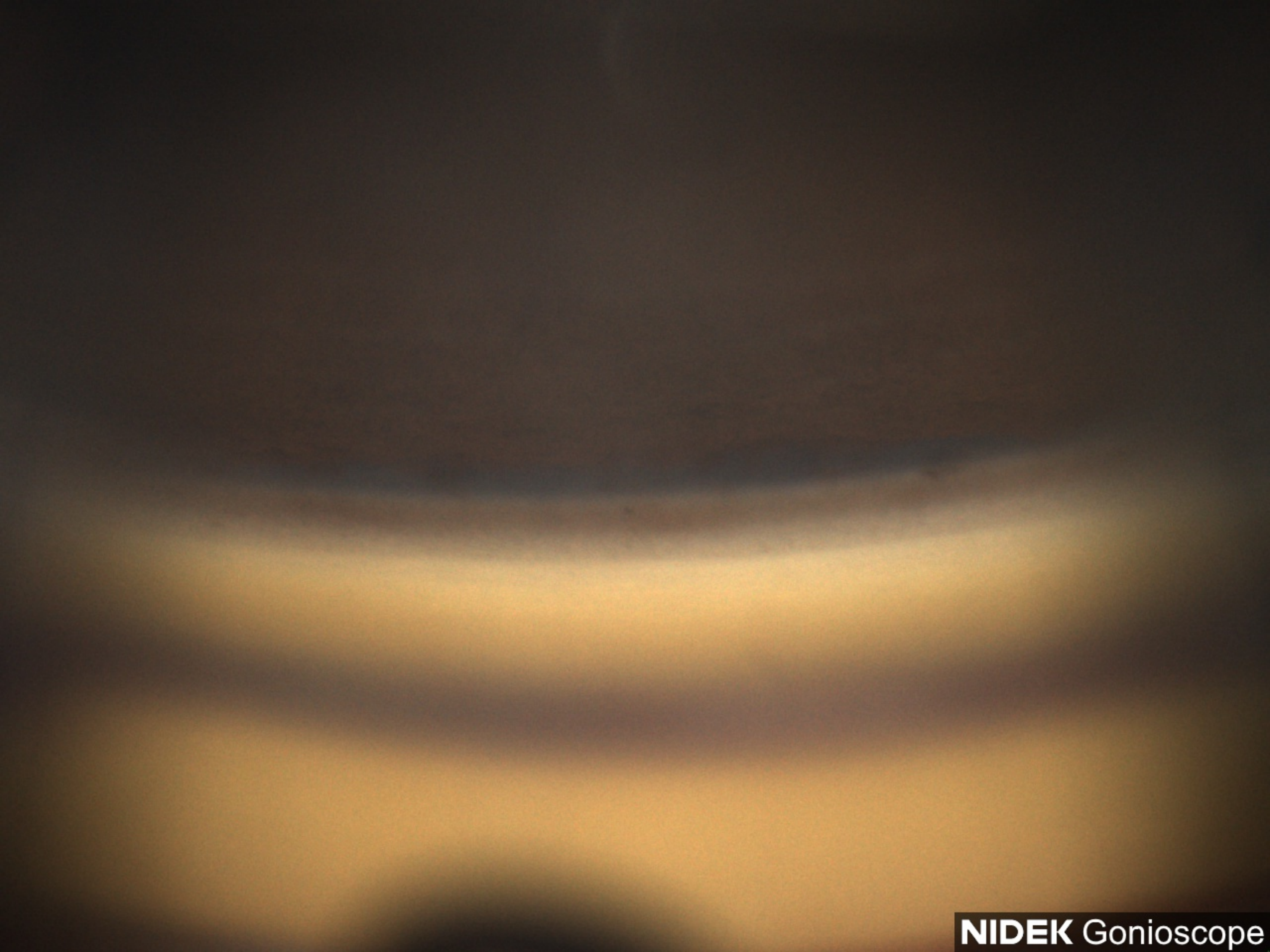

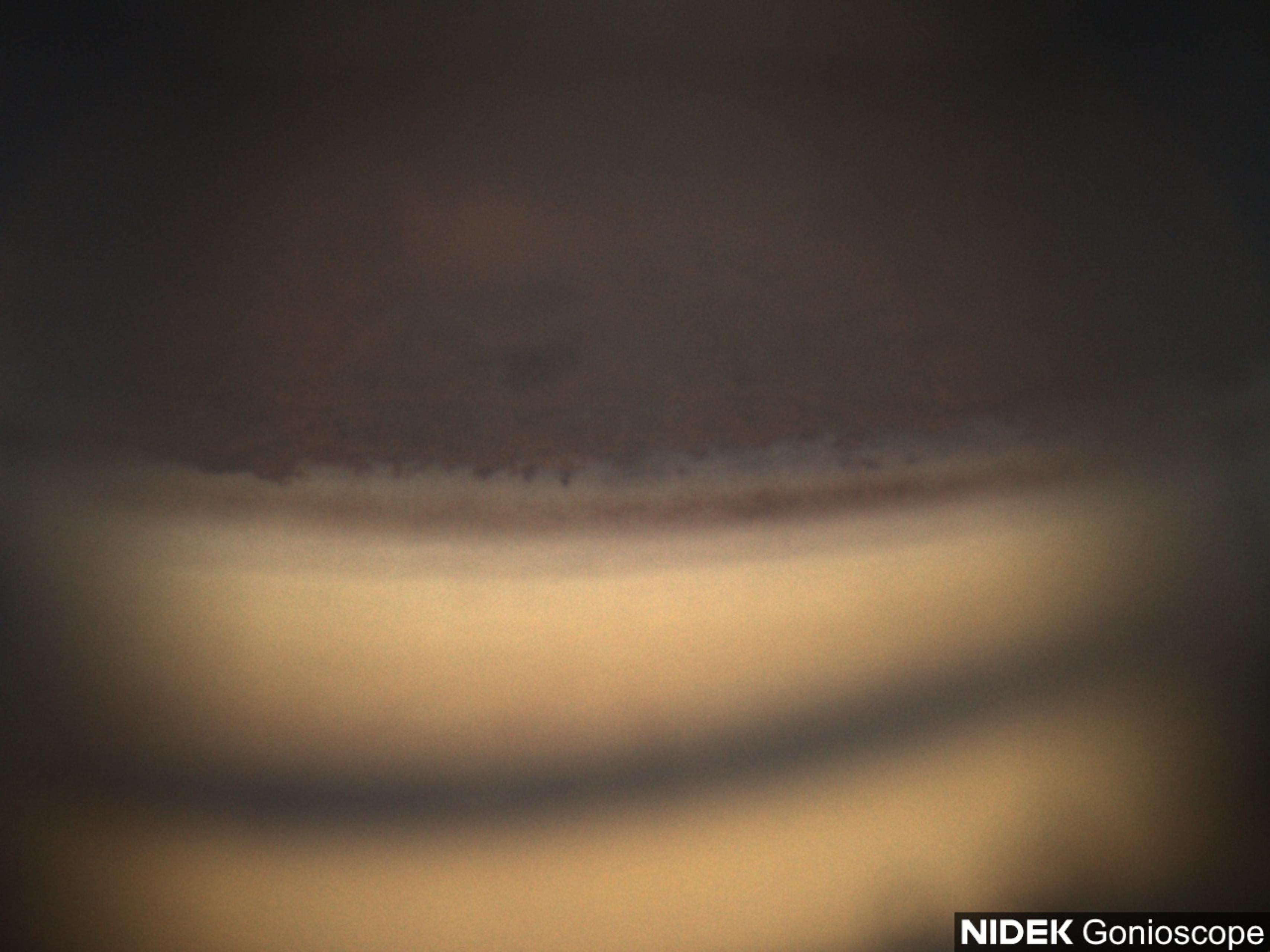

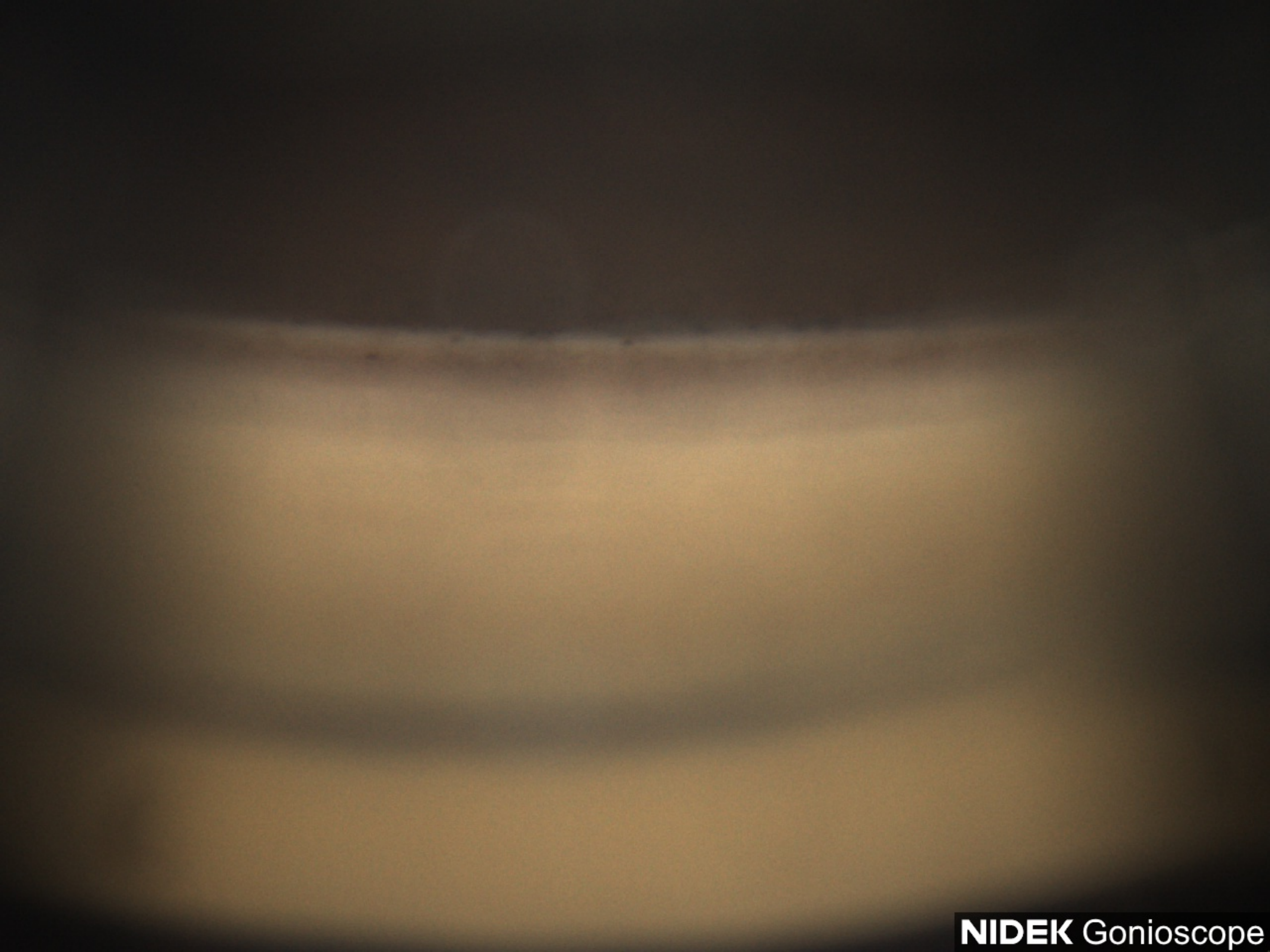

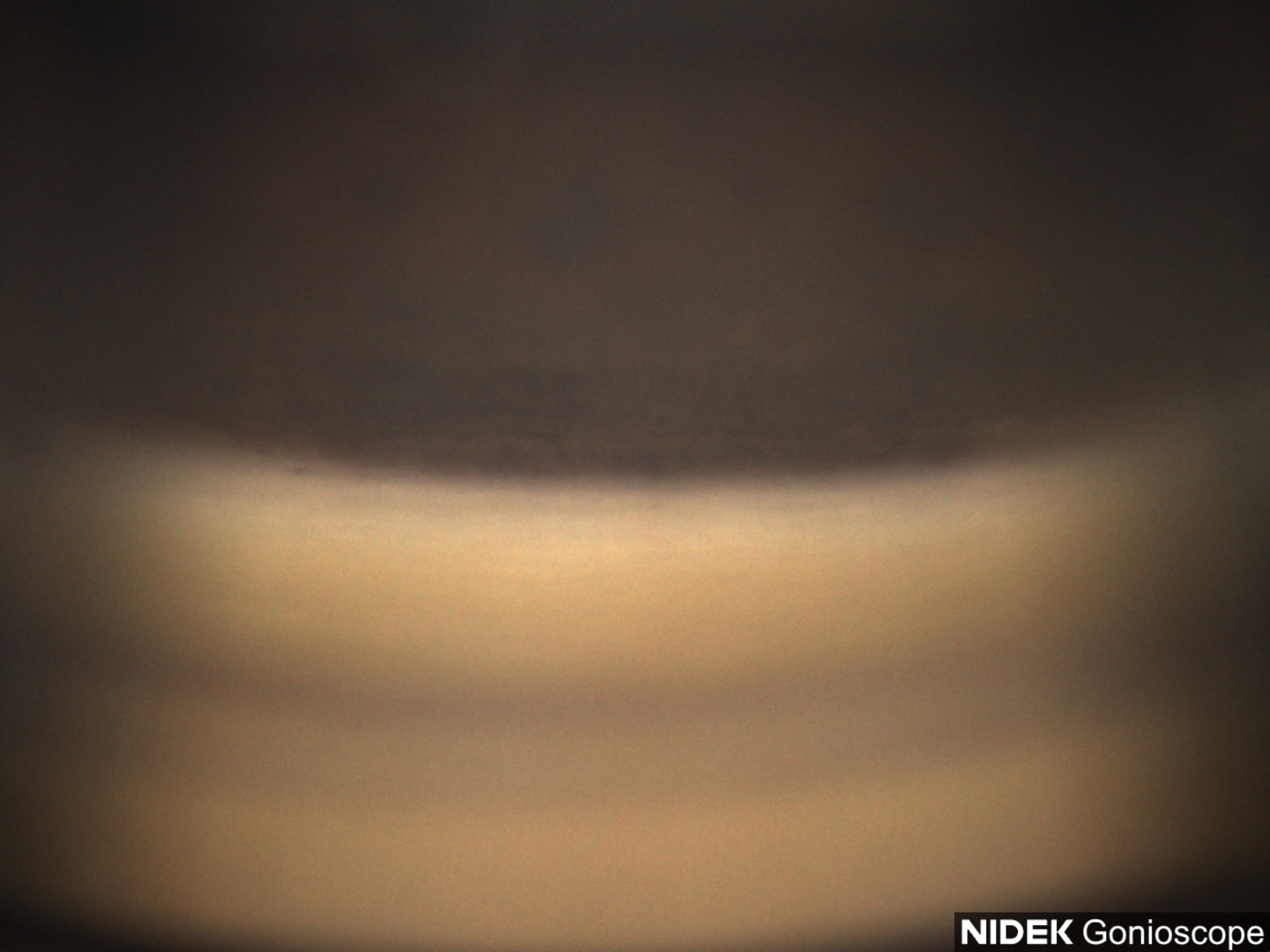

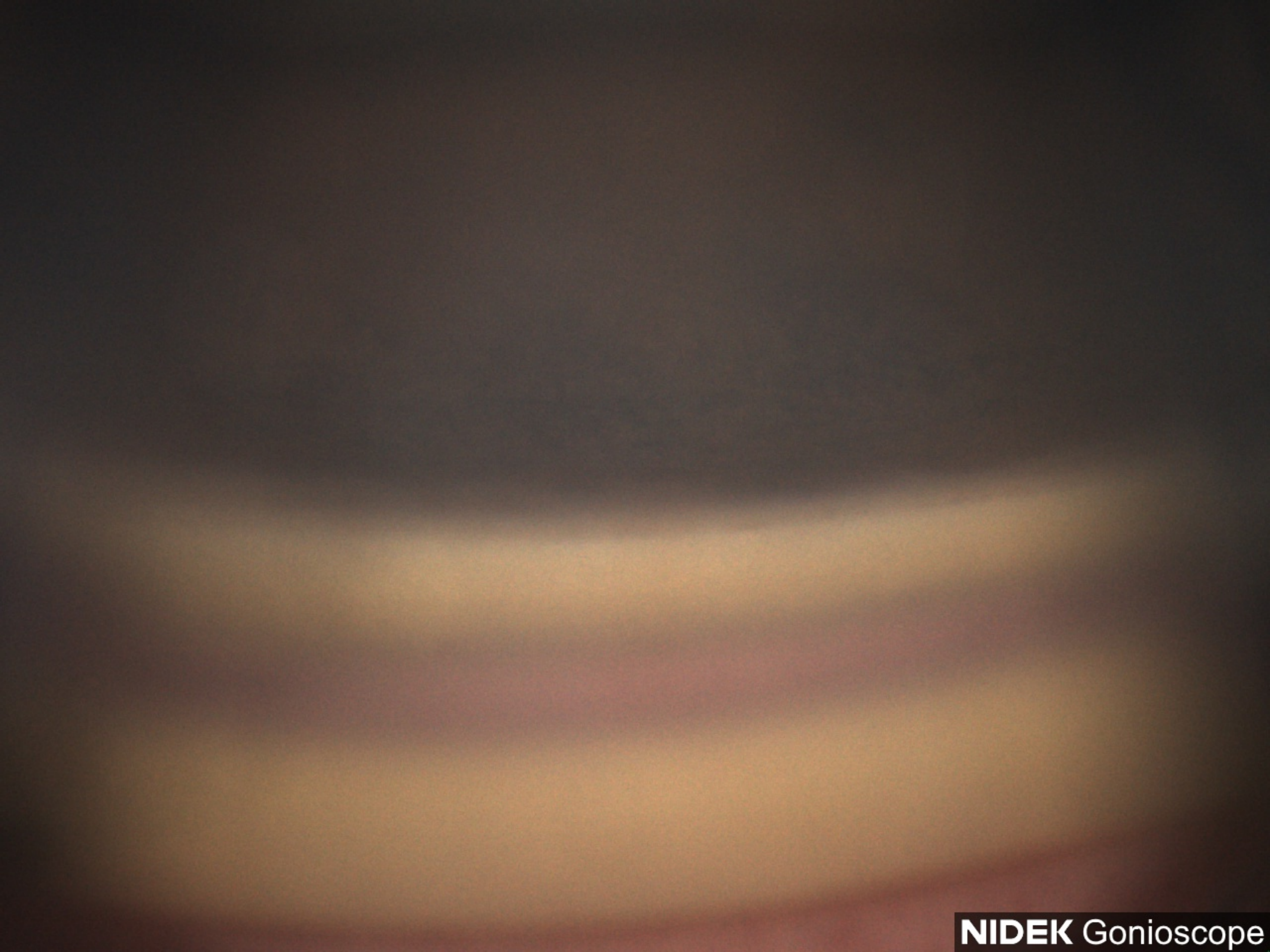

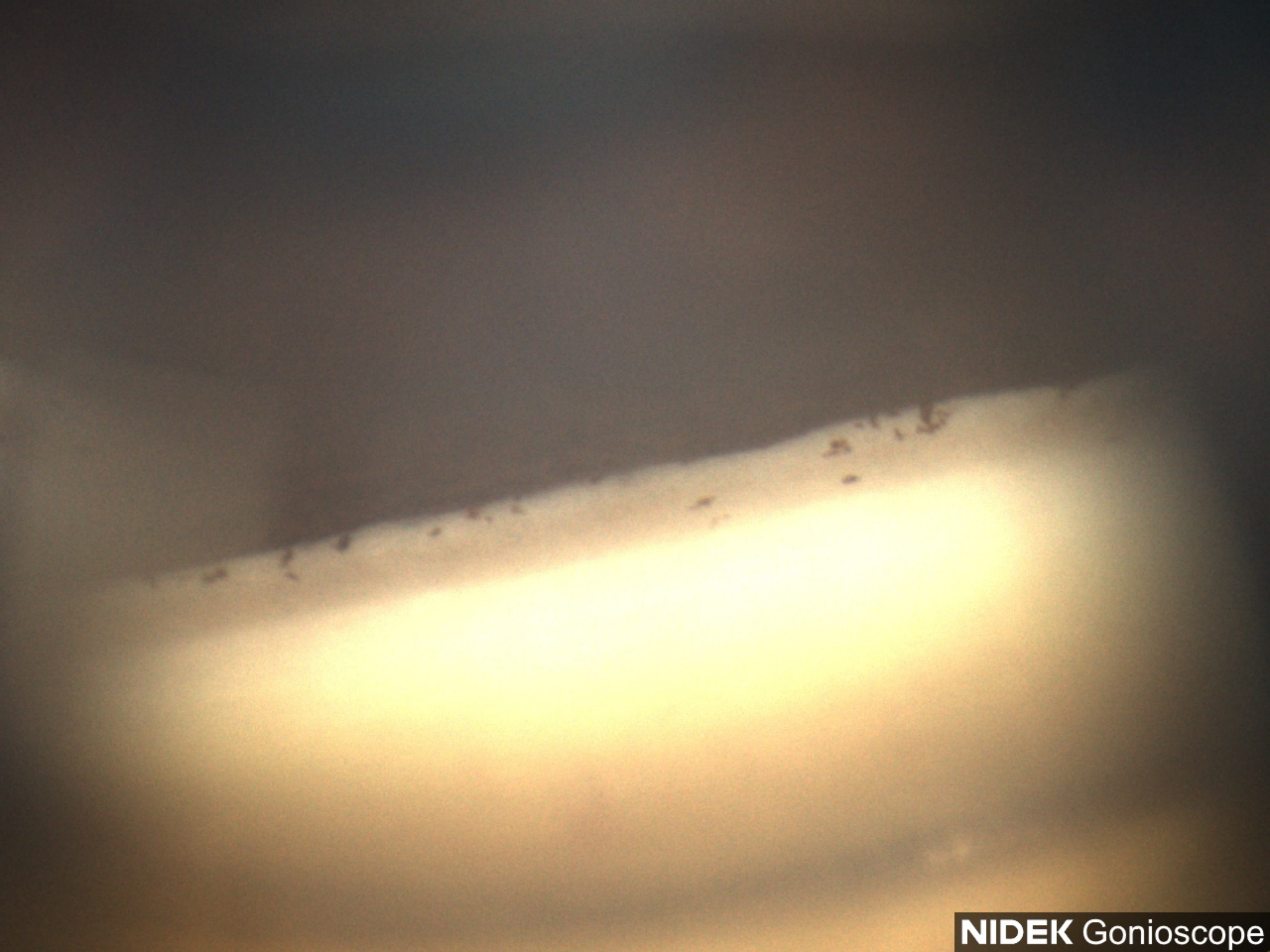

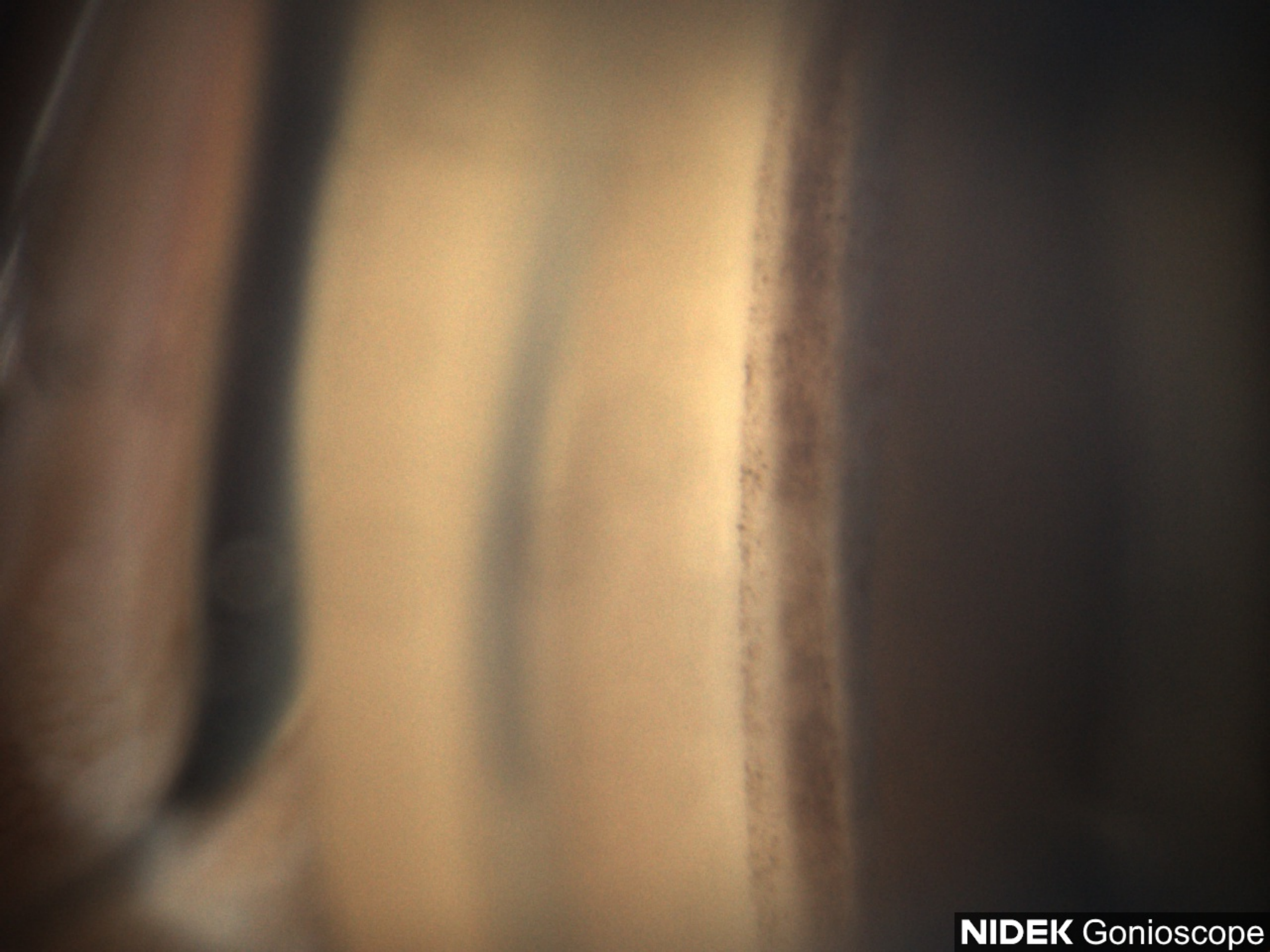

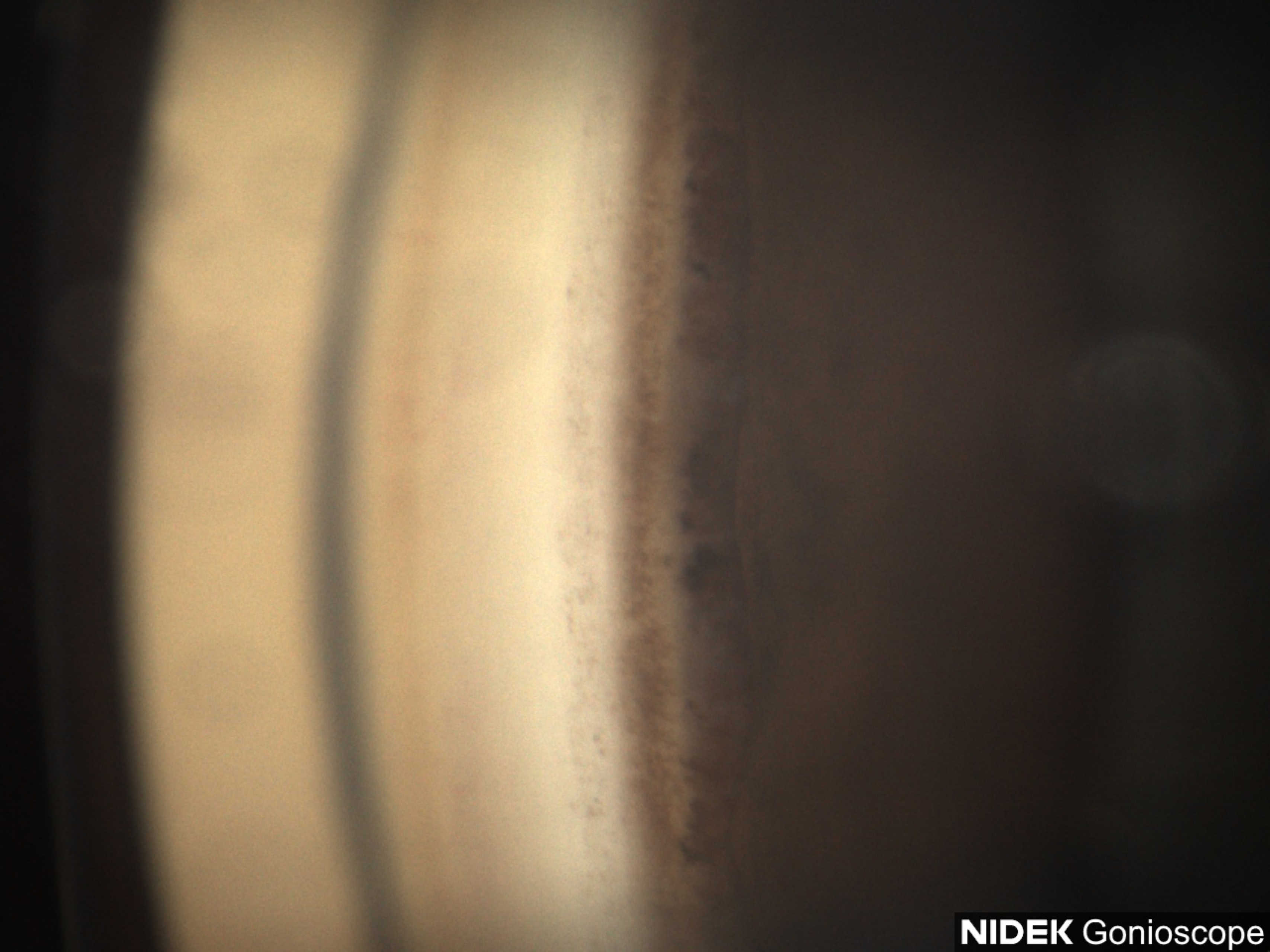

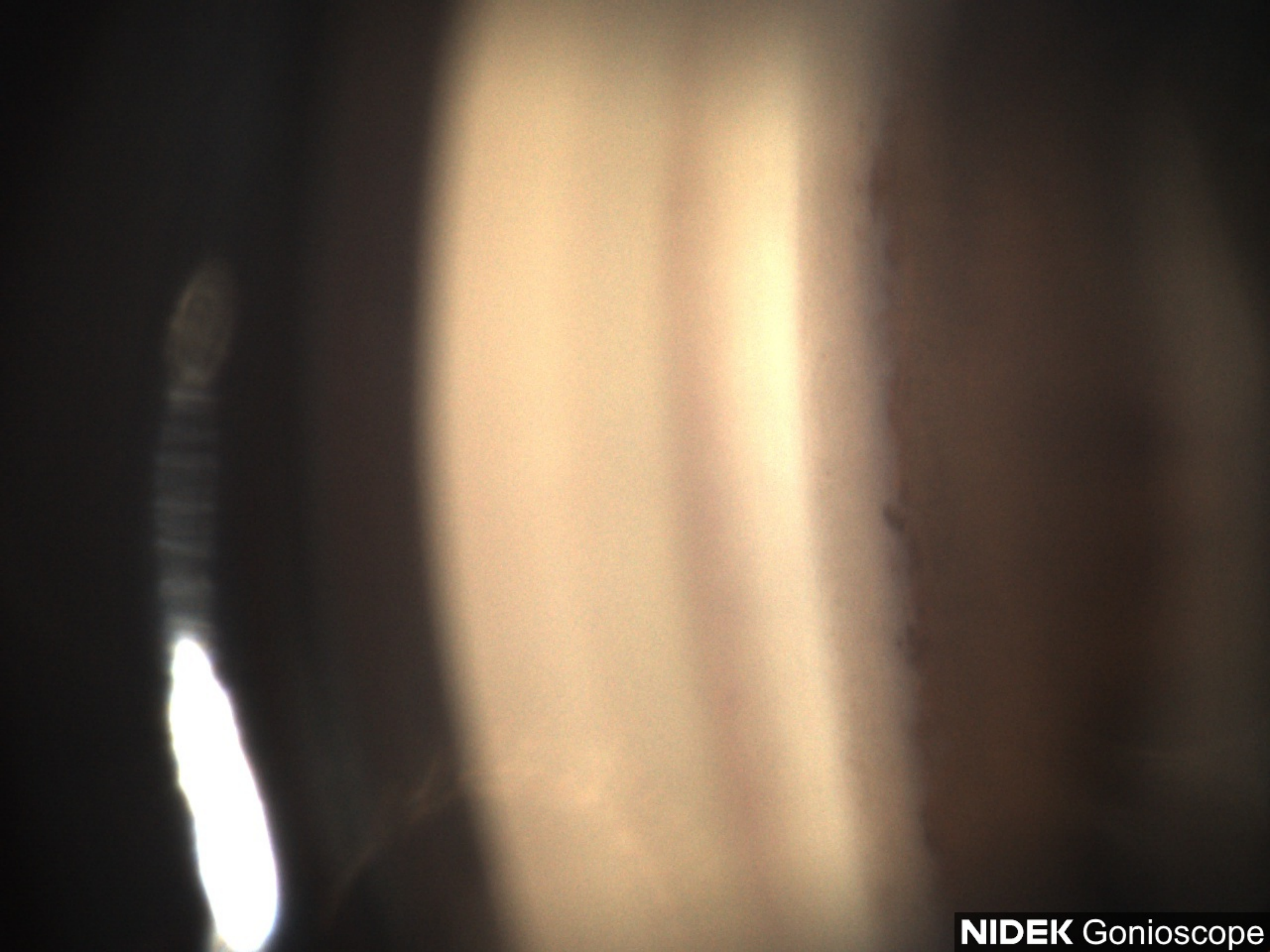

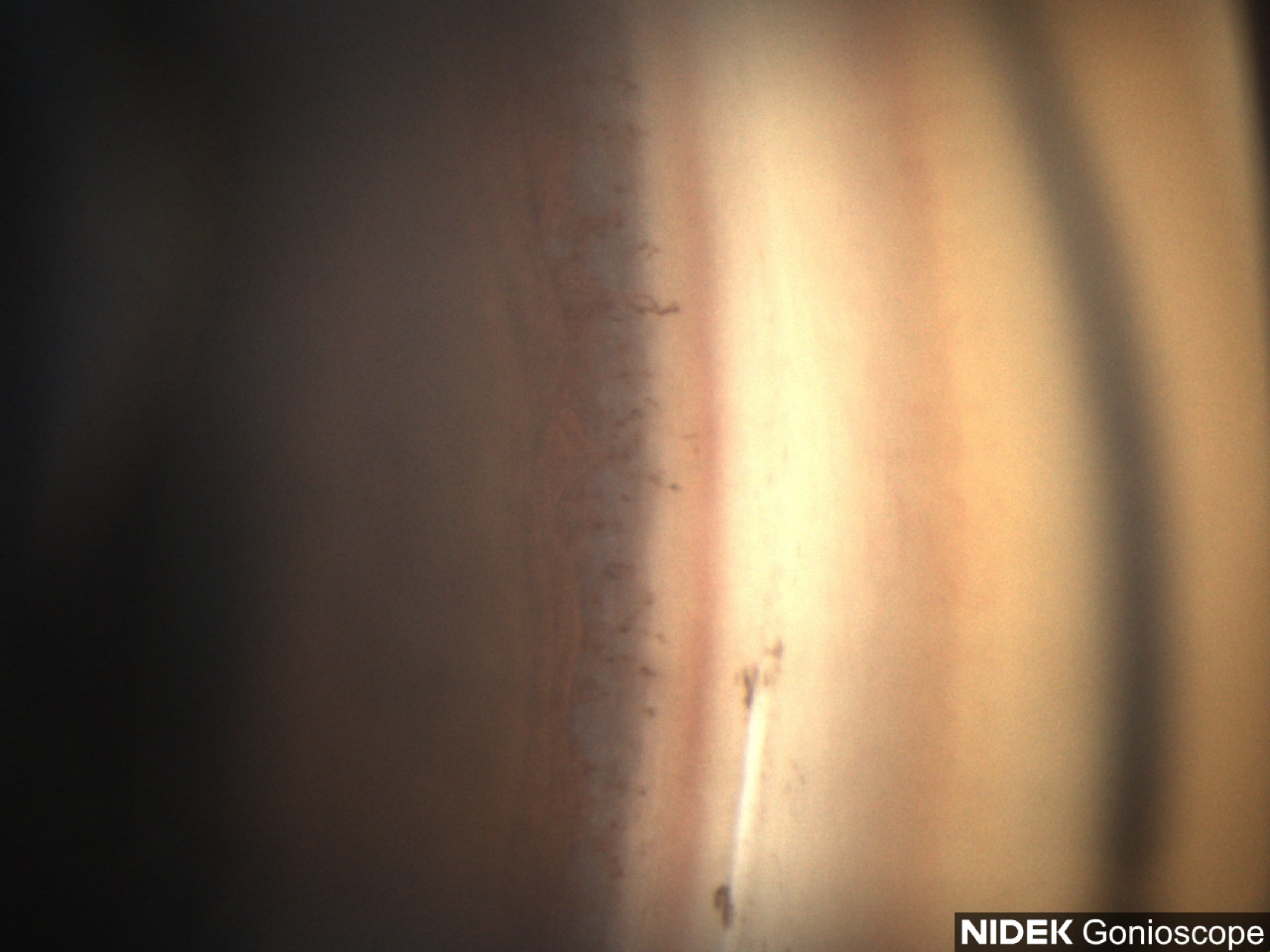

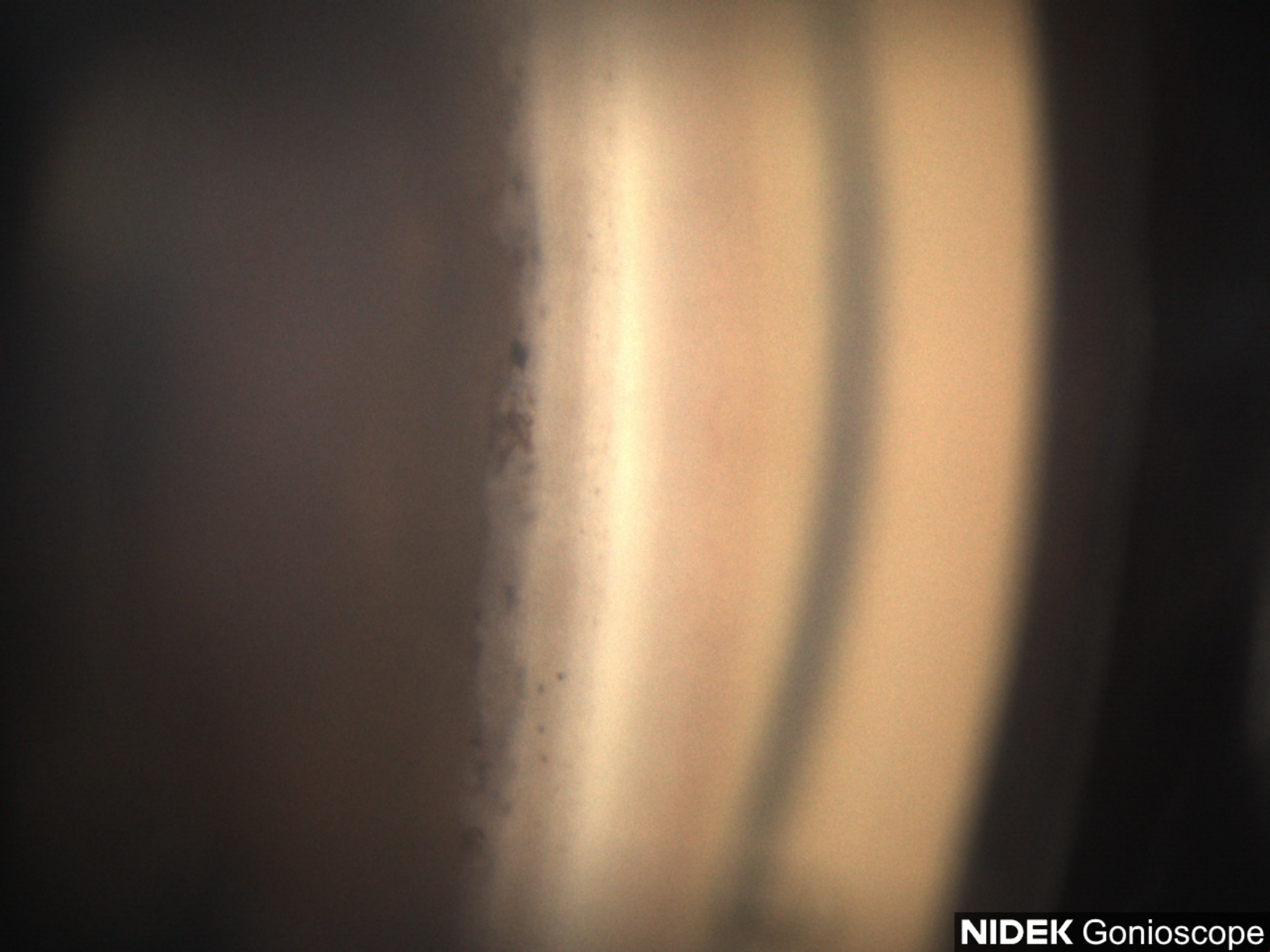

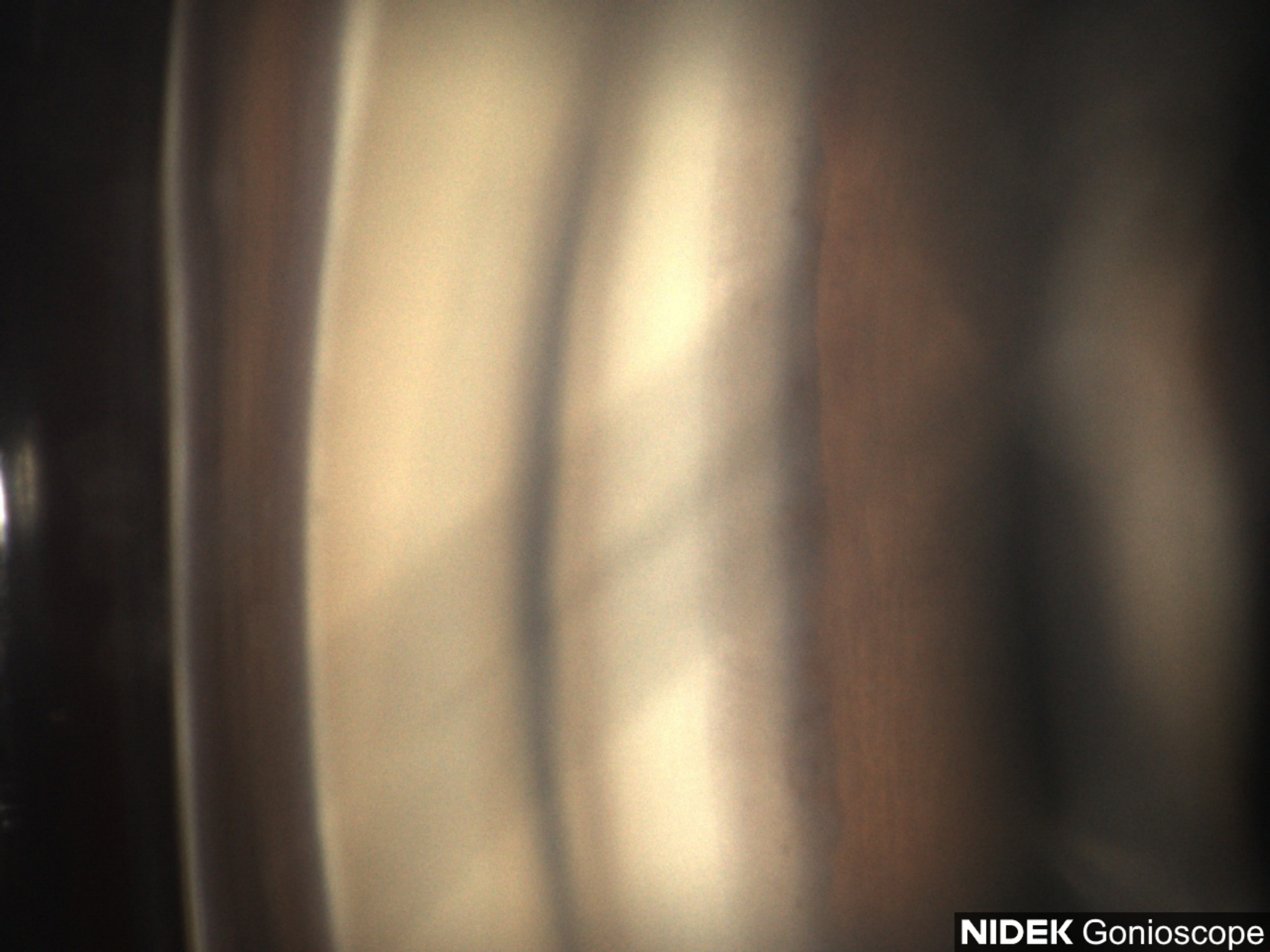

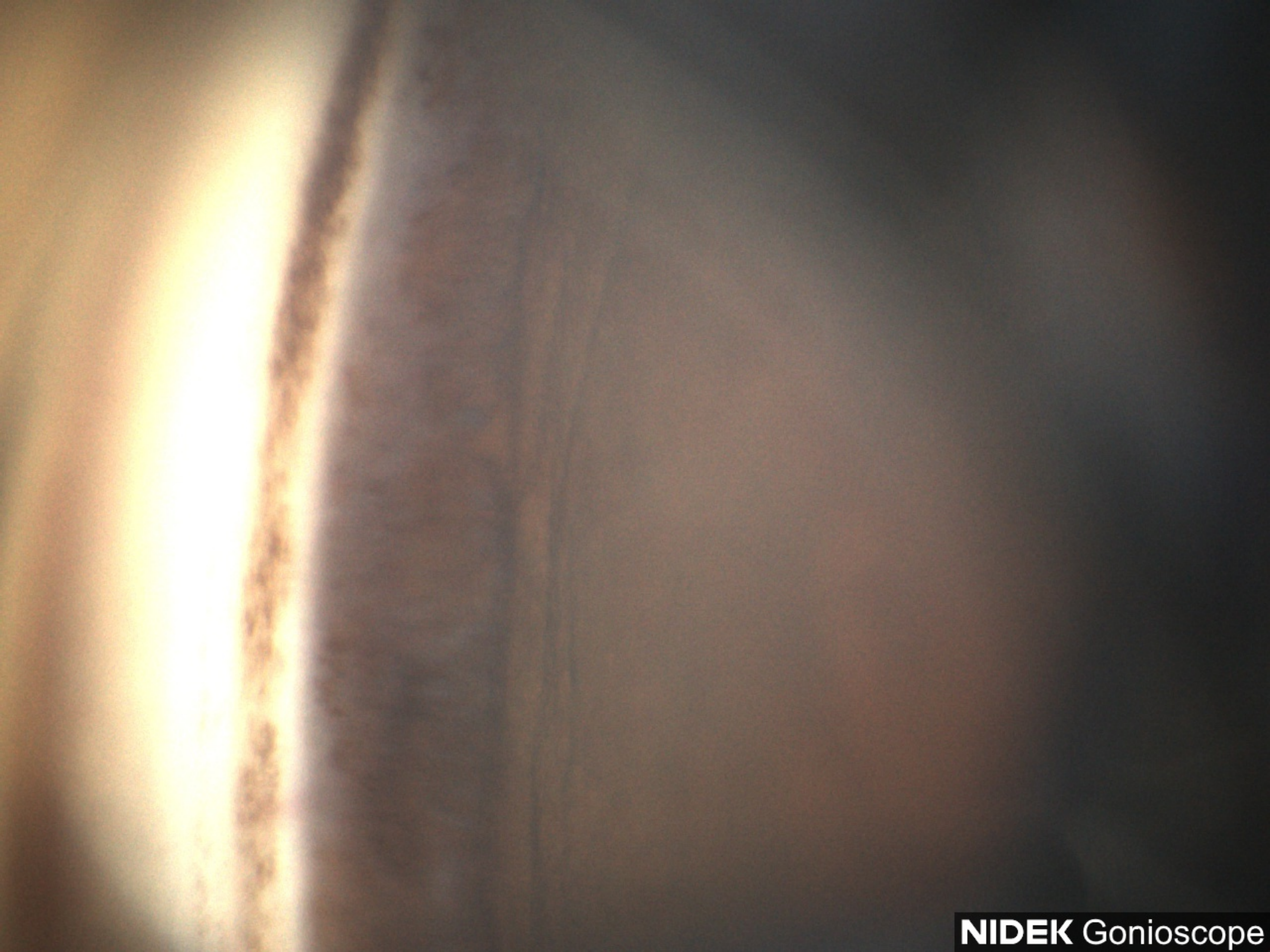

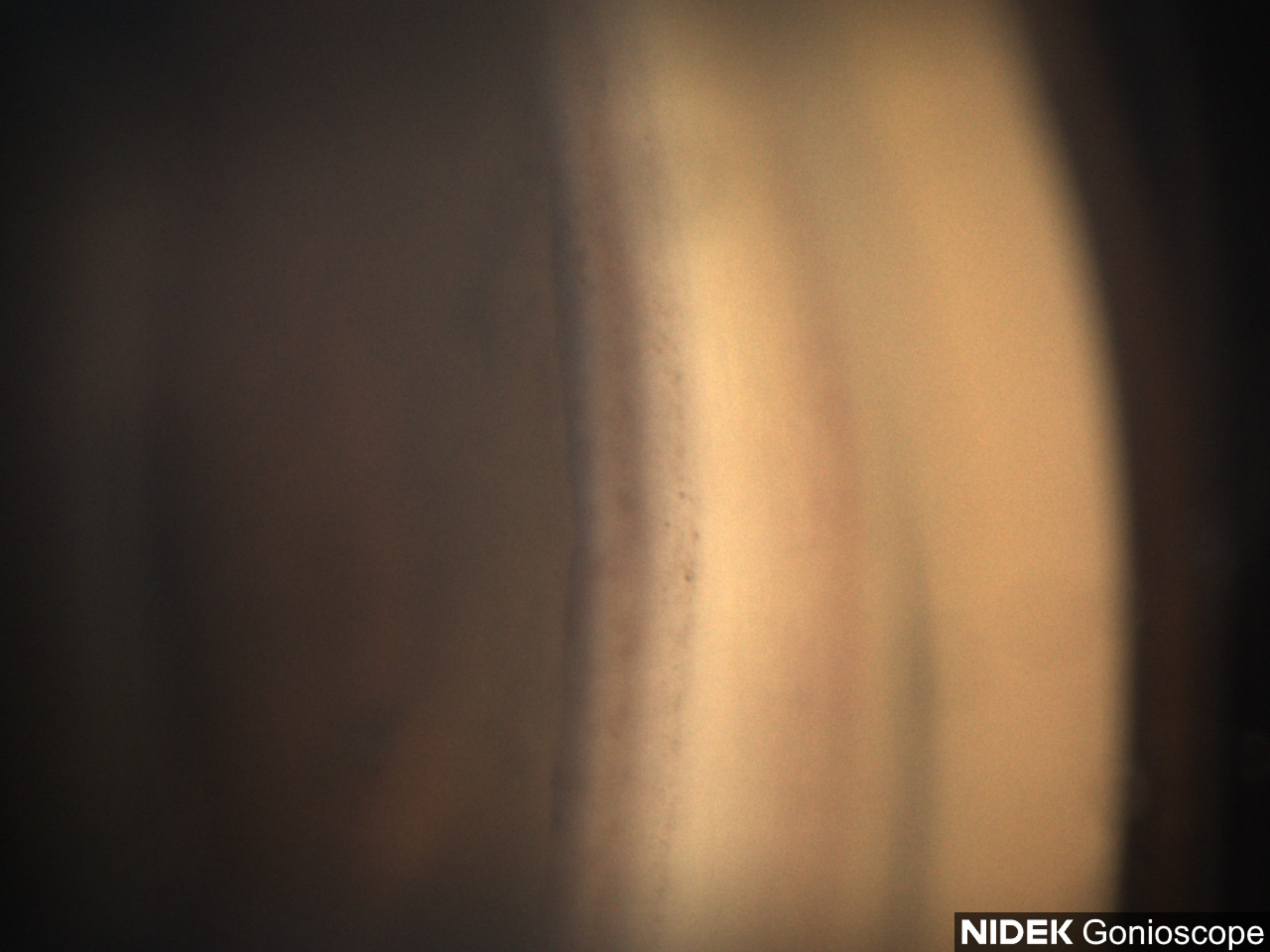



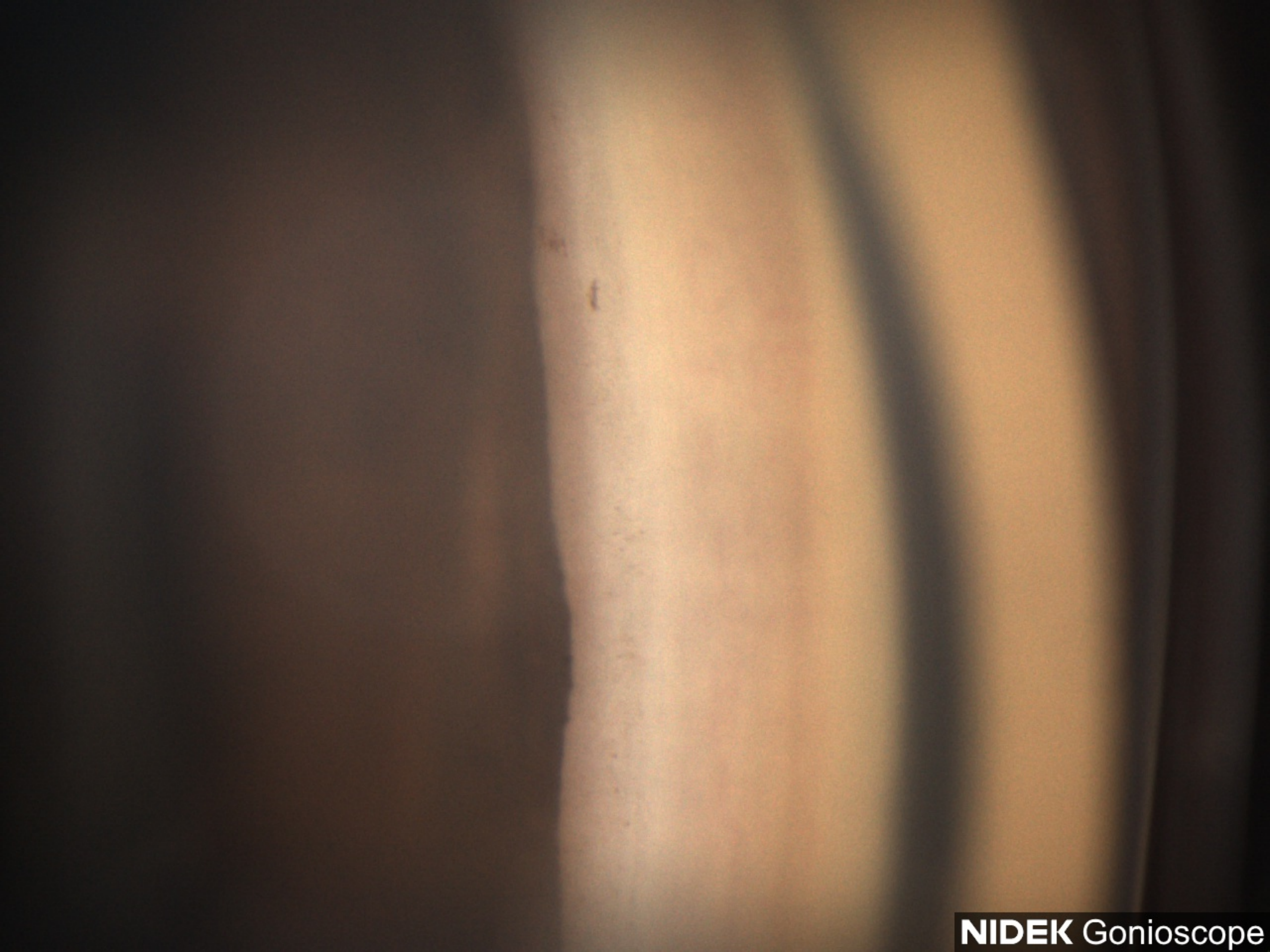

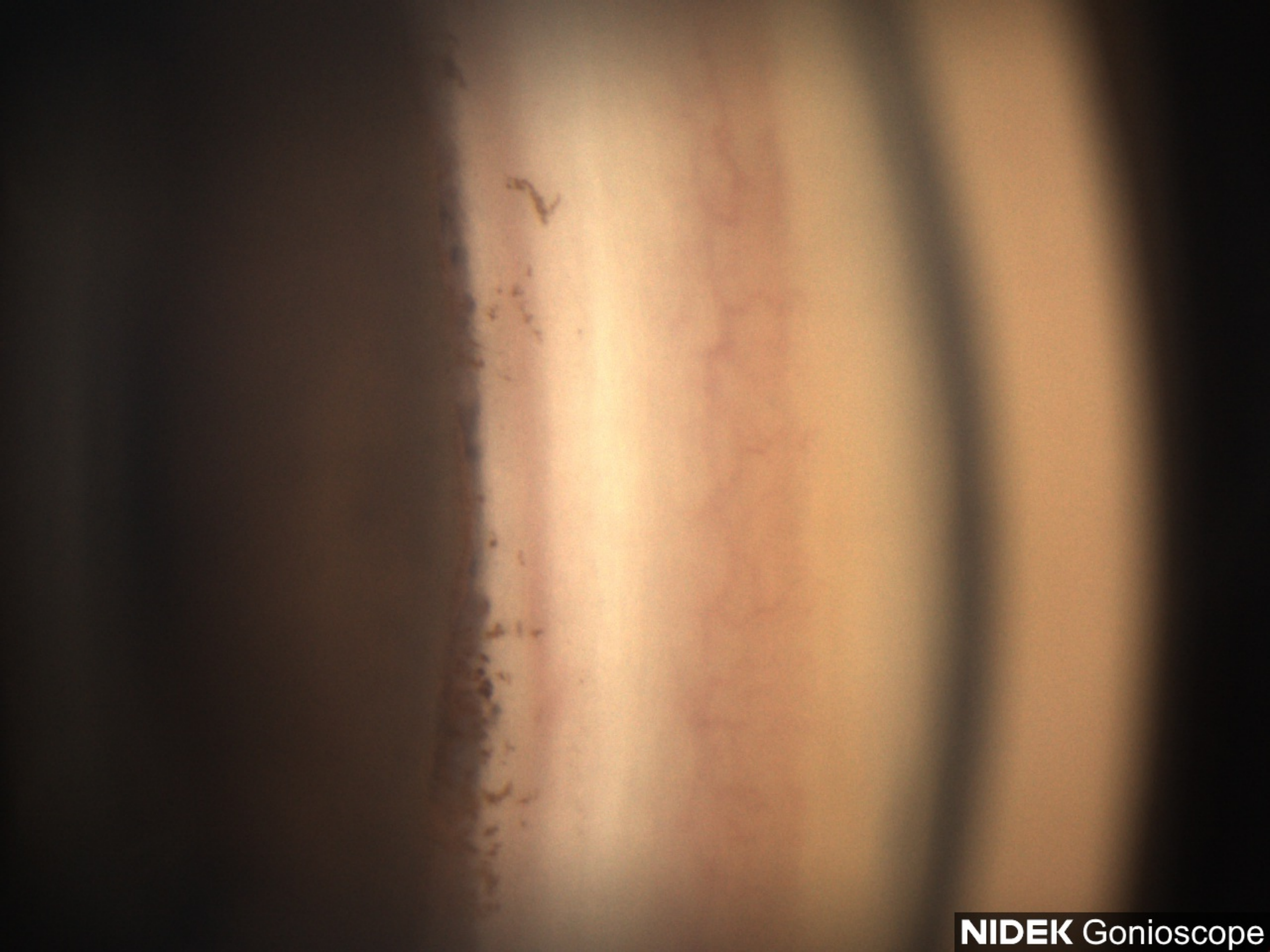

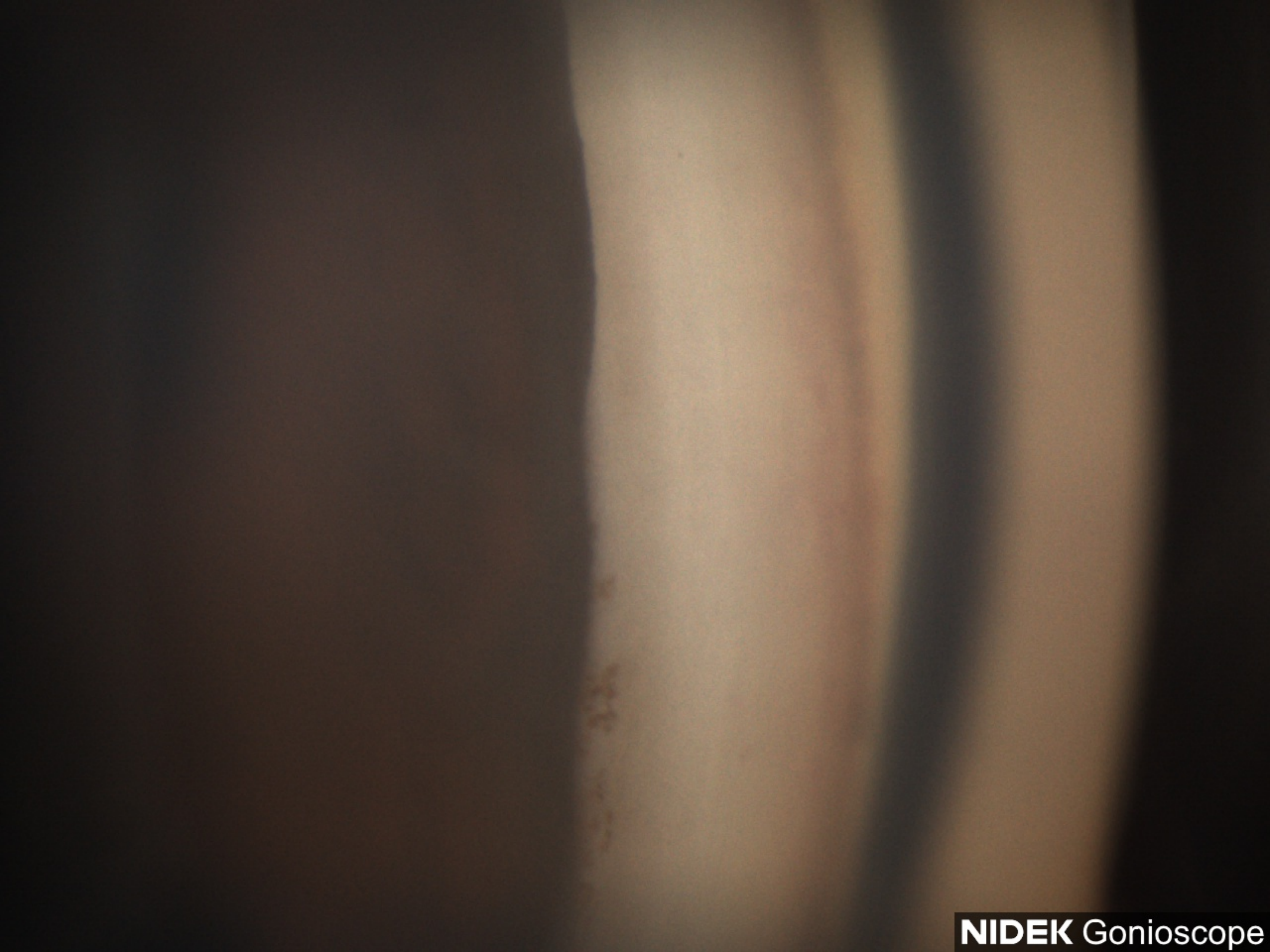

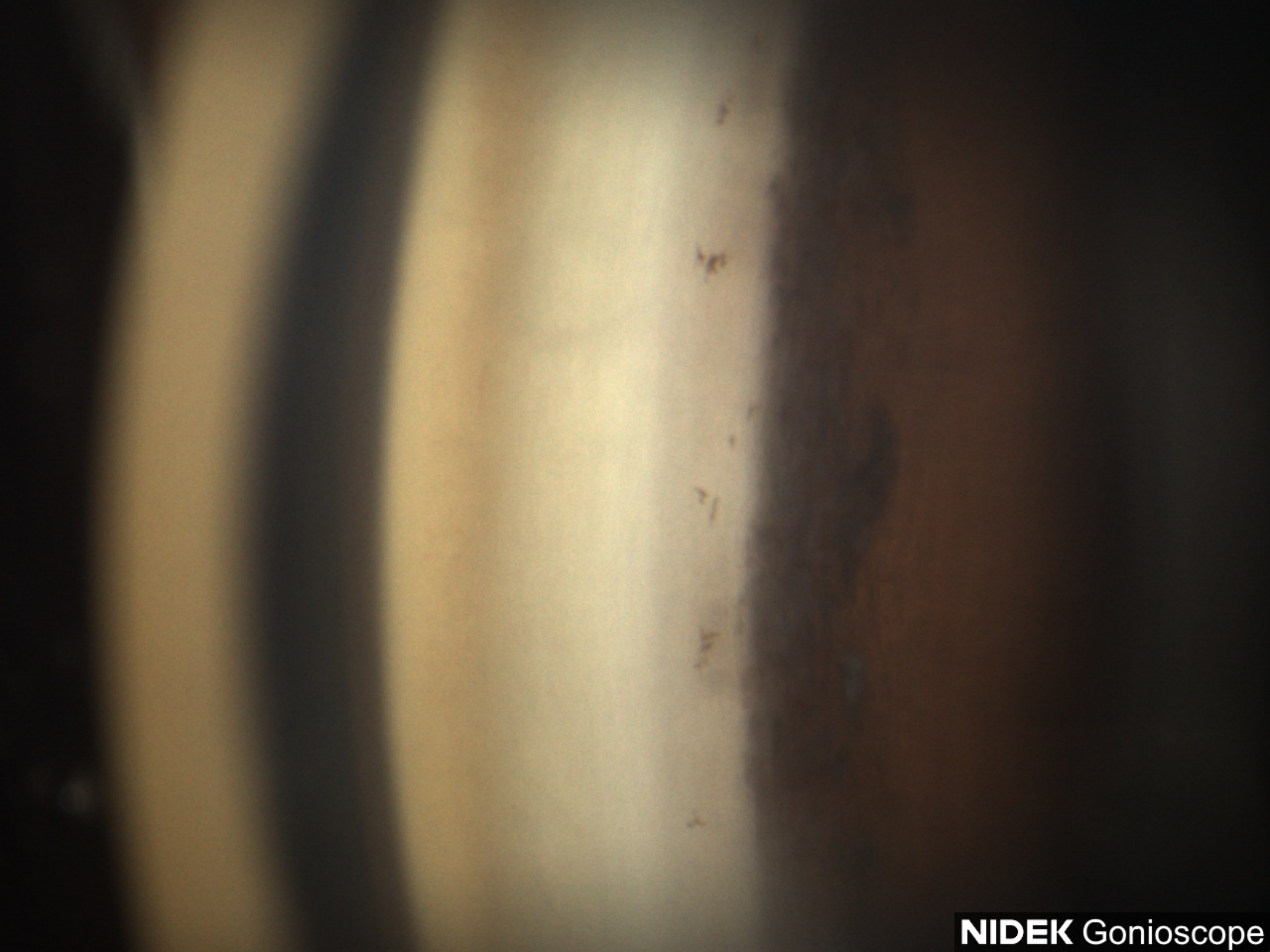

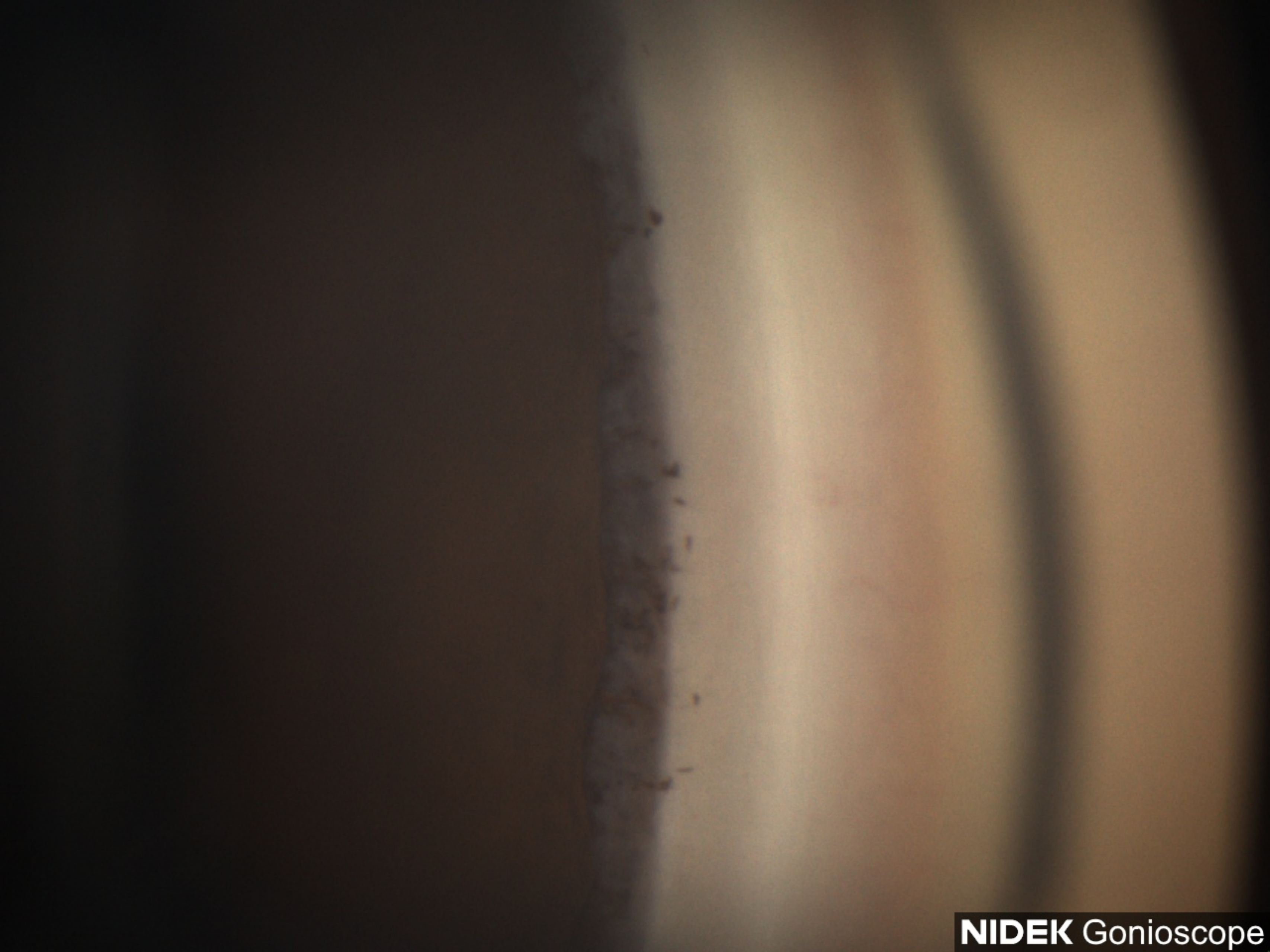

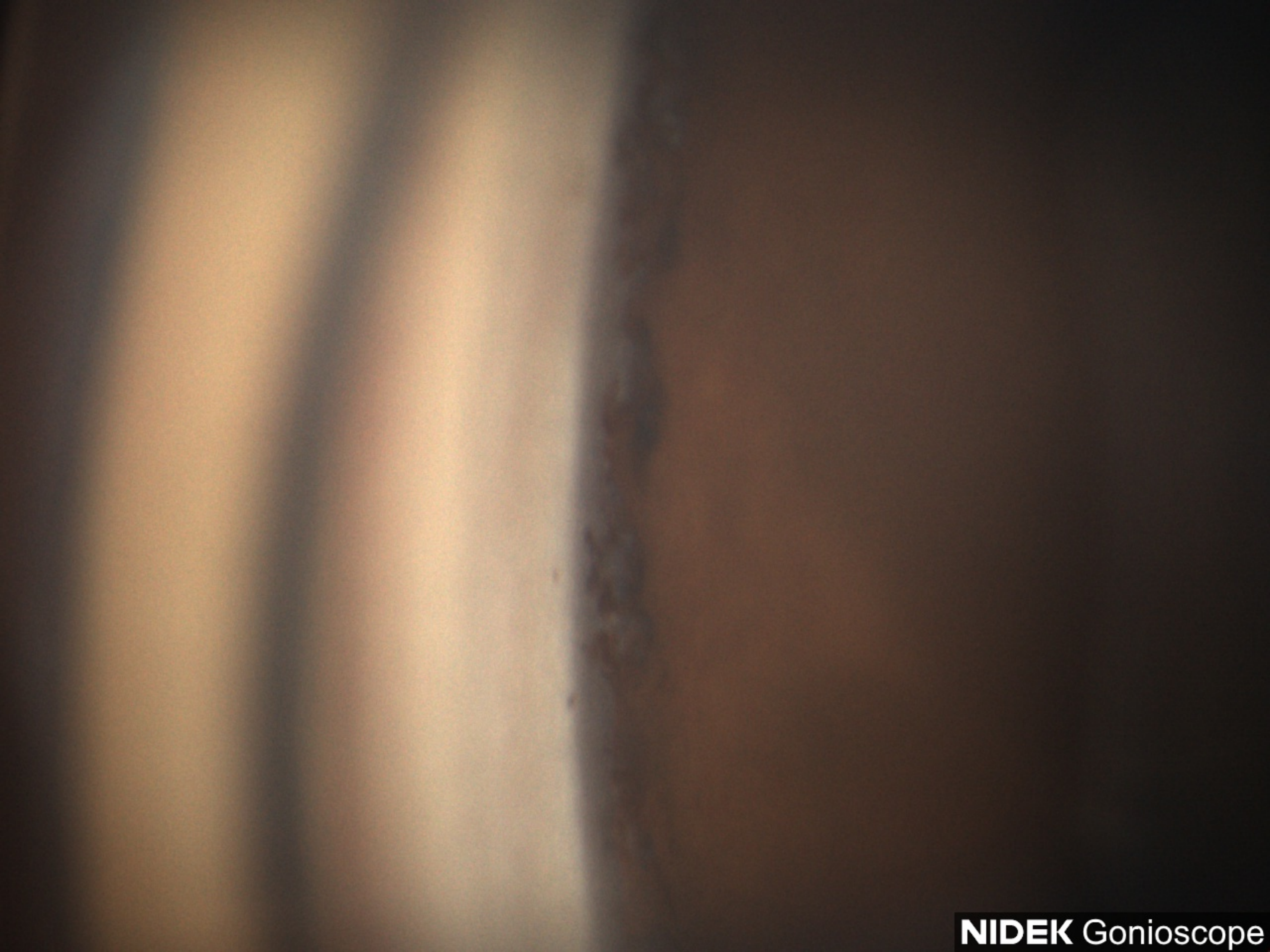

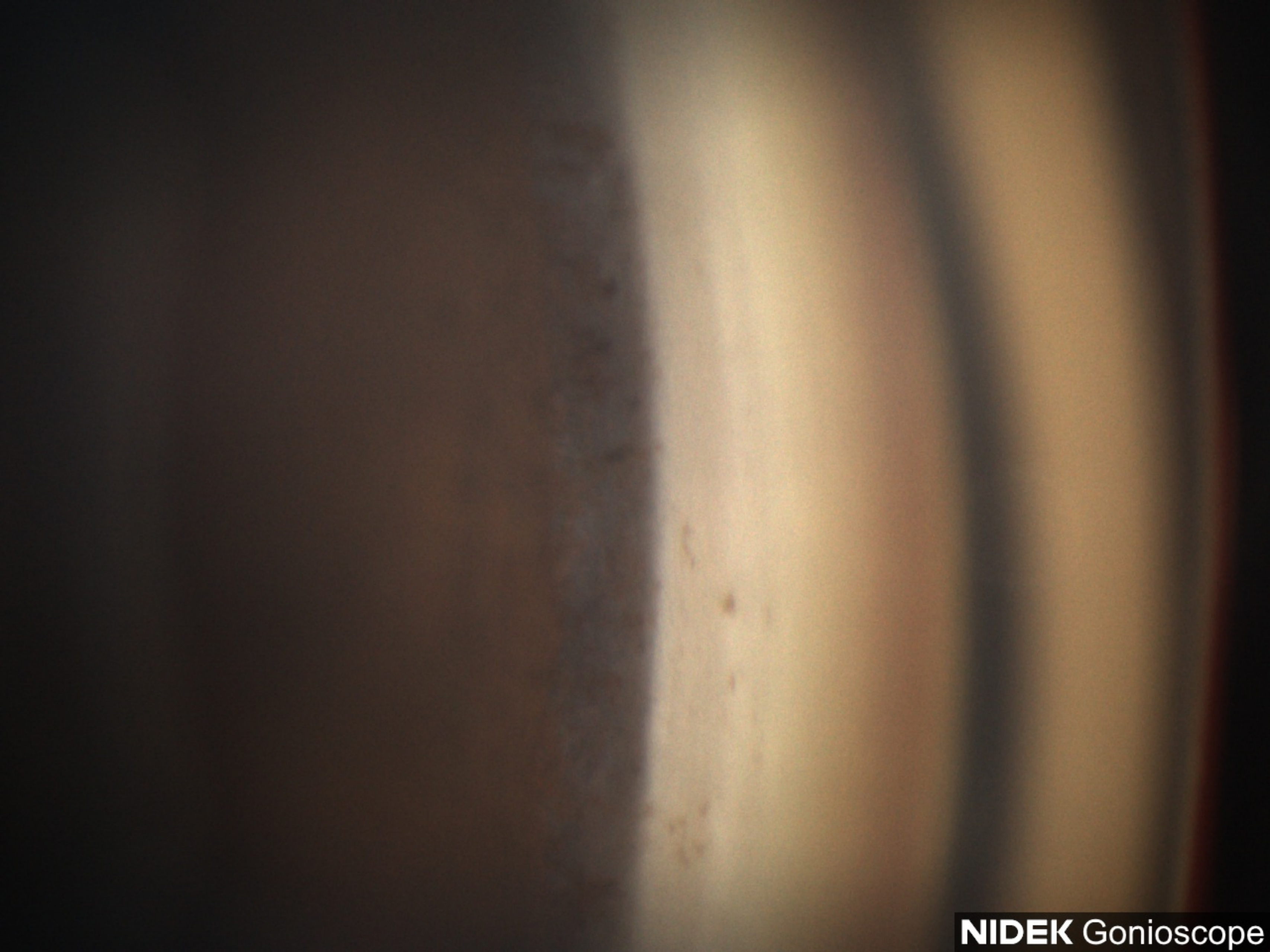

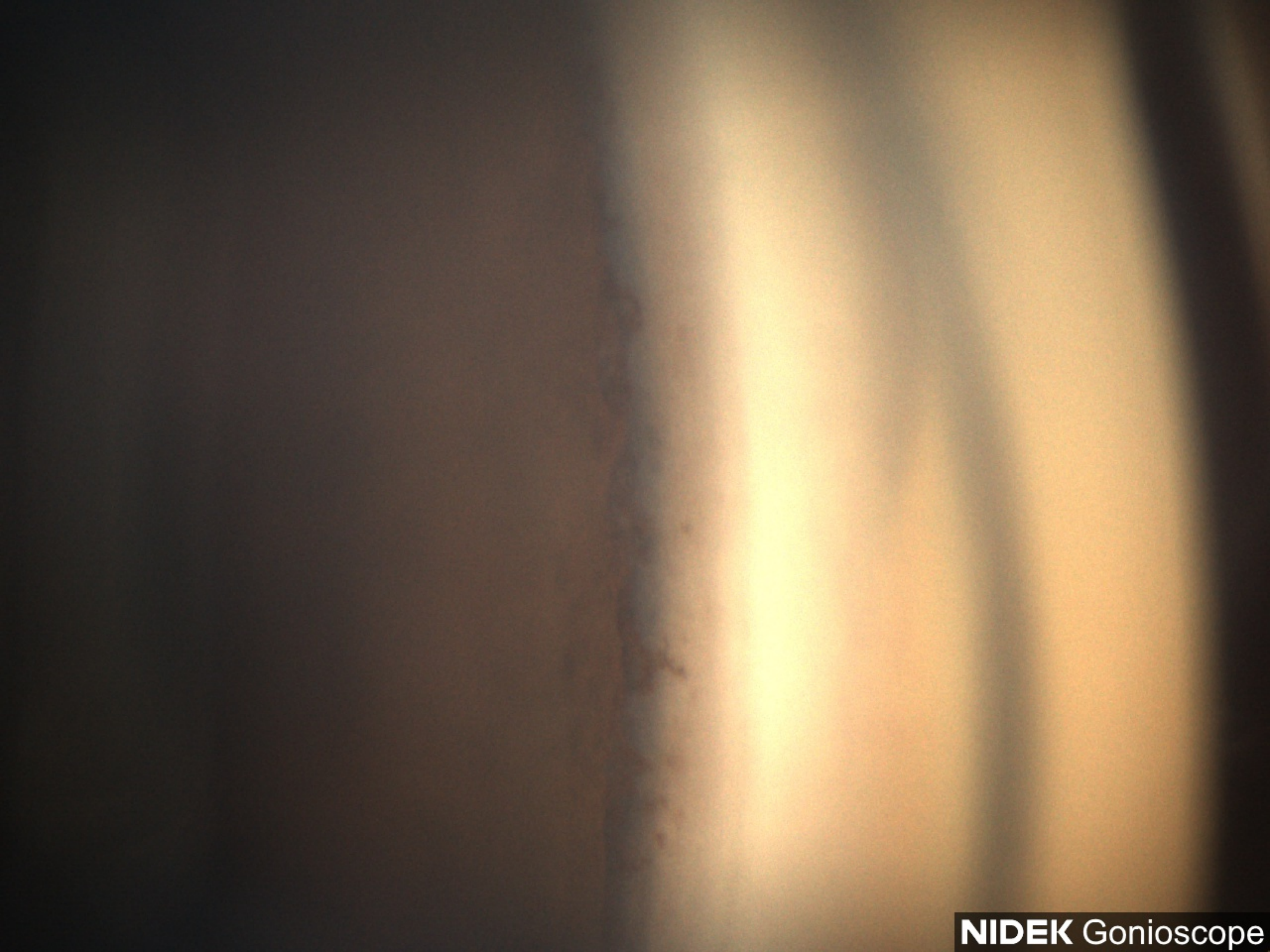

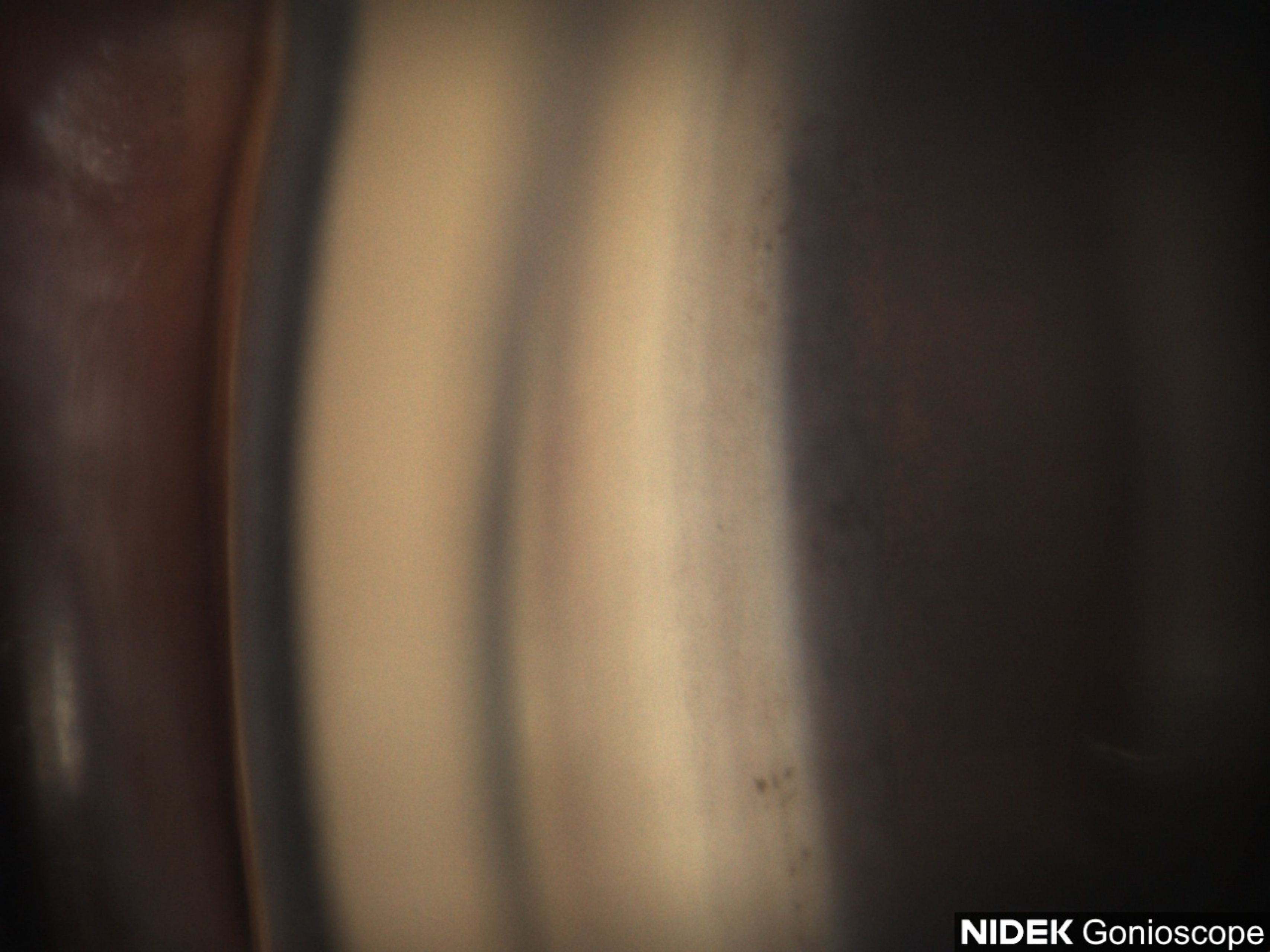

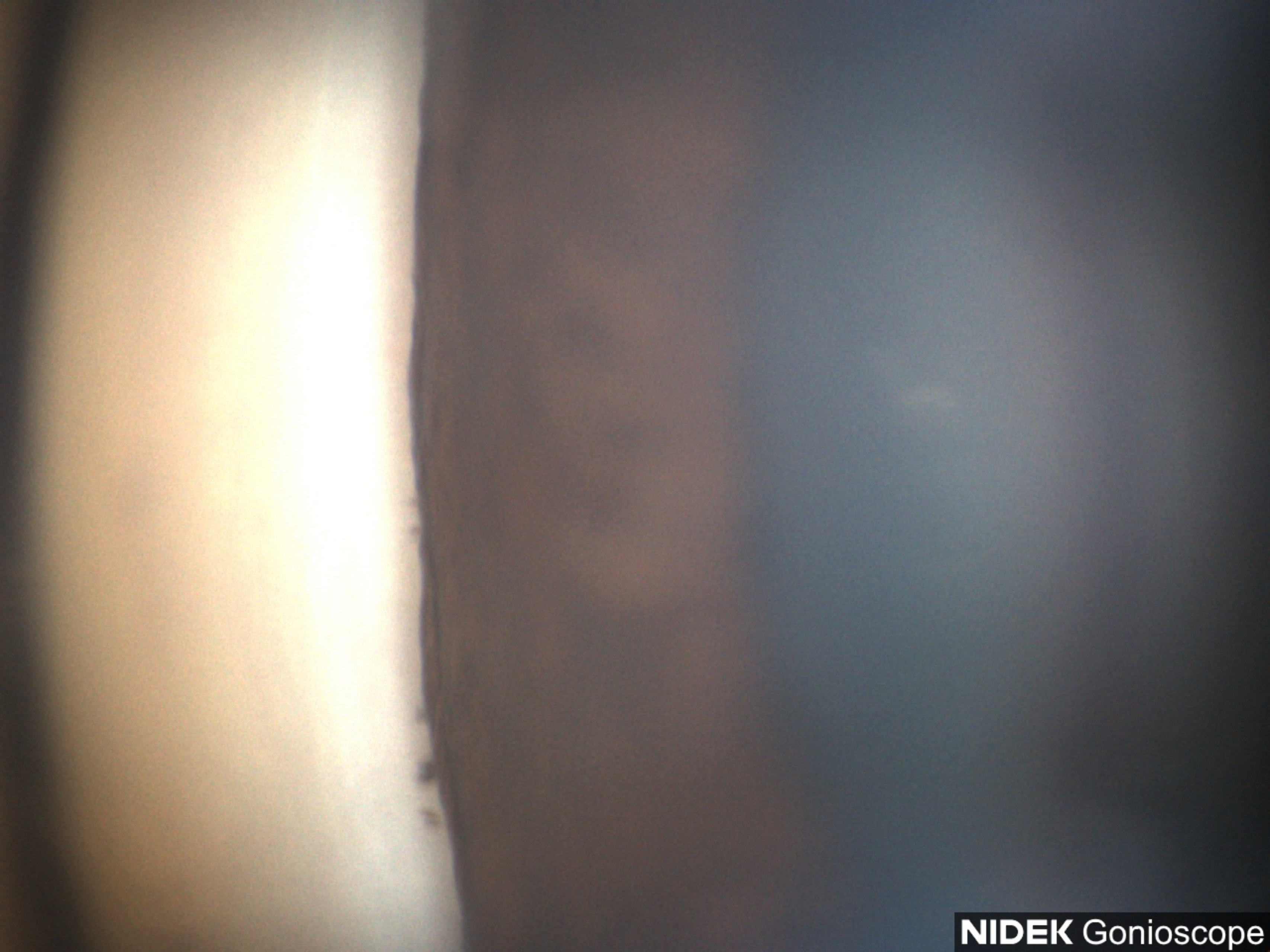

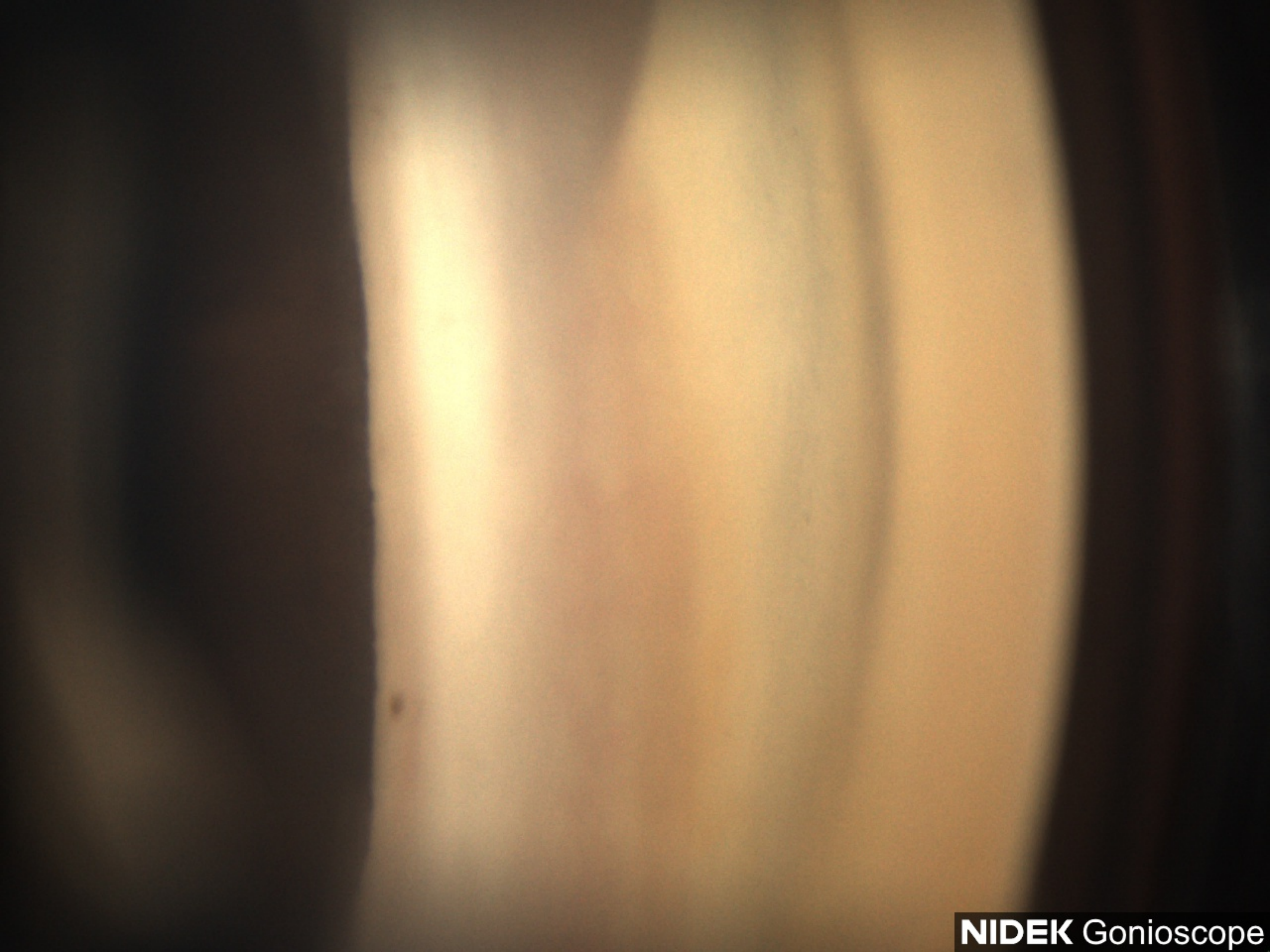

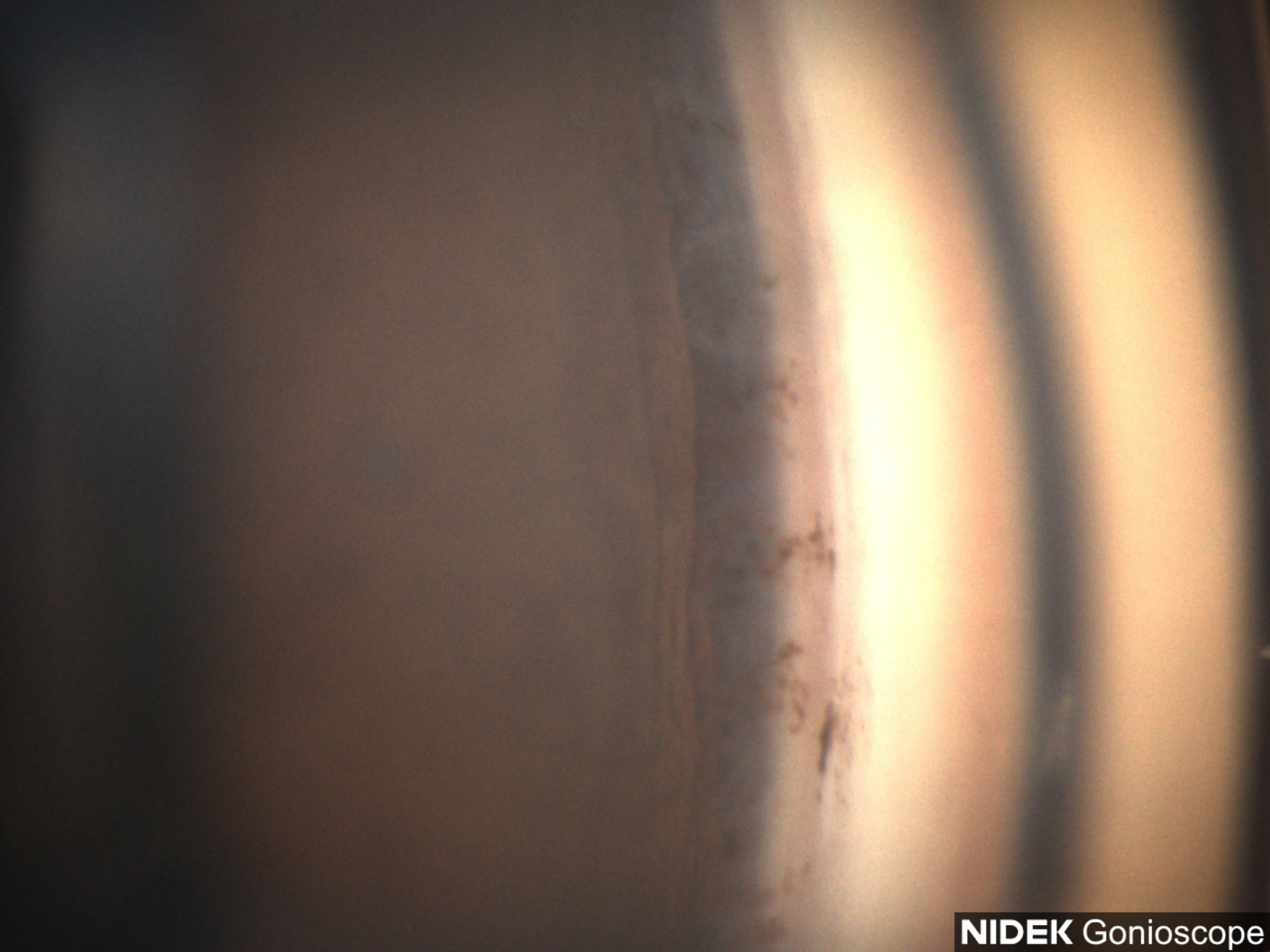

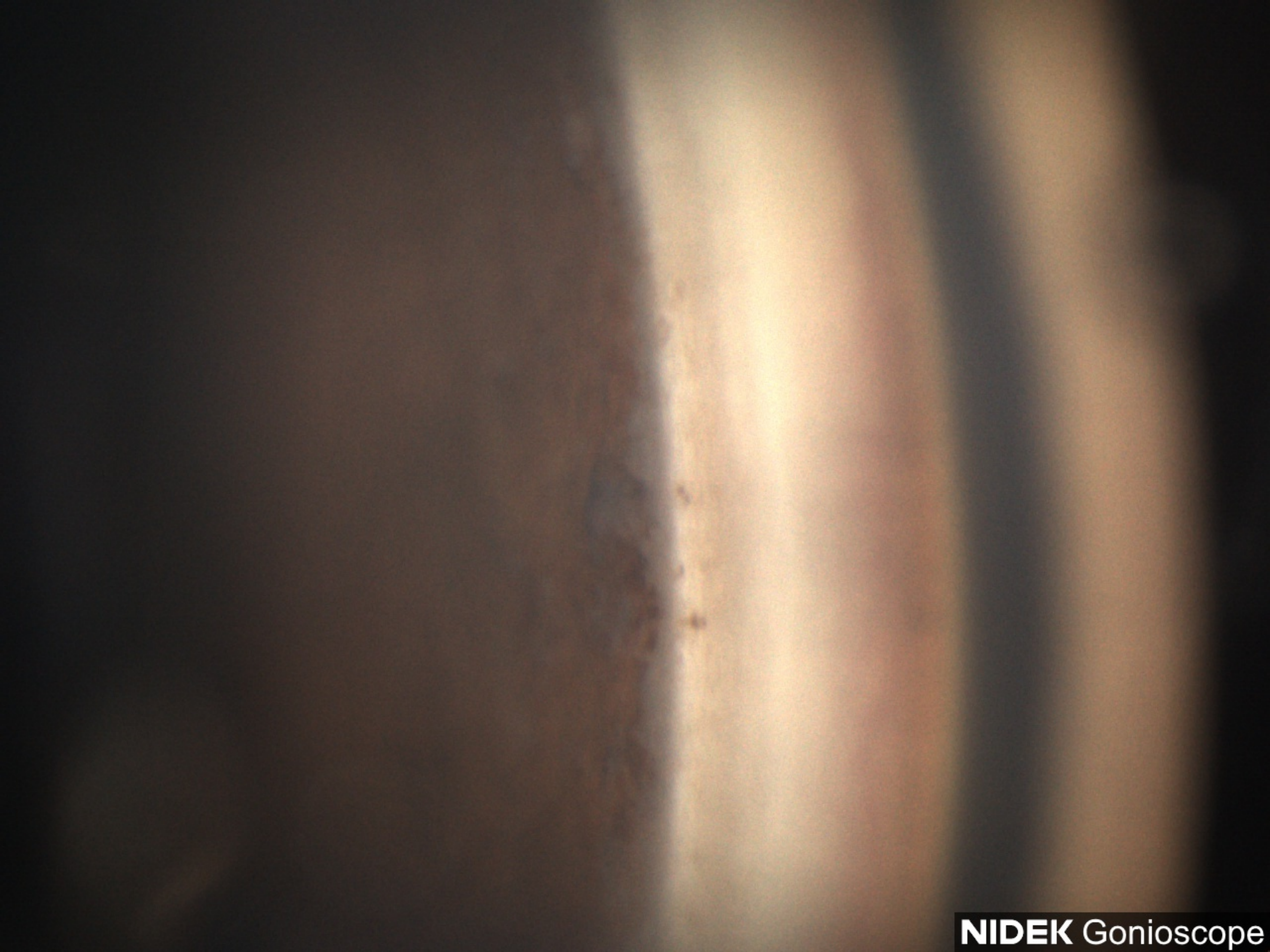

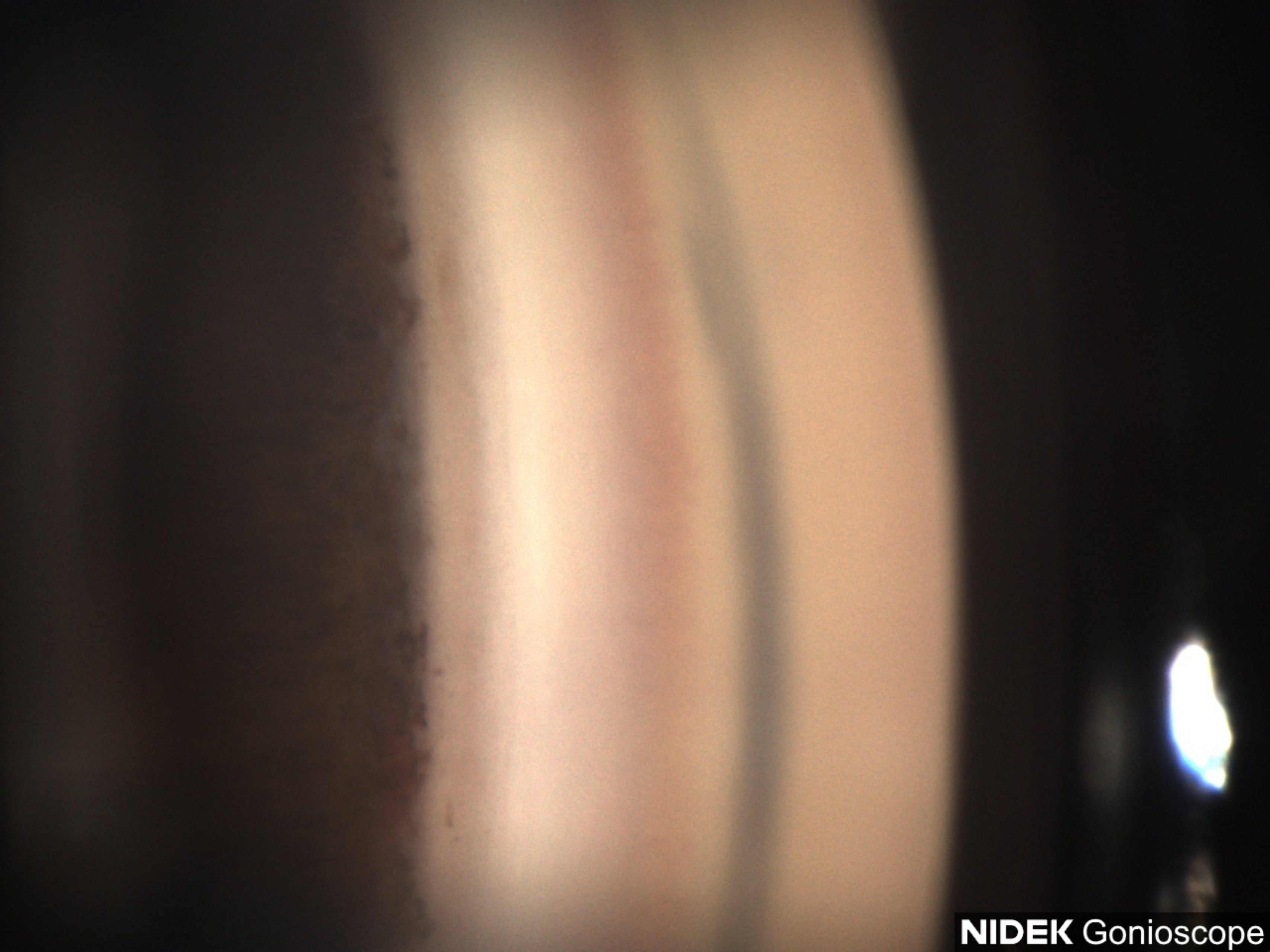

**NIDEK** Gonioscope

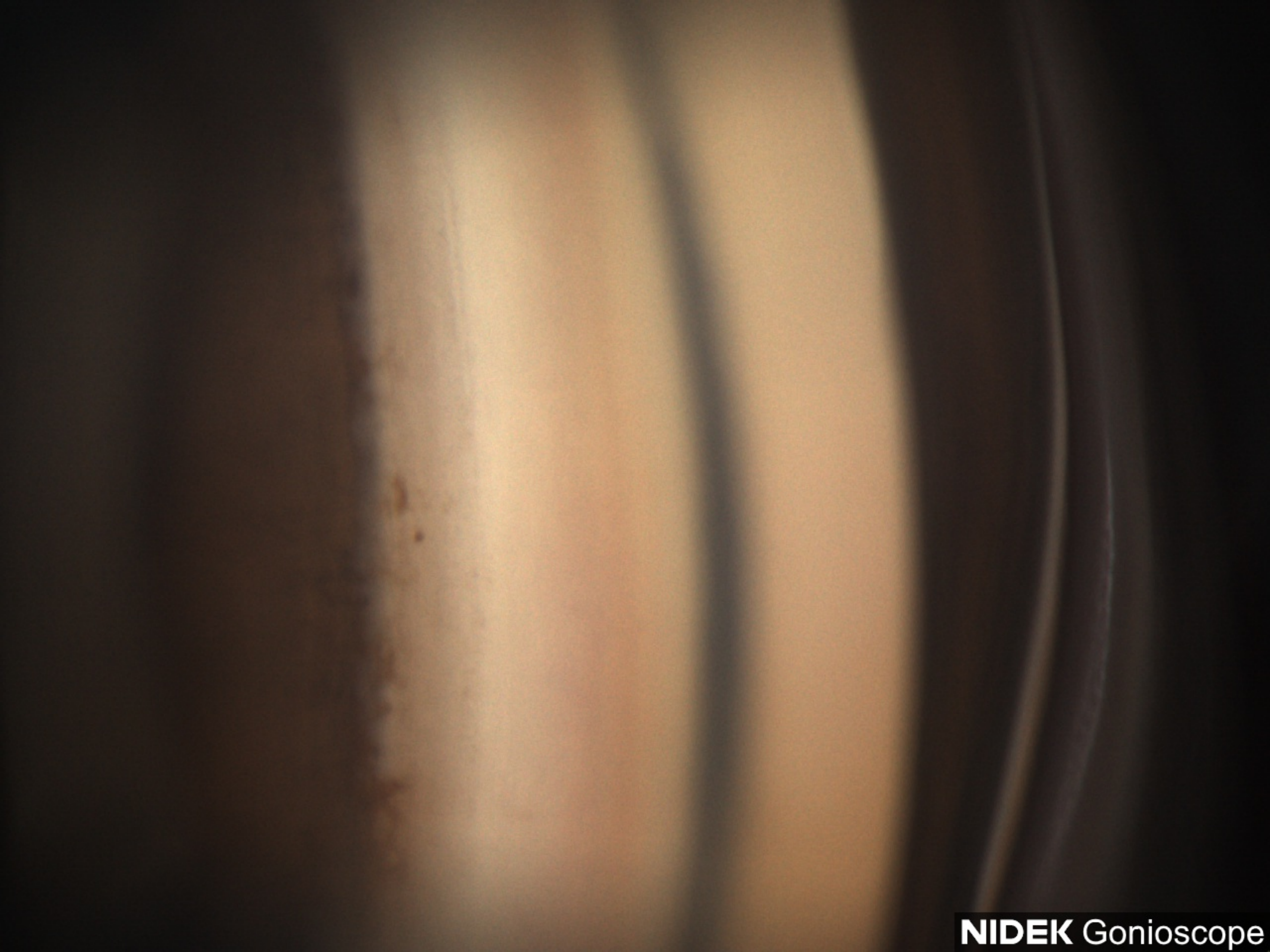



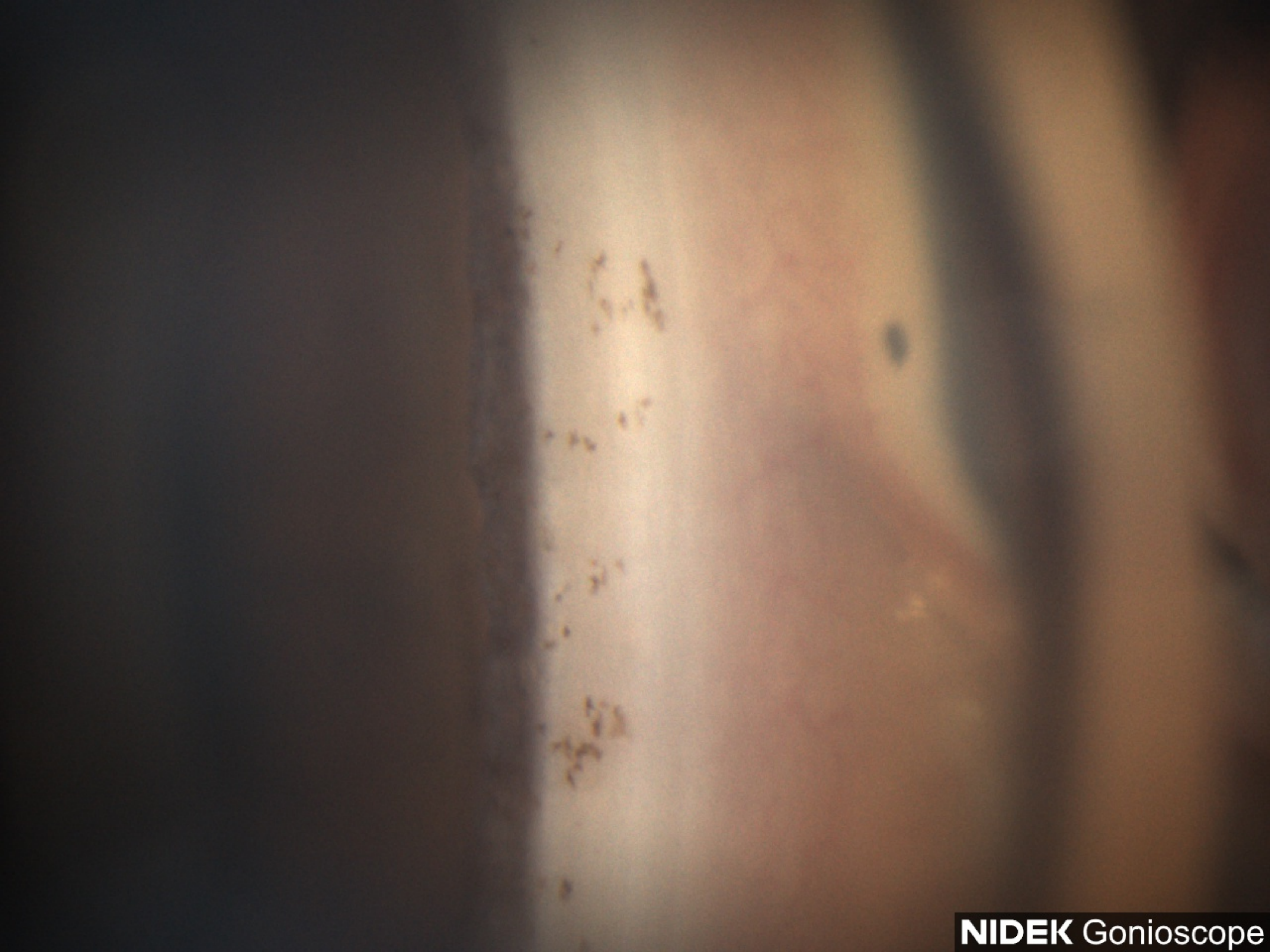



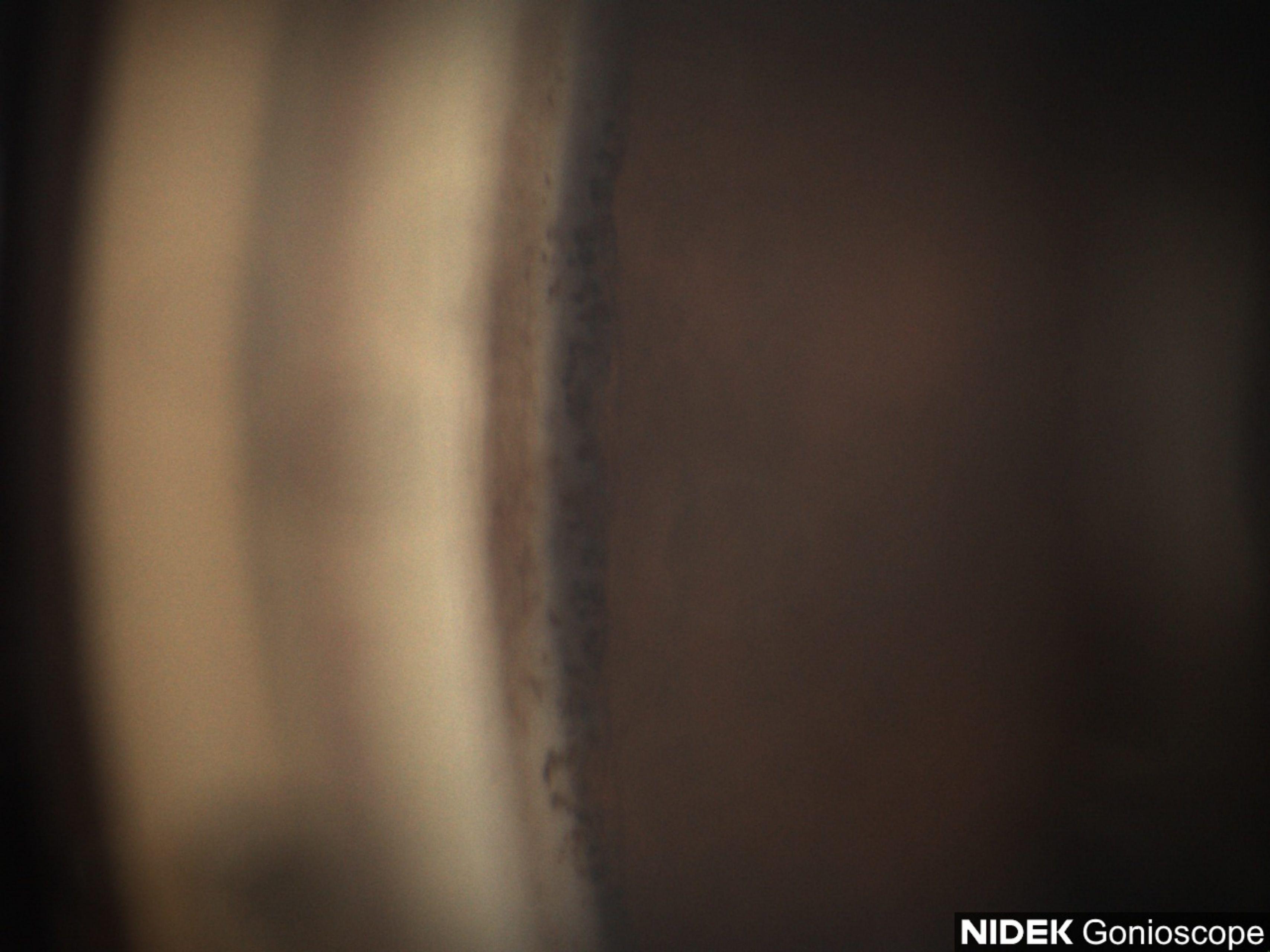

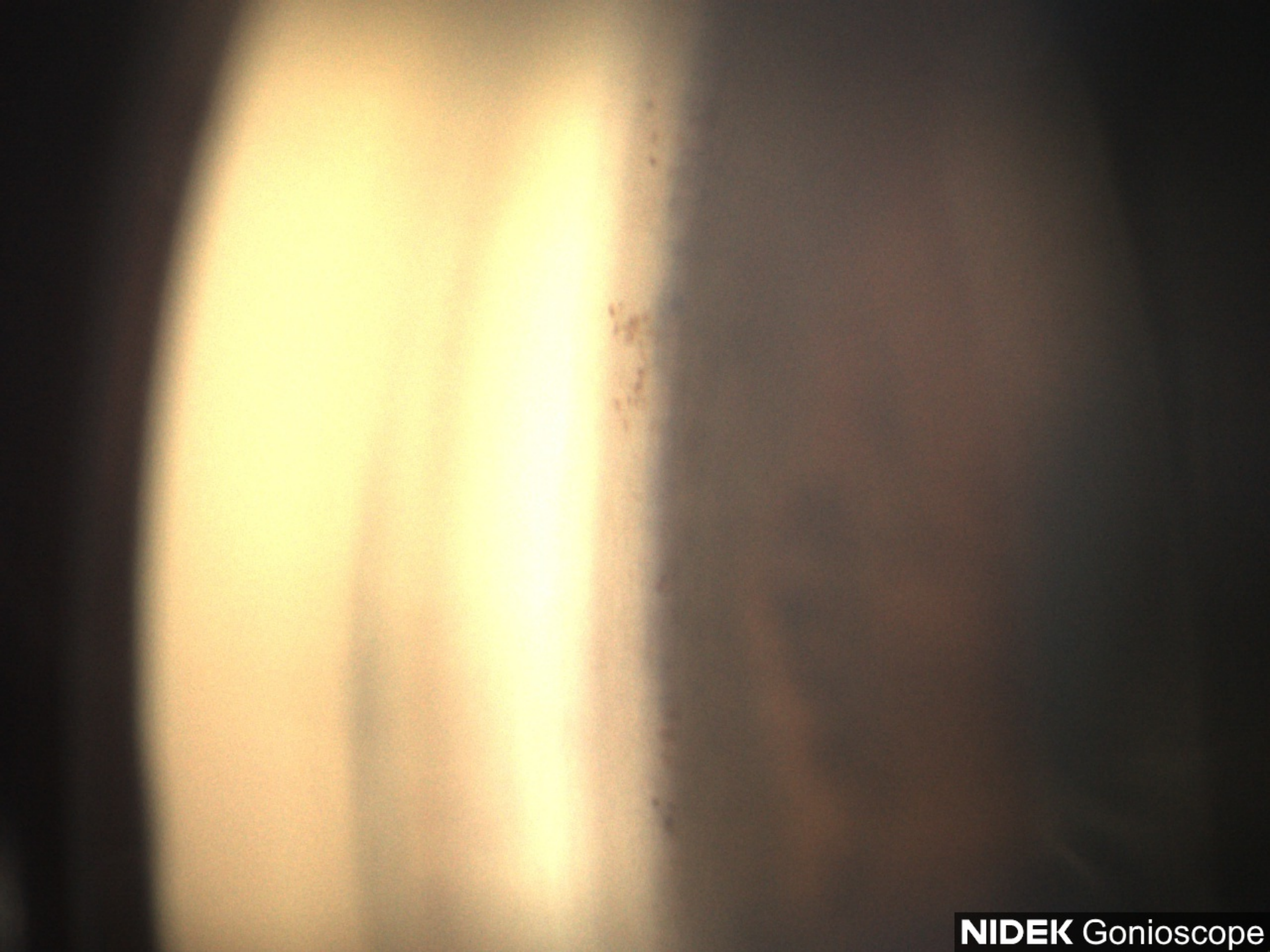

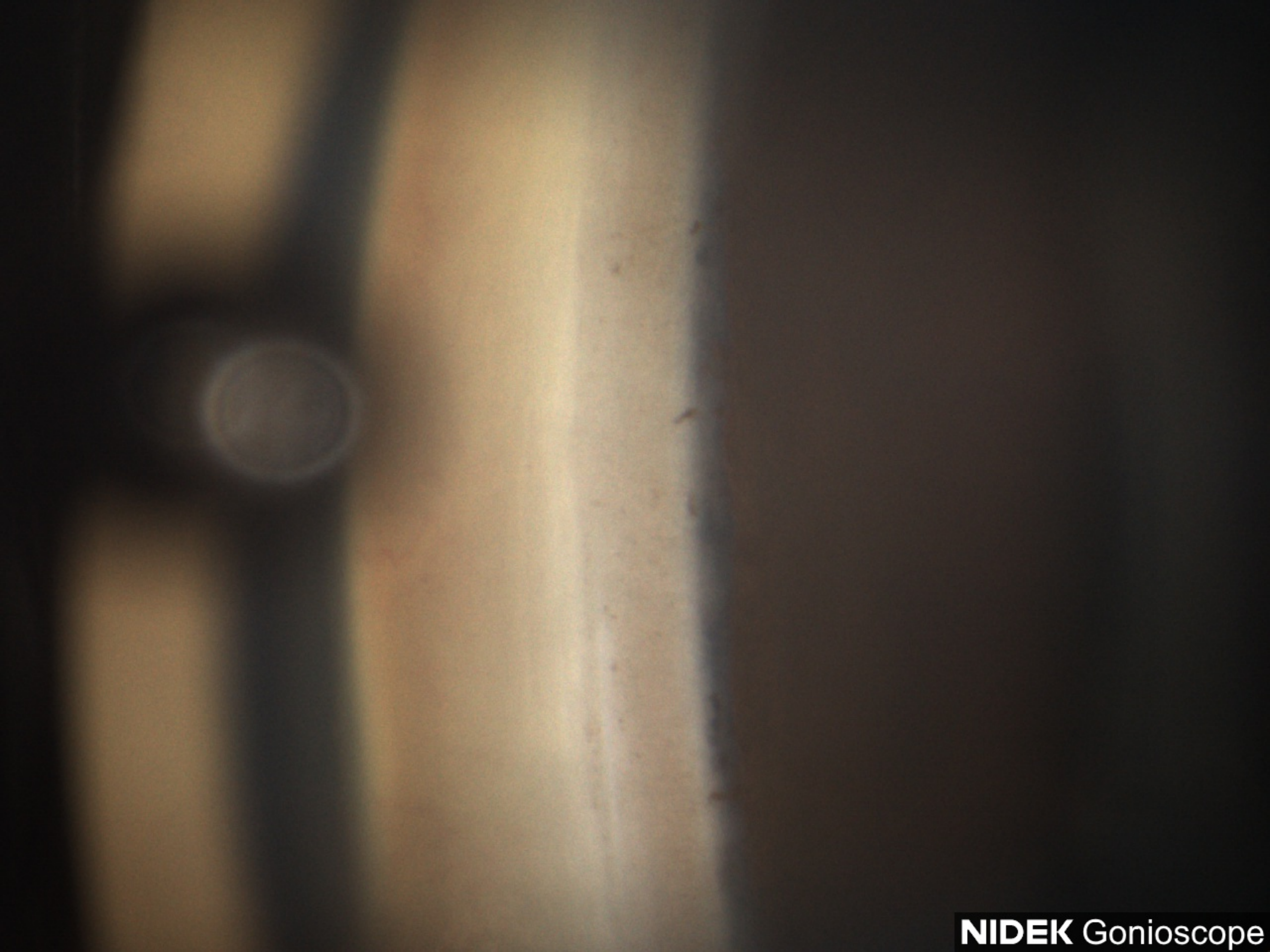

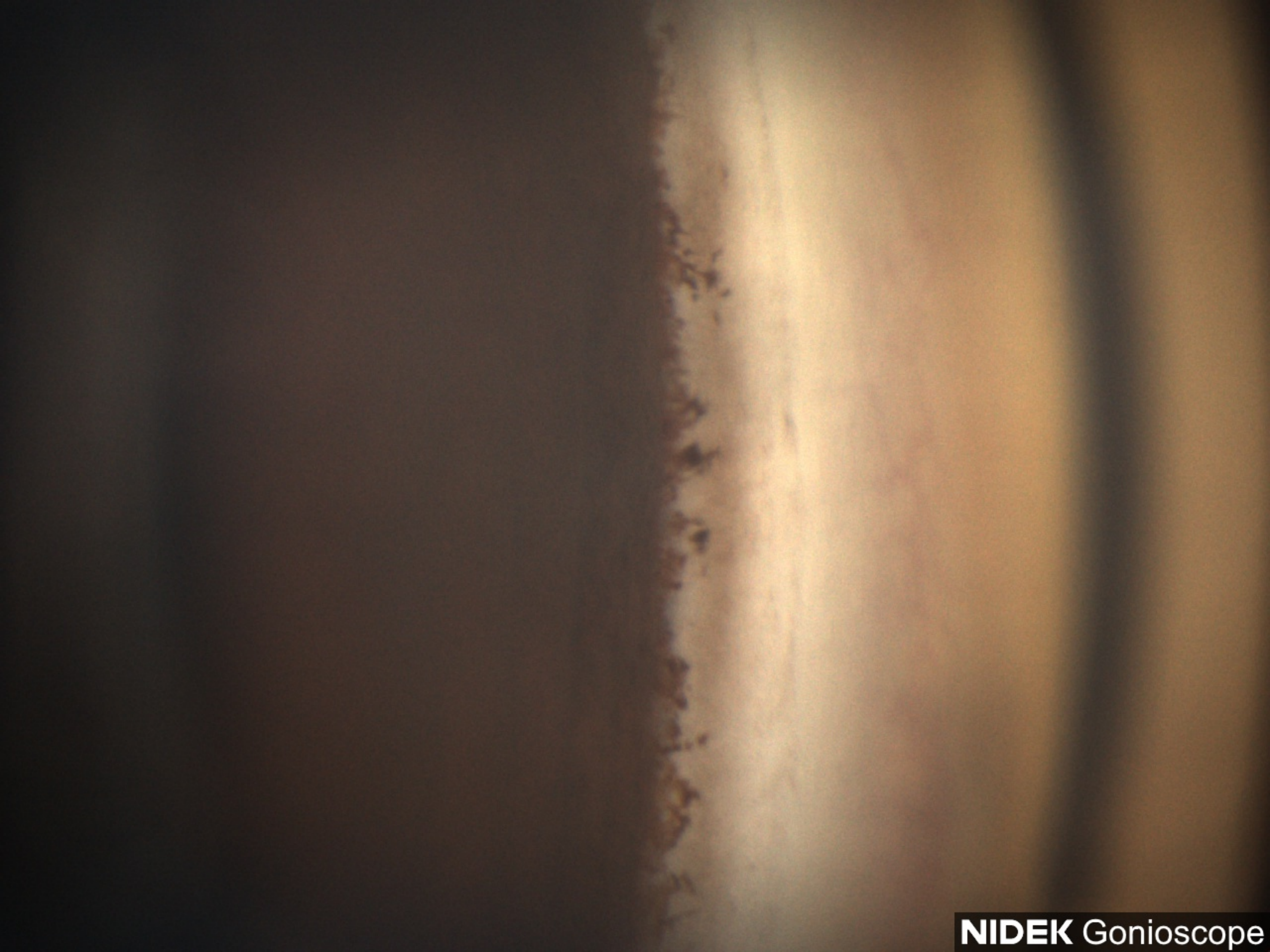



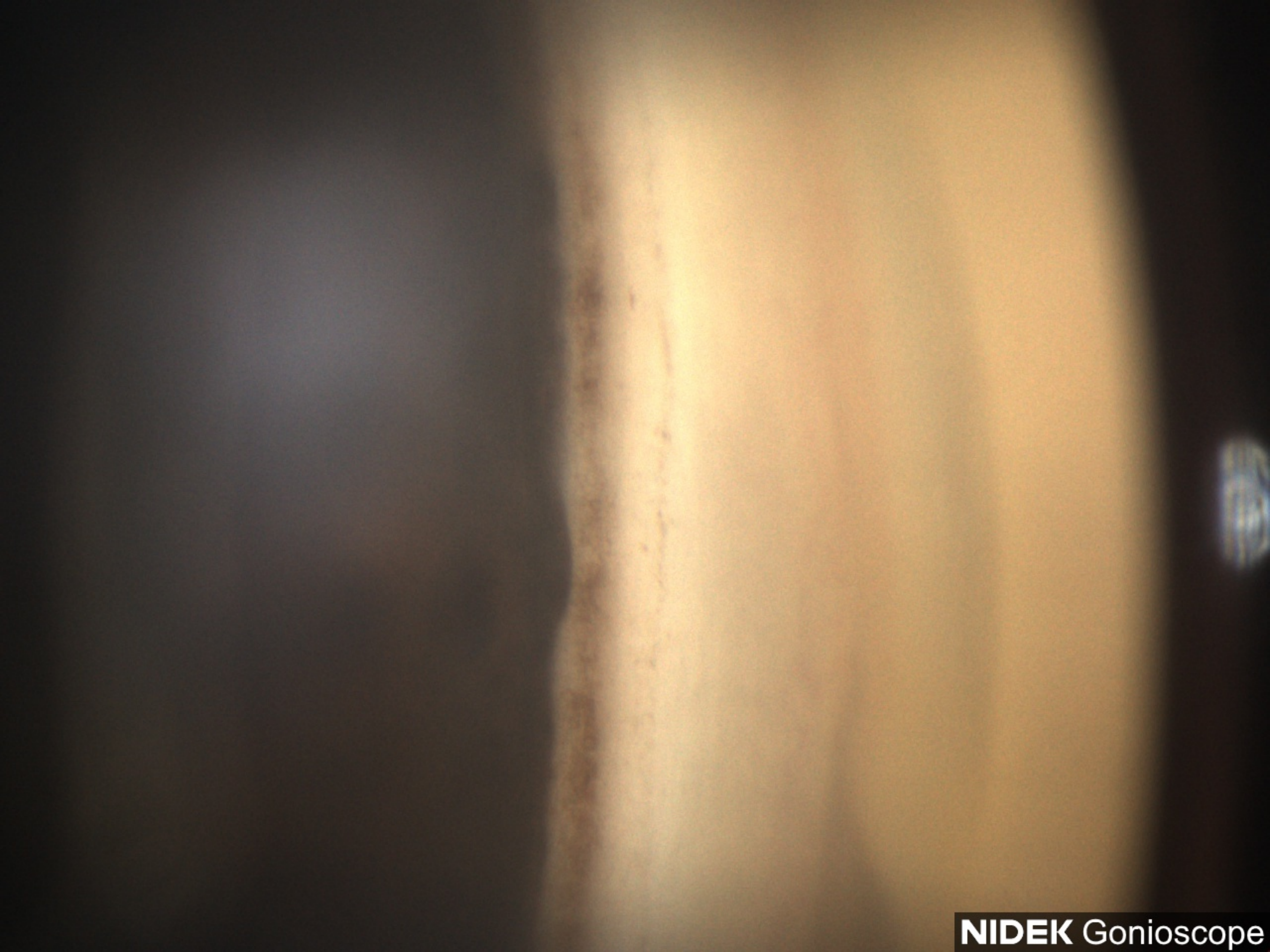

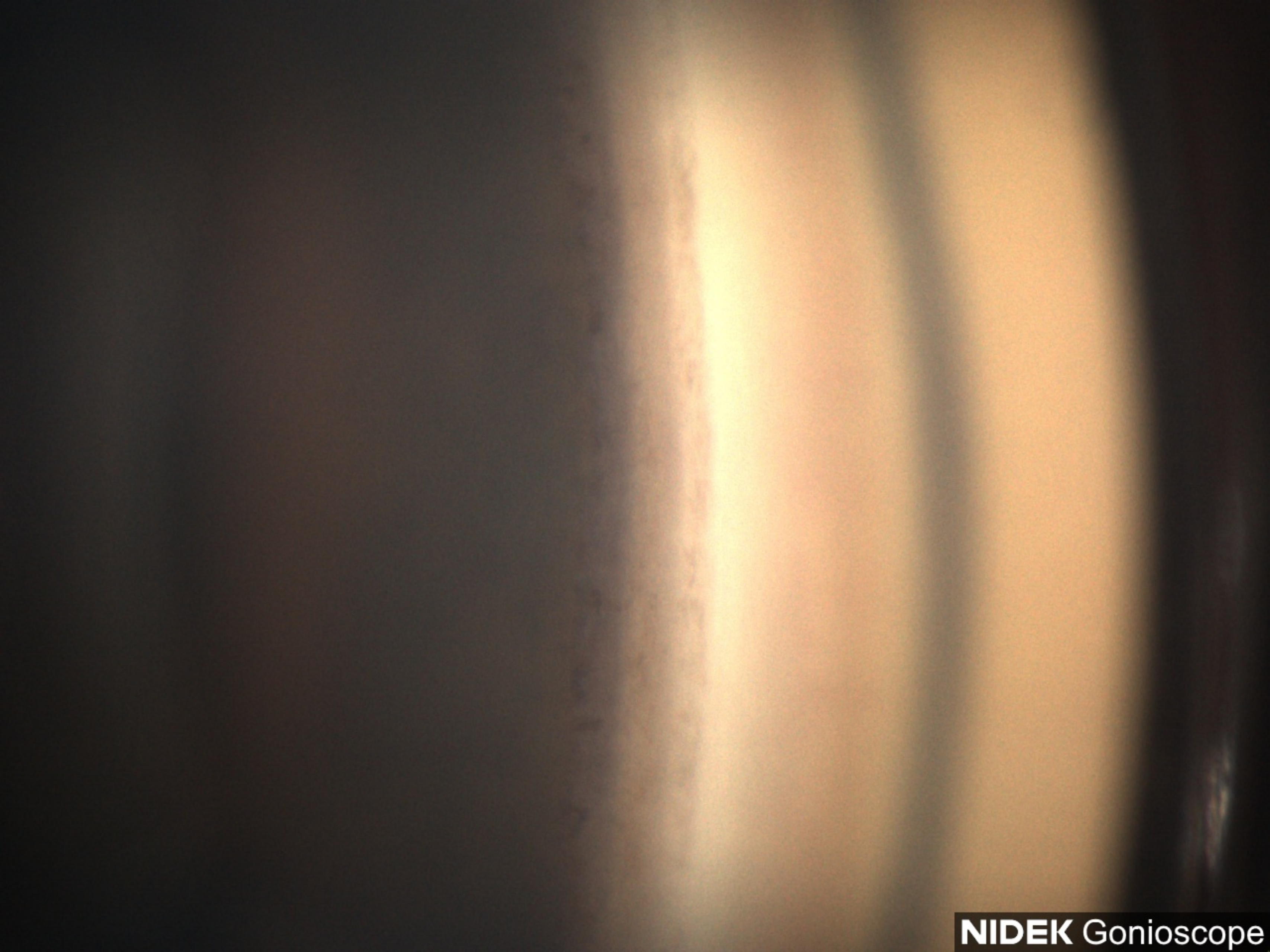

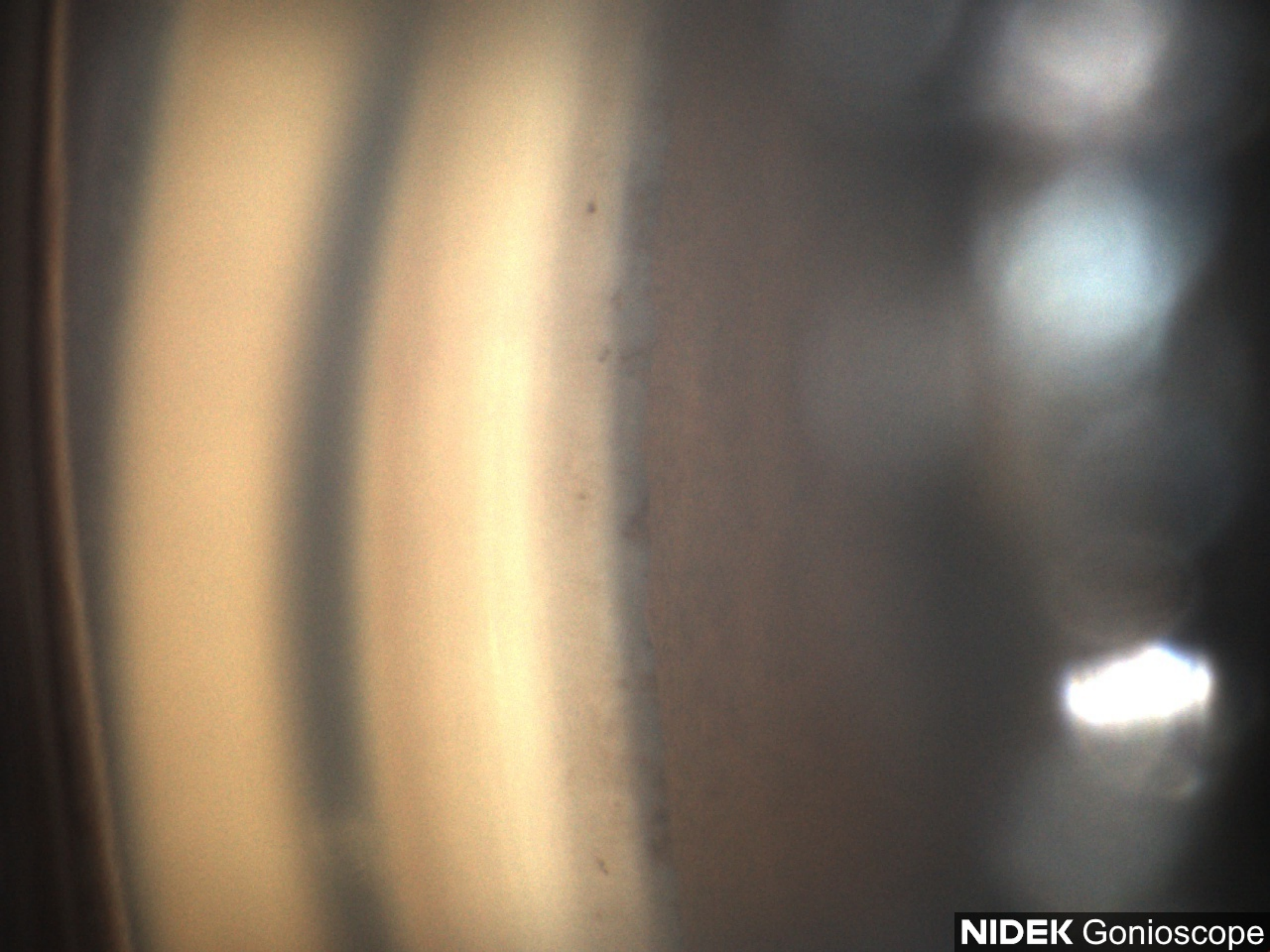

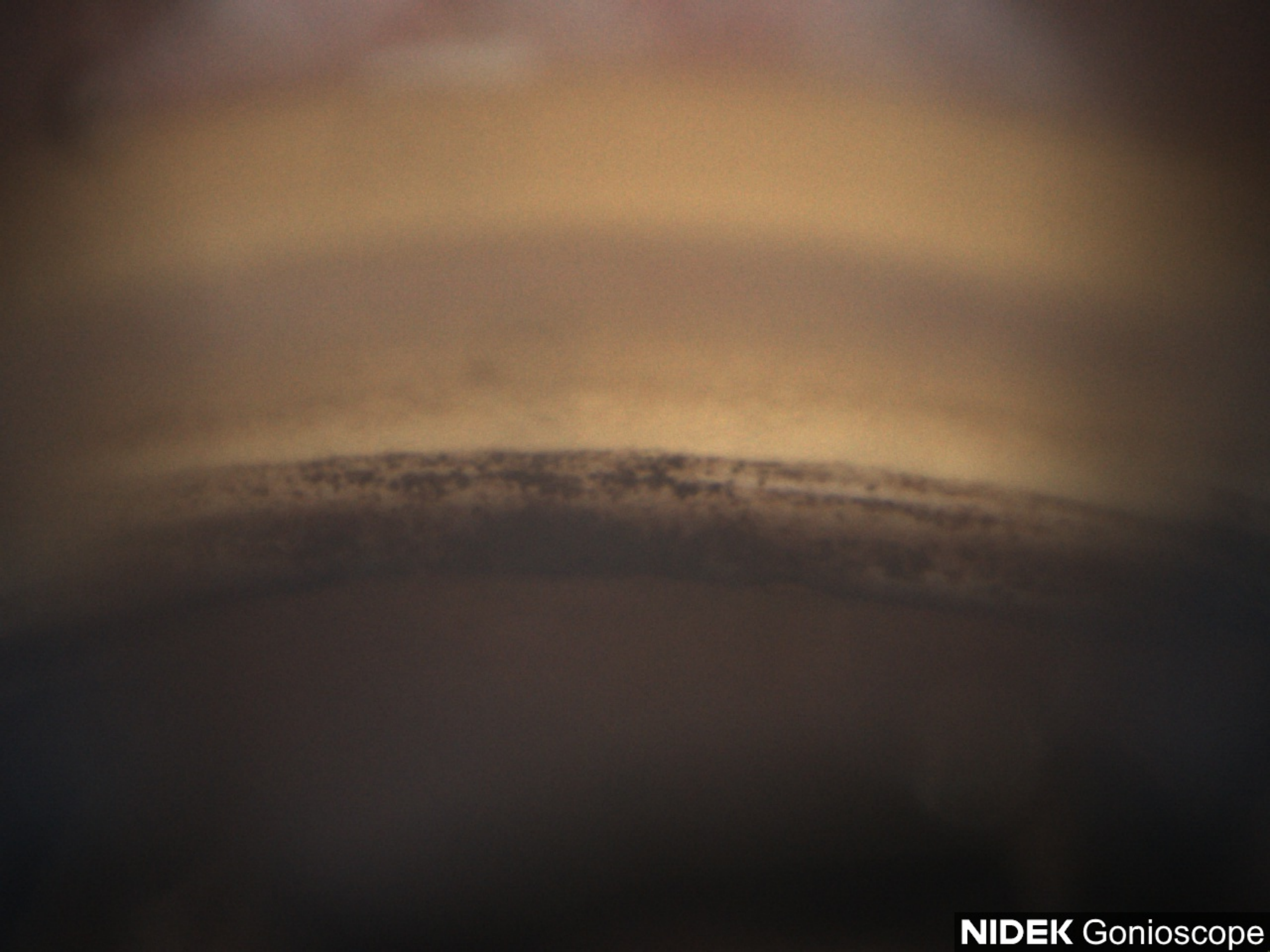

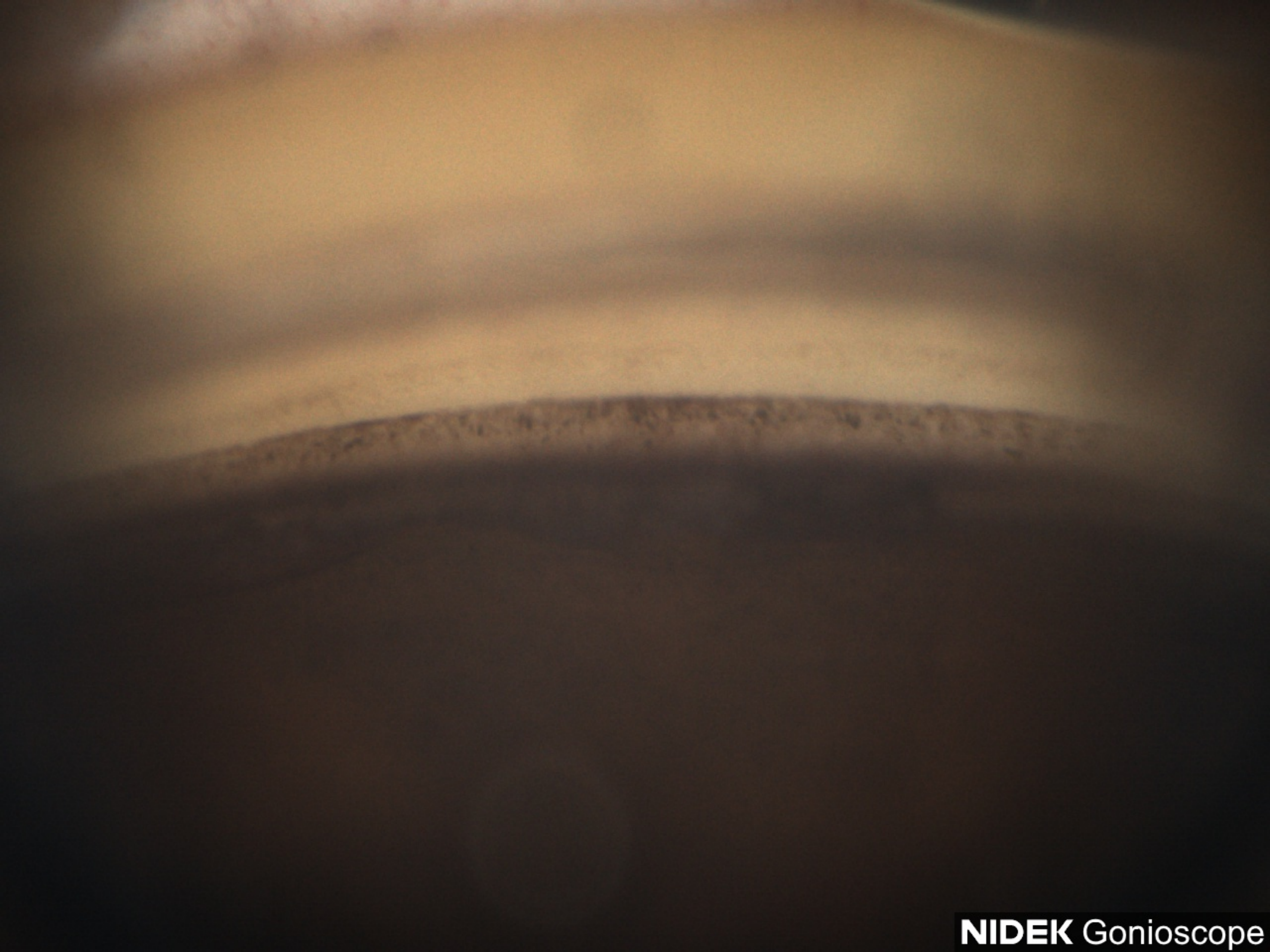

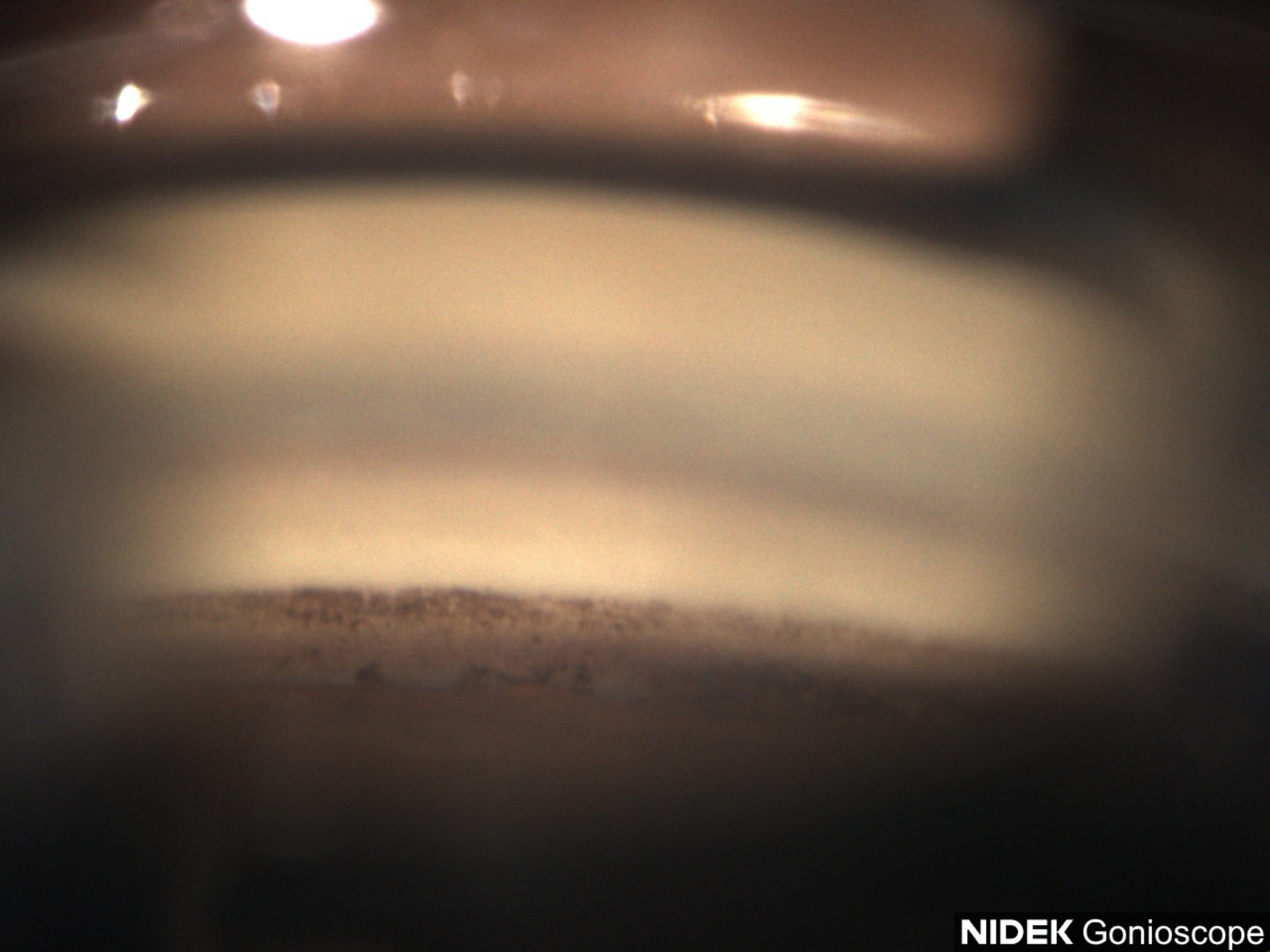

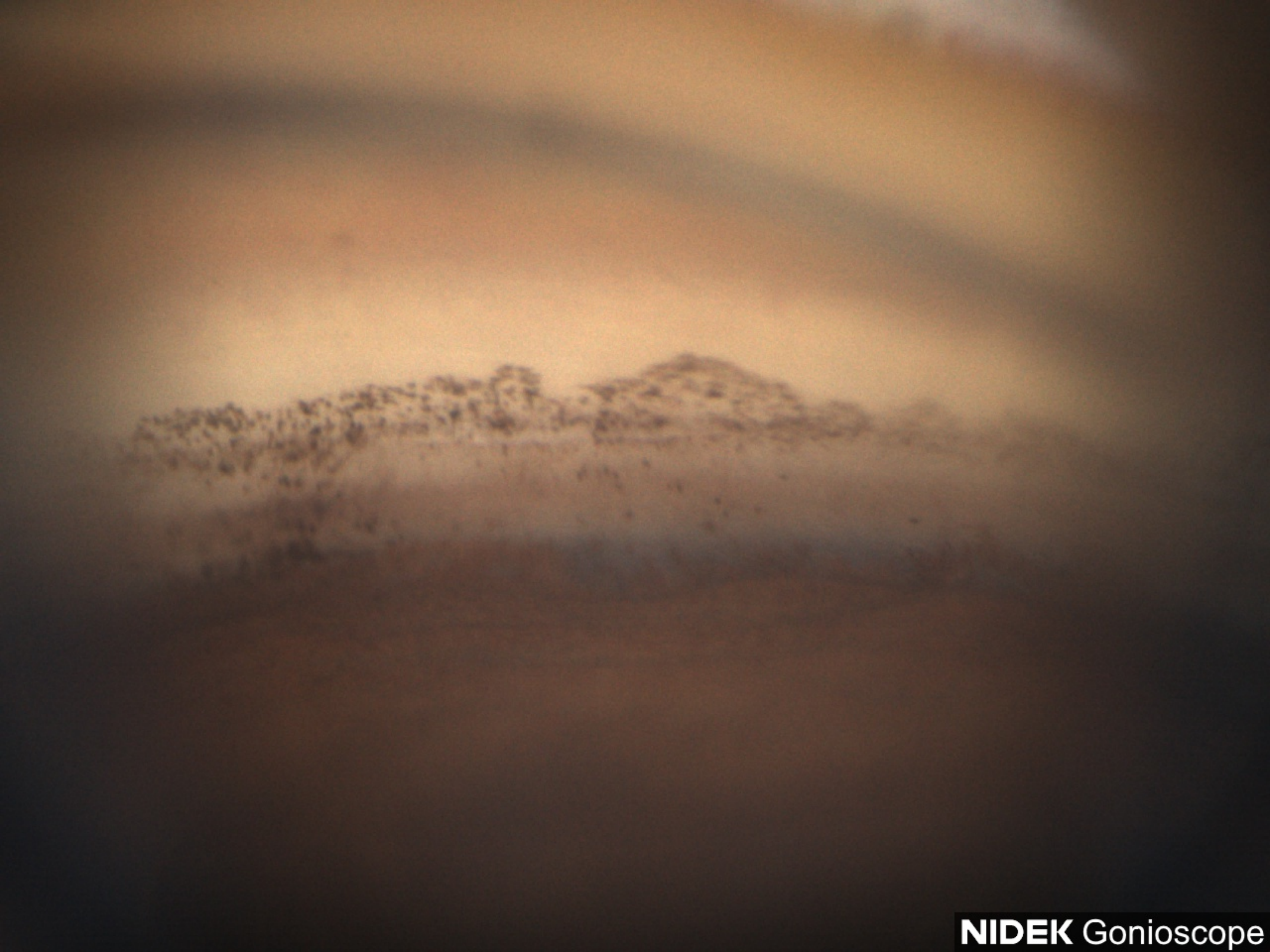

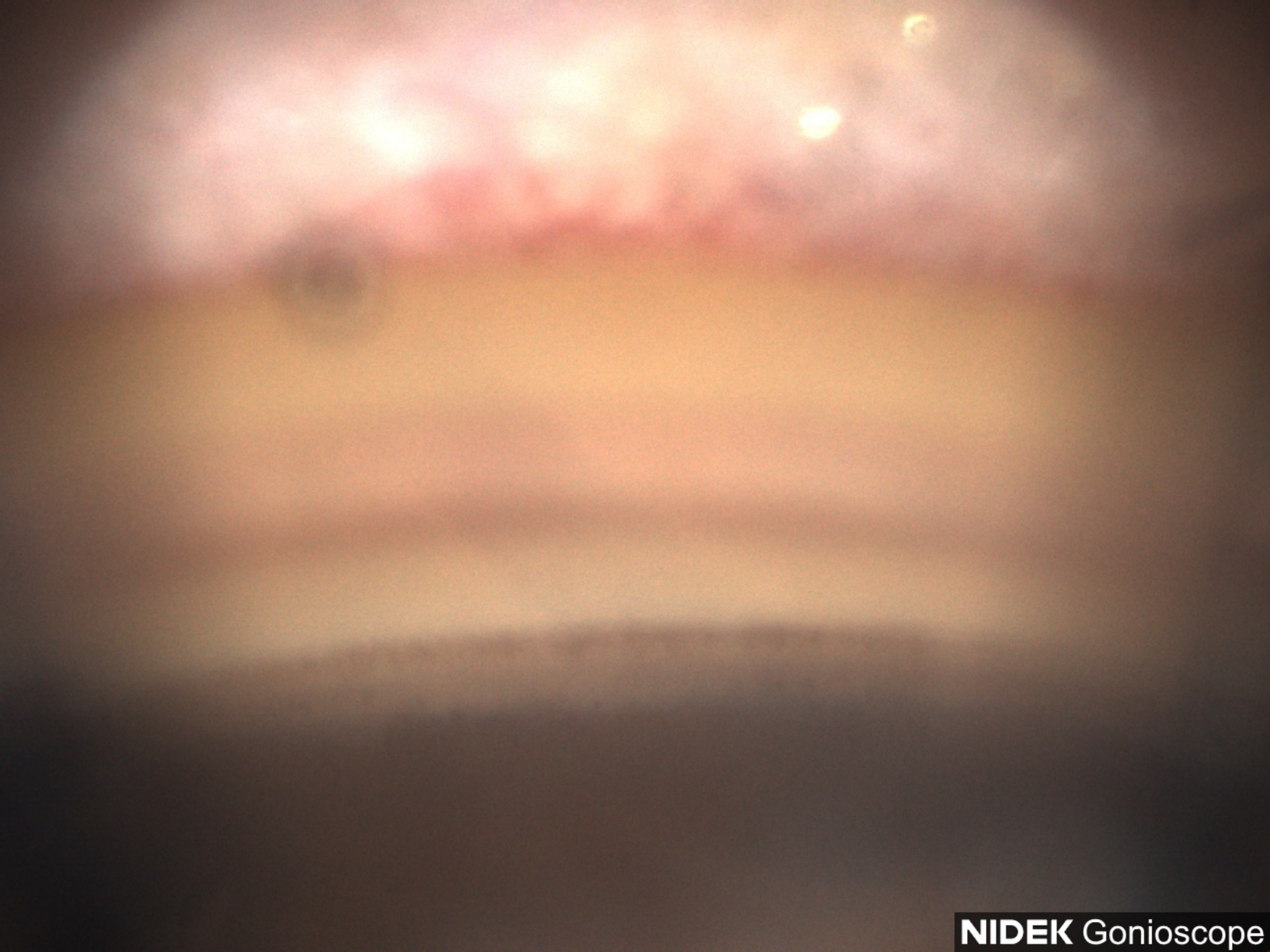

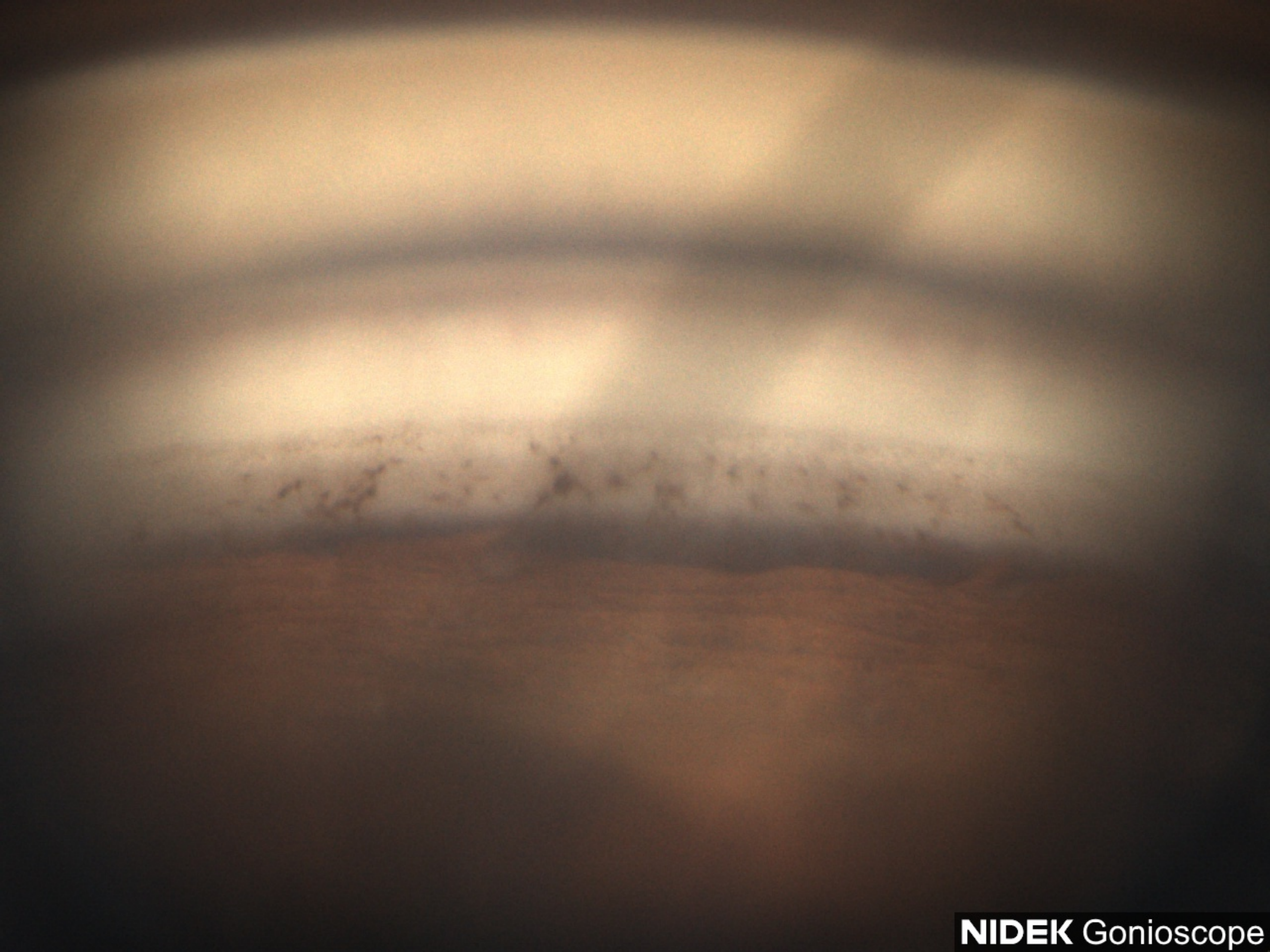

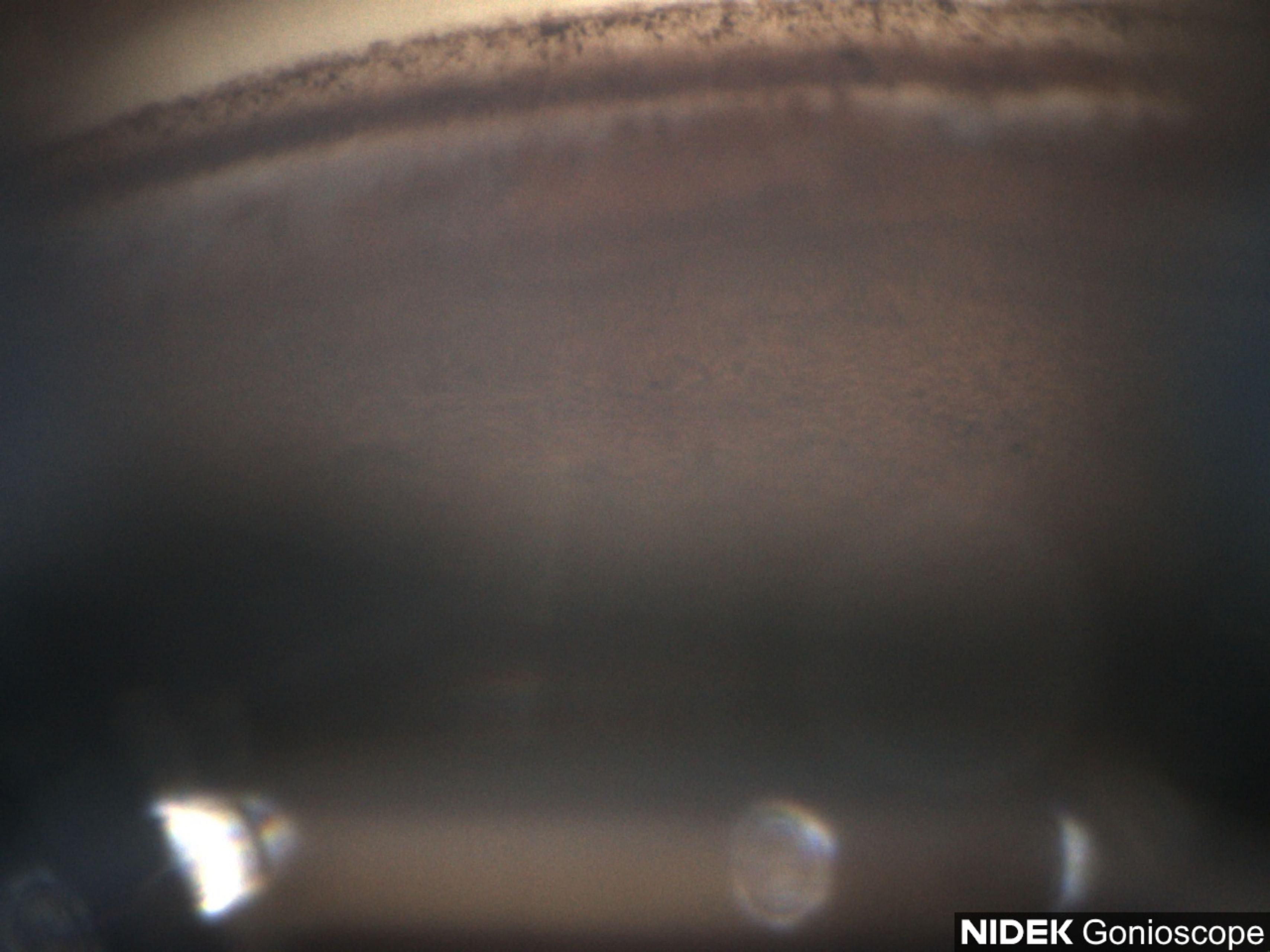

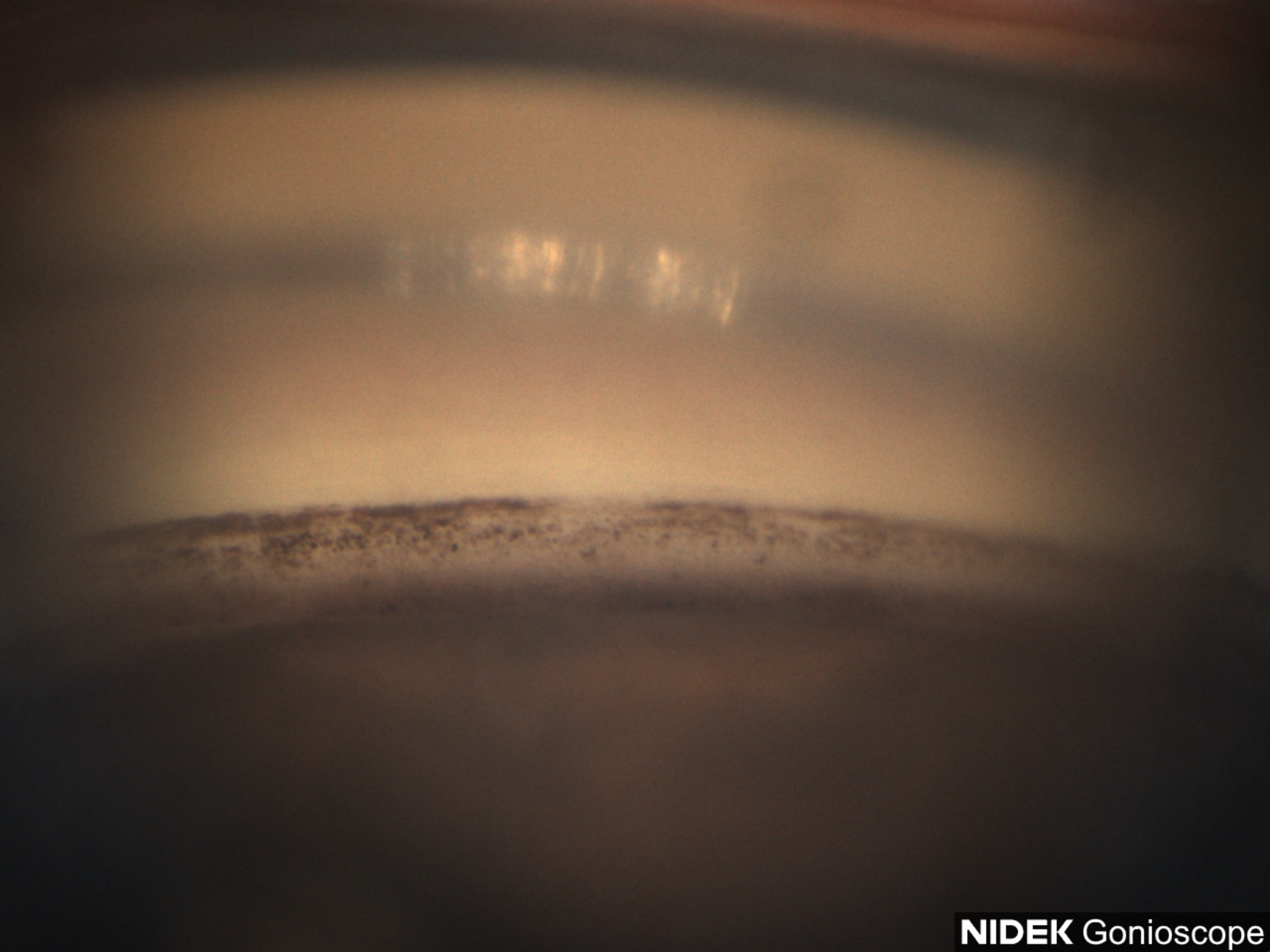

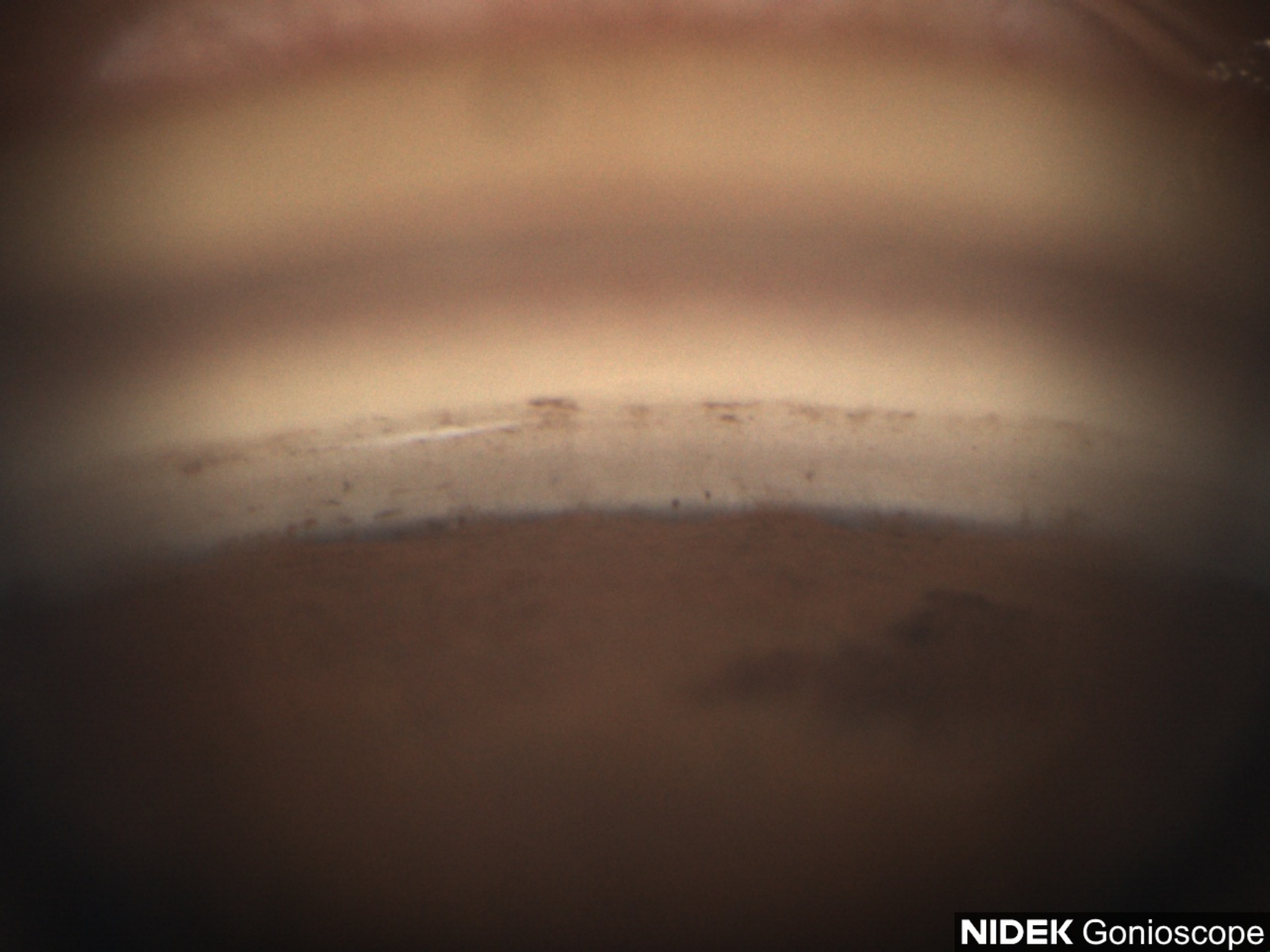

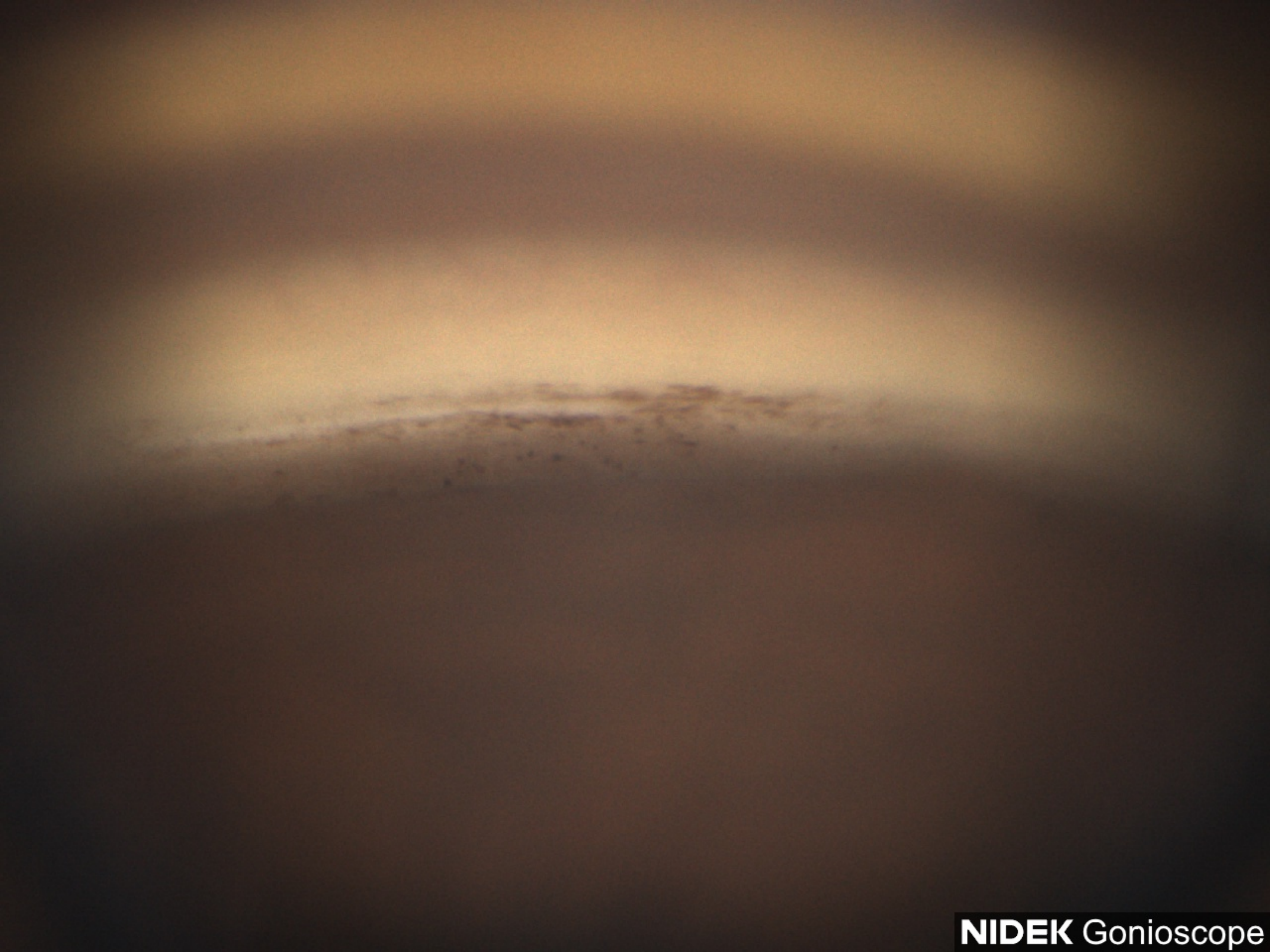

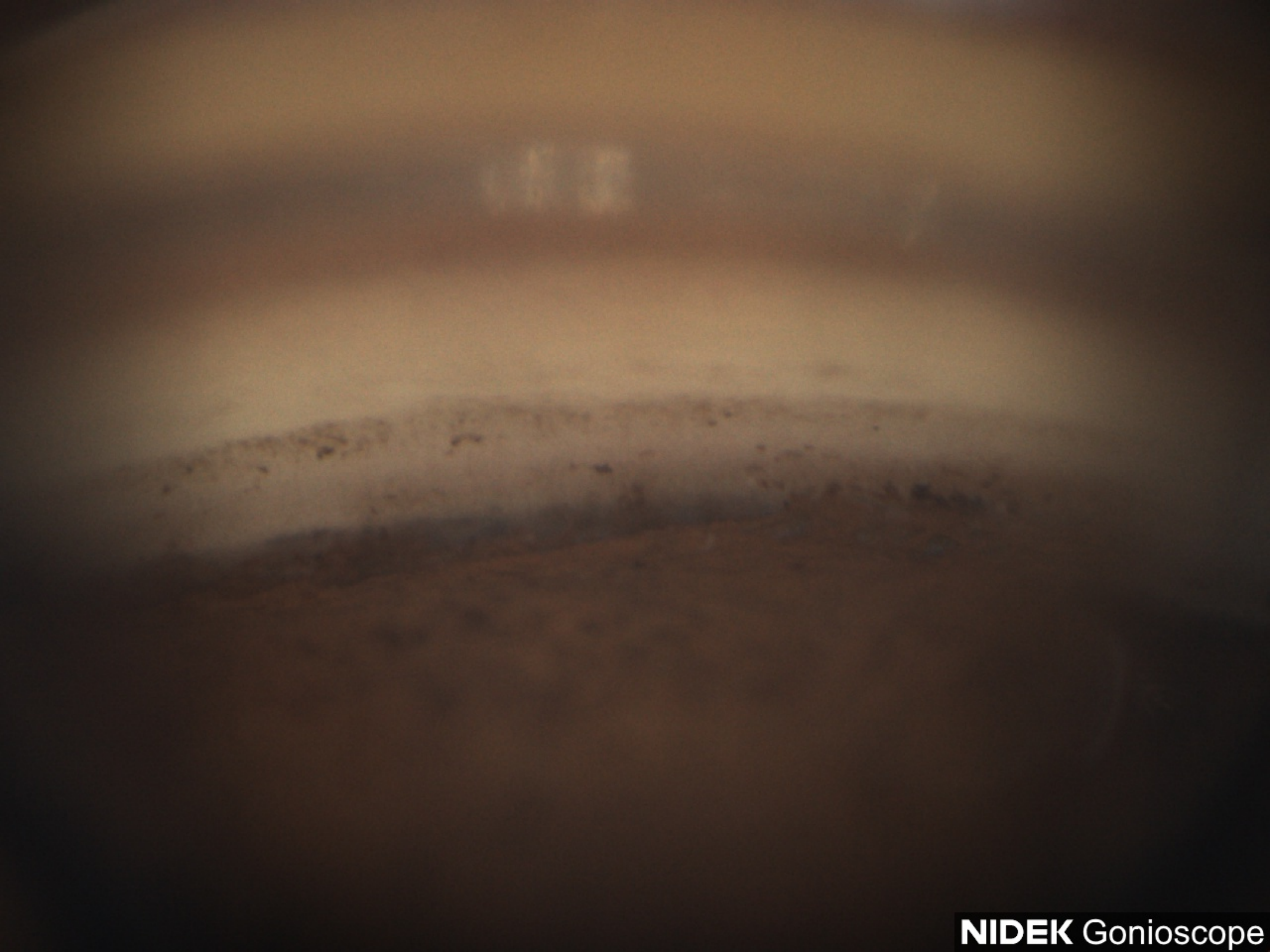

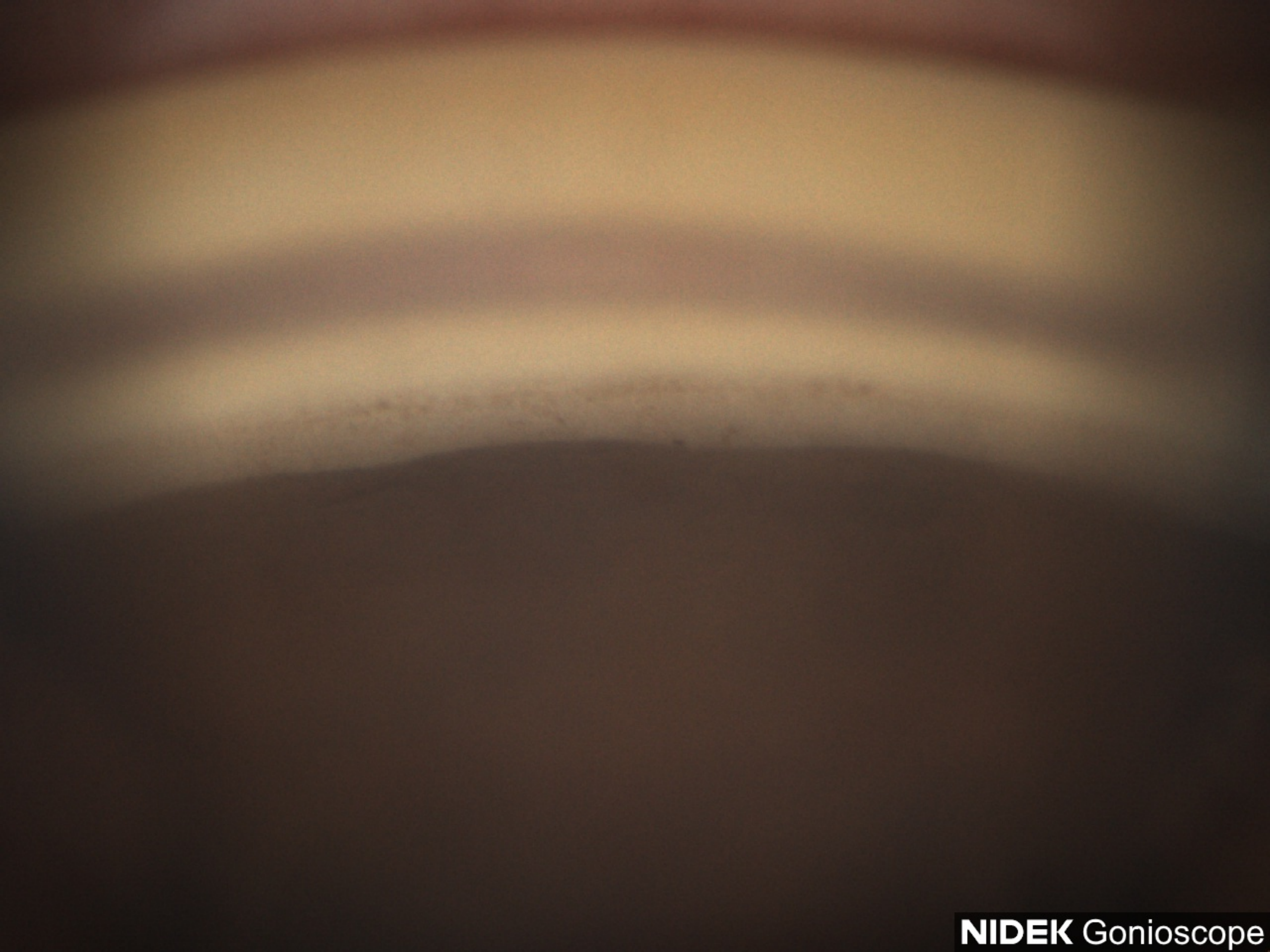

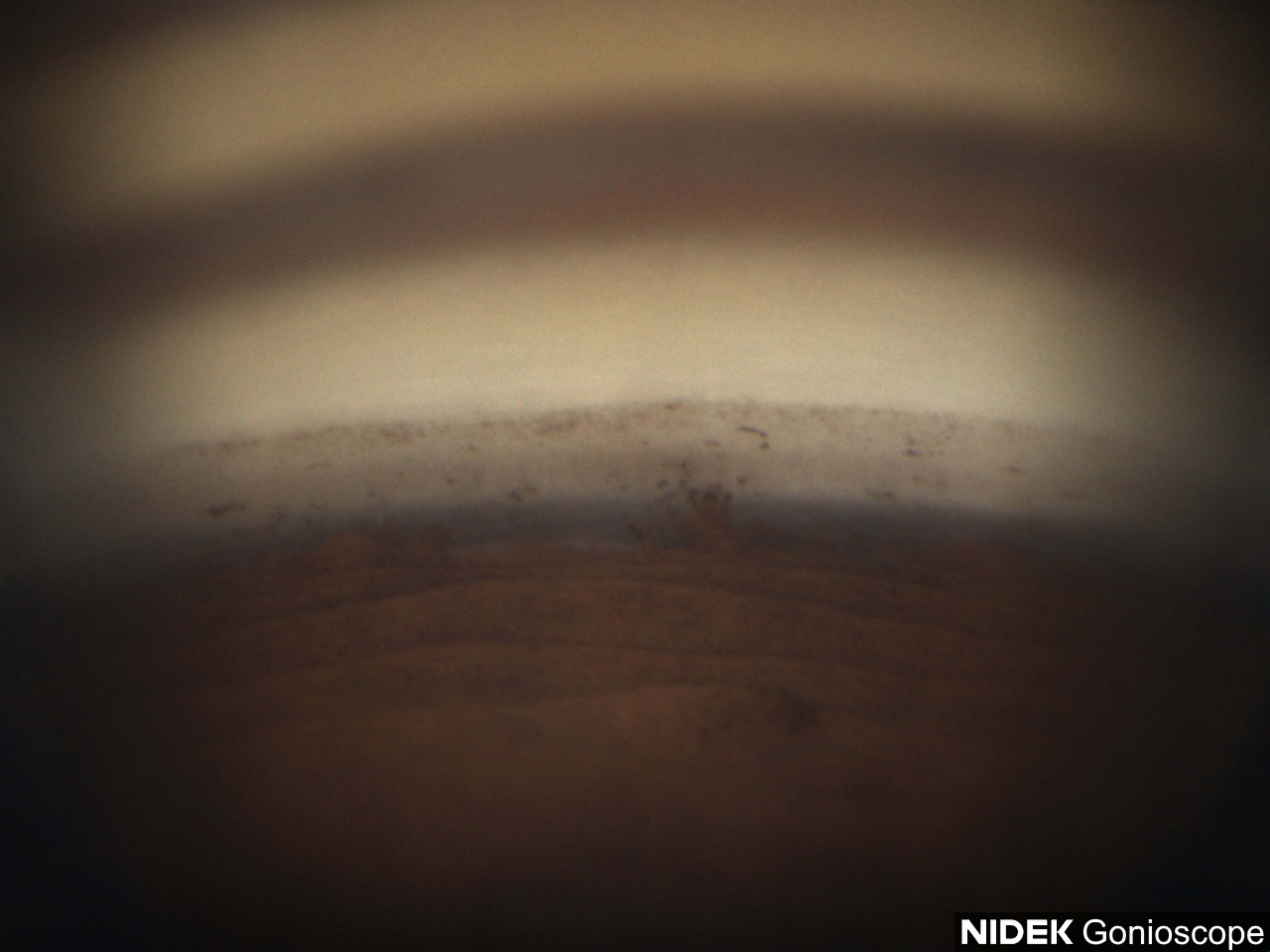

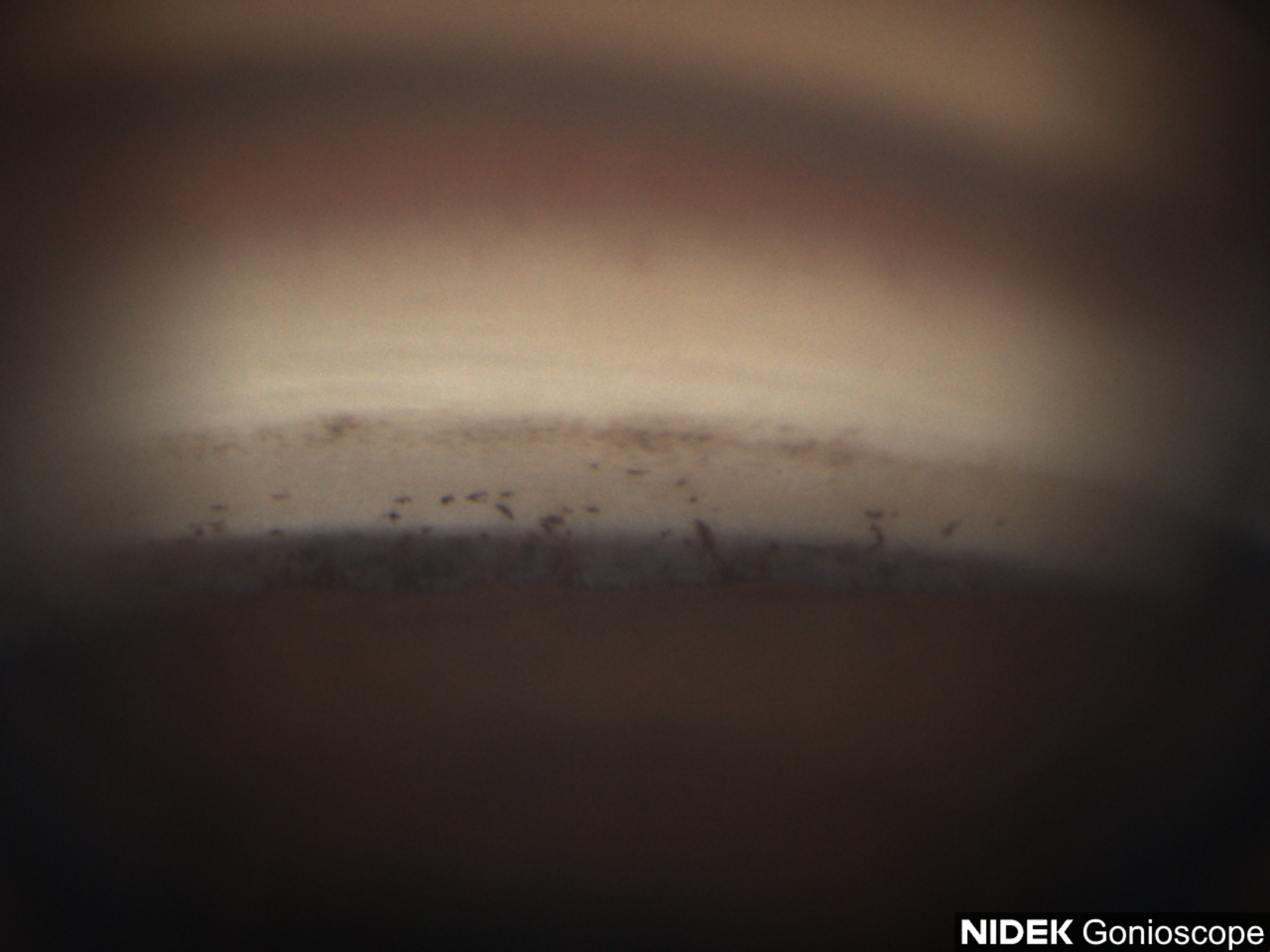

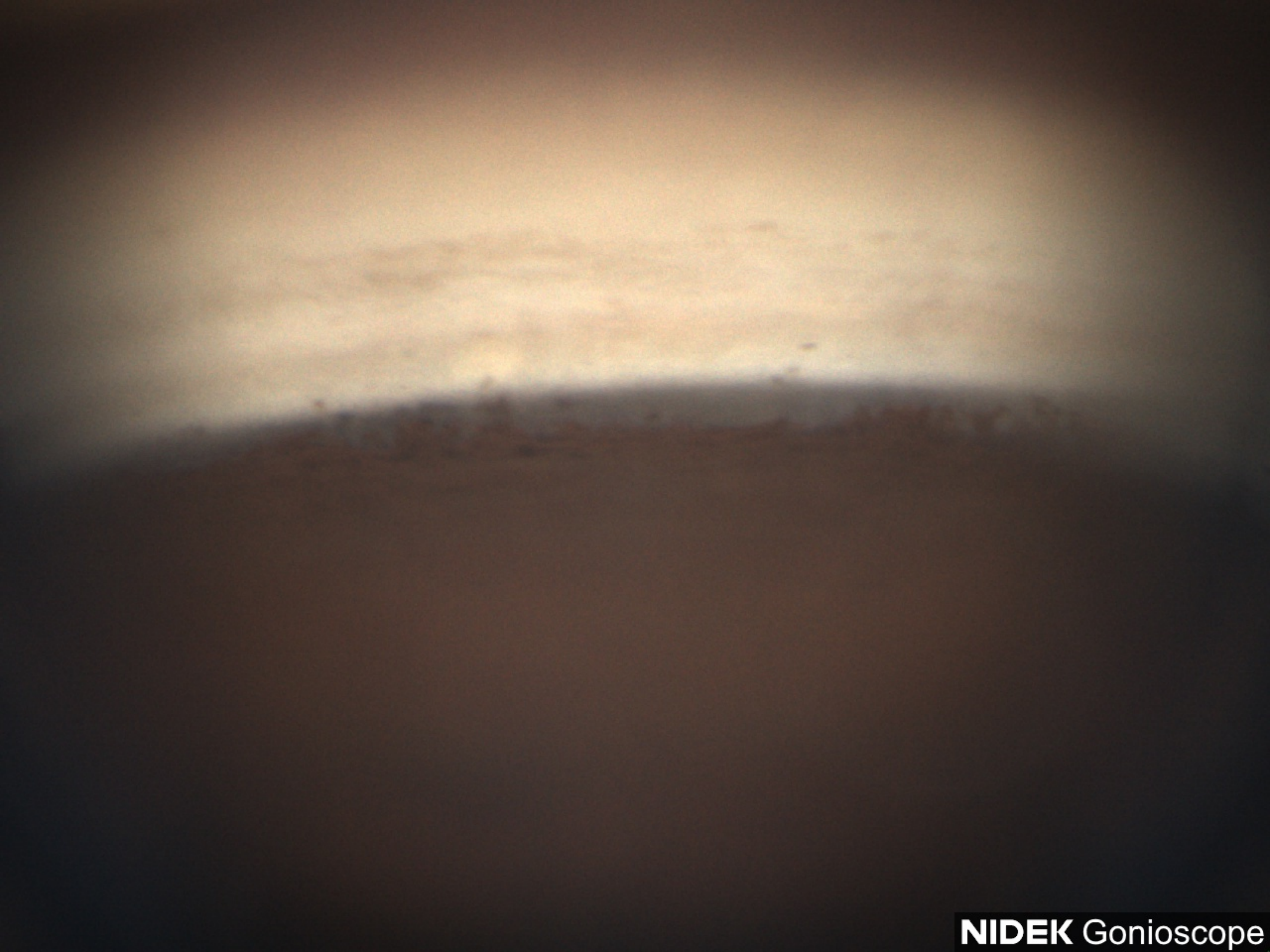

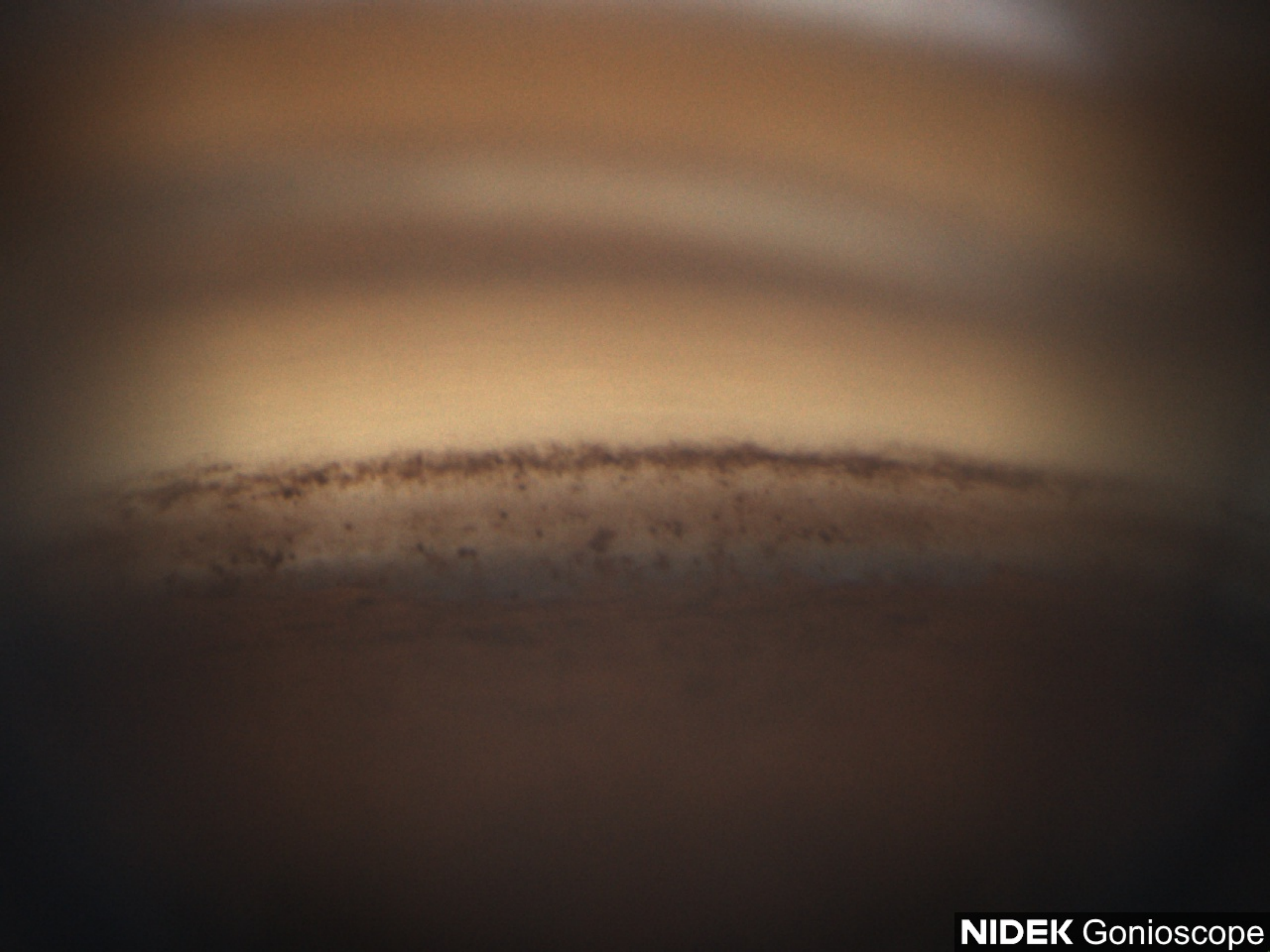

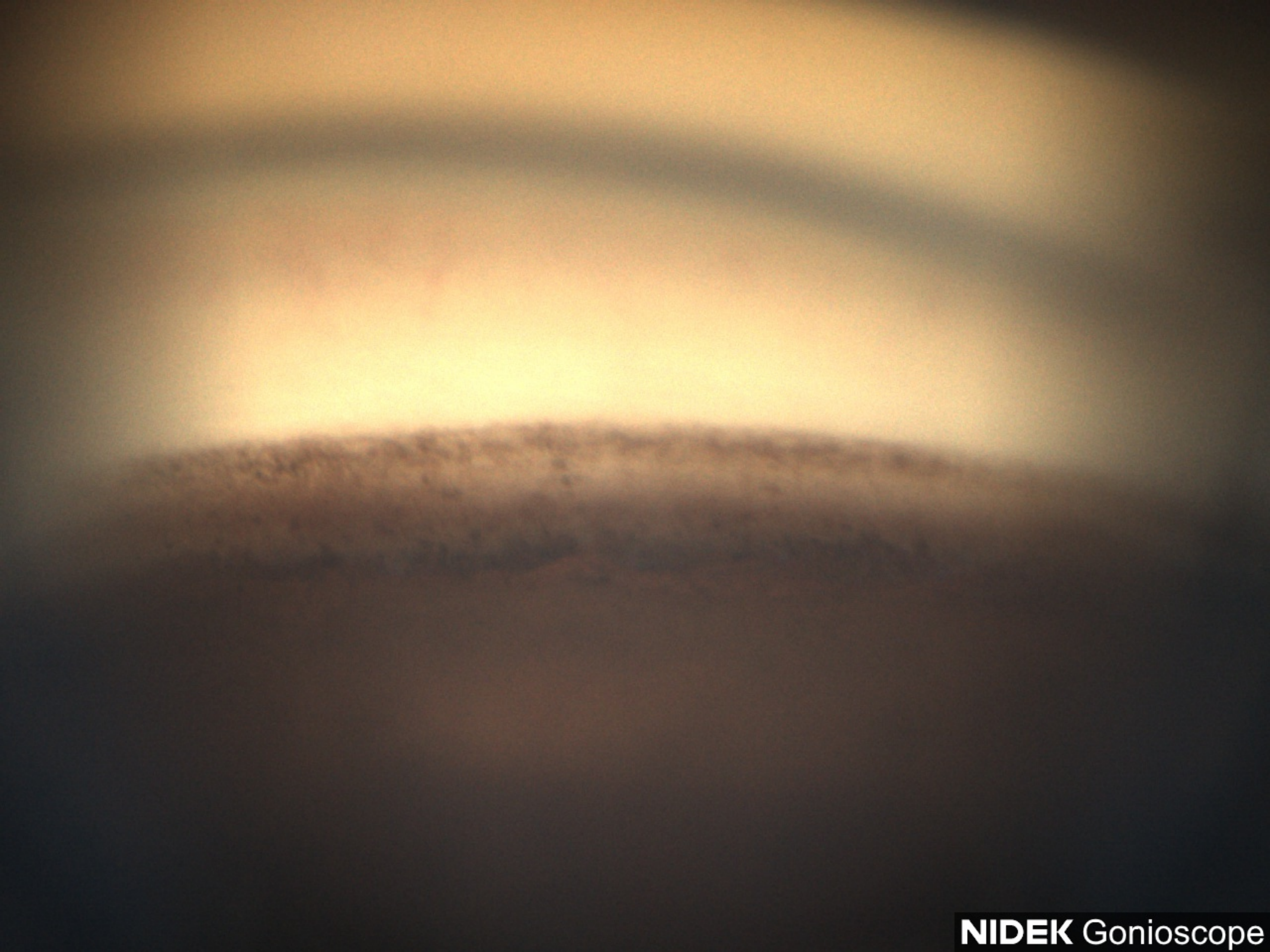

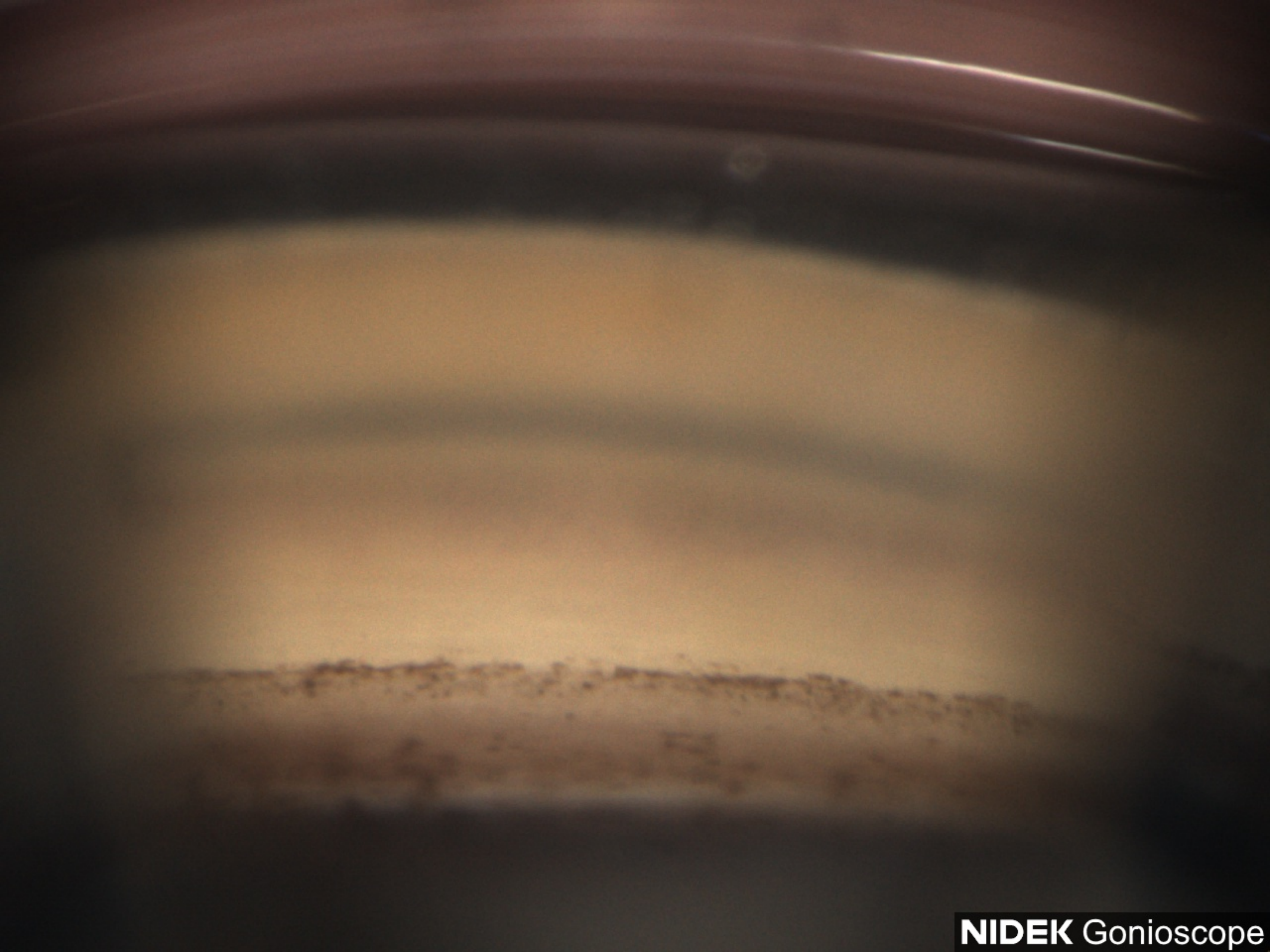

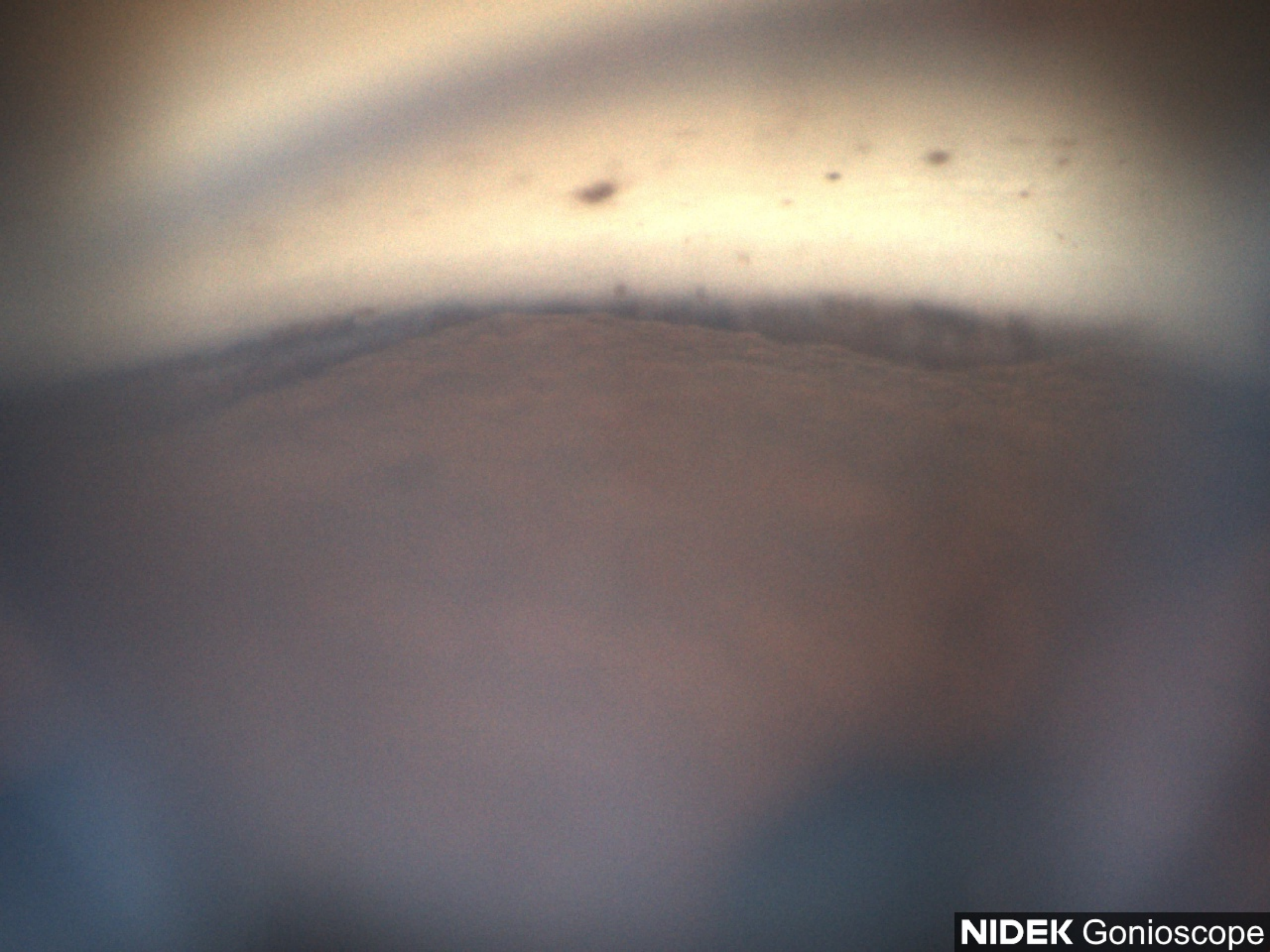

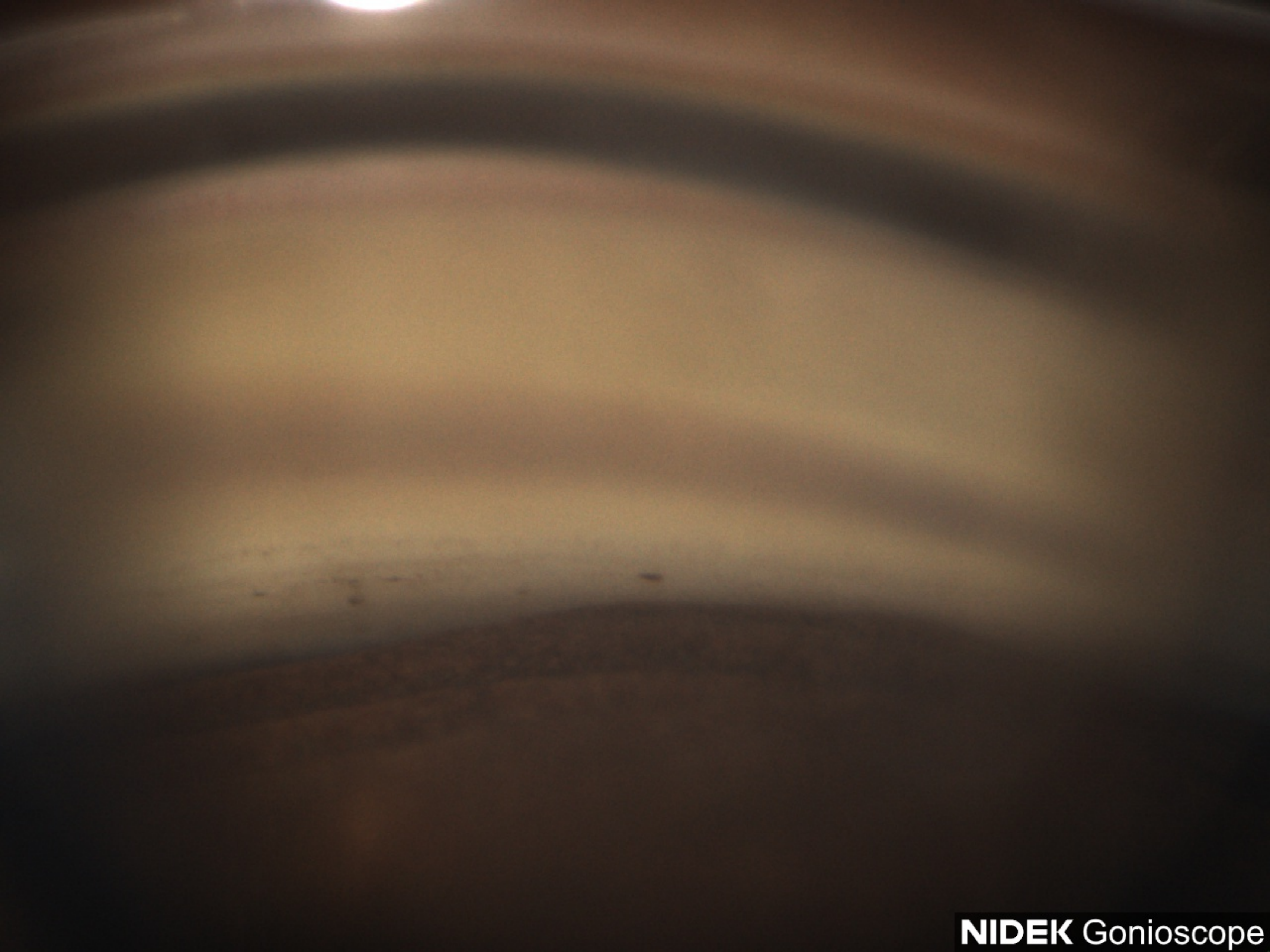

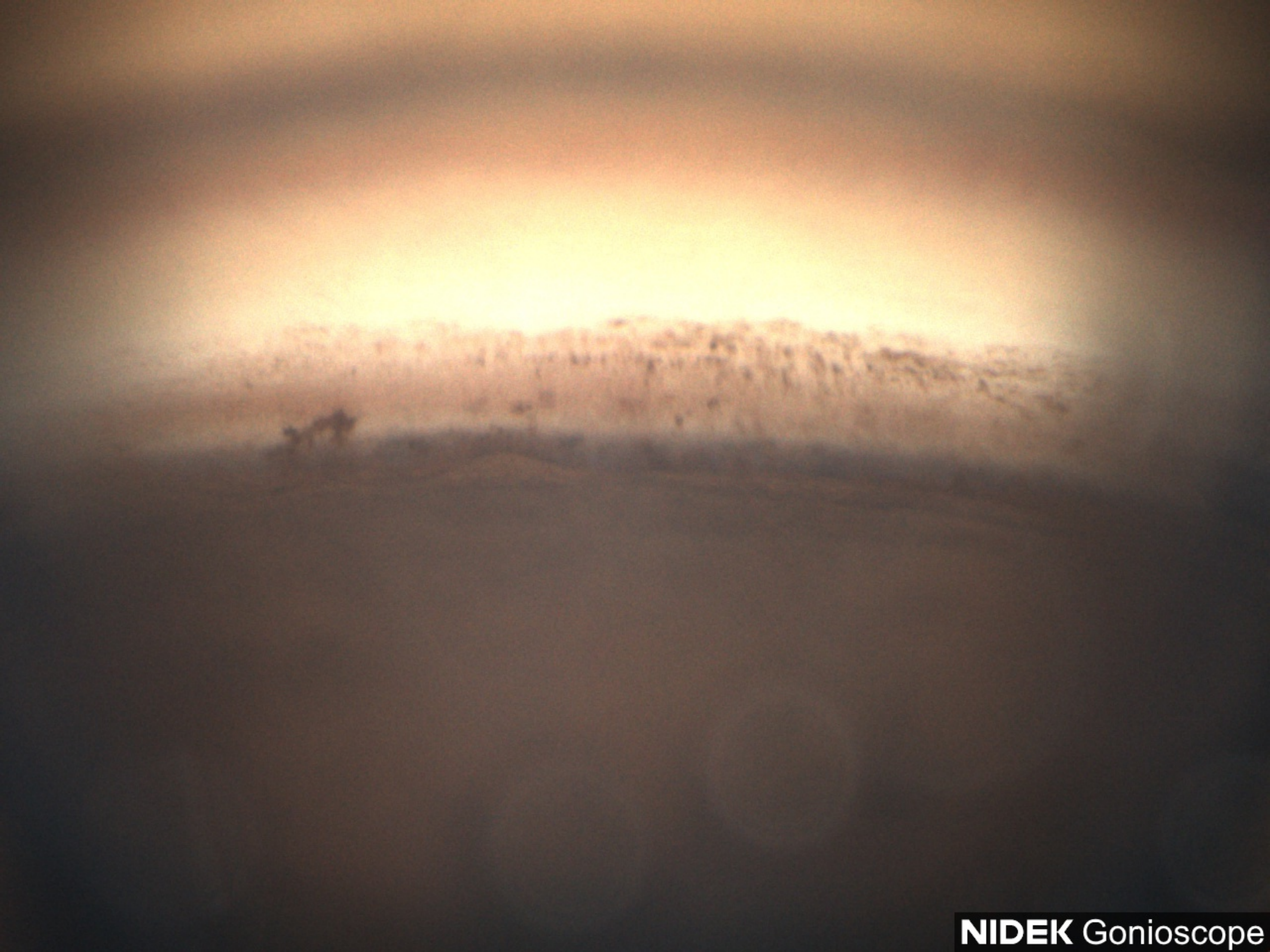

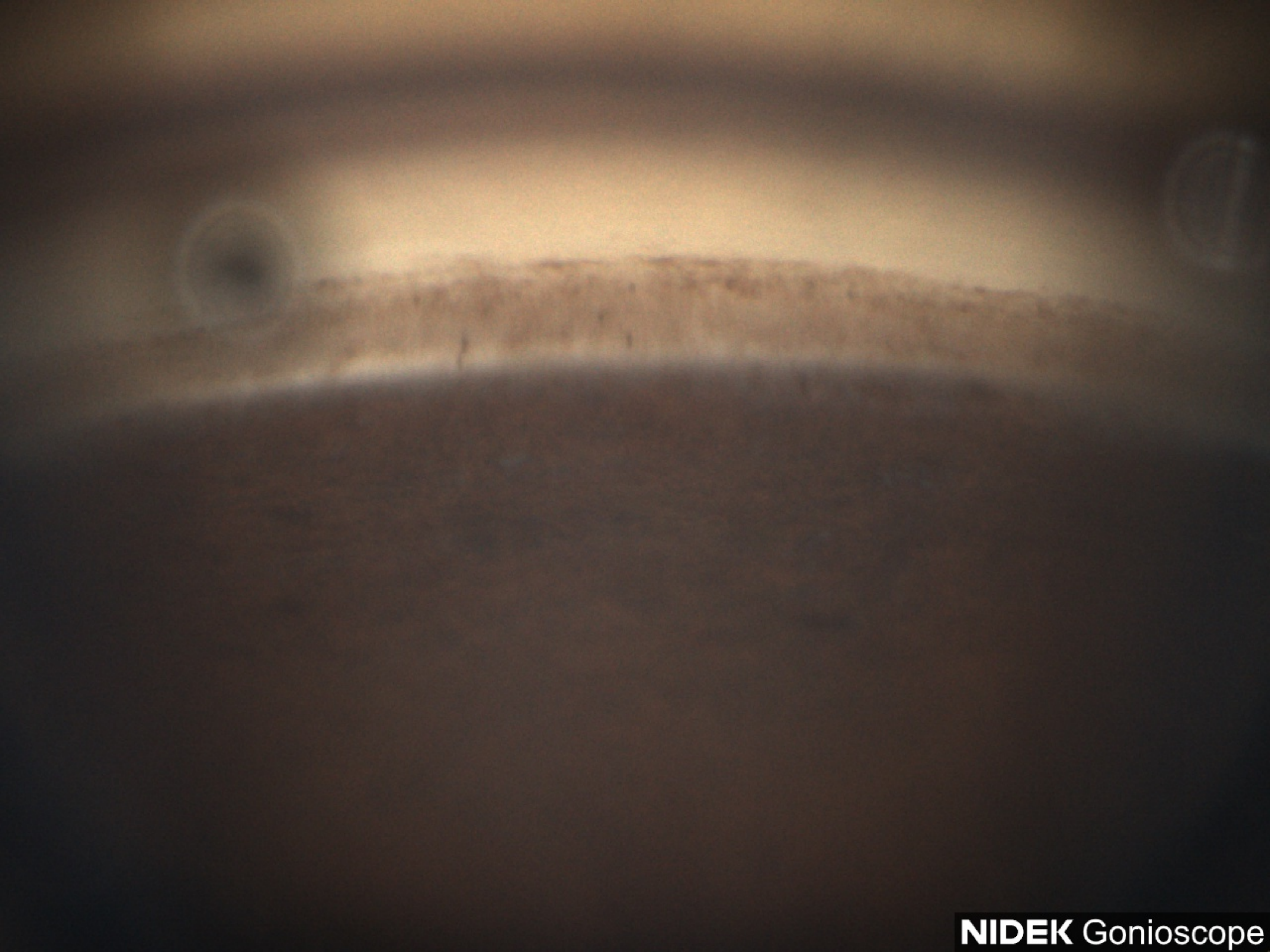

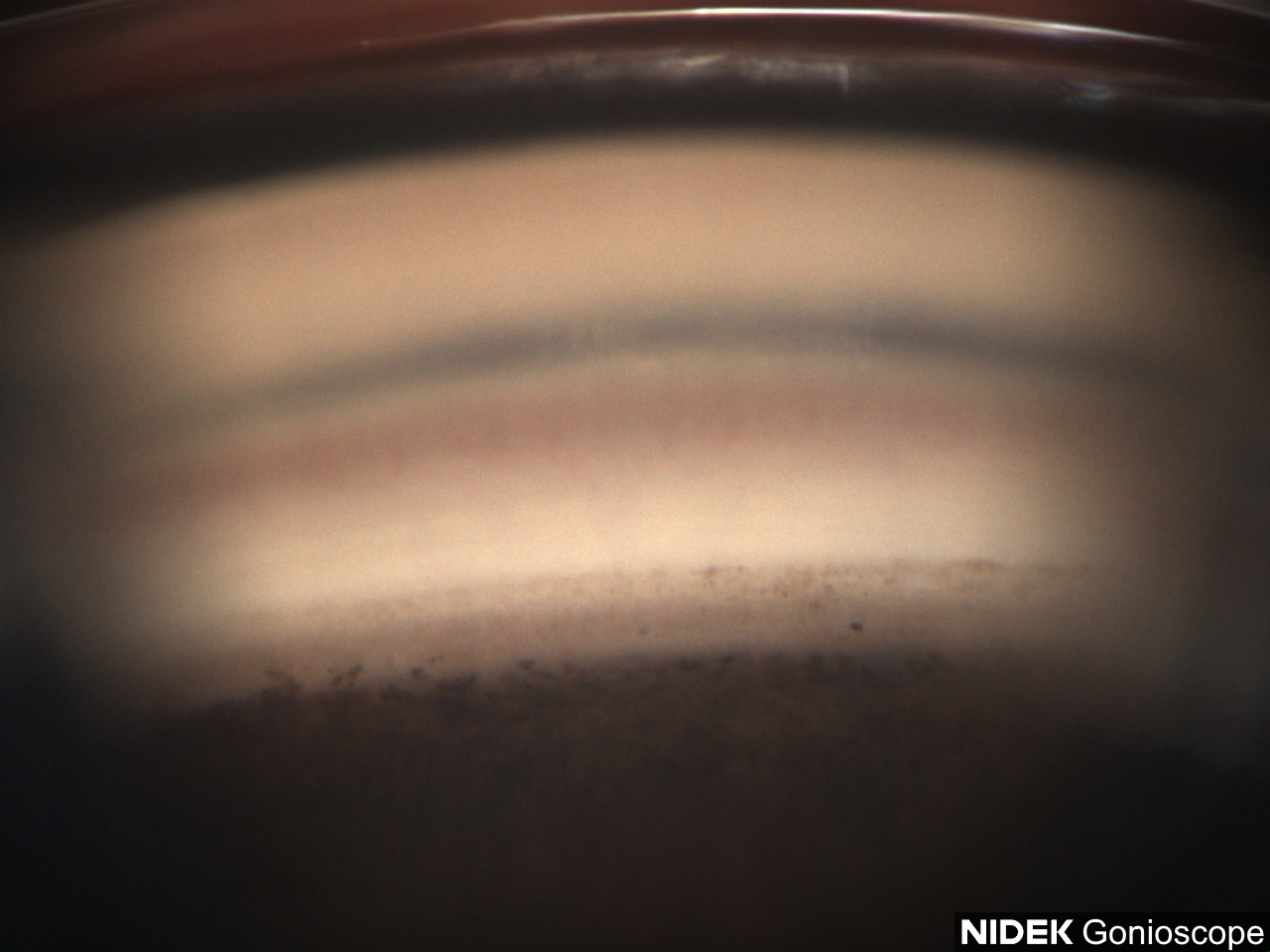

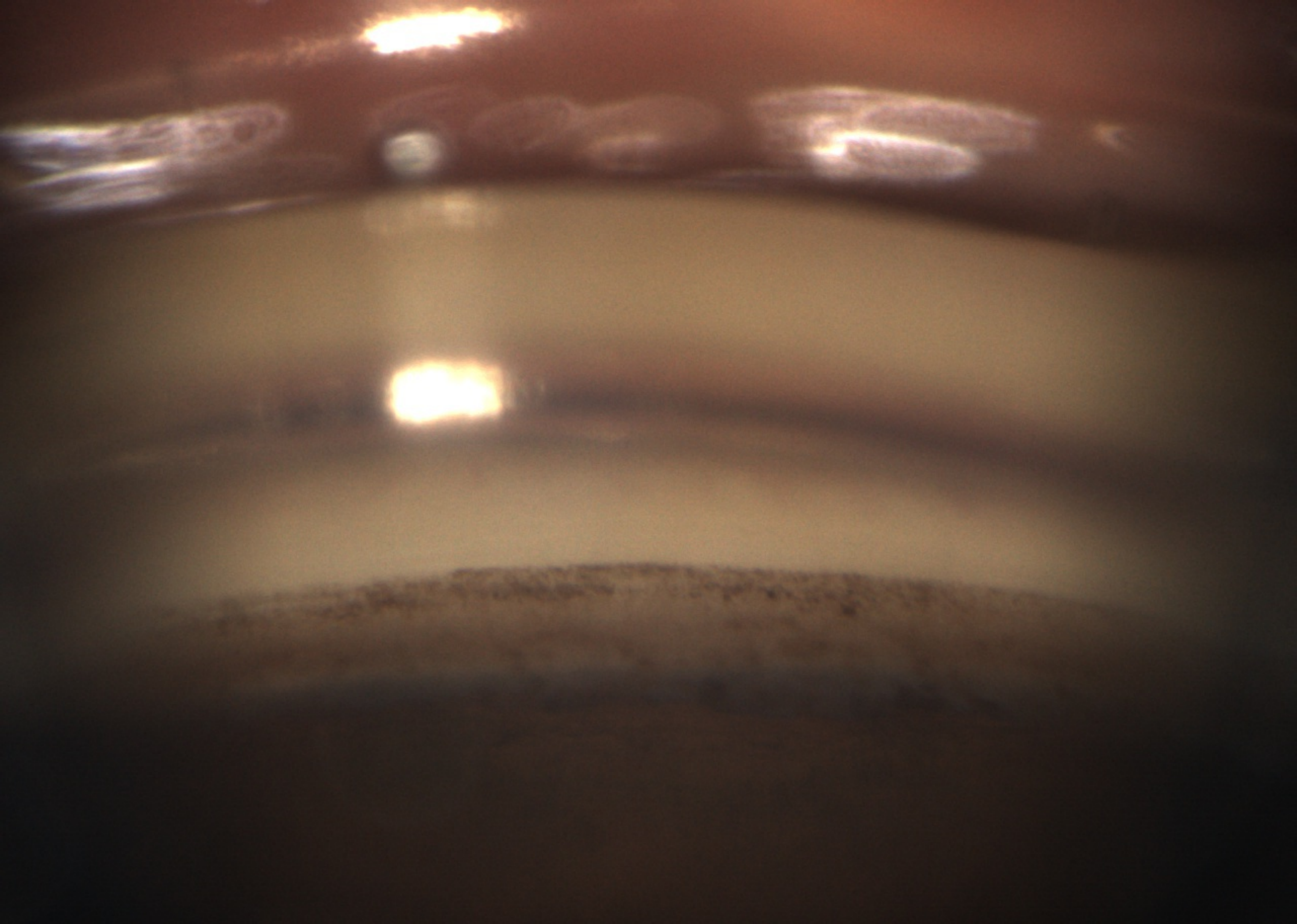

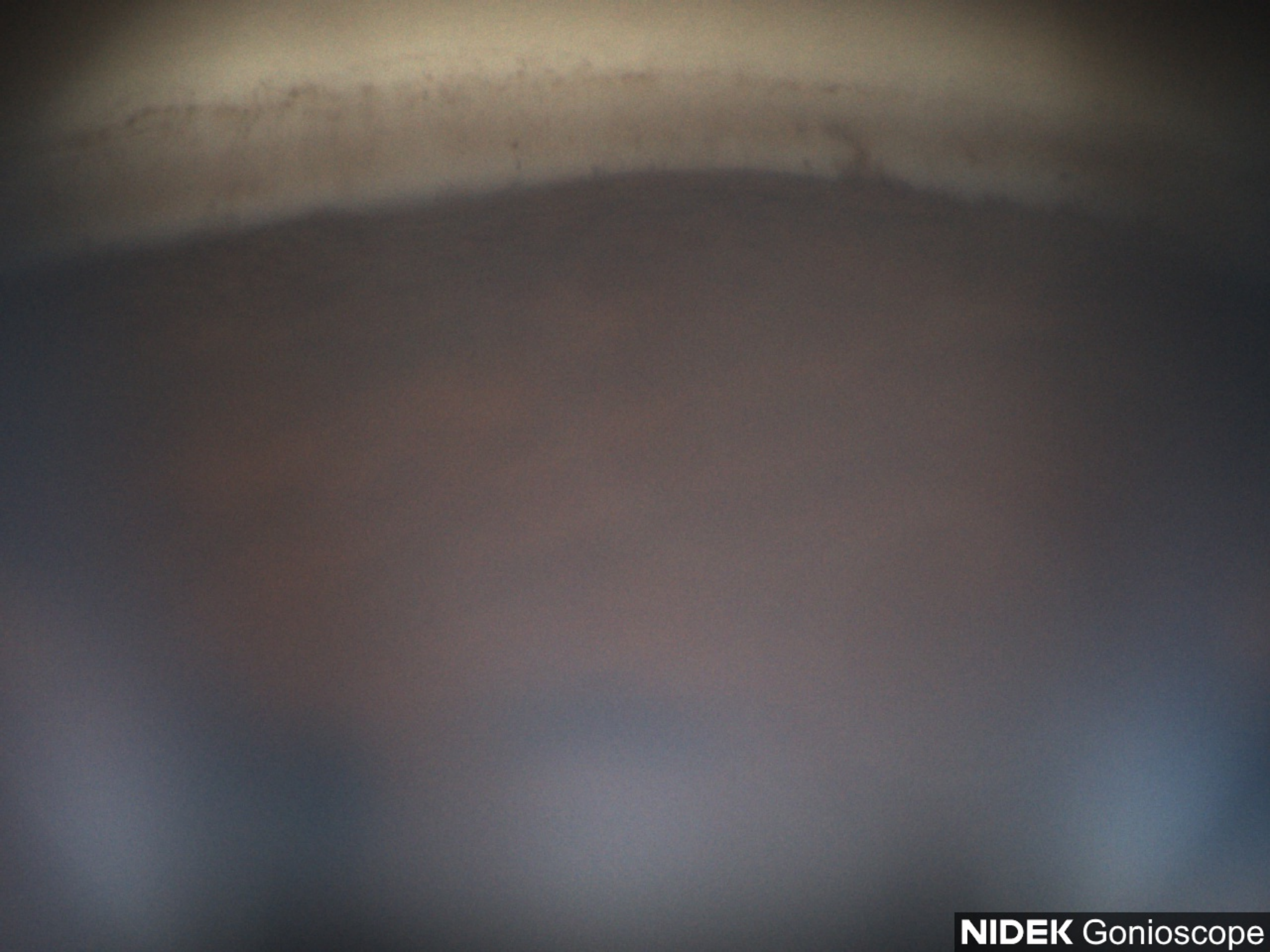

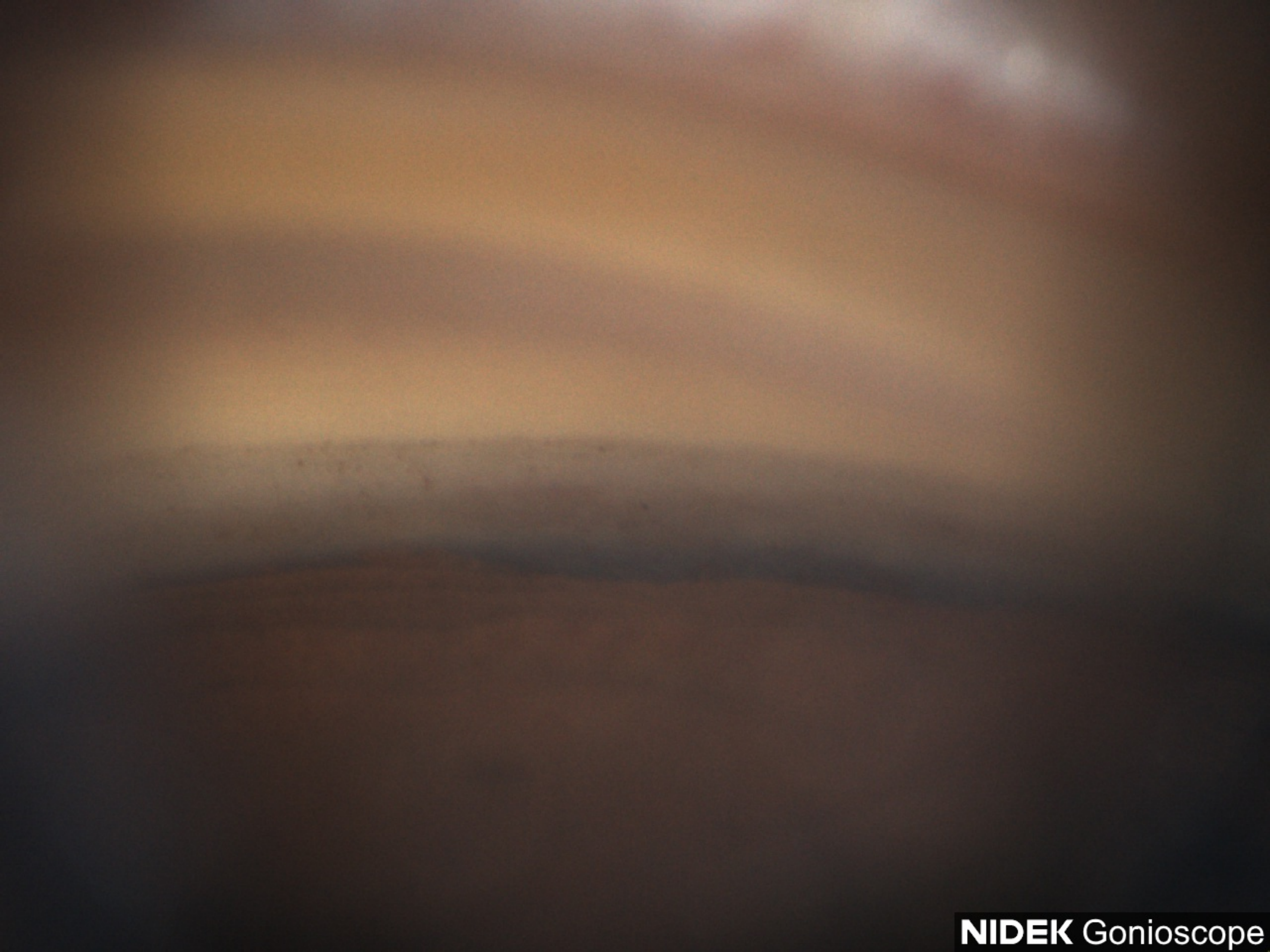

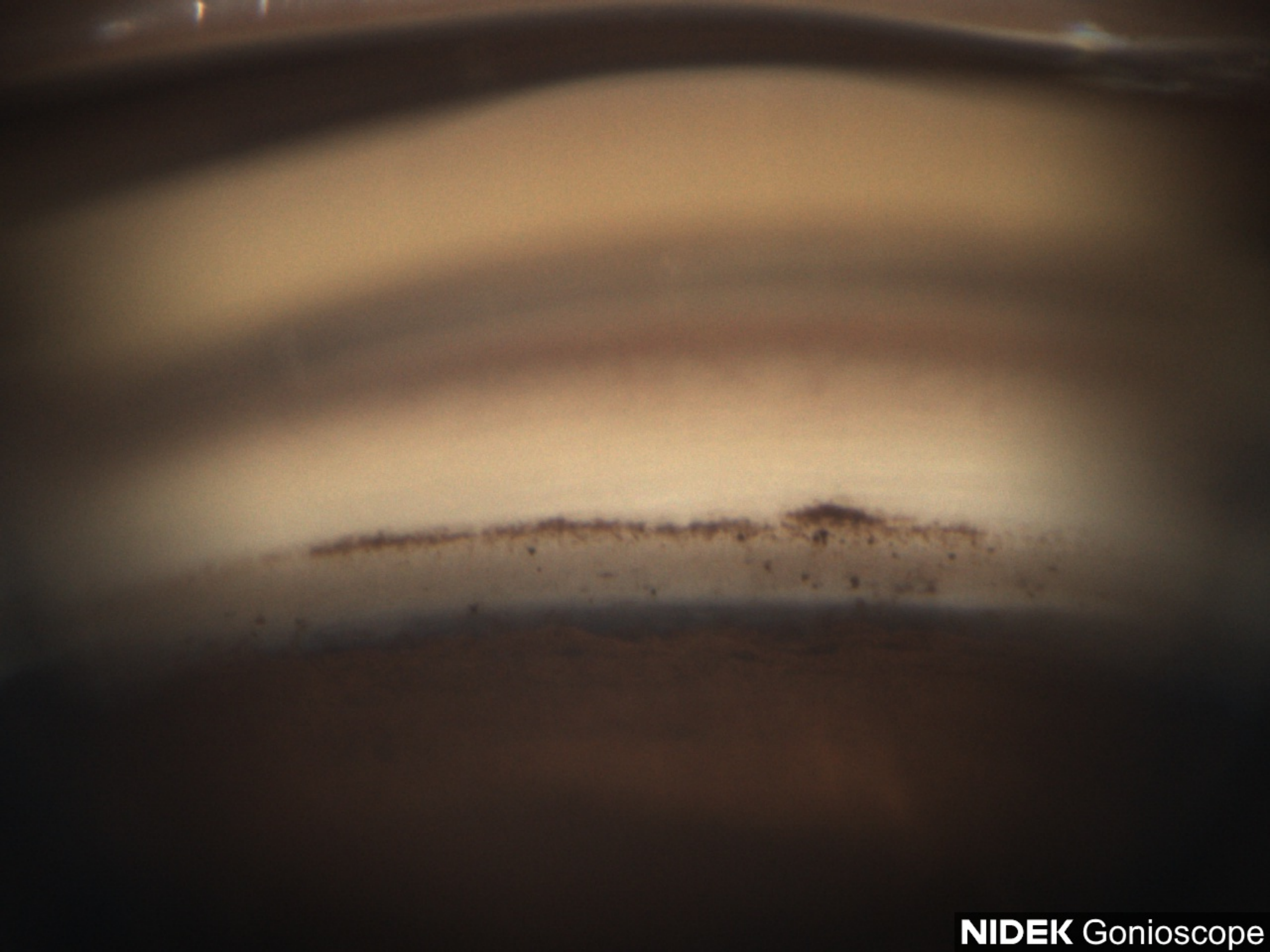

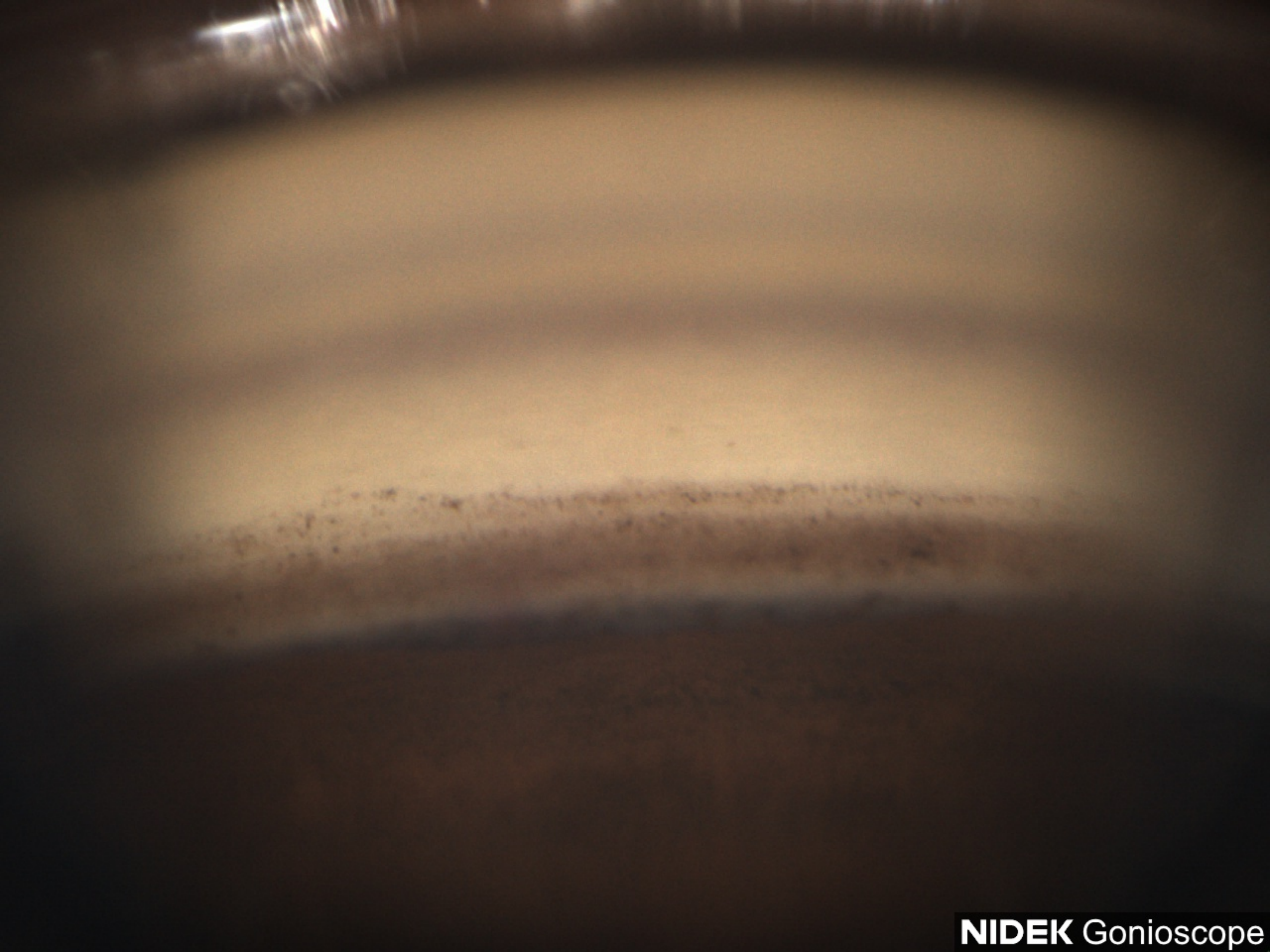

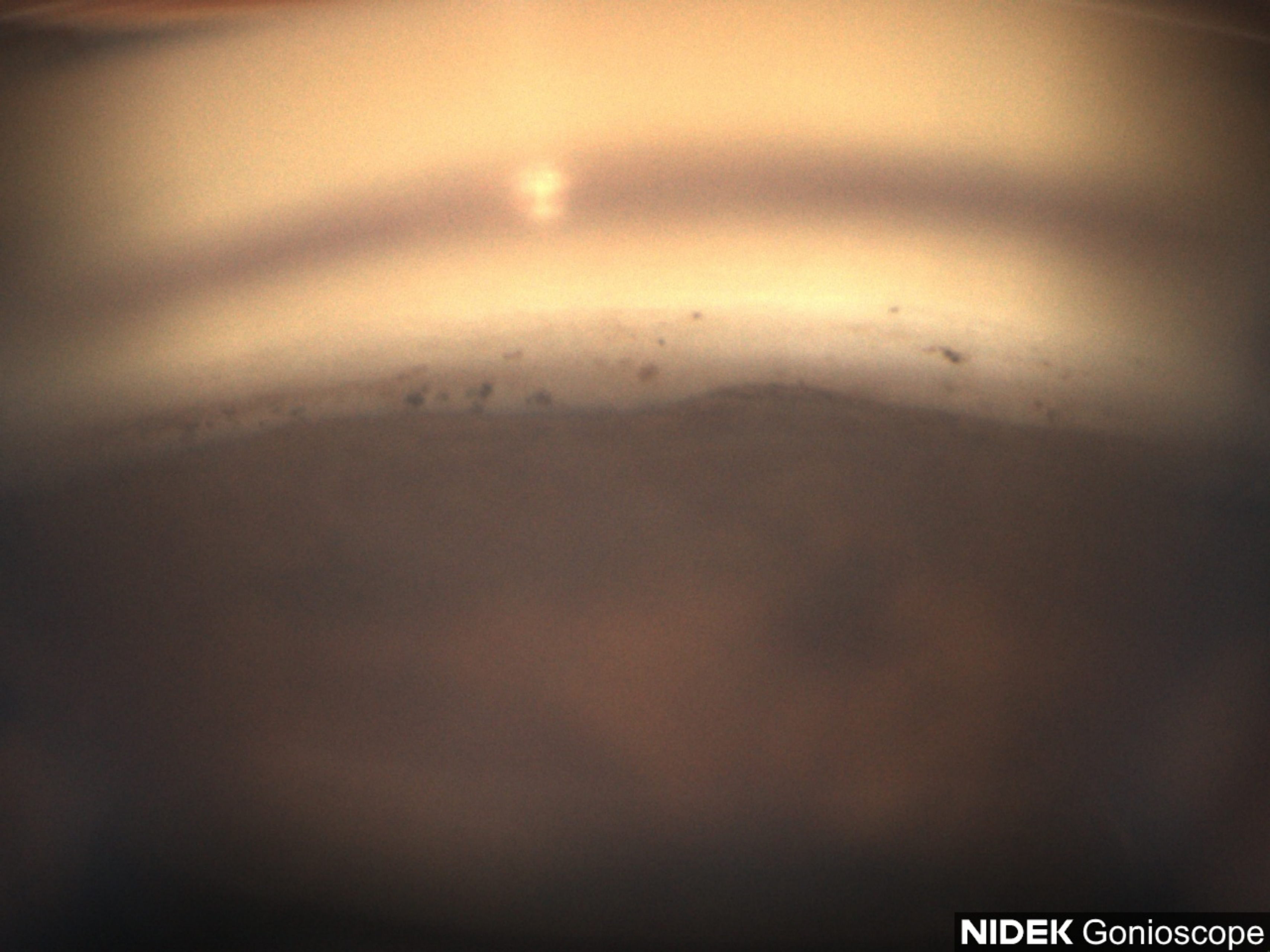

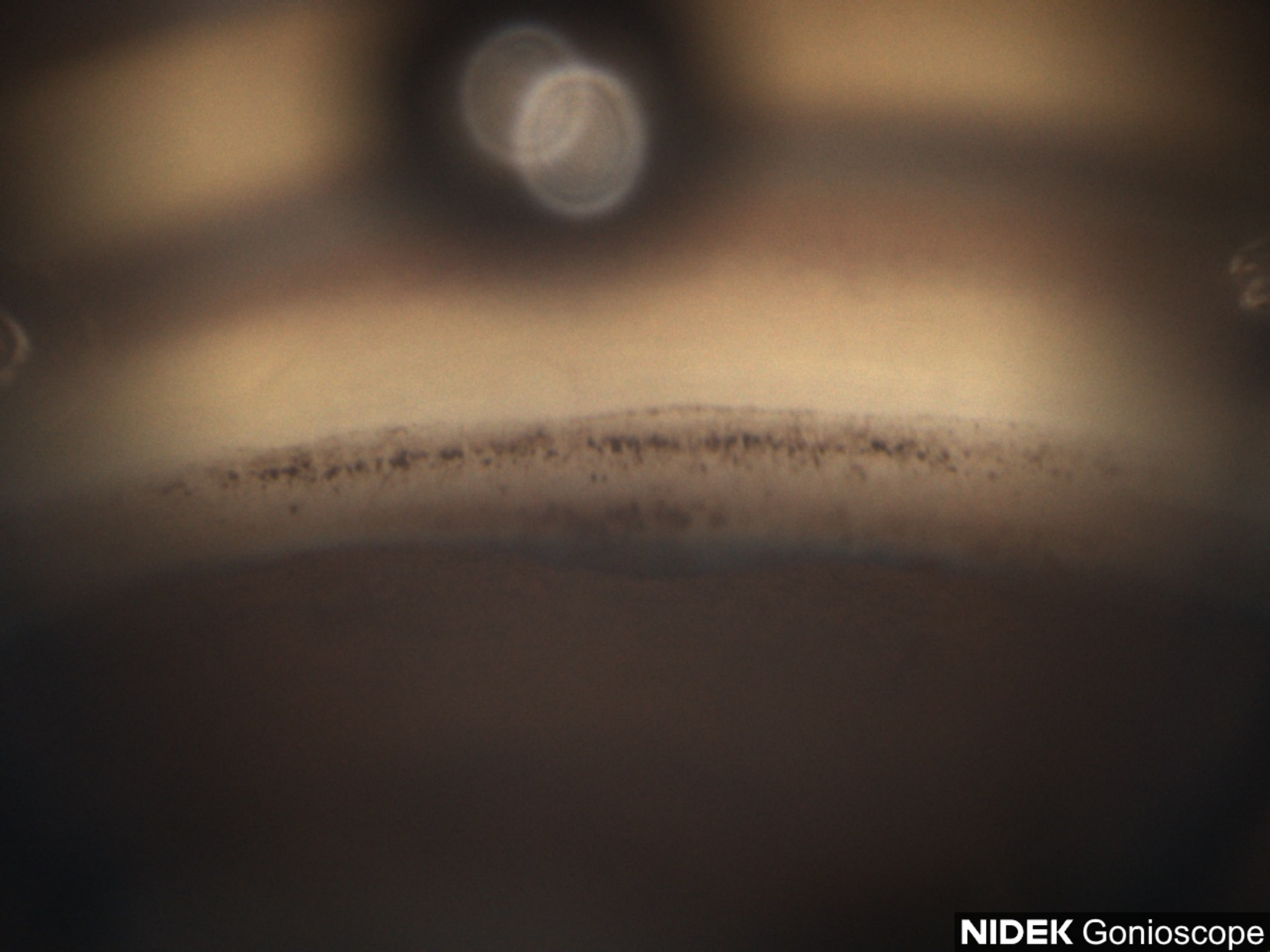

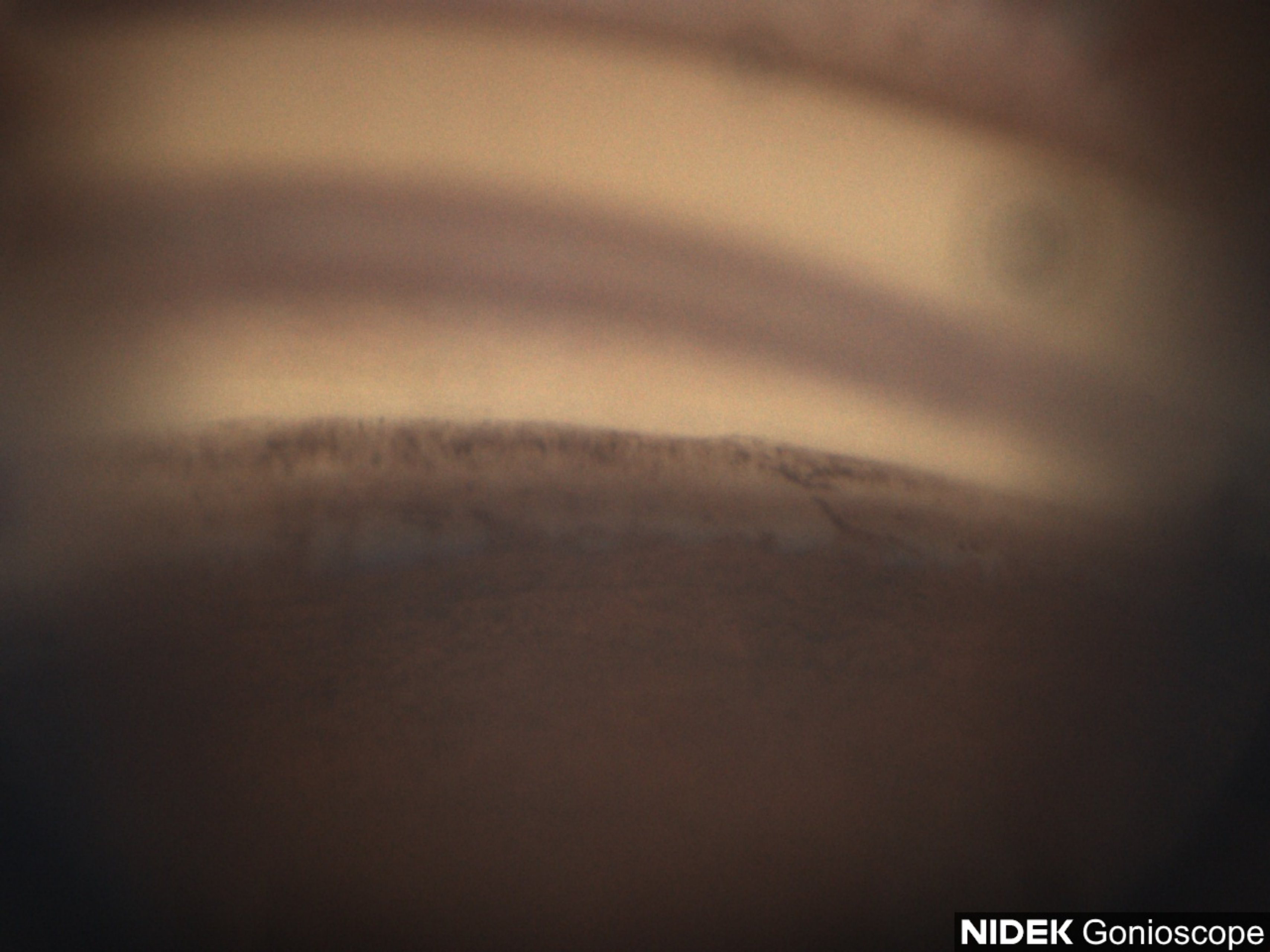

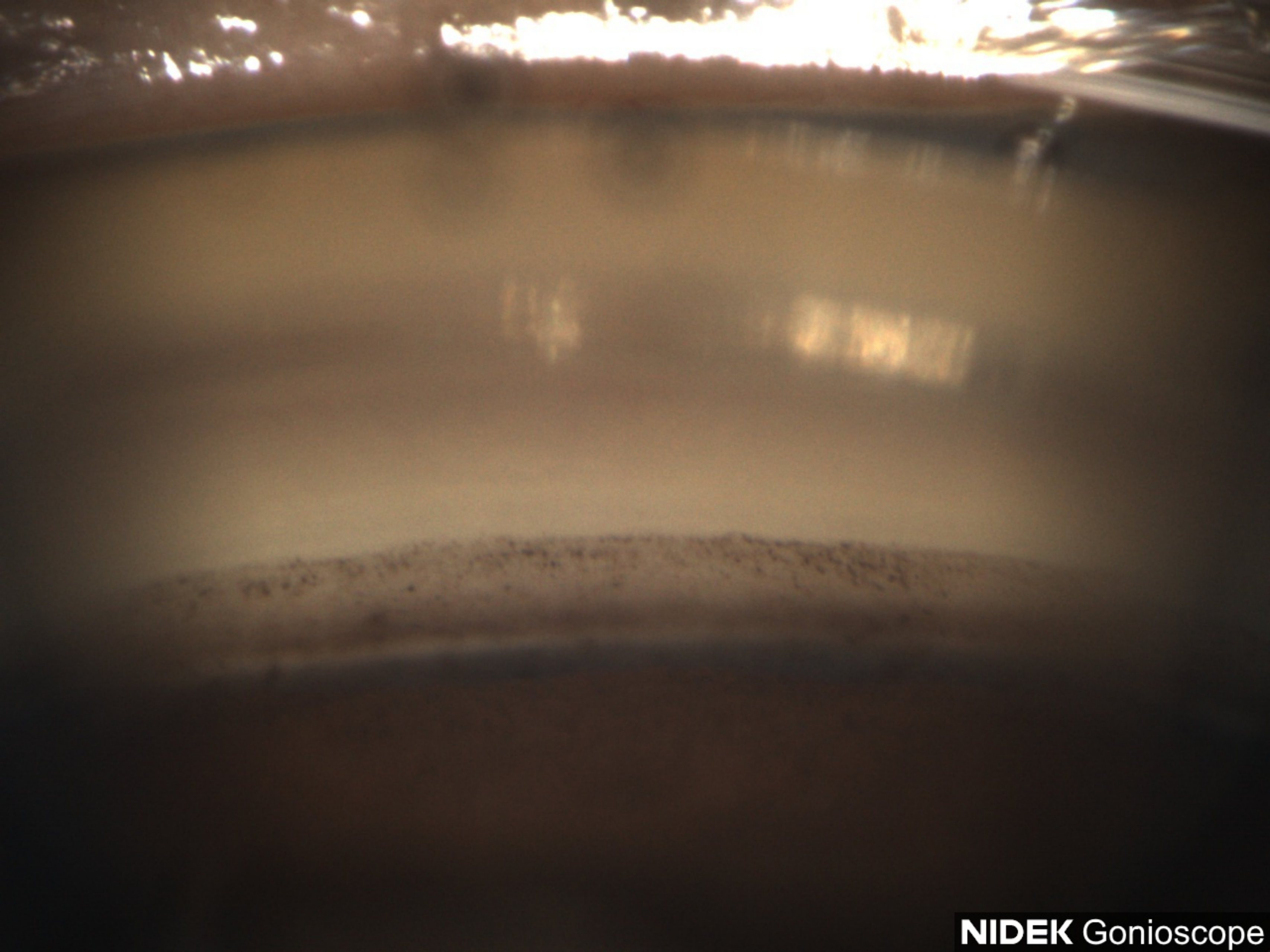

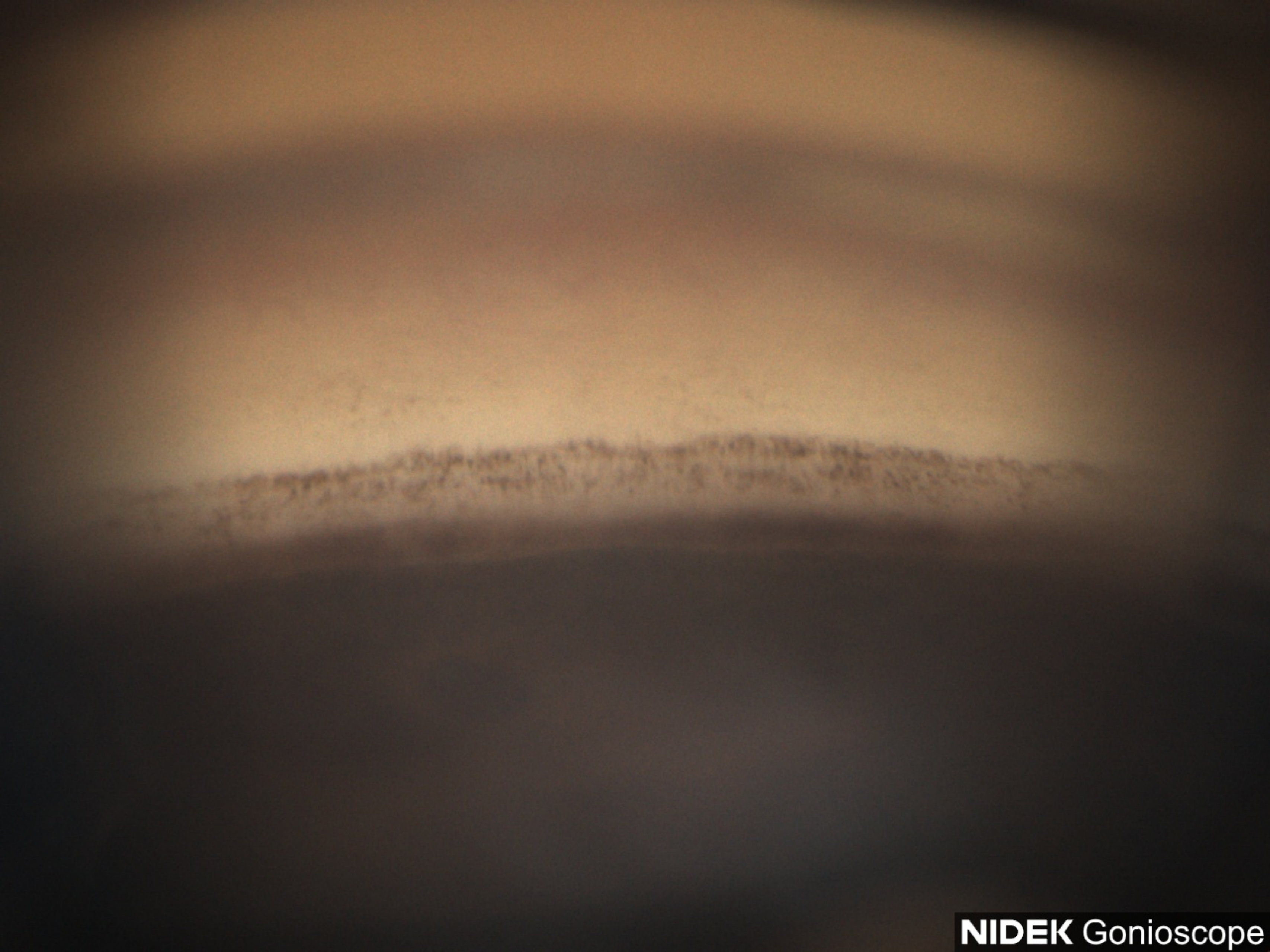

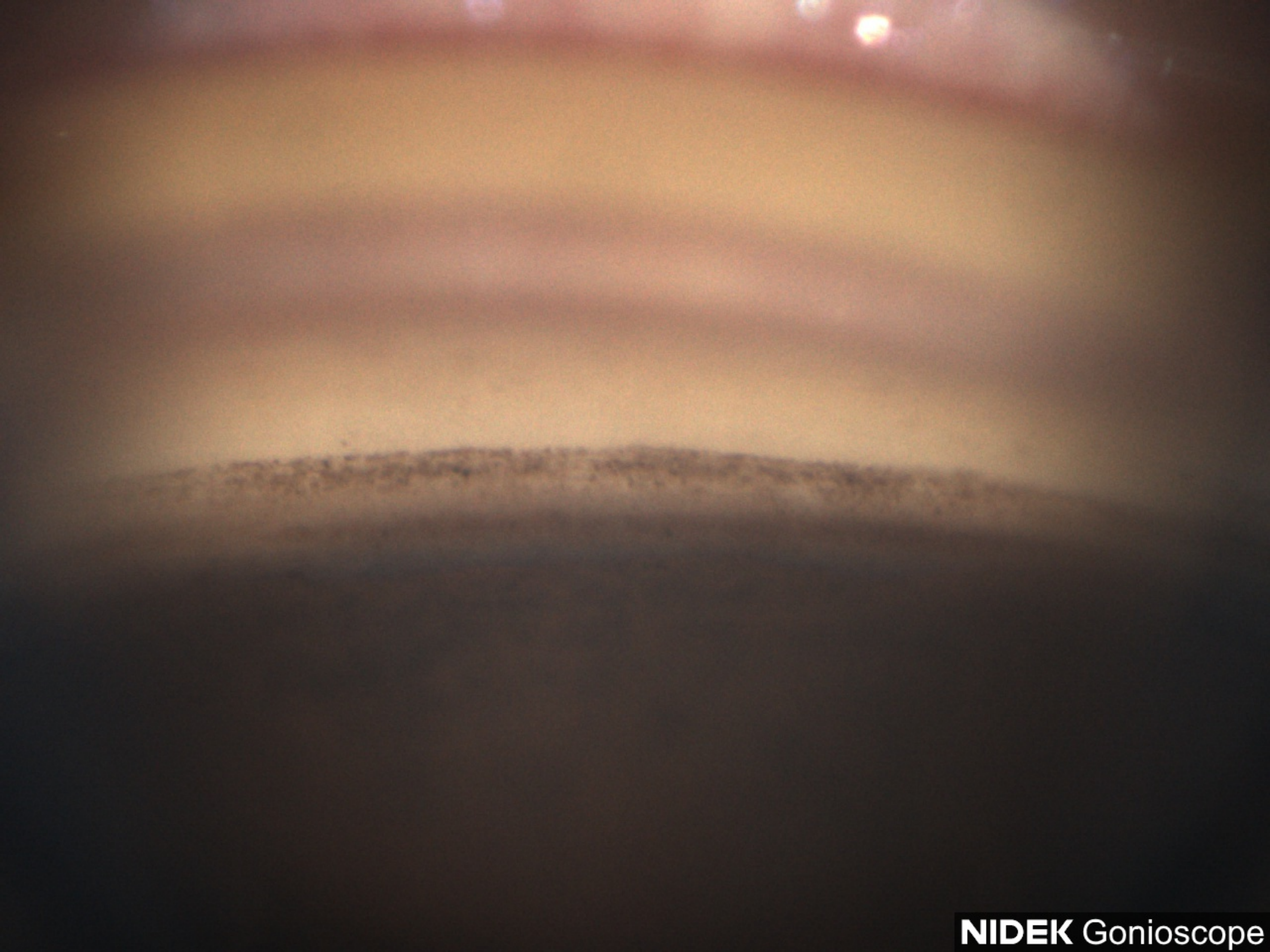

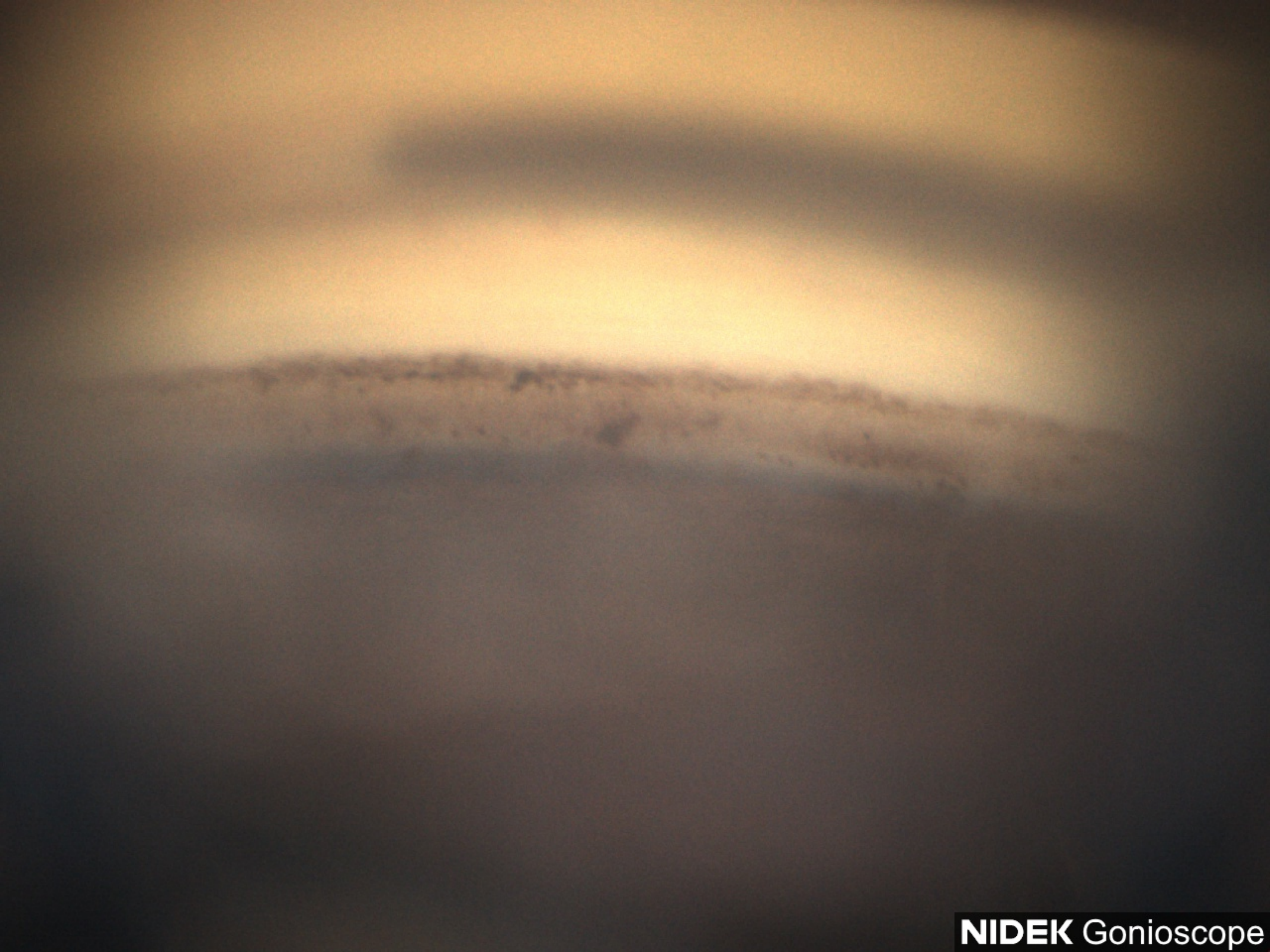

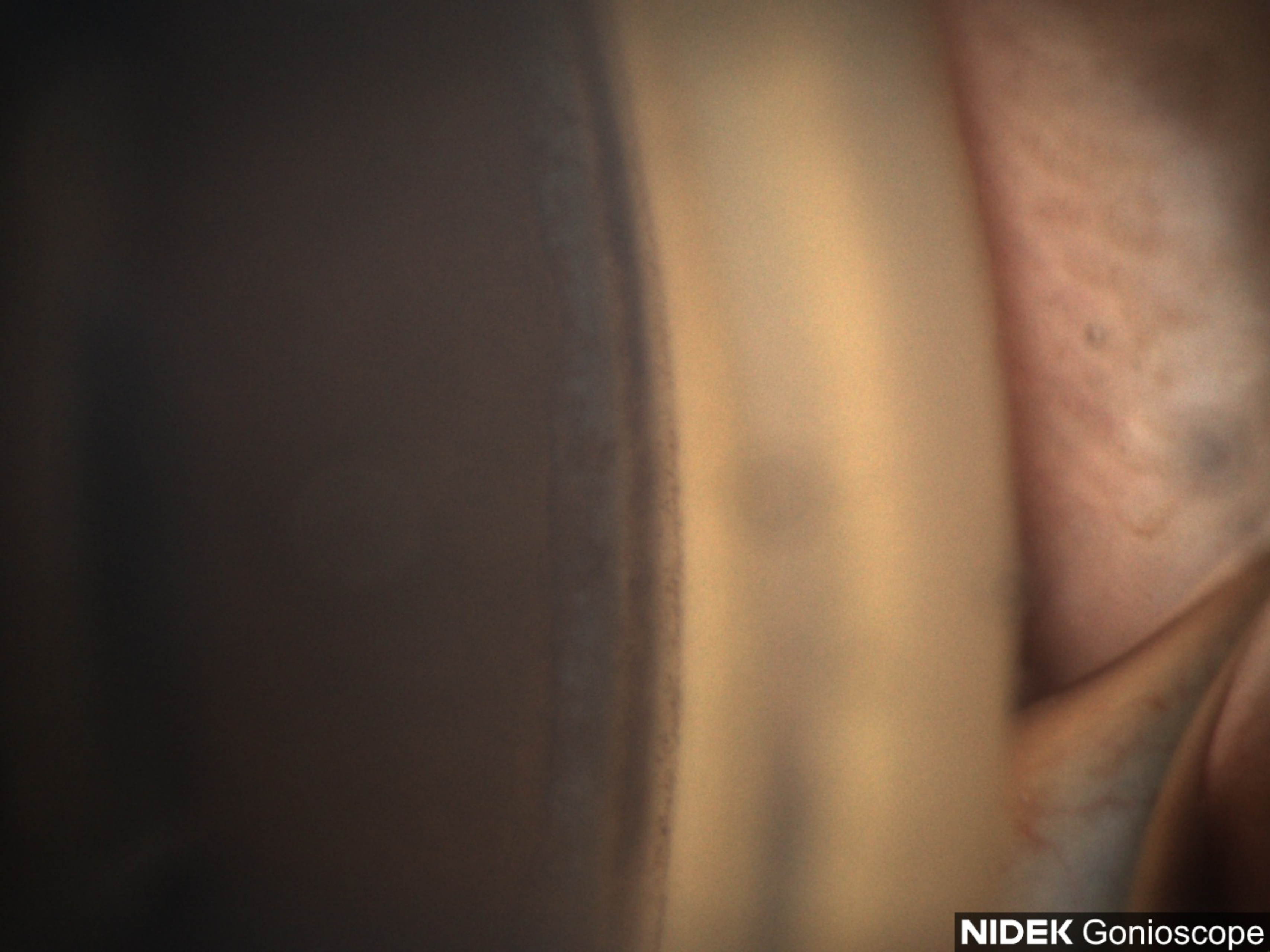

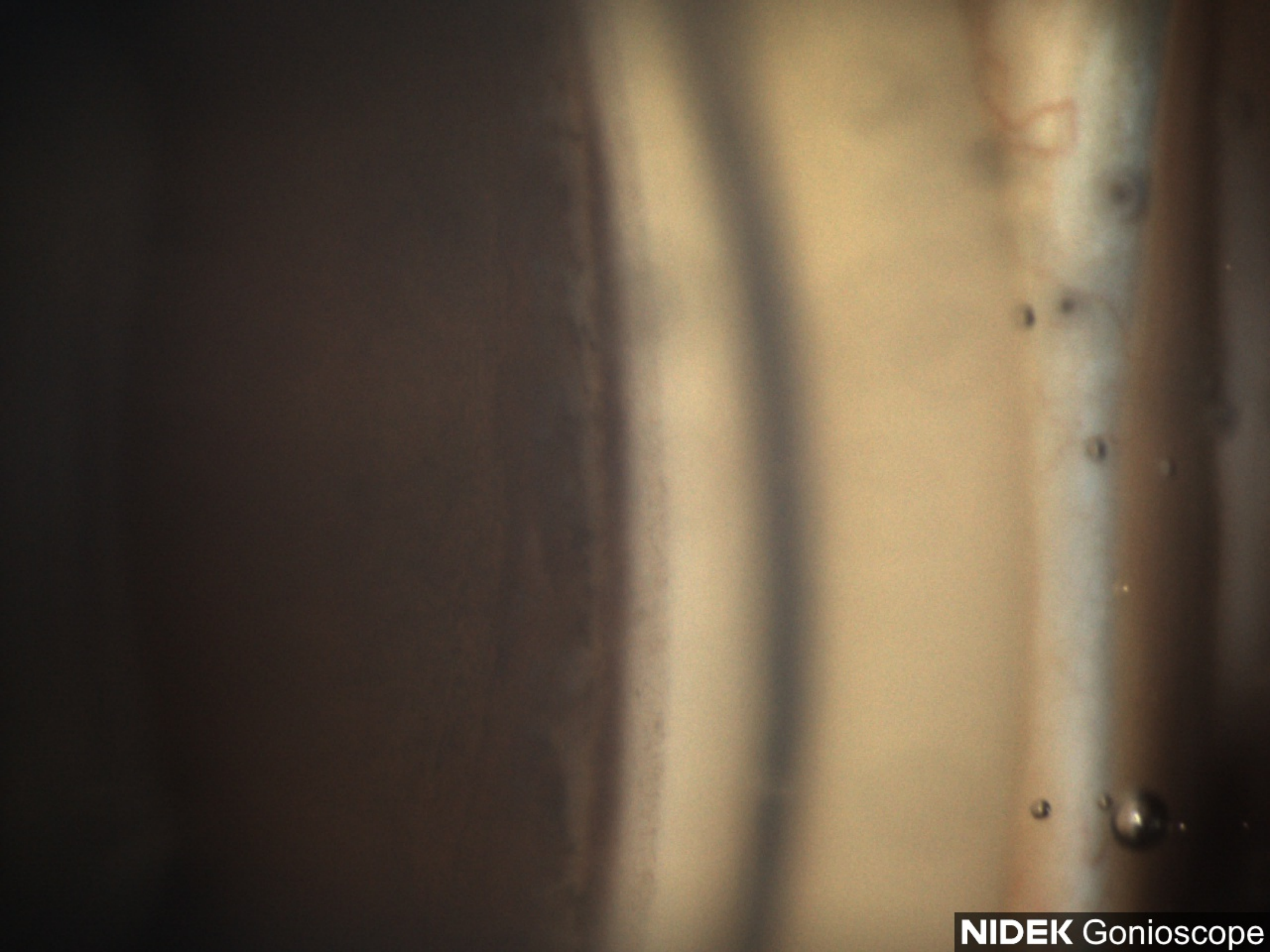

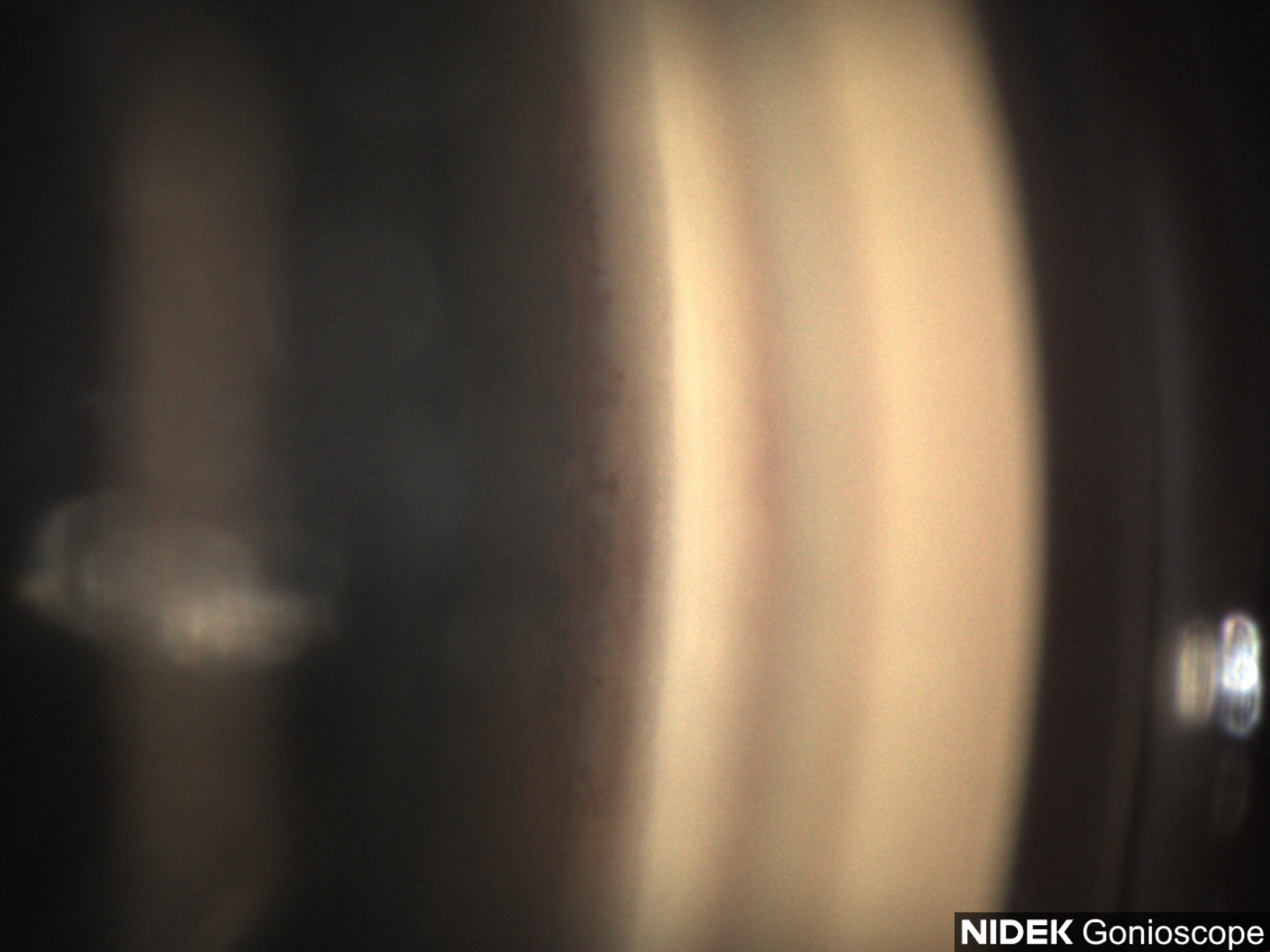

**NIDEK** Gonioscope



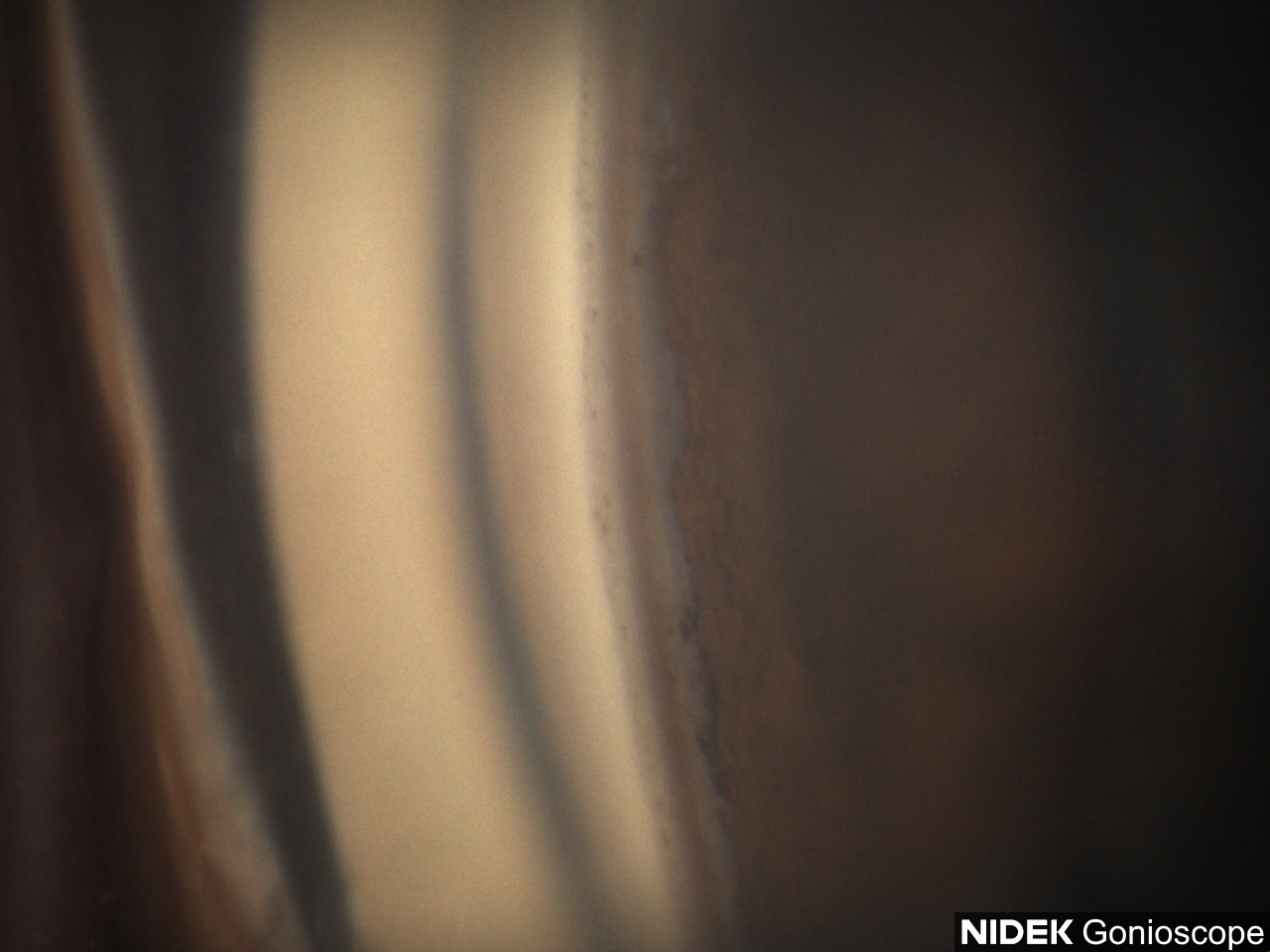



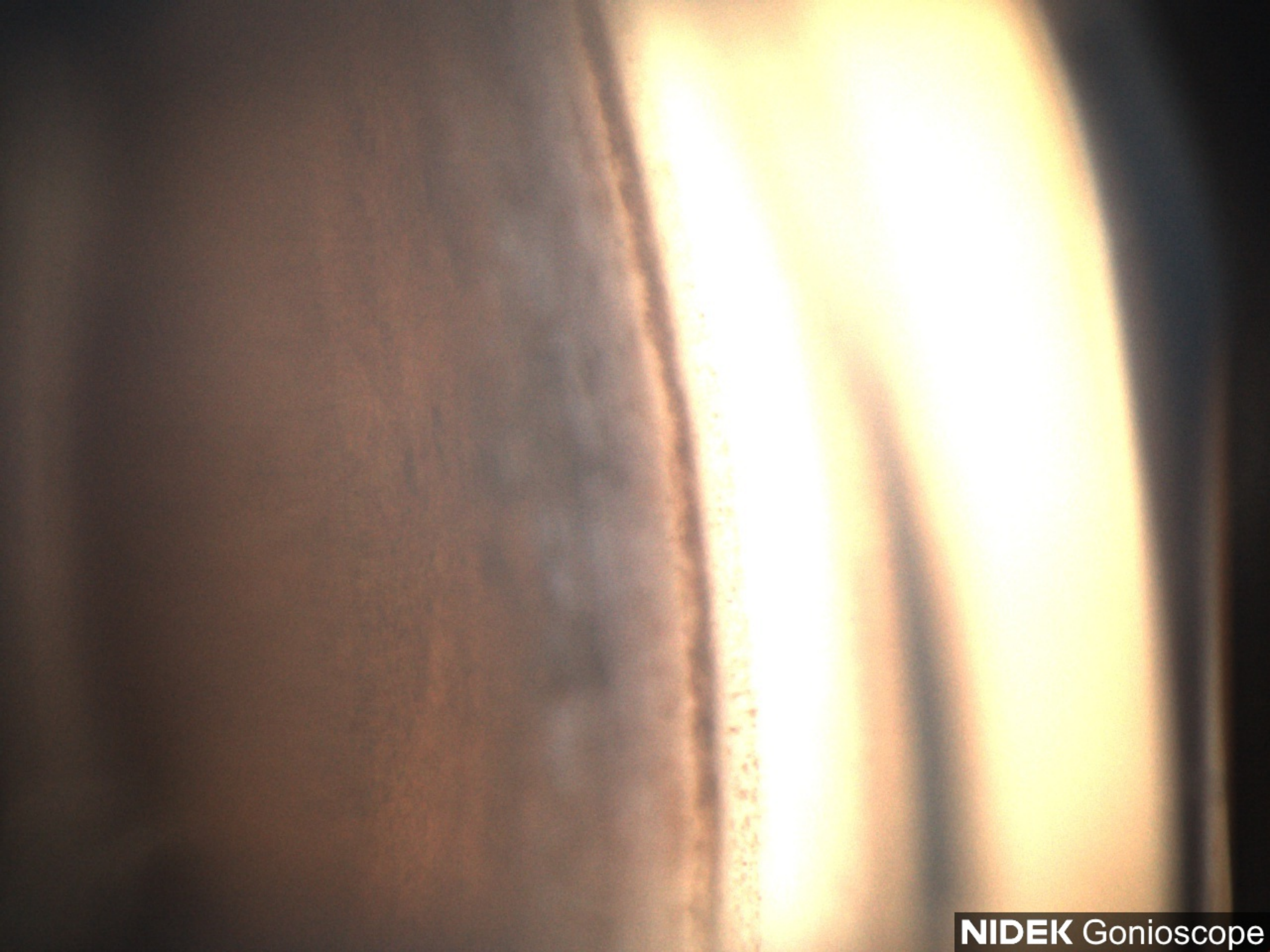

**NIDEK** Gonioscope

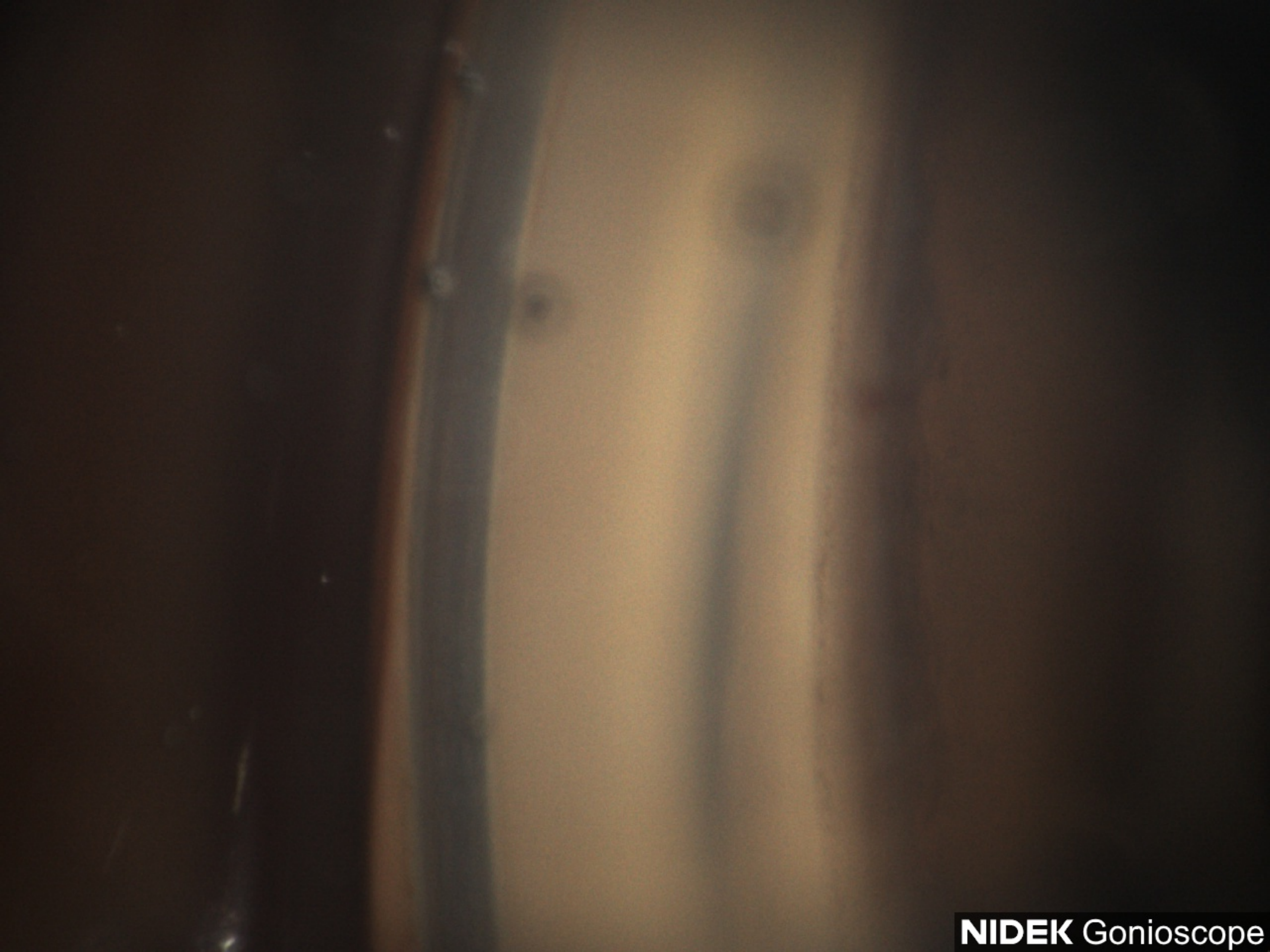



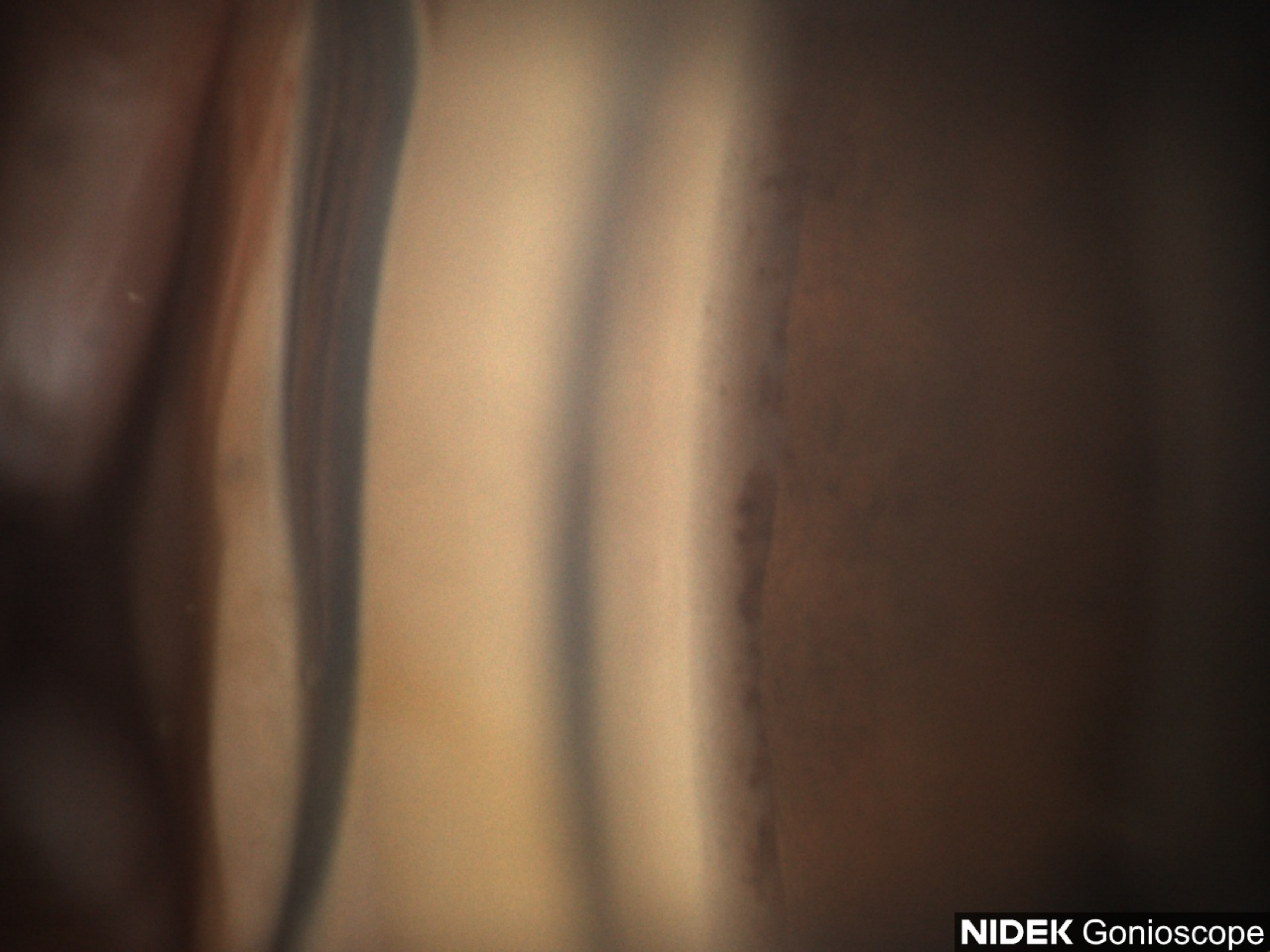

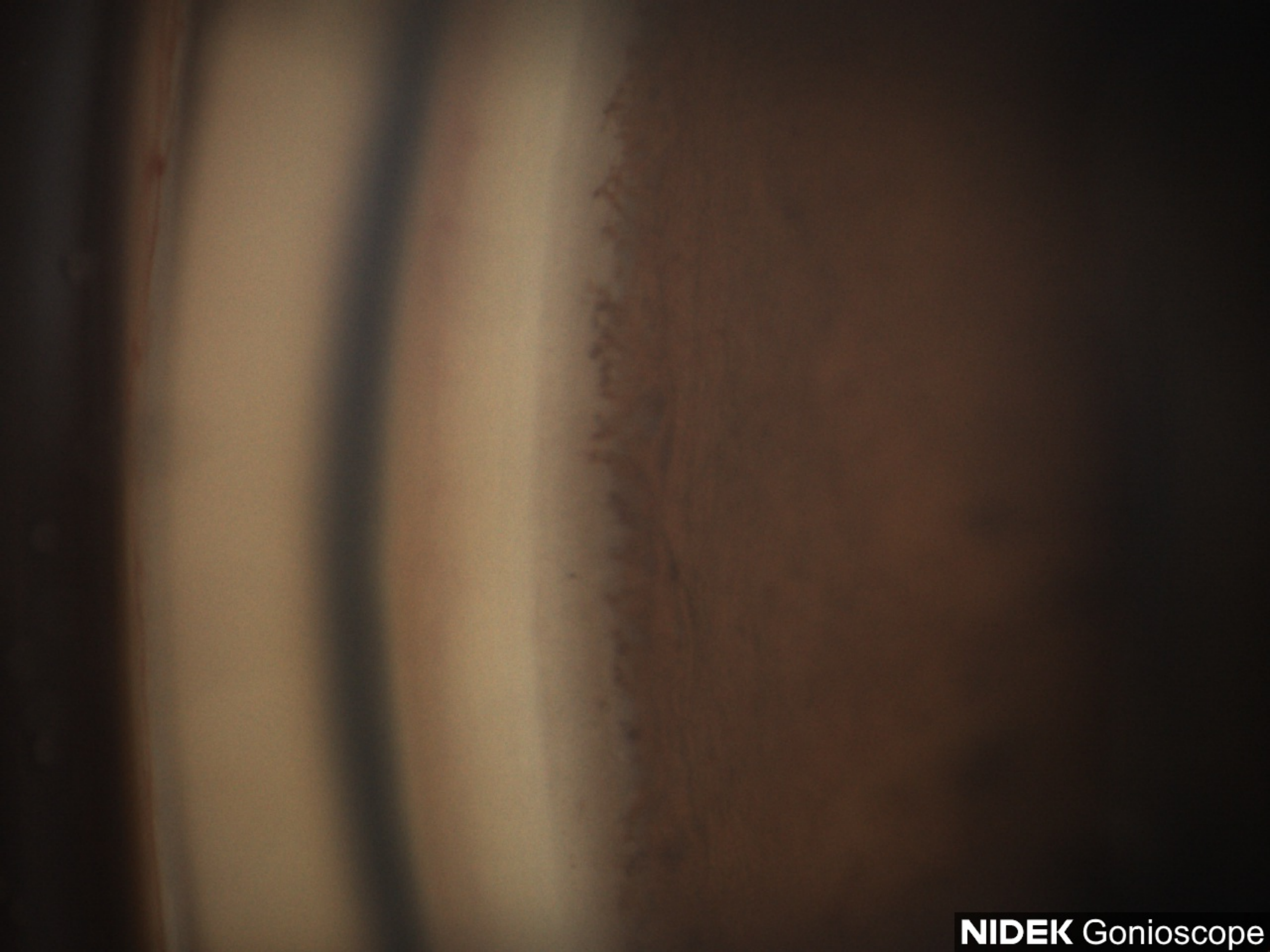

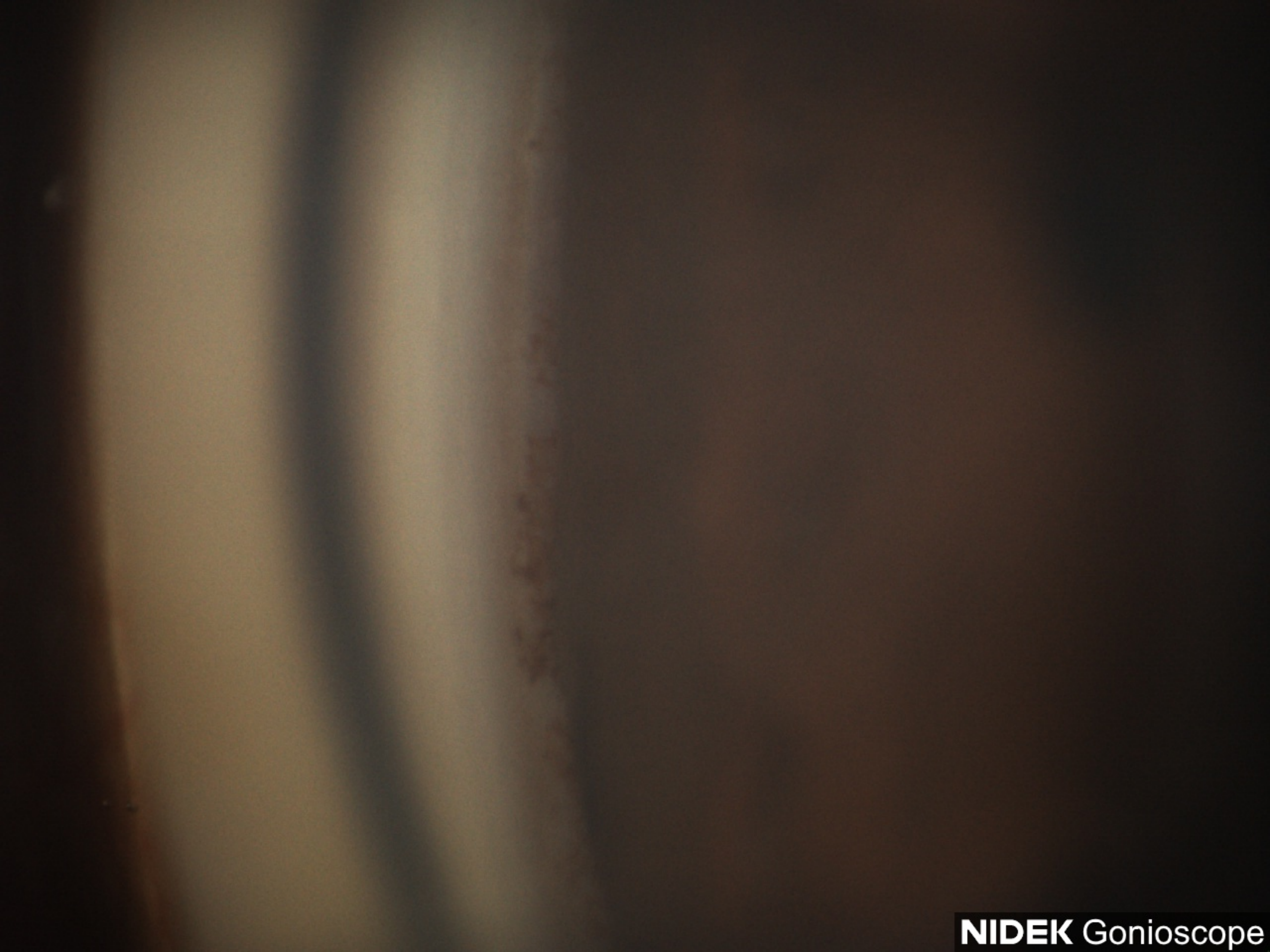

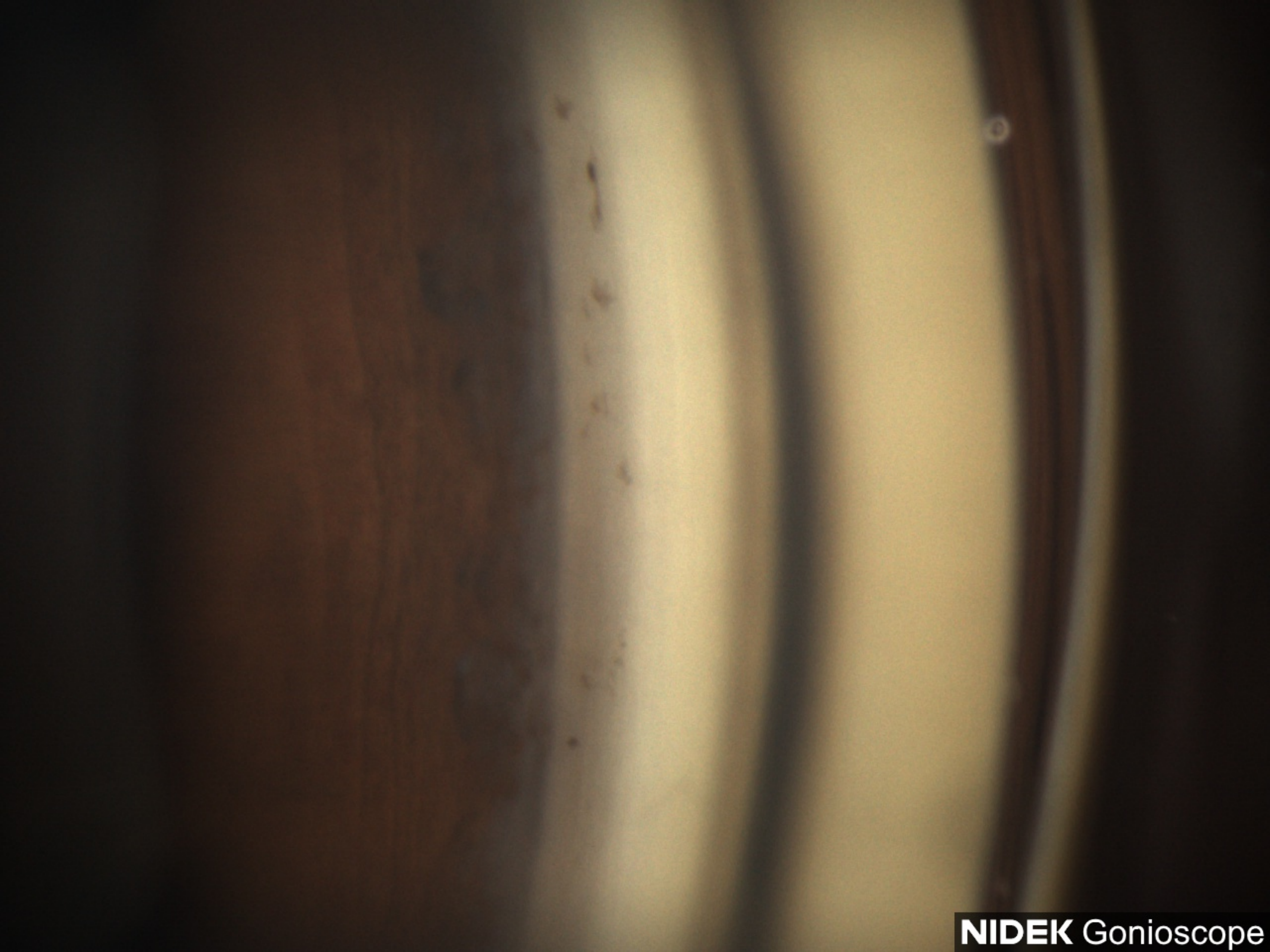

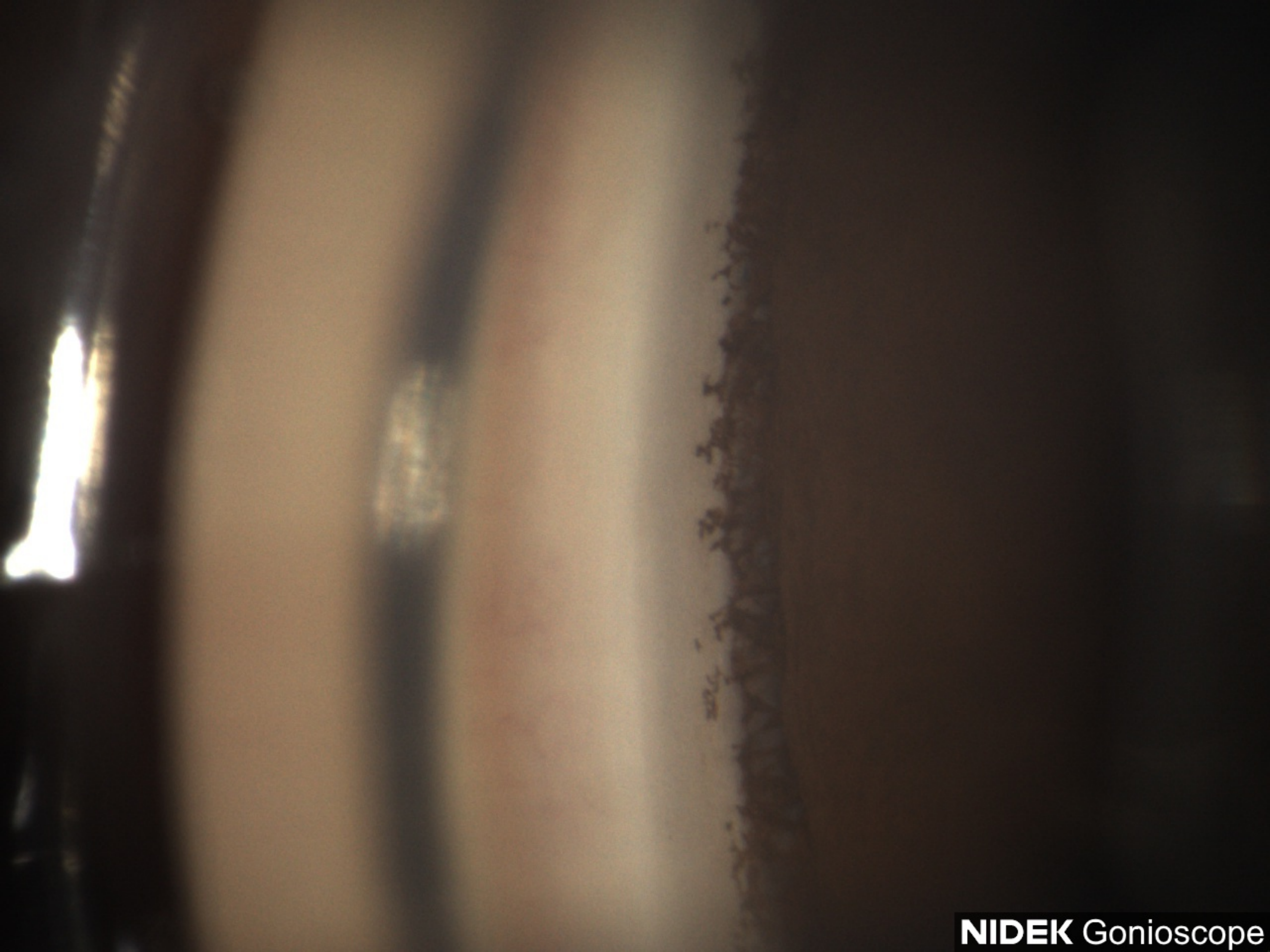

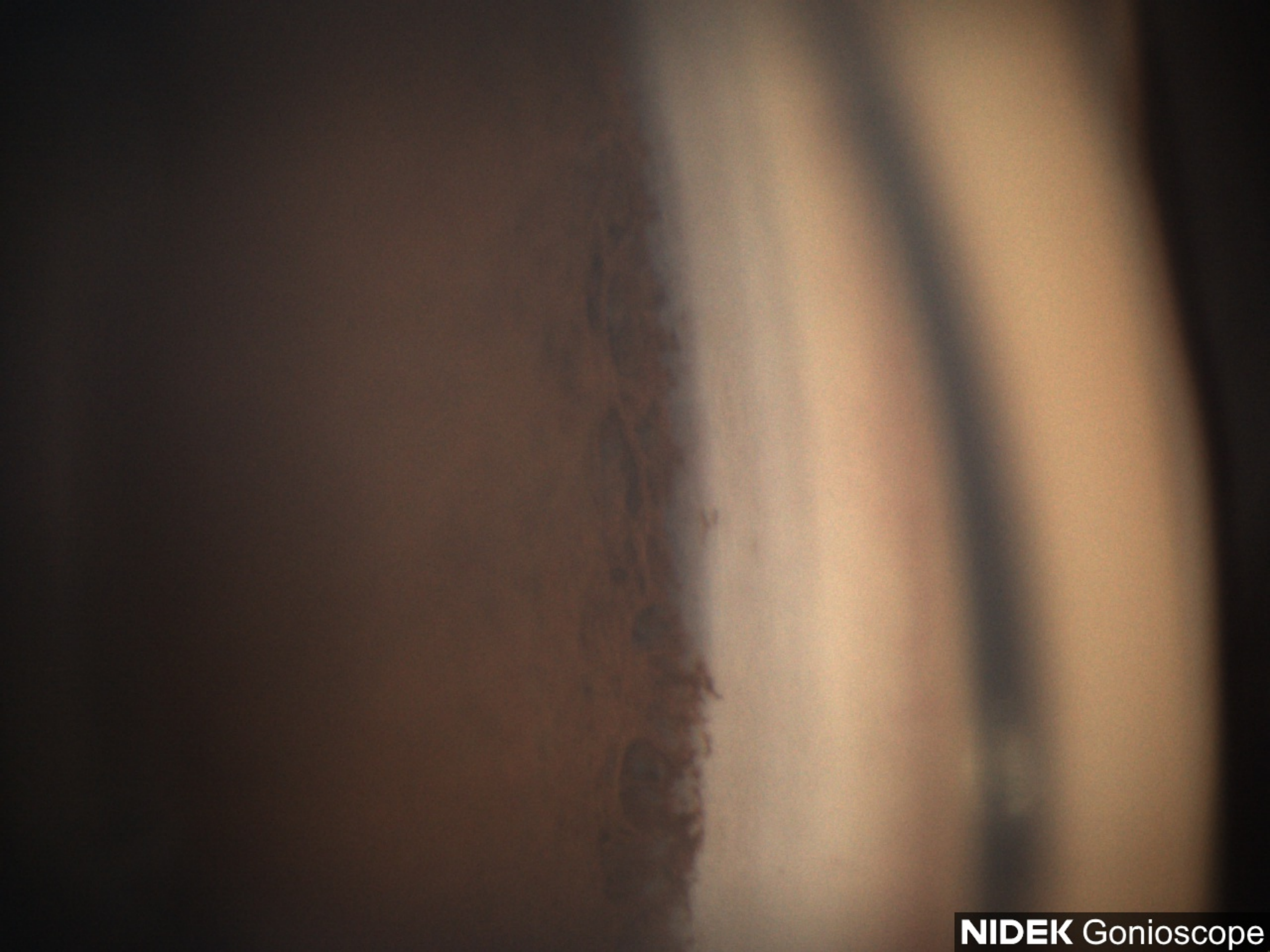

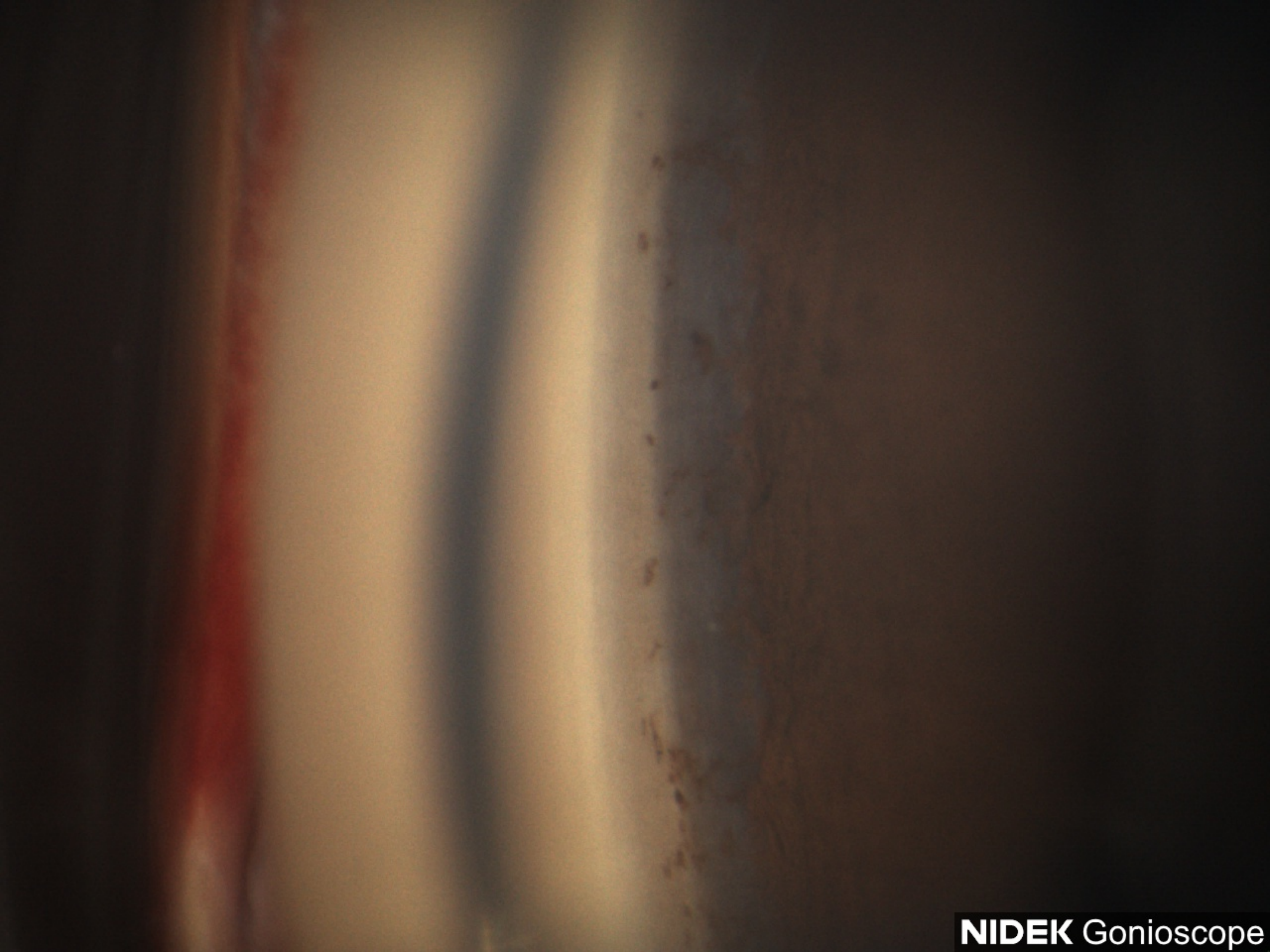

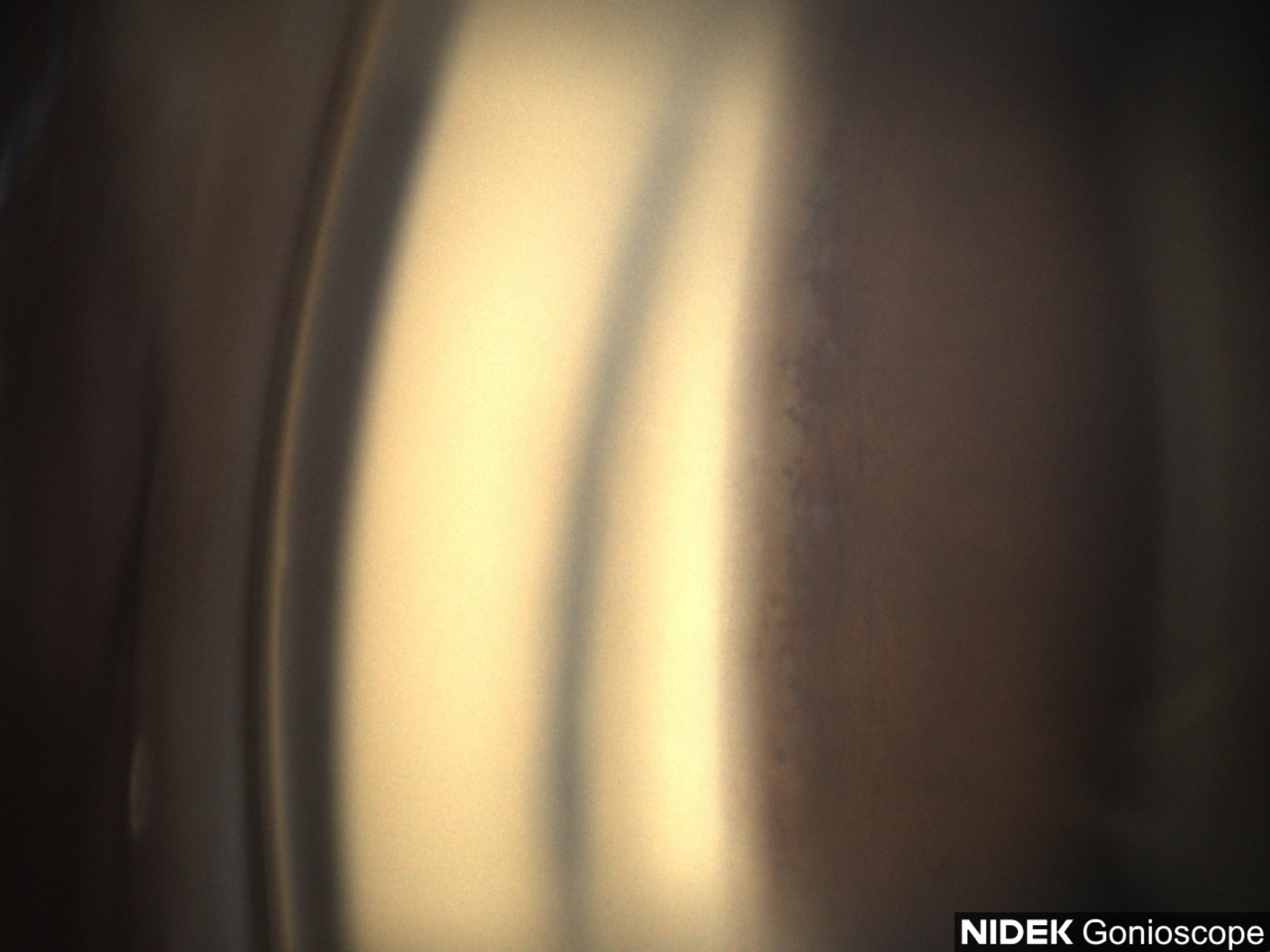

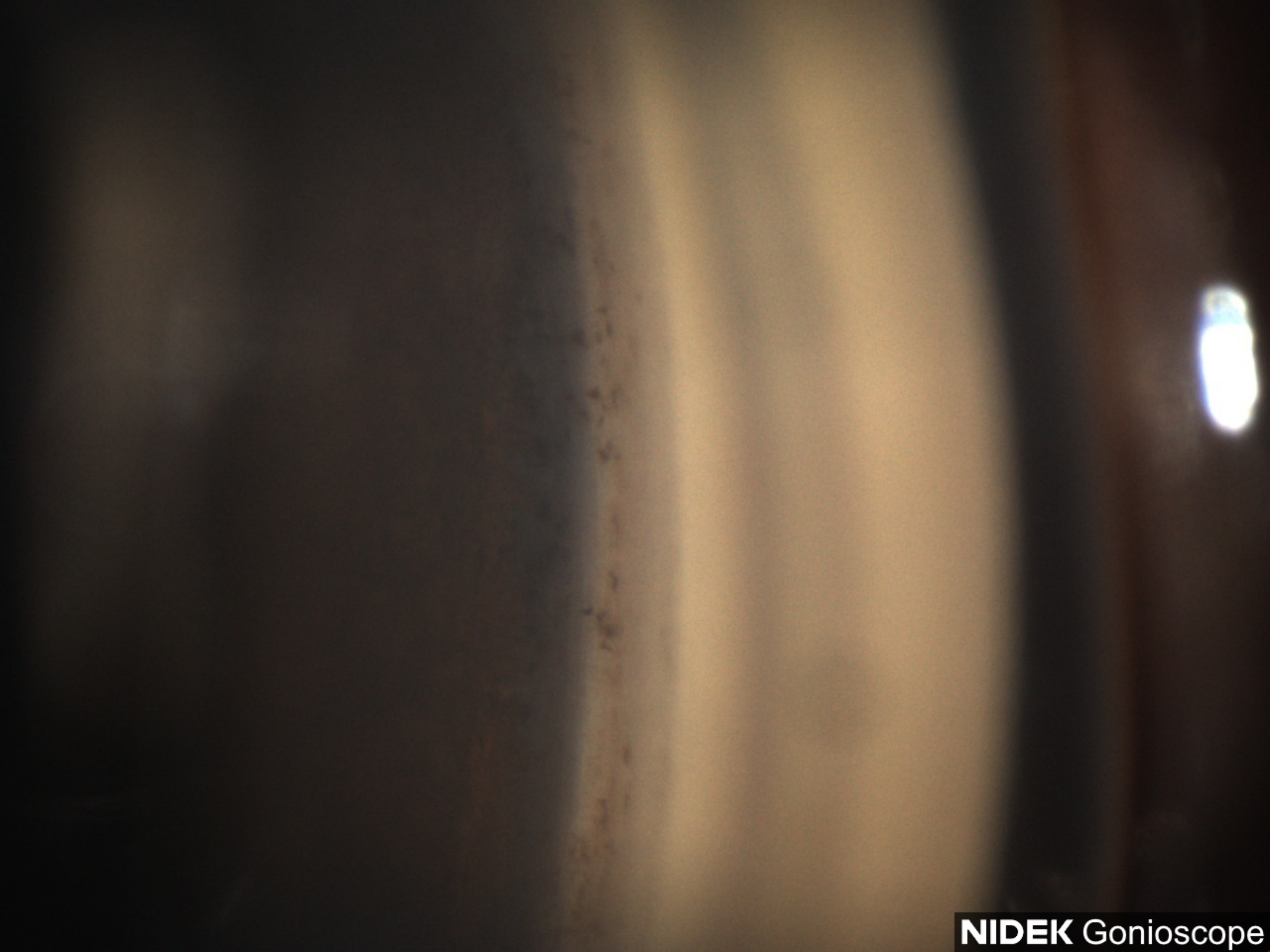

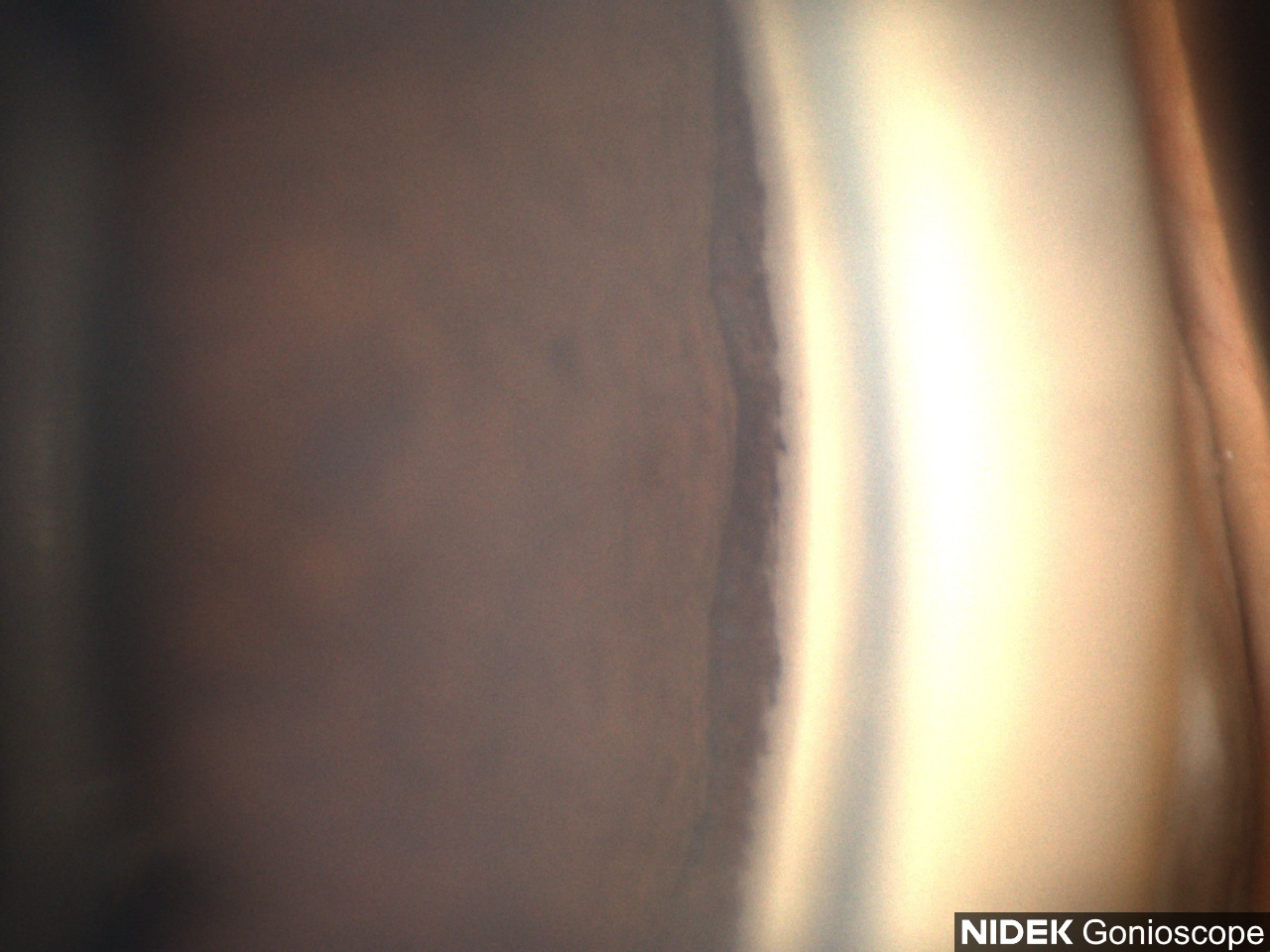

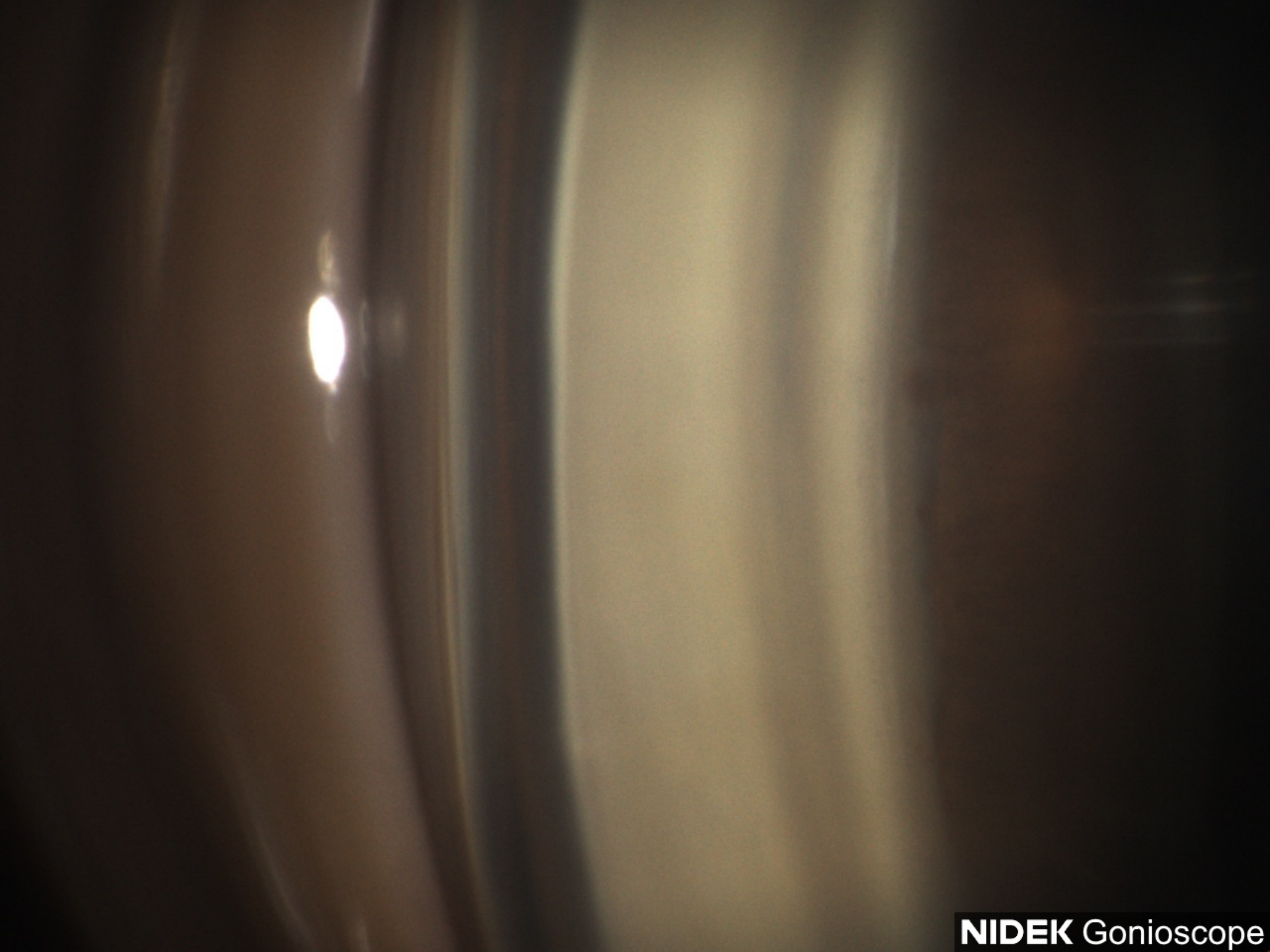

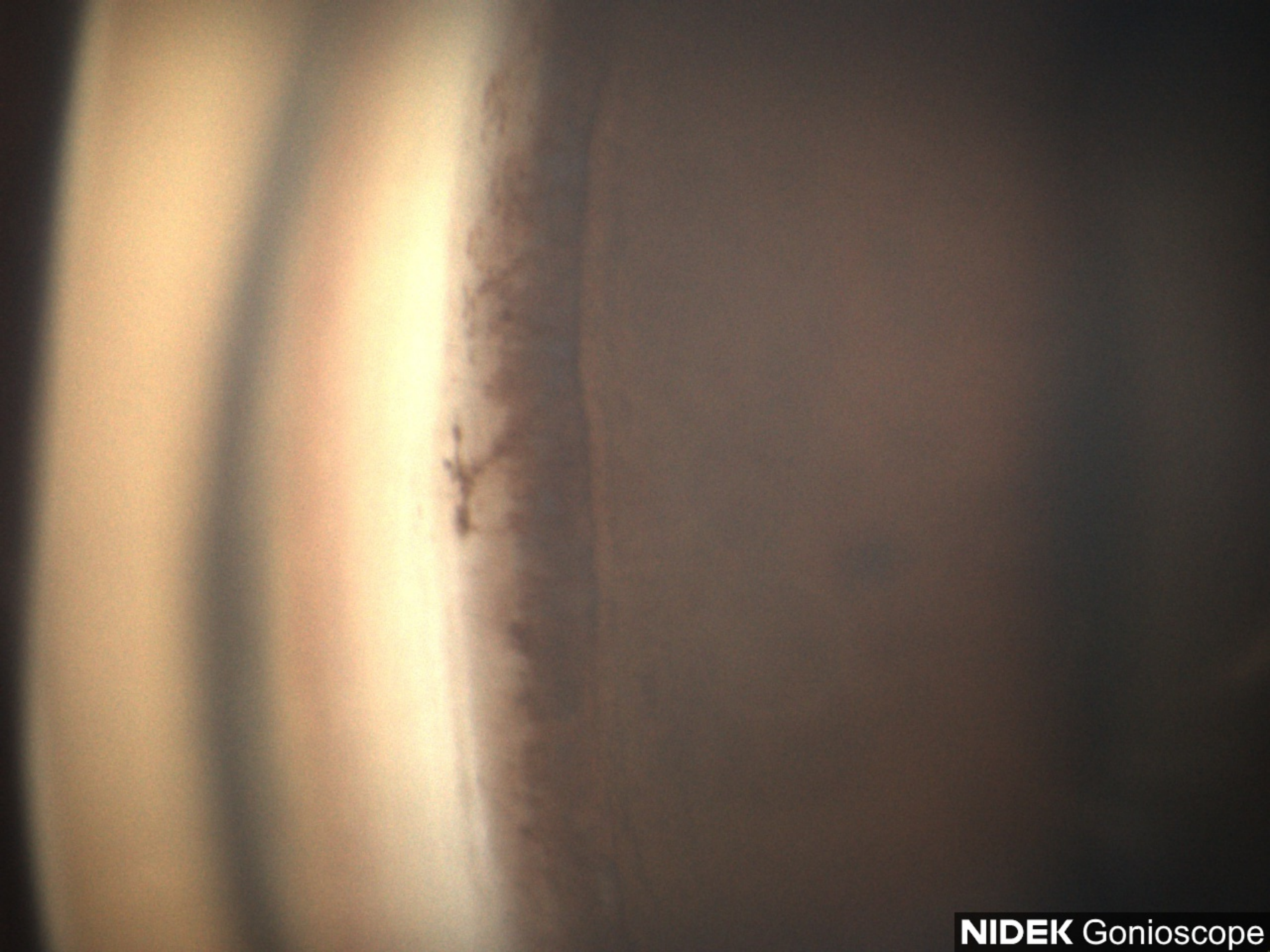

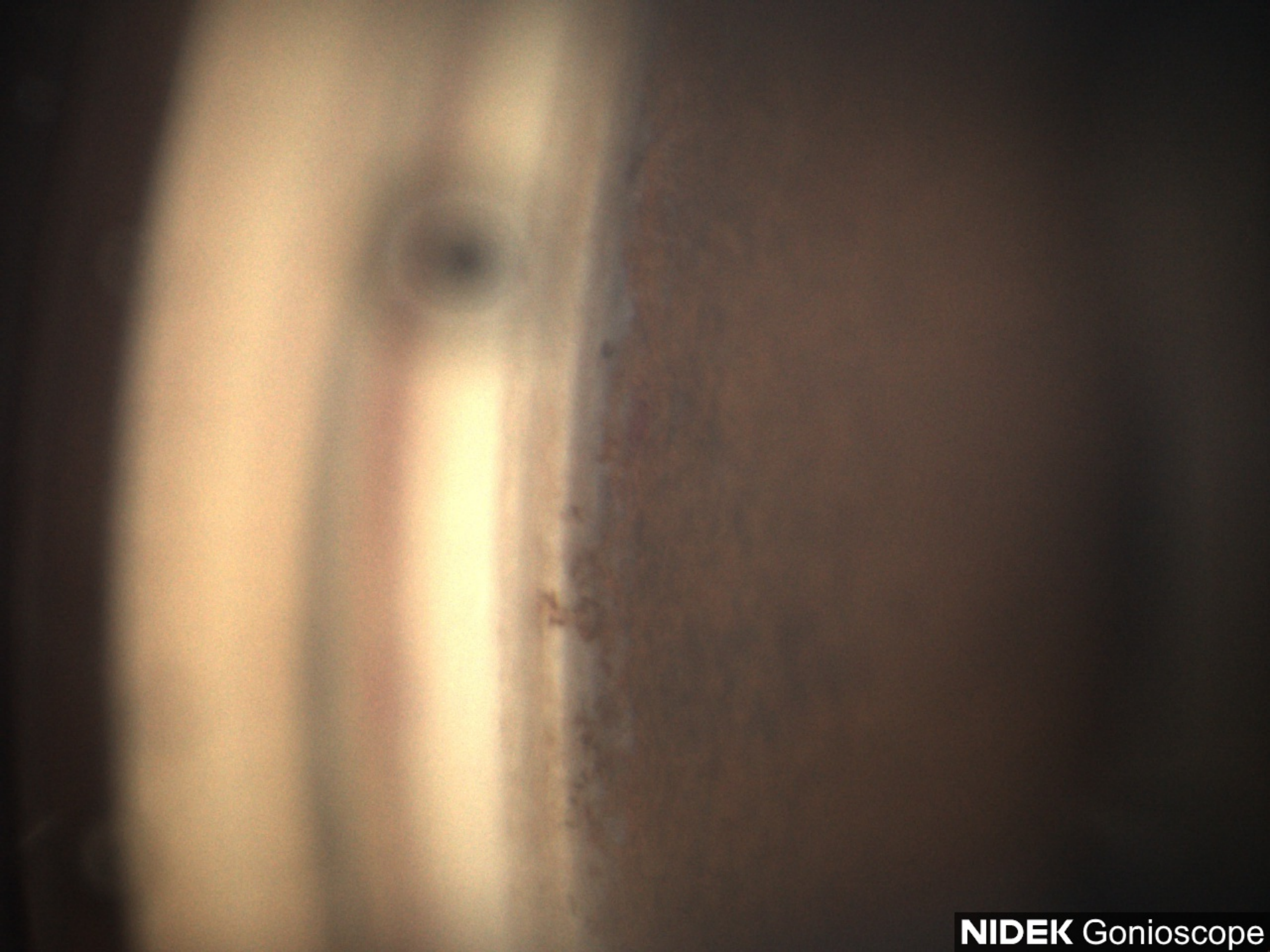

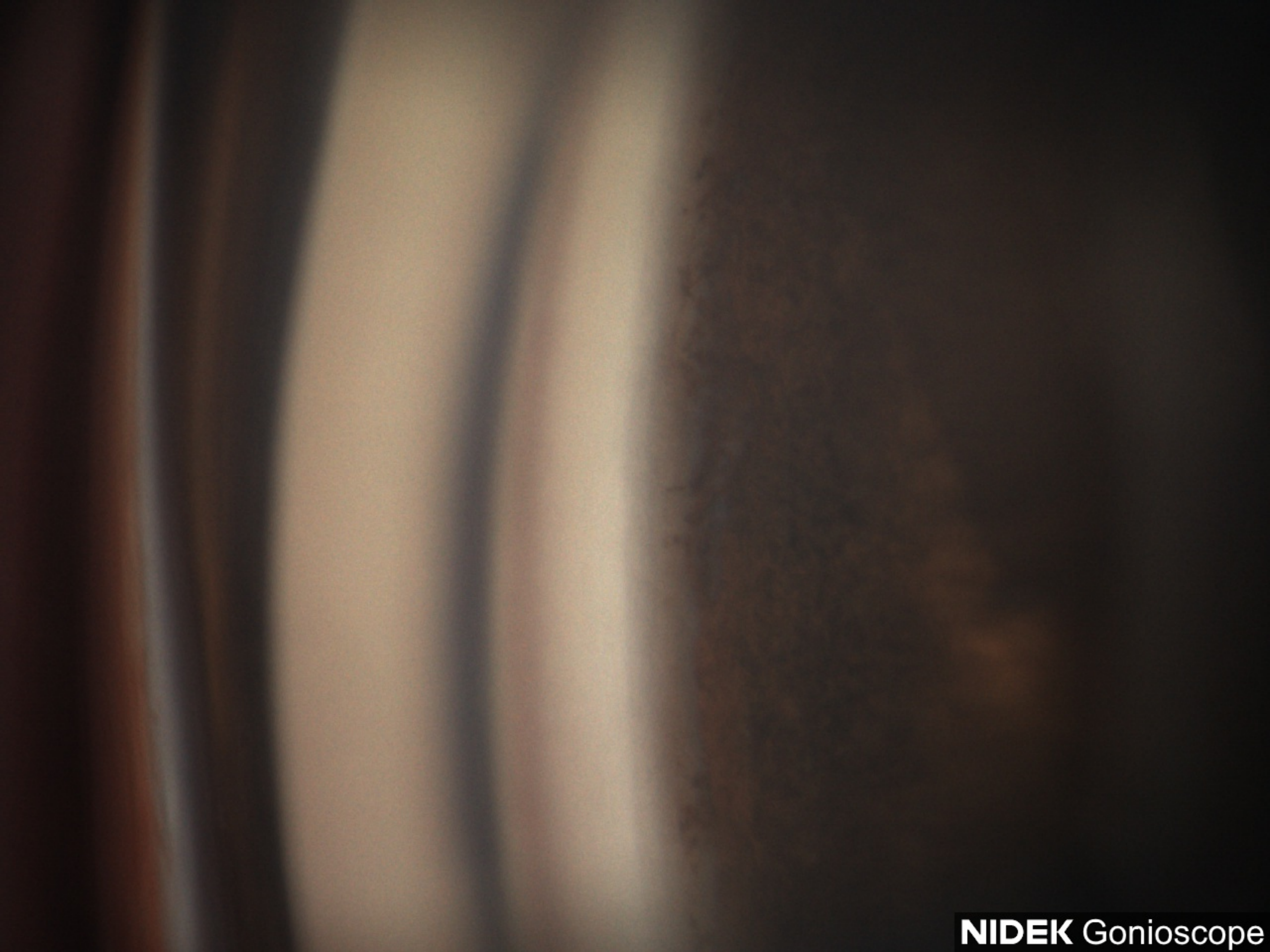





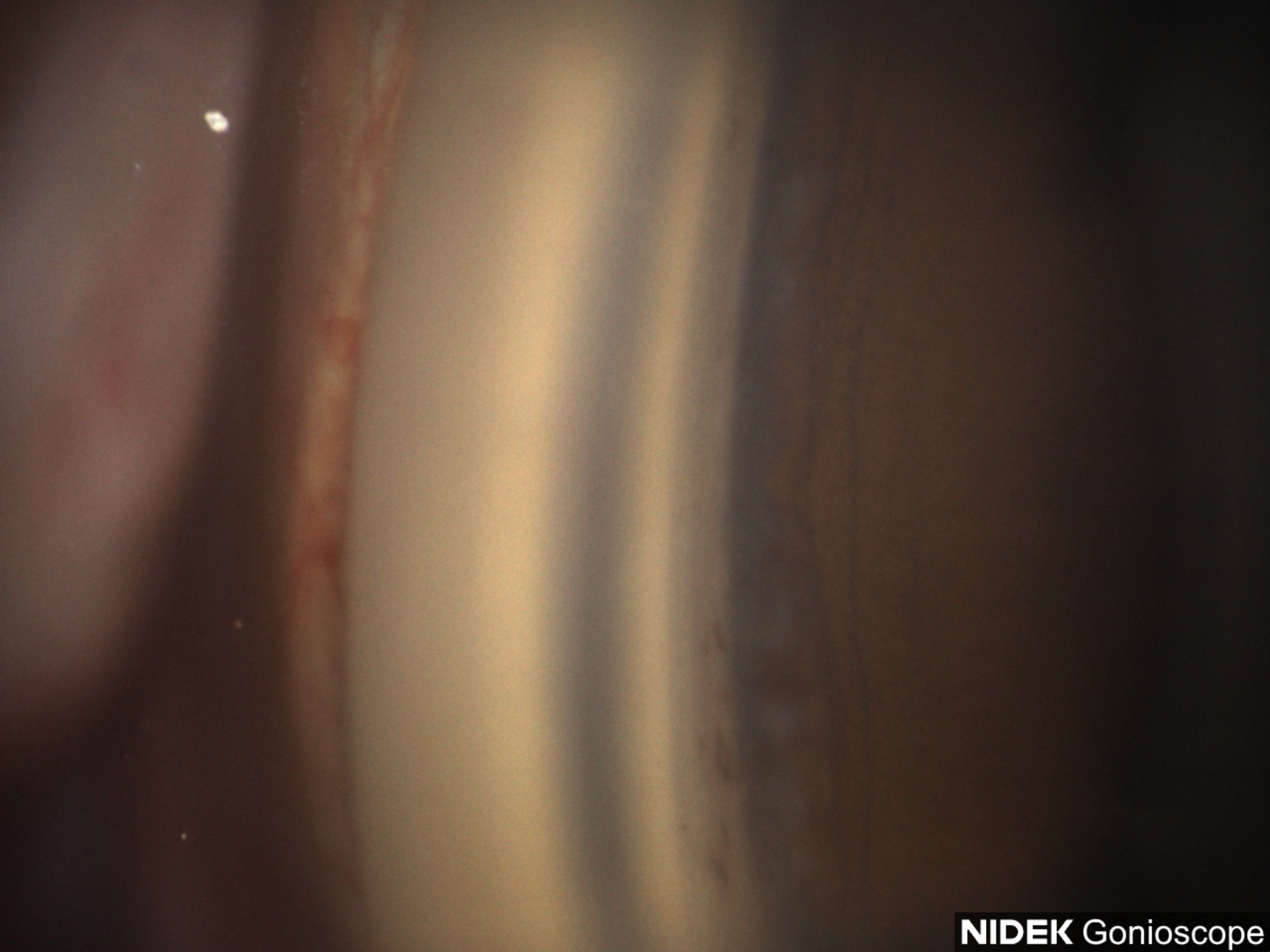

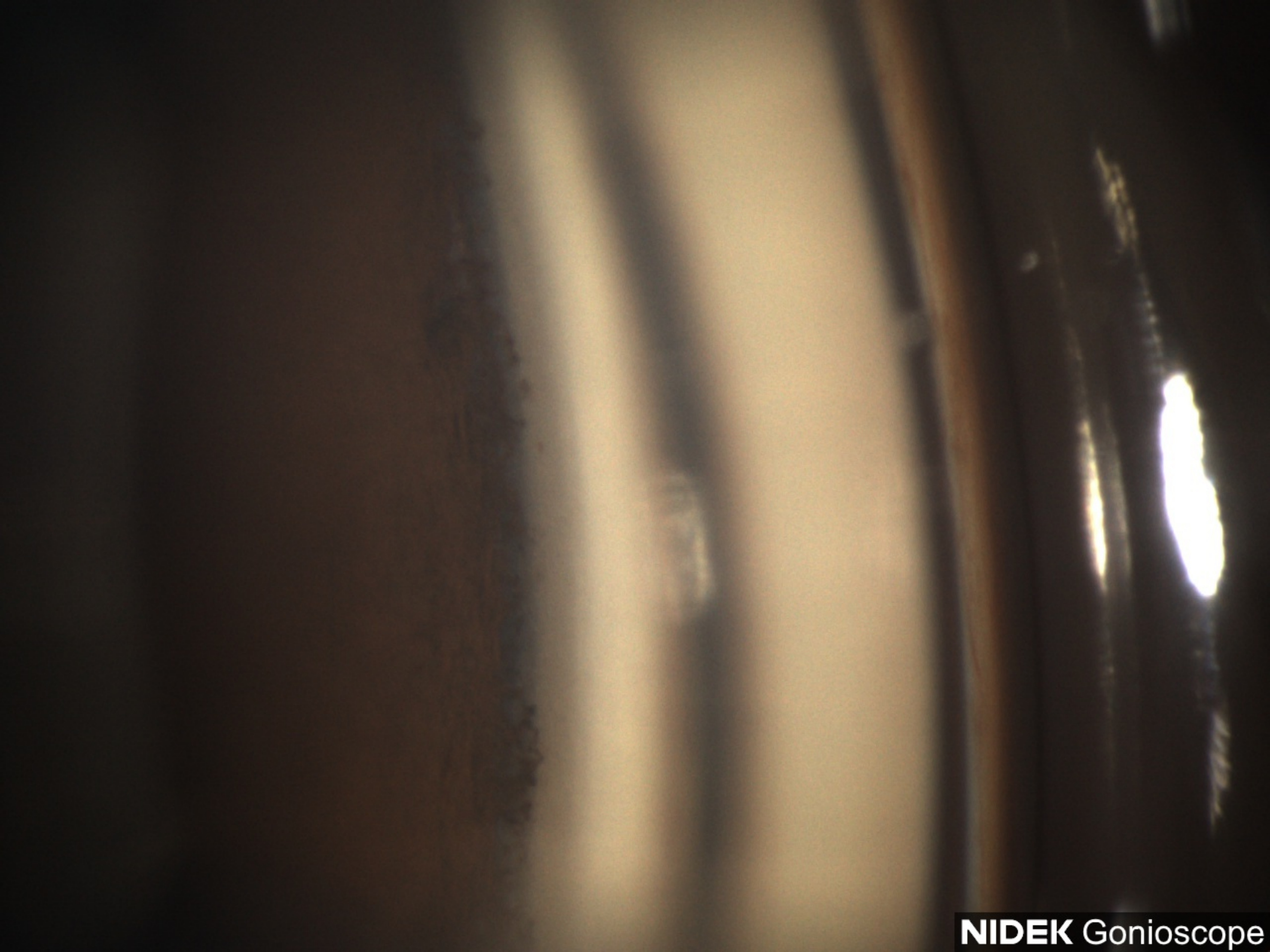

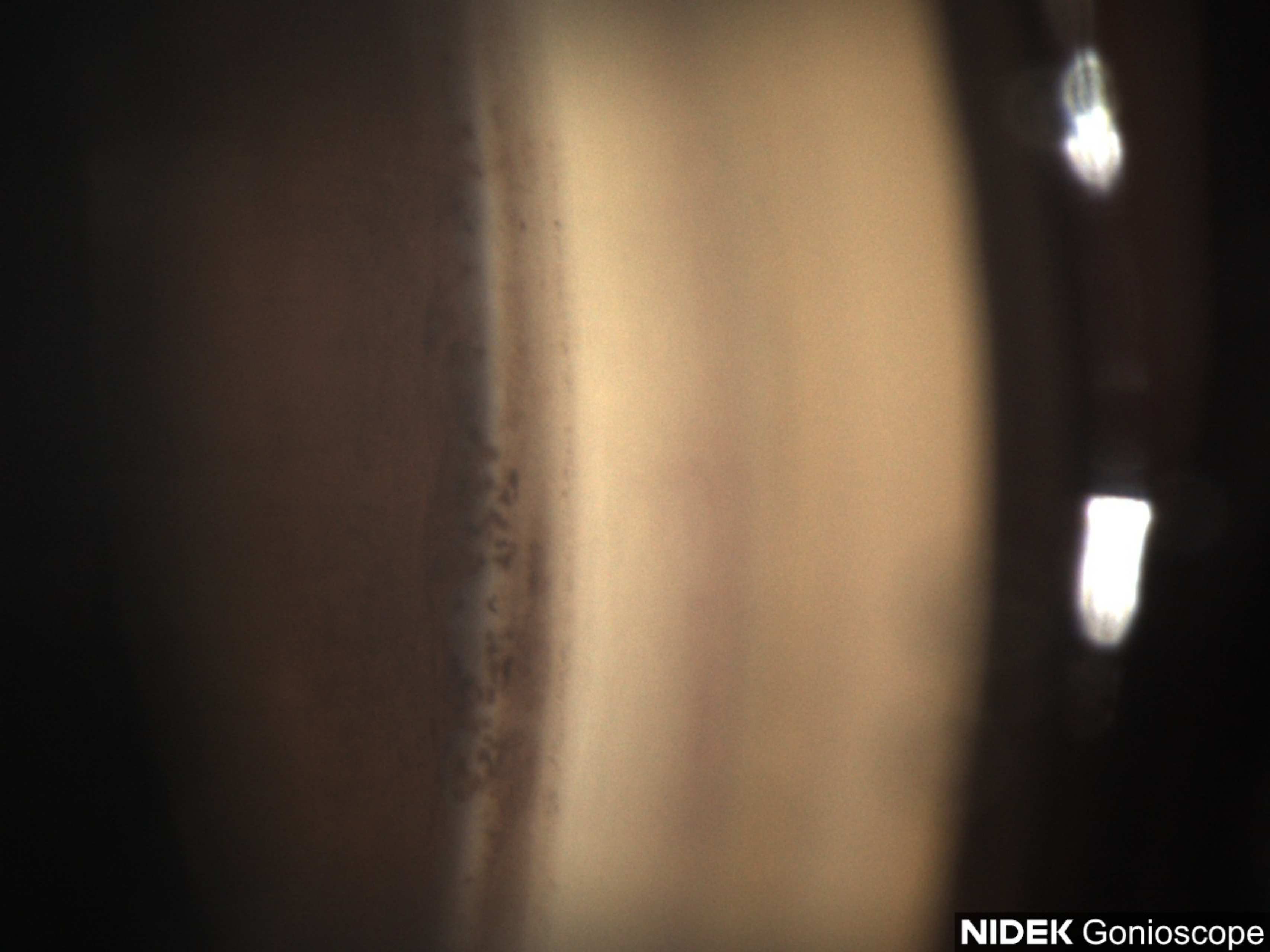

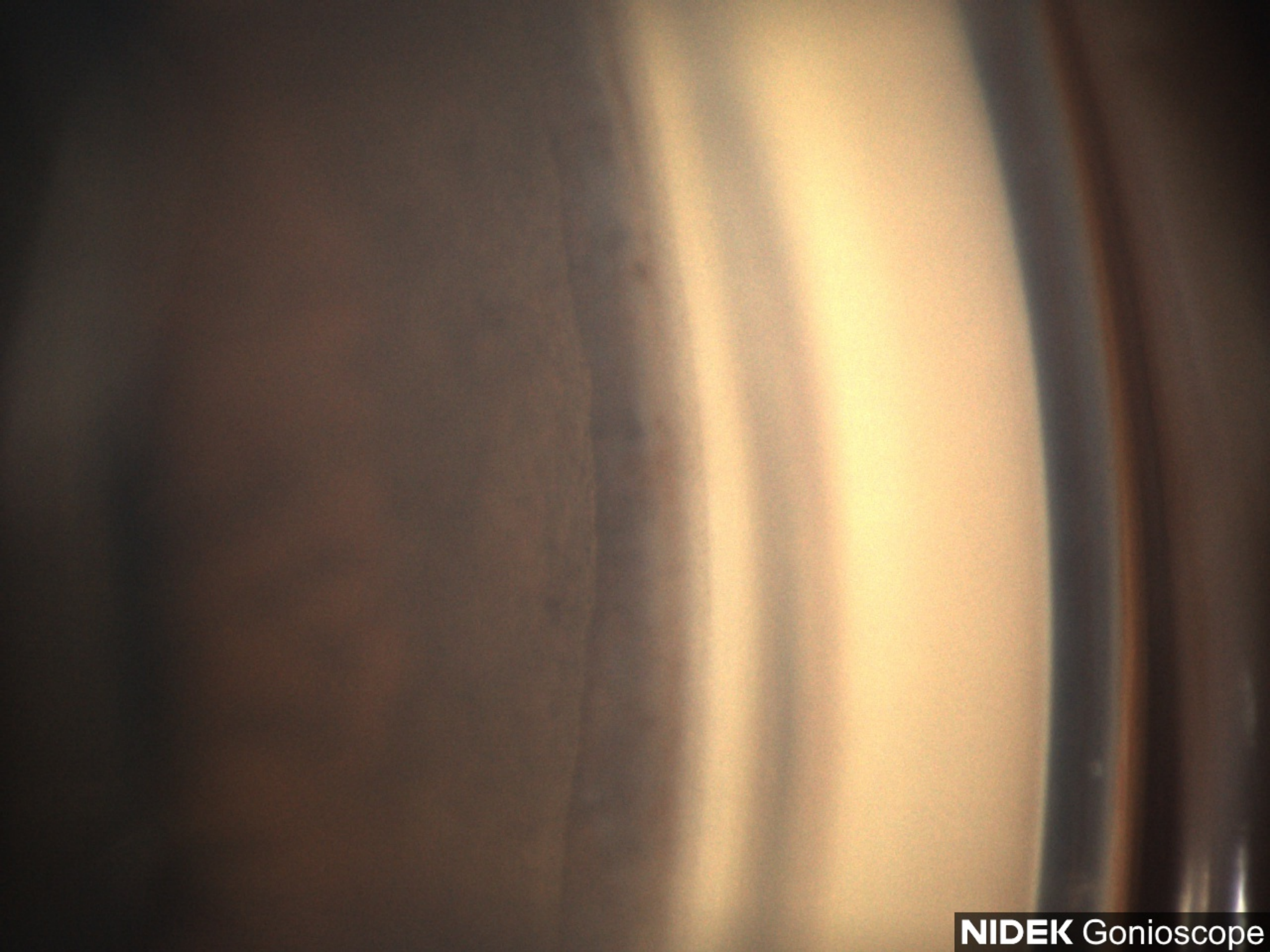

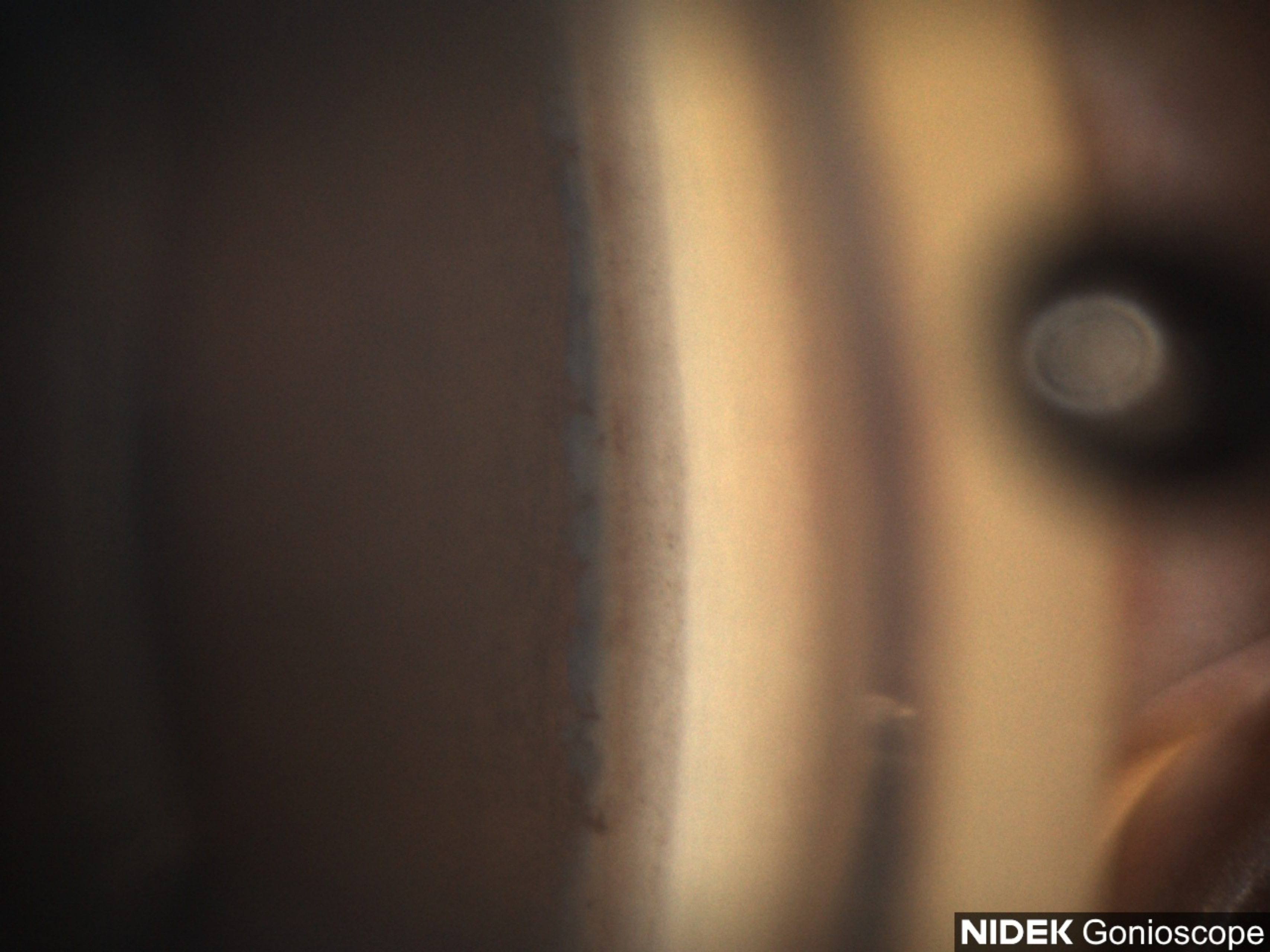

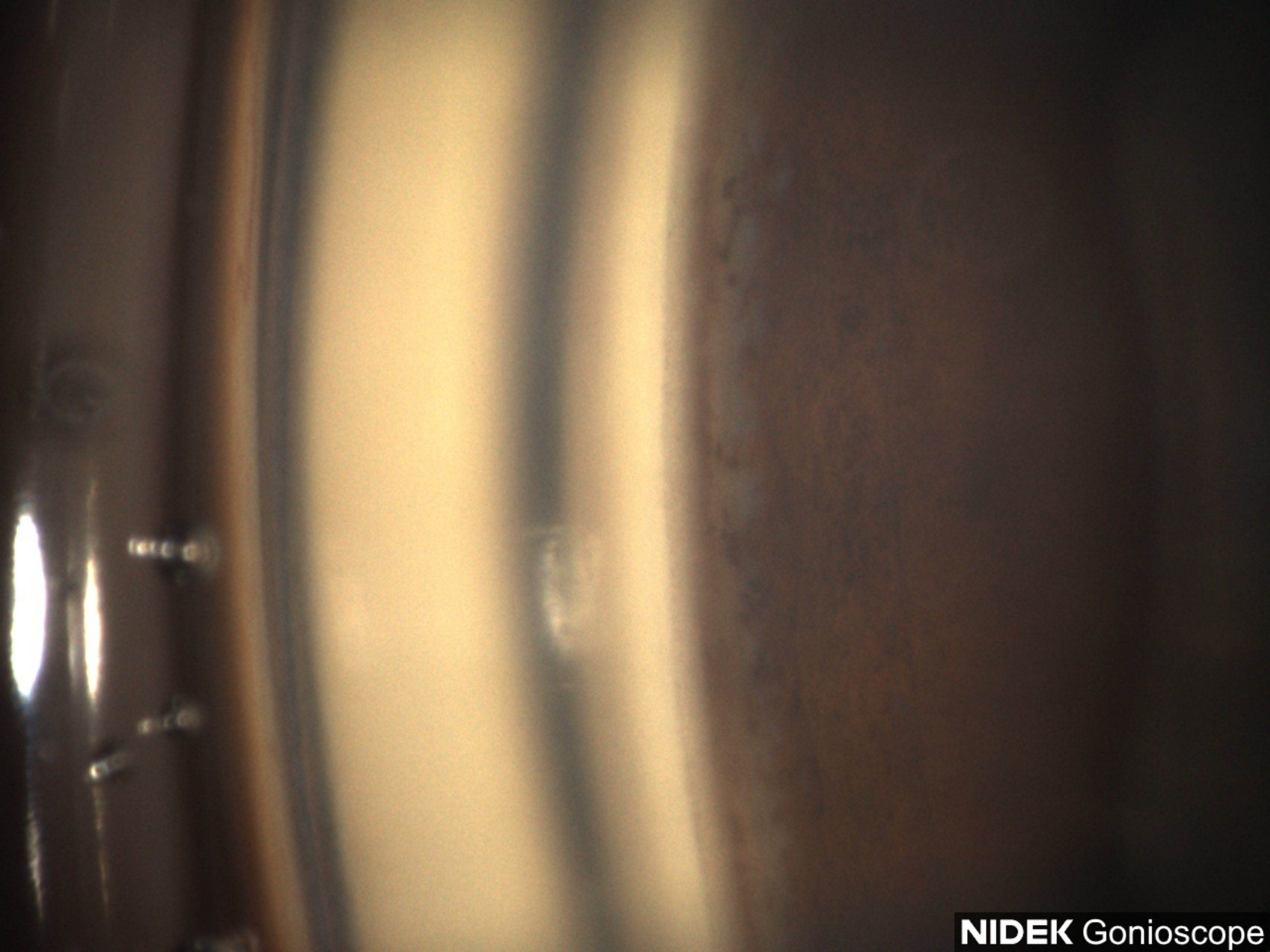

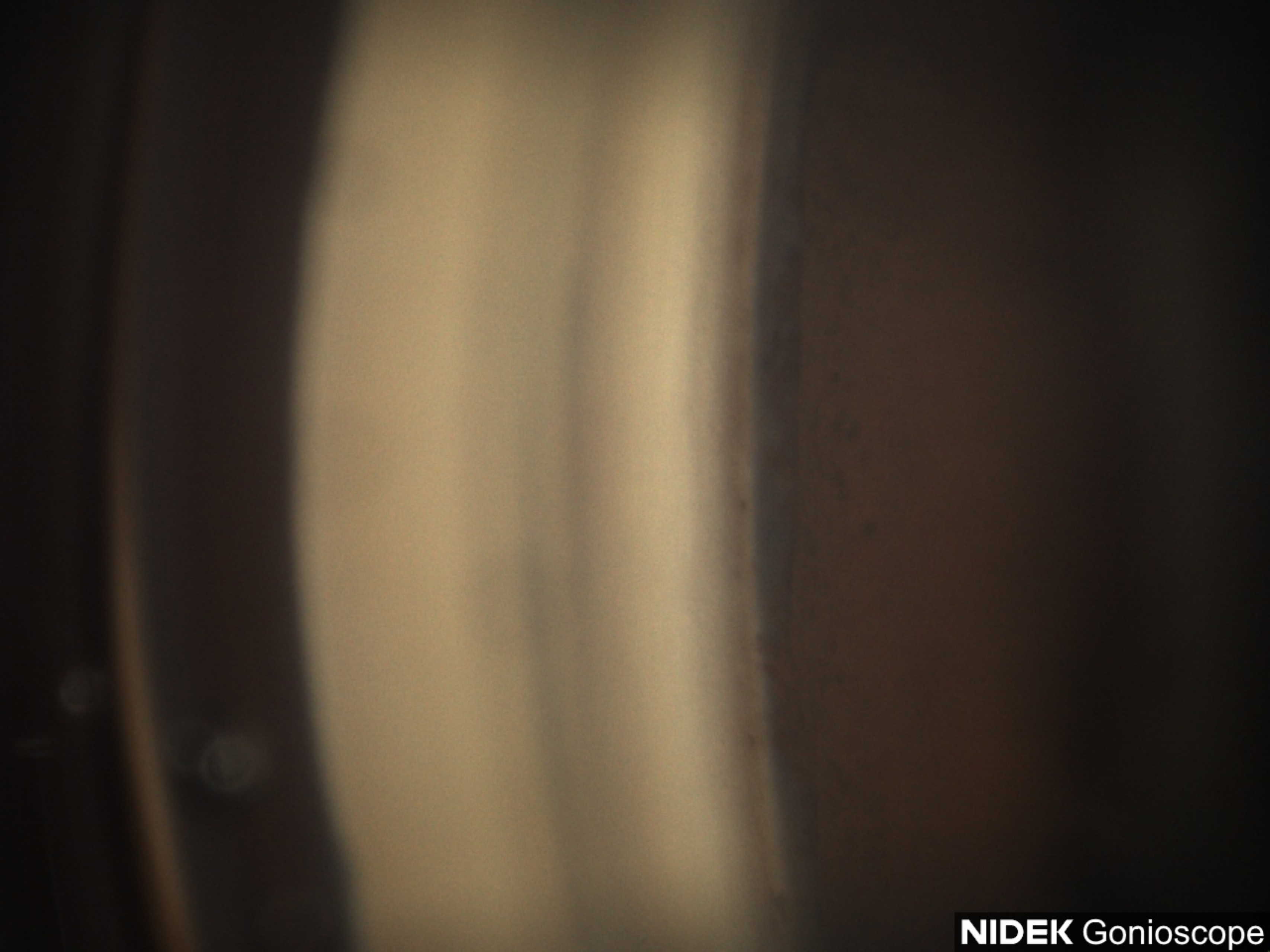

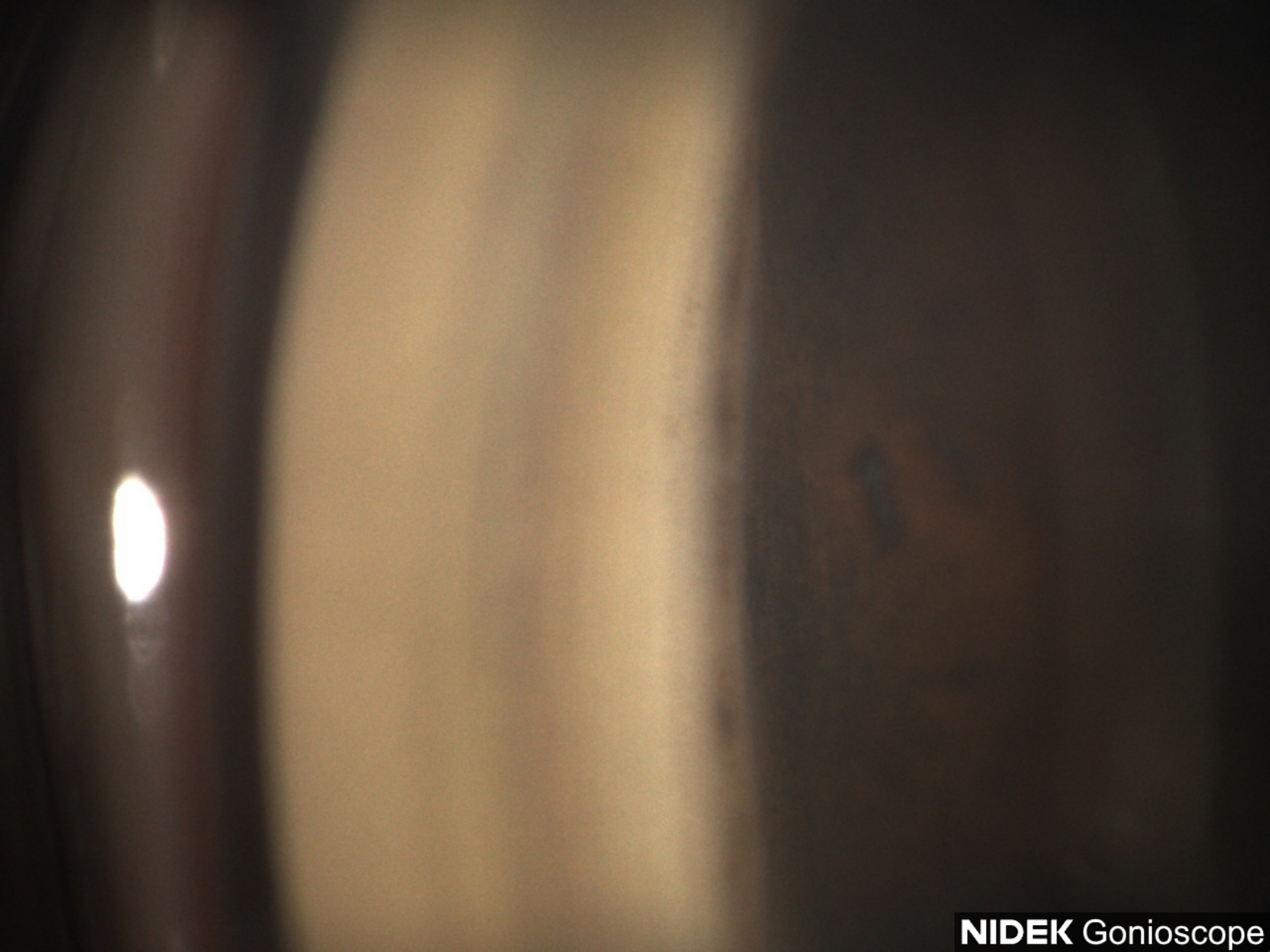

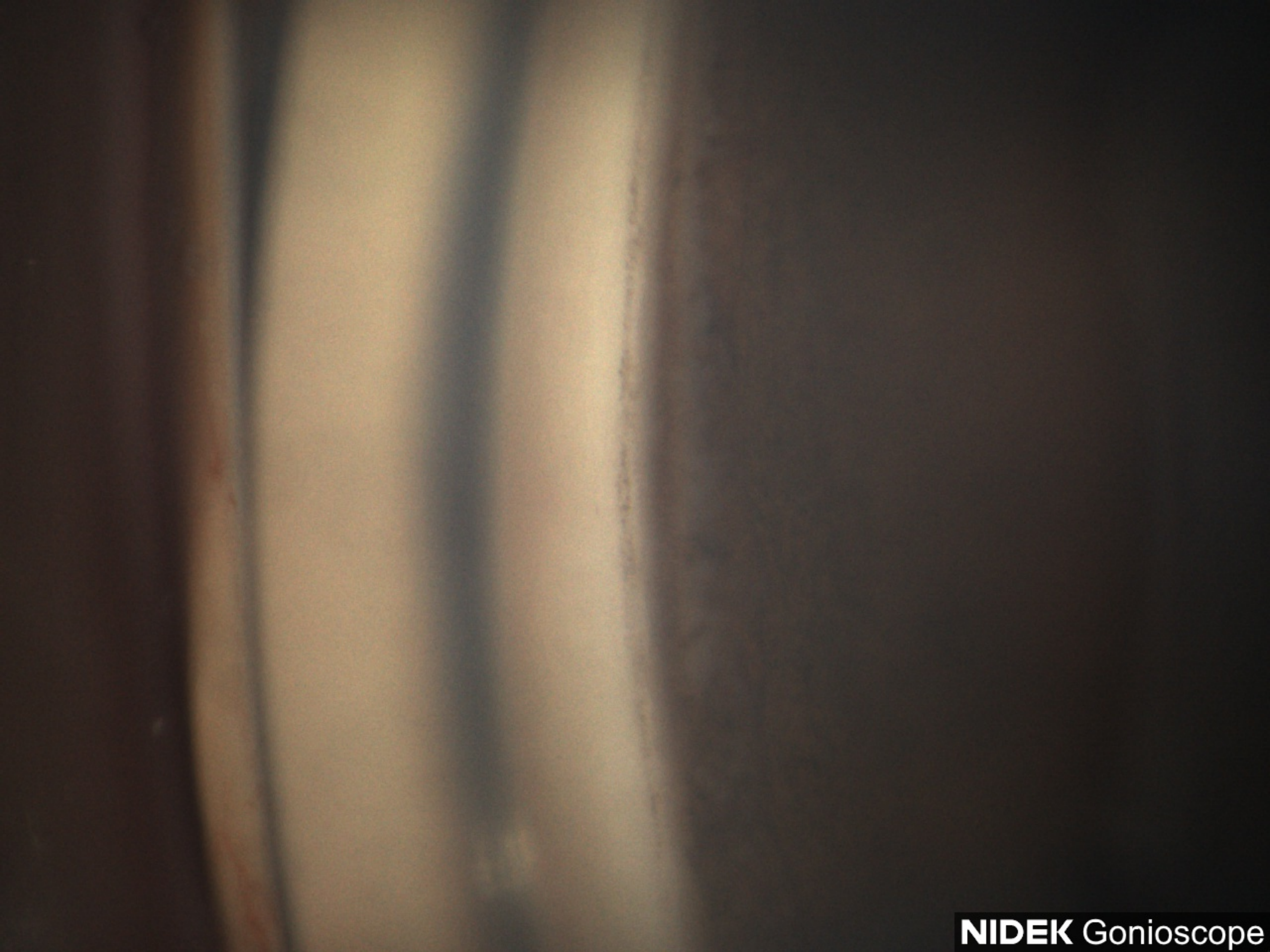

Supplement: S2 Data — (PDF) [file pone.0251249.s007.pdf]
