# Supplementary material for: Intraobserver and interobserver agreement among anterior chamber angle evaluations using automated 360-degree gonio-photos
Source: PLoS One. 2021 May 6;16(5):e0251249. doi: 10.1371/journal.pone.0251249 (PMC8101769; doi:10.1371/journal.pone.0251249)
Supplement: S1 Table — (DOCX) [file pone.0251249.s002.docx]

**S1 Table. Comparison of manual gonioscopy and automated gonioscope in all angle gradings.**

|  | **Automated gonioscope** | | | | |
| --- | --- | --- | --- | --- | --- |
|  | **Grade 0** | **Grade 1** | **Grade 2** | **Grade 3** | **Grade 4** |
| **Scheie's angle width grading with manual gonioscopy** |  |  |  |  |  |
| Grade 0 | 47 | 18 | 4 | 1 | 0 |
| Grade 1 | 23 | 12 | 11 | 2 | 1 |
| Grade 2 | 0 | 5 | 8 | 1 | 0 |
| Grade 3 | 0 | 0 | 0 | 2 | 3 |
| Grade 4 | 0 | 0 | 0 | 1 | 1 |
| **Scheie's angle pigmentation grading with manual gonioscopy** |  |  |  |  |  |
| Grade 0 | 16 | 20 | 1 | 0 | 0 |
| Grade 1 | 7 | 34 | 9 | 3 | 0 |
| Grade 2 | 1 | 5 | 19 | 9 | 2 |
| Grade 3 | 0 | 0 | 0 | 7 | 1 |
| Grade 4 | 0 | 0 | 0 | 0 | 1 |
