# Supplementary material for: Intraobserver and interobserver agreement among anterior chamber angle evaluations using automated 360-degree gonio-photos
Source: PLoS One. 2021 May 6;16(5):e0251249. doi: 10.1371/journal.pone.0251249 (PMC8101769; doi:10.1371/journal.pone.0251249)
Supplement: S2 Table — (DOCX) [file pone.0251249.s003.docx]

**S2 Table. Comparison of Scheie's angle gradings by glaucoma specialists with automated gonioscope between first and second tests in all images.**

**S2A Table. Comparison of Scheie's angle gradings by observer 1 with automated gonioscope between first and second tests in all images.**

|  | **First test** | | | | |
| --- | --- | --- | --- | --- | --- |
|  | **Grade 0** | **Grade 1** | **Grade 2** | **Grade 3** | **Grade 4** |
| **Scheie's angle width grading in second test** |  |  |  |  |  |
| Grade 0 | 61 | 6 | 0 | 0 | 0 |
| Grade 1 | 9 | 24 | 4 | 2 | 0 |
| Grade 2 | 0 | 5 | 18 | 2 | 2 |
| Grade 3 | 0 | 0 | 1 | 3 | 1 |
| Grade 4 | 0 | 0 | 0 | 0 | 2 |
| **Scheie's angle pigmentation grading in second test** |  |  |  |  |  |
| Grade 0 | 16 | 3 | 0 | 0 | 0 |
| Grade 1 | 7 | 48 | 2 | 0 | 0 |
| Grade 2 | 1 | 8 | 23 | 5 | 0 |
| Grade 3 | 0 | 0 | 4 | 14 | 2 |
| Grade 4 | 0 | 0 | 0 | 0 | 2 |

**S2B Table. Comparison of Scheie's angle gradings by observer 2 with automated gonioscope between first and second tests in all images.**

|  | **First test** | | | | |
| --- | --- | --- | --- | --- | --- |
|  | **Grade 0** | **Grade 1** | **Grade 2** | **Grade 3** | **Grade 4** |
| **Scheie's angle width grading in second test** |  |  |  |  |  |
| Grade 0 | 86 | 5 | 0 | 0 | 0 |
| Grade 1 | 11 | 21 | 4 | 0 | 0 |
| Grade 2 | 0 | 0 | 2 | 0 | 0 |
| Grade 3 | 0 | 1 | 0 | 0 | 0 |
| Grade 4 | 0 | 0 | 0 | 2 | 3 |
| **Scheie's angle pigmentation grading in second test** |  |  |  |  |  |
| Grade 0 | 5 | 1 | 0 | 0 | 0 |
| Grade 1 | 6 | 80 | 5 | 0 | 0 |
| Grade 2 | 0 | 3 | 18 | 4 | 0 |
| Grade 3 | 0 | 0 | 0 | 8 | 0 |
| Grade 4 | 0 | 0 | 0 | 0 | 0 |

**S2C Table. Comparison of Scheie's angle gradings by observer 3 with automated gonioscope between first and second tests in all images.**

|  | **First test** | | | | |
| --- | --- | --- | --- | --- | --- |
|  | **Grade 0** | **Grade 1** | **Grade 2** | **Grade 3** | **Grade 4** |
| **Scheie's angle width grading in second test** |  |  |  |  |  |
| Grade 0 | 88 | 12 | 2 | 0 | 0 |
| Grade 1 | 12 | 7 | 4 | 0 | 0 |
| Grade 2 | 3 | 3 | 1 | 4 | 0 |
| Grade 3 | 1 | 0 | 0 | 2 | 0 |
| Grade 4 | 0 | 0 | 0 | 0 | 1 |
| **Scheie's angle pigmentation grading in second test** |  |  |  |  |  |
| Grade 0 | 11 | 22 | 5 | 2 | 0 |
| Grade 1 | 4 | 40 | 17 | 4 | 0 |
| Grade 2 | 0 | 3 | 12 | 6 | 1 |
| Grade 3 | 0 | 2 | 4 | 5 | 0 |
| Grade 4 | 0 | 0 | 2 | 0 | 0 |

Observer 1, 2, 3 = glaucoma specialists.
