# Supplementary material for: Intraobserver and interobserver agreement among anterior chamber angle evaluations using automated 360-degree gonio-photos
Source: PLoS One. 2021 May 6;16(5):e0251249. doi: 10.1371/journal.pone.0251249 (PMC8101769; doi:10.1371/journal.pone.0251249)
Supplement: S3 Table — (DOCX) [file pone.0251249.s004.docx]

**S3 Table.** **Comparison of Scheie's angle gradings with automated gonioscope between a glaucoma specialist and the others in first test.**

**S3A Table. Comparison of Scheie's angle gradings with automated gonioscope between observer 1 and the other glaucoma specialists in first test.**

|  | **Observer 1** | | | | |
| --- | --- | --- | --- | --- | --- |
|  | **Grade 0** | **Grade 1** | **Grade 2** | **Grade 3** | **Grade 4** |
| **Scheie's angle width grading by observer 2** |  |  |  |  |  |
| Grade 0 | 68 | 25 | 5 | 1 | 0 |
| Grade 1 | 2 | 9 | 13 | 3 | 0 |
| Grade 2 | 0 | 0 | 3 | 2 | 1 |
| Grade 3 | 0 | 1 | 0 | 0 | 1 |
| Grade 4 | 0 | 0 | 0 | 0 | 3 |
| **Scheie's angle width grading by observer 3** |  |  |  |  |  |
| Grade 0 | 67 | 27 | 7 | 3 | 0 |
| Grade 1 | 2 | 8 | 10 | 2 | 0 |
| Grade 2 | 1 | 0 | 5 | 0 | 1 |
| Grade 3 | 0 | 0 | 1 | 2 | 3 |
| Grade 4 | 0 | 0 | 0 | 0 | 1 |
| **Scheie's angle pigmentation grading by observer 2** |  |  |  |  |  |
| Grade 0 | 5 | 4 | 1 | 0 | 0 |
| Grade 1 | 17 | 53 | 14 | 1 | 0 |
| Grade 2 | 0 | 0 | 14 | 10 | 0 |
| Grade 3 | 0 | 0 | 0 | 8 | 4 |
| Grade 4 | 0 | 0 | 0 | 0 | 0 |
| **Scheie's angle pigmentation grading by observer 3** |  |  |  |  |  |
| Grade 0 | 7 | 7 | 0 | 1 | 0 |
| Grade 1 | 16 | 43 | 3 | 1 | 0 |
| Grade 2 | 0 | 9 | 21 | 8 | 1 |
| Grade 3 | 1 | 0 | 5 | 9 | 2 |
| Grade 4 | 0 | 0 | 0 | 0 | 1 |

**S3B Table. Comparison of Scheie's angle gradings with automated gonioscope between observer 2 and the other glaucoma specialist in first test.**

|  | **Observer 2** | | | | |
| --- | --- | --- | --- | --- | --- |
|  | **Grade 0** | **Grade 1** | **Grade 2** | **Grade 3** | **Grade 4** |
| **Scheie's angle width grading by observer 3** |  |  |  |  |  |
| Grade 0 | 87 | 13 | 2 | 0 | 0 |
| Grade 1 | 11 | 9 | 1 | 1 | 0 |
| Grade 2 | 1 | 4 | 1 | 0 | 1 |
| Grade 3 | 0 | 1 | 2 | 1 | 1 |
| Grade 4 | 0 | 0 | 0 | 0 | 1 |
| **Scheie's angle pigmentation grading by observer 3** |  |  |  |  |  |
| Grade 0 | 3 | 10 | 1 | 0 | 0 |
| Grade 1 | 8 | 54 | 1 | 0 | 0 |
| Grade 2 | 0 | 21 | 13 | 4 | 0 |
| Grade 3 | 0 | 0 | 9 | 7 | 0 |
| Grade 4 | 0 | 0 | 0 | 1 | 0 |

Observer 1, 2, 3 = glaucoma specialists.
