# Supplementary material for: Intraobserver and interobserver agreement among anterior chamber angle evaluations using automated 360-degree gonio-photos
Source: PLoS One. 2021 May 6;16(5):e0251249. doi: 10.1371/journal.pone.0251249 (PMC8101769; doi:10.1371/journal.pone.0251249)
Supplement: S4 Table — (DOCX) [file pone.0251249.s005.docx]

**S4 Table. Effect of image quality on observer agreement for angle evaluations using gonioscopic photos of GS-1.**

| **Parameters** | **Images of grade 0 quality (n = 94)** | | | | **Images of grade 1 quality (n = 46)** | | | |
| --- | --- | --- | --- | --- | --- | --- | --- | --- |
|  | **Fleiss' kappa coefficient** | | **Kendall rank correlation coefficient** | | **Fleiss' kappa coefficient** | | **Kendall rank correlation coefficient** | |
|  | **Kappa (95% CI)** | **Landis-Koch score*** | **Tau** | **P** | **Kappa (95% CI)** | **Landis-Koch score*** | **Tau** | **P** |
| **Intra-observer agreement for Scheie's angle width grading** | | | | | | | | |
| Observer 1 | 0.63 (0.50-0.76) | Substantial agreement | 0.78 | <0.01 | 0.68 (0.51-0.85) | Substantial agreement | 0.84 | <0.01 |
| Observer 2 | 0.65 (0.50-0.80) | Substantial agreement | 0.77 | <0.01 | 0.58 (0.34-0.82) | Moderate agreement | 0.68 | <0.01 |
| Observer 3 | 0.31 (0.16-0.47) | Fair agreement | 0.55 | <0.01 | 0.32 (0.04-0.59) | Fair agreement | 0.32 | 0.02 |
| **Intra-observer agreement for Scheie's angle pigmentation grading** | | | | | | | | |
| Observer 1 | 0.63 (0.51-0.76) | Substantial agreement | 0.82 | <0.01 | 0.72 (0.57-0.88) | Substantial agreement | 0.84 | <0.01 |
| Observer 2 | 0.69 (0.54-0.84) | Substantial agreement | 0.80 | <0.01 | 0.74 (0.55-0.93) | Substantial agreement | 0.81 | <0.01 |
| Observer 3 | 0.20 (0.07-0.34) | Slight agreement | 0.52 | <0.01 | 0.34 (0.16-0.53) | Fair agreement | 0.43 | <0.01 |
| **Intra-observer agreement for PAS detection** | | | | | | | | |
| Observer 1 | 0.45 (0.13-0.77) | Moderate agreement | 0.46 | <0.01 | 0.88 (0.64-1.00) | Almost perfect agreement | 0.88 | <0.01 |
| Observer 2 | 0.82 (0.58-1.00) | Almost perfect agreement | 0.84 | <0.01 | NA | NA | NA | NA |
| Observer 3 | 0.39 (0.11-0.66) | Fair agreement | 0.41 | <0.01 | 0.26 (-0.15-0.67) | Fair agreement | 0.38 | <0.01 |
| **Intra-observer agreement for Sampaolesi line detection** | | | | | | | | |
| Observer 1 | 0.50 (0.29-0.71) | Moderate agreement | 0.54 | <0.01 | 1.00 (1.00-1.00) | Almost perfect agreement | 1.00 | <0.01 |
| Observer 2 | 0.71 (0.40-1.00) | Substantial agreement | 0.74 | <0.01 | 1.00 (1.00-1.00) | Almost perfect agreement | 1.00 | <0.01 |
| Observer 3 | 0.28 (0.03-0.53) | Fair agreement | 0.33 | <0.01 | 0.12 (-0.17-0.41) | Slight agreement | 0.16 | 0.27 |
| **Inter-observer agreement for Scheie's angle width grading** | | | | | | | | |
| Observer 1 vs. Observer 2 | 0.36 (0.23-0.49) | Fair agreement | 0.70 | <0.01 | 0.20 (0.02-0.39) | Slight agreement | 0.55 | <0.01 |
| Observer 1 vs. Observer 3 | 0.36 (0.22-0.50) | Fair agreement | 0.63 | <0.01 | 0.18 (0.02-0.35) | Slight agreement | 0.44 | <0.01 |
| Observer 2 vs. Observer 3 | 0.46 (0.30-0.62) | Moderate agreement | 0.65 | <0.01 | 0.11 (-0.13-0.34) | Slight agreement | 0.23 | 0.11 |
| **Inter-observer agreement for Scheie's angle pigmentation grading** | | | | | | | | |
| Observer 1 vs. Observer 2 | 0.38 (0.24-0.52) | Fair agreement | 0.63 | <0.01 | 0.42 (0.24-0.61) | Moderate agreement | 0.77 | <0.01 |
| Observer 1 vs. Observer 3 | 0.44 (0.29-0.58) | Moderate agreement | 0.71 | <0.01 | 0.40 (0.21-0.58) | Fair agreement | 0.55 | <0.01 |
| Observer 2 vs. Observer 3 | 0.24 (0.10-0.38) | Fair agreement | 0.61 | <0.01 | 0.48 (0.27-0.68) | Moderate agreement | 0.66 | <0.01 |
| **Inter-observer agreement for PAS detection** | | | | | | | | |
| Observer 1 vs. Observer 2 | 0.23 (-0.09-0.55) | Fair agreement | 0.24 | 0.02 | NA | NA | NA | NA |
| Observer 1 vs. Observer 3 | 0.20 (-0.11-0.51) | Slight agreement | 0.21 | 0.05 | -0.04 (-0.09-0.02) | Slight agreement | 0.23 | 0.76 |
| Observer 2 vs. Observer 3 | 0.71 (0.40-1.00) | Substantial agreement | 0.71 | <0.01 | NA | NA | NA | NA |
| **Inter-observer agreement for Sampaolesi line detection** | | | | | | | | |
| Observer 1 vs. Observer 2 | 0.18 (-0.08-0.44) | Slight agreement | 0.22 | 0.04 | 0.38 (-0.16-0.91) | Fair agreement | 0.48 | <0.01 |
| Observer 1 vs. Observer 3 | 0.13 (-0.11-0.36) | Slight agreement | 0.13 | 0.23 | 0.21 (-0.13-0.55) | Fair agreement | 0.24 | 0.11 |
| Observer 2 vs. Observer 3 | 0.23 (-0.01-0.47) | Fair agreement | 0.31 | <0.01 | 0.17 (-0.12-0.45) | Slight agreement | 0.30 | 0.05 |

Observer 1, 2, 3 = glaucoma specialists.

PAS = peripheral anterior synechia; CI = confidence interval; NA = not applicable.

*The Landis-Koch score is used to interpret the kappa coefficient.
