# Supplementary material for: Intraobserver and interobserver agreement among anterior chamber angle evaluations using automated 360-degree gonio-photos
Source: PLoS One. 2021 May 6;16(5):e0251249. doi: 10.1371/journal.pone.0251249 (PMC8101769; doi:10.1371/journal.pone.0251249)
Supplement: Attachment — Submitted filename: Response to Reviewers.docx [file pone.0251249.s008.docx]

Ref: PONE-D-20-24626

Intraobserver and interobserver agreement among anterior chamber angle evaluations using automated 360-degree gonio-photos

PLOS ONE

Dear Dr. Huang,

We appreciate your reconsideration of our manuscript entitled “Intraobserver and interobserver agreement among anterior chamber angle evaluations using automated 360-degree gonio-photos” for publication in PLoS One as a Full Paper.

We disagree with some of the reviewer's comments, however we received constructive feedback. We have revised our manuscript and made a point-by-point response to the comments as follows. We have worked hard to incorporate your feedback and hope that these revisions persuade you to accept our submission.

We state that this manuscript has not been published elsewhere and is not under consideration by another journal. All authors have approved the manuscript and agree with submission to PLOS ONE. Thank you!

Best regards,

Masato Matsuo, MD, PhD

Department of Ophthalmology,

Shimane University Faculty of Medicine,

Enya 89-1, Izumo, Shimane, JAPAN.

mmpeaceful@yahoo.ne.jp

matsuondmc@gmail.com

**Journal Requirements:**

1. Please ensure that your manuscript meets PLOS ONE's style requirements, including those for file naming. The PLOS ONE style templates can be found at

https://journals.plos.org/plosone/s/file?id=wjVg/PLOSOne_formatting_sample_main_body.pdf and

https://journals.plos.org/plosone/s/file?id=ba62/PLOSOne_formatting_sample_title_authors_affiliations.pdf

Response

We have revised our manuscript to meet the PLOS ONE's style requirements.

2. Your ethics statement should only appear in the Methods section of your manuscript. If your ethics statement is written in any section besides the Methods, please delete it from any other section.

Response

We have deleted our ethics statement from our title page.

**Reviewer #1’s Comments:**

Introduction:
1) The first two paragraphs appear to be needlessly long: the authors could consider reshaping this to identify two succinct points: 1) angle closure as a disease entity is important to identify as it has a different prognostic course compared to open angle glaucoma; 2) secondary open angle (and closed angle) glaucomas require examination of the anterior chamber angle to identify underlying causes that may alter the treatment plan. Paragraph two in particular is a bit confusing in its tone with regard to the usefulness and limitations of gonioscopy. Moreover, there are some aspects of gonioscopy that are glossed over, such as the fact that grading systems for treatment titration rely solely on the gonioscopic impression (e.g. Prum Jr et al 2016 Ophthalmology), and that major clinical trials use gonioscopy as the technique of choice (He et al 2019 Lancet). Gonioscopy lenses such as the G6 in theory offer a more uninterrupted view of the anterior chamber angle so the statement on page 6, line 10 is not quite true.

Response

Thank you for the comment, however we disagree with you. According to the journal’s submission guideline, we tried to provide the background that puts the manuscript into context and allows readers outside the field to understand the purpose and significance of the study. The readers are not limited to glaucoma specialists or ophthalmologists. Therefore, it is necessary to define the problem addressed and explain why it is important.

Regarding 1), we also think early detection of a narrow angle and peripheral anterior synechia (PAS) is vital to prevent primary angle-closure glaucoma (PACG) as described in paragraph 1. However, we cannot agree with your idea that angle closure is important to identify as it has a different prognostic course compared to open angle glaucoma, because we sometimes experience cases of open-angle glaucoma (OAG) with end-stage visual field impairment at the first visit. If we could detect such patients early enough, we surely prevent the visual field loss due to OAG. Therefore, the early OAG detection and treatment is also clinically important.

As for 2), we agree with your comment that secondary glaucomas require examination of the anterior chamber angle to identify underlying causes that may alter the treatment plan. Thus, we have added the statement in the manuscript **L18-20 (pg 4)**. We believe that the change helps our manuscript more contextual. On the other hand, regarding the other indications, we do not agree with you. We pointed out the limitation of subjective assessments with gonioscopy in the manuscript **L12-13 (pg 5)**. Additionally, we also know the major clinical trials use gonioscopy as the technique of choice, however the fact has nothing to do with the reliability of the test. Moreover, “gonioscopy can examine at one time only a limited contiguous portion of the iridocorneal angle” means that we can observe only a limited position of the angle with conventional gonioscopy at one time even if using G6 because of the manual nature. On the other hand, gonioscopic camera can take whole angle images simultaneously. Therefore, we pointed out the characteristics and limitations of the gonioscopy in the manuscript.

2) Aside from the pigmentation grade, the configuration and distribution of pigment is important clinically, such as in cases of burnt out pigment dispersion syndrome, mottled versus homogenous pigmentation - there are numerous citations for this, such as from Rob Ritch.

Response

We also believe the importance of configuration and distribution of pigment; however, it is impractical to evaluate the diagnostic reproducibility of all angle findings. Pigment dispersion syndrome is clinically rare, and the evaluation is out of our main purpose. Moreover, it is difficult to evaluate the observer agreement of rare finding with small sample size practically. Therefore, we limited the evaluation items in our study.

Methods:
1) Why did the authors use Scheie classification in the first instance (page 8), followed by Shaffer (page 9)? Both are different and provide the opposite ordinal grades. This was unclear, but suggests that one was used by the clinician for the assessment and the other for the experimental design? Needs clarification as to the purpose of the grading system.

Response

Thank you for the comment. As you pointed out, the Scheie’s angle width grading system was used for the experimental design and the Shaffer’s grading system was for the clinical assessment as we described in the manuscript **L16-23 (pg 11)**. Moreover, following your suggestion, we have added the supplementary explanations for easy understanding in the manuscript **L18 (pg 7), L14-15 (pg 8).**

2) Page 10: the reader is slightly confused with the exclusion process... was the whole eye excluded or was it just one image per eye that was excluded - based on lines 14-16. How was the better quality image determined if both were equal? Were some angles systematically more likely to have better images? For example, if considering conventional imaging modalities, it is not surprising to find that superior angle assessment is more problematic compared to other quadrants due to anatomic lid interference (e.g. Xu et al 2019 TVST).

Response

Thank you for the comment. We paraphrased the word for clarity in the manuscript **L17 (pg 9).**
No images with same quality were found in both eyes. The image quality assessment was done only to pre-exclude images for which angle evaluation was not possible, which is not the main purpose of our study. Therefore, the further analysis and consideration are redundant and will blur the purpose and results.

3) A limitation of the GS-1, like AS-OCT is that it is performed in primary gaze without lens tilt, despite many clinical trials adding the dynamic component of gonioscopy (e.g. He et al 2019 Lancet) and clinical guidelines recommending identification of iridotrabecular contact that is only possible with lens tilt or off-axis gaze (e.g. Prum Jr et al 2016 Ophthalmology). This requires a comment on page 11.

Response

Thank you for the comment. We also agree with you as we described in paragraph 4 in our discussion. Moreover, according to your suggestion, we have changed the statement to make it easier for readers to understand in the manuscript **L8-9 (pg 10).**

4) The methods require a bit more transparency in the writing. Pages 12-13 for example appear particularly disorganized. The reader is introduced to five independent observers firstly on page 12, but then on page 13 line 3 onwards, a presumably singular observer is used for the intraobserver evaluation. However, then the authors state that a "second set" was used to further evaluate intraobserver agreements presumably for the five independent observers on line 16? Another example is page 13 line 13 - are the different randomized images from the same set or another prospectively collected set? The writing needs further clarification and would perhaps benefit from separation into subheadings that allow clarity in the methods.

Response

Thank you for the comment. Following your advice, we have made the writing separated into subheadings and added the supplementary explanations in our manuscript. Please see page 11 to 12.

Results
1) There are a few issues regarding the presentation of Table 1. The authors did not seem to state the techniques used to measure many of the continuous variables reported in the table. Further to Table 1, why was pseudoexfoliative glaucoma and secondary open angle glaucoma separated? The reporting of the distribution of Shaffer's angle width but Scheie's angle pigmentation grade represents to the reader unnecessary differentiation and muddling of the grading schemes - why not just stick to one? Further, it could be argued that the reading of a mean and SD for these grades is questionable, due to their non-linearity and ordinal nature (see Phu et al 2020 OPO)... it may be more useful to break down the distribution of the exact numbers of each grade, especially since the Fleiss's kappa was used. Finally, the additive value of topical glaucoma medications is questionable: the paper is not really reporting on the contribution of these to angle grading and the hypothesis being tested is unlikely to be confounded by these factors. It would also be more informative to provide the proportion of poor image grades that were subsequently excluded from analysis in this table or in the results text. This would reinforce the authors' choice of a pragmatic approach and a more realistic impression of the deployment of GS-1.

Response

Thank you for the comment. Table 1 is only the demographics and clinical characteristics of the study subjects and stating all the measurement technique seems to be unnecessary and verbose. Regarding the second point, as we described in our introduction, pseudoexfoliation glaucoma is considered to be the most common type of secondary glaucoma and can advance rapidly with continuous high IOP and be refractory to several therapeutic interventions. Therefore, it is clinically important, and we analyzed pseudoexfoliation glaucoma separately. As for the third point, please see the above response for Methods 1). As for the fourth point, we disagree with you. The mean and SD have been used in the clinical gradings and was also used in some recent research for the angle evaluations with gonio-photos (see Teixeira et al., *Eur J Ophthalmol.* 2018, Matsuo et al, *Br J Ophthalmol*. 2019). Breaking down the distribution of the exact numbers of each grade would only make the manuscript unnecessarily redundant and obscure the purpose and results of this study. As for the final point, we can understand your meaning, however we cannot agree with you. This study already contains more than enough information to make a single treatise, and any further additions will not only unnecessarily obscure the meaning but will also lose sight of its original purpose. Again, Table 1 was just the demographics and clinical characteristics of the study subjects. Please understand the main focus of our research.

2) With regard to reporting Table 2 and the kappa values, it is unclear to the reader why Fleiss's kappa was used for binary variables (PAS and Sampaolesi line - present/absent). This confusion stems from the lack of contingency tables being presented in the results and the confusing methods. The authors should seek to clarify the most appropriate statistical method used. Fleiss's kappa may be appropriate for agreement between the observers but from the writing it is hard to say what was compared.

Response

We are sorry to mention that we disagree with you. Table 2 clearly shows the intraobserver reproducibility for angle evaluations between manual gonioscopy and automated gonioscope by one observer (MT). Because we cannot compare results analyzed by different statistical methods, we calculated both Fleiss' kappa coefficient and Kendall rank correlation coefficient for grading (nominal) and binary scales at the same time. Additionally, the Fleiss' kappa analysis in Table 2 is confirmation of the previous report (see Teixeira et al., *Eur J Ophthalmol.* 2018).

3) The reporting of closed angle detection is somewhat unclear and strikes the reader as somewhat misleading and requires clarification in writing. Not all of the 140 quadrants were closed, and so the question is whether the kappa was related to the binary outcome of "open" vs. "closed", or the agreement between observers (as noted above).

Response

Thank you for the query. As we described in the methods, we analyzed the intraobserver reproducibility for closed angle detection in each quadrant (n=140) and in each eye (n=35) comparing the outcomes with manual gonioscopy and those with automated gonioscope by one observer. Therefore, the kappa was related to the binary outcomes of "open" vs. "closed".

4) Page 17, line 14 refers to observers 1-5 and glaucoma specialists 1-3, but this was somewhat unclear from the methods as well.

Response

We described it in our manuscript. Please see **L14-16 (pg 11)**.

5) Table 3 (and similar): there was extreme variability in the kappa values across quadrants and across observers: could the authors comment on whether there was a systematic difference or random difference in the text? This is a very interesting result and the reader may be tempted to think it may be related to the distribution of angle grades, whereby some may be more obvious and easy to score (e.g. closed or very wide open angles) compared to the middling grades.

Response

Thank you for the comment. There would be the tendency that the intraobserver agreement values of the glaucoma specialist with enough experience using the GS-1 in the clinic were higher than those of the other ophthalmologists as described in the manuscript. On the other hand, the extreme variability in the kappa values across quadrants seems to be happened by chance. However, we cannot conclude it on a clear evidence because of the small sample size. Moreover, they deviate from the original purpose, thus we would like to leave them to the future large-scale research.

6) Overall, comments 3-5 above suggest that contingency tables may play a role in the reporting of the data

Response

We do not agree with you. Again, adding more data is redundant.

Discussion
1) The second paragraph of the discussion is confusing, and the reader is unsure what the authors are trying to say. The tone is confusing in whether 10% is a high number or a low number?

Response

Thank you for the advice. We have revised our manuscript to make it easier to understand for readers in the second paragraph.

2) With regard to objectivity, one questions the value of precise grading given the wealth of data required to change the management plan. For example, the decision to treat would be based on a multitude of factors aside from the angle appearance, including accessibility to health care, age, lens status, epidemiological risk factors and others (see Thomas and Walland 2013 CEO).

Response

We disagree with you. All the angle parameters in our study can be objectively evaluated based on the angle findings, and it is worthwhile to examine the accurate inter- and intra-observer agreements for angle evaluations only with the novel device in considering the reliability. Accurate angle assessments and clinical judgments based on it are separate issues, and how to apply them to each patient should be considered in the other future study.

3) Page 27, lines 6-11: with respect to the discussion on gonio photographs, there is a limitation previously noted in the literature that the authors seemed to have omitted: the fact that many of these are conducted in primary gaze, and lack the dynamism of manual gonioscopy, which is really the key advantage of the technique. This requires further discussion.

Response

Thank you for the comment. We described it in the manuscript **L22-23 (pg 27), L1-3 (pg 28)**. Moreover, we have added the sentence to make it easier to understand in the discussion, **L5-7 (pg 26)**.

4) A general question with regards to the comparison with the EyeCam, the light source for imaging and photography should be mentioned or discussed at some point. There are significant efforts taken in standardizing gonioscopy as a procedure to mitigate introduction of stray light through the pupil.

Response

Thank you for the query. According to your suggestion, we have added the related information in the manuscript, **L12-16 (pg 27)**.

5) Another general comment with the discussion: there is far too much re-reporting of the results and this causes bloat in the discussion. The authors should revise this for succinctness.

Response

Thank you for the advice. We have revised and shortened the relevant part in our discussion.

6) The issue of angle grade distribution was mentioned on page 31, but the analysis seems to be lacking in terms of accounting for it as a confounding factor.

Response

We do not agree with you. We assessed the effects of image quality on observer agreements for angle evaluations using gonioscopic photos of GS-1. As the result, the observer agreements using grade 0 images were not always better than those using grade 1 images, probably because we completely excluded the grade 2 (blurred with no discernible details) images. Therefore, it is unlikely that the differences in image quality can be the confounding factor, and such detailed examinations that deviate from the original purpose are redundant and we would like to leave it to the other future study.

7) Another limitation of the grading system for image quality, which should also be noted in the references cited by the authors, is that the entirety of the image needs not be clear for evaluation. This does not seem to be captured with this method and should be discussed.

Response

We disagree with you. Same as on the previous reports, we assessed the image quality of the gonio-images and excluded the blurred images with no discernible details, which means all the unevaluable images was eliminated. You might be worried about the slightly vague but determinable images that were included in grade 1 by our definition, which we have already discussed in the above response to comment 6).

8) The authors raise an interesting point about the learning curve and the value of experience. This was discussed by Phu and colleagues and may be worthwhile integrated as part of this discussion (Phu et al 2019 OVS). What they may consider also acknowledging is the acquisition step. Though the processes described in the methods appear to be largely automated, there may be some component of interpreting whether the image is useable. A pragmatic approach as described by the authors would necessitate consideration of practical aspects such as the limitations and barriers to successful image acquisition and whether there are systematic errors in that process. A consecutive sampling strategy would be necessary as well as reporting of the errors associated with the technique. Finally, a calibration exercise would be suitable for this particular purpose.

Response

We disagree with your comments because they are out of our main focus of the study. The primary purpose was to analyze the reproducibility for the angle evaluations with the newly released gonioscopic camera, and it was the first verification to investigate to what extent glaucoma specialists and ophthalmologists made the same angle evaluations using the standardized gonio-images. As a result of the research, we could draw some important conclusions.

First of all, the recommended reference study by Phu et al was quite different from ours in the purpose, study design and methods. It was conducted with the medical records of gonioscopy results among different practitioners retrospectively, which must have full of bias including the observer bias to know the specific clinical information about the patients. Therefore, the result would be quite different from the pure angle evaluation and it is tough to draw the conclusion for the learning curve and the value of experience, and we would not like to make it reference.

With regard to the second point, as we described in the methods, we defined the exclusion criteria as the whole eyes with poor-quality images that had at least one grade 2 image in four ocular sectors, and finally 17 poor-quality eyes (23.9%) were excluded from angle evaluations. On the other hand, in the image acquisition with prototype GS-1, Teixeira et al reported that 22.7% eyes were excluded from the angle image grading as information would be lacking concerning at least two quadrants, and Shi et al demonstrated that 8.33% sections were not gradable owing to poor image quality. These studies’ exclusion criteria, conditions, and version of GS-1 were different from ours, thus we could not compare them directly, however, our exclusion rate was quite near to that of Teixeira’s. The reviewer seemed to stick to the further analysis for the limitations and barriers to successful image acquisition and whether there are systematic errors in the GS-1. However, the study already contains more than enough information to make a single manuscript, and the result is enough for our conclusions. A further detailed examination or analysis for the reviewer’s point is out of our main purpose and redundant.

Regarding the final points, the reviewer seemed to be misunderstanding that we did not conduct the consecutive sampling and calibration exercise, which were successfully done in the study. In fact, we did the consecutive sampling, therefore, we could demonstrate the successful image acquisition rate. On the other hand, the poor-quality GS-1 image eyes were excluded in the image selection process. Thus, strictly speaking, it was not intended for all consecutive patients who underwent GS-1, and we refrained from describing it. Moreover, we had several years of experience using GS-1 since it was prototyped (Matsuo et al., *Br J Ophthalmol.* 2019), so the calibration exercise in the image acquisition would be sufficient.

Minor comments
- Page 12, line 9-10: please clarify that the participants are the ophthalmologists

Response

Thank you for the comment. We have paraphrased the word for clarity in the manuscript **L11-12 (pg 11).**

- Page 13, line 22: nominal or ordinal?

Response

Nominal is correct. The analysis was performed by regarding the grading scale as the nominal scale to compare the results with each other.

- Table 1: Dioptor should be "Diopter"

Response

Thank you for making the typo. We have revised the word in Table 1.

- There is some inconsistency between "sectors" and "quadrants" used throughout the manuscript

Response

According to your advice, we have unified the word to sector.

- Page 25: there is some inconsistency in the way that the results are reported in the text. It is quite wordy and somewhat repetitive from the tables. The authors should note this trend throughout the manuscript and seek to clarify and write succinctly.

Response

The suggestion is same as the reviewer’s comment in Discussion 5). According to your advice, we have shortened and simplified the relevant part.

- Page 25, line 15: there should be a clearer distinction between an ophthalmologist (general?) and glaucoma specialist

Response

What part were you pointing out? We did not know the relevant part.

- Why were there "NA" results in Supp Table 1 - was not clear

Response

In our study, the analyses for Fleiss’ kappa statistic were performed in R statistical software version 3.5.3. and the calculations for Kendall rank correlation coefficients were conducted with JMP Pro 14 software. Because we are not the developer of those software, we do not know the exact answer. However, the discussion about it for S1 Fig is out of the main focus of the study and redundant.

- The units on Figures 1 and 2 are not clearly described in the Figure Captions. Although somewhat intuitive from the text, this may deserve further clarification.

Response

Thank you for your suggestion. We have added the supplementary explanation in the Figure legends.
