# Supplementary material for: Intraobserver and interobserver agreement among anterior chamber angle evaluations using automated 360-degree gonio-photos
Source: PLoS One. 2021 May 6;16(5):e0251249. doi: 10.1371/journal.pone.0251249 (PMC8101769; doi:10.1371/journal.pone.0251249)
Supplement: Attachment — Submitted filename: Response to Reviewers 2nd revision.docx [file pone.0251249.s009.docx]

``````	March 3, 2020

Jinhai Huang, M.D.
Academic Editor
PLOS ONE

Dear Dr. Huang,

Ref: PONE-D-20-24626

Intraobserver and interobserver agreement among anterior chamber angle evaluations using automated 360-degree gonio-photos

PLOS ONE

We appreciate your reconsideration of our manuscript entitled “Intraobserver and interobserver agreement among anterior chamber angle evaluations using automated 360-degree gonio-photos” for publication in PLOS ONE as a Full Paper.

We received constructive feedback. We have revised our manuscript and made a point-by-point response to the comments as follows. We have worked hard to incorporate your feedback and hope that these revisions persuade you to accept our submission.

We state that this manuscript has not been published elsewhere and is not under consideration by another journal. All authors have approved the manuscript and agree with submission to PLOS ONE. Thank you!

Best regards,

Masato Matsuo, MD, PhD

Department of Ophthalmology,

Shimane University Faculty of Medicine,

Enya 89-1, Izumo, Shimane, JAPAN.

mmpeaceful@yahoo.ne.jp

matsuondmc@gmail.com

**Journal Requirements:**

Please review your reference list to ensure that it is complete and correct. If you have cited papers that have been retracted, please include the rationale for doing so in the manuscript text, or remove these references and replace them with relevant current references. Any changes to the reference list should be mentioned in the rebuttal letter that accompanies your revised manuscript. If you need to cite a retracted article, indicate the article’s retracted status in the References list and also include a citation and full reference for the retraction notice.

Response

We have checked our reference list to ensure that it is complete and correct.

**Reviewer #1’s Comments:**

1) Reviewer‘s comment:

The first two paragraphs appear to be needlessly long: the authors could consider reshaping this to identify two succinct points: 1) angle closure as a disease entity is important to identify as it has a different prognostic course compared to open angle glaucoma; 2) secondary open angle (and closed angle) glaucomas require examination of the anterior chamber angle to identify underlying causes that may alter the treatment plan. Paragraph two in particular is a bit confusing in its tone with regard to the usefulness and limitations of gonioscopy. Moreover, there are some aspects of gonioscopy that are glossed over, such as the fact that grading systems for treatment titration rely solely on the gonioscopic impression (e.g. Prum Jr et al 2016 Ophthalmology), and that major clinical trials use gonioscopy as the technique of choice (He et al 2019 Lancet). Gonioscopy lenses such as the G6 in theory offer a more uninterrupted view of the anterior chamber angle so the statement on page 6, line 10 is not quite true.

Authors' comment:

Thank you for the comment, however we disagree with you. According to the journal’s submission guideline, we tried to provide the background that puts the manuscript into context and allows readers outside the field to understand the purpose and significance of the study. The readers are not limited to glaucoma specialists or ophthalmologists. Therefore, it is necessary to define the problem addressed and explain why it is important.

Regarding 1), we also think early detection of a narrow angle and peripheral anterior synechia (PAS) is vital to prevent primary angle-closure glaucoma (PACG) as described in paragraph 1. However, we cannot agree with your idea that angle closure is important to identify as it has a different prognostic course compared to open angle glaucoma, because we sometimes experience cases of open-angle glaucoma (OAG) with end-stage visual field impairment at the first visit. If we could detect such patients early enough, we surely prevent the visual field loss due to OAG. Therefore, the early OAG detection and treatment is also clinically important.

As for 2), we agree with your comment that secondary glaucomas require examination of the anterior chamber angle to identify underlying causes that may alter the treatment plan. Thus, we have added the statement in the manuscript L18-20 (pg 4). We believe that the change helps our manuscript more contextual. On the other hand, regarding the other indications, we do not agree with you. We pointed out the limitation of subjective assessments with gonioscopy in the manuscript L12-13 (pg 5). Additionally, we also know the major clinical trials use gonioscopy as the technique of choice, however the fact has nothing to do with the reliability of the test. Moreover, “gonioscopy can examine at one time only a limited contiguous portion of the iridocorneal angle” means that we can observe only a limited position of the angle with conventional gonioscopy at one time even if using G6 because of the manual nature. On the other hand, gonioscopic camera can take whole angle images simultaneously. Therefore, we pointed out the characteristics and limitations of the gonioscopy in the manuscript.

Reviewer’s comment:

Whilst this is true (e.g. the work of Boodhna, Crabb and colleagues), the point of the paper does not appear to be related to POAG. The concept of making an introduction succinct is to engage with the reader in order to arrive at the core purpose of the study. I suggest that the authors use a new paragraph at page 4 line 20, beginning with the sentence "While" to disconnect POAG and PACG. On that point, whilst PLOS is a general science journal, the readership of a paper reporting on the outcomes of a highly specialised ophthalmic tool is most likely someone in the field.

Also, if the authors wish to describe the spectrum of angle closure disease at such length and comprehensiveness, it would be remiss of them to exclude the continuum between open and closed angles, as the prevention stage is touted by some to occur well before vision loss.

Response

Thank you for the comment. POAG is the most common type, and the condition is associated with an open anterior chamber angle without other known explanations (i.e., secondary glaucoma) for progressive glaucomatous optic nerve change. Thus, gonioscopic angle assessment is also essential for the management and our study is related to POAG. On the other hand, we have decided to add the supplementary explanations and divided into the paragraphs for easy understanding for the readers according to your suggestion in the manuscript **L9-12 (pg 4), L1, L4-10 (pg 5).**

2) Reviewer’s comment:

Aside from the pigmentation grade, the configuration and distribution of pigment is important clinically, such as in cases of burnt out pigment dispersion syndrome, mottled versus homogenous pigmentation - there are numerous citations for this, such as from Rob Ritch.

Authors' comment:

We also believe the importance of configuration and distribution of pigment; however, it is impractical to evaluate the diagnostic reproducibility of all angle findings. Pigment dispersion syndrome is clinically rare, and the evaluation is out of our main purpose. Moreover, it is difficult to evaluate the observer agreement of rare finding with small sample size practically. Therefore, we limited the evaluation items in our study.

Reviewer’s comment:

There appears to be no statement or change made describing this limitation in the study - or if there is, it has not been clearly noted by the authors.

Response

Thank you for the comment. We described the limitation in the manuscript **L22-23 (pg 35), L1 (pg 36).** Moreover, for better understanding, we have generated the additional ideas to supplement in the manuscript **L2-3 (pg 36).**

3) Reviewer’s comment:

There are a few issues regarding the presentation of Table 1. The authors did not seem to state the techniques used to measure many of the continuous variables reported in the table. Further to Table 1, why was pseudoexfoliative glaucoma and secondary open angle glaucoma separated? The reporting of the distribution of Shaffer's angle width but Scheie's angle pigmentation grade represents to the reader unnecessary differentiation and muddling of the grading schemes - why not just stick to one? Further, it could be argued that the reading of a mean and SD for these grades is questionable, due to their non-linearity and ordinal nature (see Phu et al 2020 OPO)... it may be more useful to break down the distribution of the exact numbers of each grade, especially since the Fleiss's kappa was used. Finally, the additive value of topical glaucoma medications is questionable: the paper is not really reporting on the contribution of these to angle grading and the hypothesis being tested is unlikely to be confounded by these factors. It would also be more informative to provide the proportion of poor image grades that were subsequently excluded from analysis in this table or in the results text. This would reinforce the authors' choice of a pragmatic approach and a more realistic impression of the deployment of GS-1.

Authors' comment:

Thank you for the comment. Table 1 is only the demographics and clinical characteristics of the study subjects and stating all the measurement technique seems to be unnecessary and verbose. Regarding the second point, as we described in our introduction, pseudoexfoliation glaucoma is considered to be the most common type of secondary glaucoma and can advance rapidly with continuous high IOP and be refractory to several therapeutic interventions. Therefore, it is clinically important, and we analyzed pseudoexfoliation glaucoma separately. As for the third point, please see the above response for Methods 1). As for the fourth point, we disagree with you. The mean and SD have been used in the clinical gradings and was also used in some recent research for the angle evaluations with gonio-photos (see Teixeira et al., *Eur J Ophthalmol.* 2018, Matsuo et al, *Br J Ophthalmol*. 2019). Breaking down the distribution of the exact numbers of each grade would only make the manuscript unnecessarily redundant and obscure the purpose and results of this study. As for the final point, we can understand your meaning, however we cannot agree with you. This study already contains more than enough information to make a single treatise, and any further additions will not only unnecessarily obscure the meaning but will also lose sight of its original purpose. Again, Table 1 was just the demographics and clinical characteristics of the study subjects. Please understand the main focus of our research.

Reviewer's comment:

This sounds almost contradictory. On one hand, the authors are willing to retain information on topical glaucoma medications even though its value has not been described anywhere in the text, nor is it immediately obvious to the reader - even a glaucoma expert - that its contribution is, even though the authors state it is a "main focus of their research". On the other hand, the contribution of angle grades on repeatability indices has more obvious value, but the authors have not provided justification on why it has been excluded.

Response

Thank you for the comment. It seems to be misunderstood. As we described in our previous response, information on topical glaucoma medications in Table 1 is only the demographics and clinical characteristics of the study subjects. We believe it is okay to keep it, however we would like to remove it if it interferes with the reader's understanding. Additionally, we found the typographical error in Table 1, and we have corrected it. Please see the underlined part. Moreover, according to your suggestion, we have added the contingency table demonstrating the distribution of angle grades in S1 Table.

4) Reviewers' comment:

With regard to reporting Table 2 and the kappa values, it is unclear to the reader why Fleiss's kappa was used for binary variables (PAS and Sampaolesi line - present/absent). This confusion stems from the lack of contingency tables being presented in the results and the confusing methods. The authors should seek to clarify the most appropriate statistical method used. Fleiss's kappa may be appropriate for agreement between the observers but from the writing it is hard to say what was compared.

Authors' comment:

We are sorry to mention that we disagree with you. Table 2 clearly shows the intraobserver reproducibility for angle evaluations between manual gonioscopy and automated gonioscope by one observer (MT). Because we cannot compare results analyzed by different statistical methods, we calculated both Fleiss' kappa coefficient and Kendall rank correlation coefficient for grading (nominal) and binary scales at the same time. Additionally, the Fleiss' kappa analysis in Table 2 is confirmation of the previous report (see Teixeira et al., Eur J Ophthalmol. 2018).

Reviewers' comment:

My point relates to comment 3 above - if you had the contingency table available, it would provide a more useful interpretation of repeatability at different angle grades. Kappa values are a useful statistical tool for comparison with previous studies - this is true - however if the authors are aiming for a pragmatic interpretation of the results, then kappa values become more subjective.

Response

Thank you for the suggestion. We have added the contingency tables in Supplemental material (S1 Table).

5) Reviewers' comment:

Page 17, line 14 refers to observers 1-5 and glaucoma specialists 1-3, but this was somewhat unclear from the methods as well.

Authors' comment:

We described it in our manuscript. Please see L14-16 (pg 11).

Reviewer’s comment:

this is written in an ambiguous manner, as it can be interpreted as 5 + 3 examiners, when it is 5, within which 3 were glaucoma specialists. This should be rephrased for clarity throughout the manuscript.

Response

Thank you for the suggestion. As you pointed out, we have paraphrased the relevant part and added the supplemental explanation in the manuscript **L18-19 (pg 7), L2 (pg 20), L2 (pg 21).**

6) Reviewer’s comment:

Table 3 (and similar): there was extreme variability in the kappa values across quadrants and across observers: could the authors comment on whether there was a systematic difference or random difference in the text? This is a very interesting result and the reader may be tempted to think it may be related to the distribution of angle grades, whereby some may be more obvious and easy to score (e.g. closed or very wide open angles) compared to the middling grades.

and

Overall, comments 3-5 above suggest that contingency tables may play a role in the reporting of the data

Authors' comment:

Thank you for the comment. There would be the tendency that the intraobserver agreement values of the glaucoma specialist with enough experience using the GS-1 in the clinic were higher than those of the other ophthalmologists as described in the manuscript. On the other hand, the extreme variability in the kappa values across quadrants seems to be happened by chance. However, we cannot conclude it on a clear evidence because of the small sample size. Moreover, they deviate from the original purpose, thus we would like to leave them to the future large-scale research.

and

We do not agree with you. Again, adding more data is redundant.

Reviewer’s comment:

Unfortunately, I disagree with these points. It is not redundant because it plans an important role in data visualization and potentially to a less biased interpretation of the results.

Response

Thank you for the suggestion. We have added the contingency tables in Supplemental materials (S2 Table and S3 Table).

7) Reviewer’s comment:

With regard to objectivity, one questions the value of precise grading given the wealth of data required to change the management plan. For example, the decision to treat would be based on a multitude of factors aside from the angle appearance, including accessibility to health care, age, lens status, epidemiological risk factors and others (see Thomas and Walland 2013 CEO).

Authors' comment:

We disagree with you. All the angle parameters in our study can be objectively evaluated based on the angle findings, and it is worthwhile to examine the accurate inter- and intra-observer agreements for angle evaluations only with the novel device in considering the reliability. Accurate angle assessments and clinical judgments based on it are separate issues, and how to apply them to each patient should be considered in the other future study.

Reviewer’s comment:

Unfortunately, I also disagree with the conviction of the authors here and their statement that they are fully separate issues. It is important to remain skeptical of emphasising objective measures too much, and the authors have too quickly dismissed their contribution into a more holistic model of health care.

Response:

Thank you for the suggestion. We have added the contingency tables in Supplemental materials (S2 Table and S3 Table).

8) Reviewer’s comment:

The issue of angle grade distribution was mentioned on page 31, but the analysis seems to be lacking in terms of accounting for it as a confounding factor.

Authors' comment:

We do not agree with you. We assessed the effects of image quality on observer agreements for angle evaluations using gonioscopic photos of GS-1. As the result, the observer agreements using grade 0 images were not always better than those using grade 1 images, probably because we completely excluded the grade 2 (blurred with no discernible details) images. Therefore, it is unlikely that the differences in image quality can be the confounding factor, and such detailed examinations that deviate from the original purpose are redundant and we would like to leave it to the other future study.

Reviewer’s comment:

I have noted that the authors appear to say a lot of things are redundant and deviate from the original purpose. I disagree with the notion that image quality - such an important component of any imaging based study - is redundant. As a reader, that is one of the first things one would question. The authors could consider at least reporting this in the supplementary material.

Response

Thank you for the comment. Interpreting our results, we believe it is unlikely that the differences in image quality could be the confounding factor when grade 2 images were excluded. Moreover, as far as we know, there is no statistical method to adjust the confounding factors to evaluate the Fleiss’ kappa value.
